# Intestinal dysbiosis in preterm infants preceding necrotizing enterocolitis: a systematic review and meta-analysis

**DOI:** 10.1186/s40168-017-0248-8

**Published:** 2017-03-09

**Authors:** Mohan Pammi, Julia Cope, Phillip I. Tarr, Barbara B. Warner, Ardythe L. Morrow, Volker Mai, Katherine E. Gregory, J. Simon Kroll, Valerie McMurtry, Michael J Ferris, Lars Engstrand, Helene Engstrand Lilja, Emily B. Hollister, James Versalovic, Josef Neu

**Affiliations:** 1 Section of Neonatology, Department of Pediatrics, Baylor College of Medicine and Texas Children’s Hospital, 77030 Houston, TX USA; 2 Alkek Center for Metagenomics and Microbiome Research, Baylor College of Medicine, Houston, TX USA; 3 Department of Pediatrics, Washington University in St. Louis School of Medicine, St. Louis, MO USA; 4 Department of Pediatrics, Perinatal Institute, Cincinnati Children’s Hospital Medical Center, University of Cincinnati College of Medicine, Ohio, USA; 5 Department of Epidemiology, College of Public Health and Health Professions and College of Medicine and Emerging Pathogens Institute, University of Florida, Gainesville, FL USA; 6 Department of Newborn Medicine, Brigham and Women’s Hospital, Boston, MA USA; 7 Department of Medicine, Section of Paediatrics, Imperial College London, London, UK; 8 Department of Microbiology, Immunology and Parasitology, Children’s Hospital, New Orleans, LA USA; 9 Director of Clinical Genomics and Department of Microbiology, Tumor and Cell Biology, Karolinska Institute, Stockholm, Sweden; 10 Department of Women’s and Children’s Health, Uppsala University, 751 85 Uppsala, Sweden; 11 Texas Children’s Microbiome Center, Department of Pathology, Texas Children’s Hospital and Baylor College of Medicine, Houston, TX USA

**Keywords:** Microbiome, Intestinal, Preterm, Neonate, NEC, 16S rRNA sequencing

## Abstract

**Background:**

Necrotizing enterocolitis (NEC) is a catastrophic disease of preterm infants, and microbial dysbiosis has been implicated in its pathogenesis. Studies evaluating the microbiome in NEC and preterm infants lack power and have reported inconsistent results.

**Methods and results:**

Our objectives were to perform a systematic review and meta-analyses of stool microbiome profiles in preterm infants to discern and describe microbial dysbiosis prior to the onset of NEC and to explore heterogeneity among studies. We searched MEDLINE, PubMed, CINAHL, and conference abstracts from the proceedings of Pediatric Academic Societies and reference lists of relevant identified articles in April 2016. Studies comparing the intestinal microbiome in preterm infants who developed NEC to those of controls, using culture-independent molecular techniques and reported α and β-diversity metrics, and microbial profiles were included. In addition, 16S ribosomal ribonucleic acid (rRNA) sequence data with clinical meta-data were requested from the authors of included studies or searched in public data repositories. We reprocessed the 16S rRNA sequence data through a uniform analysis pipeline, which were then synthesized by meta-analysis.

We included 14 studies in this review, and data from eight studies were available for quantitative synthesis (106 NEC cases, 278 controls, 2944 samples). The age of NEC onset was at a mean ± SD of 30.1 ± 2.4 weeks post-conception (*n* = 61). Fecal microbiome from preterm infants with NEC had increased relative abundances of *Proteobacteria* and decreased relative abundances of *Firmicutes* and *Bacteroidetes* prior to NEC onset. Alpha- or beta-diversity indices in preterm infants with NEC were not consistently different from controls, but we found differences in taxonomic profiles related to antibiotic exposure, formula feeding, and mode of delivery. Exploring heterogeneity revealed differences in microbial profiles by study and the target region of the 16S rRNA gene (V1-V3 or V3-V5).

**Conclusions:**

Microbial dysbiosis preceding NEC in preterm infants is characterized by increased relative abundances of *Proteobacteria* and decreased relative abundances of *Firmicutes* and *Bacteroidetes*. Microbiome optimization may provide a novel strategy for preventing NEC.

**Electronic supplementary material:**

The online version of this article (doi:10.1186/s40168-017-0248-8) contains supplementary material, which is available to authorized users.

## Background

Necrotizing enterocolitis (NEC) is a catastrophic disease that is a major cause of mortality in preterm infants who survive the first few days after birth [1]. NEC occurs in 7% of infants born at less than 1500 g and up to 5% of admissions to the neonatal intensive care unit [2–5]. NEC is associated with a high mortality (15–30%) and long-term neurodevelopmental morbidity [2, 6].

The pathogenesis of NEC is not clear, and a unifying concept is lacking but microbial dysbiosis, formula feeding, and excessive inflammation have all been implicated [2, 7–9]. Compared to term infants, the intestinal microbiota of preterm infants has fewer bacterial species, less diversity, and increased proportions of potential pathogens [10, 11]. The microbial dysbiosis hypothesis of NEC is supported by the fact that NEC cannot be produced in germ free animals [12, 13] and by an association between early antibiotic use and NEC [14, 15]. Immune dysregulation in association with microbial dysbiosis including excessive toll-like receptor 4 (TLR4) signaling in response to lipopolysaccharide (LPS) [12, 16–18] and an exaggerated inflammatory response [8, 9] have been reported, but these are largely animal data.

Technological advances and availability of new molecular and analytic techniques such as those used in Human Microbiome Project [19, 20] have provided greater resolution in the evaluation of the neonatal intestinal microbiome. Most studies on the human microbiome do not have sufficient power to detect clinically important differences [21]. Previously reported microbiome studies on NEC in preterm infants may have been underpowered to detect differences and have reported inconsistent results. We systematically reviewed studies that reported the intestinal microbiome in preterm infants who developed NEC in relation to controls and performed a meta-analysis of 16S ribosomal ribonucleic acid (rRNA) gene sequence data from eight of the included studies. To our knowledge, this is the first systematic review and meta-analysis of microbiome studies on NEC in preterm infants.

## Objectives

The primary objective of this review is to determine and describe intestinal microbial dysbiosis patterns in preterm infants preceding NEC (defined as stage II or stage III of Bell’s classification [22]). The secondary objective is to explore heterogeneity among studies that might explain inconsistency in the reported results.

## Methods

We performed our systematic review according to the recommended “Preferred Reporting Items for Systematic Reviews and Meta-analyses” (PRISMA) guidelines [23] and “Meta-analysis of Observational Studies in Epidemiology” (MOOSE) consensus statement [24]. We followed previously published methods for the meta-analyses of microbiome data [25].

### Inclusion criteria

Prospective or retrospective, case-control or cohort studies were included if they evaluated the neonatal intestinal microbiome in preterm infants with NEC compared to those infants without NEC, using culture-independent molecular techniques and reported α and β-diversity metrics and microbial profiles.

We searched for eligible studies using the Cochrane Neonatal Review Group’s (CNRG) search strategy (http://neonatal.cochrane.org/) without language restriction in April 2016. Search strategy and databases searched are outlined in Additional file 1.

### Data collection and analyses

All titles and abstracts identified by our search strategy were screened for relevance by MP, and those deemed relevant were retrieved in full and evaluated for inclusion eligibility by MP and JN independently. All results were compared, and disagreements were resolved by mutual discussion. Relevant data were extracted from included studies and additional information to clarify the study design, and data was sought from the authors via email, for at least three attempts. The data extracted by the author were discussed for any discrepancies by input from a second author JN, and any conflicts were resolved by mutual discussion. In addition, 16S rRNA gene sequence data with clinical meta-data were requested from the principal investigators of the published studies. The methodological quality of each study was assessed using relevant items of the checklist proposed for observational studies by Viswanathan et al. [26].

## Meta-analysis of microbiome data

We compiled all sequence files and subject phenotype data received from the investigators or downloaded from the National Center for Biotechnology Information Short Read Archive (NCBI SRA), Database of Genotypes and Phenotypes (NCBI dbGaP), and/or the European Nucleotide Archive (ENA) (accessed 06/27/2015). All sequences were processed using Quantitative Insights into Microbial Ecology package (QIIME 1.8.0) [25, 27]. Quality parameters of ≥25 average quality score, 200–1000 base length, no mismatches to barcode and primer, primer removal, no ambiguous bases, and maximum homopolymer length of 8 were used. As there were several 16S variable regions targeted, operational taxonomic units (OTUs) were assigned by closed-reference picking against the GreenGenes v13_8 database with the UCLUST algorithm in QIIME [28, 29]. We removed all samples that failed to yield at least 1200 sequences, resulting in the exclusion of one complete study [30] due to shallow sequencing depth. All analyses were performed on the rarefied data.

We derived the corrected gestational age (CGA) at diagnosis of NEC by adding gestational age at birth to the day of diagnosis of NEC for 61 individual cases of NEC. The distribution of NEC diagnoses in CGA (weeks) was tested for a continuous normal distribution with the Shapiro-Wilk method.

QIIME was used to calculate alpha diversity metrics, including observed OTUs, and the Shannon and Simpson diversity indices. Differences in alpha diversity metrics were tested with Mann-Whitney or Kruskal-Wallis tests, depending on the number of groups being compared, and Dunn’s multiple comparison testing was implemented for those with significant differences. We also utilized a negative binomial regression model to evaluate the degree to which number of species (our dependent variable) could be explained by gestational age, group (NEC vs. control), and a gestational age by group interaction term.

The weighted and unweighted UniFrac metrics were used for beta diversity comparisons. Differences in taxonomic relative abundance were evaluated with Mann-Whitney or Kruskal-Wallis tests and Benjamini-Hochberg false discovery rate correction.

## Results

**Fig. 1:**
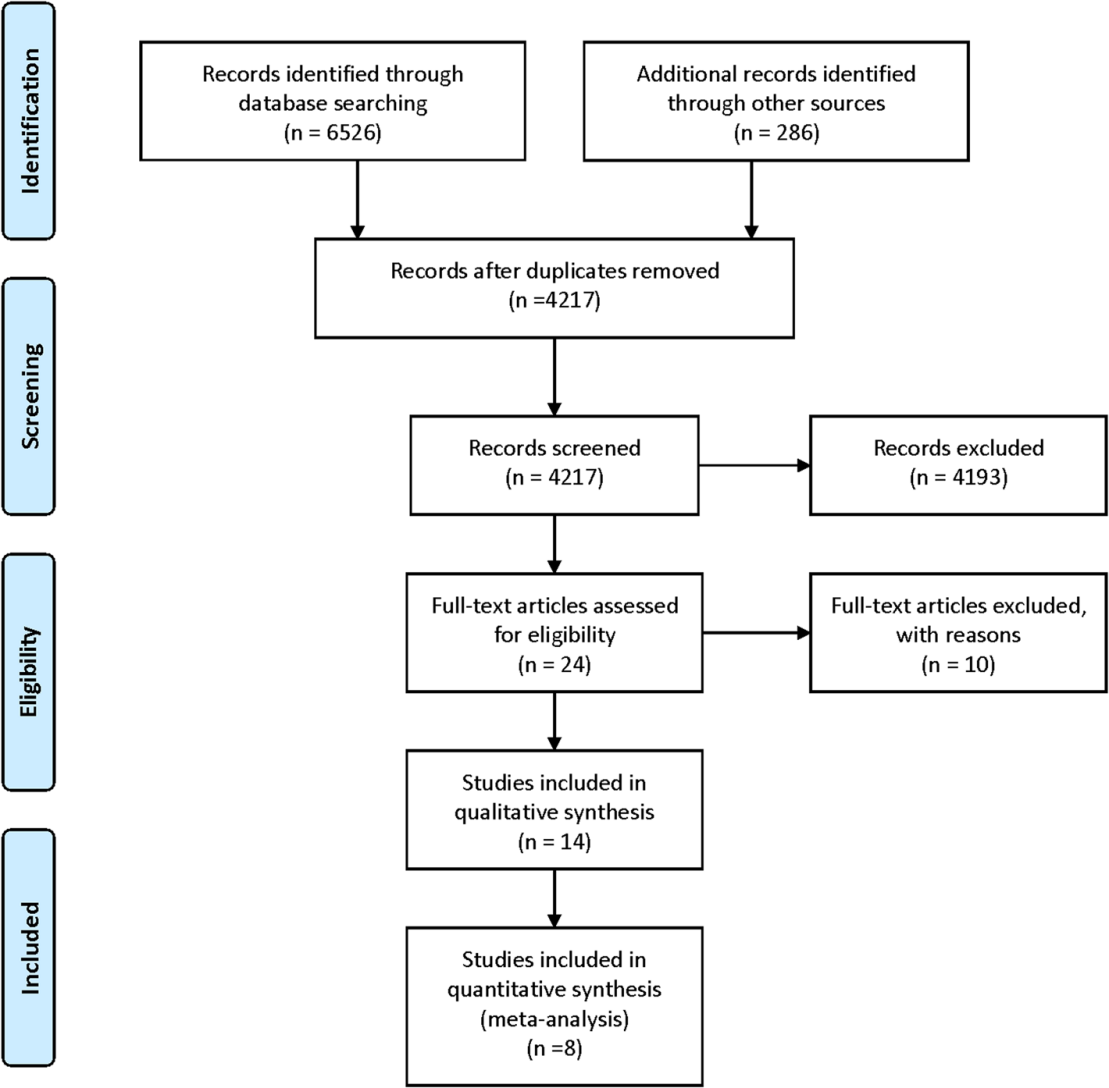
PRISMA flow diagram depicts our search results and selection of included studies in this systematic review

Our search identified 6812 records. After removing duplicate reports, we screened 4217 records for inclusion and excluded 4193 records (PRISMA flow diagram, Fig. 1). Of the 24 full text articles reviewed for eligibility, 14 studies met our inclusion criteria. Ten excluded studies and reasons for exclusion are summarized in Table 1. Methodological quality of the 14 included studies was assessed using the item checklist and responses as “yes”, “no,” or “unclear” are reported in Table 2. The microbiota characteristics reported by the 14 included studies including alpha and beta diversity indices and microbial profiles are summarized in Additional file 2. It should be noted that when evaluating diversity in a non-complex microbial population, shifts may be attributed to one or few bacterial taxa. As such, differences in diversity may serve as a proxy for overrepresentation or underrepresentation of small number of organisms and are discussed later in the paper.

**Table 1:** Table of excluded studies

Study	Reason for exclusion
Schwiertz 2003 [57]	A study of 29 preterm infants by PCR-DGGE analysis of which only one case of NEC was observed. No comparison of NEC and controls.
Bjorkstorm 2009 [58]	Only stool cultures and fecal calprotectin were measured.
LaTuga 2011 [59]	A study of eleven ELBW infants was excluded as there were no direct comparison of NEC and controls.
Morowitz 2011 [49]	Community genomic analysis at the strain level in one premature infant was excluded because the study did not compare NEC and controls.
Sharon 2013 [60]	Time shifts in community genomics was excluded because the study did not compare NEC and controls.
Carlisle 2013 [10]	A review was excluded.
Grishin 2013 [61]	A review was excluded.
Torraza 2013 [56]	A review was excluded.
Taft 2014 [55]	Description of the microbiome in preterm infants without NEC or sepsis and not a comparison of NEC with controls.
Raveh-Sadka 2015 [62]	Not a comparison of NEC with controls.

**Table 2:** Methodological assessment of included studies

Assessment criteria	Millar 1996 [63]	De la Cochetiere 2004 [39]	Wang 2009 [11]	Mshvildadze 2010 [30]	Mai 2011 [31]	Smith 2012 [64]	Stewart 2012 [40]	Norman 2013 [34]	Torraza 2013 [56]	Morrow 2013 [33]	Zhou 2015 [37]	McMurtry 2015 [32]	Sim 2015 [35]	Warner 2016 [38]
Do inclusion/exclusion criteria vary across comparison groups?	Yes^a^	No	No	No	No	No	No	No	No	No	No	No	No	No
Is the selection of the comparison group inappropriate?	No	No	No	No	No	No	No	No	No	No	No	No	No	No
Were valid and reliable measures (outcomes) applied consistently across all study participants?	Yes	Yes	Yes	No^b^	Yes	Yes	No^c^	Yes	Yes	Yes	Yes	Yes	Yes	Yes
Are any important primary outcomes missing from the results?	No	No	No	No	No	No	No	No	No	No	No	No	No	No
Are results believable given the study limitations? (overall quality of the study)	Yes	Yes	Yes	Yes	Yes	Yes	Yes	Yes	Yes	Yes	Yes	Yes	Yes	Yes
To assess confounding														
Any attempt to balance allocation between groups (e.g., stratification, matching)	No	Yes	Yes	Yes	Yes	No	No	Yes	Yes	Yes	Yes	Yes	Yes	Yes
Were important confounding variables (gestational age or birth weight) taken into account in the design and/or analysis (by matching, stratification, multivariate analysis, etc.)	No	Yes	Yes	Yes	Yes	No	No	Yes	Yes	Yes	Yes	Yes	Yes	Yes

Assessing methodological quality by the key components of study design is recommended over assigning quality scores in observational studies. The inclusion and exclusion criteria were similar in cases and controls in all included studies except Millar et al., where one intestinal sample along with fecal samples was included in the NEC group. Molecular methods of microbiome analysis were reported for all patients in all studies except Mshvildadze [30], Stewart 2012, where data for only a subset of patients were reported. All studies had appropriate comparison groups, reported all of our pre-specified outcomes and results “were believable given the study limitations.” Ten out of 14 included studies addressed the issue of confounding by matching or stratification to avoid known confounders

^a^One NEC sample was tissue sample and was obtained post-mortem

^b^DGGE was performed in 23 infants and pyrosequencing was performed in a subset of 12 infants (6 of whom had NEC or sepsis)

^c^Molecular assessment of stools by DGGE was assessed in only 27 infants out of 38 (16 infants with NEC or sepsis and 11 control infants)

### Results of the meta-analyses

Nine of 14 studies included in the systematic review provided sequence and clinical metadata for both the NEC cases and control patients. Data from one study [30] was excluded due to shallow sequencing depth, and hence, data from eight studies were incorporated in our quantitative synthesis (106 NEC cases, 278 controls, 2944 samples) [31–38] (Additional file 3). The results of the systematic review and meta-analysis are reported as recommened by PRISMA guidelines (Additional file 4).

### Microbiome profile differences between NEC and controls

**Fig. 2:**
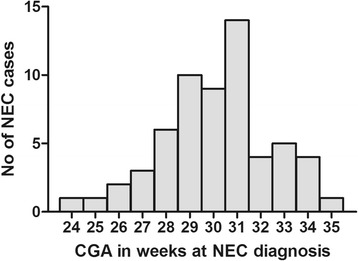
Histogram of necrotizing enterocolitis (NEC) cases by weeks corrected gestational age (CGA). The distribution of 61 cases of NEC plotted against CGA at the time of diagnosis is normal (Shapiro-Wilk test, *p =* 0.26). The mean ± SD, CGA at the time of NEC diagnosis was 30.1 ± 2.4 weeks.

**Fig. 3:**
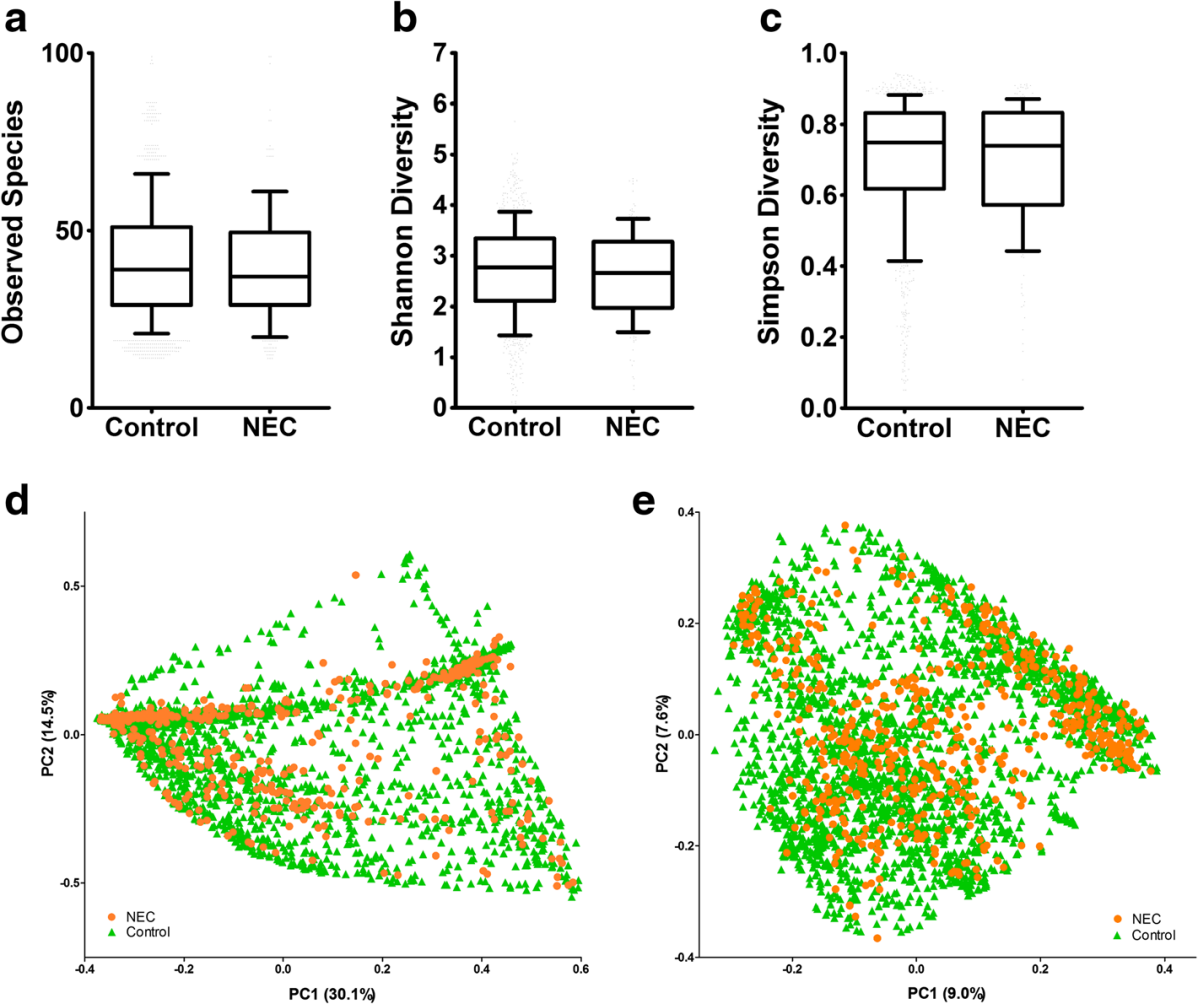
Alpha and beta diversity-NEC vs. controls. Alpha-diversity comparison for all corrected gestational ages (CGA) by NEC case vs. control by three metrics. **a** Observed species, **b** Shannon diversity, and **c** Simpson diversity, none of the comparisons are significantly different. Data is represented in *box* and *whisker plots* with median and *whiskers* representing 10–90th centiles. Principal co-ordinate (PCoA) plots of weighted UniFrac distance (**d**) and unweighted UniFrac distance (**e**) including all time points from all studies shows a lack of clustering between cases and controls. The *figure in parenthesis* next the axis labels represents the proportion of variation explained along each axis. *Orange circles* represent samples from preterm infants with NEC, and *green triangles* represent samples from control preterm infants

Corrected gestational age CGA at NEC diagnosis in preterm infants forms a normal distribution (*n* = 61, a subset of the population, Shapiro-Wilk test, *p =* 0.26) with a mean ± SD of 30.1 ± 2.4 weeks (median, 30 weeks) post-conception (Fig. 2). We analyzed data from samples collected at all CGAs and compared alpha-diversity metrics between infants with NEC and controls (Additional file 5). None of the alpha-diversity metrics calculated differed significantly between infants with NEC and controls (Fig. 3a–c). When alpha-diversity metrics (means of OTU richness and SDI) were plotted over CGA, we found that in control infants, OTU richness and SDI decreased from birth to their lowest level at about a CGA of 27 weeks and gradually increased to 36 weeks CGA (Additional file 6: Fig. S1a, b). The results of our negative binomial regression modeling support this pattern, indicating that the number of species increased significantly with gestational age (*p* < 0.001) and that NEC patients tended to have fewer species than controls after controlling for gestational age (*p =* 0.053). Principal coordinates analysis (PCoA) with the UniFrac metrics did not show clustering of samples as a function of NEC vs. control status (Fig. 3d, e).

**Fig. 4:**
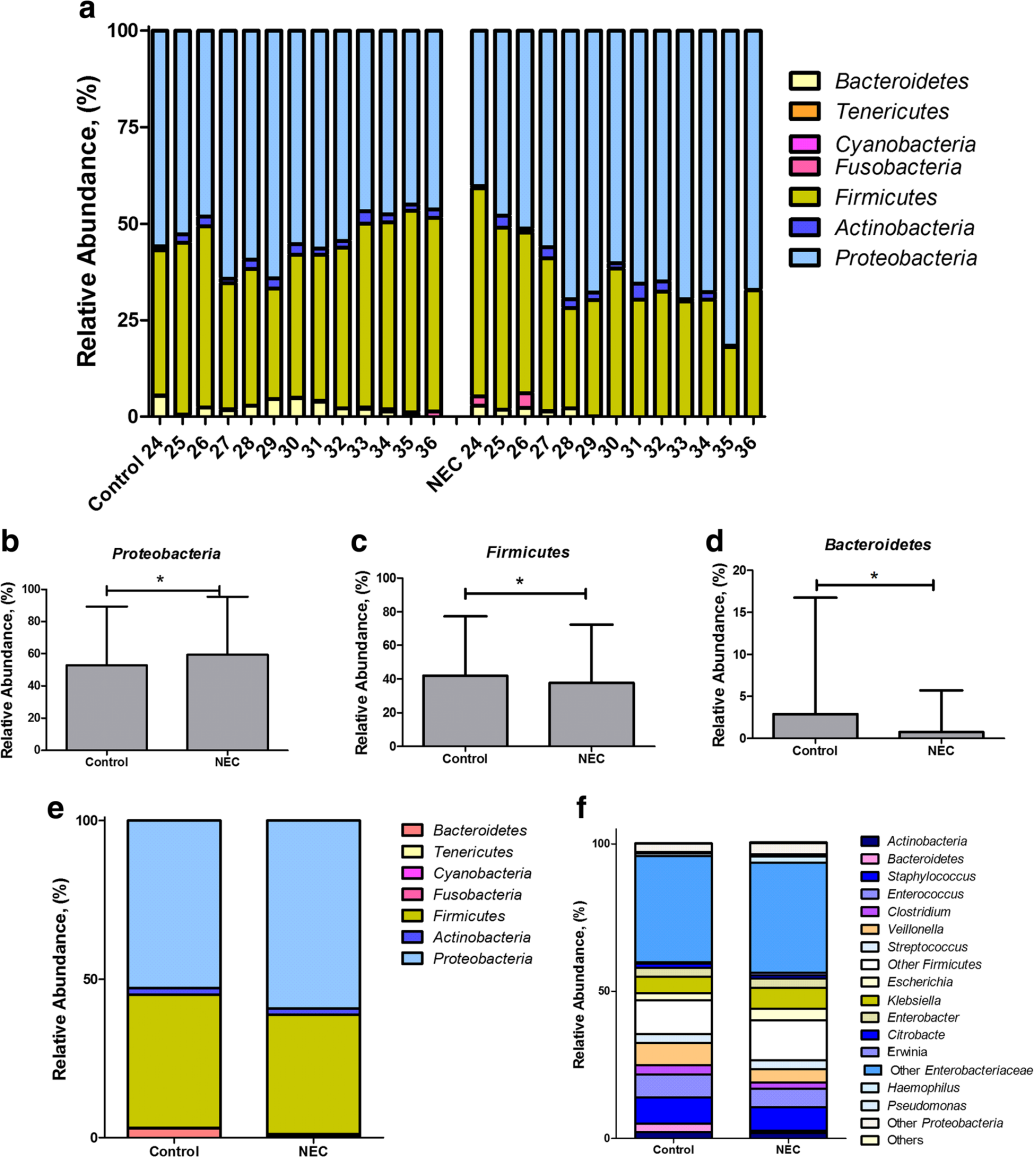
Comparison of taxonomic profiles between infants with necrotizing enterocolitis (NEC) and controls. **a** NEC infants had trends of increased relative abundance in *Proteobacteria* from 24 to 36 weeks corrected gestational age (CGA) accompanied by decreased abundances in *Firmicutes* and *Bacteroidetes*, relative to controls. In control infants, the relative abundance of *Proteobacteria* decreased after 27 weeks and coincided with an increase in *Firmicutes* and *Bacteroidetes*. **b**–**d** Phylum level differences between NEC cases and controls across CGA (data in means and SD) showed significant differences in *Proteobacteria*, *Firmicutes*, and *Bacteroidetes* (*p < 0.05). **e**, **f** Mean relative abundance distributions between NEC cases and controls at the phylum level (**e**) and genus level (**f**) when data from all CGAs are included

Taxonomic abundances grouped by CGA showed consistent trends toward increased relative abundances of *Proteobacteria* and decreased relative abundances of *Firmicutes* and *Bacteroidetes* in infants with NEC, which contrasted with decreased relative abundances of *Proteobacteria* and increased abundances of *Firmicutes* observed in control infants (Fig. 4). Of note, this deviation in taxonomic abundances in preterm infants with NEC vs. controls happens around 30 weeks CGA (Fig. 4a), which was the mean and median age of onset of NEC in preterm infants in this pooled cohort. When examined at all CGAs, significant differences were observed with respect to the relative abundances of *Proteobacteria*, *Firmicutes*, and *Bacteroidetes* (Fig. 4b–d). Differences in the relative abundance of bacterial phyla and genera in NEC infants vs. controls are depicted in Fig. 4e, f, respectively.

### Comparison of microbiota profiles between infants who had NEC compared to controls in relation to antibiotic exposure, diet, and mode of delivery

Investigating antibiotic exposure, which was defined inconsistently among the different studies (samples analyzed; controls with antibiotics *n =* 1822, controls without antibiotics, *n* = 107, NEC infants with antibiotics *n* = 273, and NEC infants without antibiotics, *n* = 29), OTU richness and SDI differed significantly between control infants who did not receive antibiotics compared to NEC infants who received antibiotics. OTU richness was significantly different between control infants with and without antibiotics, as was SDI between control infants with antibiotics and NEC infants with antibiotics (Additional file 7: Fig. S2a, b). PCoA by antibiotic exposure demonstrated clustering by the weighted and unweighted UniFrac metrics (Additional file 7: Fig. S2c, d). In conjunction with antibiotic exposure, we observed in control and NEC infants, increased relative abundances of *Proteobacteria*, and decreased relative abundances of *Firmicutes*, *Actinobacteria*, and *Bacteroidetes.* Among control infants, antibiotic exposure was associated with increased relative abundances of *Klebsiella*, unclassified *Enterobacteriaceae*, *Proteus*, *Paenibacillus*, *Epulopiscium*, and *Pseudomonas* at the genus level. Control infants not exposed to antibiotics had increased abundances of the genus *Clostridium* and unclassified *Clostridiaceae* (Additional file 7: Fig. S2e, f).

Investigating the role of diet (i.e., breast milk, formula, or both) (samples analyzed, breast milk-fed controls *n =* 967, formula-fed controls *n =* 55, both breast milk- and formula-fed controls *n =* 1300; breast milk-fed NEC *n =* 251, formula-fed NEC *n =* 15, both breast milk- and formula-fed NEC *n =* 241), significant differences in OTU richness but not SDI were observed between control breast milk-fed infants vs. NEC breast milk-fed infants and control subjects receiving breast milk vs. both breast milk and formula (Additional file 8: Fig. 3a, b). Meaningful clustering as a function of diet was not observed on PCoA plots (Additional file 8: Fig. S3c, d). Among control infants, we observed increased relative abundances of the phylum *Firmicutes* and decreased abundances of *Proteobacteria* in formula-fed infants compared to breast milk fed-infants (Additional file 8: Fig. S3e, f). Formula-fed babies who developed NEC had more *Proteobacteria* and less *Firmicutes* compared to breast milk-fed controls.

Investigating the role of mode of delivery (samples analyzed, vaginal delivery; control *n* = 683, NEC *n* = 178; C-section; control *n* = 1618, NEC *n* = 350), we found no differences in OTU richness or SDI betweeen NEC and controls but observed significant differences between control infants born by vaginal delivery compared to cesarean-section delivery (Additional file 9: Fig. S4a, b). No clustering was observed by mode of delivery on PCoA plots (Additional file 9: Fig. S4c, d). In control infants, we found increased relative abundance of the phylum *Firmicutes* in infants born by cesarean section and increased abundance of *Bacteroidetes* in infants born via vaginal delivery (Additional file 9: Fig. S4e, d).

### Heterogeneity among included studies

**Fig. 5:**
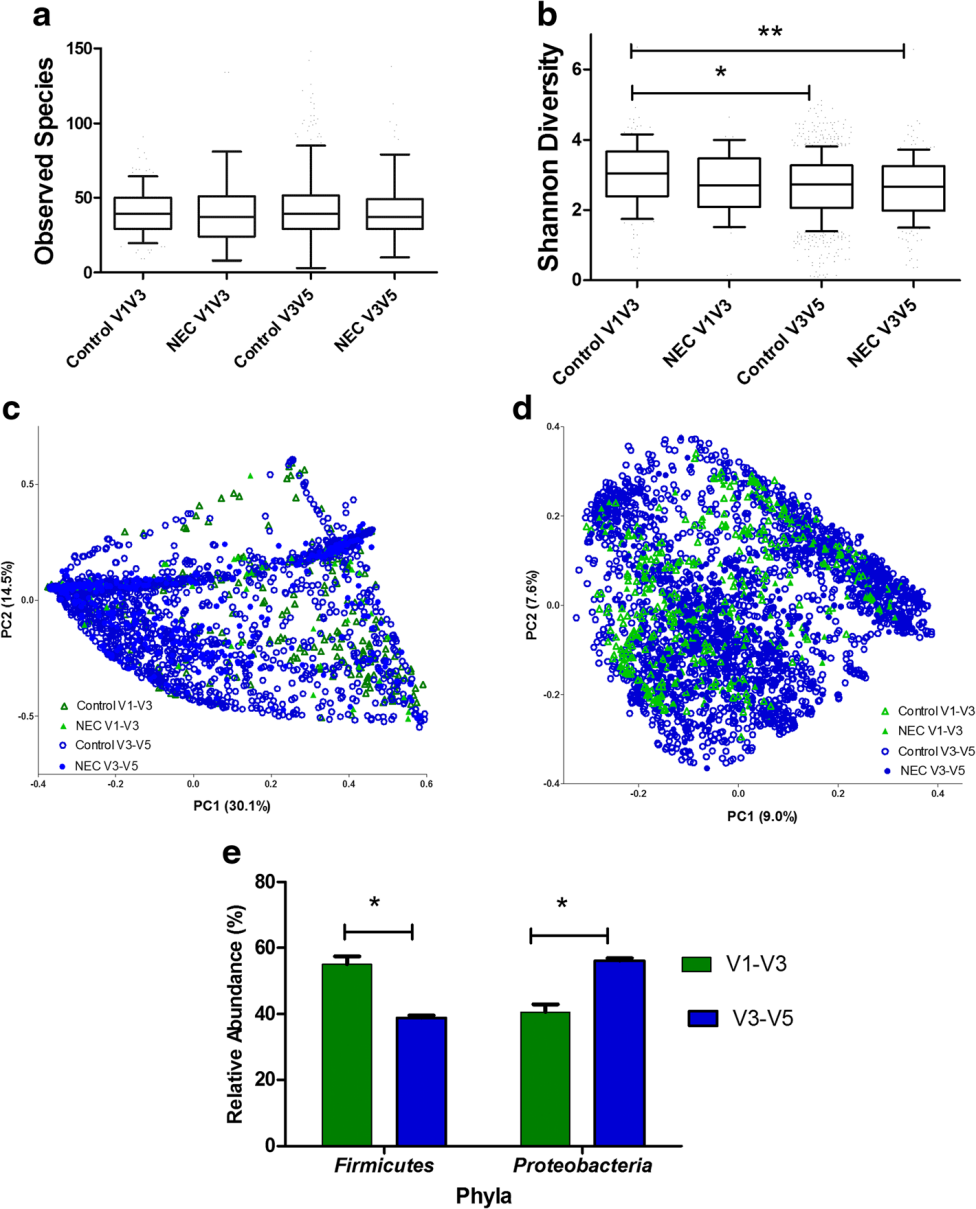
Heterogeneity assessment by 16S rRNA target region. Observed species (operational taxonomic unit, OTU) richness (**a**) and Shannon diversity index (SDI) values (**b**) in cases and controls are subgrouped by 16S rRNA target region (V1-V3 vs. V3-V5). Data is represented in *box* and *whisker plots* with median and *whiskers* representing 10–90th centiles. Significant differences were observed in SDI between controls of V1-V3 compared to controls of V3-V5 (*p < 0.05) and controls of V1-V3 compared to NEC V3-V5. No other significant differences were observed. **c**, **d** Depict weighted and unweighted UniFrac distances in PCoA plots of NEC and controls subgrouped 16S rRNA target region (V1-V3 vs V3-V5), and notable clustering was observed. The proportion of variation explained along each axis is listed in parenthesis with the axis labels. **e** Represents differences in proportion of sequences based on 16S rRNA target regions; V3-V5 targeting resulted in a significant increase in proportion of sequences of *Proteobacteria* and significant decrease in proportion of sequences of *Firmicutes* compared to studies targeting V1-V3.

Three studies in our meta-analysis targeted the V1-V3 region of the 16S rRNA gene, and five studies targeted the V3-V5 region. There were no differences in alpha-diversity metrics (OTU richness or SDI), nor was meaningful clustering observed within PCoA plots of UniFrac distances, as a function of variable region (Fig. 5a–d). In addition, studies targeting the V3-V5 region compared to the V1-V3 region reported increased relative abundances of *Proteobacteria* and decreased relative abundances of *Firmicutes* (Fig. 5e).

**Fig. 6:**
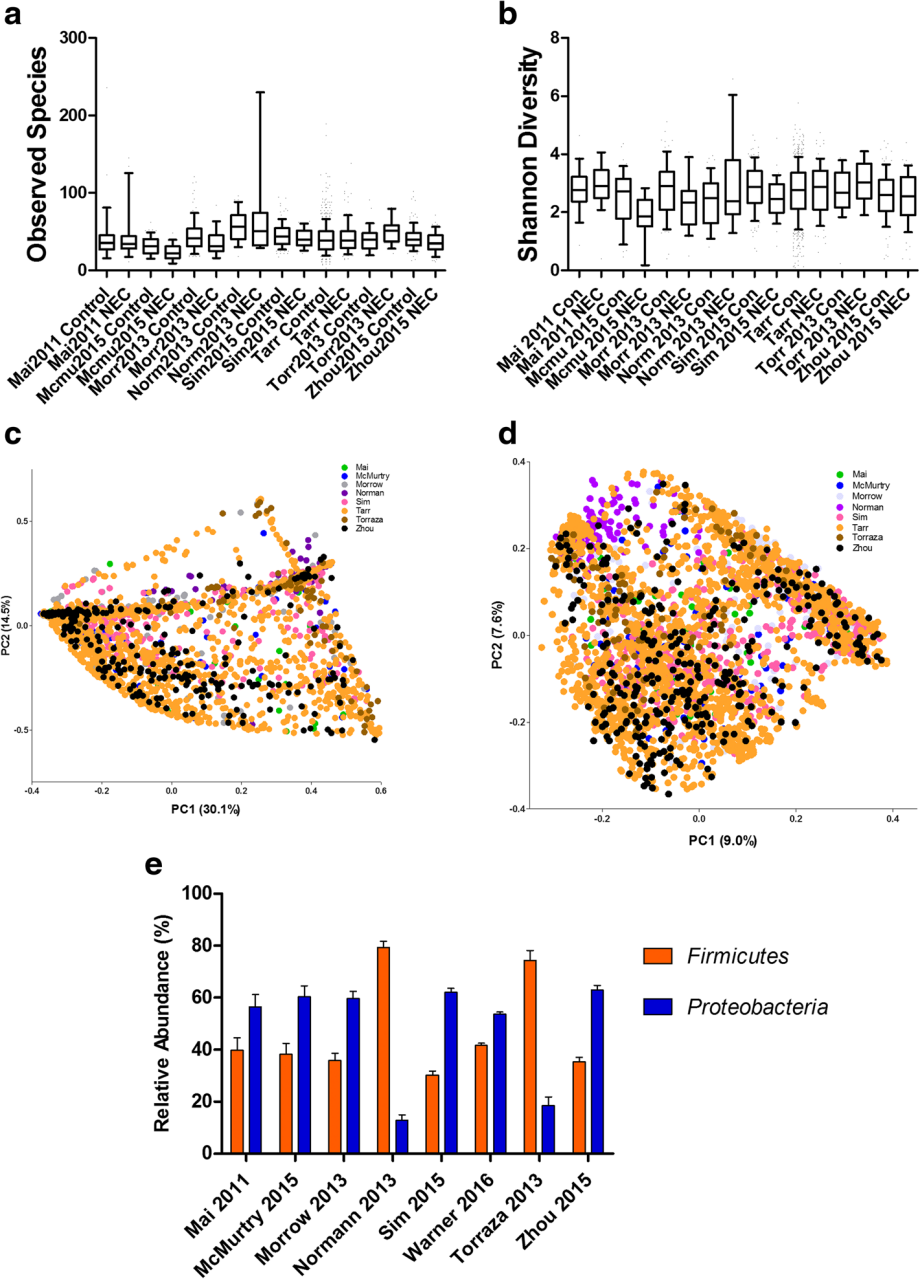
Heterogeneity assessment by study. Observed species (operational taxonomic unit, OTU) richness (**a**) and Shannon diversity index (SDI) values (**b**) in cases and controls are subgrouped by study. Data is represented in *box* and *whisker plots* with median and *whiskers* representing 10–90th centiles. **c**, **d** Depict weighted and unweighted UniFrac distances in PCoA plots of NEC and controls subgrouped by study, and no notable clustering was observed. The proportion of variation explained along each axis is listed in parenthesis with the axis labels. **e** Represents differences in proportion of sequences based on study. Studies by Normann [34] and Torraza [56] showed significant increase in proportion of sequences of *Firmicutes* and significant decrease in proportion of sequences of *Proteobacteria* compared to other studies (*p < 0.05)

We did not find significant differences in alpha diversity among studies although some variations were noted (Fig. 6a, b), and some clustering as a function of study was observed on PCoA plots of UniFrac distances (Fig. 6c, d). NEC samples from studies by Normann et al. (targeting V3-V5) and Torraza et al. (targeting V1-V3) had significantly increased relative abundance of the phylum *Firmicutes* and significantly decreased relative abundance of the phylum *Proteobacteria* compared to other studies (Fig. 6e).

## Discussion

Our review identified 14 eligible studies of which data from eight studies were synthesized by meta-analysis. We assessed the methodological quality and risk of bias in the 14 included studies by the key components of study design, which has been deemed to be more important than assigning quality scores in observational studies [24]. The methodological quality of the included observational studies was adequate [26]. All studies had appropriate comparison groups, reported all our pre-specified outcomes, and results were plausible given the study limitations. Few studies evaluated outcomes in a subset of participants. Eleven out of 14 included studies addressed the issue of confounding by matching or stratification to avoid known confounders.

Alpha-diversity metrics reported by the included studies were inconsistent. Three studies reported a decrease in alpha diversity in preterm infants with NEC compared to controls [11, 32, 37], one study reported decreasing trends [33] and others did not report differences [31, 34, 36]. One study [38] included mixed models predicting Shannon diversity and controlling for gestational age at birth, route of delivery, and birth weight. Significant time-associated trends were identified among control subjects, which increased with respect to diversity over time, while NEC cases did not. Significant differences in beta-diversity metrics between NEC cases, and controls were reported by six studies, two of which were fingerprinting-based [39, 40] and four were based on sequencing small pools of PCR amplification products [11, 31, 33, 36].

Reported microbial profiles preceding NEC in preterm infants were variable across the 14 included studies. Eight of the 14 studies report an increase in the relative abundance of the phylum *Proteobacteria* (class *Gammaproteobacteria* or family *Enterobacteriaceae*) in the stools of infants who developed NEC [11, 30, 31, 33–37]. Various studies reported an increased relative abundance of *Gammaproteobacteria* and a decrease in other bacterial species [11], increased relative abundances of *Enterococcus spp*. and *Citrobacter-*like sequences [30], increased relative abundances of *Proteobacteria*, or a decreased relative abundance of *Firmicutes* in stools before NEC diagnosis [31]. Increased relative abundances of *Proteobacteria* two weeks before and Actinobacteria 1 week before NEC diagnosis as well as lower counts of *Bifidobacteria* and *Bacteriodetes* have also been observed [36]. Morrow et al. reported two types of intestinal dysbiosis associated with NEC [33]; one was dominated by *Firmicutes* and the other by *Proteobacteria*, specifically *Enterobacteriaceae*. All NEC cases in the fore-mentioned study lacked *Propionibacterium*. Some studies reported the abundance of *Clostridium perfringens* [35, 37, 39] in the stools of neonates with NEC, but one study reported the absence of Clostridia in association with NEC [32]. Three studies reported unique OTUs in preterm infants with NEC: a unique bacterial OTU belonging to the *Enterobacteriaceae* family [31] and in the first stool samples, the presence of a novel sequence closest to *Klebsiella pneumoniae* [36]. In our pooled meta-analysis, we found that decreased abundances of *Firmicutes* and *Bacteroidetes* and increased abundances of *Proteobacteria* precede the diagnosis of NEC in preterm infants.

We synthesized high-quality data available from eight studies in a sequence-based meta-analysis. The mean CGA at NEC diagnosis (*n* = 61) was 30.1 ± 2.4 weeks (median of 30 weeks). The Canadian network study suggested a bimodal distribution for the onset of NEC in preterm infants with an early onset and a late onset NEC [41]. NEC occurred later after birth in infants born at younger gestational ages and earlier after birth in infants born at later gestational ages. Other investigators report a distribution for the onset of NEC in preterm infants similar to our findings [38, 42, 43]. We observed patterns of increased OTU richness over time where the number of species increased significantly with gestational age. This may represent the transition beyond the perinatally acquired microbiome which may be influenced by mode of delivery, the NICU environment, feeding, antibiotic exposure, or other factors and warrants further study.

A patterned progression of the intestinal microbiome has been observed in preterm infants [44]. In a study of 922 16S rRNA sequence libraries from 58 infants, a patterned progression of bacterial classes from *Bacilli* to *Gammaproteobacteria* and then to *Clostridia* was described, and by CGA 33–36 weeks, the communities were well populated by anaerobes [44]. We were unable to demonstrate this patterned progression in our meta-analysis due to lack of associated meta-data in some studies. In our meta-analyses, we found gradual shifts in the relative abundances of multiple phyla at about 27 weeks CGA, where decreased abundances of *Firmicutes* and *Bacteroidetes* and increased abundances of *Proteobacteria* precede the diagnosis of NEC in preterm infants. *Proteobacteria* are recognized by the innate immune system by TLR4 in the intestine, which may play a significant role in intestinal inflammation, enterocyte injury that may lead to the development of NEC in preterm infants [18]. TLR4 expression is increased in the intestinal tract of preterm neonates and may regulate the balance between intestinal repair and injury [12]. It is interesting to note that TLR4 mutant mice and TLR4 knock out mice are protected from NEC [17, 45–47], and inhibition of the TLR4 pathway may provide novel strategies in the treatment or prevention of NEC.

Our sequence-based analysis portrayed inconsistent differences in SDI, OTU richness, and Simpson diversity index between NEC cases and controls. Comparison of species richness over time found that when controlling for corrected gestational, NEC patients tended to have fewer species than controls (*p =* 0.053, negative binomial regression model). The absence of consistent differences with respect to alpha diversity may be due to the low starting point of microbial diversity that is observed in preterm infants, relatively small sample sizes in each of the included studies, or variability with respect to methodology for generating microbiome data. Of particular concern are the limited numbers of taxa present in preterm stool. This limitation places constraints on interpreting diversity changes as diversity in a non-complex population could reflect changes in only one taxon.

We did not observe distinct clustering of NEC and control samples by unweighted or weighted UniFrac metrics. The lack of consistency in distinct clustering of cases and controls may be due to the methodological, clinical, or study heterogeneity of the included studies. We found a consistent trend toward increased relative abundances of *Proteobacteria* and decreased in the relative abundances of *Firmicutes* and *Bacteroidetes* preceding NEC. This is consistent with the predominance of *Proteobacteria* in NEC infants reported in some studies [11, 31, 33, 36]. Although the pooling of data across studies increased sample size to give statistical power to detect differences, this benefit may have been outweighed by variation with respect to sampling and lab-based methodology (e.g., sample handling, storage, or DNA extraction method) associated with the different studies. Although no single genus or bacterium appears to cause or precede NEC, increased relative abundances of *Proteobacteria* appear to be consistent with NEC-associated dysbiosis. A recent study that studied fecal microbiome in preterm and term infants by shotgun metagenomic sequencing has reported association with uropathogenic *Escherichia coli* colonization and necrotizing enterocolitis [48], though this study was unusual in the predominance of *E. coli* among NEC cases. The clinical significance of the differences in phyla and genera between cases and controls of the pooled data clearly needs further scrutiny. If dysbiosis with increased *Proteobacteria* and decreased *Firmicutes* and *Bacteroidetes* is associated with NEC in preterm infants, then measures to balance these taxa by probiotic therapy or luminal antibiotics such as aminoglycosides may be beneficial.

We report our bacterial data at the taxonomic depth of phylum. However, we cannot state with certainty if phylum is the appropriate level to conduct these analyses. If the inciting mechanism is a shared phenotype across all genera within the class, then attempts to further refine an etiology (or protective group of bacteria) to a specific genus, species, clade, or genotype would obscure associations. Alternatively, identification of important strain level differences have been reported, which analyses focusing on higher taxonomic levels would overlook [33, 49]. Indeed, Warner et al. have been able to identify risk-associated populations at the class level (*Gammaproteobacteria*) and protection-associated populations at the genus level (the members of the putatively protective *Negativicutes* class were overwhelmingly *Veillonella*)[38]. We look forward to continuing analysis of these data sets and, in additional cohorts, in future attempts to confirm or refute the associations we identify and to identify injurious or protective microbes more narrowly, if possible.

We examined clinical determinants that are known to affect the developing intestinal microbiome, namely, antibiotic exposure preceding NEC, mode of delivery, and diet. We observed differences in the microbial profiles in the preterm infants who had antibiotics (any antibiotic) prior to the onset of NEC and found increased relative abundances of *Proteobacteria* and decreased relative abundances of *Firmicutes* and *Actinobacteria*. This may coincide with the proteobacterial bloom preceding NEC [11, 31, 33, 36]. Our observations support the results of studies that report association of prior antibiotic use with increased incidence of NEC [14, 15, 50], though we were not able to take into account various classes of antibiotics, which have been reported to be determinants of intra-gut bacterial community structure [51]. We also sought taxonomic differences by mode of delivery (cesarean section or vaginal delivery) and found no differences in NEC infants but observed increased relative abundances of *Firmicutes* after cesarean section and increased relative abundances of *Bacteroides* after vaginal delivery in control infants. We also assessed taxonomic profiles of fecal microbiota based on type of feeding (breast milk, formula, or both). We found no differences in NEC infants but observed an increased relative abundance of *Firmicutes* in infant-fed formula compared to breast milk and an increased relative abundance of *Proteobacteria* in those fed breast milk compared to formula (controls). We identified no significant associations of mode of delivery or type of feeding with the microbial profiles of preterm infants who developed NEC.

We also investigated for potential sources of heterogeneity in the studies with respect to the variable region of the 16S rRNA gene targeted (V1-V3 vs. V3-V5) and by study and did observe differences in alpha-diversity metrics and UniFrac distances with respect to study and 16S rRNA target regions. *Proteobacteria* were more abundant and *Firmicutes* less abundant in studies targeting V3-V5 compared to V1-V3. NEC samples from studies by Normann et al. (targeting V3-V5) and Torraza et al. (targeting V1-V3) had significantly greater relative abundances of the phylum *Firmicutes* and significantly decreased relative abundances of the phylum *Proteobacteria* compared to other studies. It is widely recognized that the use of partial 16S rRNA gene sequences instead of the whole gene, or whole genome, can give rise to inconsistencies in evaluating microbiota composition. Primer bias may influence which members of the microbial community are amplified and detected, and OTU counts arising from different 16S rRNA gene regions can be inconsistent, challenging interpretation and, to some degree, limiting consensus with respect to which region best reflects the gut microbial community. Indeed, investigators have reported somewhat differing composition and varying phylogenetic divergence [52, 53]when multiple hypervariable regions were sequenced on the same samples simultaneously, leading to differential detection of members of *Prevotella*, *Fusobacterium*, *Eubacterium*, *Enterococcus*, *Streptococcus*, *Granulicatella*, *Bacteroides*, *Porphyromonas*, and *Treponema* [54, 55].

These studies were performed in different locations and hospitals, and recent studies have highlighted the role of geographical differences in the intestinal microbiota of preterm infants [55]. We were unable to explore heterogeneity resulting from differences in DNA extraction protocols and sample processing methods due to considerable variation in included studies. The methodological variations were too great to provide statistical power for reasonable conclusions.

### Strengths and limitations of the review

We adhered to the standard methods of conducting a systematic review as per recommended guidelines [23, 24]. We searched comprehensively for all eligible studies using clinically relevant inclusion criteria and strove to explain sources of heterogeneity by careful analysis of the study methods and reporting subgroup analyses. Unlike meta-analyses of randomized control trials, heterogeneity is a well-recognized problem in reviews of observational studies [24]. Publication bias in studies reporting negative results may have excluded eligible unpublished studies. Poor reporting of study design, method of enrollment, and patient characteristics may hamper methodological assessment and external validity of the studies. The inconsistency in the microbiome profiles in the stools preceding NEC may be due to considerable clinical and methodological heterogeneity among the studies. In addition, while metadata was derived from both NCBI dbGaP as well as from investigators individually, not all metadata could be standardized and/or be available for incorporation into this analysis. The other reasons for inconsistency of results among studies may be differences in location emphasizing the importance of the unique NICU environment and associated infection control practices [55] and differences in time periods of study. We were not able to determine the time to event (NEC) analysis due to unavailability of day of NEC diagnosis from the meta-data. The time to event analysis may have enabled us to describe pre-NEC changes in microbiome more accurately.

Although there are many narrative reviews, to our knowledge, this is the first systematic review and meta-analysis of microbial dysbiosis preceding NEC. Our review supports the hypothesis that intestinal dysbiosis with an increase in microbes found primarily within the phylum *Proteobacteria* and a decrease in *Firmicutes* and *Bacteroidetes* precedes NEC in preterm infants. Furthermore, antibiotic usage is associated with increased abundance of *Proteobacteria*, which is also associated with higher incidence of NEC. In contrast, increased *Proteobacteria* were associated with intake of breast milk but type of feeding did not associate with development of NEC. The relationship between *Proteobacteria* and NEC seen in our review supports a strong association but should be viewed in the context of considerable variations in clinical and methodological characteristics of included studies. This certainly highlights the need for standardization of methods and transparent reporting of microbiome studies to advance this field. Our data with its limitations supports the microbial dysbiosis theory for the development of NEC but also highlights the lack of host response data to complete the story. Studies supporting a biologic plausibility will need to include metagenomics, transcriptomics, and additional data on inflammatory mediators. Proof of causality will need to further fulfill Koch’s postulates by recapitulating certain aspects of the disease in model systems. 16S rRNA gene sequencing often limits resolution to the level of the genus, whereas metagenomic data from whole genome shotgun sequencing may provide resolution at the strain level. Metagenomic analysis and metabolomics may reveal the differences in the functionality of the microbiome in patients who develop NEC. Optimizing the microbiome and correcting the microbial profile perturbations by microbial biotherapy may prevent NEC and improve clinical outcomes.

## Additional files

Additional file 1: Search strategy for the systematic review in word document. (DOCX 14 kb)
Additional file 2: Characteristics of included studies and results reported. This excel document describes the details the 14 included studies and results of alpha-, beta-diversity metrics and microbial profiles. (XLSX 16 kb)
Additional file 3: Characteristics of studies included in the meta-analysis. This excel document describes the details of the data from 8 studies that were included in the meta-analysis. (XLSX 12 kb)
Additional file 4: PRISMA checklist. This word document profiles the details of the systematic review that are recommended to be reported by the PRISMA statement. (DOC 63 kb)
Additional file 5: Zip file containing sequence data shared by authors for Mshvildadze [30], Mai [31], Normann [34], and Torraza [56]. (ZIP 83304 kb)
Additional file 6: Alpha diversity trends over corrected gestational age alpha-diversity metrics (mean with SD) by corrected gestational age (weeks). Fig. A depicts observed species (in operational taxonomic units, OTUs) and Fig. B depicts Shannon diversity index (SDI) against corrected gestational age (CGA) in controls and in infants with necrotizing enterocolitis (NEC). Data is represented in box and whisker plots with median and whiskers representing 10–90th centiles. The results of a fitted negative binomial regression model showed that the number of species increased significantly with gestational age (*p* < 0.001), and NEC patients tended to have fewer species than controls after controlling for gestational age (*p =* 0.053). (TIF 39 kb)
Additional file 7: Comparison of microbiome profiles in NEC and control infants subgrouped by antibiotic usage before the onset of NEC. Observed species (operational taxonomic unit, OTU) richness (**a**) and Shannon diversity index (SDI) values (**b**) in cases and controls are subgrouped by antibiotic usage. Data is represented in box and whisker plots with median and whiskers representing 10–90th centiles. Significant differences were observed in OTU richness between controls who were exposed to antibiotics compared to controls who were not (**p* < 0.05). No other significant differences were observed. Figs. c and d depict weighted and unweighted UniFrac distances in PCoA plots of NEC and controls subgrouped by antibiotic usage, and no notable clustering was observed. The proportion of variation explained along each axis is listed in parenthesis with the axis labels. Figs. e and f represent mean relative abundances of bacterial taxa in NEC cases and controls stratified by antibiotic usage at the phylum and genus levels, respectively. (TIF 712 kb)
Additional file 8: Comparison of microbiota profiles in NEC and control infants subgrouped by diet. Observed species (operational taxonomic unit, OTU) richness (**A**) and Shannon diversity index (SDI) values (**B**) in cases and controls are subgrouped by diet (breast milk, formula, or both breastmilk and formula). Data is represented in box and whisker plots with median and whiskers representing 10–90th centiles. We observed significant differences in OTUs between these groups; breast milk-fed controls vs. breast milk-fed NEC infants (**p* < 0.05) and both milk-fed controls vs. breast milk-fed controls (***p* < 0.05). No other significant differences were observed. Figs. c and d depict weighted and unweighted PCoA plots of UniFrac distances of NEC cases and controls stratified by diet, and no notable clustering was observed. The proportion of variation explained along each axis is listed in parenthesis with the axis labels. Figs. e and f represent mean relative abundance distributions by NEC cases and controls subgrouped by diet at the phylum and genus levels, respectively. (TIF 931 kb)
Additional file 9: Comparison of microbiota profiles in NEC and control infants subgrouped by mode of delivery. Observed species (operational taxonomic unit, OTU) richness (**a**) and Shannon diversity index (SDI) values (**b**) in cases and controls are subgrouped by mode of delivery: cesarean section (C-sec) or vaginal (Vag) delivery. Data is represented in box and whisker plots with median and whiskers representing 10–90th centiles. We observed significant differences in OTUs and SDI between controls delivered by C-section vs. vaginal delivery (**p* < 0.05) and controls delivered by C-section vs. NEC infants delivered by vaginal delivery (***p* <0.05). No other significant differences were observed. Figs. c and d depict weighted and unweighted PCoA plots of UniFrac distances of NEC cases and controls (all CGAs) stratified by mode of delivery, respectively, and no notable clustering was observed. The proportion of variation explained along each axis is listed in parenthesis with the axis labels. Figs. e and f represent mean relative abundance distributions by NEC cases and controls subgrouped by mode of delivery at the phylum and genus levels, respectively. (TIF 1002 kb)
