# Supplementary material for: Intestinal dysbiosis in preterm infants preceding necrotizing enterocolitis: a systematic review and meta-analysis
Source: Microbiome. 2017 Mar 9;5:31. doi: 10.1186/s40168-017-0248-8 (PMC5343300; doi:10.1186/s40168-017-0248-8)
Supplement: Additional file 1: — Search strategy for the systematic review in word document. (DOCX 14 kb) [file 40168_2017_248_MOESM1_ESM.docx]

**Additional file 1: Search Strategy** **and databases searched**

We searched for eligible studies using the Cochrane Neonatal Review Group's (CNRG) search strategy (**supplementary file 1**) without language or publication date restriction in April 2016 (<http://neonatal.cochrane.org/>). The following electronic databases were searched: MEDLINE (<http://ovidsp.ovid.com>, 1966 to April 2016), PubMed (<http://www.ncbi.nlm.nih.gov/pubmed>), Cumulative Index to Nursing and Allied Health Literature (CINAHL, <http://www.ebscohost.com/biomedical-libraries/the-cinahl-database>, 1982 to April 2016). Conference abstracts were identified from the proceedings of Pediatric Academic Societies (<http://www.abstracts2view.com/pasall>, 2002 to April 2016). We also searched reference lists of relevant identified articles and contacted authors in the field for any eligible studies.

**Search methods for identification of studies**

Our search for relevant articles using the Cochrane Neonatal Review Group's search strategy without language restriction was performed in April 2016. The following sources were searched:

1. The Cochrane Central Register of Controlled Trials (CENTRAL, *The Cochrane Library*);
2. Electronic journal reference databases: MEDLINE (1966 to present) and PREMEDLINE, EMBASE (1980 to May 2015), CINAHL (1982 to April 2015), Web of Science (1975 to April 2015) and the Oxford Database of Perinatal Trials;
3. Ongoing trials at the following web sites: <http://www.clinicaltrials.gov/>, <http://www.controlled-trials.com/> and the WHO International Clinical Trials Platform (ICTRP) at [www.who.int/ctrp/en](http://www.who.int/ctrp/en).
4. Abstracts of conferences - proceedings of Pediatric Academic Societies (American Pediatric Society, Society for Pediatric Research and European Society for Pediatric Research) (PAS electronic version from 2000 to April 2015 and ESPR hand search from 2000 to April 2015);
5. Reference lists of identified clinical trials and in the authors' personal files.

In addition, we contacted authors who have published in the field of neonatal microbiome to identify relevant articles.

**Search strategy** for MEDLINE and PREMEDLINE. This strategy was adapted to suit EMBASE, CINAHL and CENTRAL.

1. explode 'microbiome' [all subheadings in MIME, MJME]
2. ‘gastrointestinal microbiome’ as a key word
3. ‘Microbiota’
4. ‘Necrotizing enterocolitis’
5. ‘NEC’
6. ‘enterocolitis’
7. # 1 OR # 2 OR # 3 OR # 4 OR # 5
8. explode 'infant - newborn' [all subheadings in MIME, MJME]
9. Neonat*
10. Newborn*
11. # 7 OR # 8 OR # 9
12. # 6 AND # 10
