# Supplementary material for: Intestinal dysbiosis in preterm infants preceding necrotizing enterocolitis: a systematic review and meta-analysis
Source: Microbiome. 2017 Mar 9;5:31. doi: 10.1186/s40168-017-0248-8 (PMC5343300; doi:10.1186/s40168-017-0248-8)
Supplement: Additional file 5: — Zip file containing sequence data shared by authors for Mshvildadze [30], Mai [31], Normann [34], and Torraza [56]. (ZIP 83304 kb) [file 40168_2017_248_MOESM5_ESM.zip › NECfirstfecsampl.docx]

NEC cases (n=13)

RUN1 1.TCA.454Reads.fna ACGAG GACTACHVGGGTATCTAATCC **29A** 16 FNV1

RUN2 1.TCA.454Reads.fna TACTGCA GACTACHVGGGTATCTAATCC **60A** 111 FNC1

RUN1 1.TCA.454Reads.fna TGAGT GACTACHVGGGTATCTAATCC **66A** 46 FNC1

RUN2 1.TCA.454Reads.fna TAGACTC GACTACHVGGGTATCTAATCC **73A** 116 FCC1

RUN2 1.TCA.454Reads.fna TAGCACT GACTACHVGGGTATCTAATCC **98A** 121 FNV1

RUN2 1.TCA.454Reads.fna TCATGAC GACTACHVGGGTATCTAATCC **110A** 133 FNC1

RUN2 1.TCA.454Reads.fna TGTACGA GACTACHVGGGTATCTAATCC **130A** 151 FNV1

RUN2 1.TCA.454Reads.fna TGTCACA GACTACHVGGGTATCTAATCC **141A** 153 FCV1

RUN2 1.TCA.454Reads.fna TGAGTAC GACTACHVGGGTATCTAATCC **158A** 160 FNV1

RUN2 1.TCA.454Reads.fna ATACTGC GACTACHVGGGTATCTAATCC **167A** 169 FNV1

RUN2 1.TCA.454Reads.fna ATCGTGT GACTACHVGGGTATCTAATCC **176A** 178 FNV1

RUN2 1.TCA.454Reads.fna ACTAGCT GACTACHVGGGTATCTAATCC **198A** 192 FNC1

RUN2 1.TCA.454Reads.fna ACTGACT GACTACHVGGGTATCTAATCC **206A** 200 FNC1

**Bold =** Patient and sampling date ID information
