# Supplementary material for: Intestinal dysbiosis in preterm infants preceding necrotizing enterocolitis: a systematic review and meta-analysis
Source: Microbiome. 2017 Mar 9;5:31. doi: 10.1186/s40168-017-0248-8 (PMC5343300; doi:10.1186/s40168-017-0248-8)
Supplement: Additional file 5: — Zip file containing sequence data shared by authors for Mshvildadze [30], Mai [31], Normann [34], and Torraza [56]. (ZIP 83304 kb) [file 40168_2017_248_MOESM5_ESM.zip › NEC2_11222013.fna.htm]

>n.538.6.bb_1
GTAGTTAGCCGGTGCTTCTTCTGCAGGTACCGTACACCACAAGCTTCGCCCCGTGCTGAAAGCGGTTTACAACCCGAAGGCCGTCATCCCGCACGCGGCGCTGCTGCATCAGGCTTCCGCCCATTGTGCAATATTCCCCACTGCTGCCTCCCGTAGGAGTCTGGACCGTGTCTCAGTTCCAGTGTGGCTGGTCATCCTCTCAGACCAGCTAGGGGATCGTCGCCTAGGTGAGCCGTTACCCACCTACTAGCTAATCCCATCTGGGCACATCTGATGGCAAGAGGCCCGAAGGT
>n.538.6.bb_2
GTAGTTAGCCGGTGCTTCTTCTGCAGGTACCGTCACCACAAGCTTCGCCCCTGCTGAAAGCGGTTTACAACCCGAAGGCCGTCATCCCGCACGCGGCGTTGCTGCATCAGGCTTCCGCCCATTGTGCAATATTCCCCACTGCTGCCTCCCGTAGGAGTCTGGGCCGTATCTCAGTCCCAATGTGGCCGGTCGCCCTCTCAGGCCGGCTACCCGTCAAAGGCCTTGGTAAGCCACTACCCCACCAACAAGCTGATAAGCCGCGAGTCCATCCAAAACGCCGAAGCTTTCCAACCCCACCATGCAGCAAGGATTCCTATCCGGTATTAGCCCCAGTTTCCTGAAGTT
>n.538.6.bb_3
TAGTTAGCCGTGGCTTTCTGATTAGGTACCGTCAAGACGTGCATAGTTACTTACACATTTGTTCTTCCCTAATAACAGAGTTTTACGATCCGAAGACCTTCATCACTCACGCGGCGTTGCTCCGTCAGGCTTTCGCCCATTGCGGAAGATTCCCTACTGCTGCCTCCCGTAGGAGTCTGGACCGTGTCTCAGTTCCAGTGTGGCCGATCACCCTCTCAGGGTCGGCTACGCATCGTTGCCTTGGTAAGCCGTTACCTTACCAACTAGCTAATGCGGCGCGGATCCATCTATAAGTGACAGC
>n.538.6.bb_4
GTAGTTAGCCGGTGCTTCTTCTGCAGGTACCGTCACCACAAGCTTCGCCCCTGCTGAAAGCGGTTTACAACCCGAAGGCCGTCATCCCGCACGCGGCGTTGCTGCATCAGGCTTCCGCCCATTGTGCAATATTCCCCACTGCTGCCTCCCGTAGGAGTCTGGGCCGTATCTCAGTCCCAATGTGGCCGGTCGCCCTCTCAGGCCGGCTACCCGTCAAAGCCTTGGTAAGCCACTACCCCACCAACAAGCTGATAAGCCGCGAGTCCATCCAAAAACCGCCGAAGCTTTCCAACCCCACCATGCAGCAAGGATTCCTATCCGGTATTAGCCCCAGTTTCCTGAAGTTATCCCGAAG
>n.538.6.bb_5
GTAGTTAGCCGGTGCTTCTTCTGCAGGTACCGTCACCACAAGCTTCGCCCCTGCTGAAAGCGGTTTACAACCCGAAGGCCGTCATCCCGCACGCGGCGTTGCTGCATCAGGCTTCCGCCCATTGTGCAATATTCCCCACTGCTGCCTCCCGTAGGAGTCTGGGCCGTATCTCAGTCCCAATGTGGCCGGTCGCCCTCTCAGGCCGGCTACCCGGTCAAAGCCTTGGTAAGCCACTACCCCACCAACAAGCTGATAAGCCGCGAGTCCATCCAAAACCGCCGAAGCTTTCCAACCCCACCATGCAGCAAGGATTCCTATCCGGTATTAGCCCCAGTTTCC
>n.538.6.bb_6
GGAGTTAGCCGGTGCTTCTTCTGCGGGTAACGTCAATTGCTGAGGTTATTAACCTCAACACCTTCCTCCCCGCTGAAAGTACTTTACAACCCGAAGGCCTTCTTCATACACGCGGCATGGCTGCATCAGGCTTGCGCCCATTGTGCAATATTCCCCACTGCTGCCTCCCGTAGGAGTCTGGACCGTGTCTCAGTTCCAGTGTGGCTGGGTCATCCTCTCAGACCAGCTAGGGATCGTCGCCTAGGTGAGCCGTTACCCCACCTACTAGCTAATCCCATCTGGGCACATCTGATGGCAAGAGGCCCGAAGGTCCCCTCTTTGGTCTTGCGACGTTATGCGGTATTAGCTACCGTTTCCAG
>n.538.6.bb_7
GTAGTTAGCCGGTGCTTCTTCTGCAGGTACCGTCACCACAAGCTTCGCCCCTGCTGAAAGCGGTTTACAACCCGAAGGCCGTCATCCCGCACGCGGCGTTGCTGCATCAGGCTTCCGCCCATTGTGCAATATTCCCCACTGCTGCCTCCCGTAGGAGTCTGGGCCGTATCTCAGTCCCAATGTGGCCGGTCGCCCTCTCAGGCCGGCTACCCGTCAAAGCCTTGGTAAGCCACTACCCCACCAACAAGCTGATAAGCCGCGAGTCCATCCAAAACGCCGAAGCTTTCCAACCCCACCATGCAGCAAGGATTCCTATCCGGTATTAGCCCCAGTTTCCTGAAGTTATCCCGAAGTCAAAGGGCAGGGTTACTCACGTGTTACTCACCCGTTCGCCA
>n.538.6.bb_8
GGAGTTAGCCGGTGCTTCTTCTGCGGGTAACGTCAATCGACAAGGTTATTAACCTTATCGCCTTCCTCCCCGCTGAAAGTACTTTACAACCCGAAGGCCTTCTTCATACACGCGGCATGGCTGCATCAGGCTTGCGCCCATTGTGCAATATTCCCCACTGCTGCCTCCCGTAGGAGTCTGGACCGTGTCTCAGTTCCAGTGTGGCTGGTCATCCTCTCAGACCAGCTAGGGATCGTCGCCTAGGTGAGCCGTTACCCACCTACTAGCTAATCCCATCTGGGCACATCTGATGGCAAGAGGCCCGAAGGTCCCCCTCTTTGGTCTTGCGACGTTATGCGGTATTAGCTA
>n.538.6.bb_9
GAGTTAGCCGGTGCTTCTTCTGCGGGTAACGTCAATTGCTGAGGTTATTAACCTCAACACCTTCCTCCCCGCTGAAAGTACTTTACAACCCGAAGGCCTTCTTCATACACGCGGCATGGCTGCATCAGGCTTGCGCCCATTGTGCAATATTCCCCACTGCTGCCTCCCGTAGGAGTCTGGACCGTGTCTCAGTTCCAGTGTGGCTGGGTCATCCTCTCAGACCAGCTAGGGATCGTCGCCTAGGTGAGCCGTTACCCACCTACTAGCTAATCCCATCTGGGCACATCTGATGGCAA
>n.538.6.bb_10
GTAGTTAGCCGGTGCTTCTTCTGCAGGTACCGTCACCACAAGCTTCGCCCCTGCTGAAAGCGGTTTACAACCCGAAGGCCGTCATCCCGCACGCGGCGTTGCTGCACCAGGCTTCCGCCCATTGTGCAATATTCCCCACTGCTGCCTCCCGTAGGAGTCTGGGCCGTATCTCAGTCCCAATGTGGCCGGTCGCCCTCTCAGGCCGGCTACCCGGTCAAAGCCTTGGTAAGCCACTACCCACCAACAAGCTGATAAGCCGCGAGTCCATCCAAAACCGCCGAAG
>n.538.6.bb_11
GTAGTTAGCCGTGGCTTTCTGATTAGGTACCGTCAAGACGTGCATAGTTACTTACACATTTGTTCTTCCCTAATAACAGAGTTTTACGATCCGAAGACCTTCATCACTCACGCGGCGTTGCTCCGTCAGGCTTTCGCCCATTGCGGAAGATTCCCTACTGCTGCCTCCCGTAGGAGTCTGGACCGTGTCTCAGTTCCAGTGTGGCCGATCACCCTCTCAGGGTCGGCTACGCATCGTTGCCTTGGTAAGCCGTTACCTTACCAACTAGCTAATGCGGCGCGGATCCATCTATAAAGTGACAGCAAAAACCGTCTTTCACTATTGAACCATGCGGTTCAATATATTATCCGGTATTAGCTCCGGTTTCCCGAAGTTATCCCAGTCTTATAGGTAGGTTATCCCACGTGTTACTCACCCCGTCCCGCCCGCTAACGTCAGAGGAGCAAGCTCCCTCGTCTGTTCGCTCGACTTGCATGTATTAGGGCACGCCGCCAGCGTTCATCCTGAGCCA
>n.538.6.bb_12
GTAGTTAGCCGGTGCTTCTTCTGCAGGTACCGTCACCACAAGCTTCGCCCCTGCTGAAAGCGGTTTACAACCCGAAGGCCGTCACCCCGCACGCGGCGTTGCTGCATCAGGCTTCCGCCCATTGTGCAATATTCCCCACTGCTGCCTCCCGTAGGAGTCTGGGCCGTATCTCAGTCCCAATGTGGCCGGTCGCCCTCTCAGGCCGGCTACCCGGTCAAAGCCTTGGTAAGCCACTACCCACCAACAAGCTGATAAGCCGCGAGTCCATCCAAAACCGCCGAAGCTTTCCAACCCCACCATGCAGCAAGGATTCCTATCCGGTATTAGCCCCAGTTTCCTGAAGTTATCCCGAAG
>n.538.6.bb_13
GTAGTTAGCCGGTGCTTCTTCTGCAGGTACCGTCACCACAAGCTTCGCCCCTGCTGAAAGCGGTTTACAACCCGAAGGCCGTCATCCCGCACGCGGCGTTGCTGCATCAGGCTTCCGCCCATTGTGCAATATTCCCCACTGCTGCCTCCCGTAGGAGTCTGGGCCGTATCTCAGTCCCAATGTGGCCGGTCGCCCTCTCAGGCCGGCTACCCGTCAAAGCCTTGGTAAGCCACTACCCACCAACAAGCTGATAAGCCGCGAGTCCATCCAA
>n.538.6.bb_14
GTAGTTAGCCGGTGCTTCTTCTGCAGGTACCGTCACCACAAGCTTCGCCCCTGCTGAAAGCGGTTTACAACCCGAAGGCCGTCATCCCGCACGCGGCGTTGCTGCATCAGGCTTCCGCCCATTGTGCAATATTCCCCACTGCTGCCTCCCGTAGGAGTCTGGGCCGTATCTCAGTCCCAATGTGGCCGGTCGCCCTCTCAGGCCGGCTACCCGTCAAAGCCTTGGTAAGCCACTACCCACCAACAAGCTGATAAGCCGCGAGTCCATCCAAAACCGCCGAAGCTTTTCCAACCCCCACCATGCAGCAAGGATTCCTATCCGGTATTAGCCCCAGTTTCCTGAAGTTATCCCGAAGTCAAGGGCAGGTTACTCACGTGTTACTCACCCGTTCGCCA
>n.538.6.bb_15
GTAGTTAGCCGTGGCTTTCTGATTAGGTACCGTCAAGACGTGCATAGTTACTTACACATTTGTTCTACCCTAATAACAGAGTTTTACGATCCGAAGACCTTCATCACTCACGCGGCGTTGCTCCGTCAGGCTTTCGCCCATTGCGGAAGATTCCCTACTGCTGCCTCCCGTAGGAGTCTGGACCGTGTCTCAGTTCCAGTGTGGCCGATCACCCTCTCAGGGTCGGCTACGCATCGTTGCCTTGGTAAGCCGTTACCTTACCAACTAGCTAATGCGGCGCGGATCCATCTATAAGTGACAGCAAAACCGTCTTTCACTATTGAACCATGCGGTTCAATATATTATCCGGTATTAGCTCCGGTTTCCGAAGTTATCCCAGTCTTATAGGTAGGTTATCCCACGTGTTACTCACCCCGTCCCGCCCGCTAACGTCAGAGGGAGCAAGCCTCCCTCGTCTGTTCGCTCGACTTGCATGTATTAGGGC
>n.538.6.bb_16
GTAGTTAGCCGTGGCTTTCTGATTAGGTACCGTCAAGACGTGCATAGTTACTTACACATTTGTTCTTCCCTAATAACAGAGTTTTACGATCCGAAGACCTTCATCACTCACGCGGCGTTGCTCCGTCAGGCTTTCGCCCATTGCGGAAGATTCCCTACTGCTGCCTCCCGTAGGAGTCTGGACCGTGTCTCAGTTCCAGTGTGGCCGATCACCCTCTCAGGGTCGGCTACGCATCGTTGCCTTGGTAAGCCGTTACCTTACACTAGCTAATGCGGCGCGGATCCATCTATAAGTGACAGCAAAACCGTCTTTCACTATTGAACCATGCGGTTCAATATATTATCCGGTATTAGCTCCGGTTCCCGAAGTTATCCCAGTCTTATAGGTAGGTTATCCCACGTGTTACTCACCCCGTCCCGCCCGCTAACGTCAGAGGGAGCAAGCTCCCTCGTCTGTTCGCTCGACTTGCCATGTATTAGGGCACGCCGCCAGCGTTCATCCTGAGCCA
>n.538.6.bb_17
GTAGTTAGCCGGTGCTTCTTCTGCAGGTACCGTCACCACAAGCTTCGCCCCTGCTGAAAGCGGTTTACAACCCGAAGGCCGTCATCCCGCACGCGGCGTTGCTGCATCAGGCTTCCGCCCATTGTGCAATATTCCCCACTGCTGCCTCCCGTAGGAGTCTGGGCCGTATCTCAGTCCCAATGTGGCCGGTCGCCCTCTCAGGCCGGCTACCCGGTCAAAGCCTTGGTAAGCCACTACCCCACCAACAAGCTGATAAGCCGCGAGTCCATCCAAAAACCGCCGAAGCTTTCCAACCCCACCATGCAGCAAGGATTCCTATCCGGTATTAGCCCCAGTTTCCTGAAGTTATCCCGAAGTCAAGG
>n.538.6.bb_18
GTAGTTAGCCGTGGCTTTCTGATTAGGTACCGTCAAGACGTGCATAGTTACTTACACATTTGTTCTTCCCTAATAACAGAGTTTTACGATCCGAAGACCTTCATCACTCACGCGGCGTTGCTCCGTCAGGCTTTCGCCCATTGCGGAAGATTCCCTACTGCTGCCTCCCGTAGGAGTCTGGACCGTGTCTCAGTTCCAGTGTGGCCGATCACCCTCTCAGGGTCGGCTACGCATCGTTGCCTTGGTAAGCCGTTACCTTACCAACTAGCTAATGCGGCGCGGATCCATCTATAAGTGACAGCAAAACCGTCTTTCACTATTGAACCATGCGGTTCAATATATTATCCGGTATTAGCT
>n.538.6.bb_19
GGAGTTAGCCGGTGCTTCTTCTGCGGGTAACGTCAATTGCTGAGGTTATTAACCTCAACACCTTCCTCCCCGCTGAAAGTACTTTACAACCCGAAGGCCTTCTTCATACACGCGGCATGGCTGCATCAGGCTTGCGCCCATTGTGCAATATTCCCCACTGCTGCCTCCCGTAGGAGTCTGGACCGTGTCTCAGTTCCAGTGTGGCTGGGTCATCCTCTCAGACCAGCTAGGGATCGTCGCCTAGGTGAGCCGTTACCCCACCTACTAGCTAATCCCATCTGGGCACATCTGATGGCAAGAGGCCCGAAGGTCCCCCTCTTTGGTCTTGCGACGTTATGCGGTATTAGCTACCGTTTCCAG
>n.538.6.bb_20
GTAGTTAGCCGTGGCTTTCTGATTAGGTACCGTCAAGACGTGCATAGTTACTTACACATTTGTTCTTCCCTAATAACAGAGTTTTACGGTCCGAAGACCTTCATCACTCACGCGGCGTTGCTCCGTCAGGCTTTCGCCCATTGCGGAAGATTCCCTACTGCTGCCTCCCGTAGGAGTCTGGACCGTGTCTCAGTTCCAGTGTGGCCGATCACCCTCTCAGGGTCGGCTACGCATCGTTGCCTTGGTAAGCCGTTACCTTACCAACTAGCTAATGCGGCGCGGATCCATCCATAAGTGACAGCAAAACCGTCTTTCACTATTGAACCATGCGGTTCAATATATTATCCGGTATTAGCTCCGGTTTCCCCGAAGTTATCCCAGTCTTATAGGTAGGTTATCCACCGTGTTACTCACCCCGTCCCGCCCGCTAACGTCAGAGGAGCAAGCTCCCTCGTCCTGTTCCGCTCGACTTGCATGTATTAGGGCACGCC
>n.538.6.bb_21
GTAGTTAGCCGGTGCTTCTTCTGCAGGTACCGTCACCACAAGCTTCGCCCCTGCTGAAAGCGGTTTACAACCCGAAGGCCGTCATCCCGCACGCGGCGTTGCTGCATCAGGCTTCCGCCCATTGTGCAATATTCCCCACTGCTGCCTCCCGTAGGAGTCTGGGCCGTATCTCAGTCCCAATGTGGCCGGTCGCCCTCTCAGGCCGGCTACCCGTCAAAGCCTTGGTAAGCCACTACCCACCAACAAGCTGATAAGCCGCGAGTCCATCCAAAACCGCCGAAGCTTTCCAACCCCCACCATGCAGCAAGGATTCCTATCCGGTATTAGCCCCAGTTTCCTGAAGTTATCCCGAAGCCCAAGGGCAGGTTACTCACGTGTTACTCACCCGTT
>n.538.6.bb_22
GTAGTTAGCCGGTGCTTCTTCTGCAGGTACCGTCACCACAAGCTTCGCCCCTGCTGAAAGCGGTTTACAACCCGAAGGCCGTCATCCCGCACGCGGCGTTGCTGCATCAGGCTTCCGCCCATTGTGCAATATTCCCCACTGCTGCCTCCCGTAGGAGTCTGGGCCGTATCTCAGTCCCAATGTGGCCGGTCGCCCTCTCAGGCCGGCTACCCGTCAAAGCCTTGGTAAGCCACTACCCACCAACAAGCTGATAAGCCGCGAGTCCATCCAAAACCGCCGAAGCTTTCCAACCCCACCATGCAGCAAGGATTCCTATCCGGTATTAGCCCCAGTTTCCTGAAGTTATCCCGAAGTCAAGGGCAGGTTACTCACGTGTTACTCACCCGTTCGCCACTCGACGT
>n.538.6.bb_23
GTAGTTAGCCGGTGCTTCTTCTGCAGGTACCGTCACCACAAGCTTCGCCCCTGCTGAAAGCGGTTTACAACCCGAAGGCCGTCATCCCGCACGCGGCGTTGCTGCATCAGGCTTCCGCCCATTGTGCAATATTCCCCACTGCTGCCTCCCGTAGGAGTCTGGGCCGTATCTCAGTCCCAATGTGGCCGGTCGCCCTCTCAGGCCGGCTACCCGTCAAAGCCTTGGTAAGCCACTACCCCACCAACAAGCTGATAAGCCGCGAGTCCATCCAAAACCGCCGAAGCTTTCCAACCCCACCATGCAGCAAGGATTCCTATCCGGTATTAGCCCCAGTTTCCTGAAGTTATCCCGAAGTCAAAGGGCAGGTTACTCACGTGTACTCACCCGTT
>n.538.6.bb_24
GTAGTTAGCCGTGGCTTTCTGATTGGGTACCGTCGAGACGTGCATAGTTACTTACACATTTGTTCTTCCCTAATAACAGAGTTTTACGATCCGAAGACCTTCATCACTCACGCGGCGTTGCTCCGTCAGGCTTTCGCCCATTGCGGAAGATTCCCTACTGCTGCCTCCCGTAGGAGTCTGGGCCGTATCTCAGTCCCAATGTGGCCGGTCGCCCTCTCAGGGCCGGGCTACCCCGTCAAAGCCTTGGTAAGCCACTACCCCACCAACAAGCTGATAAGCCGCGAGTCCATCCAAAACCGCCGAAGCTTTCCAACCCCACCATGCAGCAAGGATTCCTATCCGGTATTAGCCCCAGTTT
>n.538.6.bb_25
GTAGTTAGCCGGTGCTTCTTCTGCAGGTACCGTCACCACAAGCTTCGCCCCTGCTGAAAGCGGTTTACAACCCGAAGGCCGTCATCCCGCACGCGGCGTTGCTGCATCAGGCTTCCGCCCATTGTGCAATATTCCCCACTGCTGCCTCCCGTAGGAGTCTGGGCCGTATCTCAGTCCCAATGTGGCCGGTCGCCCTCTCAGGCCGGCTACCCGTCAAAGCCTTGGTAAGCCACTACCCACCAACAAGCTGATAAGCCGCGAGTCCATCCAAAACCGCCGAAGCTTTCCAACCCCACCATGCAGCAAGGATTCCTATCCGGTATTAGCCCCAGTTTCCTGAAGTTATCCCGAAG
>n.538.6.bb_26
GGAGTTAGCCGGTGCTTCTTCTGCGGGTAACGTCAATCGACAGGGTTATTAACCCTGTCGCCTTCCTCCCCGCTGAAAGTACTTTACAACCCGAAGGCCTTCTTCATACACGCGGCATGGCTGCATCAGGCTTGCGCCCATTGTGCAATATTCCCCACTGCTGCCTCCGTAGGAGTCTGGACCGTGTCTCAGTTCCAGTGTGGCTGGGTCATCCTCTCAGACCAGCTAGGGATCGTCGCCTAGGTGAGCCGTTACCACCTACTAGCTAATCCCATCTGGGCACATCCGATGGC
>n.538.6.bb_27
GTAGTTAGCCGTGGCTTTCTGATTAGGTACCGTCAAGACGTGCATAGTTACTTACACATTTGTTCTTCCCTAATAACAGAGTTTTACGATCCGAAGATCTTCATCACTCACGCGGCGTTGCTCCGTCAGGCTTTCGCCCATTGCGGAAGATTCCCTACTGCTGCCTCCCGTAGGAGTCTGGACCGTGTCTCAGTTCCAGTGTGGCCGATCACCCTCTCAGGTCGGCTACGCATCGTTGCCTTGGTAAGCCGTTACCTTACCAACTAGCTAATGCGGCGCGGATCCATCTATAAGTGACAGCAAAACCGTCTTTCACTATTGAACCATGCGGTTCAATATATTATCCCGGTATTAGCTCCGGTTTCCCGAAGTTTATCCCAGTCTTATAGGTAGGTTATCCCACGTGTTACTCACCCCGTCCCGCCCGCTAACGTCAGAGAGCAAGCTCCCTCGTCTGTTCGCTCGACTTGCATGTATTAGGGCACGCCGCCAGCGTT
>n.538.6.bb_28
GGAGTTAGCCGGTGCTTCTTCTGCGGGTAACGTCAATCGACAGGGTTATTAACCCTGTCGCCTTCCTCCCCGCTGAAAGTACTTTACAACCCGAAGGCCTTCTTCATACACGCGGCATGGCTGCATCAGGCTTGCGCCCATTGTGCAATATTCCCCACTGCTGCCTCCCGTAGGAGTCTGGACCGTGTCTCAGTTCCAGTGTGGCTGGTCATCCTCTCAGACCAGCTAGGGATCGTCGCCTAGGTGAGCCGTTACCCACCTACTAGCTAATCCCATCTGGGCACATCCGATGGCAAGAGG
>n.538.6.bb_29
GTAGTTAGCCGGTGCTTCTTCTGCAGGTACCGTCACCACAAGCTTCGCCCCTGCTGAAAGCGGTTTACAACCCGAAGGCCGTCATCCCGCACGCGGCGTTGCTGCATCAGGCTTCCGCCCATTGTGCAATATTCCCCACTGCTGCCTCCCGTAGGAGTCTGGGCCGTATCTCAGTCCCAATGTGGCCGGTCGCCCTCTCAGGCCGGCTACCCGTCAAAGCCTTGGTAAGCCACTACCCACCAACAAGCTGATAAGCCGCGAGTCCATCCAAAACCGCCGAAGCTTTCCAACCCCACCATGCAGCAAGGATTCCTATCCGGTATTAGCCCCAGTTTCCTGAAGTTATCCCGAAGTCAAGGGCAGGTTACTCACGTGTTACTCACCCGTTCG
>n.538.6.bb_30
GTAGTTAGCCGTGGCTTTCTGATTAGGTACCGTCAAGACGTGCATAGTTACTTACACATTTGTTCTTCCCTAATAACAGAGTTTTACGATCCGAAGACCTTCATCACTCACGCGGCGTTGCTCCGTCAGGCTTTCGCCCATTGCGGAAGATTCCCTACTGCTGCCTCCCGTAGGAGTCTGGACCGTGTCTCAGTTCCAGTGTGGCCGATCACCCTCTCAGGGTCGGCTACGCATCGTTGCCTTGGTAAGCCGTTACCTTACCAACTAGCTAATGCGGTGCGGATCCATCTATAAGTGACAGCAAAACCGTCTTTCACTATTGAACCATGCGGTTCAATATATTATCCGGTATTAGCTCCGGTTTCCCGAAGTATCCCAGTCTTATAGGTAGGTTATC
>n.538.6.bb_31
GTAGTTAGCCGTGGCTTTCTGATTAGGTACCGTCAAGACGTACATAGTTACTTACACATTTGTTCTTCCCTAATAACAGAGTTTTACGATCCGAAGACCTTCATCACTCACGCGGCGTTGCTCCGTCAGGCTTTCGCCCATTGCGGAAGATTCCCTACTGCTGCCTCCCGTAGGAGTCTGGACCGTGTCTCAGTTCCAGTGTGGCCGATCACCCTCTCAGGGTCGGCTACGCATCGTTGCCTTGGTAAGCCGTTACCTTACCAACTAGCTAATGCGGCGCGGATCCATCTATAAGTGACAGCAAAAACCGTCTTTCACTATTGAACCATGCAGTTCAATATATTATCCGGTATTAGCTCC
>n.538.6.bb_32
GTAGTTAGCCGGTGCTTCTTCTGCAGGTACCGTCACCACAAGCTTCGCCCCTGCTGAAAGCGGTTTACAACCCGAAGGCCGTCATCCCGCACGCGGCGTTGCTGCATCAGGCTTCCGCCCATTGTGCAATATTCCCCACTGCTGCCTCCCGTAGGAGTCTGGGCCGTATCTCAGCCCCAATGTGGCCGGTCGCCCTCTCAGGCCGGCTACCCGTCAAAGCCTTGGTAAGCCACTACCCACCAACAAGCTGATAAGCCGCGAGTCCATCCAAAACCGCCGAAGCTTTCCAACCCCACCATGCAGCAAGGATTCCTATCCGGTATTAGCCCCAGTTTCCTGAAGTTATCCCGAAGTCAAGGGCAGGTACTCACGTGTTA
>n.538.6.bb_33
GGAGTTAGCCGGTGCTTCTTCTGCGGGTAACGTCAATTGCTGAGGTTATTAACCTCAACACCTTCCTCCCCGCTGAAAGTACTTTACAACCCGAAGGCCTTCTTCATACACGCGGCATGGCTGCATCAGGCTTGCGCCCATTGTGCAATATTCCCCACTGCTGCCTCCCGTAGGAGTCTGGACCGTGTCTCAGTTCCAGTGTGGCTGGGTCATCCTCTCAGACCAGCTAGGGATCGTCGCCTAGGTGAGCCGTTACCCCACCTACTAGCCAATCCCATCTGGGCACATCTGATGGCAAGAGGCCCGAAGGTCCCCCTCTTTGGTCTTGCGACGTTATGCGGTATTAGCTACCGTTTCCA
>n.538.6.bb_34
GTAGTTAGCCGTGGCTTTCTGATTAGGTACCGTCAAGACGTGCATAGTTACTTACACATTTGTTCTTCCCTAATAACAGAGTTTTACGATCCGAAGACCTTCATCACTCACGCGGCGTTGCTCCGTCAGGCTTTCGCCCATTGCGGAAGATTCCCTACTGCTGCCTCCCGTAGGAGTCTGGACCGTGTCTCAGTTCCAGTGTGGCCGATCACCCTCTCAGGGTCGGCTACGCATCGTTGCCTTGGTAAGCCGTTACCTTACCAACTAGCTAATGCGGCGCGGATCCATCTATAAGTGACAGCAAAACCGTCTTTCACTATTGAACCATGCGGTTCAATAT
>n.538.6.bb_35
GTAGTTAGCCGTGGCTTTCTGATTAGGTACCGTCAAGACGTGCATAGTTACTTACACATTTGTTCTTCCCTAATAACAGAGTTTTACGATCCGAAGACCTTCATCACTCACGCGGCGTTGCTCCGTCAGGCTTTCGCCCATTGCGGAAGATTCCCTACTGCTGCCTCCCGTAGGAGTCTGGACCGTGTCTCAGTTCCAGTGTGGCCGATCACCCTCTCAGGGTCGGCTACGCATCGTCGCCTTGGTAAGCCGTTACCTTACCAACTAGCTAATGCGGCGCGGATCCATCTATAAGTGACAGCAAAACCGTCTTTCACTATTGAACCATGCGGTTCAATATATTATCCGGTATTAGCTCCGGT
>n.538.6.bb_36
GTAGTTAGCCGGTGCTTCTTCTGCAGGTACCGTCACCACAAGCTTCGCCCCTGCTGAAGGCGGTTTACAACCCGAAGGCCGTCATCCCGCACGCGGCGTTGCTGCATCAGGCTTCCGCCCATTGTGCAATATTCCCCACTGCTGCCTCCCGTAGGAGTCTGGGCCGTATCTCAGTCCCAATGTGGCCGGTCGCCCTCTCAGGCCGGCTACCCGTCAAAGCCTTGGTAAGCCACTACCCACCAACAAGCTGATAAGCCGCGAGTCCATCCAAAACCGCCGAAGCTTTTCCAACCCCACCATGCAGCAAGGATTCCTATCCGGTATTAGCCCCAGTTTCCTGAAGTT
>n.538.6.bb_37
GGAGTTAGCCGGTGCTTCTTCTGCGGGTAACGTCAATTGCTGAGGTTATTAACCTCAACACCTTCCTCCCCGCTGAAAGTACTTTACAACCCGAAGGCCTTCTTCATACACGCGGCATGGCTGCATCAGGCTTGCGCCCATTGTGCAATATTCCCCACTGCTGCCTCCCGTAGGAGTCTGGACCGTGTCTCAGTTCCAGTGTGGCTGGGTCATCCTCTCAGACCAGCTAGGGATCGTCGCCTAGGTGAGCCGTTACCCACCTACTAGCTAATCCCATCTGGGCACATCTGATGGCAAGAGGCCCGAAGGTCCCCCTCTTTGGTCTTGCGACGTTATGCGGTATTAGC
>n.538.6.bb_38
GTAGTTAGCCGGTGCTTCTTCTGCAGGTACCGTCACCACAAGCTTCGCCCCTGCTGAAAGCGGTTTACAACCCGAAGGCCGTCATCCCGCACGCGGCGTTGCTGCATCAGGCTTCCGCCCATTGTGCAATATTCCCCACTGCTGCCTCCCGTAGGAGTCTGGGCCGTATCTCAGTCCCAATGTGGCCGGTCGCCCTCTCAGGCCGGCTACCCGTCAAAGCCTTGGTAAGCCACTACCCCACCAACAAGCTGATAAGCCGCGAGTCCATCCAAAACCGCCGAAGCTTTCCAACCCCACCATGCAGCAAGGATTCCTATCCGGTATTAGCCCCAGTTTCCTGAAGTTATCCCGAAGTCAA
>n.538.6.bb_39
TAGTTAGCCGTGGCTTTCTGATTAGGTACCGTCAAGACGTGCATAGTTACTTACACATTTGTTCTTCCCTAATAACAGAGTTTTACGATCCGAAGACCTTCATCACTCACGCGGCGTTGCTCCGTCAGGCTTTCGCCCATTGCGGAAGATTCCCTACTGCTGCCTCCCGTAGGAGTCTGGACCGTGTCTCAGTTCCAGTGTGGCCGATCACCCTCTCAGGGTCGGCTACGCATCGTTGCCTTGGTAAGCCGTTACCTTACCAACTAGCTAATGCGGCGCGGATCCATCTATAAGTGACAG
>n.538.6.bb_40
GTAGTTAGCCGGTGCTTCTTCTGCAGGTACCGTCACCACAAGCTTCGCCCCTCGCTGAAAGACGGTTTACTAACCCGAACGGCCGTCATCCCGCCACGCGGCGTTGCTGCATCAGGCTTCCGCCCATTGTGCAATATTCCCCACTGCTGCCTCCCGTAGGAGTCTGGGCCGTATCTCAGTCCCAATGTGGCCGGTCGCCCTCTCAGGCCGGCTACCCGTCAAAGCCTTGGTAAGCCACTACCCACCAACAAGCCGATAAGCCGCGAGT
>n.538.6.bb_41
GTAGTTAGCCGGTGCTTCTTCTGCAGGTACCGTCACCACAAGCTTCGCCCCTGCTGAAAGCGGTTTACAACCCGAAGGCCGTCATCCCGCACGCGGCGTTGCTGCATCAGGCTTCCGCCCATTGTGCAATATTCCCCACTGCTGCCTCCCGTAGGAGTCTGGGCCGTATCTCAGTCCCAATGTGGCCGGTCGCCCTCTCAGGCCGGCTACCCGTCAAAGCCTTGGTAAGCCACTACCCACCAACAAGCTGATAAGCCGCGAGTCCAT
>n.538.6.bb_42
GTAGTTAGCCGTGGCTTTCTGATTAGGTACCGTCAAGACGTGCATAGTTACTTACACATTTGTTCTTCCCTAATAACAGAGTTTTACGATCCGAAGACCTTCATCACTCACGCGGCGTTGCTCCGTCAGGCTTTCGCCCATTGCGGAAGATTCCCTACTGCTGCCTCCCGTAGGAGTCTGGACCGTGTCTCAGTTCCAGTGTGGCCGATCACCCTCTCAGGGTCGGCTACGCATCGTTGCCTTGGTAAGCCGTTACCTTACCAACTAGCTAATGCGGCGCGGATCCATCTATAAGTGACAGCAAAACCGTCTTTCACTATTGAACCATGCGGTTCAATATATTATCCGGTATTAGCTCCGGTTTCCCGAAGTTATCCCAGTCTTATAGGTAGGTTATCCCACCGTGTTACTCACCCCGTCCCGCCCGCTAACGTCAGAGGAGCAAGCTCCCTCGTCTGTTCGC
>n.538.6.bb_43
GTAGTTAGCCGGTGCTTCTTCTGCAGGTACCGTCACCACAAGCTTCGCCCCTGCTGAAAGCGGTTTACAACCCGAAGGCCGTCATCCCGCACGCGGCGTTGCTGCATCAGGCTTCCGCCCATTGTGCAATATTCCCCACTGCTGCCTCCCGTAGGAGTCTGGGCCGTAGTCTCAGTCCCAATGTGGCCGGTCGCCCTCTCAGGCCGGCTACCCGGTCAAAGCCTTGGTAAGCCACTACCCACCAACAAGCTGATAAGCCGCGAGTCCATCC
>n.538.6.bb_44
GAGTTAGCCGGTGCTTCTTCTGCGGGTAACGTCAATCGACAAGGTTATTAACCTTATCGCCTTCCTCCCCGCTGAAAGTACTTTACAACCCGAAGGCCTTCTTCATACACGCGGCATGGCTGCATCAGGCTTGCGCCCATTGTGCAATATTCCCCACTGCTGCCTCCCGTAGGAGTCTGGACCGTGTCTCAGTTCCAGTGTGGCTGGTCATCCTCTCAGACCAGCTAGGGATCGTCGCCTAGGTGAGCCGTTACCCACCTACTAGCTAATCCCATCTGGGCACATCTGATGGCAAGAGGCCCGAAGGTCCCCTCTTTGGTCTTGCGACGTTATGCGGTATTAGCTACC
>n.538.6.bb_45
GTAGTTAGCCGGTGCTTCTTCTGCAGGTACCGTCACCACAAGCTTCGCCCCTGCTGAAAGCGGTTTACAACCCGAAGGCCGTCATCCCGCACGCGGCGTTGCTGCATCAGGCTTCCGCCCATTGTGCAATATTCCCCACTGCTGCCTCCCGTAGGAGTCTGGGCCGTATCTCAGTCCCAATGTGGCCGGTCGCCCTCTCAGGCCGGCTACCCGTCAAAGCCTTGGTAAGCCACTACCCCACCAACAAGCTGATAAGCCGCGAGTCCATCCAAAACCGCCGAAGCTTTCCAACCCCACCATGCAGCAAGGATTCCTATCCGGTATTAGCCCCAGTTTCCTGAAGTTATCCCGAAGTCAAGGGCAGGTTACTCACGTGTTACTCACCCGTTCG
>n.538.6.bb_46
GTAGTTAGCCGTGGCTTTCTGATTAGGTACCGTCAAGACGTGCATAGTTACTTACACATTTGTTCTTCCCTAATAACAGAGTTTTACGATCCGAAGACCTTCATCACTCACGCGGCGTTGCTCCGTCAGGCTTTCGCCCATTGCGGAAGATTCCCTACTGCTGCCTCCCGTAGGAGTCTGGACCGTGTCTCAGTTCCAGTGTGGCCGATCACCCTCTCAGGGTCGGCTACGCATCGTCGCCTTGGTAAGCCGTTACCTTACCAACTAGCTAATGCGGCGCGGATCCATCTATAAGTGACAGCAAAACCGTC
>n.538.6.bb_47
GTAGTTAGCCGGTGCTTCTTCTGCAGGTACCGTCACCACAAGCTTCGCCCCTGCTGAAAGCGGTTTACAACCCGAAGGCCGTCATCCCGCACGCGGCGGTGCTGCATCAGGCTTCCGCCCATTGTGCAATATTCCCCACTGCTGCCTCCCGTAGGAGTCTGGGCCGTATCTCAGTCCCAATGTGGCCGGTCGCCCTCTCAGGCCGGCTACCCGTCAAAGCCTTGGTAAGCCACTACCCCACCAACAAGCTGATAAGCCGCGAGTCCATCCAAAACCGCCGAAGCTTTTCCAACCCCACCATGCAGCAAGGATTCCTATCCGGTATTAGCCCCAGTTTCCTGAAGTTATCCCGAAGTCAAGGGCAGGTTACTCACGTGTTACTCACCCGTTCGCCACTCGACGT
>n.538.6.bb_48
GGAGTTAGCCGGTGCTTCTTCTGCGGGTAACGTCAATCGACAAGGTTATTAACCTTATCGCCTTCCTCCCCGCTGAAAGTACTTTACAACCCGAAGGCCTTCTTCATACACGCGGCATGGCTGCATCAGGCTTGCGCCCATTGTGCAATATTCCCCACTGCTGCCTCCCGTAGGAGTCTGGACCGTGTCTCAGTTCCAGTGTGGCCGATCACCCTNTCAGGTCGCTACGCATCGTTGCCTTGGTAAGCCGTTACCTTACCAACTAGCTAATGCGGCGCGGATCCATCTATAAGTGACAGCAAAACCGTCTTTCACTATTGAACCATGCGGTTCAATATATTATCCGGTATTAGCTCCGGTTTCCCGAAGTTATCCCAGTCTTATAGGTAGGTTATCCCACGTGTTACTCACCCCGTCCCGCCCGCTAACGTCAGAGGAGCAAGCCTCCTCGTCTGTTCGCTCGACTTGCATGTATTAGGGCACGCCGCCAGCGTTCATCCTGA
>n.538.6.bb_49
GTAGTTAGCCGGTGCTTCTTCTGCAGGTACCGTCACCACAAGCTTCGCCCCTGCTGAAAGCGGTTTACAACCCGAAGGCCGTCATCCCGCACGCGGCGTTGCTGCATCAGGCTTCCGCCCATTGTGCAATATTCCCCACTGCTGCCTCCCGTAGGAGTCTGGGCCGTATCTCAGTCCCAATGTGGCCGGTCGCCCTCTCAGGCCGGCTACCCGTCAAAGCCTTGGTAAGCCACTACCCCACCAACAAGCTGATAAGCCGCGAGTCCATCCAAAACCGC
>n.538.6.bb_50
GTAGTTAGCCGTGGCTTTCTGATTAGGTACCGTCAAGACGTGCATAGTTACTTACACATTTATTCTTCCCTAATAACAGAGTTTTACGATCCGAAGACCTTCATCACTCACGCGGCGTTGCTCCGTCAGGCTTTCGCCCATTGCGGAAGATTCCCTACTGCTGCCTCCCGTAGGAGTCTGGACCGTGTCTCAGTTCCAGTGTGGCCGATCACCCTCTCAGGGTCGGCTACGCATCGTTGCCTTGGTAAGCCGTTACCTTACCACTAGCTAATGCGGCGCGGATCCATCTATAAGTGACAGCAAAACCGTCTTTCACTATTGAACCATGCGGTTCAATATATTATCCGGTATTAGCTCCGGTTTCCCGAGTTATCCCAGTCTTATAGGTAGGTTATCCCACGTGTT
>n.538.6.bb_51
GTAGTTAGCCGTGGCTTTCTGATTAGGTACCGTCAAGACGTGCATAGTTACTTACACATTTGTTCTTCCCTAATAACAGAGTTTTACGATCCGAAGGCCTTCATCACTCACGCGGCGTTGCTCCGTCAGGCTTTCGCCCATTGCGGAAGATTCCCTACTGCTGCCTCCCGTAGGAGTCTGGACCGTGTCTCAGTTCCAGTGTGGCCGATCACCCTCTCAGGGTCGGCTACGCATCGTTGCCTTGTAAGCCGTTACCTTACCAACTAGCTAATGCGGCGCGGATCCATCTATAAGTGACAGCAAAAACCGTCTTTCACTATTTGAACCATGCGGTTCAATATATTATCCGGTATTAGC
>n.538.6.bb_52
GTAGTTAGCCGGTGCTTCTTCTGCAGGTACCGTCACCACAAGCTTCGCCCCTGCTGAAAGCGGTTTACAACCCGAAGACCGTCATCCCGCACGCGGCGTTGCTGCATCAGGCTTCCGCCCATTGTGCAATATTCCCCACTGCTGCCTCCCGTAGGAGTCTGGGCCGTATCTCAGTCCCAATGTGGCCGGTCGCCCTCTCAGGCCGGCTACCCGGTCAAAGCCTTGGTAAGCCACTACCCCACCAACAAGCTGATAAGCCGCGAGTCCATCCAAAACCGCCGAAAGCTTTCCAACCCCACCATGCAGCAAAGGATTCCTATCCGGTATTAGCCCCAGTTTCCTGAAGTTATCCCGAAAGTC
>n.538.6.bb_53
GTAGTTAGCCGGTGCTTCTTCTGCAGGTACCGTCACCACAAGCTTCGCCCCTGCTGAAAGCGGTTTACAACCCGAAGGCCGTCATCCCGCACGCGGCGTTGCTGCATCAGGCTTCCGCCCGTTGTGCAATATTCCCCACTGCTGCCTCCCGTAGGAGTCTGGGCCGTATCTCAGTCCCAAATGTGGCCGGTCGCCCTCTCAGGCCGGCTACCCGTCAAAAGCCTTGGTAAGCCACTACCCACCAACAAGCTGATAAGCCGCGAGTCCATCC
>n.538.6.bb_54
GTAGTTAGCCGGTGCTTCTTCTGCAGGTACCGTCACCACAAGCTTCGCCCCTGCTGAAAGCGGTTTACAACCCGAAGGCCGTCATCCCGCACGCGGCGTTGCTGCATCAGGCTTCCGCCCATTGTGCAATATTCCCCACTGCTGCCTCCCGTAGGAGTCTGGGCCGTATCTCAGTCCCAATGTGGCCGGTCGCCCTCTCAGGCCGGCTACCCGTCAAAGCCTTGGTAAGCCACT
>n.538.6.bb_55
GTAGTTAGCCGTGGCTTTCTGATTAGGTACCGTCAAGACGTGCATAGTTACTTACACATTTGTTCTTCCCTAATAACAGAGTTTTACGATCCGAAGACCTTCATCACTCACGCGGCGTTGCTCCGTCAGGCTTTCGCCCATTGCGGAAGATTCCCTACTGCTGCCTCCCGTAGGAGTCTGGACCGTGTCTCAGTTCCAGTGTGGCCGATCACCCTCTCAGGGTCGGCTACGCATCGTTGCCTTGGTAAGCCGTTACCTTACCAACTAGCTAATGCGGCGCGGATCCATCTATAAGTGACAGCAAAACCGTCTTTCACTATTGAACCATGCGGTTCAATATATTATCCGGTATTAGCTCCGGTTTCCCGAAGTTATCCCAGTCTTATAGGTAGGTTATCCCACCGTGTTACCTCACCCCGTCCCGCCCGCTAACGTCAGAGGGAGCAAGCTCCCTCGTCTGTTCGCTCGACTTGCATGTATTAGGGCACGCCGCCAGCGTTCATCCT
>n.538.6.bb_56
GTAGTTAGCCGGTGCTTCTTCTGCAGGTACCGTCACCACAAGCTTCGCCCCTGCTGAAAGCGGTTTACAACCCGAAGGCCGTCATCCCGCACGCGGCGTTGCTGCATCAGGCTTCCGCCCATTGTGCAATATTCCCCACTGCTGCCTCCCGTAGGAGTCTGGGCCGTATCTCAGTCCCAATGTGGCCGGTCGCCCTCTCAGGCCAGGCTACCCGTCAAAGCCTTGGTAAGCCACTACCCACCAACAAGCTGATAAGCCGCGAGTCCATCCAAAACGCCGAAGCTTTCCAACCCCACCATGCAGCAAGGATTCCTATCCGGTATTAGCCCAGTCTCCTGAAGTTATCCCCGAAGTCAAGGGCAGGTTACTCACGTGTTACTCACCCGTTCGCCCA
>n.538.6.bb_57
GGAGTTAGCCGGTGCTTCTTCTGCGGGTAACGTCAATTGCTGAGGTTATTAACCTCAACACCTTCCTCCCCGCTGAAAGTACTTTACAACCCGAAGGCCTTCTTCATACACGCGGCATGGCTGCATCAGGCTTGCGCCCATTGTGCAATATTCCCCACTGCTGCCTCCCGTAGGAGTCTGGACCGTGTCTCAGTTCCAGTGTGGCTGGGTCATCCTCTCAGACCAGCTAGGGATCGTCGCCTAGGTGAGCCGTTACCCACCTACTAGCTAATCCCATCTGGGCACATCTGATGGCAA
>n.538.6.bb_58
GTAGTTAGCCGTGGCTTTCTGATTAGGTACCGTCAAGACGTGCATAGTTACTTACACATTTGTTCTTCCCTAATAACAGAGTTTTACGATCCGAAGACCTTCATCACTCACGCGGCGTTGCTCCGTCAGGCTTTCGCCCATTGCGGAAGATTCCCTACTGCTGCCTCCCGTAGGAGTCTGGACCGTGTCTCAGTTCCAGTGTGGCCGATCACCCTCTCAGGGTCGGCTACGCATCGTTGCCTTGGTAAGCCGTTACCTTACCAACTAGCTAATGCGGCGCGGATCCATCTATAAGTGACAGCAAAACCGTCTTTCACTATTGAACCATGCGGTTCAATATATTATCCGGTATTAGCT
>n.538.6.bb_59
GTAGTTAGCCGTGGCTTTCTGATTAGGTACCGTCAAGACGTGCATAGTTACTTACACATTTGTTCTTCCCTAATAACAGAGTTTTACGATCCGAAGACCTTCATCACTCACGCGGCGTTGCTCCGTCAGGCTTTCGCCCATTGCGGAAGATTCCCTACTGCTGCCTCCCGTAGGAGTCTGGACCGTGTCTCAGTTCCAGTGTGGCCGATCACCCTCTCAGGGTCGGCTACGCATCGTTGCCTTGGTAAGCCGTTACCTTACCAACTAGCTAATGCGGCGCGGATCCATCTATAAGTGACAGCAAAACCGTCTTTCACTATTGAACCATGCGGTTCAATATATTATCCGGTATTAGCTCCGGTTTCCCGAAGTTATCCCAGTCTTATAGGTAGGTTATCCCACCGTGTTACCTCACCCCGTCCCGCCCGCTAACGTCAGAGGGAGCAAGCTCCCTCGTCTGTTCGCTCGACTTGCATGTATTAGGGCACGCCGCCAGCGTTCATCCTGA
>n.538.6.bb_60
GTAGTTAGCCGTGGCTTTCTGATTAGGTACCGTCAAGACGTGCATAGTTACTTACACATTTGTTCTTCCCTAATAACAGAGTTTTACGATCCGAAGACCTTCATCACTCACGCGGCGTTGCTCCGTCAGGCTTTCGCCCATTGCGGAAGATTCCCTACTGCTGCCTCCCGTAGGAGTCTGGACCGTGTCTCAGTTCCAGTGTGGCCGATCACCCTCCCAGGGTCGGCTACGCATCGTTGCCTTGGTAAGCCGTTACCTTACCAACTAGCTAACGCGGCGCGGATCCATCTATAAGTGACAGCAAAACCGTCTTTCACTATTAAACCATGCGGTTCAATATATTATCCGGTATTAGCTCCGGTTTCCCCGAAGTTATCCCAGTCTTATAGGTAGGTTATCCACGTGTTACCTCCACCCCGTCCCGCCCGCTAACGTCAGAGGAGCAAGCTCCTCGTCTGTTCGCTCGACTTGCATGTATTAGGGCCACGCCGCCAGCGTTCATCCTGAGCCAGGGATCAAAC
>n.538.6.bb_61
GTAGTTAGCCGGTGCTTCTTCTGCAGGTACCGTCACCACAAGCTTCGCCCCTGCTGAAAGCGGTTTACAACCCGAAGGCCGTCATCCCGCACGCGGCGTTGCTGCATCAGGCTTCCGCCCATTGTGCAATATTCCCCACTGCTGCCTCCCGTAGGAGTCTGGGCCGTATCTCAGTCCCAATGTGGCCGGTCGCCCTCTCAGGCCGGCTACCCGTCAAAGCCTTGGTAAGCCACTACCCCACCAACAAGCTGATAAGCCGCGAGTCCATCCAAAACCGCCGAAGCTTTCCAACCCCACCATGCAGCAAGGATTCCTATCCGGTATTAGCCCCAGTTTCCTGAAGTTATCCCGAAGTCAAGGGCAGGTTACTCACGTGTTACTCACCCGTT
>n.538.6.bb_62
GTAGTTAGCCGTGGCTTTCTGATTAGGTACCGTCAAGACGTGCATAGTTACTTACACATTTGTTCTTCCCTAATAACAGAGTTTTACGATCCGAAGACCTTCATCACTCACGCGGCGTTGCTCCGTCAGGCTTTCGCCCATTGCGGAAGATTCCCTACTGCTGCCTCCCGTAGGAGTCTGGACCGTGTCTCAGTTCCAGTGTGGCCGATCACCCTCTCAGGGTCGGCTACGCATCGTCGCCTTGGTAAGCCGTTACCTTACCAACTAGCTAATGCGGCGCGGATCCATCTATAAGTGACAGCAAAACCGTCTTTCACTATTGAACCATGCGGTTCAATATATTATCCGGTATTAGCTCCGGTTTCCGAAGTTATCCCAGTCTTATAGGTAGGTTATCCCACGTGTTACCTCACCCCGTCCCGCCCGCTAACGTCAGAGGGAGCAAGCTCCCTCATCTGTTCGCTCGACTTGCCATGTATTAGGGCACGCCGCCAGCGTT
>n.538.6.bb_63
GTAGTTAGCCGTGGCTTTCTGATTAGGTACCGTCAAGACGTGCATAGTTACTTACACATTTGTTCTTCCCTAATAACAGAGTTTTACGATCCGAAGACCTTCATCACTCACGCGGCGTTGCTCCGTCAGGCTTTCGCCATTGCGGAAGATTCCCTACTGCTGCCTCCCGTAGGAGTCTGGACCGTGTCTCAGTTCCAGTGTGGCCGATCACCCTCTCAGGGTCGGCTACGCATCGTGCCTTGTAAGCCGTTACCTTACACTAGCTAATGCGGCGCGGATCCATCTATAAGTGACAGCAAAACCGTCTTTCACTATTGAACCATGCGGTTCAATATATTATCCGGTATTAGCTCCGGT
>n.538.6.bb_64
GAAGTTAGCCGGTGCTTCTTCTGCGGGTAACGTCAATCGATGAGGTTATTAACCTCACCGCCTTCCTCCCCGCTGAAAGTGCTTTACAACCCGAAGGCCTTCTTCACACACGCGGCATGGCTGCATCAGGCTTGCGCCCATTGTGCAATATTCCCCACTGCTGCCTCCCGTAGGAGTCTGGACCGTGTCTCAGTTCCAGTGTGGCTGGGTCATCCTCTCAGACCAGCTAGGGATCGTCGCCTAGGTGAGCCGTTACCCACCTACCAGCTAATCCCATCTGGGCACATCTGATGGCATGAGGCCCGAAGGTCCCCACTTTGGTCTTGCGACATTATGCGGTATTAGCTACCGTTTCCAGTAGTT
>n.538.6.bb_65
GTAGTTAGCCGTGGCTTTCTGATTAGGTACCGTCAAGACGTGCATAGTTACTTACACATTTGTTCTTCCCTAATAACAGAGTTTTACGATCCGAAGACCTTCATCACTCACGCGGCGTTGCTCCGTCAGGCTTTCGCCCATTGCGGAAGATTCCCTACTGCTGCCTCCCGTAGGAGTCTGGACCGTGTCTCAGTTCCAGTGTGGCCGATCACCCTCTCAGGGTCGGCTACGCATCGTTGCCTTGGTAAGCCGTTACCTTACCAACTAGCTAATGCGGCGCGGATCCATCTATAAGTGACAGCAAAACCGTCTTTCACTATTGAACCATGCGGTTCAATATATTATCCGGTATTAGCTCCGGT
>n.538.6.bb_66
GTAGTTAGCCGGTGCTTCTTCTGCAGGTACCGTCACCACAAGCTTCGCCCCTGCTGAAAGCGGTTTACAACCCGAAGGCCGTCATCCCGCACGCGGCGTTGCTGCATCAGGCTTCCGCCCATTGTGCAATATTCCCCACTGCTGCCTCCCGTAGGAGTCTGGGCCGTATCTCAGTCCCAATGTGGCCGGTCGCCCTCTCAGGCCGGCTACCCGTCAAAGCCTTGGTAAGCCACTACCCCACCAACAAGCTGATAAGCCGCGAGTCCATCCAAAACCGCCG
>n.538.6.bb_67
GTAGTTAGCCGTGGCTTTCTGATTAGGTACCGTCAAGACGTGCATAGTTACTTACACATTTGTTCTTCCCTAATAACAGAGTTTTACGATCCGAAGACCTTCATCACTCACGCGGCGTTGCTCCGTCAGGCTTTCGCCCATTGCGGAAGATTCCCTACTGCTGCCTCCCGTAGGAGTCTGGACCGTGTCTCAGTTCCAGTGTGGCCGATCACCCTCTCAGGGTCGGCTACGCATCGTTGCCTTGGTAAGCCGTTACCTTACCAACTAGCTAATGCGGCGCGGATCCATCTATAAGTGACAGCAAAACCGTCTTTCACTATTGAACCATGCGGTTCAATATATTATCCGGTATTAG
>n.538.6.bb_68
GAAGTTAGCCGGTGCTTCTTCTGCGGGTAACGTCAATCGACAAGGTTATTAACCTTATCGCCTTCCTCCCCGCTGAAAGTACTTTACAACCCGAAGGCCTTCTTCATACACGCGGCATGGCTGCATCAGGCTTGCGCCCATTGTGCAATATTCCCCACTGCTGCCTCCCGTAGGAGTCTGGACCGTGTCTCAGTTCCAGTGTGGCCGATCACCCTCTCAGGTCGGCTACGCATCGCTGCCTTGGTAAGCCGTTACCTTACCAACTAGCTAATGCGGCGCGGATCCATCTATAAGTGACAGCAAAACCGTCTTTTCACTATTGAACCATGCGGTTCAATATATTATCCCGGTATTAGCTCCGGTTTCCCGAAGTTATCCCAGTCTTATAGGTAGGTTATCCCACGTGTTACCTCACCCCGTCCCGCCCGCTAACGTCAGAGGAGCAAGCTCCTCGTCTGTTCGCTCGACTTGCATGTATTAGGGCACGCCGCCAGCGTTCAT
>n.538.6.bb_69
GTAGTTAGCCGTGGCTTTCTGATTAGGTACCGTCAAGACGTGCATAGTTACTTACACATTTGTTCTTCCCTAATAACAGAGTTTTACGATCCGAAGACCTTCATCACTCACGCGGCGTTGCTCCGTCAGGCTTTCGCCCATTGCGGAAGATTCCCTACTGCTGCCTCCCGTAGGAGTCTGGACCGTGTCTCAGTTCCAGTGTGGCCGATCACCCTCTCAGGGTCGGCTACGCATCGTCGCCTTGGTAAGCCGTTACCTTACCAACTAGCTAATGCGGCGCGGATCCATCTATAAGTGACAGCAAAACCGTCTTTCACTATTGAACCATGCGGTTCAATATATTATCCGGTATTAGCTCCGGTTTCCCGAAGTTATCCCAGTCTTATAGGTAGGTTATCCCACGTGTTACTCACCCCGTCCCGCCCGCTAACGTCAGAGGGAGCAAGCTCCCTCGTCTGTTCCGCTCGACTTGCATGTATTAGGGCACGCCGCCAGCGTTCATCC
>n.538.6.bb_70
GTAGTTAGCCGGTGCTTCTTCTGCAGGTACCGTCACCACAAGCTTCGCCCCTGCTGAAAGCGGTTTACAACCCGAAGGCCGTCATCCCGCACGCGGCGTTGCTGCATCAGGCTTCCGCCCATTGTGCAATATTCCCCACTGCTGCCTCCCGTAGGAGTCTGGGCCGTATCTCAGTCCCAATGTGGCCGGTCGCCCTCTCAGGCCGGCTACCCGTCAAAGCCTTGGTAAGCCACTACCCACCAACAAGCTGATAAGCCGCGAGTCCATCCAAAACCGCCG
>n.538.6.bb_71
GTAGTTAGCCGTGGCTTTCTGATTAGGTACCGTCAAGACGTGCATAGTTACTTACACATTTGTTCTTCCCTAATAACAGAGTTTTACGATCCGAAGACCTTCATCACTCACGCGGCGTTGCTCCGTCAGGCTTTCGCCCATTGCGGAAGATTCCCTACTGCTGCCTCCCGTAGGAGTCTGGACCGTGTCTCAGTTCCAGTGTGGCCGATCACCCTCTCAGGGTCGGCTACGCATCGTCGCCTTGGTAAGCCGTTACCTTACCAACTAGCTAATGCGGCGCGGATCCATCTATAAGTGACAGCAAAACCGTCTTTCACTATTGAACCATGCGGTTCAATATATTATCCGGTATTAGCTCCGGT
>n.538.6.bb_72
GGAGTTAGCCGGTGCTTCTTCTGCGGGTAACGTCAATCGACAAGGTTATTAACCTTATCGCCTTCCTCCCCGCTGAAAGTACTTTACAACCCGAAGGCCTTCTTCATACACGCGGCATGGCTGCATCAGGCTTGCGCCCATTGTGCAATATTCCCCACTGCTGCCTCCCGTAGGAGTCTGGACCGTGTCTCAGTTCCAGTGTGGCTGGTCATCCTCTCAGACCAGCTAGGGGATCGTCGCCTAGGTGAGCCGTTACCCACCTACTAGCTAATCCCATCTGGGCACATCTGATGGCAAGAGGCCCGAAGGTCCCCTCTTTGGTCTTGCGACGTTATGCGGTATTAGCTACCGTTTCCAG
>n.538.6.bb_73
GGAGTTAGCCGGTGCTTCTTCTGCGGGTAACGTCAATTGCTGAGGTTATTAACCTCAACACCTTCCTCCCCGCTGAAAGTACTTTACAACCCGAAGGCCTTCTTCATACACGCGGCATGGCTGCATCAGGCTTGCGCCCATTGTGCAATATTCCCCACTGCTGCCTCCCGTAGGAGTCTGGACCGTGTCTCAGTTCCAGTGTGGCTGGGTCATCCTCTCAGACCAGCTAGGGATCGTCGCCTAGGTGAGCCGTTACCCCACCTACTAGCTAATCCCATCTGGGCACATCTGATGGCAA
>n.538.6.bb_74
GTAGTTAGCCGTGGCTTTCTGATTAGGTACCGTCAAGACGTGCATAGTTACTTACACATTTGTTCTTCCCTAATAACAGAGTTTTACGATCCGAAGACCTTCATCACTCACGCGGCGTTGCTCCGTCAGGCTTTCGCCCATTGCGGAAGATTCCCCACTGCTGCCTCCCGTAGGAGTCTGGGCCGTATCTCAGTCCCAATGTGG
>n.538.6.bb_75
GTAGTTAGCCGTGGCTTTCTGATTAGGTACCGTCAAGACGTGCATAGTTACTTACACATTTGTTCTTCCCTAATAACAGAGTTTTACGATCCGAAGACCTTCATCACTCACGCGGCGTTGCTCCGTCAGGCTTTCGCCCATTGCGGAAGATTCCCTACTGCTGCCTCCCGTAGGAGTCTGGACCGTGTCTCAGTTCCAGTGTGGCCGATCACCCTCTCAGGGTCGGCTACGCATCGTTGCCTTGGTAAGCCGTTACCTTACAACTAGCTAATGCGGCGCGGATCCATCTATAAGTGACAGCAAAACCGTCTTTCACTATTGAACCATGCGGTTCAATATATTATCCGGTATTAGCTCCGGT
>n.538.6.bb_76
GTAGTTAGCCGTGGCTTTCTGATTAGGTACCGTCAAGACGTGCATAGTTACTTACACATTTGTTCTTCCCTAATAACAGAGTTTTACGATCCGAAGACCTTCATCACTCACGCGGCGTTGCTCCGTCAGGCTTTCGCCCATTGCGGAAGATTCCCTACTGCTGCCTCCCGTAGGAGTCTGGACCGTGTCTCAGTTCCAGTGTGGCCGATCACCCTCTCAGGGTCGGCTACGCATCGTTGCCTTGGTAAGCCGTTACCTTACAACTAGCTAATGCGGCGCGGATCCATCTATAAGTGACAGCAAAACCGTCTTTCACTATTGAACCATGCGGTTCAATATATTATCCGGTATTAGCTCCGGTC
>n.538.6.bb_77
GTAGTTAGCCGTGGCTTTCTGATTAGGTACCGTCAAGACGTGCATAGTTACTTACACATTTGTTCTTCCCTAATAACAGAGTTTTACGATCCGAAGACCTTCATCACTCACGCGGCGTTGCTCCGTCAGGCTTTCGCCCATTGCGGAAGATTCCCTACTGCTGCCTCCCGTAGGAGTCTGGACCGTGTCTCAGTTCCAGTGTGGCCGATCACCCTCTCAGGGTCGGCTACGCATCGTTGCCTTGGTAAGCCGTTACCTTACCAACTAGCTAATGCGGCGCGGATCCATCTATAAGTGACAGCAAAAACCGTCTTTCACTATTGAACCATGCGGTTCAATATA
>n.538.6.bb_78
GTAGTTAGCCGGTGCTTCTTCTGCAGGTACCGTCACCACAAGCTTCGCCCCTGCTGAAAGCGGTTTACAACCCGAAGGCCGTCATCCCGCACGCGGCGTTGCTGCATCAGGCTTCCGCCCATTGTGCAATATTCCCCACTGCTGCCTCCCGTAGGAGTCTGGGCCGTATCTCAGTCCCAATGTGGCCGGTCGCCCTCTCAGGCCGGCTACCCGGTCAAAGCCTTGGTAAGCCACTACCCCACCAACAAGCTGATAAGCCGCGAGTCCATCCAAAACCGCCGAAGCTTTCCAACCCCCACCATGCAGCAAGGATTCCTATCC
>n.538.6.bb_79
GGAGTTAGCCGGTGCTTCTTCTGCGGGTAACGTCAATTGCTGAGGTTATTAACCTCAACACCTTCCTCCCCGCTGAAAGTACTTTACAACCCGAAGGCCTTCTTCATACACGCGGCATGGCTGCATCAGGCTTGCGCCCATTGTGCAATATTCCCCACTGCTGCCTCCCGTAGGAGTCTGGACCGTGTCTCAGTTCCAGTGTGGCTGGGTCATCCTCTCAGACCAGCTAGGGATCGTCGCCTAGGTGACGCCGTTACCCCACCTACTAGCTAATCCCATCTGGGCACATCTGATGGC
>n.538.6.bb_80
GTAGTTAGCCGTGGCTTTCTGATTAGGTACCGTCAAGACGTGCATAGTTACTTACACATTTATTCTTCCCTAATAACAGAGTTTTACGATCCGAAGACCTTCATCACTCACGCGGCGTTGCTCCGTCAGGCTTTCGCCCATTGCGGAAGATTCCCTACTGCTGCCTCCCGTAGGAGTCTGGACCGTGTCTCAGTTCCAGTGTGGCCGATCACCCTCTCAGGGTCGGCTACGCATCGTTGCCTTGGTAAGCCGTTACCTTACCAACTAGCTAATGCGGCGCGGATCCATCTATAAGTGACAGCAAAACCGTCTTTCACTATTGAACCATGCGGTTCAATATATTATCCGGTATTAGCTCCGGTTTCCCGAAGTTATCCCAGTCTTATAGGTAGGTTATCCCACCGTGTTACTCACCCCGTCCCGCCCGCTAACGTCAGAGGGGGCAAGCTCCCTCGTCTGTTCGCTCGACTTGCATGTATTAGGGCACGC
>n.538.6.bb_81
GTAGTTAGCCGGTGCTTCTTCTGCAGGTACCGTCACCACAAGCTTCGCCCCTGCTGAAAGCGGTTTACAACCCGAAGGCCGTCATCCCGCACGCGGCGTTGCTGCATCAGGCTTCCGCCCATTGTGCAATATTCCCCACTGCTGCCTCTCGTAGGAGTCTGGGCCGTATCTCAGTCCCAATGTGGCCGGTCGCCCTCTCAGGCCGGCTACCCGTCAAAGCCTTGGTAAGCCACTACCCACCAACAAGCTGATAGGCCGCGAGTCCATCCAAAACCGCCGAAGCTTTCCAACCCCCACCATGCAGCAAGGATTCCTATCCGGTATTAGCCCCAGTTTCCTGAAGTTATCCCGAAGTCAAGGGCAGGTTACTCACGTGTTACTCACCCGTTCGCCACTCCGAC
>n.538.6.bb_82
GTAGTTAGCCGTGGCTTTCTGATTAGGTACCGTCAAGACGTGCATAGTTACTTACACATTTGTTCTTCCCTAATAACAGAGTTTTACGATCCGAAGACCTTCATCACTCACGCGGCGTTGCTCCGTCAGGCTTTCGCCCATTGCGGAAGATTCCCTACTGCTGCCTCCCGTAGGAGTCTGGACCGTGTCTCAGTTCCAGTGTGGCCGATCACCCTCTCAGGGTCGGCTACGCATCGTTGCCTTGGTAAGCCGTTACCTTACCAACTAGCTAATGCGGCGCGGATCCATCTATAAGTGACAGCAAAACCGTCTTTCACTATTGAACCATGCGGTTCAATATATTATCCGGTATTAGCTCCGGTTTCCGAAGTTATCCCAGTCTTATAGGTAGGTTATC
>n.538.6.bb_83
GTAGTTAGCCGTGGCTTTCTGATTAGGTACCGTCAAGACGTGCATAGTTACTTACACATTTGTTCTTCCCTAATAACAGAGTTTTACGATCCGAAGACCTTCATCACTCACGCGGCGTTGCTCCGTCAGGCTTTCGCCCATTGCGGAAGATTCCCTACTGCTGCCTCCCGTAGGAGTCTGGACCGTGTCTCAGTTCCAGTGTGGCCGATCACCCTCTCAGGGTCGGCTACGCATCGTTGCCTTGGTAAGCCGTTACCTTACCAACTAGCTAATGCGGCGCGGATCCATCTATAAGTGACAGCAAAACCGTCTTTCACTATTGAACCATGCGGTTCAATATATTATCCGGTATTAGCTCCGGTTTCCCGAAGTTATCCCAGTCTTATAGGTAGGTTATCCCACCGTGTTACTCACCCCGTCCCGCCCGCTAACGTCAGAGGAGCAAGCTCCCTCGTCTGTTCGCTCGACTTGCCATGTATTAGGGCACGCCGCCAG
>n.538.6.bb_84
GTAGTTAGCCGTGGCTTTCTGATTAGGTACCGTCAAGACGTGCATAGTTACTTACACATTTGTTCTTCCCTAATAACAGAGTTTTACGATCCGAAGACCTTCATCACTCACGCGGCGTTGCTCCGTCAGGCTTTCGCCCATTGCGGAAGATTCCCTACTGCTGCCTCCCGTAGGAGTCTGGACCGTGTCTCAGTTCCAGTGTGGCCGATCACCCTCTCAGGGTCGGCTACGCATCGTCGCCTTGGTAAGCCGTTACCTTACCAACTAGCTAATGCGGCGCGGATCCATCTATAAGTGACAGCAAAACCGTCTTTCACTATTGAACCATGCGGTTCAATATATT
>n.538.6.bb_85
TAGTTAGCCGGTGCTTCTTCTGCAGGTACCGTCACCACAAGCTTCGCCCCTGCCGAAAGCGGTTTACAACCCGAAGGCCGTCATCCCGCACGCGGCGTTGCTGCATCAGGCTTCCGCCCATTGTGCAATATTCCCCACTGCTGCCTCCCGTAGGAGTCTGGGCCGTATCTCAGTCCCAATGTGGCCGGTCGCCCTCTCAGGCCGGCTACCCGTCAAAGCCTTGGGTAAGCCCACTACCCCACAACAAGCTGATAAGCCGCGAGTCCATCCAAAACCGCCGAAGCTTTCAACCCCACCATGCAGCAAGGATTCCTATCCGGTATTAGCCCCAGTTTCCTGAGTTATCCCGAAGTCAAGGGCAGGTTACTCACGTGTTACTCA
>n.538.6.bb_86
GTAGTTAGCCGGTGCTTCTTCTGCAGGTACCGTCACCACAAGCTTCGCCCCTGCTGAAAGCGGTTTACAACCCGAAGGCCGTCATCCCGCACGCGGCGTTGCTGCATCAGGCTTCCGCCCATTGTGCAATATTCCCCACTGCTGCCTCCCGTAGGAGTCTGGGCCGTATCTCAGTCCCAATGTGGCCGGTCGCCCTCTCAGGCCGGCTACCCGTCAAAGCCTTGGTAAGCCACTACCCCACCAACAAGCTGATAAGCCGCGAGTCCATCC
>n.538.6.bb_87
GTAGTTAGCCGTGGCTTTCTGATTAGGTACCGTCAAGACGTGCATAGTTACTTACACATTTGTTCTTCCCTAATAACAGAGTTTTACGATCCGAAGACCTTCATCACTCACGCGGCGTTGCTCCGTCAGGCTTTCGCCCATTGCGGAAGATTCCCTACTGCTGCCTCCCGTAGGTGTCTGGACCGTGTCTCAGTTCCAGTGTGGCCGATCACCCTCTCAGGGTCGGCTACGCATCGTTGCCTTGGTAAGCCGTTACCTTACCAACTAGCTAATGCGGCGCGGATCCATCTATAAGTGACAGCAAAACCGTCTTTCACTATTGAACCATGCGGTTCAATATATTATCCGGTATTAGCTCCGGT
>n.538.6.bb_88
GTAGTTAGCCGTGGCTTTCTGATTAGGTACCGTCAAGACGTGCATAGTTACTTACACATTTGTTCTTCCCTAATAACAGAGTTTTACGATCCGAAGACCTTCATCACTCACGCGGCGTTGCTCCGTCAGGCTTTCGCCCATTGCGGAAGATTCCCTACTGCTGCCTCCCGTAGGAGTCTGGACCGTGTCTCAGTTCCAGTGTGGCCGATCACCCTCTCAGGGTCGGCTACGCATCGTTGCCTTGGTAAGCCGTTACCTTACCAACTAGCTAATGCGGCGCGGATCCATCTATAAGTGACAGCAAAACCGTCTTTCACTATTGAACCATGCGGTTCAATATATTATCCGGTATTAGCTCCGGTTTCCCGAAGTTATCCCAGTCTTATAGGTAGGTTATCCCACCGTGTTACTCACCCCGTCCCGCCCACTCGTCACCCCGAGAGCAAGCTCTCTGTGCCTACCGTTCGACTTGCATGTGTTAGGGCCTGCCGCCAGCGTTCAATCTGA
>n.538.6.bb_89
GTAGTTAGCCGTGGCTTTCTGATTAGGTACCGTCAAGACGTGCATAGTTACTTACACATTTGTTCTTCCCTAATAACAGAGTTTTACGATCCGAAGACCTTCATCACTCACGCGGCGTTGCTCCGTCAGGCTTTCGCCCATTGCGGAAGATTCCCTACTGCTGCCTCCCGTAGGAGTCTGGACCGTGTCTCAGTTCCAGTGTGGCCGATCACCCTCTCAGGTCGGCTACGCATCGTTGCCTTGGTAAGCCGTTACCTTACCAACTAGCTAATGCGGCGCGGATCCATCTATAAGTGACAGCAAAACCGTCTTTCACTATTGGACCATGCGGTTCAATATATTATCCGGTATTAGCTCCGGTTTCCCGAAGTTATCCCAGTCTTATAGGTAGG
>n.538.6.bb_90
GGAGTTAGCCGGTGCTTCTTCTGCGGGTAACGTCAATTGCTGAGGTTATTAACCTCAACACCTTCCTCCCCGCTGAAAGTACTTTACAACCCGAAGGCCTTCTTCATACACGCGGCATGGCTGCATCAGGCTTGCGCCCATTGTGCAATATTCCCCACTGCTGCCTCCCGTAGGAGTCTGGACCGTGTCTCAGTTCCAGTGTGGCTGGGTCATCCTCTCAGACCAGCTAGGGATCGTCGCCTAGGTGAGCCGTTACCCCACCTACTAGCTAATCCCATCTGGGCACATCTGATGGCAAGAGGCCCGAAGGTCCCCCACTTTTGGCTTGCGACGTTATGCGGTATTAGCTACCGTTTCCAGTAG
>n.538.6.bb_91
GGAGTTAGCCGGTGCTTCTTCTGCGGGTAACGTCAATCGACAAGGTTATTAACCTTATCGCCTTCCTCCCCGCTGAAAGTACTTTACAACCCGAAGGCCTTCTTCATACACGCGGCATGGCTGCATCAGGCTTGCGCCCATTGTGCAATATTCCCCACTGCTGCCTCCCGTAGGAGTCTGGACCGTGTCTCAGTTCCAGTGTGGCTGGTCATCCTCTCAGACCAGCTAGGGATCGTCGCCTAGGTGAGCCGTTACCCACCTACTAGCTAATCCCATCTGGGCACATCTGATGGCAAGAGG
>n.538.6.bb_92
GTAATTAGCCGGTGCTTCTTCTGCAGGTACCGTCACCACAAGCTTCGCCCCTGCTGAAAGCGGTTTACAACCCGAAGGCCGTCATCCCGCACGCGGCGTTGCTGCATCAGGCTTCCGCCCATTGTGCAATATTCCCCACTGCTGCCTCCCGTAGGAGTCTGGGCCGTATCTCAGTCCCAATGTGGCCGGTCGCCCTCTCAGGCCGGCTACCCGTCAAAGCCTTGGTAAGCCACTACCCCACCAACAAGCTGATAAGCCGCGAGTCCATCCAAAACCGCCGAAGCTTTCCAACCCCACCATGCAGCAAGGATTCCTATCCGGTATTAGCCCCAGTTTCCTGAAGTTATCCCGAAGTCAAGGGCAGGTTACTCACGTGTTACTCACCCGTTCGCCA
>n.538.6.bb_93
GTAGTTAGCCGGTGCTTCTTCTGCAGGTACCGTCACCACAAGCTTCGCCCCTGCTGAAAGCGGTTTACAACCCGAAGGCCGTCATCCCGCACGCGGCGTTGCTGCATCAGGCTTCCCCCATTGTGCAATATTCCCCACTGCTGCCTCCCGTAGGAGTCTGGGCCGTATCTCAGTCCCAATGTGGCCGGTCGCCCTCTCAGGCCGGCTACCCGGTCAAAGCCTTGGTAAGCCACTACCCACCAACAAGCTGATAAGCCGCGAGTCCATCCAAAACCGCCGAAGCTTTCCAACCCCACCATGCAGCAAGGATTCCTATCCGGTATTAGCCCCAGTTTCCTGAAGTTATCCCGAAGTCAAGGGCAGGTTACTCACGTGTACTCACCCGTTCG
>n.538.6.bb_94
GTAGTTAGCCGGTGCTTCTTCTGCAGGTACCGTCACCACAAGCTTCGCCCCTGCTGAAAGCGGTTTACAACCCGAAGGCCGTCATCCCGCACGCGGCGTTGCTGCATCAGGCTTCCGCCCATTGTGCAATATTCCCCACTGCTGCCTCCCGTAGGAGTCTGGACCGTGTCTCAGTTCCAGTGTGGCCGATCACCCTNTCAGGTCGGCTACGCATCGTTGCCTTGGTAACGCCGTTACCTTACCAACTAGCTAATGCGGCGCGGGATCCATCTATAAAGTGACAGCAAAACCGTCTTTCACTATTGAACC
>n.538.6.bb_95
GGAGTTAGCCGGTGCTTCTTCTGCGGGTAACGTCAATCGACAAGGTTATTAACCTTATCGCCTTCCTCCCCGCTGAAAGTACTTTACAACCCGAAGGCCTTCTTCATACACGCGGCATGGCTGCATCAGGCTTGCGCCCATTGTGCAATATTCCCCACTGCTGCCTCCCGTAGGAGTCTGGACCGTGTCTCAGTTCCAGTGTGGCTGGTCATCCTCTCAGACCAGCTAGGGATCGTCGCCTAGGTGAGCCGTTACCCACCTACTAGCTAATCCCATCTGGGCACATCTGATGGCAAG
>n.538.6.bb_96
GTAGTTAGCCGGTGCTTCTTCTGCAGGTACCGTCACCACAAGCTTCGCCCCTGCTGAAAGCGGTTTACAACCCGAAGGCCGTCATCCCGCACGCGGCGTTGCTGCATCAGGCTTCCGCCCATTGTGCAATATTCCCCACTGCTGCCTCCCGTAGGAGTCTGGGCCGTATCTCAGTCCCAATGTGGCCGGTCGCCCTCTCAGGCCGGCTACCCGGTCAAAGCCTTGGTAAGCCACTACCCCACCAACAAGCTGATAAGCCGCGAGTCCATCCAAAAC
>n.538.6.bb_97
GGAGTTAGCCGGTGCTTCTTCTGCGGGTAACGTCAATCGACAAGGTTATTAACCTTATCGCCTTCCTCCCCGCTGAAAGTACTTTACAACCCGAAGGCCTTCTTCATACACGCGGCATGGCTGCATCAGGCTTGCGCCCATTGTGCAATATTCCCCACTGCTGCCTCCGTAGGAGTCTGGACCGTGTCTCAGTTCCAGTGTGGCTGGTCATCCTCTCAGACCAGCTAGGGATCGTCGCCTAGGTGAGCCGTTACCCACCTACTAGCTAATCCCATCTGGGCACATCTGATGGCAAGAGGCCCGAAGGTCCCCCTCTTTGGTCTTGCGACGTTATGCGGTATTAGCTACCGTTTCCAG
>n.538.6.bb_98
GTAGTTAGCCGTGGCTTTCTGATTAGGTACCGTCAAGACGTGCATAGTTACTTACACATTTGTTCTTCCCTAATAACAGAGTTTTACGATCCGAAGACCTTCATCACTCACGCGGCGTTGCTCCGTCAGGCTTTCGCCCATTGCGGAAGATTCCCTACTGCTGCCTCCCGTAGGAGTCTGGACCGTGTCTCAGTTCCAGTGTGGCCGATCACCTTCTCAGGGTCGGCTACGCATCGTCGCCTTGGTAAGCCGTTACCTTACCAACTAGCTAATGCGGCGCGGATCCATCTATAAGTGAC
>n.538.6.bb_99
GTAGTTAGCCGTGGCTTTCTGATTAGGTACCGTCAAGACGTGCATAGTTACTTACACATTTGTTCTTCCCTAATAACAGAGTTTTACGATCCGAAGACCTTCATCACTCACGCGGCGTTGCTCCGTCAGGCTTTCGCCCATTGCGGAAGATTCCCTACTGCTGCCTCCCGTAGGAGTCTGGACCGTGTCTCAGTTCCAGTGTGGCCGATCACCCTCTCAGGGTCGGCTACGCATCGTCGCCTTGGTAAGCCGTTACCTTGCCAACTAGCTAATGCGGCGTGGATCCATCTATAAGTGACAGCAAAACCGTCTTTCACTATTGAACCATGCGGTTCAATATATTATCCGGTATTAGCTCCGGT
>n.538.6.bb_100
GTAGTTAGCCGGTGCTTCTTCTGCAGGTACCGTCACCACAAGCTTCGCCCCTGCTGAAAGCGGTTTACAACCCGAAGGCCGTCATCCCGCACGCGGCGTTGCTGCATCAGGCTTCCGCCCATTGTGCAATATTCCCCACTGCTGCCTCCCGTAGGAGTCTGGGCCGTATCTCAGTCCCAATGTGGCCGGTCGCCCTCTCAGGCCGGCTACCCGTCAAAGCCTTGGTAAGCCACTACCCACCAACAAGCTGATAAGCCGCGAGTCCATCC
>n.538.6.bb_101
GTAGTTAGCCGGTGCTTCTTCTGCAGGTACCGTCACCACAAGCTTCGCCCCTGCTGAAAGCGGTTTACAACCCGAAGGCCGTCATCCCGCACGCGGCGTTGCTGCATCAGGCTTCCGCCCATTGTGCAATATTCCCCACTGCTGCCTCCCGTAGGAGTCTGGGCCGTATCTCAGTCCCAATGTGGCCGGTCGCCCTCTCAGGCCGGCTACCCGTCAAAGCCTTGGTAAGCCACTACCCACCAACAAGCTGATAAGCCGCGAGTCCATCCAAAACCGCCG
>n.538.6.bb_102
GTAGTTAGCCGTGGCTTTCTGATTAGGTACCGTCAAGACGTGCATAGTTACTTACACATTTGTTCTTCCCTAATAACAGAGTTTTACGATCCGAAGACCTTCATCACTCACGCGGCGTTGCTCCGTCAGGCTTTCGCCCATTGCGGAAGATTCCCTACTGCTGCCTCCCGTAGGAGTCTGGACCGTGTCTCAGTTCCAGTGTGGCCGATCACCCTCTCAGGGTCGGCTACGCATCGTTGCCTTGGTAAGCCGTTACCTTACCAACTAGCTAATGCGGACGCGGATCCATCTATAAGTGACAGCAAAACCGTCTTTCACTATTGAACCATGCGGTTCAATATATTATCCGGTATTAG
>n.538.6.bb_103
GTAGTTAGCCGGTGCTTCTTCTGCAGGTACCGTCACCACAAGCTTCGCCCCTGCTGAAAGCGGTTTACAACCCGAAGGCCGTCATCCCGCACGCGGCGTTGCTGCATCAGGCTTCCGCCCATTGTGCAATATTCCCCACTGCTGCCTCCCGTAGGAGTCTGGGCCGTATCTCAGTCCCAATGTGGCCGGTCGCCCTCTCAGGCCGGCTGCCCGTCAAAGCCCTTGGTAGGCCACTACCCACCAACAAGCTGATAAGCCGCGAGTCCATCCAAAACCGCCGAAGCTTTCCAACCCCCACCATGCAGCAAGGATTCCTATCCGGTATTAGCCCCAGTTTCCTGAAGTTATCCCGAAGTCAAGGGCAGGTTACTCACGT
>n.538.6.bb_104
GTAGTTAGCCGTGGCTTTCTGATTAGGTACCGTCAAGACGTGCATAGTTACTTACACATTTGTTCTTCCCTAATAACAGAGTTTTTACGATCCGAAAGACCTTTCATCACTCACGCGGCGTTGCTCCGTCAGGCTTTCGCCCATTGCGGAAGATTCCCTACTGCTGCCTCCCGTAGGAGTCTGGACCGTGTCTCAGTTCCAGTGTGGCCGATCACCCTNTCAGGGTCGGCTACGCATCGTTGCCTTGTAAGCCGTTACCTTACAACTAGCTAATGCGGCGCGGATCCAT
>n.538.6.bb_105
GTAGTTAGCCGGTGCTTCTTCTGCAGGTACCGTCACCACAAGCTTCGCCCCTGCTGAAAGCGGTTTACAACCCGAAGGCCGTCATCCCGCACGCGGCGTTGCTGCATCAGGCTTCCGCCCATTGTGCAATATTCCCCACTGCTGCCTCCCGTAGGAGTCTGGGCCGTATCTCAGTCCCAATGTGGCCGGTCGCCCTCTCAGGCCGGCTACCCGTCAAAGCCTTGGTAAGCCACTACCCCACCAACAAGCTGATAAGCCGCGAGTCCATCCAAAACCGCCGAAGCTTTTCCAACCCCCACCATGCAGCAAGGATTCCTATCCGGTAT
>n.538.6.bb_106
GTAGTTAGCCGTGGCTTTCTGATTAGGTACCGTCAAGACGTGCATAGTTACTTACACATTTGTTCTTCCCTAATAACAGAGTTTTACGATCCGAAGACCTTCATCACTCACGCGGCGTTGCTCCGTCAGGCTTTCGCCCATTGCGGAAGATTCCCTACTGCTGCCTCCCGTAGGAGTCTGGACCGTGTCTCAGTTCCAGTGTGGCCGATCACCCTCTCAGGGTCGGCTACGCATCGTTGCCTTGGTAAGCCGTTACCTTACCAACTAGCTAATGCGGCGCGGATCCATCTATAAGTGACAGCAAAACCGTCTTTCACTATTGAACCATGCGGTTCAATATATTATCCGGTATTAGCTCCGGTTTCCCGAAGTTATCCCAGTCTTATAGGTAGGTTATC
>n.538.6.bb_107
GTAGTTAGCCGTGGCTTTCTGATTAGGTACCGTCAAGACGTGCATAGTTACTTACACATTTATTCTTCCCTAATAACAGAGTTTTACGATCCGAAGACCTTCATCACTCACGCGGCGTTGCTCCGTCAGGCTTTCGCCCATTGCGGAAGATTCCCTACTGCTGCCTCCCGTAGGAGTCTGGACCGTGTCTCAGTTCCAGTGTGGCCGATCACCCTCTCAGGGTCGGCTACGCATCGTTGCCTTGGTAAGCCGTTACCTTACCAACTAGCTAATGCGGCGCGGATCCATCTATAAGTGACAGCAAAACCGTCTTTCACTATTGAACCATGCGGTTCAATATATTATCCGGTATTAGCTCC
>n.538.6.bb_108
GTAGTTAGCCGGTGCTTCTTCTGCAGGTACCGTCACCACAAGCTTCGCCCCTGCTGAAAGCGGTTTACAACCCGAAGGCCGTCATCCCGCACGCGGCGTTGCTGCATCAGGCTTCCGCCCATTGTGCAATATTCCCCACTGCTGCCTCCCGTAGGAGTCTGGGCCGTATCTCAGTCCCAATGTGGCCGGTCGCCCTCTCAGGCCGGCTACCCGGTCAAAGGCCTTGGTAAGCCACTACCCCACCAACAAGCTGATAAGCCGCGAGTCCATCC
>n.538.6.bb_109
GTAGTTAGCCGGTGCTTCTTCTGCAGGTACCGTCACCACAAGCTTCGCCCCTGCTGAAAGCGGTTTACAACCCGAAGGCCGTCATCCCGCACGCGGCGTTGCTGCATCTAGGCTTCCGCCCATTGTGCAATATTCCCCACTGCTGCCTCCCGTAGGAGTCTGGGCCGTATCTCAGTCCCAATGTGGCCGGTCGCCCTCTCAGGCCGGCTACCCGTNAAAGCCTTGGTAAGCCACTACCCCACCAACAAGCTGATAAGCCGCGAGTCCATC
>n.538.6.bb_110
GGAGTTAGCCGGTGCTTCTTCTGCGGGTAACGTCAATCGATGAGGTTATTAACCTCACCGCCTTCCTCCCCGCTGAAAGTGCTTTACAACCCGAAGGCCTTCTTCACACACGCGGCATGGCTGCATCAGGCTTGCGCCCATTGTGCAATATTCCCCACTGCTGCCTCCCGTAGGAGTCTGGACCGTGTCTCAGTTCCAGTGTGGCTGGGTCATCCTCTCAGACCAGCTAGGGATCGTCGCCTAGGTGAGCCGTTACCCACCTACCAGCTAATCCCATCTGGGCACATCTGATGGCATGAGGCCCGAAGGTCCCCACTTTGGTCTTGCGACATTATGCGGTATTAGCTACCGTTTCCAGTAGT
>n.538.6.bb_111
GTAGTTAGCCGTGGCTTTCTGATTAGGTACCGTCAAGACGTGCATAGTTACTTACACATTTGTTCTTCCCTAATAACAGAGTTTTACGATCCGAAGACCTTCATCACTCACGCGGCGTTGCTCCGTCAGGCTTTCGCCCATTGCGGAAGATTCCCTACTGCTGCCTCCCGTAGGAGTCTGGACCGTGTCTCAGTTCCAGTGTGGCCGATCACCCTCTCAGGGTCGGCTACGCATCGTCGCCTTGGTAAGCCGTTACCTTACCAACTAGCTAATGCGGCGCGGATCCATCTATAAGTGACAGCAAAACCGTCTTTCACTATTGAACCATGCGGTTCAATATATTATCCGGTATTAACTCCGGTTTCCCCGAAGTTATCCCAGTCTTATAGGTA
>n.538.6.bb_112
GTAGTTAGCCGGTGCTTCTTCTGCAGGTACCGTCACCACAAGCTTCGCCCCTGCTGAAAGCGGTTTACAACCCGAAGGCCGTCATCCCGCACGCGGCGTTGCTGCATCAGGCTTCCGCCCATTGTGCAATATTCCCCACTGCTGCCTCCCGTAGGAGTCTGGGCCGTATCTCAGTCCCAATGTGGCCGGTCGCCCTCTCAGGGCCGGCTACCCGTCAAAGCCTTGGTAAGCCACTACCCACCAACAAGCTGATAAGCCGCGAGTCCATCCAAAACCGCCGAAGCTTTCCAACCCCCACCATGCAGCAAGGATTCCTATCCGGTATTAGCCCCAGTTTCCTGAAGTTATCCCGAAGTCAAGGGCAGGTTACTCACGTGTTACCTCACCCGTTCGCCA
>n.538.6.bb_113
GTAGTTAGCCGGTGCTTCTTCTGCAGGTACCGTCACCACAAGCTTCGCCCCTGCTGAAAGCGGTTTACAACCCGAAGGCCGTCATCCCGCACGCGGCGTTGCTGCATCAGGCTTCCGCCCATTGTGCAATATTCCCCACTGCTGCCTCCCGTAGGAGTCTGGGCCGTATCTCAGTCCCAATGTGGCCGTCGCCCTCTCAGGCCGGCTACCCGGTCAAAGGCCCTTTGGTAAGCCACTACCCCACCAACAAGCTGATAAGCCGCGAGTCCATCC
>n.538.6.bb_114
TAGTTAGCCGGTGCTTCTTCTGCAGGTACCGTCACCACAAGCTTCGCCCCTGCTGAAAGCGGTTTACAACCCGAAGGCCGTCATCCCGCACGCGGCGTTGCTGCATCAGGCTTCCGCCCATTGTGCAATATTCCCCACTGCTGCCTCCCGTAGGAGTCTGGGCCGTATCTCAGTCCCAATGTGGCCGTCGCCCTCTCAGGCCGGCTACCCGTCAAAGCCTTGGTAAGCCACTACCCCACCAACAAGCTGATAAGCCGCGAGTCCATCC
>n.538.6.bb_115
GTAGTTAGCCGGTGCTTCTTCTGCAGGTACCGTCACCACAAGCTTCGCCCCTGCTGAAAGCGGTTTACAACCCGAAGGCCGTCATCCCGCACGCGGCGTTGCTGCATCAGGCTCCCGCCCATTGTGCAATATTCCCCACTGCTGCCTCCCGTAGGAGTCTGGGCCGTATCTCAGTCCCAATGTGGCCGGTCGCCCTCTCAGGCCGGCTACCCGTCAAAGCCTTGGTAAGCCACTACCCCACCAACAAGCTGATAAGCCGCGAGTCCAT
>n.538.6.bb_116
GTAGTTAGCCGGTGCTTCTTCTGCAGGTACCGTCACCACAAGCTTCGCCCCTGCTGAAAGCGGTTTACAACCCGAAGGCCGTCATCCCGCACGCGGCGTTGCTGCATCAGGCTTCCGCCCATTGTGCAATATTCCCCACTGCTGCCTCCCGTAGGAGTCTGGGCCGTATCTCAGTCCCAATGTGGCCGGTCGCCCTCTCAGGCCGGCTACCCGTCAAAGCCTTGGTAAGCCACTACCCACCAACAAGCTGATAAGCCGCGAGTCCATCCAAAACCGCCGAAGCTTTCCAACCCCACCATGCAGCAAGGATTCCTATCCGGTATTAGCCCCAGTTTCCTGAAGTTATCCCGAAGT
>n.538.6.bb_117
GTAGTTAGCCGTGGCTTTCTGATTAGGTACCGTCAAGACGTGCATAGTTACTTACACATTTGTTCTTCCCTAATAACAGAGTTTTACGATCCGAAGACCTTCATCACTCACGCGGCGTTGCTCCGTCAGGCTTTCGCCCATTGCGGAAGATTCCCTACTGCTGCCTCCCGTAGGAGTCTGGACCGTGTCTCAGTTCCAGTGTGGCCGATCACCCTCTCAGGGTCGGCCACGCATCGTTGCCTTGGTAAGCCGTTACCTTACCAACTAGCTAATGCGGCGCGGATCCATCTATAAGTGACAGCAAAACCGTCTTTCACTATTGAACCATGCGGTTCAATATATTATCCGGTATTAGCTCCGGTTTCCCGAAGTTATCCCAGTCTTATAGGTAGGTTATCCCACCGTGTTACTCACCCCGTCCCGCCCGCTAACGTCAGAGGGAGCAAGCCTCCCTCGTCTGTTCCGCTCGACTTGCATGTATTAGGGCACGCCGCCAGCGTTCATCCT
>n.538.6.bb_118
GTAGTTAGCCGTGGCTTTCTGATTAGGTACCGTCAAGACGTGCATAGTTACTTACACATTTGTTCTTCCCTAATAACAGAGTTTTACGATCCGAAGACCTTCATCACTCACGCGGCGTTGCTCGGTCAGACTTTCGTCCATTGCCGAAGATTCCCTACTGCTGCCTCCCGTAGGAGTCTGGGCCGTGTCTCAGTCCCAGTGTGGCCGATCACCCTCTCAGGGTCGGCTATGTATCGTCGCCTTGGTGGGCCGTTACCCACCAACTAGCTAATACAACGCAGGTCCATCTACTAGTGATGCAATTGCATCTTTCAAGCATCTAACATGTGTTAAACACTGTTATGCGGTATTAGCTATCGTTTCCAATAGTTATCCCCGCTAGTAGG
>n.538.6.bb_119
GTAGTTAGCCGTGGCTTTCTGATTAGGTACCGTCAAGACGTGCATAGTTACTTACACATTTGTTCTTCCCTAATAACAGAGTTTTACGATCCGAAGACCTTCATCACTCACGCGGCGTTGCTCCGTCAGGCTTTCGCCCATTGCGGAAGATTCCCTACTGCTGCCTCCCGTAGGAGTCTGGACCGTGTCTCAGTTCCAGTGTGGCCGATCACCCTCTCAGGGTCGGCTACGCATCGTTGCCTTGGTAAGCCGTTACCTTACCAACTAGCTAATGCGGCGCGGATCCATCTATAAGTGACAGCAAAACCGTCTTTCACTATTGAACCATGCGGTTCAATATATTATCCGGTATTAGCTCCGG
>n.538.6.bb_120
GTAGTTAGCCGGTGCTTCTTCTGCAGGTACCGTCACCACAAGCTTCGCCCCTGCTGAAAGCGGTTTACAACCCGAAGGCCGTCATCCCGCACGCGGCGTTGCTGCATCAGGCTTCCGCCCATTGTGCAATATTCCCCACTGCTGCCTCCCGTAGGAGTCTGGGCCGTATCTCAGTCCCAATGTGGCCGGTCGCCCTCTCAGGCCGGCTACCCGGTCAAAGCCTTGGTAAGCCACTACCCCACCAACAAGCTGATAAGCCGCGAGTCCATCCAAAACCGCC
>n.538.6.bb_121
GTAGTTAGCCGGTGCTTCTTCTGCAGGTACCGTCACCACAAGCTTCGCCCCTGCTGAAAGCGGTTTACAACCCGAAGGCCGTCATCCCGCACGCGGCGTTGCTGCATCAGGCTTCCGCCCATTGTGCAATATTCCCCACTGCTGCCTCCCGTAGGAGTCTGGGCCGTATCTCAGTCCCAATGTGGCCGGTCGCCCTCTCAGGCCGGCTACCCGTCAAAGCCTTGGTAAGCCACTACCCCACCAACAAGCTGATAAGCCGCGAGTCCATCCAAAACCGCCGAAGCTTTCCAACCCCACCATGCAGCAAGGATTCCTATCCGGTATTAGCCCCAGTTTCCTGAAGTTATCCCGAAGTCAAGGGCAGGGTTACTCACGTGTTACTCACCCGTTCGCCA
>n.538.6.bb_122
GTAGTTAGCCGGTGCTTCTTCTGCAGGTACCGTCACCACAAGCTTCGCCCCTGCTGAAAGCGGTTTACAACCCGAAGGCCGTCATCCCGCACGCGGCGTTGCTGCATCAGGCTTCCGCCCATTGTGCAATATTCCCCACTGCTGCCTCCCGTAGGAGTCTGGGCCGTATCTCAGTCCCAATGTGGCCGGTCGCCCTCTCAGGCCGGCTACCCGTCAAAGCCTTGGTAAGCCACTACCCCACCAACAAGCTGATAAGCCGCGAGTCCATCCAAAACCGCCGAAGCTTTCCAACCCCACCATGCAGCAAGGATTCCTATCCGGTATTAGCCCCAGTTTCCTGAAGTTA
>n.538.6.bb_123
GTAGTTAGCCGTGGCTTTCTGATTAGGTACCGTCAAGACGTGCATAGTTACTTACACATTTATTCTTCCCTAATAACAGAGTTTTACGATCCGAAGACCTTCATCACTCACGCGGCGTTGCTCCGTCAGGCTTTCGCCCATTGCGGAAGATTCCCTACTGCTGCCTCCCGTAGGAGTCTGGACCGTGTCTCAGTTCCAGTGTGGCCGATCACCCTCTCAGGGTCGGCTACGCATCGTTGCCTTGGTAAGCCGTTACCTTACCAACTAGCTAATGCGGCGCGGATCCATCTATAAGTGACAGCAAAACCGTCTTTCACTATTGAACCATGCGGTTCAATATATTATCCGGTATTAGCTCCG
>n.538.6.bb_124
GTAGTTAGCCGTGGCTTTCTGATTAGGTACCGTCAAGACGTGCATAGTTACTTACACATTTGTTCTTCCCTAATAACAGAGTTTTACGATCCGAAGACCTTCATCACTCACGCGGCGTTGCTCCGTCAGGCTTTCGCCCATTGCGGAAGATTCCCTACTGCTGCCTCCCGTAGGAGTCTGGACCGTGTCTCAGTTCCAGTGTGGCCGATCACCCTCTCAGGGTCGGCTACGCATCGTTGCCTTGGTAAGCCGTTACCTTACCAACTAGCTAATGCGGCGCGGATCCATCTATAAGTGACAGCAAAACCGTCTTTCACTATTGAACCATGCGGTTCAATATATTATCCGGTATTAGCTCCCGGT
>n.538.6.bb_125
CGTAGTTAGCCGTGGCTTTCTGATTAGGTACCGTCAAGACGTGCATAGTTACTTACACATTTATTCTTCCCTAATAACAGAGTTTTACGATCCGAAGACCTTCATCACTCACGCGGCGTTGCTCCGTCAGGCTTTCGCCCATTGCGGAAGATTCCCTACTGCTGCCTCCCGTAGGAGTCTGGACCGTGTCTCAGTTCCAGTGTGGCCGATCACCCTCTCAGGGTCGGCTACGCATCGTTGCCTTGGTAAGCCGTTACCTTACCAACTAGCTAATGCGGCGCGGATCCATCTATAAGTGACAGC
>n.538.6.bb_126
GGAGTTAGCCGGTGCTTCTTCTGCGGGTAACGTCAATTGCTGAGGTTATTAACCTCAACACCTTCCTCCCCGCTGAAAGTACTTTACAACCCGAAGGCCTTCTTCATACACGCGGCATGGCTGCATCAGGCTTGCGCCCATTGTGCAATATTCCCCACTGCTGCCTCCCGTAGGAGTCTGGACCGTGTCTCAGTTCCAGTGTGGCTGGGTCATCCTCTCAGACCAGCTAGGGGATCGTCGCCTAGGTGAGCCGTTACCCCACCTACTAGCTAATCCCATCTGGGCACATCTGATGGCAAGAGGCCCGAAGGTCCCCCTCTTTGGTCTTGCGACGTTATGCGGTATTAGCTACCGTTTCCAGTAGTT
>n.538.6.bb_127
GTAGTTAGCCGTGGCTTTCTGATTAGGTACCGTCAAGACGTGCATAGTTACTTACACATTTATTCTTCCCTAATAACAGAGTTTTACGATCCGAAGACCTTCATCACTCACGCGGCGTTGCTCCGTCAGGCTTTCGCCCATTGCGGAAGATTCCCTACTGCTGCCTCCCGTAGGAGTCTGGACCGTGTCTCAGTTCCAGTGTGGCCGATCACCCTCTCAGGGTCGGCTACGCATCGTTGCCTTGGTAAGCCGTTACCTTACCAACTAGCTAATGCGGCGCGGATCCATCTATAAGTGACAGCAAAACCGTCTTTCACTATTGAACCATGCGGTTCAATATATTATCCGGTATTAGCTCCGGT
>n.538.6.bb_128
GTAGTTAGCCGTGGCTTTCTGATTAGGTACCGTCAAGACGTGCATAGTTACTTACACATTTGTTCTTCCCTAATAACAGAGTTTTACGATCCGAAGACCTTCATCACTCACGCGGCGTTGCTCCGTCAGGCTTTCGCCCATTGCGGAAGATTCCCTACTGCTGCCTCCCGTAGGAGTCTGGACCGTGTCTCAGTTCCAGTGTGGCCGATCACCCTCTCAGGGTCGGCTACGCATCGTTGCCTTGGTAAGCCGTTACCTTACCAACTAGCTAATGCGGCGCGGATCCATCTATAAGTGACAGCAAAACCGTCTTTCACTATTGAACCATGCGGTTCAATATATTAT
>n.538.6.bb_129
GTAGTTAGCCGGTGCTTCTTCCGCAGGTACCGTCACCACAAGCTTCGCCCCTGCTGAAAGCGGTTTACAACCCGAAGGCCGTCATCCCGCACGCGGCGTTGCTGCATCAGGCTTCCGCCCATTGTGCAATATTCCCCACTGCTGCCTCCCGTAGGAGTCTGGGCCGTATCTCAGTCCCAATGTGGCCGGTCGCCCTCTCAGGCCGGCTACCCGGTCAAAGCCTTGGTAAGCCACTACCCACCAACAAGCTGATAAGCCGCGAGTCCATCCAAAACCGCCGAAGCTTTCCAACCCCACCATGCAGCAAGGATTCCTATCCGGTATTAGCCCCAGTTTCCTGAAGTTATCCCG
>n.538.6.bb_130
GTAGTTAGCCGTGGCTTTCTGATTAGGTACCGTCAAGACGTGCATAGTTACTTACACATTTGTTCTTCCCTAATAACAGAGTTTTACGATCCGAAGACCTTCATCACTCACGCGGCGTTGCTCCGTCAGGCTTTCGCCCATTGCGGAAGATTCCCTACTGCTGCCTCCCGTAGGAGTCTGGACCGTGTCTCAGTTCCAGTGTGGCCGATCACCCTCTCAGGGTCGGCTACGCATCGTTGCCTTGGTAAGCCGTTACCTTACCAACTAGCTAATGCGGCGCGGATCCATCTATAAGTGACAGCAAAACCGTCTTTCACTATTGAACCATGCGG
>n.538.6.bb_131
GTAGTTAGCCGTGGCTTTCTGATTAGGTACCGTCAAGACGTGCATAGTTACTTACACATTTGTTCTTCCCTAATAACAGAGTTTTACGATCCGAAGACCTTCATCACTCACGCGGCGTTGCTCCGTCAGGCTTTCGCCCATTGCGGAAGATTCCCTACTGCTGCCTCCCGTAGGAGTCTGGACCGTGTCTCAGTTCCAGTGTGGCCGATCACCCTCTCAGGGTCGGCTACGCATCGTTGCCTTGGTAAGCCGTTACCTTACCAACTAGCTAATGCGGCGCGGATCCATCTATAAGTGACAGCAAAACCGTCTTTCACTATTGAACCATGCGGTTCAATATATTATCCGGTATTA
>n.538.6.bb_132
GTAGTTAGCCGTGGCTTTCTGATTAGGTACCGTCAAGACGTGCATAGTTACTTACACATTTGTTCTTCCCTAATAACAGAGTTTTACGATCCGAAGACCTTCATCACTCACGCGGCGTTGCTCCGTCAGGCTTTCGCCCATTGCGGAAGATTCCCTACTGCTGCCTCCCGTAGGAGTCTGGACCGTGTCTCAGTTCCAGTGTGGCCGATCACCCTCTCAGGGTCGGCTACGCATCGTTGCCTTGGTAAGCCGTTACCTTACCAACTAGCTAATGCGGCGCGGATCCATCTATAAGTGACAGCAAAACCGTCTTTCACTACTGAACCATGCGGTTCAATATATTATCCGGTATTA
>n.538.6.bb_133
GGAGTTAGCCGGTGCTTCTTCTGCGGGTAACGTCAATCGACAGGGTTATTAACCCTGTCGCCTTCCTCCCCGCTGAAAGTACTTTACAACCCGAAGGCCTTCTTCATACACGCGGCATGGCTGCATCAGGCTTGCGCCCATTGTGCAATATTCCCCACTGCTGCCTCCCGTAGGAGTCTGGACCGTGTCTCAGTTCCAGTGTGGCTGGGTCATCCTCTCAGACCAGCTAGGGATCGTCGCCTAGGTGAGCCGTTACCCACCTACTAGCTAATCCCAT
>n.538.6.bb_134
GTAGTTAGCCGGTGCTTCTTCTGCAGGTACCGTCACCACAAGCTTCGCCCCTGCTGAAAGCGGTTTACAACCCGAAGGCCGTCATCCCGCACGCGGCGTTGCTGCATCAGGCTTCCGCCCATTGTGCAATATTCCCCACTGCTGCCTCCCGTAGGAGTCTGGGCCGTATCTCAGTCCCAATGTGGCCGGTCGCCCTCTCAGGCCGGCTACCCGTCAAAGCCTTGGTAAGCCACTACCCCACCAACGAGCTGATAAGCCGCGAGTCCGTCCAAAACCGCCGAAGCTTTCCAACCCCACCATGCAGCAAGGATTCCTATCCGGTATTAGCCCCAGTTTCCTGAAGTTATCCCGAGGTCAAGGGCAGGTTACTCACGTGTACTCACCCGTT
>n.538.6.bb_135
GTAGTTAGCCGTGGCTTTCTGATTAGGTACCGTCAAGACGTGCATAGTTACTTACACATTTGTTCTTCCCTAATAACAGAGTTTTACGATCCGAAGACCTTCATCACTCACGCGGCGTTGCTCCGTCAGGCTTTCGCCCATTGCGGAAGATTCCCTACTGCTGCCTCCCGTAGGAGTCTGGACCGTGTCTCAGTTCCAGTGTGGCCGATCACCCTCTCAGGGTCGGCTACGCATCGTTGCCTTGGTAAGCCGTTACCTTACCAACTAGCTAATGCGGCGCGGATCCATCTATAAGTGACAGCAAAACCGTCTTTCACTATTGAACCATGCGGTTCAATATATTATCCGGTATTAGCTCCGGTTTCCCGAAGTTATCCCAGTCTTATAGGTAGGTTATC
>n.538.6.bb_136
GTAGTTAGCCGGTGCTTCTTCTGCAGGTACCGTCACCACAAGCTTCGCCCCTGCTGAAAGCGGTTTACAACCCGAAGGCCGTCATCCCGCACGCGGCGTTGCTGCATCAGGCTTCCGCCCATTGTGCAATATTCCCCACTGCTGCCTCCCGTAGGAGTCTGGGCCGTATCTCAGTCCCAATGTGGCCGGTCGCCCTCTCAGGCCGGCTACCCGTTAAAGCCTTGGTAAGCCACTACCCACCAACAAGCTGATAAGCCGCGAGTCCATCCAAAACCGCCGAAGCTTTTCCAACCCCACCATGCAGCAAGGATTCCTATCCGGTATTAGCCCCAGTTTCC
>n.538.6.bb_137
GTAGTTAGCCGGTGCTTCTTCTGCAGGTACCGTCACCACAAGCTTCGCCCCTGCTGAAAGCGGTTTACAACCCGAAGGCCGTCATCCCGCACGCGGCGTTGCTGCATCAGGCTTCCGCCCATTGTGCAATATTCCCCACTGCTGCCTCCCGTAGGAGTCTGGGCCGTATCTCAGTCCCAATGTGGCCGGTCGCCCTCTCAGGCCGGCTACCCGTCAAAGCCTTGGTAAGCCACTACCCCACCAACAAGCTGATAAGCCGCGAGTCCATCC
>n.538.6.bb_138
GTAGTTAGCCGTGGCTTTCTGATTAGGTACCGTCAAGACGTGCATAGTTACTTACACATTTATTCTTCCCTAATAACAGAGTTTTACGATCCGAAGACCTTCATCACTCACGCGGCGTTGCTCCGTCAGGCTTTCGCCCATTGCGGAAGATTCCCTACTGCTGCCTCCCGTAGGAGTCTGGACCGTGTCTCAGTTCCAGTGTGGCCGATCACCCTCTCAGGGTCGGCTACGCATCGTTGCCTTGGTAAGCCGTTACCTTACCAACTAGCTAATGCGGCGCGGATCCATCTATAAGTGACAGCAAAAACCGTCTTTCACTATTGAACCATGCGGTTCAATATATTATCCGGTATTAGCTCCGGT
>n.538.6.bb_139
GTAGTTAGCCGTGGCTTTCTGATTAGGTACCGTCAAGACGTGCATAGTTACTTACACATTTGTTCTTCCCTAATAACAGAGTTTTACGATCCGAAGACCTTCATCACTCACGTGGCGTTGCTCCGTCAGGCTTTCGCCCATTGCGGAAGATTCCCTACTGCTGCCTCCCGTAGGAGTCTGGACCGTGTCTCAGTTCCAGTGTGGCCGATCACCTTCTCAGGGTCGGCTACGCATCGTTGCCTTGGTAAGCCGTTACCTTACCAACTAGCTAATGCGGCGCGGATCCATCTATAAGTGACAGCAAAACCGTCTTTCACTATTGAACCATGCGGTTCAATATATTATCCGGTATTAGCTCCGGT
>n.538.6.bb_140
GTAGTTAGCCGGTGCTTCTTCTGCAGGTACCGTCACCACAAGCTTCGCCCCTGCTGAAAGCGGTTTACAACCCGAAGGCCGTCATCCCGCACGCGGCGTTGCTGCATCAGGCTTCCGCCCATTGTGCAATATTCCCCACTGCTGCCTCCCGTAGGAGTCTGGGCCGTATCTCAGTCCCAATGTGGCCGGTCGCCCTCTCAGGCCGGCTACCCGTCAAAGCCTTGGTAAGCCACTACCCCACCAACAAGCTGATAAGCCGCGAGTCCATCCAAAACCGCCGAAGCTTTCCAACCCCACCATGCAGCAAGGATTCCTATCCGGTATTAGCCCCAGTTTCCCGAAGTTATCCCAGTCTTATAGGTAGGTTA
>n.538.6.bb_141
GTAGTTAGCCGTGGCTTTCTGATTAGGTACCGTCAAGACGTGCATAGTTACTTACACATTTGTTCTTCCCTAACTAACAGAGTTTTACGTATCCGAGACCTTCATCACTCACGCGGCGTTGCTCCGTCAGGCTTTCGTCCCATTGCGGAAGATTCCCTACTGCTGCCTCCCGTAGGAGTCTGGACCGTGTCTCAGTTCCAGTGTGGCCGATCACCCTCTCAGGGTCGGGCTACGCATCGTCGCCTTGGTTAAGCCGTTACCTTACCAACTAGCTAATGCGGCGCGGATCCATCTATAAGTGACAGC
>n.538.6.bb_142
GGAGTTAGCCGGTGCTTCTTCTGCGGGTAACGTCAATTGCTGAGGTTATTAACCTCAACACCTTCCTCCCCGCTGAAAGTACTTTACAACCCGAAGGCCTTCTTCATACACGCGGCATGGCTGCATCAGGCTTGCGCCCATTGTGCAATATTCCCCACTGCTGCCTCCCGTAGGAGTCTGGACCGTGTCTCAGTTCCAGTGTGGCTGGGTCATCCTCTCAGACCAGCTAGGGATCGTCGCCTAGGTGAGCCGTTACCCACCTACTAGCTAATCCCATCTGGGCACATCTGATGGCAAG
>n.538.6.bb_143
GTAGTTAGCCGTGGCTTTCTGATTAGGTACCGTCAAGACGTGCATAGTTACTTACACATTTGTTCTTCCCTAATAACAGAGTTTTACGATCCGAAGACCCTCATCACTCACGCGGCGTTGCTCCGTCAGGCTTTCGCCCATTGCGGAAGATTCCCTACTGCTGCCTCCCGTAGGAGTCTGGACCGTGTCTCAGTTCCAGTGTGGCCGATCACCCTCTCAGGGTCGGCTACGCATCGTTGCCTTGGTAAGCCGTTACCTTACCAACTAGCTAATGCGGCGCGGATCCATCTATAAGTGACAGCAAAACCGTCTTTCACTATTGAACCATGCGGTTC
>n.538.6.bb_144
GGAGTTAGCCGGTGCTTCTTCTGCGGGTAACGTCAATCGATGAGGTTATTAACCTCACCGCCTTCCTCCCCGCTGAAAGTGCTTTACAGCCCGAAGGCCTTCTTCACACACGCGGCATGGCTGCATCAGGCTTGCGCCCATTGTGCAATATTCCCCACTGCTGCCTCCCGTAGGAGTCTGGACCGTGTCTCAGTTCCAGTGTGGGCTGGGTCATCCTCTCAGACCAGCTAGGGATCGTCGCCTAGGTGAGCCGTTACCCACCTACCAGCTAATCCCATCTGGGCACATCTGATGGC
>n.538.6.bb_145
GTAGTTAGCCGTGGCTTTCTGATTAGGTACCGTCAAGACGTGCATAGTTACTTACACATTTGTTCTTCCCTAATAACAGAGTTTTACGATCCGTAGACCTTCATCACTCACGCGGCGTTGCTCCGTCAGGCTTTCGCCCATTGCGGAAGATTCCCTACTGCTGCCTCCCGTAGGAGTCTGGACCGTGTCTCAGTTCCAGTGTGGCCGATCACCCTCTCAGGGTCGGCTACGCATCGTTGCCTTGGTAAGCCGTTACCTTACCAACTAGCTAATGCGGCGCGGATCCATCTATAAGTGACAGCAAAAACCGTCTTTCACTATTGAACCATGCGGTTCAATATATTATCCGGTATT
>n.538.6.bb_146
GTAGTTAGCCGGTGCTTCTTCTGCAGGTACCGTCACCACAAGCTTCGCCCCTGCTGAAAGCGGGTTTACAACCCGAAGGCCGTCATCCCGCACGCGGCGTTGCTGCATCAGGCTTCCGCCCATTGTGCAATATTCCCCACTGCTGCCTCCCGTAGGAGTCTGGGCCGTATCTCAGTCCCAATGTGGCCGGTCGCCCTCTCAGGCCGGCTACCCGTCAAAGCCTTGGTAAGCCACTA
>n.538.6.bb_147
GTAGTTAGCCGGTGCTTCTTCTGCAGGTACCGTCACCACAAGCTTCGCCCCTGCTGAAAGCGGTTTACAACCCGAAGGCCGTCATCCCGCACGCGGCGTTGCTGCATCAGGCTTCCGCCCATTGTGCAATATTCCCCACTGCTGCCTCCCGTAGGAGTCTGGGCCGTATCTCAGTCCCAATGTGGCCGGTCGCCCTCTCAGGCCGGCTACCCGTCAAAGCCTTGGTAAGCCACTACCCACCAACAAGCTGATAAGCCGCGAGTCCATCCAAAACCGCCGAAGCTTTCCAACCCCACCATGCAGCAAGGATTCCTATCCGGTATTAGCCCCAGTTTCC
>n.538.6.bb_148
GTAGTTAGCCGGTGCTTCTTCTGCAGGTACCGTCACCACAAGCTTCGCCCCTGCTGAAAGCGGTTTACAACCCGAAGGCCGTCATCCCGCACGCGGCGTTGCTGCATCAGGCTTCCGCCCATTGTGCAATATTCCCCACTGCTGCCTCCCGTAGGAGTCTGGGCCGTATCTCAGTCCCAATGTGGCCGGTCGCCCTCTCAGGCCGGCTACCCGTCAAAGCCTTGGTAAGCCACTACCCACCAACAAGCTGATAAGCCGCGAGTCCAT
>n.538.6.bb_149
GTAGTTAGCCGGTGCTTCTTCTGCAGGTACCGTCACCACAAGCTTCGCCCCTGCTGAGAGCGGTTTACAACCCGAAGGCCGTCATCCCGCACGCGGCGTTGCTGCATCAGGCTTCCGCCCATTGTGCAATATTCCCCACTGCTGCCTCCCGTAGGAGTCTGGGCCGTATCTCAGTCCCAATGTGGCCGGTCGCCCTCTCAGGCCGGCTACCCGGTCAAAGCCTTGGTAAGCCACTACCCACCAACAAGCTGATAAGCCGCGAGTCCAT
>n.538.6.bb_150
GTAGTTAGCCGGTGCTTCTTCTGCAGGTACCGTCACCACAAGCTTCGCCCCTGCTGAAAGCGGTTTACAACCCGAAGGCCGTCATCCCGCACGCGGCGTTGCTGCATCAGGCTTCCGCCCATTGTGCAATATTCCCCACTGCTGCCTCCCGTAGGAGTCTGGGCCGTATCTCAGTCCCAATGTGGCCGGTCGCCCTCTCAGGCCGGCTACCCGTCAAAGCCTTGGTAAGCCACTACCCACCAACAAGCTGATAAGCCGCGAGTCCATCCAAAACCG
>n.538.6.bb_151
GTAGTTAGCCGGTGCTTCTTCTGCAGGTACCGTCACCACAAGCTTCGCCCCTGCTGAAAGCGGTTTACAACCCGAAGGCCGTCATCCCGCACGCGGCGTTGCTGCATCAGGCTTCCGCCCATTGTGCAATATTCCCCACTGCTGCCTCCCGTAGGAGTCTGGGCCGTATCTCAGTCCCAATGTGGCCGGTCGCCCTCTCAGGGCCCGGCTACCCGGTCAAAGGCCTTGGTAAGCCACTACCCCACCAACAAAGCTGATAAGCCGCGAGTCCATCC
>n.538.6.bb_152
GAGTTAGCCGGTGCTTCTTCTGCGGGTAACGTCAATTGCTGAGGTTATTAACCTCAACACCTTCCTCCCCGCTGAAAGTACTTTACAACCCGAAGGCCTTCTTCATACACGCGGCATGGCTGCATCAGGCTTGCGCCCATTGTGCAATATTCCCCACTGCTGCCTCCCGTAGGAGTCTGGACCGTGTCTCAGTTCCAGTGTGGCTGGGTCATCCTCTCAGACCAGCTAGGGATCGTCGCCTAGGTGAGCCGTTACCCACCTACTAGCTAATCCCATCTGGGCACATCTGAT
>n.538.6.bb_153
GTAGTTAGCCGTGGCTTTCTGATAGGTACCGTCAAGACGGTGCATAGTTACTTACACATTTGTTCCTCCCTAATAACAGTAGTTTTACGATACCGAAGACCTTCATCACTCACGCGGCGTTGCTCCGTCAGGCTTTCGCCCATTGCCGGAAGATTCCCTACTGCCTGCCTCCCGTAGGAGTCTGGACCGTGTCTCAGTTCCAGTG
>n.538.6.bb_154
GTAGTTAGCCGGTGCTTCTTCTGCAGGTACCGTCACCACAAGCTTCGCCCCTGCTGAAAGCGGTTTACAACCCGAAGGCCGTCATCCCGCACGCGGCGTTGCTGCATCAGGCTTCCGCCCATTGTGCAATATTCCCCACTGCTGCCTCCCGTAGGAGTCTGGGCCGTATCTCAGTCCCAATGTGGCCGGTCGCCCTCTCAGGCCGGCTACCCGTCAAAGCCTTGGTAAGCCACTACCCCACCAACAAGCTGATAAGCCGCGAGTCCATCCAAAACCGCCGAAGCTTTCCAACCCCACCATGCAGCAAGGATTCCTATCCGGTATTAGCCCCAGTTTCCTGAAGTTATCCCGAAGTCAAAGGGCAGGTTACTCACGTGTTACTCACCCGTTCGCCA
>n.538.6.bb_155
GTAGTTAGCCGGTGCTTCTTCTGCAGGTACCGTCACCACAAGCTTCGCCCCTGCTGAAAGCGGTTTACAACCCGAAGGCCGTCATCCCGCACGCGGCGTTGCTGCATCAGGCTTCCGCCCATTGTGCAATATTCCCCACTGCTGCCTCCCGTAGGAGTCTGGGCCGTATCTCAGTCCCAATGTGGCCGGTCGCCCTCTCAGGCCGGCTACCCGTCAAAGCCTTGGTAAGCCACTACCCCACCAACAAGCTGATAAGCCGCGAGTCCATCC
>n.538.6.bb_156
GTAGTTAGCCGGTGCTTCTTCTGCAGGTACCGTCACCACAAGCTTCGCCCCTGCTGAAAGCGGTTTACAACCCGAAGGCCGTCATCCCGCACGCGGCGTTGCTGCATCAGGCTTCCGCCCATTGTGCAATATTCCCCACTGCTGCCTCCCGTAGGAGTCTGGGCCGTATCTCAGTCCCAATGTGGCCGTCGCCCTCTCAGGCCGGCTACCCGGTCAAAGCCTTGGTAAGCCACTACCCCACCAACAAGCTGATAAGCCGCGAGTCCATCCAAAACCGCCGAAGCTTTCCAACCCCACCATGCAGCAAGGATTCCTATCCGGTAT
>n.538.6.bb_157
GTAGTTAGCCGGTGCTTCTTCTGCAGGTACCGTCACCACAAGCTTCGCCCCTGCTGAAAGCGGTTTACAACCCGAAGGCCGTCATCCCGCACGCGGCGTTGCTGCATCAGGCTTCCGCCCATTGTGCAATATTCCCCACTGCTGCCTCCCGTAGGAGTCTGGGCCGTATCTCAGTCCCAATGTGGCCGGTCGCCCTCTCAGGCCGGGCTACCCGGTCAAAGCCTTGGTAAGCCACTACCCCACCAACAAGCTGATAAGCCGCGAGTCCATCCAAAACCGCCGAAGCTTTCCAACCCCACCATGCAGCAAGGATTCCTATCCGGTATTAGCCCCAGTTTCCTGAAGTTATCCCGAAGT
>n.538.6.bb_158
GGAGTTAGCCGGTGCTTCTTCTGCGGGTAACGTCAATCGACAAGGTTATTAACCTTATCGCCTTCCTCCCCGCTGAGAGTACTTTACAACCCGAAGGCCTTCTTCATACACGCGGCATGGCTGCATCAGGCTTGCGCCCATTGTGCAATATTCCCCACTGCTGCCTCCCGTAGGAGTCTGGACCGTGTCTCAGTTCCAGTGTGGCTGGGTCATCCTCTCAGACCAGCTAGGGATCGTCGCCTAGGTGAGCCGTTACCCCACCTACTAGCTAATCCCATCTGGGCACATCTGATGGCAAGAGG
>n.538.6.bb_159
GTAGTTAGCCGTGGCTTTCTGATTAGGTACCGTCAAGACGTGCATAGTTACTTACACATTTGTTCTTCCCTAATAACAGAGTTTTACGATCCGAAGACCTTCATCACTCACGCGGCGTTGCTCCGTCAGGCTTTCGCCCATTGCGGAAGATTCCCTACTGCTGCCTCCCGTAGGAGTCTGGACCGTGTCTCAGTTCCAGTGTGGCCGATCACCCTCTCAGGGTCGGCTACGCATCGTCGCCTTGGTAAGCCGTTACCTTACCAACTAGCTAATGCGGCGCGGATCCATCTATAAGTGACAGCAAAACCGTCTTTCACTATTGAACCATGCGGTTCAATATATTATCCGGTATTAGCTCGGTTTCCCGAAGTTATCCCAGTCTTATAGGTAGGTTATCCCACGTGTTACTCACCCCGTCCCGCCCGCTAACGTCCAGAGGGAGCAAGCTCCCTCGTCTGTTCGCTCGACTTGCATGTATTAGGGCACGCCGCCAGCGTTCATCCTGA
>n.538.6.bb_160
GTAGTTAGCCGTGGCTTTCTGATTAGGTACCGTCAAGACGTGCATAGTTACTTACACATTTGTTCTTCCCTAATAACAGAGTTTTACGATCCGAAGACCTTCATCACTCACGCGGCGTTGCTCCGTCAGGCTTTCGCCCATTGCGGAAGATTCCCTACTGCTGCCTCCCGTAGGAGTCTGGACCGTGTCTCAGTTCCAGTGTGGCCGATCACCCTCTCAGGGTCGGCTACGCATCGTCGCCTTGGTAAGCCGTTACCTTACCAACTAGCTAATGCGGCGCGGATCCATCTATAAGTGACAGCAAAAACCGTCTTTCACTATTGAACCATGCGGTTCAATATATTATCCGGTATTAGCTCCGGTTTCCCGAAGTTATCCCAGTCTTATAGGTAGGTTATCCCACGTGTTACTCACCCCGTCCCGCCCGCTAACGTCAGAGGAGCAAGCCTCCCTCGTCTGTTCGCTCGACTTGCATGTATTAGGGCACGCCGCCAGCGTTCATCCT
>n.538.6.bb_161
GTAGTTAGCCGGTGCTTCTTCTGCAGGTACCGTCACCACAAGCTTCGCCCCTGCTGAAAGCGGTTTACAACCCGAAGGCCGTCATCCCGCACGCGGCGTTGCTGCATCAGGCTTCCGCCCATTGTGCAATATTCCCCACTGCTGCCTCCCGTAGGAGTCTGGGCCGTATCTCAGTCCCAATGTGGCCGGTCGCCCTCTCAGGCCGGCTACCCGTCAAAGCCTTGGTAAGCCACTACCCCACCAACAAGCTGATAAGCCGCGAGTCCATCCAAAAACCGCCGAAGCTTTTCCAACCCCACCATGCAGCAAGGATTCCTATCCGGTATTAGCCCCAGTTTCCTGAAGTTATCCCGAAGTCAAAGGGCAGGTTACTCACGTGTTACTCACCCGTTCGCCACTCGACGTA
>n.538.6.bb_162
GGAGTTAGCCGGTGCTTCTTCTGCGGGTAACGTCAATCGACAAGGTTATTAACCTTATCGCCTTCCTCCCCGCTGAAAGTACTTTACAACCCGAAGGCCTTCTTCATACACGCGGCATGGCTGCATCAGGCTTGCGCCCATTGTGCAATATTCCCCACTGCTGCCTCCCGTAGGAGTCTGGACCGTGTCTCAGTTCCAGTGTGGCTGGTCATCCTCTCAGACCAGCTAGGGATCGTCGCCTAGGTGAGCCGTTACCCACCTACTAGCTAATCCCATCTGGGCACATCTGATGGCAA
>n.538.6.bb_163
GTAGTTAGCCGGTGCTTCTTCTGCAGGTACCGTCACCACAAGCTTCGCCCCTGCTGAAAGCGGTTTACAACCCGAAGGCCGTCATCCCGCACGCGGCGTTGCTGCATCAGGCTTCCGCCCATTGTGCAATATTCCCCACTGCTGCCTCCCGTAGGAGTCTGGGCCGTATCTCAGTCCCAATGTGGCCGGTCGCCCTCTCAGGCCGGCTACCCGTCAAAGCCTTGGTAAGCCACTACCCCACCAACAAGCTGATAAGCCGCGAGTCCATCCAAAACCGCCGAAGCTTTCCAACCCCACCATGCAGCAAGGATTCCTATCCGGTAT
>n.538.6.bb_164
GGAGTTAGCCGGTGCTTCTTCTGCGGGTAACGTCAATCGACAGGGTTATTAACCCTGTCGCCTTCCTCCCCGCTGAAAGTACTTTACAACCCGAAGGCCTTCTTCATACACGCGGCATGGCTGCATCAGGCTTGCGCCCATTGTGCAATATTCCCCACTGCTGCCTCCGTAGGAGTCTGGACCGTGTCTCAGTTCCAGTGTGGCTGGTCATCCTCTCAGACCAGCTAGGGATCGTCGCCTAGGTGAGCCGTTACCCACCTACTAGCTAATCCCATCTGGGCACATCCGATGGCAAGAGGCCCGAAGATCCCCCTCTTTGGTCTTGCGACGTTATGCGGTATT
>n.538.6.bb_165
GTAGTTAGCCGGTGCTTCTTCTGCAGGTACCGTCACCACAAGCTTCGCCCCTGCTGAAAGCGGTTTACAACCCGAAGGCCGTCATCCCGCACGCGGCGTTGCTGCATCAGGCTTCCGCCCATTGTGCAATATTCCCCACTGCTGCCTCCCGTAGGAGTCTGGGCCGTATCTCAGTCCCAATGTGGCCGGTCGCCCTCTCAGGCCGGCTACCCGTCAAAGCCTTGGTAAGCCACTACCCACCAACAAGCTGATAAGCCGCGAGTCCATCCAAAACCGCC
>n.538.6.bb_166
GTAGTTAGCCGGTGCTTCTTCTGCAGGTACCGTCACCACAAGCTTCGCCCCTGCTGAAAGCGGTTTACAACCCGAAGGCCGTCATCCCGCACGCGGCGTTGCTGCATCAGGCTTCCGCCCATTGTGCAATATTCCCCACTGCTGCCTCCCGTAGGAGTCTGGGCCGTATCTCAGTCCCAATGTGGCCGGTCGCCCTCTCAGGCCGGCTACCCGTCAAAGCCTTGGTAAGCCACTACCCCACCAACAAGCTGATAAGCCGCGAGTCCATCCAAAACCGC
>n.538.6.bb_167
GGAGTTAGCCGGTGCTTCTTCTGCGGGTAACGTCAATCGACAAGGTTATTAACCTTATCGCCTTCCTCCCCGCTGAAAGTACTTTACAACCCGAAGGCCTTCTTCATACACGCGGCATGGCTGCATCAGGCTTGCGCCCATTGTGCAATATTCCCCACTGCTGCCTCCCGTAGGAGTCTGGACCGTGTCTCAGTTCCAGTGTGGCTGGTCATCCTCTCAGACCAGCTAGGGATCGTCGCCTAGGTGAGCCGTTACCCACCTACTAGCTAATCCCATCTGGGCACATCTGATGGC
>n.538.6.bb_168
GTAGTTAGCCGTGGCTTTCTGATTAGGTACCGTCAAGACGTGCATAGTTACTTACACATTTGTTCTTCCCTAATAACAGAGTTTTACGATCCGAAGACCTTCATCACTCACGCGGCGTTGCTCCGTCAGGCTTTCGCCCATTGCGGAAGATTCCCTACTGCTGCCTCCCGTAGGAGTCTGGACCGTGTCTCAGTTCCAGTGTGGCCGATCACCCTCTCAGGGTCGGCTACGCATCGTTGCCTTGGTAAGCCGTTACCTTACCAACTAGCTAATGCGGCGCGGATCCATCTATAAGTGACAGCAAAACCGTCTTTCACTATTGAACCATGCGGTTCAATATATTATCCGGTATT
>n.538.6.bb_169
GTAGTTAGCCGGTGCTTCTTCTGCAGGTACCGTCACCACAAGCTTCGCCCCTGCTGAAAGCGGTTTACAACCCGAAGGCCGTCATCCCGCACGCGGCGTTGCTGCATCAGGCTTCCGCCCATTGTGCAATATTCCCCACTGCTGCCTCCCGTAGGAGTCTGGGCCGTATCTCAGTCCCAATGTGGCCGATCGCCCTCTCAGGCCGGGCTACCCGTCAAAGCCTTGGTAAGCCACTACCCACCAACAAGCTGATAAGCCGCGAGTCCATCCAAAACGCCGAAGCTTTCCAACCCCACCATGCAGCAAGGATTCCTA
>n.538.6.bb_170
TAGTTAGCCGTGGCTTTCTGATTAGGTACCGTCAAGACGTGCATAGTTACTTACACATTTGTTCTTCCCTAATAACAGAGTTTTACGATCCGAAGACCTTCATCACTCACGCGGCGTTGCTCCGTCAGGCTTTCGCCCATTGCGGAAGATTCCCTACTGCTGCCTCCCGTAGGAGTCTGGACCGTGTCTCAGTTCCAGTGTGGCCGATCACCCTCTCAGGGTCGGCTACGCATCGTTGCCTTGGTAAGCCGTTACCTTACCAACTAGCTAATGCGGCGCGGATCCATCTATAAGTGAC
>n.538.6.bb_171
GTAGTTAGCCGTGGCTTTCTGATTAGGTACCGTCAAGACGTGCATAGTTACTTACACATTTGTTCTTCCCTAATAACAGAGTTTTACGATCCGAAGACCTTCATCACTCACGCGGCGTTGCTCCGTCAGGCTTTCGCCCATTGCGGAAGATTCCCTACTGCTGCCTCCCGTAGGAGTCTGGACCGTGTCTCAGTTCCAGTGTGGCCGATCACCCTCTCAGGGTCGGCTACGCATCGTCGCCTTGGTAAGCCGTTACCTTACCAACTAGCTAATGCGGCGCGGATCCATCTATAAGTGACAGCAAAACCGTCTTTCACTATTGAACCATGCGGTTCAATATATTATCCGGTATTAGCTCCGGTTTCCGAAGTTATCCCAGTCTTATAGGTA
>n.538.6.bb_172
GTAGTTAGCCGTGGCCTTCTGATTAGGTACCGTCAAGACGTGCATAGTTACTTACACATTTGTTCTTCCCTAATAACAGAGTTTTACGATCCGAAGACCTTCATCACTCACGCGGCGTTGCTCCGTCAGGCTTTCGCCCATTGCGGAAGATTCCCTACTGCTGCCTCCCGTAGGAGTCTGGACCGTGTCTCAGTTCCAGTGTGGCCGATCACCCTCTCAGGGTCGGCTACGCATCGTTGCCTTGGTAAGCCGTTACCTTACCAACTAGCTAATGCGGCGCGGATCCATCTATAAGTGACAGCAAAACCGTCTTTCACTATTGAACCATGCGGTTCAATATATTATCCGGTATTAGCTCCGGTTTCCCGAAGTTATCCCAGTCTTATAGGTAGGTTATCCCACGTGTTACTCACCCCGTCCCGCCCGCTAACGTCAGAGAAGCAAGCTCCCTCGTCTGTTCCGCTCGACTTGCCATGTATTAGGGCACGCCGCCAGCGTTC
>n.538.6.bb_173
GTAGTTAGCCGTGGCTTTCTGATTAGGTACCGTCAAGACGTGCATAGTTACTTACACATTTGTTCTTCCCTAATAACAGAGTTTTACGATCCGAAGACCTTCATCACTCACGCGGCGTTGCTCCGTCAGGCTTTCGCCCATTGCGGAAGATTCCCTACTGCTGCCTCCCGTAGGAGTCTGGACCGTGTCTCAGTTCCAGTGTGGCCGATCACCCTCTCAGGGTCGGCTACGCATCGTTGCCTTGGTAAGCCGTTACCTTACAACTAGCTAATGCGGCGCGGATCCATCTATAAGTGACAGCAAAAACCGTCTTTCACTATTGAACCATGCGGTTCAATATATTATCCGGTATTAGCTCCGGTTTCCGAAGTTATCCCAGTCTTATAGGTAGGTTATC
>n.538.6.bb_174
GTAGTTAGCCGGTGCTTCTTCTGCAGGTACCGTCACCACAAGCTTCGCCCCTGCTGAAAGCGGTTTACAACCCGAAGGCCGTCATCCCGCACGCGGCGTTGCTGCATCAGGCTTCCGCCCATTGTGCAATATTCCCCACTGCTGCCTCCCGTAGGAGTCTGGGCCGTATCTCAGTCCCAATGTGGCCGTCGCCCTCTCAGGCCGGCTACCCGGTCAAAGCCTTGGTAAGCCACTACCCCACCAACAAGCTGATAAGCCGCGAGTCCATCCAA
>n.538.6.bb_175
GTAGTTAGCCGTGGCTTTCTGATTAGGTACCGTCAAGACGTGCATAGTTACTTACACATTTNTTCTTCCCTAATAACAGAGTTTTACGATCCGAAGACCTTCATCACTCACGCGGCGTTGCTCCGTCAGGCTTTCGCCCATTGCGGAAGATTCCCTACTGCTGCCTCCCGTAGGAGTCTGGACCGTGTCTCAGTTCAGTGTGGCCGATCACCCTCTCAGGGTCGGCTACGCATCGTGCCTTGGTAAGCCGTTACCTTACCAACTAGCTAATGCGGCGCGGATCCATCTATAAGTGACAGCAAAACCGTCTTTCACTATTGAACCATGCGGTTCAATATATTATCCGGTATTAGCTCCGGTTTCCCGAAGTTATCCCAGTCTTATAGGTAGGTTATCCCACCGTGTTACTCACCCCGTCCCGCCCGCTAACGTCA
>n.538.6.bb_176
GGAGTTAGCCGGTGCTTCTTCTGCGGGTAACGTCAATCGACAAGGTTATTAACCTTATCGCCTTCCTCCCCGCTGAAAGTACTTTACAACCCGAAGGCCTTCTTCATACACGCGGCATGGCTGCATCAGGCTTGCGCCCATTGTGCAATATTCCCCACTGCTGCCTCCCGTAGGAGTCTGGACCGTGTCTCAGTTCCAGTGTGGCTGGTCATCCTCTCAGACCAGCTAGGGATCGTCGCCTAGGTGAGCCGTTACCCACCTACTAGCTAATCCCATCTGGGCACATCTGATGGCAAGAGGCCCGAAGGTCCCCCTCTTTGGTCTTGCGACGTTATGCGGTAT
>n.538.6.bb_177
GTAGTTAGCCGGTGCTTCTTCTGCAGGTACCGTCACCACAAGCTTCGCCCCCGCTGAAAGTACTTTGCAACCCGAAGGCCTTCTTCATACACGCGGCATGGCTGCATCAGGCTTGCGCCCATTGTGCAATATTCCCCACTGCTGCCTCCCGTAGGAGTCTGGACCGTGTCTCAGTTCCAGTGTGGCTGGTCATCCTCTCAGACCAGCTAGGGATCGTCGCCTAGGTGAGCCGTTACCCACCTACTAGCTAATCCATCTGGGCACATCTGATGGCAA
>n.538.6.bb_178
GTAGTTAGCCGTGGCTTTCTGATTAGGTACCGTCAAGACGTGCATAGTTACTTACACATTTGTTCTTCCCTAATAACAGAGTTTTACGATCCGAAGACCTTCATCACTCACGCGGCGTTGCTCCGTCAGGCTTTCGCCCATTGCGGAAGATTCCCTACTGCTGCCTCCCGTAGGAGTCTGGACCGTGTCTCAGTTCCAGTGTGGCCGATCACCCTCTCAGGGTCGGCTACGCATCGTTGCCTTGGTAAGCCGTTACCTTACCAACTAGCTAATGCGGCGCGGATCCATCTATAAGTGACAGCAAAACCGTCTTTCACTATTGAACCATGCGGTTCAATATATTATCCGGTATTAGCTCCGGTTTCCCGAAGTTATCCCAGTCTTATAGGTAGGTTATC
>n.538.6.bb_179
GTAGTTAGCCGTGGCTTTCTGATTAGGTACCGTCAAGACGTGCATAGTTACTTACACATTTGTTCTTCCCTAATAACAGAGTTTTACGATCCGAAGACCTTCATCACTCACGCGGCGTTGCTCCGTCAGGCTTTCGCCCATTGCGGAAGATTCCCTACTGCTGCCTCCCGTAGGAGTCTGGACCGTGTCTCAGTTCCAGTGTGGCCGATCACCCTCTCAGGGTCGGCTACGCATCGTTGCCTTGGTAAGCCGTTACCTTACCAACTAGCTAATGCGGCGCGGATCCATCTATAAGTGACAGCAAAACCGTCTTTCAC
>n.538.6.bb_180
GTAGTTAGCCGGTGCTTCTTCTGCGGGTAACGTCAATCGACAAGGTTATTAACCTTATCGCCTTCCTCCCCGCTGAAAGTACTTTACAACCCGAAGGCCTTCTTCATACACGCGGCATGGCTGCATCAGGCTTGCGCCCATTGTGCAATATTCCCCACTGCTGCCTCCGTAGGAGTCTGGACCGTGTCTCAGTTCCAGTGTGGCTGGTCATCCTCTCAGACCAGCTAGGGATCGTCGCCTAGGTGAGCCGTTACCCACCTACTAGCTAATCCCATCTGGGCACATCTGATG
>n.538.6.bb_181
GTAGTTAGCCGTGGCTTTCTGATTAGGTACCGTCAAGACGTGCATGGTTACTTACACATTTGTTCTTCCCTAATAACAGAGTTTTACGATCCGAAGACCTTCATCACTCACGCGGCGTTGCTCCGTCAGGCTTTCGCCCATTGCGGAAGATTCCCTACTGCTGCCTCCCGTAGGAGTCTGGACCGTGTCTCAGTTCCAGTGTGGCCGATCACCCTCTCAGGGTCGGCTACGCATCGTTGCCTTGGTAAGCCGTTACCTTACCAACTAGCTAATGCGGCGCGGATCCATCTATAAGTGACAGCAAAACCGTCTTTCACTATTGAACCATGCGGTTCAATATATTATCCGGTATTAGCTCCGGTTTCCGAAGTTATCCCAGTCTTATAGGTAGGTTATCCCACGTGTT
>n.538.6.bb_182
GGAGTTAGCCGGTGCTTCTTCTGCGGGTAACGTCAATCGACAAGGTTATTAACCTTATCGCCTTCCTCCCCGCTGAAAGTACTTTACAACCCGAAGGCCTTCTTCATACACGCGGCATGGCTGCATCAGGCTTGCGCCCATTGTGCAATATTCCCCACTGCTGCCTCCGTAGGAGTCTGGACCGTGTCTCAGTTCCAGTGTGGCCGATCACCCTNTCAGGTCGCTACGCATCGTTGCCTTGGTAAGCCGTTACTTACCAACTAGCTAATGCGGCGCGGATCCATCTATAAGTGAC
>n.538.6.bb_183
GTAGTTAGCCGGTGCTTCTTCTGCAGGTACCGTCACCACAAGCTTCGCCCCTGCTGAAAGCGGTTTACAACCCGAAGGCCGTCATCCCGCACGCGGCGTTGCTGCATCAGGCTTCCGCCCATTGTGCAATATTCCCCACTGCTGCCTCCCGTAGGAGTCTGGGCCGTATCTCAGTCCCAATGTGGCCGGTCGCCCTCTCAGGCCGGCTACCCGTCAAAGCCTTGGTAAGCCACTACCCACCAACAAGCTGATAAGCCGCGAGTCTATCCAAAACCGCCGAAGCTTTCCAACCCCCACCATGCAGCAAGGATTCCTATCCGGTATTAGCCCCAGTTTCCTGAAGTT
>n.538.6.bb_184
GTAGTTAGCCGGTGCTTCTTCTGCAGGTACCGTCACCACAAGCTTCGCCCCTGCTGAAAGCGGTTTACAACCCGAAGGCCGTCATCCCGCACGCGGCGTTGCTGCATCAGGCTTCCGCCCATTGTGCAATATTCCCCACTGCTGCCTCCCGTAGGAGTCTGGGCCGTATCTCAGTCCCAATGTGGCCGGTCGCCCTCTCAGG
>n.538.6.bb_185
GTAGTTAGCCGGTGCTTCTTCTGCAGGTACCGTCACCACAAGCTTCGCCCCTGCTGAAAGCGGTTTACAACCCGAAGGCCGTCATCCCGCACGCGGCGTTGCTGCATCAGGCTTCCGCCCATTGTGCAATATTCCCCACTGCTGCCTCCCGTAGGAGTCTGGGCCGTATCTCAGTCCCAATGTGGCCGGTCGCCCTCTCAGGCCGGCTACCCGTCAAAGCCTTGGTAAGCCACTACCCCACCAACAAGCTGATAAGCCGCGAGTCCATCCAAAAACCGCCGAAGCTTTCCAACCCCCACCATGCAGCAAGGATTCCTATCCGGTATTAGCCCCAGTTTCCTGAAGTTATCCCGAAGTCAAGGGCAGGTTACTCACGTGTTACTCACCCGTTCGCCA
>n.538.6.bb_186
GTAGTTAGCCGTGGCTTTCTGATTAGGTACCGTCAAGACGTGCATAGTTACTTACACATTTGTTCTTCCCTAATAACAGAGTTTTACGATCCGAAGACCTTCATCACTCACGCGGCGTTGCTCCGTCAGGCTTTCGCCCATTGCGGAAGATTCCCTACTGCTGCCTCCCGTAGGAGTCTGGACCGTGTCTCAGTTCCAGTGTGGCCGATCACCCTCTCAGGGTCGGCTACGCATCGTTGCCTTGGTAAGCCGTTACCTTACCAACTAGCTAATGCGACGCGGATCCATCTATAAGTGACAGCAAAACCGTCTTTCACTATTGAACCATGCGGTTCAATATATTATCCGGTATTAGCTCCGGTTTCCCGAAGTTATCCCAGTCTTATAGGTAGGTTATCCCACGTGTTACTCACCCCGTCCCGCCCGCTAACGTCAGAGGGAGCAAGCTCCCTCGTCTGTTCGCTCGACTTG
>n.538.6.bb_187
GTAGTTAGCCGTGGCTTTCTGATTAGGTACCGTCAAGACGTGCATAGTTACTTACACATTTGCTCTTCCTTAATAACAGAGTTTTACGATCCGAAGACCTTCATCACTCACGCGGCGTTGCTCCGTCAGGCTTTCGCCCATTGCGGAAGATTCCCTACTGCTGCCTCCGTAGGAGTCTGGACCGTGTCTCAGTTCCAGTGTGGCCGATCACCCTCTCAGGTCGCTACGCATCGTTGCCTTGGTAAGCCGTTACCTTACCAACTAGCTAATGCGGCGCGGATCCATCTATAAGTGACAGCAAAACCGTCTTTCACTATTGAACCATGCGGTTCAATATATTATCCGGTATTAGCTCCGGT
>n.538.6.bb_188
GTAGTTAGCCGTGGCTTTCTGATTGGGTACCGTCAAGACGTGCATAGTTACTTACACATTTGTTCTTCCCTAATAACAGAGTTTTACGATCCGAAGACCTTCATCACTCACGCGGCGTTGCTCCGTCAAGCTTTCGCCCATTGCGGAAGATTCCCTACTGCTGCCTCCCGTAGGAGTCTGGACCGTGTCTCAGTTCCAGTGTGGCCGATCACCCTCTCAGGGTCGGCTACGCATCGTTGCCTTGGTAAGCCGTTACCTTACCAACTAGCTAATGCGGCGCGGATCCATCTATAAGTGACAGCAAAACCGTCTTTCACTATTGAACCATGCGGTTCAATATATTATCCGGTATTAGCTCCGGTTTCCCGAAGTTATCCCAGTCTTATAGGTAGGTTATCCCACGTGTTACTCACCCCGTCCCGCCCGCTAACGTCA
>n.538.6.bb_189
GGGGTTAGCCGGTGCTTCTTCTGCGGGTAACGTCAATCGACAGGGTTATTAACCCTGTCGCCTTCCTCCCCGCTGAAAGTACTTTACAACCCGAAGGCCTTCTTCATACACGCGGCATGGCTGCATCAGGCTTGCGCCCATTGTGCAATATTCCCCACTGCTGCCTCCCGTAGGAGTCTGGACCGTGTCTCAGTTCCAGTGTGGCTGGTCATCTTCTCAGACCCAGCTAGGGGACCGTCGCCTAGGTG
>n.538.6.bb_190
GTAGTTAGCCGTGGCTTTCTGATTAGGTACCGTCAAGACGTGCATAGTTACTTACACATTTGTTCTTCCCTAATAACAGAGTTTTACGATCCGAAGACCTTCATCACTCACGCGGCGTTGCTCCGTCAGGCTTTCGCCCATTGCGGAAGATTCCCTACTGCTGCCTCCCGTAGGAGTCTGGACCGTGTCTCAGTTCCAGTGTGGCCGATCACCCTCTCAGGGTCGGCTACGCATCGTTGCCTTGGTAAGCCGTTACCTTACCAACTAGCTAATGCGGCGCGGATCCATCTATAAGTGACAGCAAAACCGTCTTTCACTATTGAACCATGCGGTTCAATATATTATCCGGTATT
>n.538.6.bb_191
GTAGTTAGCCGTGGCTTTCTGATTAGGTACCGTCAAGACGTGCATAGTTACTTACACATTTGTTCTTCCCTAATAACAGAGTTTTACGATCCGAAGACCTTCATCACTCACGCGGCGTTGCTCCGTCAGGCTTTCGCCCATTGCGGAAGATTCCCTACTGCTGCCTCCCGTAGGAGTCTGGACCGTGTCTCAGTTCCAGTGTGGCCGATCACCCTCTCAGGGTCGGCTACGCATCGTCGCCTTGGTAAGCCGTTACCTTACCAACTAGCTAATGCGGCGCGGATCCATCTATAAGTGACAGCAAAACCGTCTTTCACTATTGAACCATGCGGTTCAATATATTATCCGGTATTAGCTCCGGTTTCCCGAAGTTATCCCAGTCTTATAGGTAGGTTATCCCACGTGTT
>n.538.6.bb_192
GTAGTTAGCCGGTGCTTCTTCTGCAGGTACCGTCACCACAAGCTTCGCCCCTGCTGAAAGCGGTTTACAACCCGAAGGCCGTCATCCCGCACGCGGCGTTGCTGCATCAGGCTTCCGCCCATTGTGCAATATTCCCCACTGCTGCCTCCCGTAGGAGTCTGGGCCGTATCTCAGTCCCAATGTGGCCGGTCGCCCTCTCAGGCCGGCTACCCGGTCAAAGCCTTGGTAAGCCACTACCCCACCAACAAGCTGATAAGCCGCGAGTCCATCCAAAACCGCCGAA
>n.538.6.bb_193
GTAGTTAGCCGGTGCTTCTTCTGCAGGTACCGTCACCACAAGCTTCGCCCCTGCTGAAAGCGGTTTACAACCCGAAGGCCGTCATCCCGCACGCGGCGTTGCTGCATCAGGCTTCCGCCCATTGTGCAATATTCCCCACTGCTGCCTCCCGTAGGAGTCTGGGCCGTATCTCAGTCCCAATGTGGCCGGTCGCCCTCTCAGGCCGGCTACCCGGTCAAAGGCCTTGGTAAGCCACTACCCCACCAACAAGCTGATAAGCCGCGAGTCCATCCAAAACCGCCGAAGCTTTTCCAACCCCACCATGCAGCAAGGATTCCTATCCGGTATTAGCCCCAGTTTCCTGAAGTTATCCCGAAGTCAAAGGGCAGGTTACTCACGTGTTACTCACCCGTTCGCCA
>n.538.6.bb_194
GGAGTTAGCCGGTGCTTCTTCTGCGGGTAACGTCAATCGACAAGGTTATTAACCTTATCGCCTTCCTCCCCGCTGAAAGTACTTTACAACCCGAAGGCCTTCTTCATACACGCGGCATGGCTGCATCAGGCTTGCGCCCATTGTGCAATATTCCCCATGCTGCCTCCGTAGGAGTCTGGACCGTGTCTCAGTTCCAGTGTGGCTGGGTCATCCTCTCAGACCAGCTAGGGATCGTCGCCTAGGTGAGCCGTTACCCACCTACTAGCTAATCCCATCTGGGCACATCTGATGGCAAGAGGCCCGAAGGT
>n.538.6.bb_195
GTAGTTAGCCGTGGCTTTCTGATTAGGTACCGTCAAGACGTGCATAGTTACTTACACATTTATTCTTCCCTAATAACAGAGTTTTACGATCCGAAGACCTTCATCACTCACGCGGCGTTGCTCCGTCAGGCTTTCGCCCATTGCGGAAGATTCCCTACTGCTGCCTCCCGTAGGAGTCTGGACCGTGTCTCAGTTCCAGTGTGGCCGATCACCCTCTCAGGGTCGGCTACGCATCGTTGCCTTGGTAAGCCGTTACCTTACCAACTAGCTAATGCGGCGCGGATCCATCTATAAGTGACAGCAAAACCGTCTTTCACTATTGAACCATGCGGTTCAATATATTATCCGGTATTAGCTCCGGT
>n.538.6.bb_196
GTAGTTAGCCGGTGCTTCTTCTGCAGGTACCGTCACCACAAGCTTCGCCCCTGCTGAAAGCGGTTTACAACCCGAAGGCCGTCATCCCGCACGCGGCGTTGCTGCATCAGGCTTCCGCCCATTGTGCAATATTCCCCACTGCTGCCTCCCGTAGGAGTCTGGGCCGTATCTCAGTCCCAATGTGGCCGGTCGCCCTCTCAGGCCGGCTACCCGTCAAAGCCTTGGTAAGCCACTACCCCACCAACAAGCTGATAAGCCGCGAGTCCATCCAAAACCGCCGAAGCTTTCCAACCCCACCATGCAGCAAGGATTCCTATCCGGTATTAGCCCCAGTTTCC
>n.538.6.bb_197
GTAGTTAGCCGGTGCTTCTTCTGCAGGTACCGTCACCACAAGCTTCGCCCCTGCTGAAAGCGGTTTACAACCCGAAGGCCGTCATCCCGCACGCGGCGTTGCTGCATCAGGCTTCCGCCCATTGTGCAATATTCCCCACTGCTGCCTCCCGTAGGAGTCTGGGCCGTATCTCAGTCCCAATGTGGCCGGTCGCCCTCTCAGGCCGGCTACCCGTCAAAGCCTTGGTAAGCCACTACCCACCAACAAGCTGATAAGCCGCGAGTCCATCCAAAACCGCCGAAGCTTTTCCAACCCCACCATGCAGCAAGGATTCCTATCCGGTATTAGCCCCAGTTTCCTGAAGTTATCCCCGAAGTCAAGGGCAGGTTACTCACGTGTTACTCACCCGTTCGCCACT
>n.538.6.bb_198
GGAGTTAGCCGGTGCTTCTTCTGCGGGTAACGTCAATCGACAAGGTTATTAACCTTATCGCCTTCCTCCCCGCTGAAAGTACTTTACAACCCGAAGGCCTTCTTCATACACGCGGCATGGCTGCATCAGGCTTGCGCCCATTGTGCAATATTCCCCATGCTGCCTCCCGTAGGAGTCTGGACCGTGTCTCAGTTCCAGTGTGGCT
>n.538.6.bb_199
GTAGTTAGCCGGTGCTTCTTCTGCAGGTACCGTCACCACAAGCTTCGCCCCTGCTGAAAGCGGTTTACAACCCGAAGGCCGTCATCCCGCACGCGGCGTTGCTGCATCAGGCTTCCGCCCATTGTGCAATATTCCCCACTGCTGCCTCCCGTAGGAGTCTGGGCCGTATCTCAGTCCCAATGTGGCCGTCGCCCTCTCAGGCCGGCTA
>n.538.6.bb_200
GTAGTTAGCCGTGGCTTTCTGATTAGGTACCGTCAAGACGTGCATAGTTACTTACACATTTGTTCTTCCCTAATAACAGAGTTTTACGATCCGAAGACCTTCATCACTCACGCGGCGTTGCTCCGTCAGGCTTTCGCCCATTGCGGAAGATTCCCTACTGCTGCCTCCCGTAGGAGTCTGGACCGTGTCTCAGTTCCAGTGTGGCCGATCACCCTCTCAGGGTCGGCTACGCATCGTTGCCTTGGTAAGCCGTTACCTTACCAACTAGCTAATGCGGCGCGGATCCATCTATAAGTGACAGCAAAACCGTCTTTCACTATTGAACCATGCGGTTCAATATATTATCCGGTATTAGCT
>n.538.6.bb_201
GTAGTTAGCCGTGGCTTTCTGATTAGGTACCGTCAAGACGTGCATGGTTACTTACACATTTGTTCTTCCCTAATAACAGAGTTTTACGATCCGAAGACCTTCATCACTCACGCGGCGTTGCTCCGTCAGGCTTTCGCCCATTGCGGAAGATTCCCTACTGCTGCCTCCCGTAGGAGTCTGGACCGTGTCTCAGTTCCAGTGTGGCCGATCACCCTCTCAGGGTCGGCTACGCATCGTTGCCTTGGTAAGCCGTTACCTTACACTAGCTAATGCGGCGCGGATCCATCTATAAGTGACAGCAAAACCGTCTTTCACTATTGAACCATGCGGTTCAATATATTATCCGGTATTAGCTCCGGTTTCCGAAGTTATCCCAGTCTTATAGGTAGGTTATC
>n.538.6.bb_202
GTAGTAAGCCGTGGCTTTCTGATTAGGTACCGTCAAGACGTGCATAGTTACTTACACATTTGTTCTTCCCTAATAACAGAGTTTTACGATCCGAAGACCTTCATCACTCACGCGGCGTTGCTCCGTCAGGCTTTCGCCCATTGCGGAAGATTCCCTACTGCTGCCTCCGTAGGAGTCTGGACCGTGTCTCAGTTCCAGTGTGGCCGATCACCCTNTCAGGTCGCTACGCATCGTTGCCTTGGTAAGCCGTTACCTTACCAACTAGCTAATGCGGCGCGGATCCATCTATAAGTGAC
>n.538.6.bb_203
GTAGTTAGCCGTGGCTTTCTGATTAGGTACCGTCAAGGCGTGCATAGTTACTTACACATTTGTTCTTCCCTAATAACAGAGTTTTACGATCCGAAGACCTTCATCACTCACGCGGCGTTGCTCCGTCAGGCTTTCGCCCATTGCGGAAGATTCCCTACTGCTGCCTCCCGTAGGAGTCTGGACCGTGTCTCAGTTCCAGTGTGGCCGATCACCCTCTCAGGGTCGGCTACGCATCGTTGCCTTGGTAAGCCGTTACCTTACCAACTAGCTAATGCGGCGCGGATCCATCTATAAGTGACAGCAAAACCGTCTTTCACTATTGAACCATGCGGTTCAATATATTATCCGGTATTAG
>n.538.6.bb_204
GGAGTTAGCCGGTGCTTCTTCTGCGGGTAACGTCAATCGACAAGGTTATTAACCTTATCGCCTTCCTCCCCGCTGAAAGTACTTTACAACCCGAAGGCCTTCTTCATACACGCGGCATGGCTGCATCAGGCTTGCGCCCATTGTGCAATATTCCCCACTGCTGCCTCCCGTAGGAGTCTGGACCGTGTCTCAGTTCCAGTGTGGCTGGTCATCCTCTCAGACCAGCTAGGGATCGTCGCCTAGGTGAGCCGTTACCCACCTACTAGCTAATCCCATCTGGGCACATCTGATGGCAAGAGGCCCGAAGGTCCCCCTCTTTGGTCTTGCGACGTTATGCGGTATTAGCTACCGTTTCCAGTAGTATCCCCTCCATCAGGCAGTTTCCCCAGACCATTACCTCACCCCGTCCCGCCCACTCGTCACCCGAGAGCAAGCTCTCTGTGCTACCGTTCGACTTGCATGTGT
>n.538.6.bb_205
GGAGTTAGCCGGTGCTTCTTCTGCGGGTAACGTCAATCGACAAGGTTATTAACCTTATCGCCTTCCTCCCCGCTGAAAGTACTTTACAACCCGAAGGCCTTCTTCATACACGCGGCATGGCTGCATCAGGCTTGCGCCCATTGTGCAATATTCCCCACTGCTGCCTCCGTAGGAGTCTGGACCGTGTCTCAGTTCCAGTGTGGCTGGTCATCCTCTCAGACCAGCTAGGGATCGTCGCCTAGGTGAGCCGTTACCCACCTACTAGCTAATCCCATCTGGGCACATCTGA
>n.538.6.bb_206
GGAGTTAGCCGGTGCTTCTTCTGCGGGTAACGTCAATCGACAAGGTTATTAACCTTATCGCCTTCCTCCCCGCTGAAAGTACTTTACAACCCGAAGGCCTTCTTCATACACGCGGCATGGCTGCATCAGGCTTGCGCCCATTGTGCAATATTCCCCACTGCTGCCTCCCGTAGGAGTCTGGACCGTGTCTCAGTTCCAGTGTGGCTGGTCATCCTCTCAGACCAGCTAGGGATCGTCGCCTAGGTGAGCCGTTACCCACCTACTAGCTAATCCCATCTGGGCACATCTGATGGCAAGAGGCCCGAAGGTCCCCTCTTTGGTCTTGCGACGTTATGCGGTATTAGCTACCGTTT
>n.538.6.bb_207
GTAGTTAGCCGGTGCTTCTTCTGCAGGTACCGTCACCACAAGCTTCGCCCCTGCTGAAAGCGGTTTACAACCCGAAGGCCGTCATCCCGCACGCGGCGTTGCTGCATCAGGCTTCCGCCCATTGTGCAATATTCCCCACTGCTGCCTCCCGTAGGAGTCTGGGCCGTATCTCAGTCCCAATGTGGCCGGTCGCCCTCTCAGGCCGGCTACCCGGTCAAAGCCTTGGTAAGCCACTACCCCACCAACAAGCTGATAAGCCGCGAGTCCATCCAAAACCGCCG
>n.538.6.bb_208
GGAGTTAGCCGGTGCTTCTTCTGCGGGTAACGTCAATCGATGAGGTTATTAACCTTATCGCCTTCCTCCCCGCTGAAAGTACTTTACAACCCGAAGGCCTTCTTCATACACGCGGCATGGCTGCATCAGGCTTGCGCCCATTGTGCAATATTCCCCACTGCTGCCTCCCGTAGGAGTCTGGACCGTGTCTCAGTTCCAGTGTGGCTGGGTCATCCTCTCAGACCAGCTAGGGATCGTCGCCTAGGTGAGCCGTTACCCACCTACTAGCTAATCCCATCTGGGCACATCTGATGGCAAGAGGCCCGAAGGTCCCCCTCTTTGGTCTTGCGACGTTATGCGGTATT
>n.538.6.bb_209
GTAGTTAGCCGGTGCTTCTTCTGCAGGTACCGTCGCCACAAGCTTCGCCCCTGCTGAAAGCGGTTTACAACCCCGAAGGCCGTCATCCCGCACGCGGCGTTGCTGCATCAGGCTTCCGCCCATTGTGCAATATACCCCACTGCTGCCTCCCGTAGGAGTCTGGGCCGTATCTCAGTCCCAATGTGGCCGGTCGCCCTCTCAGGGCCGGCTACCCGTCAAAAGCCTTGGTAAGCCACTACCCCACCAACAAGCTGATAAGCCGCGAGTCCATCCAAAAACCGCGAAAGCTTTTCCAACCCCACCATGCAGCAAAGGATTCCTATCCGGTATTAGCCCAGTTTCCTGAAGTTATCCCG
>n.538.6.bb_210
GTAGTTAGCCGTGGCTTTCTGATTAGGTACCGTCAAGACGTGCATAGTTACTTACACATTTGTTCTTCCCTAATAACAGAGTTTTACGATCCGAAGACCTTCATCACTCACGCGGCGTTGCTCCGTCAGGCTTTCGCCCATTGCGGAAGATTCCCTACTGCTGCCTCCCGTAGGAGTCTGGACCGTGTCTCAGTTCCAGTGTGGCCGATCACCCTCTCAGGGTCGGCTACGCATCGTTGCCTTGGTAAGCCGTTACCTTACAACTAGCTAATGCGGCGCGGATCCATCTATAAGTGACAGCAAAACCGTCTTTCACTATTGAACCATGCGGTTCAATATATTATCCCGGTATTAGCTCC
>n.538.6.bb_211
GTAGTTAGCCGGTGCTTCTTCTGCAGGTACCGTCACCACAAGCTTCGCCCCTGCTGAAAGCGGTTTACAACCCGAAGGCCGTCATCCCGCACGCGGCGTTGCTGCATCAGGCTTCCGCCCATTGTGCAATATTCCCCACTGCTGCCTCCCGTAGGAGTCTGGGCCGTATCTCAGTCCCAATGTGGCCGGTCGCCCTCTCAGGCCGGCTACCCGTCAAAGCCTTGGTAAGCCACTACCCCACCAACAAGCTGATAAGCCGCGAGTCCATCCAAAACCGCCGAAGCTTTTCCAACCCCACCATGCAGCAAGGATTCCTATCCGGTATTAGCCCCAGTTTCCTGAAGTTATCCCGAAG
>n.538.6.bb_212
GGAGTTAGCCGGTGCTTCTTCTGCGGGTAACGTCAATTGCTGAGGTTATTAACCTCAACACCTTCCTCCCCGCTGAAAGTACTTTACAACCCGAGGCCTTCTTCATACACGCGGCATGGCTGCATCAGGCTTGCGCCCATTGTGCAATATTCCCCACTGCTGCCTCCCGTAGGAGTCTGGACCGTGTCTCAGTTCCAGTGTGGCTGGGTCATCCTCTCAGACCAGCTAGGGATCGTCGCCTAGGTGAGCCGTTACCCACCTACTAGCTAATCCCATCTGGGCACATCTGATGGCAAGAGG
>n.538.6.bb_213
GGAGTTAGCCGGTGCTTCTTCTGCGGGTAACGTCAATCGACAAGGTTATTAACCTTATCGCCTTCCTCCCCGCTGAAAGTACTTTACAACCCGAAGGCCTTCTTCATACACGCGGCATGGCTGCATCAGGCTTGCGCCCATTGTGCAATATTCCTCACTGCTGCCTCCCGTAGGAGTCTGGACCGTGTCTCAGTTCCAGTGTGGCTGGTCATCCTCTCAGACCAGCTAGGGATCGTCGCCTAGGTGAGCCGTTACCCACCTACTAGCTAATCCCATCTGGGCACATCTGATGGCAAGAGGCCCGAAGGTCCCCCTCTTTGGTCTTGCGACGTTATGCGGTATTAGCTACCGTTT
>n.538.6.bb_214
GTAGTTAGCCGGTGCTTCTTCTGCAGGTACCGTCACCACAAGCTTCGCCCCTGCTGAAAGCGGTTTACAACCCGAAGGCCGTCATCCCGCACGCGGCGTTGCTGCATCAGGCTTCCGCCCATTGTGCAATATTCCCCACTGCTGCCTCCCGTAGGAGTCTGGGCCGTATCTCAGTCCCAATGTGGCCGGTCGCCCTCTCAGGCCGGCTACCCGTCAAAGCCTTGGTAAGCCACTACCCCACCAACAAGCTGATAAGCCGCGAGTCCATCCAAAACCGCCG
>n.538.6.bb_215
GTAGTTAGCCGGTGCTTCTTCTGCAGGTACCGTCACCACAAGCTTCGCCCCTGCTGAAAGCGGTTTACAACCCGAAGGCCGTCATCCCGCACGCGGCGTGGCTGCATCAGGCTTCCGCCCATTGTGCAATATTCCCCACTGCTGCCTCCCGTAGGAGTCTGGGCCGTATCTCAGTCCCAATGTGGCCGGTCGCCCTCTCAGGCCGGCTACCCGTCAAAGCCTTGGTAAGCCACTACCCACCAACAAGCTGATAAGCCGCGAGTCCATCCAAAACCGCCGAAGCTTTTCCAACCCCACCATGCAGCAAGGATTCCTATCCGGTATTAGCCCCAGTTTCCTGAAGTTATCCCGAAGTCAAGGGCAGGTTACTCACGTGTTACTCACCCG
>n.538.6.bb_216
CGTAGTTAGCCGTGGCTTTCTGATTAGGTACCGTCAAGACGTGCATAGTTACTTACACATTTGTTCTTCCCTAATAACAGAGTTTTACGATCCGAAGACCTTCATCACTCACGCGGCGTTGCTCCGTCAGGCTTTCGCCCATTGCGGAAGATTCCCTACTGCTGCCTCCCGTAGGAGTCTGGACCGTGTCTCAGTTCCAGTGTGGCCGATCACCCTCTCAGGGTCGGCTACGCATCGTTGCCTTGGTAAGCCGTTACCTTACACTAGCTAATGCGGCGCGGATCCATCTATAAGTGACAGCAAAACCGTCTTTCACTATTGAACCATGCGGTTCAATATATTAT
>n.538.6.bb_217
GTAGTTAGCCGGTGCTTCTTCTGCAGGTACCGTCACCACAAGCTTCGCCCCTGCTGAAAGCGGTTTACAACCCGAAGGCCGCCATCCCGCACGCGGCGTTGCTGCATCAGGCTTCCGCCCATTGTGCAATATTCCCCACTGCTGCCTCCCGTAGGAGTCTGGGCCGTATCTCAGTCCCAATGTGGCCGGTCGCCCTCTCAGGCCGGCTAC
>n.538.6.bb_218
GGAGTTAGCCGGTGCTTCTTCTGCGGGTAACGTCAATTGCTGAGGTTATTAACCTCAACACCTTCCTCCCCGCTGAAAGTACTTTACAACCCGAAGGCCTTCTTCATACACGCGGCATGGCTGCATCAGGCTTGCGCCCATTGTGCAATATTCCCCACTGCTGCCTCCCGTAGGAGTCTGGACCGTGTCTCAGTTCCAGTGTGG
>n.538.6.bb_219
GTAGTTAGCCGTGGCTTTCTGATTAGGTACCGTCAAGACGTGCATAGTTACTTACACATTTGTTCTTCCCTAATAACAGAGTTTTACGATCCGAAGACCTTCATCACTCACGCGGCGTTGCTCCGTCAGGCTTTCGCCCATTGCGGAAGATTCCCTACTGCTGCCTCCCGTAGGAGTCTGGACCGTGTCTCAGTTCCAGTGTGGCCGATCACCCTCTCAGGGTCGGCTACGCATCGTTGCCTTGGTAAGCCGTTACCTTACCAACTAGCTAATGCGGCGCGGATCCATCTATAAGTGACAGCAAAACCGTCTTTCACTATTGAACCATGCGGTTCAATATATTATCCGGTATT
>n.538.6.bb_220
GTAGTTAGCCGTGGCTTTCTGATTAGGTACCGTCAAGACGTGCACAGTTACTTACACGTTTGTTCTTCCCTAATAACAGAGTTTTACGATCCTAAGACCTTCATCACTCACGCGGCGTTGCTCCGTCAGGCTTTCGCCCATTGCGGAAGATTCCCTACTGCTGCCCTCCCGTAGGAGTCTGGACCGTGTCTCAGTTCCAGTGTGGCCGATCACCCTNTCAGGGTCGGCTACGTATCGTTGCCTTGGNGAGCCGTTACCTCACCAACTAGCTAATACGGCG
>n.538.6.bb_221
GTAGTTAGCCGTGGCTTTCTGATTAGGTACCGTCAAGACGTGCATAGTTACTTACACATTTGTTCTTCCCTAATAACAGAGTTTTACGATCCGAAGACCTTCATCACTCACGCGGCGTTGCTCCGTCAGGCTTTCGCCCATTGCGGAAGATTCCCTACTGCTGCCTCCCGTAGGAGTCTGGACCGTGTCTCAGTTCCAGTGTGGCCGATCACCCTCTCAGGGTCGGCTACGCATCGTTGCCTTGGTAAGCCGTTACCTTACCAACTAGCTAATGCGGCGCGGATCCATCTATAAGTGACAGCAAAACCGTCTTTCACTATTGAACCATGCGGTTCAATATATTATCCGGTATTAGCTCCGGTTTCCCGAAGTTATCCCAGTCTTATAGGTAGGTTATCCCACCGTGTTACTCACCCCGTCCCGCCCGCTAACGTCAGAGAAGCAAGCCTCCCTCGTCTGTTCGCTCGACTTGCATGTAT
>n.538.6.bb_222
GTAGTTAGCCGTGGCTTTCTGATTAGGTACCGTCAAGACGTGCATAGTTACTTACACATTTATTCTTCCCTAATAACAGAGTTTTACGATCCGAAGACCTTCATCACTCACGCGGCGTTGCTCCGTCAGGCTTTCGCCCATTGCGGAAGATTCCCTACTGCTGCCTCCCGTAGGAGTCTGGACCGTGTCTCAGTTCCAGTGTGGCCGATCACCCTCTCAGGGTCGGCTACGCATCGTTGCCTTGGTAAGCCGTTACCTTACCAACTAGCTAATGCGGCGCGGATCCATCTATAAGTGACAGCAAAACCGTCTTTCACTATTGAACCATGCGGTTCAATATATTATCCGGTATTAG
>n.538.6.bb_223
GGAGTTAGCCGGTGCTTCTTCTGCGGGTAACGTCAATCGACAAGGTTATTAACCTTATCGCCTTCCTCCCCGCTGAAAGTACTTTACAACCCGAAGGCCTTCTTCATACACGCGGCATGGCTGCATCAGGCTTGCGCCCATTGTGCAATATTCCCCACTGCTGCCTCCGTAGGAGTCTGGACCGTGTCTCAGTTCCAGTGTGGCTGGTCATCCTCTCAGACCAGCTAGGGATCGTCGCCTAGGTGAGCCGTTACCCACCTACTAGCTAATCCCATCTGGGCACATCTGATG
>n.538.6.bb_224
GAGTTAGCCGGTGCTTCTTCTGCGGGTAACGTCAATCGACAAGGTTATTAACCTTATCGCCTTCCTCCCCGCTGAAAGTACTTTACAACCCGAAGGCCTTCTTCATACACGCGGCATGGCTGCATCAGGCTTGCGCCCATTGTGCAATATTCCCCACTGCTGCCTCCCGTAGGAGTCTGGACCGTGTCTCAGTTCCAGTGTGGCTGGTCATCCTCTCAGACCAGCTAGGGATCGTCGCCTAGGTGAGCCGTTACCCACCTACTAGCTAATCCCATCTGGGCACATCTGATGGCAAGAGGCCCGAAGGTCCCCCTCTTTGGTCTTGCGACGTTATGCGGTATTAGCTACCGTTTCCAGTAGT
>n.538.6.bb_225
GTAGTTAGCCGTGGCTTTCTGATTAGGTACCGTCAAGACGTGCATAGTTACTTACACATTTGTTCTTCCCTAATAACAGAGTTTTACGATCCGAAGACCTTCATCACTCACGCGGCGTTGCTCCGTCAGGCTTTCGCCCATTGCGGAAGATTCCCTACTGCTGCCTCCCGTAGGAGTCTGGACCGTGTCTCAGTTCCAGTGTGGCCGATCACCCTCTCAGGGTCGGCTACGCATCGTTGCCTTGGTAAGCCGTTACCTTACCAACTAGCTAATGCGACGCGGATCCATCTATAAGTGACAGCAAAACCGTCTTTCACTATTGAACCATGCGGTTCAATATATTATCCGGTATTAGCTCCGGTTTCCCGAAGTTATCCCAGTCTTATAGGTAGGTTATCCCACGTGTTAC
>n.538.6.bb_226
GTAGTTAGCCGTGGCTTTCTGATTAGGTACCGTCAAGACGTGCATAGTTACTTACACATTTGTTCTTCCCTAATAACAGAGTTTTACGATCCGAAGACCTTCATCACTCACGCGGCGTTGCTCCGTCAGGCTTTCGCCCATTGCGGAAGATTCCCTACTGCTGCCTCCCGTAGGAGTCTGGACCGTGTCTCAGTTCCAGTGTGGCCGATCACCCTCTCAGGGTCGGCTACGCATCGTTGCCTTGGTAAGCCGTTACCTTACCAACTAGCTAATGCGGCGCGGATCCATCTATAAGTGACAGCAAAACCGTCTTTCACTATTGAACCATGCGGTTCAATATATTATCCGGTATTAGCTCCGGTTTCCCGAAGTTATCCCAGTCTTATAGGTAGGTTATCCCACGTGTTACCTCACCCCGTCCCGCCCGCTAACGTCAGAGAAGCAAGCCTCCCTCGTCTGTTC
>n.538.6.bb_227
GTAGTTAGCCGTGGCTTTCTGATTAGGTACCGTCAAGACGTGCATAGTTACTTACACATTTGTTCTTCCCTAATAACAGAGTTTTACGATCCGAAGACCTTCATCACTCACGCGGCGTTGCTCCGTCAGGTCTTTCGCCCATTGCGGAAGATTCCCTACTGCTGCCTCCCGTAGGAGTCTGGGACCGTGTCTCAGTTCCAGTGTGGCCGATCCACCCTCTCGAGGGGTCGCTACGCATCGTTGCCTCGGTAAGCCGTTA
>n.538.6.bb_228
GAGTTAGCCGGTGCTTCTTCTGCGGGTAACGTCAATTGCTGAGGTTATTAACCTCAACACCTTCCTCCCGCTGAAGTACTTTACAACCCGAAGGCCTTCTTCATACACGCGGCATGGCTGCATCAGGCTTGCGCCCATTGTGCAATATTCCCCACTGCTGCCTCCGTAGGAGTCTGGACCGTGTCTCAGTTCCAGTGCGGCTGGTCATCCTCTCAGACCAGCTAGGGATCGTCGCCTAGGTGAGCCGTTACCCACCTACTAGCTAATCCCAT
>n.538.6.bb_229
GTAGTTAGCCGTGGCTTTCTGATTAGGTACCGTCAAGACGTGCATAGTTACTTACACATTTGTTCTTCCCTAATAACAGAGTTTTACGATCCGAAGACCTTCATCACTCACGCGGCGTTGCTCCGTCAGGCTTTCGCCCATTGCGGAAGATTCCCTACTGCTGCCTCCCGTAGGAGTCTGGACTGTGTCTCAGTTCCAGTGTGGCCGATCACCCTCTCAGGTCGGCTACGCATCGTTGCCTTGGTAAGCCGTTACCTTACCAACTAGCTAATGCGGCGCGGATCCATCTATAAGTGACAGCAAAACCGTCTTTCACTATTGAACCATGCGGTTCAATATATTA
>n.538.6.bb_230
GTAGTTAGCCGTGGCTTTCTGATTAGGTACCGTCAAGACGTGCATAGTTACTTACACATTTGTTCTTCCCTAATAACAGAGTTTTACGATCCGAAGACCTTCATCACTCACGCGGCGTTGCTCCGTCAGGCTTTCGCCCATTGCGGAAGATTCCCTACTGCTGCCTCCCGTAGGAGTCTGGACCGTGTCTCAGTTCCAGTGTGGCCGATCACCCTCTCAGGGTCGGCTACGCATCGTTGCCTTGGTAAGCCGTTACCTTACCAACTAGCTAATGCGGCGCGGATCCATCTATAAGTGACAGCAAAACCGTCTTTCACTATTGAACCATGCGGTTCAATATATTATCCGGTATTAGCTCCGGTTTCCCGAAGTTATCCCAGTCTTATAGGTAGGTTATCCCACGTGTTACTCCACCCCGTCCCGCCCGCTAACGTCAGAGAAGCAAGCTCCCTCGTCTG
>n.538.6.bb_231
GGAGTTAGCCGGTGCTTCTTCTGCGGGTAACGTCAATCGACAAGGTTATTAACCTTATCGCCTTCCTCCCCGCTGAAAGTACTTTACAACCCGAAGGCCTTCTTCATACACGCGGCATGGCTGCATCAGGCTTGCGCCCATTGTGCAATATTCCCCACTGCTGCCTCCCGTAGGAGTCTGGACCGTGTCTCAGTTCCAGTGTGGCTGGTCATCCTCTCAGACCAGCTAGGGATCGTCGCCTAGGTGAGCCGTTACCCACCTACTAGCTAATCCCATCTGGGCACATCTGATGGC
>n.538.6.bb_232
TAGTTAGCCGTGGCTTTCTGATTAGGTACCGTCAAGACGTGCATAGTTACTTACACATTTGTTCTTCCCTAATAACAGAGTTTTACGATCCGAAGACCTTCATCACTCACGCGGCGTTGCTCCGTCAGGCTTTCGCCCATTGCGGAAGATTCCCTACTGCTGCCTCCCGTAGGAGTCTGGACCGTGTCTCAGTTCCAGTGTGGCCGATCACCCTCTCAGGGTCGGGCTACGCATCGTCGCCTTGGTAAGCCGTTACCTTACCAACTAGCTAATGCGGCGCGGATCCATCTATAAGTGACAGCAAAAACCGTCTTTCACTATTGAACCATGCGGTTCAATATATTATCCCGGTATTAGCTCCGGTTTCCCCGAAGTTATCCCAGTCTTATAGG
>n.538.6.bb_233
GTAGTTAGCCGGTGCTTCTTCTGCAGGTACCGTCACCACAAGCTTCGCCCCTGCTGAAAGCGGTTTACAACCCGAAGGCCGTCATCCCGCACGCGGCGTTGCTGCATCAGGCTTCCGCCCATTGTGCAATATTCCCCACTGCTGCCTCCCGTAGGAGTCTGGGCCGTATCTCAGTCCCAATGTGGCCGGTCGCCCTCTCAGGCCGGCTACCCGGTCAAAGGCCTTGGTAAGCCACTACCCCACCAACAAGCTGATAAGCCGCGAGTCCATCCAAAACCGC
>n.538.6.bb_234
GTAGTTAGCCGTGGCTTTCTGATTAGGTACCGTCAAGACGTGCATAGTTACTTACACATTTGTTCTTCCCTAATAACAGAGTTTTACGATCCGAAGACCTTCATCACTCACGCGGCGTTGCTCCGTCAGGCTTTCGCCCATTGCGGAAGATTCCCTACTGCTGCCTCCCGTAGGAGTCTGGACCGTGTCTCAGTTCCAGTGTGGCTGGTCATCCTCTCAGACCAGCTAGGGATCGTCGCCTAGGCGAGCCGTTACCCACCTACTAGCTAATCCCATCTGGGCACATCTGATGGCAAGAGGCCGAAGGTCCCCTCTTTGGTCTTGCGACGTTATGCGGTATT
>n.538.6.bb_235
GTAGTTAGCCGTGGCTTTCTGATTAGGTACCGTCAAGACGTGCATAGTTACTTACACATTTATTCTTCCCTAATAACAGAGTTTTACGATCCGAAGACCTTCATCACTCACGCGGCGTTGCTCCGTCAGGCTTTCGCCCATTGCGGAAGATTCCCTACTGCTGCCTCCCGTAGGAGTCTGGACCGTGTCTCAGTTCCAGTGTGGCCGATCACCCTCTCAGGGTCGGCTACGCATCGTTGCCTTGGTAAGCCGTTACCTTACCAACTAGCTAATGCGGCGCGGATCCATCTATAAGTGACAGCAAAACCGTCTTTCACTATTGAACCAT
>n.538.6.bb_236
GTAGTTAGCCGGTGCTTCTTCTGCAGGTACCGTCACCACAAGCTTCGCCCCTGCTGAAAGCGGTTTACAACCCGAAGGCCGTCATCCCGCACGCGGCGTTGCTGCATCAGGCTTCCGCCCATTGTGCAATATTCCCCACTGCTGCCTCCCGTAGGAGTCTGGGCCGTATCTCAATCCCAATGTGGCCGGTCGCCCTCTCAGGCCGGCTACCCGTCAAAGCCTTGGTAAGCCACTACCCCACCAACAAGCTGATAAGCCGCGAGTCCATCCAAAACCGCCG
>n.538.6.bb_237
GTAGTTAGCCGTGGCTTTCTGATTAGGTACCGTCAAGACGTGCATAGTTACTTACACATTTGTTCTTCCCTAATAACAGAGTTTTACGATCCGAAGACCTTCATCACTCACGCGGCGTTGCTCCGTCAGGCTTTCGCCCATTGCGGAAGATTCCCTACTGCTGCCTCCCGTAGGAGTCTGGACCGTGTCTCAGTTCCAGTGTGGCTGGTCATCCTCTCAGACCAGCTAGGGATCGTCGCCTAGGTGAGCCGTTACCCACCTACTAGCTAATCCCATCTGGGCACATCTGA
>n.538.6.bb_238
GTAGTTAGCCGTGGCTTTCTGATTAGGTACCGTCAAGACGTGCATAGTTACTTACACATTTGTTCTTCCCTAATAACAGAGTTTTACGATCCGAAGACCTTCATCACTCACGCGGCGTTGCTCCGTCAGGCTTTCGCCCATTGCGGAAGATTCCCTACTGCTGCCTCCCGTAGGAGTCTGGACCGTGTCTCAGTTCCAGTGTGGCCGATCACCCTCTCAGGGTCGGCTACGCATCGTTGCCTTGGTAAGCCGTTACCTTACCAACTAGCTAATGCGGCGCGGATCCATCCATAAGTGACAGCAAAACCGTCTTTCACTATTGAACCATGCGGTTCAATATATTATCCGGCATTAGCTCCGGTTTCCCGAAGTTATCCCAGTCTTATAGGTAGGTTATCCCACGTGT
>n.538.6.bb_239
GTAGTTAGCCGTGGCTTTCTGATTAGGTACCGTCAAGACGTGCATAGTTACTTACACATTTGTTCTTCCCTAATAACAGAGTTTTACGATCCGAAGACCTTCATCACTCACGCGGCGTTGCTCCGTCAGGCTTTCGCCCATTGCGGAAGATTCCCTACTGCTGCCTCCCGTAGGAGTCTGGACCGTGTCTCAGTTCCAGTGTGGCCGATCACCCTCTCAGGGTCGGCTACGCATCGTTGCCTTGGTAAGCCGTTACCTTACCAACTAGCTAATGCGGCGCGGATCCATCTATAAGTGACAGCAAAACCGTCTTTCACTATTGAACCATGCGGTTCAATATATTATCCGGTATTAGCTCCGGT
>n.538.6.bb_240
GTAGTTAGCCGTGGCTTTCTGATTAGGTACCGTCAAGACGTGCATAGTTACTTACACATTTGTTCTTCCCTAATAACAGAGTTTTACGATCCGAAGACCTTCATCACTCACGCGGCGTTGCTCCGTCAGGCTTTCGCCCATTGCGGAAGATTCCCTACTGCTGCCTCCCGTAGGAGTCTGGACCGTGTCTCAGTTCCAGTGTGGCCGATCACCCTCTCAGGGTCGGCTACGCATCGTTGCCTTGGTAAGCCGTTACCTTACCAACTAGCTAATGCGGCGCGGATCCATCTATAAGTGACAGCAAAAACCGTCTTTCACTATTTGAACCATGCGGTTCAATATATTATCCGGTATTAGCTCCAGTTTCCCGAAGTTATCCCAGTCTTATAGGTAGGTTATCCCACGTGTTACTCACCCCGT
>n.538.6.bb_241
GGAGTTAGCCGGTGCTTCTTCTGCGGGTAACGTCAATCGACAAGGTTATTAACCTTATCGCCTTCCTCCCCGCTGAAAGTACTTTACAACCCGAAGGCCTTCTTCATACACGCGGCATGGCTGCATCAGGCTTGCGCCCATTGTGCAATATTCCCCACTGCTGCCTCCCGTAGGAGTCTGGGCCGTATCTCAGTCCCAATGTGGCCGGTCGCCCTCTCAGGGCCGGCTACCCGTCAAAGCCTTGGTAAGCCACTACCCCACCAACAAGCTGATGAGCCGCGAGTCCATCCAAAACCGCCGAAGCTTTCCAACCCCACCATGCAGCAAGGATTCCTATCCGGTAT
>n.538.6.bb_242
GTAGTTATCCGTGGCTTTCTGATTAGGTACCGTCAAGACGTGCATAGTTACTTACACATTTGTTCTTCCCTAATAACAGAGTTTTACGATCCGAAGACCTTCATCACTCACGCGGCGTTGCTCCGTCAGGCTTTCGCCCATTGCGGAAGATTCCCTACTGCTGCCTCCCGTAGGGGTCTGGACCGTGTCTCAGTTCCAGTGTGGCCGATCACCCTCTCAGGGTCGGGCTACGCATCGTTGCCTTGGTAAGCCGTTACCTTACCAACTAGCTAATGCGGCGCGGATCCATCTATAAGTGACA
>n.538.6.bb_243
GTAGTTAGCCGTGGCTTTCTGATTAGGTACCGTCAAGACGTGCATAGTTACTTACACATTTGTTCTTCCCTAATAACAGAGTTTTACGATCCGAAGACCTTCATCACTCACGCGGCGTTGCTCCGTCAGGCTTTCGCCCATTGCGGAAGATTCCCTACTGCTGCCTCCCGTAGGAGTCTGGACCGTGTCTCAGTTCCAGTGTGGCCGATCACCCTCTCAGGGTCGGCTACGCATCGTTGCCTTGGTAAGCCGTTACCTTACCAACTAGCTAATGCGGCGCGGATCCATCTATAAGTGACAGCAAAACCGTCTTTCACTATTGAACCATGCGGTTCAATATATTATCCGGTATT
>n.538.6.bb_244
GTAGTTAGCCGGTGCTTCTTCTGCAGGTACCGTCACCACAAGCTTCGCCCCTGCTGAAAGCGGTTTACAACCCGAAGGCCGTCATCCCGCACGCGGCGTTGCTGCATCAGGCTTCCGCCCATTGTGCAATATTCCCCACTGCTGCCTCCCGTAGGAGTCTGGGCCGTATCTCAGTCCCAATGTGGCCGGTCGCCCTCTCAGGCCGGCTACCCGTCAAAGCCTTGGTAAGCCACTACCCCACCAACAAGCTGATAAGCCGCGAGTCCATCCAAAACCGCCGAAGCTTTCCAACCCCCACCATGCAGCAAGGATTCCTATCCGGTATTAG
>n.538.6.bb_245
GTGGTTAGCGTGGCTTCTCGAGTTAGTACCGTACAAGACGTGCAGTAGGTTACTTACGACAAGTTTGTTCTTCCCTAATAACAGAGTTTTACGATCCGAAGACCTTCATCACTCACGCGGCGTTGCTCCGTCAGGCTTTCGCCCATTGCGGAAGATTCCCTACTGCTGCCTCCCGTAGGAGTCTGGACCGTGTCTCAGTTCCAGTGTGGCCGATCACCCT
>n.538.6.bb_246
GTAGTTAGCCGGTGCTTCTTCTGCAGGTACCGTCACCACAAGCTTCGCCCCTGCTGAAAGCGGTTTACAACCCGAAGGCCGTCATCCCGCACGCGGCGTTGCTGCATCAGGCTTCCGCCCATTGTGCAATATTCCCCACTGCTGCCTCCCGTAGGAGTCTGGGCCGTATCTCAGTCCCAATGTGGCCGGTCGCCCTCTCAGGCCGGCTACCCGTCAAAGCCTTGGTAAGCCACTACCCCACCAACAAGCTGATAAGCCGCGAGTCCATCCAAAACCGCCGAAGCTTTTCCAACCCCCACCATGCAGCAAGGATTCCTATCCGGTATTAGCCCCAGTTTCCTGAAGT
>n.538.6.bb_247
GTAGTTAGCCGTGGCTTTCTGATTAGGTACCGTCAAGACGTGCATAGTTACTTACACATTTGTTCTTCCCTAATAACAGAGTTTTACGATCCGAAGACCTTCATCACTCACGCGGCGTTGCTCCGTCAGGCTTTCGCCCATTGCGGAAGATTCCCTACTGCTGCCTCCCGTAGGAGTCTGGACCGTGTCTCAGTTCCAGTGTGGCCGATCACCCTCTCAGGGTCGCTACGCATCGTTGCCTTGGTAAGCCGTTACCTTACCACTAGCTAATGCGGCG
>n.538.6.bb_248
GTAGTTAGCCGTGGCTTTCTGATTAGGTACCGTCAAGACGTGCATAGTTACTTACACATTTGTTCTTCCCTAATAACAGAGTTTTACGATCCGAAGACCTTCATCACTCACGCGGCGTTGCTCCGTCAGGCTTTCGCCCATTGCGGAAGATTCCCTACTGCTGCCTCCCGTAGGAGTCTGGACCGTGTCTCAGTTCCAGTGTGGCCGATCACCCTCTCAGGGTCGGCTACGCATCGTTGCCTTGGTAAGCCGTTACCTTACCAACTAGCTAATGCGGCGCGGATCCATCTATAAGTGACAGCAAAACCGTCTTTCACTATTGAACCATGCGGTTCAATATATTATCCGGTATTAGCT
>n.538.6.bb_249
GTAGTTAGCCGTGGCTTTCTGATTAGGTACCGTCAAGACGTGCATAGTTACTTACACATTTGTTCTTCCCTAATAACAGAGTTTTACGATCCGAAGACCTTCATCACTCACGCGGCGTTGCTCCGTCAGGCTTTCGCCCATTGCGGAAGATTCCCTACTGCTGCCTCCCGTAGGAGTCTGGACCGTGTCTCAGTTCCAGTGTGGCCGATCACCCTCTTAGGGTCGGCTACGCATCGTTGCCTTGGTAAGCCGTTACCTTACCAACTAGCTAATGCGGCGCGGATCCATCTATAAAGTGACAGCAAAAACCGTCTTTCACTATTGAACCATGCGGTTCAATATATTATCCGGTATTAGCTCC
>n.538.6.bb_250
GTAGTTAACCGTGGCTTTCTGATTAGGTACCGTCAAGACGTGCATAGTTACTTACACATTTGTTCTTCCCTAATAACAGAGTTTTACGATCCGAAGACCTTCATCACTCACGCGGCGTTGCTCCGTCAGGCTTTCGCCCATTGCGGAAGATTCCCTACTGCTGCCTCCCGTAGGAGTCTGGACCGTGTCTCAGTTCCAGTGTGGCCGATCACCCTCTCAGGGTCGGCTACGCATCGTTGCCTTGGTAAGCCGTTACCTTACCAACTAGCTAATGCGGCGCGGATCCATCTATAAGTGACAGCAAAACCGTCTTTCACTATTGAACCATGCGGTTCAATATAT
>n.538.6.bb_251
GTAGTTAGCCGTGGCTTTCTGATTAGGTACCGTCAAGACGTGCATAGTTACTTACACATTTGTTCTTCCCTAATAACAGAGTTTTACGATCCGAAGACCTTCATCACTCACGCGGCGTTGCTCCGTCAGGCTTTCGCTCATTGCGGAAGATTCCCTACTGCTGCCTCCCGTAGGAGTCTGGACCGTGTCTCAGTTCCAGTGTGGCCGATCACCCTCTCAGGTCGGCTACGCATCGTTGCCTTGGTAAGCCGTTACCTTACCAACTAGCTAATGCGGCGCGGATCCATCTATAAGTGACAGCAAAACCGTCTTTCACTATTGAACCATGCGGTTCAATATATTATCCGGTATTAGCTCCGGTTTCCCGAAGTTATCCCAGTCTTATAGGTAGGCCTATCCCACGTGTTACCTCA
>n.538.6.bb_252
GTAGTTAGCCGTGGCTTTCTGATTAGGTACCGTCAAGACGTGCATAGTTACTTACACATTTGTTCTTCCCTAATAACAGAGTTTTACGATCCGAAGACCTTCATCACTCACGCGGCGTTGCTCCGTCAGGCTTTCGCCCATTGCGGAAGATTCCCTACTGCTGCCTCCCGTAGGAGTCTGGACCGTGTCTCAGTTCCAGTGTGGTCGATCACCCTCTCAGGGTCGGCTACGCATCGTTGCCTTGGTAAGCCGTTACCTTACCAACTAGCTAATGCGGCGCGGATCCATCTATAAGTGACAGCAAAACCGTCTTTCACTATTGAGCCATGCGGTTCAATATATTATCCGGTA
>n.538.6.bb_253
GTAGTTAGCCGTGGCTTTCTGATTAGGTACCGTCAAGACGTGCATAGTTACTTACACACTTTGTTCTTCCCTAATAACAGAGTTTTACGATCCGAAGACCTTCATCACTCACGCGGCGTTGCTCCGTCAGGCTTTCGCCCATTGCGGAAGATTCCCTACTGCTGCCTCCCGTAGGAGTCTGGACCGTGTCTCAGTTCCAGTGTGGCCGATCACCCTCTCAGGGTCGGCTACGCATCGTTGCCTTGGTAAGCCGTTACCTTACCAACTAGCTAATGCGGCGCGGATCCATCTAT
>n.538.6.bb_254
GTAGTTAGCCGTGGCTTTCTGATTAGGTACCGTCAAGACGTGCATAGTTACTTACACATTTGTTCTTCCCTAATAACAGAGTTTTACGATCCGAAGACCTTCATCACTCACGCGGCGTTGCTCCGTCAGGCTTTCGCCCATTGCGGAAGATTCCCTACTGCTGCCTCCCGTAGGAGTCTGGACCGTGTCTCAGTTCCAGTGTGGCCGATCACCCTCTCAGGGTCGGCTACGCATCGTTGCCTTGGTAAGCCGTTACCTTACCAACTAGCTAATGCGGCGCGGATCCATCTATAAGTGACAGCAAAACCGTCTTTCACTATTGAACCATGCGGTTCAATATATTATCCGGTATTA
>n.538.6.bb_255
GTAGTTAGCCGGTGCTTCTTCTGCAGGTACCGTCACCACAAGCTTCGCCCCTGCTGAAAGCGGTTTACAACCCGAAGGCCGTCATCCCGCACGCGGCGTTGCTGCATCAGGCTTCCGCCCATTGTGCAATATTCCCCACTGCTGCCTCCCGTAGGAGTCTGGGCCGTATCTCAGTCCCAATGTGGCCGGTCGCCCTCTCAGGCCGGCTACCCGTCAAAGCCTTGGTAAGCCACTACCCACCAACAAGCTGATAAGCCGTGAGTCCATCCAAAACCGCCGAAGCTTTTCCAACCCCACCATGCAGCAAGGATTCCTATCCGGTATTAGCCCCAGTTTCCTGAAGTTATCCCGAA
>n.538.6.bb_256
GGGGTTAGCCGGTGCTTCTTCTGCGGGTAACGTCAATCGACAAGGTTATTAACCTTATCGCCTTCCTCCCCGCTGAAAGTACTTTACAACCCGAAGGCCTTCTTCATACACGCGGCATGGCTGCATCAGGCTTGCGCCCATTGTGCAATATTCCCCACTGCTGCCTCCCGTAGGAGTCTGGACCGTGTCTCAGTTCCAGTGTGGCTGGTCATCCTCTCAGACCCAGCTAGGGGATCGTCGCCTAGGTGAGCCGTTACCCACCTACTAGCTAATCCCATCTGGGCACATCTGATGGCA
>n.538.6.bb_257
GTAGTTAGCCGGTGCTTCTTCTGCAGGTACCGTCACCACAAGCTTCGCCCCTGCTGAAAGCGGTTTACAACTCGAAGGCCGTCATCCCGCACGCGGCGTTGCTGCATCAGGCTTCCGCCCATTGTGCAATATTCCCCATGCTGCCTCCCGTAGGAGTCTGGACCGTGTCTCAGTTCCAGTGTGGCCGATCCACCCTCTCGAGGGTCGGCTACGCATCGTTGCCTTGGTAAGCCGTTACCTTACAACTAGCTAATGCGGCGCGGAT
>n.538.6.bb_258
GTAGTTAGCCGTGGCTTTCTGATTAGGTACCGTCAAGAACGTGCATAGTTACTTACGACATTTGTTCTTCCCTAATAACAGAGTTTTACGATCCGAAGACCTTCATCACTCACGCGGCGTTGCTCCGTCAGGCTTTCGCCCATTGCGGAAGATTCCCTACTGCTGCCTCCCGTAGGAGTCTGGACCGTGTCTCAGTTCCAGTGTGGCCGATCACCCTCTCAGGGTCGGCTACGCATCGTCGCCTTGGTAAGCCGTTACCTTACCAACTAGCTAATGCGGCGCGGATCCATCTATAAGTGACA
>n.538.6.bb_259
GTAGTTAGCCGTGGCTTTCTGATTAGGTACCGTCAAGACGTGCATAGTTACTTACACATTTATTCTTCCCTAATAACAGAGTTTTACGATCCGAAGACCTTCATCACTCACGCGGCGTTGCTCCGTCAGGCTTTCGCCCATTGCGGAAGATTCCCTACTGCTGCCTCCCGTAGGAGTCTGGACCGTGTCTCAGTTCCAGTGTGGCCGATCACCCTCTCAGGGTCGGCTACGCATCGTTGCCTTGGTAAGCCGTTACCTTACCAACTAGCTAATGCGGCGCGGATCCATCTATAAGTGACAGCAAAACCGTCTTTCACTATTGAACCATGCGGTTCAATATATT
>n.538.6.bb_260
GTAGTTAGCCGGTGCTTCTTCTGCAGGTACCGTCACCACAAGCTTCGCCCCTGCTGAAAGCGGTTTACAACCCGAAGGCCGTCATCCCGCACGCGGCGTTGCTGCATCAGGCTTCCGCCCATTGTGCAATATTCCCCACTGCTGCCTCCCGTAGGAGTCTGGGCCGTATCTCAGTCCCAATGTGGCCGGTCGCCCTCTCAGGCCGGCTACCCGTCAAAGCCTTGGTAAGCCACTACCCACCAACAAGCTGATAAGCCGCGAGTCCATCC
>n.538.6.bb_261
GGAGTTAGCCGGTGCTTCTTCTGCGGGTAACGTCAATCGATGAGGTTATTAACCTCACCGCCTTCCTCCCCGCTGAAAGTGCTTTACAACCCGAAGGCCTTCTTCACACACGCGGCATGGCTGCATCAGGCTTGCGCCCATTGTGCAATATTCCCCACTGCTGCCTCCCGTAGGAGTCTGGACCGTGTCTCAGTTCCAGTGTGGCTGGGTCATCCTCTCAGACCAGCTAGGGATCGTCGCCTAGGTGAGCCGTTACCCACCTACCAGCTAATCCCATCTGGGCACATCTGATGGCATGAGGCCCGAAGGTCCCCACTTTGGTCTTGCGACGTTATGCGGTATTAGCTACCGTTTCCAGTAGTTATCCCCT
>n.538.6.bb_262
GTAGTTAGCCGTGGCTTTCTGATTAGGTACCGTCAAGACGTGCATAGTTACTTACACATTTGTTCTTCCCTAATAACAGAGTTTTACGATCCGAAGACCTTCATCACTCACGCGGCGTTGCTCCGTCAGGCTTTCGCCCATTGCGGAAGATTCCCTACTGCTGCCTCCCGTAGGAGTCTGGACCGTGTCTCAGTTCCAGTGTGGCCGATCACCCTCTCAGGGTCGGCTACGCATCGTTGCCTTGGTAAGCCGTTACCTTACCAACTAGCTAATGCGGCGCGGATCCATCTATAAGTGACAGCAAAACCGTCTTTCACTACTGGACCATGCGGTTCAAT
>n.538.6.bb_263
GTAGTTAGCCGTGGCTTTCTGATTAGGTACCGTCAAGACGTGCATAGTTACTTACACATTTGTTCTTCCCTAATAACAGAGTTTTACGATCCGAAGACCTTCATCACTCACGCGGCGTTGCTCCGTCAGGCTTTCGCCCATTGCGGAAGATTCCCTACTGCTGCCTCCCGTAGGAGTCTGGACCGTGTCTCAGTTCCAGTGTGGCCGATCACCCTNTCAGGGTCGGCTACGCATCGTTGCCTTGGTAAGCCGTTACCTTACCAACTAGCTAAATGCGGCGCGGATCCATCTATAAGTGACAGCAAAACCGTCTTTCACTATTGAACCATGCGGTTCAATATATTATCCGGTATT
>n.538.6.bb_264
GTAGTTAGCCGGTGCTTCTTCTGCAGGTACCGTCACCACAAGCTTCGCCCCTGCTGAAAGCGGTTTACAACCCGAAGGCCGTCATCCCGCACGCGGCGTTGCTGCATCAGGCTTCCGCCCATTGTGCAATATTCCCCACTGCTGCCTCCCGTAGGAGTCTGGGCCGTATCTCAGTCCCAATGTGGCCGGTCGCCCTCTCAGGCCGGCTACCCGTCAAAGCCTTGGTAAGCCACTACCCCACCAACAAGCTGATAAGCCGCGAGTCCATCCAAAACCGCCGAAGC
>n.538.6.bb_265
GGAGTTAGCCGGTGCTTCTTCTGCGGGTAACGTCAATCGACAAGGTTATTAACCTTATCGCCTTCCTCCCCGCTGAAAGTACTTTACAACCCGAAGGCCTTCTTCATACACGCGGCATGGCTGCATCAGGCTTGCGCCCATTGTGCAATATTCCCCACTGCTGCCTCCCGTAGGAGTCTGGACCGTGTCTCAGTTCCAGTGTGGCTGGTCATCCTCTCAGACCAGCTAGGGATCGTCGCCTAGGTGAGCCGTTACCCACCTACTAGCTAATCCCATCTGGGCACATCTGATGGCAAGAGGCCCGAAGGTCCCCCTCTTTGGTCTTGCGACGTTATGCGGTAT
>n.538.6.bb_266
GGAGTTAGCCGGTGCTTCTTCTGCGGGTAACGTCAATCGATGAGGTTATTAACCTCACCGCCTTCCTCCCCGCTGAAAGTGCTTTACAACCCGAAGGCCTTCTTCACACACGCGGCATGGCTGCATCAGGCTTGCGCCCATTGTGCAATATTCCCCACTGCTGCCTCCCGTAGGAGTCTGGACCGTGTCTCAGTTCCAGTGTGGCTGGGTCATCCTCTCAGACCAGCTAGGGATCGTCGCCTAGGTGAGCCGTTACCCACCTACTAGCTAATCCCATCTGGGCACATCTGATGGCAT
>n.538.6.bb_267
GTAGTTAGCCGTGGCTTTCTGATTAGGTACCGTCAAGACGTGCATAGTTACTTACACATTTGTTCTTCCCTAATAACAGAGTTTTACGATCCGAAGACCTTCATCACTCACGCGGCGTTGCTCCGTCAGGCTTTCGCCCATTGCGGAAGATTCCCTACTGCTGCCTCCCGTAGGAGTCTGGACCGTGTCTCAGTTCCAGTGTGGCCGATCACCCTCTCAGGGTCGGCTACGCATCGTTGCCTTGGTAAGCCGTTACCTTACCAACTAGCTAATGCGGCGCGGATCCATCTATAAGTGACAGCAAAACCGTCTTTCACTATTGAACCATGCGGTTCAATATATTATCCGGTATT
>n.538.6.bb_268
GTAGTTAGCCGTGGCTTTCTGATTAGGTACCGTCAAGACGTGCATAGTTACTTACACATTTGTTCTTCCCTAATAACAGAGTTTTACGATCCGAAGACCTTCATCACTCACGCGGCGTTGCTCCGTCAGGCTTTCGCCCATTGCGGAAGATTCCCTACTGCTGCCTCCCGTAGGAGTCTGGACCGTGTCTCAGTTCCAGTGTGGCCGATCACCCTCTCAGGGTCGGCTACGCATCGTTGCCTTGGTAAGCCGTTACCTTACCAACTAGCTAATGCGGCGCGGATCCATCTATAAGTGACAGCAAAACCGTCTTTCACTATTGAACCATGCGGTTCAATATATTATCCGGTATTAGCTCCGGTTTCCCGAAGTTATCCCAGTCTTATAGGTAGGTTATCCCACGTGTTACCT
>n.538.6.bb_269
GTAGTTAGCCGTGGCTTTCTGATTAGGTACCGTCAAGACGTGCATAGTTACTTACACATTTGTTCTTCCCTAATAACAGAGTTTTACGATCCGAAGACCTTCATCACTCACGCGGCGTTGCTCCGTCAGGCTTTCGCCCATTGCGGAAGATTCCCTACTGCTGCCTCCCGTAGGAGTCTGGACCGTGTCTCAGTTCCAGTGTGGCCGATCACCCTCTCAGGGTCGGCTACGCATCGTTGCCTTGGTAAGCCGTTACCTTACCAACTAGCTAATGCGGCGCGGATCCATCTATAAGTGACAGCAAAACCGTCTTTCACTATTGAACCATGCGGTTCAATATATTATCCGGTATTAGCTCCGGTTTCCCGAAGTTATC
>n.538.6.bb_270
GTAGTTAGCCGGTGCTTCTTCTGCAGGTACCGTCACCACAAGCTTCGCCCCTGCTGAAAGCGGTTTACAACCCCGAAGGCCGTCATTCCCGCACGCGGCGTTGCTGCATCAGGCTTCCGCCCATTGTGCAATATTCCCCACTGCTGCCTCCCGTAGGAGTCTGGGCCGTATCTCAGTCCCAATGTGCCGGTCGCCCTCTCAGGCCGGCTACCCGTCAAAGCCTTGGTAAGCCACTACCCCACCAACAAGCTGATAAGCCGCGAGTCCATCCAAAACCG
>n.538.6.bb_271
GGAGTTAGTCGGTGCTTCTTCTGCGGGTAACGTCAATCGACAAGGTTATTAACCTTATCGCCTTCCTCCCCGCTGAAAGTACTTTACAACCCGAAGGCCTTCTTCATACACGCGGCATGGCTGCATCAGGCTTGCGCCCATTGTGCAATATTCCCCACTGCTGCCTCCGTAGGAGTCTGGACCGTGTCTCAGTTCCAGTGTGGCTGGTCATCCTCTCAGACCAGCTAGGGATCGTCGCCTAGGTGAGCCGTTACCCACCTACTAGCTAATCCCATCTGGGCACATCTGATGGCAAGAGGCCCGAAGGTCCCCCTCTTTGGTCTTGCGACGTTATGCGGTATTAGCTACCGTTTCCA
>n.538.6.bb_272
GAAGTTAGCCGGGGCTTCTTCTCCGGTTACCGTCATTATCTTCACCGGTGAAAGAGCTTTACAACCCTAGGGCCTTCATCACTCACGCGGCATGGCTGGATCAGGCTTGCGCCCATTGTCCAATATTCCCCACTGCTGCCTCCCGTAGGAGTCTGGGCCGTGTCTCAGTCCCAGTGTGGCTGATCACCCTCTCAGGCCGGCTACCCGTCAAAAGCCTTGGGTAAGCCCACTACCCCACAACAAGCTGATAAGCCGCGAGTCCATCCAAAACCGCCGAAGCTTTCCAACCCCCACCATGCAGCAAGGATTCCTATCCGGTATTAGCCCCAGTTTCC
>n.538.6.bb_273
GTAGTTAGCCGTGGCTTTCTGATTAGGTACCGTCAAGACGTGCATAGTTACTTACACATTTGTTCTTCCCTAATAACAGAGTTTTACGATCCGAAGACCTTCATCACTCACGCGGCGTTGCTCCGTCAGGCTTTCGCCCATTGCGGAAGATTCCCTACTGCTGCCTCCCGTAGGAGTCTGGACCGTGTCTCAGTTCCAGTGTGGCCGATCACCCTCTCAGGGTCGGCTACGCATCGTTGCCTTGGTAAGCCGTTACCTTACCAACTAGCTAATGCGGCGCGGATCCATCTATAAGTGACAGCAAAACCGTCTTTCACTATTGAACCATGCGGTTCAATATATTATCCGGTATT
>n.538.6.bb_274
GTAGTTAGCCGTGGCTTTCTGATTAGGTACCGTCAAGACGTGCATAGTTACTTACACATTTGTTCTTCCCTAATAACAGAGTTTTACGATCCGAAGACCTTCATCACTCACGCGGCGTTGCTCCGTCAGGCTTTCGCCCATTGCGGAAGATTCCCTACTGCTGCCTCCCGTAGGAGTCTGGACCGTGTCTCAGTTCCAGTGTGGCCGATCACCCTCTCAGGGTCGGCTACGCATCGTTGCCTTGGTAAGCCGTTACCTTACAACTAGCTAATGCGGCGCGGATCCATCTATAAGTGAC
>n.538.6.bb_275
GTAGTTAGCCGTGGCTTTCTGATTAGGTACCGTCAAGACGTGCATAGTTACTTACACATTTAGTTCTTCCCTAATAACAGAGTTTTACGATCCGAAGACCTTCATCACTCACGCGGCGTTGCTCCGTCAGGCTTTCGCCCATTGCGGAAGATTCCCTACTGCTGCCTCCCGTAGGAGTCTGGACCGTGTCTCAGTTCCAGTGTGGCCGATCACCCTCTCAGGGTCGGCTACGCATCGTTGCCTTGGTAAGCCGTTACCTTACCAACTAGCTAATGCGGCGCGGATCCATCTATAAGTGACAGCAAAACCGTCTTTCACTATTGAACCATGCGGTTCAATATA
>n.538.6.bb_276
GTAGTTAGCCGTGGCTTTCTGATTAGGTACCGTCAAGACGTGCATAGTTACTTACACATTTGTTCTTCCCTAATAACAGAGTTTTACGATCCGAAGACCTTCATCACTCACGCGGCGTTGCTCCGTCAGGCTTTCGCCCATTGCGGAAGATTCCCTACTGCTGCCTCCCGTAGGAGTCTGGACCGTGTCTCAGTTCCAGTGTGGCCGATCACCCTCTCAGGGTCGGCTACGCATCGTTGCCTTGGTAAGCCGTTACCTTACCAACTAGCTAATGCGGCGCGGATCCATCTATAAGTGACAGCAAAACCGTCTTTCACTATTGAACCATGC
>n.538.6.bb_277
GTAGTTAGCCGGTGCTTCTTCTGCAGGTACCGTCACCACAAGCTTCGCCCCTGCTGAAAGCGGTTTACAACCCGAAGGCCGTCATCCCGCACGCGGCGTTGCTGCATCAGGCTTCCGCCCATTGTGCAATATTCCCCACTGCTGCCTCCCGTAGGAGTCTGGGCCGTATCTCAGTCCCAATGTGGCCGGTCGCCCTCTCAGGCCGGCTACCCGTCAAAGCCTTGGTAAGCCACTACCCNCCAACAAGCTGATAAGCCGCGAGTCCATCCAAAACCGCCG
>n.538.6.bb_278
GTAGTTAGCCGGTGCTTCTTCTGCAGGTACCGTCACCACAAGCTTCGCCCCTGCTGAAAGCGGTTTACAACCCGAAGGCCGTCATCCCGCACGCGGCGTTGCTGCATCAGGCTTCCGCCCATTGTGCAATATTCCCCACTGCTGCCTCCCGTAGGAGTCTGGGCCGTATCTCAGTCCCAATGTGGCCGGTCGCCCTCTCAGGCCGGCTACCCGTCAAAGCCTTGGTAAGCCACTACCCACCAACAAGCTGATAAGCCGCGAGTCCATCC
>n.538.6.bb_279
GTAGTTAGCCGTGGCTTTCTGATTAGGTACCGTCAAGACGTGCATAGTTACTTACACATTTGTTCTTCCCTAATAACAGAGTTTTACGATCCGAAGACCTTCATCACTCACGCGGCGTTGCTCCGTCAGGCTTTCGCCCATTGCGGAAGATTCCCTACTGCTGCCTCCCGTAGGAGTCTGGACCGTGTCTCAGTTCCAGTGTGGCCGATCACCCTCTCAGGGTCGGCTACGCATCGTTGCCTTGGTAAGCCGTTACCTTACCAACTAGCTAATGCGGCGCGGATCCATCTATAAGTGACAGCAAAACCGTCTTTCACTATTGAACCATGCGGTTCAATATATTATCCGGTATTAGCTCCGGTTTCCGAAGTTATCCCAGTCTTATAGGTAGGTTATCCCACCGTGTTACTCACCCCGTCCCGCCCGCTAACGTCAGAGGAGCAAGCTCCCTCGTCTGTTC
>n.538.6.bb_280
GTAGTTAGCCGGTGCTTCTTCTGCAGGTACCGTCACCACAAGCTTCGCCCCTGCTGAAAGCGGTTTACAACCCGAAGGCCGTCATCCCGCACGCGGCGTTGCTGCATCAGGCTTCCGCCCATTGTGCAATATTCCCCACTGCTGCCTCCCGTAGGAGTCTGGGCCGTATCTCAGTCCCAATGTGGCCGGTCGCCCTCTCAGGCCGGCTACCCGGTCAAAGCCTTGGTAACGCCACTACCCACCAACAAGCTGATAAGCCGCGAGTCCATCCAAAACCGC
>n.538.6.bb_281
GTAGTTAGCCGTGGCTTTCTGATTAGGTACCGTCAAGACGTGCATAGTTACTTACACATTTGTTCTTCCCTAATAACAGAGTTTTACGATCCGAAGACCTTCATCACTCACGCGGCGTTGCTCCGTCAGGCTTTCGCCCATTGCGGAAGATTCCCTACTGCTGCCTCCCGTAGGAGTCTGGACCGTGTCTCAGTTCCAGTGTGGCCGATCACCCTCTCAGGGTCGGCTACGCATCGTGCCTTGGTAAGCCGTTACCTTACCAACTAG
>n.538.6.bb_282
GTAGTTAGCCGGTGCTTCTTCTGCAGGTACCGTCGCCACAAGCTTCGCCCCTGCTGAAAGCGGTTTACAACCCGAAGGCCGTCATCCCGCACGCGGCGTTGCTGCATCAGGCTTCCGCCCATTGTGCAATATTCCCCACTGCTGCCTCCCGTAGGAGTCTGGGCCGTATCTCAGTCCCAATGTGGCCGGTCGCCCTCTCAGGCCGGCTACCCGTCAAAGCCTTGGTAAGCCACTACCCCACCAACAAGCTGATAAGCCGCGAGTCCATCCAAAACCGCCGAAGCTTTTCCAACCCCACCATGCAGCAAGGATTCCTATCCGGTATTAGCCCCAGTTTCCTGAAGTTATCCCGAAGTCAAAGGGCAGGGTTACTCACGTGTTACTCACCCGTTCGCACTCGACGTA
>n.538.6.bb_283
GTAGTTAGCCGTGGCTTTCTGATTAGGTACCGTCAAGACGTGCATAGTTACTTACACATTTGTTCTTCCCTAATAACAGAGTTTTACGATCCGAAGACCTTCATCACTCACGCGGCGTTGCTCCGTCAGGCTTTCGCCCATTGCGGAAGACTTCCCTACTGCTGCCCTCCCGTAGGAGTCTGGACCGTGTCTCAGTTCCAGTGTGGCCGATCCACCCTCTCGAGGGTCGGCTACGCATCGTTGCCTTGGTAAGCCGTTACCTTACCAACTAGCTAATGCGGCGCGGATCCATCTATAAGTGACAGACAAAACCGTCTTTCACTA
>n.538.6.bb_284
GTAGTTAGCCGTGGCTTTCTATTAGGTACCGTCAGACGTGCATAGTTACTTACACATTTGTTTCTTCCCTAACTAACAGAGTTTTACGTATCCGAAGACCTTCATCACTCACGCGGCGTTGCTCCGTCAGGCTTTCGCCCATTGCGGAAGATTCCCTACTGCTGCCTCCCGTAGGAGTCTGGACCGTATCTCAGTCCCAATGTGGCCGGT
>n.538.6.bb_285
GTAGTTAGCCGTGGCTTTCTGATTAGGTACCGTCAAGACGTGCATAGTTACTTACACATTTGTTCTTCCCTAATAACAGAGTTTTACGATCCGAAGACCTTCATCACTCACGCGGCGTTGCTCCGTCAGGCTTTCGCCCATTGCGGAAGATTCCCTACTGCTGCCTCCCGTAGGAGTCTGGACCGTGTCTCAGTTCCAGTGTGGCCGATCACCCTCTCAGGGTCGGCTACGCATCGTCGCCTTGGTAAGCCGTTACCTTACCACTAGCTAATGCGGCGCGGATCCATCTATAAGTGACAGCAAAACCGTCTTTCACTATTGAACCATGCGGTTCAATATATTATCCGGTATT
>n.538.6.bb_286
GTAGTTAGCCGGTGCTTCTTCTGCAGGTACCGTCACCACAAGCTTCGCCCCTGCTGAAAGCGGTTTACAACCCGAAGGCCGTCATCCCGCACGCGGCGTTGCTGCATCAGGCTTCCGCCCATTGTGCAATATTCCCCACTGCTGCCTCCCGTAGGAGTCTGGGCCGTATCTCAGTCCCAATGTGGCCGGTCGCCCTCTCAGGCCGGCACCCGTCAAAGCCTTGGTAAGCCACTACCCACCAACAAGCTGATAAGCCGCGAGTCCATCCAAAACCGCCGAAGCTTTTCCAACCCCACCATGCAGCAAGGATTCCTATCCGGTATTAGCCCCAGTTTCCTGAAGTTATCCCGAAGTCAAGGGCAGGTTACTCACGTGTTACTCACCCGTTCGCCACT
>n.538.6.bb_287
GTAGTTAGCCGTGGCTTTCTGATTAGGTACCGTCAAGACGTGCATAGTTACTTACACATTTGTTCTTCCCTAATAACAGAGTTTTACGATCCGAAGACCTTCATCACTCACGCGGCGTTGCTCCGTCAGGCTTTCGCCCATTGCGGAAGATTCCCTACTGCTGCCTCCCGTAGGAGTCTGGACCGTGTCTCAGTTCCAGTGTGGCCGATCACCCTCTCAGGGTCGGCTACGCATCGTTGCCTTGGTAAGCCGTTACCTTACCAACTAGCTAATGCGGCGCGGATCCATCTATAAGTGACAGCAAAACCGTCTTTCACTATTGAACCATGCGGTTCAATATATTATCCGGTATTAGCTCCGGT
>n.538.6.bb_288
GTAGTTAGCCGTGGCTTTCTGATTAGGTACCGTCAAGACGTGCATAGTTACTTACACATTTGTTCTTCCCTAATAACAGAGTTTTACGGTCCGAAGACCTTCATCACTCACGCGGCGTTGCTCCGTCAGGCTTTCGCCCATCGCGGAAGATTCCCTACTGCTGCCTCCCGTAGGAGTCTGGACCGTGTCTCAGTTCCAGTGTGGCCGATCACCCTCTCAGGGTCGGCTACGCATCGTTGCCTTGGTAAGCCGTTACCTTACCAACTAGCTAATGCGGCGCGGATCCATCTATAAGTGACAGCAAAACCGTCTTTCACTATTGAACCATGCGGTTCAATATATTA
>n.538.6.bb_289
GTAGTTAGCCGGTGCTTCTTCTGCAGGTACCGTCACCACAAGCTTCGCCCCTGCTGAAAGCGGTTTACAACCCGAAGGCCGTCATCCCGCACGCGGCGTTGCTGCATCAGGCTTCCGCCCATTGTGCAATATTCCCCACTGCTGCCTCCCGTAGGAGTCTGGGCCGTATCTCAGTCCCAATGTGGCCGGTCGCCCTCTCAGGCCGGCTACCCGGTCAAAGCCTTGGTAAGCCACTACCCCACCAACAAGCTGATAAGCCGCGAGTCCATCCAAAACCG
>n.538.6.bb_290
GGAGTTAGCCGGTGCTTCTTCTGCGGGTAACGTCAATTGCTGAGGTTATTAACCTCAACACCTTCCTCCCCGCTGAAAGTACTTTACAACCCGAAGGCCTTCTTCATACACGCGGCATGGCTGCATCAGGCTTGCGCCCATTGTGCAATATTCCCCACTGCTGCCTCCCGTAGGAGTCTGGACCGTGTCTCAGTTCCAGTGTGGCTGGGTCATCCTCCCAGACCAGCTAGGGATCGTCGCCTAGGTGAGCCGTTACCCACCTACTAGCTAATCCCATCTGGGCACATCTGATGGCAAGAGGCCCGAAGGTCCCCTCTTTGGTCTTGCGACGTTATGCGGTATTAGCTACCGTTTCCAG
>n.538.6.bb_291
GTAGTTAGCCGTGGCTTTCTGATTAGGTACCGTCAAGACGTGCATAGTTACTTACACATTTGTTCTTCCCTAATAACAGAGTTTTACGATCCGAAGACCTTCATCACTCACGCGGCGTTGCTCCGTCAGGCTTTCGCCCATTGCGGAAGATTCCCTACTGCTGCCTCCCGTAGGAGTCTGGACCGTGTCTCAGTTCCAGTGTGGCCGATCACCCTCTCAGGGTCGGCTACGCATCGTTGCCTTGGTAAGCCGTTACCTTACCAACTAGCTAATGCGGCGCGGATCCATCTATAAGTGACAGCAAAACCGTCTTTCACTATTGAACCATGCGGTTCAATATATT
>n.538.6.bb_292
GTAGTTAGCCGGTGCTTCTTCTGCGGGTAACGTCAATCGACAGGGTTATTAACCCTGTCGCCTTCCTCCCCGCTGAAAGTACTTTACAACCCGAAGGCCTTCTTCATACACGCGGCATGGCTGCATCAGGCTTGCGCCCATTGTGCAATATTCCCCACTGCTGCCTCCCGTAGGAGTCTGGACCGTGTCTCAGTTCCAGTGTGGCTGGTCATCCTCTCAGACCAGCTAGGGATCGTCGCCTAGGTGAGCCGTTACCCACTACTAGCTAATCCCATCTGGGCACATCCGATGG
>n.538.6.bb_293
GTAGTTAGCCGTGGCTTTCTGATTAGGTACCGTCAAGACGTGCATAGTTACTTACACATTTGTTCTTCCCTAATAACAGAGTTTTACGATCCGAAGACCTTCATCACTCACGCGGCGTTGCTCCGTCAGGCTTTCGCCCATTGCGGAAGATTCCCTACTGCTGCCTCCCGTAGGAGTCTGGACCGTGTCTCAGTTCCAGTGTGGCCGATCACCCTCTCAGGGTCGGCTACGCATCGTTGCCTTGGTAAGCCGTTACCTTACCAACTAGCTAATGCGGCGCGGATCCATCTATAAGTGACAGCAAAACCGTCTTTCACTATTGAACCATGCGGTTCAATAT
>n.538.6.bb_294
GTAGTTAGCCGTGGCTTTCTGATTAGGTACCGTCAAGACGTGCATAGTTACTTACACATTTGTTCTTCCCTAATAACAGAGTTTTACGATCCGAAGACCTTCATCACTCACGCGGCGTTGCTCCGTCAGGCTTTCGCCCATTGCGGAAGATTCCCTACTGCTGCCTCCCGTAGGAGTCTGGACCGTGTCTCAGTTCCAGTGTGGCCGATCACCCTCTCAGGGTCGGCTACGCATCGTTGCCTTGGTAAGCCGTTACCTTACCAACTAGCTAATGCGGCGCGGATCCATCTATAAGTGACAGCAAAACCGTCTTTCACTATTGAACCATGCGGTTCAATATATTATCCGGTATTAGCTCCGG
>n.538.6.bb_295
GTAGTTAGCCGTGGCTTTCTGATTAGGTACCGTCAAGACGTGCATAGTTACTTACACATTTGTTCTTCCCTAATAACAGAGTTTTACGATCCGAAGACCTTCATCACTCACGCGGCGTTGCTCCGTCAGGCTTTCGCCCATTGCGGAAGATTCCCTACTGCTGCCTCCCGTAGGAGTCTGGACCGTGTCTCAGTTCCAGTGTGGCCGATCACCCTCTCAGGGTCGGCTACGCATCGTTGCCTTGGTAAGCCGTTACCTTACCAACTAGCTAATGCGGCGCGGATCCATCTATAAGTGACAGCAAAACCGTCTTTCACTATTGAACCATGCGGTTCAATATATTATCCGGTAT
>n.538.6.bb_296
GTAGTTAGCCGGTGCTTCTTCTGCAGGTACCGTCACCACAAGCTTCGCCCCTGCTGAAAGCGGTTTACAACCCGAAGGCCGTCATCCCGCACGCGGCGTTGCTGCATCAGGCTTCCGCCCATTGTGCAATATTCCCCACTGCTGCCTCCCGTAGGAGTCTGGGCCGTATCTCAGTCCCAATGCGGCCGTCGCCCTCTCAGGCCGGCTACCCGTCAAAGCCTTGGTAACGCCACTACCCCACCAAACAAGCTGATAAGCCGCGAGTCC
>n.538.6.bb_297
GTAGTTAGCCGGTGCTTCTTCTGCAGGTACCGTCACCACAAGCTTCGCCCCTGCTGAAAGCGGTTTACAACCCGAAGGCCGTCATCCCGCACGCGGCGTTGCTGCATCAGGCTTCCGCCCATTGTGCAATATTCCCCACTGCTGCCTCCCGTAGGAGTCTGGGCCGTATCTCAGTCCCAATGTGGCCGGTCGCCCTCTCAGGCCGGCTACCCGTCAAAGCCTTGGTAAGCCACTACCCCACCAACAAGCTGATAAGCCGCGAGTCCATCCAAAACCGCCG
>n.538.6.bb_298
GTAGTTAGCCGGTGCTTCTTCTGCAGGTACCGTCACCACAAGCTTCGCCCCTGCTGAAAGCGGTTTACAACCCGAAGGCCGTCATCCCGCACGCGGCGTTGCTGCATCAGGCTTCCGCCCATTGTGCAATATTCCCCACTGCTGCCTCCCGTAGGAGTCTGGGCCGTATCTCAGTCCCAATGTGGCCGGTCGCCCTCTCAGGCCGGCTACCCGGTNAAAGCCTTGGTAAGCCACTACCCCACCAACAAGCTGATAAGCCGCGAGTCCATCCA
>n.538.6.bb_299
GTAGTTAGCCGTGGCTTTCTGATTAGGTACCGTCAAGACGTGCATAGTTACTTACACATTTGTTCTTCCCTAATAACAGAGTTTTACGATCCGAAGACCTTCATCACTCACGCGGCGTTGCTCCGTCAGGCTTTCGCCCATTGCGGAAGATTCCCTACTGCTGCCTCCCGTAGGAGTCTGGACCGTGTCTCAGTTCCAGTGTGGCTGGCCATCCTCTCAGACCAGCTAGGGGATCGTCGCCTAGGTGAGCCGTTACCCACCTACTAGCTAATCCCATCTGGGCACATCTGATGGCAAGAGGCCGAAGGTCCCCTCTTTGGTCTTGCGACGTTA
>n.538.6.bb_300
GGAGTTAGCCGGTGCTTCTTCTGCGGGTAACGTCAATTGCTGAGGTTATTAACCTCAACACCTTCCTCCCCGCTGAAAGTACTTTACAACCCGAAGGCCTTCTTCATACACGCGGCATGGCTGCATCAGGCTTGCGCCCATTGTGCAATATTCCCCACTGCTGCCTCCCGTAGGAGTCTGGACCGTGTCTCAGTTCCAGTGTAGCTGGTCATCCTCTCAGACCAGCTAGGGNTCGTCGCCTAGGT
>n.538.6.bb_301
GTAGTTAGCCGGTGCTTCTTCTGCAGGTACCGTCACCACAAGCTTCGCCCCTGCTGAAAGCGGTTTACAACCCGAAGGCCGTCATCCCGCACGCGGCGTTGCTGCATCAGGCTTCCGCCCATTGTGCAATATTCCCCACTGCTGCCTCCCGTAGGAGTCTGGGCCGTATCTCAGTCCCAATGTGGCCGGTCGCCCTCTCAGGCCGGCTACCCGTCAAAGCCTTGGTAAGCCACTACCCCACCAACAAGCTGATAAGCCGCGAGTCCATCCAAAAACCGCCGAAGCTTTTCCAACCCCCACCATGCAGCAAGGATTCCTATCCGGTATTAGCCCCAGTTTCCTGAAGTTATCCCGAAGTCAAGAGCAGGTTACTCACGTGTTACTCACCCGTT
>n.538.6.bb_302
GTAGTTAGCCGTGGCTTTCTGATTAGGTACCGTCAAGACGTGCATAGTTACTTACACATTTGTTCTTCCCTAATAACAGAGTTTTACGATCCGAAGACCTTCATCACTCACGCGGCGTTGCTCCGTCAGGCTTTCGCCCATTGCGGAAGATTCCCTACTGCTGCCTCCCGTAGGAGTCTGGACCGTGTCTCAGTTCCAGTGTGGCCGATCACCCTCTCAGGGTCGGCTACGCATCGTCGCCTTGGTAAGCCGTTACCTTACCAACTAGCTAATGCGGCGCGGATCCATCTATAAGTGACAGCAAAACCGTCTTTCACTATTGAACCATGCGGTTCAATATATTATCCCGGTAT
>n.538.6.bb_303
GTAGTTAGCCGTGGCTTTCTGATTAGGTACCGTCAAGACGTGCATAGTTACTTACACATTTGTTCTTCCCTAATAACAGAGTTTTACGATCCGAAGACCTTCATCACTCACGCGGCGTTGCTCCGTCAGGCTTTCGCCCATTGCGGAGGATTCCCTACTGCTGCCTCCCGTAGGAGTCTGGACCGTGTCTCAGTTCCAGTGTGGCCGATCACCCTCTCAGGGTCGGCTACGCATCGTTGCCTTGGTAAGCCGTTACCTTACCAACTAGCTAATGCGGCGCGGATCCATCTATAAGTGACAGCAAAACCGTCTTTCACTATTGAACCATGCGGTTCAATATATTATCCGGTATTA
>n.538.6.bb_304
GTAGTTAGCCGGTGCTTCTTCTGCAGGTACCGTCACCACAAGCTTCGCCCCTGCTGAAAGCGGTTTACAACCCGAAGGCCGTCATCCCGCACGCGGCGTTGCTGCATCAGGCTTCCGCCCATTGTGCAATATTCCCCACTGCTGCCTCCCGTAGGAGTCTGGGCCGTATCTCAGTCCCAATGTGGCCGGTCGCCCTCTCAGGCCGGCTACCCGGTCAAAGCCTTGGTAAGCCACTACCCACCAACAAGCTGATAAGCCGCGAGTCCATCC
>n.538.6.bb_305
GTAGTTAGCCGGTGCTTCTTCTGCAGGTACCGTCACCACAAGCTTCGCCCCTGCTGAAAGCGGTTTACAACCCGAAGGCCGTCATCCCGCACGCGGCGTTGCTGCATCAGGCTTCCGCCCATTGTGCAATATTCCCCACTGCTGCCTCCCGTAGGAGTCTGGGCCGTATCTCAGTCCCAATGTGGCCGGTCGCCCTCTCAGGCCGGCTACCCGTCAAAGCCTTGGTAAGCCACTACCCCACCAACAAGCTGATAAGCCGCGAGTCCATCCAAAACCGCCG
>n.538.6.bb_306
GTAGTTAGCCGGTGCTTCTTCTGCAGGTACCGTCACCACAAGCTTCGCCCCTGCTGAAAGCGGTTTACAACCCGAAGGCCGTCATCCCGCACGCGGCGTTGCTGCATCAGGCTTCCGCCCATTGTGCAATATTCCCCACTGCTGCCTCCCGTAGGAGTCTGGGCCGTATCTCAGTCCCAATGTGGCCGGTCGCCCTCTCAGGCCGGCTACCCGTCAAAGCCTTGGTAAGCCACTACCCCACCAACAAGCTGATAAGCCGCGAGTCCATCCAAAACCGCC
>n.538.6.bb_307
GGAGTTAGCCGGTGCTTCTTCTGCGGGTAACGTCAATTGCTGAGGTTATTAACCTCAACACCTTCCTCCCCGCTGAAAGTACTTTACAACCCGAAGGCCTTCTTCATACACGCGGCATGGCTGCATCAGGCTTGCGCCCATTGTGCAATATTCCCCACTGCTGCCTCCCGTAGGAGTCTGGACCGTGTCTCAGTTCCAGTGTGGCTGGGTCATCCTCTCAGACCAGCTAGGGATCGTCGCCTAGGTGAGCCGTTACCCACCTACTAGCTAATCCCATCTGGGCACATCTGATG
>n.538.6.bb_308
GTAGTTAGCCGTGGCTTTCTGATTAGGTACCGTCAAGACGTGCATAGTTACTTACACATTTATTCTTCCCTAATAACAGAGTTTTACGATCCGAAGACCTTCATCACTCACGCGGCGTTGCTCCGTCAGGCTTTCGCCCATTGCGGAAGATTCCCTACTGCTGCCTCCCGTAGGAGTCTGGACCGTGTCTCAGTTCCAGTGTGGCCGATCACCCTCTCAGGGTCGGCTACGCATCGTTGCCTTGGTAAGCCGTTACCTTACCAACTAGCTAATGCGGCGCGGATCCATCTATAAGTGACAGCAAAACCGTCTTTCACTATTGAACCATGCGGTTCAATATATTATCCGGTATTAGCTCCGGT
>n.538.6.bb_309
GTAGTTAGCCGTGGCTTTCTGATTAGGTACCGTCAAGACGTGCATAGTTACTTACACATTTGTTCTTCCCTAATAACAGAGTTTTACGATCCGAAGACCTTCATCACTCACGCGGCGTTGCTCCGTCAGGCTTTCGCCCATTGCGGAAGATTCCCTACTGCTGCCTCCCGTAGGAGTCTGGACCGTGTCTCAGTTCCAGTGTGGCCGATCACCCTCTCAGGGTCGGCTACGCATCGTGCCTTGGTAAGCCGTTACCTTACCAACTAGCTAATGCGGCGCGGATCCATCTATAAGTGACAGCAAAAACCGTCTTTCACTATTGAACCATGCGGTTCAATATATTATCCGGTATTAGCT
>n.538.6.bb_310
GGAGTTAGCCGGTGCTTCTTCTGCGGGTAACGTCAATCGACAAGGTTATTAACCTTATCGCCTTCCTCCCCGCTGAAAGTACTTTACAACCCGAAGGCCTTCTTCATACACGCGGCATGGCTGCATCAGGCTTGCGCCCATTGTGCAATATTCCCCACTGCTGCCTCCGTAGGAGTCTGGACCGTGTCTCAGTTCCAGTGTGGCTGGTCATCCTCTCAGACCAGCTAGGGATCGTCGCCTAGGTGAGCCGTTACCCACCTACTAGCTAATCCCATCTGGGCACATCTGATGGC
>n.538.6.bb_311
GTAGTTAGCCGGTGCTTCTTCTGCAGGTACCGTCACCACAAGCTTCGCCCCTGCTGAAAGCGGTTTACAACCCGAAGGCCGTCATCCCGCACGCGGCGTTGCTGCATCAGGCTTCCGCCCATTGTGCAATATTCCCCACTGCTGCCTCCCGTAGGAGTCTGGGCCGTATCTCAGTCCCAATGTGGCCGGTCGCCCTCTCAGGCCGGCTACCCGTCAAAGCCTTGGTAAGCCACTACCCACCAACAAGCTGATAAGCCGCGAGTCCATCCAAAACCGCCGAAGCCTTCCAACCCCACCATGCAGCAAGGATTCCTATCCGGTATTAGCCCCAGTTTCCTGAAGTTATCCCGAAG
>n.538.6.bb_312
GTAGTTAGCCGGTGCTTCTTCTGCAGGTACCGTCACCACAAGCTTCGCCCCTGCTGAAAGCGGTTTACAACCCGAAGGCCGTCATCCCGCACGCGGCGTTGCTGCATCAGGCTTCCGCCCATTGTGCAATATTCCCCACTGCTGCCTCCCGTAGGAGTCTGGGCCGTATCTCAGTCCCAATGTGGCCGGTCGCCCTCTCA
>n.538.6.bb_313
GTAGTTAGCCGGTGCTTCTTCTGCAGGTACCGTCACCACAAGCTTCGCCCCTGCTGAAAGCGGTTTACAACCGAGGCGTCATCCGCACGCGGCGTTGCTGCATCAGGCTTCCGCCCATTGTGCAATATTCCCCACTGCTGCCTCCCGTAGGAGTCTGGGCCGTATCTCAGTCCCAATGTGGCCGGTCGCCCTCTCAGGCCGGCCACCCGTCAAAGCCTTGGTAAGCCACTACCCACCAACAAGCTGATAAGCCGCGAGTCC
>n.538.6.bb_314
GTAGTTAGCCGGTGCTTCTTCTGCAGGTACCGTCACCACAAGCTTCGCCCCTGCTGAAAGCGGTTTACAACCCGAAGGCCGTCATCCCGCACGCGGCGTTGCTGCATCAGGCTTCCGCCCATTGTGCAATATTCCCCACTGCTGCCTCCCGTAGGAGTCTGGGCCGTATCTCAGTCCCAATGTGGCCGGTCGCCCTCTCAGGCCGGCTACCCGGTCAAAGCCTTGGTAAAGCCACTACCCCACCAACAAGCTGATAAGCCGCGAGTCCATCC
>n.538.6.bb_315
GTAGTTAGCCGTGGCTTTCTGATTAGGTACCGTCAAGACGTGCATAGTTACTTACACATTTATTCTTCCCTAATAACAGAGTTTTACGATCCGAAGACCTTCATCACTCACGCGGCGTTGCTCCGTCAGGCTTTCGCCCATTGCGGAAGATTCCCTACTGCTGCCTCCCGTAGGAGTCTGGACCGTGTCTCAGTTCCAGTGTGGCCGATCACCCTCTCAGGGTCGGCTACGCATCGTTGCCTTGGTAAGCCGTTACCTTACAACTAGCTAATGCGGCGCGGATCCATCTATAAGTGACAGCAAAACCGTCTTTCCCTATTGAACCATGCGGTTCAATATATTATCCGGTATT
>n.538.6.bb_316
GTAGTTAGCCGTGGCTTTCTGATTAGGTACCGTCAAGACGTGCATAGTTACTTACACATTTGTTCTTCCCTAATAACAGAGTTTTACGATCCGAAGACCTTCATCACTCACGCGGCGTTGCTCCGTCAGGCTTTCGCCCATTGCGGAAGATTCCCTACTGCTGCCTCCCGTAGGAGTCTGGACCGTGTCTCAGTTCCAGTGTGGCCGATCACCCTCTCAGGGTCGGCTACGCATCGTTGCCTTGGTAAGCCGTTACCTTACCAACTAGCTAATGCGGCGCGGATCCATCTATAAGTGACAGCAAAACCGTCTTTCACTATTGAACCATGCGGTTCAATATATTATCCGGTATTAGCTCCGG
>n.538.6.bb_317
GTAGTTAGCCGTGGCTTTCTGATTAGGTACCGTCAAGACGTGCATAGTTACTTACACATTTGTTCTTCCCTAATAACAGAGTTTTACGATCCGAAGACCTTCATCACTCACGCGGCGTTGCTCCGTCAAGCTTTCGCCCATTGCGGAAGATTCCCTACTGCTGCCTCCCGTAGGAGTCTGGACCGTGTCTCAGTTCCAGTGTGGCCGATCACCCTCTCAGGGTCGGCTACGCATCGTTGCCTTGGTAAGCCGTTACCTTACCAACTAGCTAATGCGGCGCGGATCCATCTATAAGTGACAGCAAAACCGTCTTTCACTATTGAACCATGCGGTTCAATATATTATCCCGGTATTAGCTCCGGT
>n.538.6.bb_318
GTAGTTAGCCGGTGCTTCTTCTGCAGGTACCGTCACCACAAGCTTCGCCCCTGCTGAAAGCGGTTTACAACCCGAAGGCCGTCATCCCGCACGCGGCGTTGCTGCATCAGGCTTCCGCCCATTGTGCAATATTCCCCACTGCTGCCTCCCGTAGGAGTCTGGGCCGTATCTCAGTCCCAATGTGGCCGGTCGCCCTCTCAGGCCGGCTACCCGTCAAAGCCTTGGTAAGCCACTACCCCACCAACAAGCTGATAAGCCGCGAGTCCATCCAAAACCGCC
>n.538.6.bb_319
GTAGTTAGCCGTGGCTTTCTGATTAGGTACCGTCAAGACGTGCATAGTTACTTACACATTTGTTCTTCCCTAATAACAGAGTTTTACGATCCGAAGACCTTCATCACTCACGCGGCGTTGCTCCGTCAGGCTTTCGCCCATTGCGGAAGATTCCCTACTGCTGCCTCCCGTAGGAGTCTGGACCGTGTCTCAGTTCCAGTGTGGCCGATCACCCTCTCAGGGTCGGCTACGCATCGCTGCCTTGGTAAGCCGTTACCTTACCAACTAGCTAATGCGGCGCGGATCCATCTATAAGTGAC
>n.538.6.bb_320
GTAGTTAGCCGTGGCTTTCTGATTAGGTACCGTCAAGACGTGCATAGTTACTTACACATTTGTTCTTCCCTAATAACAGAGTTTTACGATCCGAAGACCTTCATCACTCACGCGGCGTTGCTCCGTCAGGCTTTCGCCCATTGCGGAAGATTCCCTACTGCTGCCTCCCGTAGGAGTCTGGACCGTGTCTCAGTTCCAGTGTGGCCGATCACCCTCTTAGGGTCGGCTACGCATCGTCGCCTTGGTAAGCCGTTACCTTACCAACTAGCTAATGCGGCGCGGATCCATCTA
>n.538.6.bb_321
GGAGTTAGCCGGTGCTTCTTCTGCGGGTAACGTCAATCGACAGGGTTATTAACCCTGTCGCCTTCCTCCCCGCTGAAAGTACTTTACAACCCGAAGGCCTTCTTCATACACGCGGCATGGCTGCATCAGGCTTGCGCCCATTGTGCAATATTCCCCACTGCTGCCTCCCGTAGGAGTCTGGACCGTGTCTCAGTTCCAGTGTGGCTGGTCATCCTCTCAGACCAGCTAGGGATCGTCGCCTAGGGTGAGCCGTTACCCACCTACTAGCTAATCCCATCTGGGCACATCTGATG
>n.538.6.bb_322
TAGTTAGCCGTGGCTTTCTGATTAGGTACCGTCAAGACGTGCATAGTTACTTACACATTTGTTCTTCCCTAATAACAGAGTTTTACGATCCGAAGACCTTCATCACTCACGCGGCGTTGCTCCGTCAGGCCTTCGCCCATTGCGGAAGATTCCCTACTGCTGCCTCCCGTAGGAGTCTGGACCGTGTCTCAGTTCCAGTGTGGCCGATCACCCTCTCAGGGTCGGCTACGCATCGTTGCCTTGGTAAGCCGTTACCTTACCAACTAGCTAATGCGGCGCGGATCCATCTATAAGTGACAGCAAAACCGTCTTTCACTATTGAACCATGCGGTTCAATATATATCCGGTATTAGCT
>n.538.6.bb_323
GTAGTTAGCCGTGGCTTTCTGATTAGGTACCGTCAAGACGTGCATAGTTACTTACACATTTATTCTTCCCTAATAACAGAGTTTTACGATCCGAAGACCTTCATCACTCACGCGGCGTTGCTCCGTCAGGCTTTCGCCCATTGCGGAAGATTCCCTACTGCTGCCTCCCGTAGGAGTCTGGACCGTGTCTCAGTTCCAGTGTGGCCGATCACCCTCTCAGGGTCGGCTACGCATCGTGCCTTGGTAAGCCGTTACCTTACCAACTAGCTAATGCGGCGCGGATCCATCTATAAGTGACAGCAAAACCGTCTTTCACTATTGAACCATGCGGTTCAATATATTATCCGGTATTA
>n.538.6.bb_324
GTAGTTAGCCGGTGCTTCTTCTGCAGGTACCGTCACCACAAGCTTCGCCCCTGCTGAAAGCGGTTTACAACCCGAAGGCCGTCATCCCGCACGCGGCGTTGCTGCATCAGGCTTCCGCCCATTGTGCAATATTCCCCACTGCTGCCTCCCGTAGGAGTCTGGGCCGTATCTCAGTCCCAATGTGGCCCGGTCGCCCTCTCAGGCCGGGCTACCCGTCAAAGCCTTGGTAAGCCACTACCCCACCAACAAGCTGATAAGCCGCGAGTCCATCCAAAACCG
>n.538.6.bb_325
GTAGTTAGCCGTGGCTTTCTGATTAGGTACCGTCAAGACGTGCATAGTTACTTACACATTTGTTCTTCCCTAATAACAGAGTTTTACGATCCGAAGACCTTCATCACTCACGCGGCGTTGCTCCGTCAGGCTTTCGCCCATTGCGGAAGATTCCCTACTGCTGCCTCCCGTAGGAGTCTGGACCGTGTCTCAGTTCCAGTGTGGCCGATCACCCTCTCAGGGTCGGCTACGCATCGTTGCCTTGGTAAGCCGTTACCTTACCAACTAGCTAATGCGGCGCGGATCCATCTATAAGTGACAGCAAAACCGTCTTTCACTATTGAACCATGCGGTTCAATATATTATCCGGTATTAGCTCCGGTTTCCCGAAGTTATCCCAGTCTTATAGGTAGGTTATCCCACGTGTTACCTCACCCCGTCCCGCCCGCTAACGTCAGAGGAGCAAGCTCCCTCGTCTGTTCGCTCGACTTGCATGTATTAGGGCACGCCGCCAGCG
>n.538.6.bb_326
GTAGTTAGCCGTGGCTTTCTGATTAGGTACCGTCAAGACGTGCATAGTTACTTACACATTTGTTCTTCCCTAATAACGGAGTTTTACGATCCGAAGACCTTCATCACTCACGCGGCGTTGCTCCGTCAGGCTTTCGCCCATTGCGGAAGATTCCCTACTGCTGCCTCCCGTAGGAGTCTGGACCGTGTCTCAGTTCCAGTGTGGCCGATCACCCTCTCAGGGTCGGCTACGCATCGTCGCCTTGGTAAGCCGTTACCTTACCAACTAGCTAATGCGGCGCGGATCCATCTATAAGTGACAGCAAAACCGTCTTTCACTATTGAACCATGCGGTTCAATATATT
>n.538.6.bb_327
GTAGTTAGCCGTGGCTTTCTGATTAGGTACCGTCAAGACGTGCATAGTTACTTACACATTTGTTCTTCCCTAATAACAGAGTTTTACGATCCGAAGACCTTCATCACTCACGCGGCGTTGCTCCGTCAGGCTTTCGCCCATTGCGGAAGATTCCCTACTGCTGCCTCCCGTAGGAGTCTGGACCGTGTCTCAGTTCCAGTGTGGCCGATCACCCTCTCAGGGTCGGCTACGCATCGTTGCCTTGGTAAGCCGTTACCTTACCAACTAGCTAATGCGGCGCGGATCCATCTATAAGTGACAGCAAAACCGTCTTTCACTATTGAACCATGCGGTTC
>n.538.6.bb_328
GGAGTTAGCCGGTGCTTCTTCTGCGGGTAACGTCAATCGACAAGGTTATTAACCTTATCGCCTTCCTCCCCGCTGAAAGTACTTTACAACCCGAAGGCCTTCTTCATACACGCGGCATGGCTGCATCAGGCTTGCGCCCATTGTGCAATATTCCCCACTGCTGCCTCCGTAGGAGTCTGGACCGTGTCTCAGTTCCAGTGTGGCTGGTCATCCTCTCAGACCAGCTAGGGATCGTCGCCTAGGTGAGCCGTTACCCACCTACTAGCTAATCCCATCTGGGCACATCTGAT
>n.538.6.bb_329
GTAGTTAGCCGTGGCTTTCTGATTAGGTACCGTCAAGACGTGCATAGTTACTTACACATTTGTTCTTCCCTAATAACAGAGTTTTGCGATCCGAAGACCTTCATCACTCACGCGGCGTTGCTCCGTCAGGCTTTCGCCCATTGCGGAAGATTCCCTACTGCTGCCTCCCGTAGGAGTCTGGACCGTGTCTCAGTTCCAGTGTGGCCGATCACCCTCTCAGGGTCGGCTACGCATCGTTGCCTTGGTAAGCCGTTACCTTACCAACTAGCTAATGCGGCGCGGATCCATCTATGAGTGACAGCAAAACCGTCTTTCACTATTGAACCATGCGGTTCAATATATTATCCGGTATTAGCTCCGGTTTCCCGAAGTTATCCCAGTCTTATAGGTAGGTTATCCCACGTGTTACTCACCCCGTCCGCCCGCTAACGTCAGAGGAGCAAGCCTCCTCGTCTGTTCGCTCGACTTGCATGTATTAGGGCACGCCGCCAGCGTTCA
>n.538.6.bb_330
GTAGTTAGCCGGTGCTTCTTCTGCAGGTACCGTCACCACAAGCTTCGCCCCTGCTGAAAGCGGTTTACAACCCGAAGGCCGTCATCCCGCACGCGGCGTTGCTGCATCAGGCTTCCGCCCATTGTGCAATATTCCCCACTGCTGCCTCCCGTAGGAGTCTGGGCCGTATCTCAGTCCCAATGTGGCCGGTCGCCCTCTCAGGCCGGCTACCCGGTCAAAGCCTTGGTAAGCCACTACCCACCAACAAGCTGATAAGCCGCGAGTCCATCCAAAACCGCC
>n.538.6.bb_331
GTAGTTAGCCGTGGCTTTCTGATTAGGTACCGTCAAGACGTGCATAGTTACTTACACATTTGTTCTTCCCTAATAACAGAGTTTTACGATCCGAAGACCTTCATCACTCACGCGGCGTTGCTCCGTCAGGCTTTCGCCCATTGCGGAAGATTCCCTACTGCTGCCTCCCGTAGGAGTCTGGACCGTGTCTCAGTTCCAGTGTGGCCGATCACCCTCTCAGGGTCGGCTACGCATCGTTGCCTTGGTAAGCCGTTACCTTACCAACTAGCTAATGCGGCGCGGATCCATCTATAAGTGACAGCAAAACCGTCTTTCATTATTGAACCATGCGGTTCAATATATTATCCGGTATTAG
>n.538.6.bb_332
GAGTTAGCCGGTGCTTCTTCTGCGGGTAACGTCAATCGACAAGGTTATTAACCTTATCGCCTTCCTCCCCGCTGAAAGTACTTTACAACCCGAAGGCCTTCTTCATACACGCGGCATGGCTGCATCAGGCTTGCGCCCATTGTGCAATATTCCCCACTGCTGCCTCCCGTAGGAGTCTGGACCGTGTCTCAGTTCCAGTGTGGCTGGTCATCCTCTCAGACCAGCTAGGGGATCGTCGCCTAGGTGAGCCGTTACCCACCTACTAGCTAATCCCATCTGGGCACATCTGATGGCA
>n.538.6.bb_333
GTAGTTAGCCGGTGCTTCTTCTGCAGGTACCGTCACCACAAGCTTCGCCCCTGCTGAAAGCGGTTTACAACCCGAAGGCCGTCATCCCGCACGCGGCGTTGCTGCATCAGGCTTCCGCCCATTGTGCAATATTCCCCACTGCTGCCTCCCGTAGGAGTCTGGGCCGTATCTCAGTCCCAATGTGGCCGGTCGCCCTCTCAGGCCGGCTACCCGTCAAAGCCTTGGTAAGCCACTACCCCACCAACAAGCTGATAAGCCGCGAGTCCATCCAAAACCGCCGAAGCTTTCCAACCCCACCATGCAGCAAGGATTCCTATCCGGTATTAGCCCCAGTTTCCTGAAGT
>n.538.6.bb_334
GTAGTTAGCCGTGGCTTTCTGATTAGGTACCGTCAAGACGTGCATAGTTACTTACACATTTGTTCTTCCCTAATAACAGAGTTTTACGATCCGAAGACCTTCATCACTCACGCGGCGTTGCTCCGTCAGGCTTTCGCCCATTGCGGAAGATTCCCTACTGCTGCCTCCCGTAGGAGTCTGGACCGTGTCTCAGTTCCAGTGTGGCCGATCACCCTCTCAGGGTCGGCTACGCATCGTTGCCTTGGTAAGCCGTTACCTTACCAACTAGCTAATGCGGCGCGGATCCATCTATAAGTGACAGCAAAACCGTCTTTCACTATTGAACCATGCGGTTCAATATATTATCCGGTATT
>n.538.6.bb_335
GGAGTTAGCCGGTGCTTCTTCTGCGGGTAACGTCAATCGACAGGGTTATTAACCCTGTCGCCTTCCTCCCCGCTGAAAGTACTTTACAACCCGAAGGCCTTCTTCATACACGCGGCATGGCTGCATCAGGCTTGCGCCCATTGTGCAATATTCCCCACTGCTGCCTCCCGTAGGAGTCTGGACCGTGTCTCAGTTCCAGTGTGGCTGGTCATCCTCTCAGACCAGCTAGGGGATCGTCGCCTAGGTGAGCCGTTACCCACCTACTAGCTAATCCCATCTGGGCACATCTGATGGCAAGAGG
>n.538.6.bb_336
GTAGTTAGCCGTGGCTTTCTGATTAGGTACCGTCAAGACGTGCATAGTTACTTACACATTTATTCTTCCCTAATAACAGAGTTTTACGATCCGAAGACCTTCATCACTCACGCGGCGTTGCTCCGTCAGGCTTTCGCCCATTGCGGAAGATTCCCTACTGCTGCCTCCCGTAGGAGTCTGGACCGTGTCTCAGTTCCAGTGTGGCCGATCACCCTCTCAGGGTCGGCTACGCATCGTTGCCTTGGTAAGCCGTTACCTTACCAACTAGCTAATGCGGCGCGGATCCATCTATAAGTGACAGCAAAACCGTCTTTCACTATTGAACCATGCGGTTCAATATATTA
>n.538.6.bb_337
GTAGTTAGCCGTGGCTTTCTGATTAGGTACCGTCAAGACGTGCATAGTTACTTACACATTTGTTCTTCCCTAATAACAGAGTTTTACGATCCGAAGACCTTCATCACTCACGCGGCGTTGCTCCGTCAGGCTTTCGCCCATTGCGGAAGATTCCCTACTGCTGCCTCCCGTAGGAGTCTGGACCGTGTCTCAGTTCCAGTGTGGCCGATCACCCTCTCAGGGTCGGGCTACGCATCGTTGCCTTGGTAAGCCGTTACCTTACCAACTAGCTAATGCGGCGCGGATCCATCTATAAGTGACAGCAAAACCGTCTTTCACTATTGAACCATGCGGTTCAATATATTATCCGGTATT
>n.538.6.bb_338
GGAGTTAGCCGGTGCTTCTTCTGCGGGTAACGTCAATCGACAAGGTTATTAACCTTATCGCCTTCCTCCCCGCTGAAAGTACTTTACAACCCGAAGGCCTTCTTCATACACGCGGCATGGCTGCATCAGGCTTGCGCCCATTGTGCAATATTCCCCACTGCTGCCTCCCGTAGGAGTCTGGACCGTGTCTCAGTTCCAGTGTGGCTGGGTCATCCTCTCAGACCAGCTAGGGATCGTCGCCTAGGTGAGCCGTTACCCACCTACTAGCTAATCCCATCTGGGCACATCTGAT
>n.538.6.bb_339
GTAGTTAGCCGTGGCTTTCTGATTAGGTACCGTCAAGACGTGCATAGTTACTTACACATTTGTTCTTCCCTAATAACAGAGTTTTACGATCCGAAGACCTTCATCACTCACGCGGCGTTGCTCCGTCAGGCTTTCGCCCATTGCGGAAGATTCCCTACTGCTGCCTCCCGTAGGAGTCTGGACCGTGTCTCAGTTCCAGTGTGGCCGATCACCCTCTCAGGGTCGGCTACGCATCGTTGCCTTGGTAAGCCGTTACCTTACCAACTAGCTAATGCGGCGCGGATCCATCTATAAGTGACAGCAAAACCGTCTTTCACTATTGAACCATGCGGTTCAATATATTATCCGGTATTAGCTCCGGTTTCCCGAAGTTATCCCAGTCTTATAGGTAGGTTATC
>n.538.6.bb_340
GTAGTTAGCCGTGGCTTTCTGATTAGGTACCGTCAAGACGTGCATAGTTACTTACACATTTGTTCTTCCCTAATAACAGAGTTTTACGATCCGAAGACCTTCATCACTCACGCGGCGTTGCTCCGTCAGGCTTTCGCCCATTGCGGAAGATTCCCTACTGCTGCCTCCCGTAGGAGTCTGGACCGTGTCTCAGTTCCAGTGTGGCCGATCACCCTCTCAGGGTCGGCTACGCATCGTTGCCTTGGTAAGCCGTTACCTTACCACTAGCTAATGCGGCGCGGATCCATCTATAAGTGACAGCAAAACCGTCTTTCACTATTG
>n.538.6.bb_341
GTAGTTAGCCGTGGCTCTCTGATTAGGTACCGTCAAGACGTGCATAGTTACTTACACATTTGTTCTTCCCTAATAACAGAGTTTTACGATCCGAAGACCTTCATCACTCACGCGGCGTTGCTCCGTCAGGCTTTCGCCCATTGCGGAAGATTCCCTACTGCTGCCTCCCGTAGGAGTCTGGACCGTGTCTCAGTTCCAGTGTGGCCGATCACCCTCTCAGGTCGGCTACGCATCGTTGCCTTGGTAAGCCGTTACCTTACCAACTAGCTAATGCGGCGCGGATCCATCTATAAGTGACAGCAAAACCGTCTTTCACTATTGAACCATGCGGTTCAATATATTATCCGGTATTAGCTCCGGTTTCCCGAAGTTATCCCAGTCTTATAGGTAGGTTATCCCACGTGTTACTCACCCCGTCCCGCCCGCTAACGTCAGAGAAGCAAGCCTCCCTCGTCTGTTCGCTCGACTTGCATGTATTAGGGCACGCC
>n.538.6.bb_342
GTAGTTAGCCGTGGCTTTCTGATTAGGTACCGTCAAGACGTGCATAGTTACTTACACATTTGTTCTTCCCTAATAACAGAGTTTTACGATCCGAAGACCTTCATCACTCACGCGGCGTTGCTCCGTCAGGCTTTCGCCCATTGCGGAAGATTCCCTACTGCTGCCTCCCGTAGGAGTCTGGACCGTGTCTCAGTTCCAGTGTGGCCGATCACCCTCTCAGGGTCGGCTACGCATCGTCGCCTTGGTAAGCCGTTACCTTACCAACTAGCTAATGCGGCGCGGATCCATCTATAAGTGACAGCAAAACCGTCTTTCACTATTGAACCATGCGGTTCAATATATTATCCGGTATTA
>n.538.6.bb_343
GTAGTTAGCCGGTGCTTCTTCTGCAGGTACCGTCACCACAAGCTTCGCCCCTGCTGAAAGCGGTTTACAACCCGAAGGCCGTCATCCCGCACGCGGCGTTGCTGCATCAGGCTTCCGCCCATTGTGCAATATTCCCCACTGCTGCCTCCCGTAGGAGTCTGGGCCGTATCTCAGTCCCAATGTGGCCGGTCGCCCTCTCAGGCCGGCTACCCGGTCAAAGCCTTGGTAAGCCACTACCCCACCAACAAGCTGATAAGCCGCGAGTCCATCCAAAACCGCCGAAGCTTTCCAACCCCACCATGCAGCAAGGATTCCTATCCGGTATTAG
>n.538.6.bb_344
GTAGTTAGCCGTGGCTTTCTGATTAGGTACCGTCAAGACGTGCATAGTTACTTACACATTTGTTCTTCCCTAATAACAGAGTTTTTACGATCCGAAAGACCTTCATCACTCACGCGGCGTTGCTCCGTCAGGCTTTCGCCCATTGCGGAAGATTCCCTACTGCTGCCTCCCGTAGGAGTCTGGACCGTGTCTCAGTTCCAGTGTGGCCGATCACCCTNTCAGGGTCGGCTACGCATCGTCGCCTTGGTAAGCCGTTACCTTACCAACTAGCTAATGCGGCGCGGATCCA
>n.538.6.bb_345
GTAGTTAGCCGGTGCTTCTTCTGCAGGTACCGTCACCACAAGCTTCGCCCCTGCTGAAAGCGGTTTACAACCCGAAGGCCGTCATCCCGCACGCGGCGTTGCTGCATCAGGCTTCCGCCCATTGTGCAATATTCCCCACTGCTGCCTCCCGTAGGAGTCTGGGCCGTATCTCAGTCCCAATGTGGCCGGTCGCCCTCTCAGGCCGGCTACCCGTCAAAGCCTTGGTAAGCCACTACCCCACCAACAAGCTGATAAGCCGCGAGTCCATCCAAAACCGCCGAAGCTTTCCAACCCCCACCATGCAGCAAGGATTCCTATCCGGTATTAGCCCCAGTTTCCTGAAGTTATCCCGAAGTCAAGGGCAGGTTACTCACGTGTTACTCACCCGTTCGCCA
>n.538.6.bb_346
GTAGTTAGCCGTGGCTTTCTGATTAGGTACCGTCAAGACGTGCATAGTTACTTACACATTTGTTCTTCCCTAATAACAGAGTTTTACGATCCGAAGACCTTCATCACTCACGCGGCGTTGCTCCGTCAAGCTTTCGCCCATTGCGGAAGATTCCCTACTGCTGCCTCCCGTAGGAGTCTGGACCGTGTCTCAGTTCCAGTGTGGCCGATCACCCTCTCAGGGTCGGCTACGCATCGTTGCCTTGGTAAGCCGTTACCTTACCAACTAGCTAATGCGGCGCGGATCCATCTATAAGTGACAGCAAAACCGTCTTTCACTATTGAACCA
>n.538.6.bb_347
GTAGTTAGCCGTGGCTTTCTGATTAGGTACCGTCAAGACGTGCATAGTTACTTACACATTTGTTCTTCCCTAATAACAGAGTTTTACGATCCGAAGACCTTCATCACTCACGCGGCGTTGCTCCGTCAGGCTTTCGCCCATTGCGGAAGATTCCCTACTGCTGCCTCCCGTAGGAGTCTGGACCGTGTCTCAGTTCCAGTGTGGCCGATCACCCTCTCAGGGTCGGCTACGCATCGTTGCCTTGGTAAGCCGTTACCTTACCAACTAGCTAATGCGGCGCGGATCCATCTATAAGTGACAGCAAAACCGTCTTTCACTATTGAACCATGCGGTTCAATATATTATCCGGTATTAGCTCCGGTTTCCCGAAGTTATCCCAGTCTTATAGGTAGGTTATCC
>n.538.6.bb_348
GGAGTTAGCCGGTGCTTCTTCTGCGGGTAACGTCAATTGCTGAGGTTATTAACCTCAACACCTTCCTCCCCGCTGAAAGTACTTTACAACCCGAAGGCCTTCTTCATACACGCGGCATGGCTGCATCAGGCTTGCGCCCATTGTGCAATATTCCCCACTGCTGCCTCCCGTAGGAGTCTGGACCGTGTCTCAGTTCCAGTGTGGCTGGGTCATCCTCTCAGACCAGCTAGGGGATCGTCG
>n.538.6.bb_349
GGAGTTAGCCGGTGCTTCTTCTGCGGGTAACGTCAATCGACAGGGTTATTAACCCTGTCGCCTTCCTCCCCGCTGAAAGTACTTTACAACCCGAAGGCCTTCTTCATACACGCGGCATGGCTGCATCAGGCTTGCGCCCATTGTGCAATATTCCCACTGCTGCCTCCGTAGGAGTCTGGACCGTGTCTCAGTTCCAGTGTGGCTGGTCATCCTCTCAGACCAGCTAGGGATCGTCGCCTAGGTGAGCCGTTACCCACCTACTAGCTAATCCCATCTGGGCACATCTGATGGC
>n.538.6.bb_350
GTAGTTAGCCGGTGCTTCTTCTGCAGGTACCGTCACCACAAGCTTCGCCCCTGCTGAAAGCGGTTTACAACCCGAAGGCCGTCATCCCGCACGCGGCGTTGCTGCATCAGGCTTCCGCCCATTGTGCAATATTCCCCACTGCTGCCTCCCGTAGGAGTCTGGGCCGTATCTCAGTCCCAATGTGGCCGGTCGCCCTCTCAGGCCGGCTACCCGGTCAAAGCCTTGGTAAGCCACTACCCACCAACAAGCTGATAAGCCGCGAGTCCATCCAAAACCGCCGAAGCTTTTCCAACCCCCACCATGCAGCAAGGATTCCTATCCGGTATTAGCCCCAGTTTCCTGAAGTT
>n.538.6.bb_351
GTAGTTAGCCGTGGCTTTCTGATTAGGTACCGTCAAGACGTGCATAGTTACTTACGACATTTGTTCTTCCCTAATAACAGAGTTTTACGATCCGAAGACCTTCATCACTCACGCGGCGTTGCTCCGTCAGGCTTTCGCCCATTGCGGAAGATTCCCTACTGCTGCCTCCCGTAGGAGTCTGGACCGTGTCTCAGTTCCAGTGTGGCCGATCACCCTCTCAGGGTCGGCTACGCATCGTTGCCTTGGTAAGCCGTTACCTTACCAACTAGCTAATGCGGCGCGGATCCATCTATAAGTGACAGCAAAACCGTCTTTCACTATTGAACCATGCGGTTCAATATATTATCCGG
>n.538.6.bb_352
GTAGTTAGCCGTGGCTTTCTGATTAGGTACCGTCAAGACGTGCATAGTTACTTACACATTTGTTCTTCCCTAATAACAGAGTTTTACGATCCGAGGACCTTCATCACTCACGCGGCGTAGCTCCGTCAGGCTTTCGCCCATTGCGGAAGATTCCCTACTGCTGCCTCCCGTAGGAGTCTGGACCGTGTCTCAGTTCCAGTGTGGCCGATCACCCTCTCAAGTCGGCTACGCATCGTTGCCTTGGTAAGCCGTTACCTTACAACTAGCTAATGCGGCGCGGATCCATCTATAAGTGACAGCAAAACCGTCTTTCACTATTGAACCATGCGGTTCAATATATTATCCGGTATTAGCTCCGGTTTCCCGAAGTTATCCCAGTCTTATAGGTAGGTTAT
>n.538.6.bb_353
GTAGTTAGCCGTGGCTTTCTGATTAGGTACCGTCAAGACGTGCATAGTTACTTACACATTTGTTCTTCCCTAATAACAGAGTTTTACGATCCGAAGACCTTCATCACTCACGCGGCGTTGCTCCGTCAGGCTTTCGCCCATTGCGGAAGATTCCCTACTGCTGCCTCCCGTAGGAGTCTGGACCGTGTCTCAGTTCCAGTGTGGCCGATCACCCTCTCAGGGTCGGCTACGCATCGTTGCCTTGGTAAGCCGTTACCTTACCAACTAGCTAATGCGGCGCGGATCCATCTATAAGTGACAGCAAAACCGTCTTTCACTATTGAACCATGCGGTTCAATATATTATCCGGTAT
>n.538.6.bb_354
GTAGTTAGCCGGTGCTTCTTCTGCAGGTACCGTCACCACAAGCTTCGCCCCTGCTGAAAGCGGTTTACAACCCGAAGGCCGTCATCCCGCACGCGGCGTTGCTGCATCGGGCTTCCGCCCATTGTGCAATATTCCCCACTGCCGCCTCCCGTAGGAGTCTGGGCCGTATCTCAGTCCCAATGTGGCCGGTCGCCCTCTCAGGCCGGCTACCCGTCAAAGCCTTGGGTAAGCCCACTACCCACCAACAAGCTGATAAGCCGCGAGTCCATCCAAAACCGCCGAAGCTTTTCCAACCCCACCATGCAGCAAGGATTCCTATCCGGTATTAGCCCCAGTTTCCTGAAGTTATCCCGAAGTCAAGGGCAGGTTACTCACGTGTTACTCACCC
>n.538.6.bb_355
GTAGTTAGCCGGTGCTTCTTCTGCAGGTACCGTCACCACAAGCTTCGCCCCTGCTGAAAGCGGTTTACAACCCGAAGGCCGTCATCCCGCACGCGGCGTTGCTGCATCAGGCTTCCGCCCATTGTGCAATATTCCCCACTGCTGCCTCCCGTAGGAGTCTGGGCCGTATCTCAGTCCCAATGTGGCCGGTCGCCCTCTCAGGCCGGCTACCCGGTCAAAGCCTTGGTAAAGCCACTACCCCACCAACAAGCTGATAAGCCGCGAGTCCATCCAAAACCGCCG
>n.538.6.bb_356
GTAGTTAGCCGGTGCTTCTTCTGCAGGTACCGTCACCACAAGCTTCGCCCCTGCTGAAAGCGGTTTACAACCCGAAGGCCGTCATCCCGCACGCGGCGTTGCTGCATCAGGCTTCCGCCCATTGTGCAATATTCCCCACTGCTGCCTCCCGTAGGAGTCTGGGCCGTATCTCAGTCCCAATGTGGCCGGTCGCCCTCTCAGGCCGGCTACCCGGTCAAAGCCTTGGTAAGCCACTACCCCACCAACAAGCTGATAAGCCGCGAGTCCATCCAAAACCGCCGAAGCTTTCCAACCCCACCATGCAGCAAGGATTCCTATCCGGTATTAGCCCCAGTTTCCTGAAGTTAT
>n.538.6.bb_357
GTAGTTAGCCGGTGCTTCTTCTGCAGGTACCGTCACCACAAGCTTCGCCCCTGCTGAAAGCGGTTTACAACCCGAAGGCCGTCATCCCGCACGCGGCGTTGCTGCATCAGGCTTCCGCCCATTGTGCAATATTCCCCACTGCTGCCTCCCGTAGGAGTCTGGGCCGTATCTCAGTCCCAATGTGGCCGGTCGCCCTCTCAGGCCCGGCTACCCCGGTCAAAGGCCTTGGTAAAGCCACTACCCCACCAACAAGCTGATAAGCCGCGAGTCCATCCAAAACCGCC
>n.538.6.bb_358
GGAGTTAGCCGGTGCTTATTCTGCGAGTAACGTCCACTATCCAAGAGTATTAGTCTCAGTAGCCTCCTCCTCGCTTAAAGTGCTTTACAACCATAAGGCCTTCTTCACACACGCGGCATGGCTGGATCAGGGTTCCCCCCATTGTCCAATATTCCCCACTGCTGCCTCCCGTAGGAGTCTGGGCCGTGTCTCAGTCCCAGTGTGGCGGATCATCCTCTCAGACCCGCTACAGATCGTCGCCTTGGTAGGCCTTTACCCACCAACTAGCTAATCCGACTTAGGCTCATCTATTAGCGCAAGGTCCGAAGA
>n.538.6.bb_359
GTAGTTAGCCGTGGCTTTCTGATTAGGTACCGTCAAGACGTGCATAGTTACTTACACATTTATTCTTCCCTAATAACAGAGTTTTACGATCCGAAGACCTTCATCACTCACGCGGCGTTGCTCCGTCAGGCTTTCGCCCATTGCGGAAGATTCCCTACTGCTGCCTCCCGTAGGAGTCTGGACCGTGTCTCAGTTCCAGTGTGGCCGATCACCCTCTCAGGGTCGGCTACGCATCGTTGCCTTGGTAAGCCGTTACCTTACCAACTAGCTAATGCGGCGCGGATCCATCTATAAGTGACAGCAAAACCGTCTTTCACTATTGAACCATGCGGTTCAATATATTATCCGGTATTAGCTCCGGTTTCCCGAAGTTATCCCAGTCTTATAGGTAGGTTATCCCACGTGTTACTCACCCCGTCCCGCCCGCTAACGTCAGAGGGAGCAAGCTCCCTCGTCTGTTCGCTCGACTTGCATGTATTAGGGCACGCCGCCAGCGTTCATCCT
>n.538.6.bb_360
GTAGTTAGCCGTGGCTTTCTGATTAGGTACCGTCAAGACGTGCATAGTTACTTACACATTTGTTCTTCCCTAATAACAGAGTTTTACGATCCGAAGACCTTCATCACTCACGCGGCGTTGCTCCGTCAGGCTTTCGCCCATTGCGGAAGATTCCCTACTGCTGCCTCCCGTAGGAGTCTGGACCGTGTCTCAGTTCCAGTGTGGCCGATCACCCTCTCAGGGTCGGCTACGCATCGTTGCCTTGGTAAGCCGTTACCTTACCAACTAGCTAATGCGGCGCGGATCCATCTATAAGTGACAGCAAAACCGTCTTTCACTATTGAACCATGCGGTTCAATATATTATCCGGTATTAG
>n.538.6.bb_361
GTAGTTAGCCGTGGCTTTCTGATTAGGTACCGTCAAGACGTGCATAGTTACTTACACATTTGTTCTTCCCTAATAACAGAGTTTTACGATCCGAAGACCTTCATCACTCACGCGGCGTTGCTCCGTCAGGCTTTCGCCCATTGCGGAAGATTCCCTACTGCTGCCTCCCGTAGGAGTCTGGACCGTGTCTCAGTTCCAGTGTGGCCGATCACCCTCTCAGGGTCGGCTACGCATCGTCGCCTTGGTAAGCCGTTACCTTACAACTAGCTAATGCGGCGCGGATCCATCTATAAGTGACAGCAAAACCGTCTTTCACTATTGAACCATGCGGTTCAATATATTATCCGGTATTAGCTCCGGTTTCCCGAAGT
>n.538.6.bb_362
GTAGTTAGCCGTGGCTTTCTGATTAGGTACCGTCAAGACGTGCATAGTTACTTACACATTTGTTCTTCCCTAATAACAGAGTTTTACGACCCGAAGACCTTCATCACTCACGCGGCGTTGCTCCGTCAGGCTTTCGCCCATTGCGGAAGATTCCCTACTGCTGCCTCCCGTAGGAGTCTGGACCGTGTCTCAGTTCCAGTGTGGCCGATCACCCTCCAGGGTCGGCTACGCATCGTTGCCTTGGTAAGCCGTTACTTACCAACTAGCTAATGCGGCGCGGA
>n.538.6.bb_363
GTAGTTAGCCGTGGCTTTCTGATTAGGTACCGTCAAGACGTGCATAGTTACTTACACATTTGTCCTTCCCTAATAACAGAGTTTTACGATCCGAAGACCTTCATCACTCACGCGGCGTTGCTCCGTCAGGCTTTCGCCCATTGCGGAGGATTCCCTACTGCTGCCTCCCGTAGGAGTCTGGACCGTGTCTCAGTTCCAGTGTGGCCGATCACCCTCTCAGGGTCGGCTACGCATCGTTGCCTTGGTAAGCCGTTACCTTACCAACTAGCTAATGCGGCGCGGATCCATCTATAAAGTGACAGCAAAACCGTCTTTCACTATTGAACCATGCGGTTCAATATATTATCCGGTATTAGCTCC
>n.538.6.bb_364
GTAGTTAGCCGTGGCTTTCTGATTAGGTACCGTCAAGACGTGCATAGTTACTTACACATTTGTTCTTCCCTAATAACAGAGTTTTACGATCCGAAGACCTTCATCACTCACGCGGCGTTGCTCCGTCAGGCTTTCGCCCATTGCGGAAGATTCCCTACTGCTGCCTCCCGTAGGAGTCTGGACCGTGTCTCAGTTCCAGTGTGGCCGATCACCCTCTCAGGGTCGGCTACGCATCGTTGCCTTGGTAAGCCGTTACCTTACCAACTAGCTAATGCGGCGCGGATCCATCTATAAGTGACAGCAAAACCGTCTTTCACTATTGAACCATGCGGTTCAATATATTATCCGGTATTAGCTCCGGT
>n.538.6.bb_365
GAGTTAGCCGGTGCTTCTTCTGCGGGTAACGTCAATCGACACGGTTATTAACCGCATCGCCTTCCTCCCCGCTGAAAGTACTTTACAACCCGAAGGCCTTCTTCATACACGCGGCATGGCTGCATCAGGCTTGCGCCCATTGCCGAAGATTCCCTACTGCTGCCTCCCGTAGGAGTCTGGGCCGTGTCTCAGTCCCAGTGTGGCCGATCACCCTCTCAGGTCGGCTATGCATCGTGGCCTTGGTGAGCCGTTACCTCACCAACTAGCTAATGCACCGCGGGTCCATCCATCAGCGACACCCGAAAGCGCCTTTCACTCTTATGCCATG
>n.538.6.bb_366
GTAGTTAGCCGTGGCTTTCTGATTAGGTACCGTCAAGACGTGCATAGTTACTTACACATTTGTTCTTCCCTAATAACAGAGTTTTACGATCCGAAGACCTTCATCACTCACGCGGCGTTGCTCCGTCAGGCTTTCGCCCATTGCGGAAGATTCCCTACTGCTGCCTCCCGTAGGAGTCTGGACCGTGTCTCAGTTCCAGTGTGGCCGATCACCCTCTCAGGGTCGGCTACGCATCGTTGCCTTGGTAAGCCGTTACCTTACAACTAGCTAATGCGACGCGGATCCATCTATAAGTGACAGCAAAACCGTCTTTCACTATTGAACCATGCGGTTCAATATATTATCCGGTATTAGCTCCGGT
>n.538.6.bb_367
GGAGTTAGCCGGTGCTTCTTCTGCGGGTAACGTCAATCGACAAGGTTATTAACCTTATCGCCTTCCTCCCCGCTGAAAGTACTTTACAACCCGAAGGCCTTCTTCATACACGCGGCATGGCTGCATCAGGCTTGCGCCCATTGTGCAATATTCCCACTGCTGCCTCCCGTAGGAGTCTGGACCGTGTCTCAGTTCCAGTGTGGCTGGTCATCCTCTCAGACCAGCTAGGGATCGTCGCCTAGGTGACGCCGTTACCCACCTACTAGCTAATCCCATCTGGGCACATC
>n.538.6.bb_368
GGAGTTAGCCGGTGCTTCTTCTGCGGGTAACGTCAATTGCTGAGGTTATTAACCTCAACACCTTCCTCCCCGCTGAAAGTACTTTACAACCCGAAGGCCTTCTTCATACACGCGGCATGGCTGCATCAGGCTTGCGCCCATTGTGCAATATTCCCCACTGCTGCCTCCCGTAGGAGTCTGGACCGTGTCTCAGTTCCAGTGTGGCTGGGTCATCCTCTCAGACCTGCTAGGGATCGTCGCCTAGGTGAGCCGTTACCCACCTACTAGCTAATCCCATCTGGGCACATCTGATGGCAAGAGGCCCGAAGGTCCCCCTCTTTGGTCTTGCGACGTTATGCGGTATTAGCTACCGTTTCCAGTAGTTACT
>n.538.6.bb_369
GTAGTTAGCCGGTGCTTCTTCTGCAGGTACCGTCACCACAAGCTTCGCCCCTGCTGAAAGCGGTTTACAACCCGAAGGCCGTCATCCCGCACGCGGCGTTGCTGCATCAGGCTTCCGCCCATTGTGCAATATTCCCCACTGCTGCCTCCCGTAGGAGTCTGGGCCGTATCTCAGTCCCAATGTGGCCGGTCGCCCTCTCAGGCCGGCTACCCGTCAAAGCCTTGGTAAGCCACTACCCCACCAACAAGCTGATAAGCCGCGAGTCCATCCAA
>n.538.6.bb_370
GTAGTTAGTCGTGGCTTTCTGATTAGGTACCGTCAAGACGTGCATAGTTACCTACACATTTGTTCTTCCCTAATAACAGAGTTTTACGATCCGAAGACCTTCATCACTCACGCGGCGTTGCTCCGTCAGGCTTTCGCCCATTGCGGAAGATTCCCTACTGCTGCCTCCCGTAGGAGTCTGGACCGTGTCTCAGTTCCAGTGTGGCCGATCACCCTCTCAGGGTCGGCTACGCATCGTCGCCTTGGTAAGCCGTTACCTTACCAACTAGCTAATGCGGCGCGGATCCATCTATAAGTGACTAGCAAAACCGTCTTTCACTATTGAACCATGCGGTTCAATATATTATCCGGTATTAGCTCCGGT
>n.538.6.bb_371
GTAGTTAGCCGGTGCTTCTTCTGCAGGTACCGTCACCACAAGCTTCGCCCTGCTGAAAGCGGTTTACAACCCGAAGGCCGTCATCCCGCACGCGGCGTTGCTGCATCAGGCTTCCGCCCATTGTGCAATATTCCCCACTGCTGCCTCCCGTAGGAGTCTGGGCCGTATCTCAGTCCCAATGTGGCCGGTCACCCTCTCAGGCCGGCTACCCGGTNAAAGCCTTGGTAAGCCACTACCCCACCAACAAGCTGATAAGCCGCGAGTCCATCCA
>n.538.6.bb_372
GAAGTTAGCCGGTGCTTCTTCTGCGGGTAACGTCAATTGCTGAGGTTATTAACCTCAACACCTTCCTCCCCGCTGAAAGTACTTTACAACCCGAAGGCCTTCTTCATACACGCGGCATGGCTGCATCAGGCTTGCGCCCATTGTGCAATATTCCCCACTGCTGCCTCCCGTAGGAGTCTGGACCGTGTCTCAGTTCCAGTGTGGCTGGTCATCCTCTCAGACCAGCTAGGGATCGTCGCCTAGGTGAGCCGTTACCCCACCTACTAGCTAATCCCATCTGGGCACATCTGATGGC
>n.538.6.bb_373
GTAGTTAGCCGTGGCTTTCTGATTAGGTACCGTCAAGACGTGCATAGTTACTTACACATTTGTTCTTCCCTAATAACAGAGTTTTACGATCCGAAGACCTTCATCACTCACGCGGCGTTGCTCCGTCAGGCTTTCGCCCATTGCGGAAGATTCCCTACTGCTGCCTCCCGTAGGAGTCTGGACCGTGTCTCAGTTCCAGTGTGGCCGATCACCCTCTCAGGGTCGGCTACGCATCGTTGCCTTGGTAAGCCGTTACCTTACCAACTAGCTAATGCGGCGCGGATCCATCTATAAGTGACAGCAAAACCGTCTTTCACTATTGAACCATGCGGTTCAATATATTATCCGGTATT
>n.538.6.bb_374
GTAGTTAGCCGTGGCTTTCTGATTAGGTACCGTCAAGACGTGCATAGTTACTTACACATTTGTTCTTCCCTAATAACAGAGTTTTACGATCCGAAGACCTTCATCACTCACGCGGCGTTGCTCCGTCAGGCTTTCGCCCATTGCGGAAGATTCCCTACTGCTGCCTCCCGTAGGAGTCTGGACCGTGTCTCAGTTCCAGTGTGGCCGATCACCCTCTCAGGGTCGGCTACGCATCGTTGCCTTGGTAAGCCGTTACCTTACCAACTAGCTAATGCGGCGCGGATCCATCTATAAGTGACAGCAAAACCGTCTTTCACTATTGAACCATGCGGTTCAATATATTATCCGGTATTAGCTCCGGTTTCCCGAAGTTATCCCAGTCTTATAGGTAGGTTATCCCACCGTGTTACCTCACCCCGTCCCGCCCGCTAACGTCAGAGAAGCAAGCCTCCCTCGTCTGTTCGCTCGACTTG
>n.538.6.bb_375
GGAGTTAGCCGGTGCTTCTTCTGCGGGTAACGTCAATCGACAAGGTTATTAACCTTATCGCCTTCCTCCCCGCTGAAAGTACTTTACAACCCGAAGGCCTTCTTCATACACGCGGCATGGCTGCATCAGGCTTGCGCCCATTGTGCAATATTCCCCACTGCTGCCTCCCGTAGGAGTCTGGACCGTGTCTCAGTTCCAGTGTGGCTGGTCATCCTCTCAGACCAGCTAGGGATCGTCGCCTAGGTGAGCCGTTACCCACCTACTAGCTAATCCCATCTGGGCACATCTGATGGCAAGAGGCCCGAAGGTCCCCTCTTTGGTCTTGCGACGTTATGCGGTAT
>n.538.6.bb_376
GTAGTTAGCCGTGGCTTTCTGATTAGGTACCGTCAAGACGTGCATAGTTACTTACACATTTGTTCTTCCCTAATAACAGAGTTTTACGATCCGAAGACCTTCATCACTCACGCGGCGTTGCTCCGTCAAGCTTTCGCCCATTGCGGAAGATTCCCTACTGCTGCCTCCCGTAGGAGCCTGGACCATGTCTCAGTTCCAGTGTGGCCGATCACCCTCTCAGGTCGCTACGCATCGTTGCCTTGGTAAGCCATTACCTTACCAACTAGCTAATGCGGCGCGGATCCATCTATAAGTGACAGCAAAACCGTCTTTCACTATTGAACCATGCGGTTCAATATATTATCCGGTATTAGCTCCGGTTTCCCGAAGTTATCCCAGTCTTATAGGTAGGTTATCCCACGTGT
>n.538.6.bb_377
GTAGTTAGCCGTGGCTTTCTGATTAGGTACCGTCAAGACGTGCATAGTTACTTACACATTTTGTTCCTTCCCTAATAACAGAGTTTTTACGATCCGAAGACCTTCATCACTCACGCGGCGTTGCTCCGTCAGGCTTTCGCCCATTGCGGAAGATTCCCTACTGCCTGCCTCCCGTAGGAGTCTGGACCGTGTCTCAGTTCCAGTGTGGCCGATCACCCTNTCAGGGTCGGCTACGCATCGTTGCCTTGGTAAGCCGTTACCTTA
>n.538.6.bb_378
GTAGTTAGCCGTGGCTTTCTGATTAGGTACCGTCAAGACGTGCAGTAGTTACTTACACATTTGTTCTTCCCTAATAACAGAGTTTTACGATCCGAAGACCTTCATCACTCACGCGGCGTTGCTCCGTCAGGCTTTCGCCCATTGCGGAAGATTCCCTACTGCTGCCTCCCGTAGGAGTCTGGACCGTGTCTCAGTTCCAGTGTGGCCGATCACCCTNTCAGGTCGGCTACGCATCGTGCCTTGGTAAGCCGTTACCTTACCAACTAGCTAATGCGGCGCGGATCCATCTATAAGTGACAGCAAAACCGTCTTTCACTATTGAACCATGCGGTTCAATATATTAT
>n.538.6.bb_379
GTAGTTAGCCGTGGCTTTCTGATTAGGTACCGTCAAGACGTGCATAGTTACTTACACATTTGTTCTTCCCTAATAACAGAGTTTTACGATCCGAAGACCTTCATCACTCACGCGGCGTTGCTCCGTCAGGCTTTCGCCCATTGCGGAAGATTCCCTACTGCTGCCTCCCGTAGGAGTCTGGACCGTGTCTCAGTTCCAGTGTGGCCGATCACCCTCTCAGGGTCGGCTACGCATCGTTGCCTTGGTAAGCCGTTACCTTACCAACTAGCTAATGCGGCGCGGATCCATCTATAAGTGACAGCAAAACCGTCTTTCACTATTGAACCATGCGGTTCAATATATTATCCGGTATTAGCTCCGG
>n.538.6.bb_380
GGAGTTAGCCGGTGCTTCTTCTGCGGGTAACGTCAATCGACAAGGTTATTAACCTTATCGCCTTCCTCCCCGCTGAAAGTACTTTACAACCCGAAGGCCTTCTTCATACACGCGGCATGGCTGCATCAGGCTTGCGCCCATTGTGCAATATTCCCCACTGCTGCCTCCGTAGGAGTCTGGACCGTGTCTCAGTTCCAGTGTGGCTGGTCATCCTCTCAGACCAGCTAGGGATCGTCGCCTAGGTGAGCCGTTACCCACCTACTAGCTAATCCCATCTGGGCACATCTGATG
>n.538.6.bb_381
GTAGTTAGCCGTGGCTTTCTGATTAGGTACCGTCAAGACGTGCATAGTTACTTACACATTTATTCTTCCCTAATAACAGAGTTTTACGATCCGAAGACCTTCATCACTCACGCGGCGTTGCTCCGTCAGGCTTTCGCCCATTGCGGAAGATTCCCTACTGCTGCCTCCCGTAGGAGTCTGGACCGTGTCTCAGTTCCAGTGTGGCCGATCACCCTCTCAGGGTCGGCTACGCATCGTTGCCTTGGTAAGCCGTTACCTTACCAACTAGCTAATGCGGCGCGGATCCATCTATAAGTGACAGCAAAACCGTCTTTCACTATTGAACCATGCGGTTCAATATATTATCCGGTATTAGCTCCGGT
>n.538.6.bb_382
GTAGTTAGCCGGTGCTTCTTCTGCAGGTACCGTCACCACAAGCTTCGCCCCTGCTGAAAGCGGTTTACAACCCGAAGGCCGTCATCCCGCACGCGGCGTTGCTGCATCAGGCTTCCGCCCATTGTGCAATATTCCCCACTGCTGCCTCCCGTAGGAGTCTGGGCCGTATCTCAGTCCCAATGTGGCCGGTCGCCCTCTCAGGCCGGCTACCCGTCAAAGCCTTGGTAAGCCACTACCCACCAACAAGCTGATAAGCCGCGAGTCCATCCAAAACCGCCGAAGCTTTCCAACCCCACCATGCAGCAAGGATTCCTATCCGGTAT
>n.538.6.bb_383
GTAGTTAGCCGGTGCTTCTTCTGCAGGTACCGTCACCACAAGCTTCGCCCCTGCTGAAAGCGGTTTACAACCCGAAGGCCGTCATCCCGCACGCGGCGTTGCTGCATCAGGCTTCCGCCCATTGTGCAATATTCCCCACTGCTGCCTCCCGTAGGAGTCTGGGCCGTATCTCAGTCCCAATGTGCCGGTCGCCCTCTCAGGCCGGCTACCCGTCAAAGCCTTGGTAAGCCACTACCCCACCAACAAGCTGATAAGCCGCGAGTCCATCCAAAACCGCCGAAGCTTTCCAACCCCACCATGCAGCAAGGATTCCTATCCGGTATTAGCCCCAG
>n.538.6.bb_384
GTAGTTAGCCGTGGCTTTCTGATTAGGTACCGTCAAGACGTGCATAGTTACTTACACATTTGTTCTTCCCTAATAACAGAGTTTTACGATCCGAAGACCTTCATCACTCACGCGGCGTTGCTCCGTCAGGCTTTCGCCCATTGCGGAAGATTCCCTACTGCTGCCTCCCGTAGGAGTCTGGACCGTGTCTCAGTTCCAGTGTGGCCGATCACCCTCTCAGGGTCGGCTACGCATCGTTGCCTTGGTAAGCCGTTACCTTACCAACTAGCTAATGCGGCGCGGATCCATCTATAAGTGACAGCAAAACCGTCTTTCACTATTGAACCATGCGGTTCAATATATTATCCGGTATT
>n.538.6.bb_385
GTAGTTAGCCGTGGCTTTCTGATTAGGTACCGTCAAGACGTGCATAGTTACTTACACATTTGTTCTTCCCTAATAACAGAGTTTTACGATCCGAAGACCTTCATCACTCACGCGGCGTTGCTCCGTCAGGCTTTCGCCCATTGCGGAAGATTCCCTACTGCTGCCTCCCGTAGGAGTCTGGACCGTGTCTCAGTTCCAGTGTGGCCGATCACCCTNTCAGGGTCGGCTACGCATCGTTGCCTTGGTAAGCCGTTACCTTACCAACTAGCTAATGCGGCGCGGATCCATCTATAAGTGAC
>n.538.6.bb_386
GTAGTTAGCCGGTGCTTCTTCTGCAGGTACCGTCACCACAAGCTTCGCCCCTGCTGAAAGCGGTTTACAACCCGAAGGCCGTCATCCCGCACGCGGCGTTGCTGCATCAGGCTTCCGCCCATTGTGCAATATTCCCCACTGCTGCCTCCCGTAGGAGTCTGGGCCGTATCTCAGTCCCAATGTGGCCGGTCGCCCTCTCAGGCCGGCTACCCGTCAAAGCCTTGGTAAGCCACTACCCACCAACAAGCTGATAAGCCGCGAGTCCATCCA
>n.538.6.bb_387
GGAGTTAGCCGGTGCTTCTTCTGCGGGTAACGTCAATCGACACGGTTATTAACCGCATCGCCTTCCTCCCCGCTGAAAGCACTTTACAACCCGAAGGCCTTCTTCATACACGCGGCATGGCTGCATCAGGCTTGCGCCCATTGTGCAATATTCCCCACTGCTGCCTCCCGTAGGAGTCTGGACCGTGTCTCAGTTCCAGTGTGGCTGGGTCATCCTCTCAGACCAGCTAGGGGTCGTCGCCTAGGTGAGCCGTTACCCCACCTACTAGCTAATCCTATCTGGGTTCATCTGATG
>n.538.6.bb_388
GTAGTTAGCCGTGGCTTTCTGATTAGGTACCGTCAAGACGTGCATAGTTACTTACACATTTGTTCTTCCCTAATAACAGAGTTTTACGATCCGAAGACCTTCATCACTCACGCGGCGTTGCTCCGTCAGGCTTTCGCCCATTGCGGAAGATTCCCTACTGCTGCCTCCCGTAGGAGTCTGGACCGTGTCTCAGTTCCAGTGTGGCCGATCACCCTCTCAGGGTCGGCTACGCATCGTCGCCTTGGTAAGCCGTTACCTTACCACTAGCTAATGCGGCGCGGATCCATCTATAAGTGA
>n.538.6.bb_389
GTAGTTAGCCGTGGCTTTCTGATTAGGTACCGTCAAGACGTGCATAGTTACTTACACATTTGTTCTTCCCTAATAACAGAGTTTTACGATCCGAAGACCTTCATCACTCACGCGGCGTTGCTCCGTCAGGCTTTCGCCCATTGCGGAAGATTCCCTACTGCTGCCTCCCGTAGGAGTCTGGACCGTGTCTCAGTTCCAGTGTGGCCGATCACCCTCTCAGGGTCGGCTACGCATCGTCGCCTTGGTAAGCCGTTACCTTACCAACTAGCTAATGCGGCGCGGATCCATCTATAAGTGACAGCAAAACCGTCTTTCACTATTGAACCATGCGGTTCAATATATTATCCGGTATTAGCTCCGGTTTCCCGAAGTTATCCCAGTCTTATAGGTAGGTTATCCCACGTGTTACCTCA
>n.538.6.bb_390
GGAGTTAGCCGGTGCTTCTTCTGCGGGTAACGTCAATCGACAAGGTTATTAACCTTATCGCCTTCCTCCCCGCTGAAAGTACTTTACAACCCGAAGGCCTTCTTCATACACGCGGCATGGCTGCATCAGGCTTGCGCCCATTGTGCAATATTCCCCACTGCTGCCTCCGTAGGAGTCTGGACCGTGTCTCAGTTCCAGTGTGGCTGGTCATCCTCTCAGACCAGCTAGGGATCGTCGCCTAGGTGAGCCGTTACCCACCTACTAGCTAATCCCATCTGGGCACATCTGATGGCAAGAGGCCCGAAGGT
>n.538.6.bb_391
GTAGTTAGCCGTGGCTTTCTGATTAGGTACCGTCAAGACGTGCATAGTTACTTACACATTTGTTCTTCCCTAATAACAGAGTTTTACGATCCGAAGACCTTCATCACTCACGCGGCGTTGCTCCGTCAGGCTTTCGCCCATTGCGGAAGATTCCCTACTGCTGCCTCCCGTAGGAGTCTGGACCGTGTCTCAGTTCCAGTGTGGCCGATCACCCTCTCAGGGTCGGCTACGCATCGTTGCCTTGGTAAGCCGTTACCTTACCAACTAGCTAATGCGGCGCGGATCCATCTATAAGTGACAGCAAAACCGTCTTTCACTATTGAACCATGCGGTTCAATATATTATCCGGTATTAG
>n.538.6.bb_392
GTAGTTAGCCGTGGCTTTCTGATTAGGTACCGTCAAGACGTGCATAGTTACTTACACATTTGTTCTTCCCTAATAACAGAGTTTTACGATCCGAAGACCTTCATCACTCACGCGGCGTTGCTCCGTCAGGCTTTCGCCCATTGCGGAAGATTCCCTACTGCTGCCTCCCGTAGGAGTCTGGACCGTGTCTCAGTTCCAGTGTGGCCGATCACCCTCTCAGGGTCGGCTACGCATCGTTGCCTTGGTAAGCCGTTACCTTACCAACTAGCTAATGCGACGCGGATCCATCTATAAGTGACAGCAAAACCGTCTTTCACTATTGAACCATGCGGTTCAATATATTATCCGGTATTAGCTCCGGT
>n.538.6.bb_393
GTAGTTAGCCGTGGCTTTCTGATTAGGTACCGTCAAGACGTGCATAGTTACTTACACATTTGTTCTTCCCTAATAACAGAGTTTTACGATCCGAAGACCTTCATCACTCACGCGGCGTTGCTCCGTCAGGCTTTCGCCCATTGCGGAAGATTCCCTACTGCTGCCTCCCGTAGGAGTCTGGACCGTGTCTCAGTTCCAGTGTGGCCGATCACCCTCTCAGGGTCGGCTACGCATCGTCGCCTTGGTAAGCCGTTACCTTACCAACTAGCTAATGCGGCGCGGATCCATCTATAAGTGACAGCAAAAACCGTCTTTCACTATTGAACCATGCGGTTCAATATATTATCCGGTATTAGCTCCGGTTTCCCGAAGT
>n.538.6.bb_394
GGAGTTAGCCGGTGCTTCTTCTGCGGGTAACGTCAATCGACAAGGTTATTAACCTTATCGCCTTCCTCCCCGCTGAAAGTACTTTACAACCCGAAGGCCTTCTTCATACACGCGGCATGGCTGCATCAGGCTTGCGCCCATTGTGCAATATTCCCCACTGCTGCCTCCGTAGGAGTCTGGACCGTGTCTCAGTTCCAGTGTGGCTGGTCATCCTCTCAGACCAGCTAGGGATCGTCGCCTAGGTGAGCCGTTACCCACCTACTAGCTAATCCCATCTGGGCACATCTGATGGC
>n.538.6.bb_395
GTAGTTAGCCGGTGCTTCTTCTGCAGGTACCGTCACCACAAGCTTCGCCCCTGCTGAAAGCGGTTTACAACCCGAAGGCCGTCATCCCGCACGCGGCGTTGCTGCATCAGGCTTCCGCCCATTGTGCAATATTCCCCACTGCTGCCTCCCGTAGGAGTCTGGGCCGTATCTCAGTCCCAATGTGGCCGGTCGCCCTCTCAGGCCGGCTACCCGTCAAAGCCTTGGTAAGCCACTACCCACCAACAAGCTGATAAGCCGCGAGTCCATCCAAAACCGCCGAAG
>n.538.6.bb_396
GTAGTTAGCCGTGGCTTTCTGATTAGGTACCGTCAAGACGTGCATAGTTACTTACACATTTGTTCTTCCCTAATAACAGAGTTTTACGATCCGAAGACCTTCATCACTCACGCGGCGTTGCTCCGTCAGGCTTTCGCCCATTGCGGAAGATTCCCTACTGCTGCCTCCCGTAGGAGTCTGGACCGTGTCTCAGTTCCAGTGTGGCCGATCACCCTCTCAGGGTCGGCTACGCATCGTTGCCTTGGTAAGCCGTTACCTTACCAACTAGCTAATGCGGCGCGGATCCATCTATAAGTGACAGCAAAACCGTCTTTCACTATTGAACCATGCGGTTCAATATATTATCCGGTATTAGCTCCGGTTTCCCGAAGTTTATCCCAGTCTTATAGGTAGGTTATCCCACCGTGTTACCTCACCCCGTCCCGCCCGCTAACGTCAGAGGGAGCAAGCTCCCTCGTCTGTTCGCTCGACTTGCCATGTATTAGGGCACGCCG
>n.538.6.bb_397
GTAGTTAGCCGTGGCTTTCTGATTAGGTACCGTCAAGACGTGCATAGTTACTTACACATTTATTCTTCCCTAATAACAGAGTTTTACGATCCGAAGACCTTCATCACTCACGCGGCGTTGCTCCGTCAGGCTTTCGCCCATTGCGGAAGATTCCCTACTGCTGCCTCCCGTAGGAGTCTGGACCGTGTCTCAGTTCCAGTGTGGCCGATCACCCTCTCAGGGTCGGCTACGCATCGTTGCCTTGGTAAGCCATTACCTTACCAACTAGCTAATGCGGCGCGGATCCATCTATAAGTGACAGCAAAACCGTCTTTCACTATTGAACCATGCGGTTCAATATATTATCCGGTATTAGCTCCGGTTTCCCGAAGTTATCCCAGTCTTATA
>n.538.6.bb_398
GTAGTTAGCCGGTGCTTCTTCTGCAGGTACCGTCACCACAAGCTTCGCCCCTGCTGAAAGCGGTTTACAACCCGAAGGCCGTCATCCCGCACGCGGCGTTGCTGCATCAGGCTTCCGCCCATTGTGCAATATTCCCCACTGCTGCCTCCCGTAGGAGTCTGGGCCGTATCTCAGTCCCAATGTGGCCGGTCGCCCTCTCAGGCCGGCTGCCCGTCAAAGCCTTGGTAAGCCACTACCCACCAACAAGCTGATAAGCCGCGAGTCCAT
>n.538.6.bb_399
GTAGTTAGCCGTGGCTTTCTGATTAGGTACCGTCAAGACGTGCGTAGTTACTTACACATTTGTTCTTCCCTAATAACAGAGTTTTACGATCCGAAGACCTTCATCACTCACGCGGCGTTGCTCCGTCAGGCTTTCGCCCATTGCGGAAGATTCCCTACTGCTGCCTCCCGTAGGAGTCTGGACCGTGTCTCAGTTCCAGTGTGGCCGATCACCCTCTCAGGGTCGGCTACGCATCGTCGCCTTGGTAAGCCGTTACCTTACCAACTAG
>n.538.6.bb_400
GTAGTTAGCCGTGGCTTTCTGATTAGGTACCGTCAAGACGTGCATAGTTACTTACACATTTGTTCTTCCCTAATAACAGAGTTTTACGATCCGAAGACCTTCATCACTCACGCGGCGTTGCTCCGTCAGGCTTTCGCCCATTGCGGAAGATTCCCTACTGCTGCCTCCCGTAGGAGTCTGGACCGTGTCTCAGTTCCAGTGTGGCCGATCACCCTCTCAGGGTCGGCTACGCATCGTTGCCTTGGTAAGCCGTTACCTTACCAACTAGCTAATGCGGCGCGGATCCATCTATAAGTGACAGCAAAACCGTCTTTCACTATTGAACCATGCGGTTCAATATATTATCCCGGTATTAGCTCCGGTTTCCCGAAGTTATCCCAGTCTTATAGGTAGGTTATCCCACCGTGTTACCTCACCCCGTCCCGCCCGCTAACGTCAGAGGAGCAAGCTCCCTCGTCTGTTCGCTCGACTTGCATGTATTAGGGCACGCCGCCAG
>n.538.6.bb_401
GTAGTTAGCCGTGGCTTTCTTGATTAGGTACCGTCAAGACGTGCATAGTTACTTACACATTTGTTTCTTCCCTAATAACAGAGTTTTACGATCCGAAGACCTTCATCACTCACGCGGCGTTGCTCCGTCAGGCTTTCGCCCATTGCGGAAGATTCCCTACTGCTGCCTCCCGTAGGAGTCTGGACCGTGTCTCAGTTCCAGTGTGGCCGATCACCCTCTCAGGGTCGGCTACGCATCGTTGCCTTGGTAAGCCGTTACCTTACCAACCAGCTAATGCGGCGCGGATCCATCT
>n.538.6.bb_402
GTAGTTAGCCGGTGCTTCTTCTGCAGGTACCGTCACCACAAGCTTCGCCCCTGCTGAAAGCGGTTTACAACCCGAAGGCCGTCATCCCGCACGCGGCGTTGCTGCATCAGGCTTCCGCCCATTGTGCAATATTCCCCACTGCTGCCTCCCGTAGGAGTCTGGGCCGTATCTCAGTCCCAATGTGGCCGGTCGCCCTCTCAGGCCGGCTACCCGGTCAAAGCCTTGGTAAGCCACTACCCACCAACAAGCTGATAAGCCGCGAGTCCATCC
>n.538.6.bb_403
GTAGTTAGCCGTGGCTTTCTGATTAGGTACCGTCAAGACGTGCATAGTTACTTACACATTTATTCTTCCCTAATAACAGAGTTTTACGATCCGAAGACCTTCATCACTCACGCGGCGTTGCTCCGTCAGGCTTTCGCCCATTGCGGAAGATTCCCTACTGCTGCCTCCCGTAGGAGTCTGGACCGTGTCTCAGTCCCAATGTGGCCGGTCGCCCTCTCAGGGCCGGGCTACCCCGTCAAAGCCTTGGTAAGCCACTACCCACCAACAAGCTGATAAGCCGCGAGTCCATCCAAAACCGCCGAAGCTTTCCAACCCCACCATGCAGCAAGGATTCCTATCCGGTATTAG
>n.538.6.bb_404
GTAGTTAGCCGTGGCTTTCTGATTAGGTACCGTCAAGACGTGCATAGTTACTTACACATTTATTCTTCCCTAATAACAGAGTTTTACGATCCGAAGACCTTCATCACTCACGCGGCGTTGCTCCGTCAGGCTTTCGCCCATTGCGGAAGATTCCCTACTGCTGCCTCCCGTAGGAGTCTGGACCGTGTCTCAGTTCCAGTGTGGCCGATCACCCTCTCAGGGTCGGCTACGCATCGTGCCTTGGTAAGCCGTTACCTTACCAACTAGCTAATGCGGCGCGGATCCATCTATAAGTGACAGCAAAACCGTCTTTCACTATTGAACCATGCGGTTCAATATATTATCCGGTATTAGCTCCGGTTTCCCGAAGTTATCCCAGTCTTATAGGTAGGTTATCCCACGTGTTACCTCACCCCGTCCCGCCCGCTAACGTCAGAGGAGCAAGCTCCCTCGTCTGTTCGCTCGACTTGCATGTATTAGGGCACGCCG
>n.538.6.bb_405
GTAGTTAGCCGGTGCTTCTTCTGCAGGTACCGTCACCACAAGCTTCGCCCCTGCTGAAAGCGGTTTACAACCCGAAGGCCGTCATCCCGCACGCGGCGTTGCTGCATCAGGCTTCCGCCCATTGTGCAATATTCCCCACTGCTGCCTCCCGTAGGAGTCTGGGCCTATCTCAGTCCCAATGTGGCCGGTCGCCCTCTCAGGCCGGCTACCCGTNAAAGCCTTGGTAACGCCACTACCCACCAACAAGCTGATAAGCCGCGAGTCCA
>n.538.6.bb_406
GTAGTTAGTCGGTGCTTCTTCTGCAGGTACCGTCACCACAAGCTTCGCCCCTGCTGAAAGCGGTTTACAACCCGAAGGCCGTCATCCCGCACGCGGCGTTGCTGCATCAGGCTTCCGCCCATTGTGCAATATTCCCCACTGCTGCCTCCCGTAGGAGTCTGGGCCGTATCTCAGTCCCAATGTGGCCGGTCGCCCTCTCAGGCCGGCTACCCGTCAAAGCCTTGGTAAGCCACTACCCCACCAACAAGCTGATAAGCCGCGAGTCCATCCAAAACCGC
>n.538.6.bb_407
GTAGTTAGCCGTGGCTTTCTGATTAGGTACCGTCAAGACGTGCATAGTTACTTACACATTTGTTCTTCCCTAATAACAGAGTTTTACGATCCGAAGACCTTCATCACTCACGCGGCGTTGCTCCGTCAGGCTTTCGCCCATTGCGGAAGATTCCCTACTGCTGCCTCCCGTAGGAGTCTGGACCGTGTCTCAGTTCCAGTGTGGCCGATCACCCTCTCAGGGTCGGCTACGCATCGTTGCCTTGGTAAGCCGTTACCTTACCAACTAGCTAATGCGGCGCGGATCCATCTATAAGTGACAGCAAAACCGTCTTTCACTATTGAACCATGCGGTTCAATATATTATCCGGTATTAGCTCCGGTTTCCGAAGTTATCCCAGTCTTA
>n.538.6.bb_408
GTAGTTAGCCGGTGCTTCTTCTGCAGGTACCGTCACCACAAGCTTCGCCCCTGCTGAAAGCGGTTTACAACCCGAAGGCCGTCATCCCGCACGCGGCGTTGCTGCATCAGGCTTCCGCCCATTGTGCAATACTTCCCCACTGCTGCCTCCCGTAGGAGTCTGGGCCGTATCTCAGTCCCAATGTGGCCGGTCGCCCTCTCGCG
>n.538.6.bb_409
GTAGTTAGCCGTGGCTTTCTGATTAGGTACCGTCAAGACGTGCATAGTTACTTACACATTTGTTCTTCCCTAATAACAGAGTTTTACGATCCGAAGACCTTCATCACTCACGCGGCGTTGCTCCGTCAGGCTTTCGCCCATTGCGGAAGATTCCCTACTGCTGCCTCCCGTAGGAGTCTGGACCGTGTCTCAGTTCCAGTGTGGCCGATCACCCTCTCAGGGTCGGCTACGCATCGTTGCCTTGGTAAGCCGTTACCTTACAACTAGCTAATGCGGCGCGGATCCATCTATAAGTGAC
>n.538.6.bb_410
GGAGTTAGCCGGTGCTTCTTCTGCGGGTAACGTCAATCGACAAGGTTATTAACCTTATCGCCTTCCTCCCCGCTGAAAGTACTTTACAACCCGAAGGCCTTCTTCATACACGCGGCATGGCTGCATCAGGCTTGCGCCCATTGTGCAATATTCCCCACTGCTGCCTCCCGTAGGAGTCTGGACCGTGTCTCAGTTCCAGTGTGGCTGGTCATCCTCTCAGACCAGCTAGGGATCGTCGCCTAGGTGAGCCGTTACCCACCTACTAGCTAATCCCATCTGGGCACATCTGATGGC
>n.538.6.bb_411
GTAGTTAGCCGTGGCTTTCTGATTAGGTACCGTCAAGACGTGCATAGTTACTTACACATTTGTTCTTCCCTAATAACAGAGTTTTACGATCCGAAGACCTTCATCACTCACGCGGCGTTGCTCCGTCAGGCTTTCGCCCATTGTGCAATATTCCCCACTGCTGCCTCCCGTAGGAGTCTGGGCCGTATCTCAGTCCCAATGTGGCCGGTCGCCCTCTCAGGGCCGGCTACCCGTCAAAGCCTTGGTAAGCCACTACCCCACCAACAAGCTGATAAGCCGCGAGTCCATCCAAAACCGCCGAAGC
>n.538.6.bb_412
GTAGTTAGCCGTGGCTTTCTGATTAGGTACCGTCAAGACGTGCATAGTTACTTACACATTTGTTCTTCCCTAATAACAGAGTTTTACGATCCGAAGACCTTCATCACTCACGCGGCGTTGCTCCGTCAGGCTTTCGCCCATTGCGGAAGATTCCCTACTGCTGCCTCCCGTAGGAGTCTGGACCGTGTCTCAGTTCCAGTGTGGCCGATCACCCTCTCAGGGTCGGCTACGCATCGTTGCCTTGGTAAGCCGTTACCTTACCAACTAGCTAATGCGGCGCGGATCCATCTATAAGTGACAGCAAAACCGTCTTTCACTATTGAACCATGCGGTTCAATATATTATCCGGTATTAGCTCCGGT
>n.538.6.bb_413
GGAGTTAGCCGGTGCTTCTTCTGCGGGTAACGTCAATTGCTGAGGTTATTAACCTCAACACCTTCCTCCCCGCTGAAAGTACTTTACAACCCGAAGGCCTTCTTCATACACGCGGCATGGCTGCATCAGGCTTGCGCCCATTGTGCAATATTCCCCACTGCTGCCTCCCGTAGGAGTCTGGACCGTGTCTCAGTTCCAGTGTGGCTGGGTCATCCTCTCAGACCAGCTAGGGGATCGTCGCCTAGGTGAGCCGT
>n.538.6.bb_414
GTAGTTAGCCGTGGCTTTCTGATTAGGTACCGTCAAGACGTGCATAGTTACTTACACATTTGTTCTTCCCTAATAACAGAGTTTTACGATCCGAAGACCTTCATCACTCACGCGGCGTTGCTCCGTCAGGCTTTCGCCCATTGCGGAAGATTCCCTACTGCTGCCTCCCGTAGGAGTCTGGACCGTGTCTCAGTTCCAGTGTGGCCGATCACCCTCTCAGGGTCGGCTACGCATCGTTGCCTTGGTAAGCCGTTACCTTACCAACTAGCTAATGCGGCGCGGATCCATCTATAAGTGACAGCAAAACCGTCTTTCACTATTGAACCATGCGGTTCAATATATTATCCGGTAT
>n.538.6.bb_415
GTAGTTAGCCGTGGCTTTCTGATTAGGTACCGTCAAGACGTGCATAGTTACTTACACATTTGTTCTTCCCTAATAACAGAGTTTTACGATCCGAAGACCTTCATCACTCACGCGGCGTTGCTCCGTCAGGCTTTCGCCCATTGCGGAAGATTCCCTACTGCTGCCTCCCGTAGGAGTCTGGACCGTGTCTCAGTTCCAGTGTGGCCGATCACCCTCTCAGGGTCGGCTACGCATCGTTGCCTTGGTAAGCCGTTACCTTACCAACTAGCTAATGCGGCGCGGATCCATCTATAAGTGACAGCAAAACCGTCTTTCACTATTGAACCATGCGGTTCAATATATT
>n.538.6.bb_416
GTAGTTAGCCGTGGCTTTCTGATTAGGTACCGTCAAGACGTGCATAGTTACTTACACATTTGTTCTTCCCTAATAACAGAGTTTTACGATCCGAAGACCTTCATCACTCACGCGGCGTTGCTCCGTCAGGCTTTCGCCCATTGCGGAAGATTCCCTACTGCTGCCTCCCGTAGGAGTCTGGACCGTGTCTCAGTTCCAGTGTGGCCGATCACCCTCTCAGGGTCGGCTACGCATCGTTGCCTTGGTAAGCCGTTACCTTACAACTAGCTAATGCGGCGCGGATCCATCTATAAGTGACAGCAAAACCGTCTTTCACTATTGAACCATGCGGTTCAATATATTATCCGGTATTAGCTCCGGT
>n.538.6.bb_417
GTAGTTAGCCGGTGCTTCTTCTGCAGGTACCGTCACCACAAGCTTCGCCCCTGCTGAAAGCGGTTTACAACCCGAAGGCCGTCATCCCGCACGCGGCGTTGCTGCATCAGGCTTCCGCCCATTGTGCAATATTCCCCACTGCTGCCTCCCGTAGGAGTCTGGGCCGTATCTCAGTCCCAATGTGGCCGTCGCCCTCTCAGGCCGGCTACCCGTCAAAGCCTTGGTAAGCCACTACCCCACCAACAAGCTGATAAGCCGCGAGTCC
>n.538.6.bb_418
GTAGTTAGCCGTGGCTTTCTGATTAGGTACCGTCAAGACGTGCATAGTTACTTACACATTTGTTCTTCCCTAATAACAGAGTTTTACGATCCGAAGACCTTCATCACTCACGCGGCGTTGCTCCGTCAGGCTTTCGCCCATTGCGGAAGATTCCCTACTGCTGCCTCCCGTAGGAGTCTGGACCGTGTCTCAGTTCCAGTGTGGCCGATCACCCTCTCAGGGTCGGCTACGCATCGTTGCCTTGGTAAGCCGTTACCTTACCAACTAGCTAATGCGGCGCGGATCCATCTATAAGTGACAGCAAAACCGTCTTTCACTATTGAACCATGCGGCTCAATATATT
>n.538.6.bb_419
GTAGTTAGCCGTGGCTTTCTGATTAGGTACCGTCAAGACGTGCATAGTTACTTACACATTTGTTCTTCCCTAATAACAGAGTTTTACGATCCGAAGACCTTCATCACTCACGCGGCGTTGCTCCGTCAGGCTTTCGCCCATTGCGGAAGATTCCCTACTGCTGCCTCCCGTAGGAGTCTGGACCGTGTCTCAGTTCCAGTGTGGCCGATCACCCTCTCAGGGTCGGCTACGCATCGTTGCCTTGGTAAGCCGTTACCTTACCAACTAGCTAATGCGGCGCGGATCCATCTATAAGTGACAGCAAAACCGTCTTTCACTATTGAACCATGCGGTTCAATATAT
>n.538.6.bb_420
GTAGTTAGCCGGTGCTTCTTCTGCAGGTACCGTCACCACAAGCTTCGCCCCTGCTGAAAGCGGTTTACAACCCGAAGGCCGTCATCCCGCACGCGGCGTTGCTGCATCAGGCTTCCGCCCATTGTGCAATATTCCCCACTGCTGCCTCCCGTAGGAGTCTGGGCCGTATCTCAGTCCCAATGTGGCCGGTCGCCCTCTCAGGGCCGGCTACCCGTCAAAGCCTTGGTAAGCCACTACCCCACCAACAAGCTGATAAGCCGCGAGTCCATCCAAAACCGCCGAAGCTTTCCAACCCCCACCATGCAGCAAGGATTCCTATCCGGTATTA
>n.538.6.bb_421
GGAGTTAGCCGGTGCTTCTTCTGCGGGTAACGTCAATCGACAAGGTTATTAACCTTATCGCCTTCCTCCCCGCTGAAAGTACTTTACAACCCGAAGGCCTTCTTCATACACGCGGCATGGCTGCATCAGGCTTGCGCCCATTGTGCAATATTCCCCACTGCTGCCTCCCGTAGGAGTCTGGACCGTGTCTCAGTTCCAGTGTGGCTGGGTCATCCTCTCAGACCAGCTAGGGATCGTCGCCTAGGTGAGCCGTTACCCCACCTACTAGCTAATCCCATCTGGGCACATCTGATGGCAAGAGGCCCGAAGGTCCCCCTCTTTGGTCTTGCGACGTTATGCGGTATTAGCTACCGTTTCCAG
>n.538.6.bb_422
GTAGTTAGCCGTGGCTTTCTGATTAGGTACCGTCAAGACGTGCATAGTTACTTACACATTTGTTCTTCCCTAATAACAGAGTTTTACGATCCGAAGACCTTCATCACTCACGCGGCGTTGCTCCGTCAAGCCTTCGCCCATTGCGGAAGATTCCCTACTGCTGCCTCCCGTAGGAGTCTGGACCGTGTCTCAGTTCCAGTGTGGCCGATCACCCTCTCAGGGTCGGCTACGCATCGTTGCCTTGGTAAGCCGTTACCTTACCAACTAGCTAATGCGGCGCGGATCCATCTATAAGTGACAGCAAAACCGTCTTTCACTATTGAACCATGCGGTTCAATATATTATCCGGTATTAGCTCCGGTTTCCGAAGTTATCCCAGTCTTATAGGTAGGTTATC
>n.538.6.bb_423
GTAGTTAGCCGTGGCTTTCTGATTAGGTACCGTCAAGACGTGCATAGTTACTTACACATTTGTTCTTCCCTAATAACAGAGTTTTACGATCCGAAGACCTTCATCACTCACGCGGCGTTGCTCCGTCAGGCTTTCGCCCATTGCGGAAGATTCCCTACTGCTGCCTCCCGTAGGAGTCTGGACCGTGTCTCAGTTCCAGTGTGGCCGATCACCCTCTCAGGGTCGGCTACGCATCGTTGCCTTGGTAAGCCGTTACCTTACCAACTAGCTAATGCGGCGCGGATCCATCTATAAGTGACAGCAAAACCGTCTTTCACTATTGAACCA
>n.538.6.bb_424
GTAGTTAGCCGGTGCTTCTTCTGCAGGTACCGTCACCACAAGCTTCGCCCCTGCTGAAAGCGGTTTACAACCCGAAGGCCGTCATCCCGCACGCGGCGTTGCTGCATCAGGCTTCCGCCCATTGTGCAATATTCCCCACTGCTGCCTCCCGTAGGAGTCTGGGCCGTATCTCAGTCCCAATGTGGCCGGTCGCCCTCTCAGGCCGGCTACCCGTCAAAGCCTTGGTAAGCCACTACCCCACCAACAAGCTGATAAGCCGCGAGTCCATCCAAAACCGCCGAAGCTTTCCAACCCCCACCATGCAGCAAGGATTCCTATCCGGTATTAGCCCCAGTTTCCTGAAGTTATCCCGAAGTCAAAGGGCAGGTTACTCACGTGTTACTCACCCGTTCGCCACTCCGACGTA
>n.538.6.bb_425
GTAGTTAGCCGTGGCTTTCTGATTAGGTACCGTCAAGACGTGCATAGTTACTTACACATTTGTTCTTCCCTAATAACAGAGTTTTACGATCCGAAGACCTTCATCACTCACGCGGCGTTGCTCCGTCAGGCTTTCGCCCATTGCGGAAGATTCCCTACTGCTGCCTCCCGTAGGAGTCTGGACCGTGTCTCAGTTCCAGTGTGGCCGATCACCCTCTCAGGGTCGGCTACGCATCGTTGCCTTGGTAAGCCGTTACCTTACCAACTAGCTAATGCGGCGCGGATCCATCTATAAGTGACAGCAAAACCGTCTTTCACTATTGAACCATGCGGTTCAATATATTATCCGGTATT
>n.538.6.bb_426
GTAGTTAGCCGGTGCTTCTTCTGCAGGTACCGTCACCACAAGCTTCACCCCTGCTGAAAGCGGTTTACAACCCGAAGGCCGTCATCCCGCACGCGGCGTTGCTGCATCAGGCTTCCGCCCATTGTGCAATATTCCCCACTGCTGCCTCCCGTAGGAGTCTGGGCCGTATCTCAGTCCCAATGTGGCCGGTCGCCCTCTCAGGCCGGCTACCCGGTCAAAGCCTTGGTAAGCCACTACCCCACCAACAAGCTGATAAGCCGCGAGTCCATCCAAAACCGCCGAAGCTTTCCAACCCCACCATGCAGCAAGGATTCCTATCCGGTATTAGCCCCAGTTTCCTGAAGTTATCCCGAAGTCAAGGGCAGGTTACTCACGTGTTACTCACCCGT
>n.538.6.bb_427
GGAGTTAGCCGGTGCTTCTTCTGCGGGTAACGTCAATCGACAAGGTTATTAACCTTATCGCCTTCCTCCCCGCTGAAAGTACTTTACAACCCGAAGGCCTTCTTCATACACGCGGCATGGCTGCATCAGGCTTGCGCCCATTGTGCAATATTCCCCACTGCTGCCTCCCGTAGGAGTCTGGACCGTGTCTCAGTTCCAGTGTGGCTGGTCATCCTCTCAGACCAGCTAGGGATCGTCGCCTAGGTGAGCCGTTACCCACCTACTAGCTAATCCCATCTGGGCACATCTGATGGC
>n.538.6.bb_428
GTAGTTAGCCGTGGCTTTCTGATTAGGTACCGTCAAGACGTGCATAGTTACTTACACATTTGTTCTTCCCTAATAACAGAGTTTTACGATCCGAAGACCTTCATCACTCACGCGGCGTTGCTCCGTCAGGCTTTCGCCCATTGCGGAAGATTCCCTACTGCTGCCTCCCGTAGGAGTCTGGACCGTGTCTCAGTTCCAGTGTGGCCGATCACCCTCTCAGGGTCGGCTACGCATCGTTGCCTTGGTGAGCCGTTACCTTACCAACTAGCTAATGCGGCGCGGATCCATCTATAAGTGACAGCAAAACCGTCTTTCACTATTGAACCATGCGGTTCAATATAT
>n.538.6.bb_429
GTAGTTAGCCGGTGCTTCTTCTGCAGGTACCGTCACCACAAGCTTCGCCCCTGCTGAAAGCGGTTTACAACCCGAAGGCCGTCATCCCGCACGCGGCGTTGCTGCATCAGGCTTCCGCCCATTGTGCAATATTCCCCACTGCTGCCTCCCGTAGGAGTCTGGGCCGTATCTCAGTCCCAATGTGGCCGGTCGCCCTCTCAGGCCGGCTACCCGGTCAAAGCCTTGGTAAGCCACTACCCCACCAACAAGCTGATAAGCCGCGAGTCCATCCAAAACCGCCGAAGCTTTCCAACCCCACCATGCAGCAAGGATTCCTATCCGGTATTAGCCCCAGTTTCCTGAAGTTAT
>n.538.6.bb_430
GTAGTTAGCCGGTGCTTCTTCTGCAGGTACCGTCACCACAAGCTTCGCCCCTGCTGAAAGCGGTTTACAACCCGAAGGCCGTCATCCCGCACGCGGCGTTGCTGCATCAGGCTTCCGCCCATTGTGCAATATTCCCCACTGCTGCCTCCCGTAGGAGTCTGGGCCGTATCTCAGTCCCAATGTGGCCGGTCGCCCTCTCAGGCCGGCTACCCGTCAAAGCCTTGGTAAGCCACTACCCCACCAACAAGCTGATAAGCCGCGAGTCCATCCAAAACCGCCGAAGCTTTCCAACCCCACCATGCAGCAAGGATTCCTATCCGGTATTAGCCCCAGTTTCCTGAAGTTATCCCGAAGTCAAAGGGCAGGTTACTCACGTGTTACTCACCCGTTCGCCA
>n.538.6.bb_431
GTAGTTAGCCGGTGCTTCTTCTGCAGGTACCGTCACCACAAGCTTCGCCCCTGCTGAAAGCGGTTTACAACCCGAAGGCCGTCATCCCGCACGCGGCGTTGCTGCATCAGGCTTCCGCCCATTGTGCAATATTCCCCACTGCTGCCTCCCGTAGGAGTCTGGGCCGTATCTCAGTCCCAATGTGGCCGGTCGCCCTCTCAGGCCGGCTACCCGTCAAAGCCTTGGTAAGCCACTACCCCACCAACAAGCTGATAAGCCGCGAGTCCATCCAAAACCGCCG
>n.538.6.bb_432
GTAGTTAGCCGGTGCTTCTTCTGCAGGTACCGTCACCACAAGCTTCGCCCCTGCTGAAAGCGGTTTACAACCCGAAGGCCGTCATCCCGCACGCGGCGTTGCTGCATCAGGCTTCCGCCCATTGTGCAATATTCCCCACTGCTGCCTCCCGTAGGAGTCTGGGCCGTATCTCAGTCCCAATGTGGCCGGTCGCCCTCTCAGGCCGGCTACCCGTCAAAGGCCTTGGTAAGCCACTACCCCACCAACAAGCTGATAAGCCGCGAGTCCATCCAAAACCGCCGAAAGCTTTCCAACCCCACCATGCAGCAAGGATTCCTATCCGGTATTAGCCCCAGTTTCCTGAAGTTATCCCGAAGTCAAGGGCAGGTTACTCACGTGTTACCTCACCCGTTCGCCA
>n.538.6.bb_433
GTAGTTAGCCGGTGCTTCTTCTGCAGGTACCGTCACCACAAGACTTCGCCCCTGCTGAAAGCGGTTTACAACCCGAAGGCCGTCATCCCGCACGCGGCGTTGCTGCATCAGGCTTCCGCCCATTGTGCAATATTCCCCACTGCTGCCTCCCGTAGGAGTCTGGGCCGTATCTCAGTCCCAATGTGGCCGGTCGCCCTCTCAGGCCGGCTA
>n.538.6.bb_434
GGAGTTAGCCGGTGCTTCTTCTGCGGGTAACGTCAATTGCTGAGGTTATTAACCTCAACACCTTCCTCCCCGCTGAAAGTACTTTACAACCCGAAGGCCTTCTTCATACACGCGGCATGGCTGCATCAGGCTTGCGCCCATTGTGCAATATTCCCCACTGCTGCCTCCCGTAGGAGTCTGGACCGTGTCTCAGTTCCAGTGTGGCTGGGTCATCCTCTCAGACCAGCTAGAGATCGTCGCCTAGGTGAGCCGTTACCCACCTACTAGCTAATCCCATCTGGGCACATCTGATGGCAAGAGG
>n.538.6.bb_435
GTAGTTAGCCGTGGCTTTCTGATTAGGTACCGTCAAGACGTGCATAGTTACTTACACATTTGTTCTTCCCTAATAACAGAGTTTTACGATCCGAAGACCTTCATCACTCACGCGGCGTTGCTCCGTCAGGCTTTCGCCCATTGCGGAAGATTCCCTACTGCTGCCTCCCGTAGGAGTCTGGACCGTGTCTCAGTTCCAGTGTGGCCGATCACCCTCTCAGGGTCGGCTACGCATCGTTGCCTTGGTAAGCCGTTACCTTACCAACTAGCTAATGCGGCGCGGATCCATCTATAAGTGACAGCAAAACCGTCTTTCACTATTGAACCATGCGGTTCAATATATTATCCGGTATTAGCTCCGGT
>n.538.6.bb_436
GTAGTTAGCCGTGGCTTTCTGATTAGGTACCGTCAAGACGTGCATAGTTACTTACACATTTATTCTTCCCTAATAACAGAGTTTTACGATCCGAAGACCTTTATCACTCACGCGGCGTTGCTCCGTCAGGCTTTCGCCCATTGCGGAAGATTCCCTACTGCTGCCTCCCGTAGGAGTCTGGACCGTGTCTCAGTTCCAGTGTGGCCGATCACCCTCTCAGGGTCGGCTACGCATCGTTGCCTTGGTAAGCCGTTACCTTACCAACTAGCTAATGCGGCGCGGATCCATCTATAAGTGACAGCAAAACCGTCTTTCACTATTGAACCATGCGGTTCAATATATT
>n.538.6.bb_437
GTAGTTAGCCGTGGCTTTCTGATTAGGTACCGTCAAGACGTGCATAGTTACTTACACATTTGTTCTTCCCTAATAACAGAGTTTTACGATCCGAAGACCTTCATCACTCACGCGGCGTTGCTCCGTCAGGCTTTCGCCCATTGCGGAAGATTCCCTACTGCTGCCTCCCGTAGGAGTCTGGACCGTGTCTCAGTTCCAGTGTGGCCGATCACCCTCTCAGGGTCGGCTACGCATCGTTGCCTTGGTAAGCCGTTACCTTACCAACTAGCTAATGCGGCGCGGATCCATCTATAAGTGACAGCAAAACCGTCTTTCACTATTGAACCATGCGGTTCAATATATT
>n.538.6.bb_438
GTAGTTAGCCGGTGCTTCTTCTGCAGGTACCGTCACCACAAGCTTCGCCCCTGCTGAAAGCGGTTTACAACCCGAAGGCCGTCATCCCGCACGCGGCGTTGCTGCATCAGGCTTCCGCCCATTGTGCAATATTCCCCACTGCTGCCTCCCGTAGGAGTCTGGGCCGTATCTCAGTCCCAATGTGGCCGGTCGCCCTCTCAGGCCGGCTACCCGTCAAAGCCTTGGTAAGCCACTACCCCACCAACAAGCTGATAAGCCGCGAGTCCATCCAAAACCGCCGAAGCTTTTCCAACCCCCACCATGCAGCAAGGATTCCTATCCGGTATTAGCCCCAGTTTCCTGAAGTT
>n.538.6.bb_439
GTAGTTAGCCGTGGCTTTCTGATTAGGTACCGTCAAGACGTGCATAGTTACTTACACATTTGTTCTTCCCTAATAACAGAGTTTTACGATCCGAAGACCTTCATCACTCACGCGGCGTTGCTCCGCCAGGCTTTCGCCCATTGCGGAAGATTCCCTACTGCTGCCTCCCGTAGGAGTCTGGACCGTGTCTCAGTTCCAGTGTGGCCGATCACCCTCTCAGGGTCGGCTACGCATCGTTGCCTTGGTAAGCCGTTACCTTACCAACTAGCTAATGCGGCGCGGATCCATCTATAAGTGACAGCAAAACCGTCTTTCACTATTGAACCATGCGGTTCAATATATTATCCGGTATT
>n.538.6.bb_440
GTAGTTAGCCGGTGCTTCTTCTGCAGGTACCGTCACCACAAGCTTCGCCCCTGCTGAAAGCGGTTTACAACCCGAAGGCCGTCATCCCGCACGCGGCGTTGCTGCATCAGGCTTCCGCCCATTGTGCAATATTCCCCACTGCTGCCTCCCGTAGGAGTCTGGGCCGTATCTCAGTCCCAATGTGGCCGGTCGCCCTCTCAGGCCGGCTACCCGTCAAAGCCTTGGTAAGCCACTACCCCACCAACAAGCTGATAAGCCGCGAGTCCATCCAAACCGCCGAAGCTTTTCCAACCCCCACCATGCAGCAAGGATTCCTATCCGGTATT
>n.538.6.bb_441
GTAGTTAGCCGTGGCTTTCTGATTAGGTACCGTCAAGACGTGCATAGTTACTTACACATTTGTTCTTCCCTAATAACAGAGTTTTACGATCCGAAGACCTTCATCACTCACGCGGCGTTGCTCCGTCAGGCTTTCGCCCATTGCGGAAGATTCCCTACTGCTGCCTCCCGTAGGAGTCTGGACCGTGTCTCAGTTCCAGTGTGGCCGATCACCCTCTCAGGGTCGGCTACGCATCGTTGCCTTGGTAAGCCGTTACCTTACCAACTAGCTAATGCGGCGCGGATCCATCTATAAGTGACAGCAAAACCGTCTTTCACTATTGAACCATGCGGTTCAATATATTATCCGGTATTAGCTCCGGTTTCCCGAAGTTATCCCAGTCTTATAGGTAGGTTATCCCACCGTGTTACTCACCCCGTCCCGCC
>n.538.6.bb_442
GCAGTTAGCCGGTGCTTCTTCTGCAGGTACCGTCACCGACAAGCTTTCGCCCCTGCTGAAAGCGGTTTAACAACCCGAAGGCCGTTCATCCCGACACGCGGCGTTGCTGCATCAGGCTTCCGCCCTATCGTGCAATATTCCCCACTGCTGCCTCCCGTAGGAGTCTGGGCCGTATCTCAGTCCCAATGTGGCCGGTCGCCCT
>n.538.6.bb_443
GTAGTTAGCCGGTGCTTCTTCTGCAGGTACCGTCACCACAAGCTTCGCCCCTGCTGAAAGCGGTTTACAACCCGAAGGCCGTCATCCCGCACGCGGCGTTGCTGCATCAGGCTTCCGCCCATTGTGCAATATTCCCCACTGCTGCCTCCCGTAGGAGTCTGGGCCGTATCTCAGTCCCAATGTGGCCGGTCGCCCTCTCAGGCCGGCTACCCCGGTCAAAGCCTTGGTAAGCCACTACCCCACCAACAAGCTGATAAGCCGCGAGTCCATCCAAAACCGC
>n.538.6.bb_444
GAGTTAGCCGGTGCTTCTTCTGCGGGTAACGTCAATCGATGAGGTTATTAACCTCACCGCCTTCCTCCCCGCTGAAAGTGCTTTACAACCCGAAGGCCTTCTTCACACACGCGGCATGGCTGCATCAGGCTTGCGCCCATTGTGCAATATTCCCCACTGCTGCCTCCCGTAGGAGTCTGGACCGTGTCTCAGTTCCAGTGTGGCTGGGTCATCCTCTCAGACCAGCTAGGGATCGTCGCCTAGGTGAGCCGTTACCCACCTACCAGCTAATCCCATCTGGGCACATCTGATGGCATGGAGGCCCGAACGGTCCCCACTTTGGTCTTGCGACATTATGCGGTATTAGCTACCGTTTCCAG
>n.538.6.bb_445
GTAGTTAGCCGTGGCTTTCTGATTAGGTACCGTCAAGACGTGCATAGTTACTTACACATTTGTTCTTCCCTAATAACAGAGTTTTACGATCCGAAGACCTTCATCACTCACGCGGCGTTGCTCCGTCAGGCTTTCGCCCATTGCGGAAGATTCCCTACTGCTGCCTCCCGTAGGAGTCTGGACCGTGTCTCAGTTCCAGTGTGGCCGATCACCCTCTCAGGGTCGGCTACGCATCGTTGCCTTGGTAAGCCGTTACCTTACCAACTAGCTAATGCGACGCGGATCCATCTATAAGTGACAGC
>n.538.6.bb_446
GTAGTTAGCCGGTGCTTCTTCTGCAGGTACCGTCACCACAAGCTTCGCCCCTGCTGAAAGCGGTTTACAACCCGAAGGCCGTCATCCCGCACGCGGCGTTGCTGCATCAGGCTTCCGCCCATTGTGCAATATTCCCCACTGCTGCCTCCCGTAGGAGTCTGGGCCGTATCTCAGTCCCAATGTGGCCGGTCGCCCTCTCAGGCCGGCTACCCGTCAAAGCCTTGGTAAGCCACTACCCACCAACAAGCTGATAAGCCGCGAGTCCATCCAAAACCGC
>n.538.6.bb_447
TAGTTAGCCGTGGCTTTCTGATTAGGTACCGTCAAGACGTGCATAGTTACTTACACATTTGTTCTTCCCTAATAACAGAGTTTTACGATCCGAAGACCTTCATCACTCACGCGGCGTTGCTCCGTCAGGCTTTCGCCCCATTGCGGAAGATTCCCTACTGCTGCCTCCCGTAGGAGTCTGGACCGTGTCTCAGTTCCAGTGTGGCCCGATCACCCTNTCAGGGTCGGCTACGCATCG
>n.538.6.bb_448
GTAGTTAGCCGTGGCTTTCTGATTAGGTACCGTCAAGACGTGCATAGTTACTTACACATTTGTTCTTCCCTAATAACAGAGTTTTACGATCCGAAGACCTTCATCACTCACGCGGCGTTGCTCCGTCAGGCTTCCGCCCATTGCGGAAGATTCCCTACTGCTGCCTCCCGTAGGAGTCTGGACCGTGTCTCAGTTCCAGTGTGGCCGATCACCCTCTCAGGGTCGGCTACGCATCGTTGCCTTGGTAAGCCGTTACCTTACCAACTAGCTAATGCGGCGCGGATCTATCTATAAGTGACAGCAAAACCGTCTTTCACTATTGAA
>n.538.6.bb_449
GTAGTTAGCCGTGGCTTTCTGATTAGGTACCGTCAAGACGTGCATAGTTACTTACACATTTGTTCTTCCCTAATAACAGAGTTTTACGATCCGAAGACCTTCATCACTCACGCGGCGTTGCTCCGTCAGGCTTTCGCCCATTACGGAAGATTCCCTACTGCTGCCTCCCGTAGGAGTCTGGACCGTGTCTCAGTTCCAGTGTGGCCGATCACCCTCTCAGGGTCGGGCTACGCATCGTTGCCTTGGTAAGCCGTTACCTTACCAACTAGCTAATGCGGCGCGGATCCATCTATAAGTGACAGCAAAACCGTCTTTCACTATTGAACCATGCGGTTCAATATATTATCCGGTATTAGCTCCGGTTTCCCCGAAGTTATCCCAGTCTTATAGGTAGGTTAT
>n.538.6.bb_450
GTAGTTAGCCGGTGCTTCTTCTGCAGGTACCGTCACCACAAGCTTCGCCCCTGCTGAAAGCGGTTTACAACCCGAAGGCCGTCATCCCGCACGCGGCGTTGCTGCATCAGGCTTCCGCCCATTGTGCAATATTCCCCACTGCTGCCTCCCGTAGGAGTCTGGGCCGTATCTCAGTCCCAATGTGGCCGGTCGCCCTCTCAGGCCGGCTACCCGTCAAAGCCTTGGTAAGCCACTACCCCACCAACAAGCTGATAAGCCGCGAGTCCATCCAAAACCGCCGAAGCTTTCCAACCCCACCATGCAGCAAGGATTCTATCCGGTATTAGCCCAGTTTCCTGAAGTTATCCCGAAGTCAAGGGCAGGTTACTCACGTGTT
>n.538.6.bb_451
GTAGTTAGCCGGTGCTTCTTCTGCAGGTACCGTCACCACAAGCTTCGCCCCTGCTGAAAGCGGTTTACAACCCGAAGGCCGTCATCCCGCACGCGGCGTTGCTGCATCAGGCTTCCGCCCATTGTGCAATATTCCCCACTGCTGCCTCCCGTAGGAGTCTGGGCCGTATCTCAGTCCCAATGTGGCCGGTCGCCCTCTCAGGCCGGCTACCCGGTCAAAGCCTTGGTAAGCCACTACCCCACCAACAAGCTGATAAGCCGCGAGTCCATCCAAAACCGC
>n.538.6.bb_452
GTAGTTAGCCGTGGCTTTCTGATTAGGTACCGTCAAGACGTGCATAGTTACTTACACATTTGTTCTTCCCTAATAACAGAGTTTTACGATCCGAAGACCTTCATCACTCACGCGGCGTTGCTCCGTCAGGCTTTCGCCCATTGCGGAAGATTCCCTACTGCTGCCTCCCGTAGGAGTCTGGACCGTGTCTCAGTTCCAGTGTGGCCGATCACCCTCTCAGGGTCGGCTACGCATCGTTGCCTTGGTAAGCTGTTACCTTACCAACTAGCTAATGCGGCGCGGATCCATCTATAAGTGACAGCAAAACCGTCTTTCACTATTGAACCATGCGGTTCAATATATTATCCGGTATTAGCTCCGGTTTCCCGAAGTTATCCCAGTCTTATAGGTAGGT
>n.538.6.bb_453
GTAGTTAGCCGGTGCTTCTTCTGCAGGTACCGTCACCACAAGCTTCGCCCCTGCTGAAAGCGGTTTACAACCCGAAGGCCGTCATCCCGCACGCGGCGTTGCTGCATCAGGCTTCCGCCCATTGTGCAATATTCCCCACTGCTGCCTCCCGTAGGAGTCTGGGCCGTATCTCAGTCCCAATGTGGCCGGTCGCCCTCTCAGGCCGGCTACCCGTCAAAGCCTTGGTAAGCCACTACCCACCAACAAGCTGATAAGCCGCGAGTCCATCCAAAACCGCCGA
>n.538.6.bb_454
GTAGTTAGCCGGTGCTTCTTCTGCAGGTACCGTCACCACAAGCTTCGCCCCTGCTGAAAGCGGTTTACAACCCGAAGGCCGTCATCCCGCACGCGGCGTTGCTGCATCAGGCTTCCGCCCATTGTGCAATATTCCCCACTGCTGCCTCCCGTAGGAGTCTGGGCCGTATCTCAGTCCCAATGTGGCCGGTCGCCCTCTCAGGCCGGCTACCCGTCAAAGCCTTGGTAAGCCACTACCCACCAACAAGCTGATAAGCCGCGAGTCCATCCAAAACCGC
>n.538.6.bb_455
GTAGTTAGCCGTGGCTTTCTGATTAGGTACCGTCAAGACGTGCATAGTTACTTACACATTTGTTCTTCCCTAATAACAGAGTTTTACGATCCGAAGACCTTCATCACTCACGCGGCGTTGCTCCGTCAGGCTTTCGCCCATTGCGGAAGATTCCCTACTGCTGCCTCCCGTAGGAGTCTGGACCGTGTCTCAGTTCCAGTGTGGCCGATCACCTTCTCAGGGTCGGCTACGCATCGTTGCCTTGGT
>n.538.6.bb_456
GTAGTTAGCCGTGGCTTTCTGATTAGGTACCGTCAAGACGTGCATAGTTACTTACACATTTATTCTTCCCTAATAACAGAGTTTTACGATCCGAAGACCTTCATCACTCACGCGGCGTTGCTCCGTCAGGCTTTCGCCCATTGCGGAAGATTCCCTACTGCTGCCTCCCGTAGGAGTCTGGACCGTGTCTCAGTTCCAGTGTGGCCGATCACCCTCTCAGGGTCGGCTACGCATCGTTGCCTTGGTAAGCCGTTACCTTACCAACTAGCTAATGCGGCGCGGATCCATCTATAAGTGACAGCAAAAACCGTCTTTCACTATTGAACCATGCGGTTCAATATATATCCGGTATTAGCTCCGGT
>n.538.6.bb_457
GTAGTTAGCCGTGGCTTTCTGATTAGGTACCGTCAAGACGTGCATAGTTACTTACACATTTGTTCTTCCCTAATAACAGAGTTTTACGATCCGAAGACCTTCATCACTCACGCGGCGTTGCTCCGTCAGGCTTTCGCCCATTGCGGAAGATTCCCTACTGCTGCCTCCCGTAGGAGTCTGGACCGTGTCTCAGTTCCAGTGTGGCCGATCACCCTCTCAGGGTCGGCTACGCATCGTGCCTTGGTAAGCCGTTACCTTACCAACTAGCTAATGCGGCGCGGATCCATCTATAAGTGACAGCAAAACCGTCTTTCACTATTGAACCATGCGGTTCAATATATTATCCCGGTATTAGCTCCGGTTTC
>n.538.6.bb_458
GTAGTTAGCCGTGGCTTTCTGATTAGGTACCGTCAAGACGTGCATAGTTACTTACACATTTGTTCTTCCCTAATAACAGAGTTTTACGATCCGAAGACCTTCATCACTCACGCGGCGTTGCTCCGTCAGGCTTTCGCCCATTGCGGAAGATTCCCTACTGCTGCCTCCCGTAGGAGTCTGGACCGTGTCTCAGTTCCAGTGTGGCCGATCACCCTCTCAGGGTCAGCTACGCATCGTTGCCTTGGTAAGCCGTTACCTTACCAACTAGCTCATGCGGCGCGGATCCATCTATAAGTGACAGCAAAACCGTCTTTCACTATTGAACCATGCGGTTCAATATATTATCCGGTAT
>n.538.6.bb_459
GTAGTTAGCCGGTGCTTCTTCTGCAGGTACCGTCACCACAAGCTTCGCCCCTGCTGAAAGCGGTTCACAACCCGAAGGCCGTCATCCCGCACGCGGCGTTGCTGCATCAGGCTTCCGCCCATTGTGCAATATTCCCCACTGCTGCCTCCCGTAGGAGTCTGGGCCGTATCTCAGTCCCAATGTGGCCGGTCGCCCTCTCAGGCCGGGCTACCCGTCAAAGCCTTGGAAGCCACTACCCACCAACAAGCTGATAAGCCGCGAGTCCAT
>n.538.6.bb_460
GTAGTTAGCCGTGGCTTTCTGATTAGGTACCGTCAAGACGTGCATAGTTACTTGCACATTTGTTCTTCCCTAATAACAGAGTTTTACGATCCGAAGACCTTCATCACTCACGCGGCGTTGCTCCGTCAGGCTTTCGCCCATTGCGGAAGATTCCCTACTGCTGCCTCCCGTAGGAGTCTGGACCGTGTCTCAGTTCCAGTGTGGCCGATCACCCTCTCAGGTCGGCTACGCATCGTTGCCTTGGTAAGCCGTTACCTTACCAACTAGCTAATGCGGCGCGGATCCATCTATAAGTGACAGCAAAACCGTCTTTCACTATTGAACCATGCGGTTCAATATATTATCCGGTATTAGCTCCGGTTTCCCGAAGTTATCCCAGTCTTATAGGTAGGTTATCCCACGTGTTACC
>n.538.6.bb_461
GGAGTTAGCCGGTGCTTCTTCTGCGGGTAACGTCAATTGCTGAGGTTATTAACCTCAACACCTTCCTCCCCGCTGAAAGTACTTTACAACCCGAAGGCCTTCTTCATACACGCGGCATGGCTGCATCAGGCTTGCGCCCATTGTGCAATATTCCCCACTGCTGCCTCCCGTAGGAGTCTGGACCGTGTCTCAGTTCCAGTGTGGCTGGGTCATCCTCTCAGACCAGCTAGGGGATCGTCGCCTAGGTGAGCCGTTACCCCACCTACTAGCTAATCCCATCTGGGCACATCTGATGGCAAGAGGCCCGAAGGTCCCCCTCTTTGGTCTTGCGACGTTATGCGGTATTAGC
>n.538.6.bb_462
GTAGTTAGCCGGTGCTTCTTCTGCAGGTACCGTCACCACAAGCTTCGCCCCTGCTGAAAGCGGTTTACAACCCGAAGGCCGTCATCCCGCACGCGGCGTTGCTGCATCAGGCTTCCGCCCATTGTGCAATATTCCCCACTGCTGCCTCCCGTAGGAGTCTGGGCCGTATCTCAGTCCCAATGTGGCCGGTCGCCCTCTCAGGCCGGCTACCCGGTCAAAGCCTTGGTAAGCCACTACCCCACCAACAAAGCTGATAAGCCGCGAGTCCATCCAAAACCGCCGAAGCTTTTCCAACCCCCACCATGCAGCAAGGATTCCTATCCGGTATTAGCCCCAGTTTCCTGAAGTTATCCCGAAGTCAAG
>n.538.6.bb_463
GTAATTAGCCGTGGCTTTCTGATTAGGTACCGTCAAGACGTGCATAGCTACTTACACATTTGTTCTTCCCTAATAACAGAGTTTTACGATCCGAAGACCTTCATCACTCACGCGGCGTTGCTCCGTCAGGCTTTCGCCCATTGCGGAAGATTCCCTACTGCTGCCTCCCGTAGGAGTCTGGACCGTGTCTCAGTTCCAGTGTGGCCGATCACCCTNTCAGGGTCGCTACGCATCGTTGCCTTGGTAAGCCGTTACCTTACCAACTAGCTAATGCGGCGCGGGATCCATCTATAAGTGACAGCAAAACCGTCTTTCACTATTGAACCATGCGGTTCAATATATTATCCGGTAT
>n.538.6.bb_464
GGAGTTAGCCGGTGCTTCTTCTGCGGGTAACGTCAATCGACAGGGTTATTAACCCTGTCGCCTTCCTCCCCGCTGAAAGTACTTTACAACCCGAAGGCCTTCTTCATACACGCGGCATGGCTGCATCAGGCTTGCGCCCATTGTGCAATATTCCCCACTGCTGCCTCCCGTAGGAGTCTGGACCGTGTCTCAGTTCCAGTGTGGCTGGTCATCCTCTCAGACCAGCTAGGGATCGTCGCCTAGGTGAGCCGTTACCCACCTACTAGCTAATCCCATCTGGGCACATCT
>n.538.6.bb_465
GTAGTTAGCCGTGGCTTTCTGATTAGGTACCGTCAAGACGTGCATAGTTACTTACACATTTGTTCTTCCCTAATAACAGAGTTTTACGATCCGAAGACCTTCATCACTCACGCGGCGTTGCTCCGTCAGGCTTTCGCCCATTGCGGAAGATTCCCTACTGCTGCCTCCCGTAGGAGTCTGGACCGTGTCTCAGTTCCAGTGTGGCCGATCACCCTCTCAGGGTCGGCTACGCATCGTTGCCTTGGTAAGCCGTTACCTTACCAACTAGCTAATGCGGCGCGGATCCATCTATAAGTGACAGCAAAACCGTCTTTCACTATTGAACCATGCGGTTCAATATATTATCCGGTATT
>n.538.6.bb_466
GTAGTTAGCCGGTGCTTCTTCTGCAGGTACCGTCACCACAAGCTTCGCCCCTGCTGAAAGCGGTTTACAACCCGAAGGCCGTCATCCCGCACGCGGCGTTGCTGCATCAGGCTTCCGCCCATTGTGCAATATTCCCCACTGCTGCCTCCCGTAGGAGTCTGGGCCGTATCTCAGTCCCAATGTGGCCGGTCGCCCTCTCAGGCCGGCTACCCGTCAAAGCCTTGGTAAGCCACTACCCCACCAACAAGCTGATAAGCCGCGAGTCCATCCAAAACCGCCGAAGCTTTCCCAACCCCCACCATGCAGCAAGGATTCCTATCCGGTATTAGCCCCAGTTT
>n.538.6.bb_467
GTAGTTAGCCGTGGCTTTCTGATTAGGTACCGTCAAGACGTGCATAGTTACTTACACATTTGTTCTTCCCTAATAACAGAGTTTTACGATCCGAAGACCTTCATCACTCACGCGGCGTTGCTCCGTCAGGCTTTCGCCCATTGCGGAAGATTCCCTACTGCTGCCTCCCGTAGGAGTCTGGACCGTGTCTCAGTTCCAGTGTGGCCGATCACCCTCTCAGGGTCGGCTACGCATCGTTGCCTTGGTAAGCCGTTACCTTACCAACTAGCTAATGCGGCGCGGATCCATCTATAAGTGAC
>n.538.6.bb_468
GTAGTTAGCCGGTGCTTCTTCTGCAGGTACCGTCACCACAAGCTTCGCCCCTGCTGAAAGCGGTTTACAACCCGAAGGCCGTCATCCCGCACGCGGCGTTGCTGCATCAGGCTTCCGCCCATTGTGCAATATTCCCCACTGCTGCCTCCCGTAGGAGTCTGGGCCGTATCTCAGTCCCAATGTGGCCGGTCGCCCTCTCAGGCCGGCTACCCGGTCAAAGCCTTGGTAAGCCACTACCCCACCAACAAGCTGATAAGCCGCGAGTCCATCCAAAGCCG
>n.538.6.bb_469
GTAGTTAGCCGGTGCTTCTTCTGCAGGTACCGTCACCACAAGCTCGCCCCTGCTGAAAGCGGTTTACAACCCGAAGGCCGTCATCCCGCACGCGGCGTTGCTGCATCAGGCTTCCGCCCATTGTGCAATATTCCCCACTGCTGCCTCCCGTAGGAGTCTGGGCCGTATCTCAGTCCCAATGTGGCCGGTCGCCCTCTCAGGCCGGCTACCCGTCAAAGCCTTGGTAAGCCACTACCCCACCAACAAGCTGATAAGCCGCGAGTCCATCCAAAACCGCCGAAGCTTTCCAACCCCACCATGCAGCAAGGATTCCTATCCGGTATTAGCCCCAGTTT
>n.538.6.bb_470
GTAGTTAGCCGTGGCTTTCTGATTAGGTACCGTCAAGACGTGCATAGTTACTTACACATTTGTTCTTCCCTAATAACAGAGTTTTACGATCCGAAGACCTTCATCACTCACGCGGCGTTGCTCCGTCAGGCTTTCGCCCATTGCGGAAGATTCCCTACTGCTGCCTCCCGTAGGAGTCTGGACCGTGTCTCAGTTCCAGTGTGGCCGATCACCCTCTCAGGGTCGGCTACGCATCGTTGCCTTGGTAAGCCGTTACCTTACCAACTAGCTAATGCGGCGCGGATCCATCTATAAGTGACAGCAAAAACCGTCTTTCACTATTGAACCATGCGGTTCAATATATTATCCGGTATTAGCTCCGGTTT
>n.538.6.bb_471
GTAGTTAGCCGGTGCTTCTTCTGCAGGTACCGTCACCACAAGCTTCGCCCCTGCTGAAAGCGGTTTACAACCCGAAGGCCGTCATCCCGCACGCGGCGTTGCTGCATCAGGCTTCCGCCCATTGTGCAATATTCCCCACTGCTGCCTCCCGTAGGAATCTGGGCCGTATCTCAGTCCCAATGTGGCCGGTCGCCCTCTCAGGCCGGCTACCCGTCAAAGCCTTGGTAAGCCACTACCCCACCAACAAGCTGATAAGCCGCGAGTCCATCCAACACCGCCGAAGCTTTCCAACCCCCACCATGCAGCAAGGATTCCTATCCGGTAT
>n.538.6.bb_472
GTAGTTAGCCGTGGCTTTCTGATTAGGTACCGTCAAGACGTGCATAGTTACTTACACATTTGTTCTTCCCTAATAACAGAGTTTTACGATCCGAAGACCTTCATCACTCACGCGGCGTTGCTCCGTCAGGCTTTCGCCCATTGCGGAAGATTCCCTACTGCTGCCTCCCGTAGGAGTCTGGACCGTGTCTCAGTTCCAGTGTGGCCGATCACCCTCTCAGGGTCGGCTACGCATCGTTGCCTTGGTAAGCCGTTACTTACAACTAGCTAATGCGGCGCGGATCCATCTATAAGTGACAGCAAAACGTCTTTCACTATTGAACCATG
>n.538.6.bb_473
GTAGTTAGCCGTGGCTTTCTGATTAGGTACCGTCAAGACGTGCATAGTTACTTACACATTTGTTCTTCCCTAATAACAGAGTTTTACGATCCGAAGACCTTCATCACTCACGCGGCGTTGCTCCGTCAGGCTTTCGCCCATTGCGGAAGATTCCCTACTGCTGCCTCCCGTAGGAGTCTGGACCGTGTCTCAGTTCCAGTGTGGCCGATCACCCTCTCAGGGTCGGCTACGCATCGTTGCCTTGGTAAGCCGTTACCTTACCAACTAGCTAATGCGGCGCGGATCCATCTATAAGTGACAGCAAAACCGTCTTTCACTATTGAACCATGCGGTTCAATATATTATCCGGTATTAGCTCCGGTTTCCCGAAGTTATCCCAGTCTTATAGGTAGGTTATCCCACGTGTTACTCACCCCGTCCCGCCCGCTAACGTCAGAGGAGCAAGCTCCCTCGTCCTGTTCGCTCGACTTGCATGTATTAGGGCACGCCGCCAGCGTT
>n.538.6.bb_474
GTAGTTAGCCGGTGCTTCTTCTGCAGGTACCGTCACCACAAGCTTCGCCCCTGCTGAAAGCGGTTTACAACCCGAAGGCCGTCATCCCGCACGCGGCGTTGCTGCATCAGGCTTCCGCCCATTGTGCAATATTCCCCACTGCTGCCTCCCGTAGGAGTCTGGGCCGTATCTCAGTCCCAATGTGGCCGGTCGCCCTCTCAGGCCGGCTACCCGGTCAAAGCCTTGGTAAGCCACTACCCCACCAACAAGCTGATAAGCCGCGAGTCCATCCAAAACCGCCGAAGCTTTCCAACCCCACCATGCAGCAAGGATTCCTATCCGGTATTAGCCCCAGTTT
>n.538.6.bb_475
GTAGTTAGCCGTGGCTTTCTGATTAGGTACCGTCAAGACGTGCATAGCTACTTACACATTTGTTCTTCCCTAATAACAGAGTTTTACGATCCGAAGACCTTCATCACTCACGCGGCGTTGCTCCGTCAGGCTTTCGCCCATTGCGGAAGATTCCCTACTGCTGCCTCCCGTAGGAGTCTGGACCGTGTCTCAGTTCCAGTGTGGCCGATCACCCTCTCAGGGTCGGCTACGCATCGTTGCCTTGGTAAGCCGTTACCTTACCAACTAGCTAATGCGGCGCGGATCCATCTATAAGTGACAGCAAAACCGTCTTTCACTATTGAACCATGCGGTTCAATATATTATCCGGTATTAGCTCCGGTTTCCCGAAGTTATCCCAGTCTTATAGGTAGGTTATCCCACGTGTTAC
>n.538.6.bb_476
GTAGTTAGCCGGTGCTTCTTCTGCAGGTACCGTCACCACAAGCTTCGCCCCTGCTGAAAGCGGTTTACAACCCGAAGGCCGTCATCCCGCACGCGGCGTTGCTGCATCAGGCTTCCGCCCATTGTGCAATATTCCCCACTGCTGCCTCCCGTAGGAGTCTGGGCCGTATCTCAGTCCCAATGTGGCCGGTCGCCCTCTCAGGCCGGCTACCCGGTCAAAGCCTTGGTAAGCCGCTACCCCACCAACAAGCTGATAAGCCGCGAGTCCATCCA
>n.538.6.bb_477
GTAGTTAGCCGTGGCTTTCTGATTAGGTACCGTCAAGACGTGCATAGTTACTTACACATTTGTTCTTCCCTAATAACAGAGTTTTACGATCCGAAGACCTTCATCACTCACGCGGCGTTGCTCCGTCAGGCTTTCGCCCATTGCGGAAGATTCCCTACTGCTGCCTCCCGTAGGAGTCTGGACCGTGTCTCAGTTCCAGTGTGGCCGATCACCCTCTCAGGGTCGGCTACGCATCGTTGCCTTGGTAAGCCGTTACCTTACCAACTAGCTAATGCGGCGCGGATCCATCTATAAGTGACAGCAAAACCGTCTTTCACTATTGAACCATGCGGTTCAATATAT
>n.538.6.bb_478
GTAGTTAGCCGGTGCTTCTTCTGCAGGTACCGTCACCACAAGCTTCGCCCCTGCTGAAAGCGGTTTACAACCCGAAGGCCGTCATCCCGCACGCGGCGTCGCTGCATCAGGCTTCCGCCCATTGTGCAATATTCCCCACTGCTGCCTCCCGTAGGAGTCTGGGCCGTATCTCAGTCCCAATGTGGCCGGTCGCCCTCTCAGGCCGGCTACCCGTCAAAGCCTTGGTAAGCCACTACCCCACCAACAAGCTGATAAGCCGCGAGTCCATCCAAAACCGCCG
>n.538.6.bb_479
GTAGTTAGCCGTGGCTTTCTGATTAGGTACCGTCAAGACGTGCATAGTTACTTACACATTTGTTCTTCCCTAATAACAGAGTTTTACGATCCGAAGACCTTCATCACTCACGCGGCGTTGCTCCGTCAGGCTTTCGCCCATTGCGGAAGATTCCCTACTGCTGCCTCCCGTAGGAGTCTGGACCGTGTCTCAGTTCCAGTGTGGCCGATCACCCTCTCAGGGTCGGCTACGCATCGTTGCCTTGGTAAGCCGTTACCTTACCAACTAGCTAATGCGGCGCGGATCCATCTATAAGTGACAGCAAAAACCGTCTTTCACTATTGAACCATGCGGTTCAATATATTATCCGGTATTAGCTCCGGTTTCCCGAAGTTATCCCAGTCTTATAGGTAGGTTATCCCACGTGTTACCTCACCCCGTCCCGCCCGCTAACGTCAGAGGAGCAAGCTCCCTCGTCTGTTCCGCTCGACTTGCATGTATTAGGGCACGCCGCCAGCGTTCA
>n.538.6.bb_480
GTAGTTAGCCGGTGCTTCTTCTGCAGGTACCGTCACCACAAGCTTCGCCCCTGCTGAAAGCGGTTTACAACCCGAAGGCCGTCATCCCGCACGCGGCGTTGCTGCATCAGGCTTCCGCCCATTGTGCAATATTCCCCACTGCTGCCTCCCGTAGGAGTCTGGGCCGTATCTCAGTCCCAATGTGGCCGGTCGCCCTCTCAGGCCGGCTACCCGGTCAAAGCCTTGGTAAGCCACTACCCCACCAACAAGCTGATAAGCCGCGAGTCCAT
>n.538.6.bb_481
GTAGTTAGCCGTGGCTTTCTGATTAGGTACCGTCAAGACGTGCATAGTTACTTACACATTTGTTCTTCCCTAATAACAGAGTTTTACGATCCGAAGACCTTCATCACTCACGCGGCGTTGCTCCGTCAGGCTTTCGCCCATTGCGGAAGATTCCCTACTGCTGCCTCCCGTAGGAGTCTGGACCGTGTCTCAGTTCCAGTGTGGCCGATCACCCTCTCAGGGTCGGCTACGCATCGTTGCCTTGGTAAGCCGTTACCTTACCAACTAGCTAATGCGGCGCGGATCCATCTATAAGTGACAGCAAAACCGTCTTTCACTATTGAACCATGCGGTTCAATATATTATCCGGTATTA
>n.538.6.bb_482
GTAGTTAGCCGTGGCTTTCTGATTAGGTACCGTCAAGACGTGCATAGTTACTTACACATTTGTTCTTCCCTAATAACAGAGTTTTACGATCCGAAGACCTTCATCACTCACGCGGCGTTGCTCCGTCAGGCTTTCGCCCATTGCGGAAGATTCCCTACTGCTGCCTCCCGTAGGAGTCTGGACTGTGTCTCAGTTCCAGTGTGGCCGATCACCCTCTCAGGTCGGCTACGCATCGTTGCCTTGGTAAGCCGTTACTTACCAACTAGCTAATGCGGCGCGGATCCATCTATAAGTGACAGCAAAACCGTCTTTCACTATTGAACCATGCGGTTCAATATATTATCCGGTATTAGCTCCGGT
>n.538.6.bb_483
GTAGTTAGCCGTGGCTTTCTGATTAGGTACCGTCAAGACGTGCATAGTTACTTACACATTTATTCTTCCCTAATAACAGAGTTTTACGATCCGAAGACCTTCATCACTCACGCGGCGTTGCTCCGTCAGGCTTTCGCCCATTGCGGAAGATTCCCTACTGCTGCCTCCCGTAGGAGTCTGGACCGTGTCTCAGTTCCAGTGTGGCCGATCACCCTCTCAGGGTCGGCTACGCATCGTTGCCTTGGTAAGCCGTTACCTTACCAACTAGCTAATGCGGCGCGGATCCATCTATAAGTGACAGCAAAACCGTCTTTCACTATTGAACCATGCGGTTCAATATATTATCCGGTATTAGCTCCGGTTTCCCGAA
>n.538.6.bb_484
GTAGTTAGCCGGTGCTTCTTCTGCAGGTACCGTCACCACAAGCTTCGCCCCTGCTGAAAGCGGTTTACAACCCGAAGGCCGTCATCCCGCACGCGGCGTTGCTGCATCAGGCTTCCGCCCATTGTGCAATATTCCCCACTGCTGCCTCCCGTAGGAGTCTGGGCCGTATCTCAGTCCCAATGTGGCCGGTCGCCCTCTCAGGCCGGCTACCCGGTNAAAGCCTTGGTAAGCCACTACCCCACCAACAAGCTGATAAGCCGCGAGTCCATCCA
>n.538.6.bb_485
GTAGTTAGCCGTGGCTTTCTCGAGTTAGGTACCGTACAAGACGTGCAGTACGGTTACTTACGACAGTTTGTTCTTCCCTAATAACAGAGTTTTACGATCCGAAGACCTTCATCACTCACGCGGCGTTGCTCCGTCAGGCTTTCGCCCATTGCGGAAGATTCCCTACTGCTGCCTCCCGTAGGAGTCTGGACCGTGTCTCAGTTCCAGTGTGG
>n.538.6.bb_486
GTAGTTAGCCGGTGCTTCTTCTGCAGGTACCGTCACCACAAGCTTCGCCCCTGCTGAAAGCGGTTTACAACCCGAAGGCCGTCATCCCGCACGCGGCGTTGCTGCATCAGGCTTCCGCCCATTGTGCAATATTCCCCACTGCTGCCTCCCGTAGGAGTCTGGGCCGTATCTCAGTCCCAATGTGGCCGGTCGCCCTCTCAGGCCGGCTACCCGTCAAAGCCTTGGTAAGCCACTACCCCACCAACAAGCTGATAAGCCGCGAGTCCATCCAAAACCGCC
>n.538.6.bb_487
GGAGTTAGCCGGTGCTTCTTCTGCGGGTAACGTCAATCGATGAGGTTATTAACCTCACCGCCTTCCTCCCCGCTGAAAGTGCTTTACAACCCGAAGGCCTTCTTCACACACGCGGCATGGCTGCATCAGGCTTGCGCCCATTGTGCAATATTCCCCACTGCTGCCTCCCGTAGGAGTCTGGACCGTGTCTCAGTTCCAGTGTGGCT
>n.538.6.bb_488
GGAGTTAGCCGGTGCTTCTTCTGCGGGTAACGTCAATTGCTGAGGTTGTTAACCTCAACACCTTCCTCCCCGCTGAAAGTACTTTACAACCCGAAGGCCTTCTTCATACACGCGGCATGGCTGCATCAGGCTTGCGCCCATTGTGCAATATTCCCCACTGCTGCCTCCCGTAGGAGTCTGGACCGTGTCTCAGTTCCAGTGTGGCTGGGTCATCCTCTCTGACCAGCTAGGGATCGTCGCCTAGGTGAGCCGTTACCCCACCTACTAGCTAATCCCATCTGGGCACATCTGATGGCAA
>n.538.6.bb_489
GTAGTTAGCCGTGGCTTTCTGATTAGGTACCGTCAAGACGTGCATAGTTACTTACACATTTGTTCTTCCCTAATAACAGAGTTTTACGATCCGAAGACCTTCATCACTCACGCGGCGTTGCTCCGTCAGGCTTTCGCCCATTGCGGAAGATTCCCTACTGCTGCCTCCCGTAGGAGTCTGGACCGTGTCTCAGTTCCAGTGTGGCCGATCACCCTCTCAGGGTCGGCTACGCATCGTTGCCTTGGTAAGCCGTTACCTTACCAACTAGCTAATGCGGCGCGGATCCATCTATAAGTGACAGCAAAACCGTCTTTCACTATTGAACCATGCGGTTCAATATATTATCCGGTATTAGCTCCGGTTTCCCGAAGTTATCCCAGTCTTATAGGTAGGTTATCCCACGTGTTACTCA
>n.538.6.bb_490
GTAGTTAGCCGTGGCTTTCTGATTAGGTACCGTCAAGACGTGCATAGTTACTTACACATTTGTTCTTCCCTAATAACAGAGTTTTACGATCCGAAGACCTTCATCACTCACGCGGCGTTGCTCCGTCAGGCTTTCGCCCATTGCGGAAGATTCCCTACTGCTGCCTCCCGTAGGAGTCTGGACCGTGTCTCAGTTCCAGTGTGGCCGATCACCCTCTCAGGGTCGGCTACGCATCGTTGCCTTGGTAAGCCGTTACCTTACCAACTAGCTAATGCGGCGCGGATCCATCTATAAGTGACAGCAAAACCGTCTTTCACTATTGAACCATGCGG
>n.538.6.bb_491
GGAGTTAGCCGGTGCTTCTTCTGCGGGTAACGTCAATCGACAAGGTTATTAACCTTATCGCCTTCCTCCCCGCTGAAAGTACTTTACAACCCGAAGGCCTTCTTCATACACGCGGCATGGCTGCATCAGGCTTGCGCCCATTGTGCAATATTCCCCACTGCTGCCTCCCGTAGGAGTCTGGACCGTGTCTCAGTTCCAGTGTGGCTGGTCATCCTCTCAGACCAGCTAGGGATCGTCGCCTAGGTGAGCCGTTACCCACCTACTAGCTAATCCCATCTGGGCACATC
>n.538.6.bb_492
GGAGTTAGCCGGTGCTTCTTCTGCGGGTAACGTCAATCGACAAGGTTATTAACCTTATCGCCTTCCTCCCCGCTGAAAGTACTTTACAACCCGAAGGCCTTCTTCATACACGCGGCATGGCTGCATCAGGCTTGCGCCCATTGTGCAATATTCCCCACTGCTGCCTCCCGTAGGAGTCTGGACCGTGTCTCAGTTCCAGTGTGGCTGGTCATCCTCTCAGACCAGCTAGGGATCGTCGCCTAGGTGAGCCGTTACCCACCTACTAGCTAATCCCATCTGGGCACATCTGATG
>n.538.6.bb_493
GTAGTTAGCCGTGGCTTTCTGATTAGGTACCGTCAAGACGTGCATAGTTACTTACACATTTGTTCTTCCCTAATAACAGAGTTTTACGATCCGAAGACCTTCATCACTCACGCGGCGTTGCTCCGTCAGGCTTTCGCCCATTGCGGAAGATTCCCTACTGCTGCCTCCCGTAGGAGTCTGGACCGTGTCTCAGTTCCAGTGTGGCCGATCACCCTCTCAGGGTCGGCTACGCATCGTTGCCTTGGTAAGCCGTTACCTTACCAACTAGCTAATGCGGCGCGGATCCATCTATAAGTGACAGCAAAACCGTCTTTCACTATTGAACCATGCGGTTCAATATATTATCCGGTATTAGCTCCGGTTTCCCGAAGTTATCCCAGTCTTATAGGTAGGTTATCC
>n.538.6.bb_494
GTAGTTAGCCGTGGCTTTCTGATTAGGTACCGTCAAGACGTGCATAGTTACTTACACATTTGTTCTTCCCTAATAACAGAGTTTTACGATCCGAAGACCTTCATCACTCACGCGGCGTTGCTCCGTCAGGCTTTCGCCCATTGCGGAAGATTCCCTACTGCTGCCTCCCGTAGGAGTCTGGACCGTGTCTCAGTTCCAGTGTGGCCGATCACCCTCTCAGGGTCGGCTACGCATCGTTGCCTGTAAGCCGTTACCTTACACTAGCTAATGCGGCGCGGATCCATCTATAAGTGACAGCAAAACCGTCTTTCACTATTGAACCATGCGGTTCAATATATTATCCGGTATT
>n.538.6.bb_495
GTAGTTAGCCGTGGCTTTCTGATTAGGTACCGTCAAGACGTGCATAGTTACTTACACATTTGTTCTTCCCTAATAACAGAGTTTTACGATCCGAAGACCTTCATCACTCACGCGGCGTTGCTCCGTCAGGCTTTCGCCCATTGCGGAAGATTCCCTACTGCTGCCTCCCGTAGGAGTCTGGACCGTGTCTCAGTTCCAGTGTGGCCGATCACCCTCTCAGGGTCGGCTACGCATCG
>n.538.6.bb_496
GTAGTTAGCCGTGGCTTTCTGATTAGGTACCGTCAAGACGTGCATAGTTACTTACACATTTGTTCTTCCCTAATAACAGAGTTTTACGATCCGAAGACCTTCATCACTCACGCGGCGTTGCTCCGTCAGGCTTTCGCCCATTGCGGAAGATTCCCTACTGCTGCCTCCCGTAGGAGTCTGGACCGTGTCTCAGTTCCAGTGTGGCCGATCACCCTCTCAGGGTCGGCTACGCATCGTTGCCTTGGTAAGCCGTTACCTTACCAACTAGCTAATGCGGCGCGGATCCATCTATAAGTGACAGCAAAACCGTCTTTCACTATTGAACCATGCGG
>n.538.6.bb_497
GGAGTTAGCCGGTGCTTCTTCTGCGGGTAACGTCAATTGCTGAGGTTATTAACCTCAGCACCTTCCTCCCCGCTGAAAGTACTTTACAACCCGAAGGCCTTCTTCATACACGCGGCATGGCTGCATCAGGCTTGCGCCCATTGTGCAATATTCCCCACTGCTGCCTCCCGTAGGAGTCTGGACCGTGTCTCAGTTCCAGTGTGGCTGGGTCATCCTCTCAGACCAGCTAGGGATCGTCGCCTAGGTGAGCCGTTACCCACCTACTAG
>n.538.6.bb_498
GGAGTTAGCCGGTGCTTCTTCTGCGGGTAACGTCAATTGCTGAGGTTATTAACCTCAACACCTTCCTCCCCGCTGAAAGTACTTTACTAACCCGAAGGCCTTCTTCATACACGCGGCATGGCTGCATCAGGCTTGCGCCCATTGTGCAATATTCCCCACTGCTGCCTCCGTAGGAGTCTGGACCGTGTCTCAGTTCCAGTGTGGCTGGGTCATCCTCTCAGACCAGCTAGGGATCGTCG
>n.538.6.bb_499
GTAGTTAGCCGGTGCTTCTTCTGCAGGTACCGTCACCACAAGCTTCGCCCCTGCTGAAAGCGGTTTACAACCCGAAGGCCGTCATCCCGCACGCGGCGTTGCTGCATCAGGCTTCCGCCCATTGTGCAATATTCCCCACTGCTGCCTCCCGTAGGAGTCTGGGCCGTATCTCAGTCCCAATGTGCCGTCGCCCTCTCAGGCCGGCTACCCCGTCAAAGCCTTGGTAAGCCACTACCCCACCAACAAGCTGATAAGCCGCGAGTCC
>n.538.6.bb_500
GTAGTTAGCCGTGGCTTTCTGATTAGGTACCGTCAAGACGTGCATAGTTACTTACACATTTGTTCTTCCCTAATAACAGAGTTTTACGATCCGAAGACCTTCATCACTCACGCGGCGTTGCTCCGTCAGGCTTTCGCCCATTGCGGAAGATTCCCTACTGCTGCCTCCCGTAGGAGTCTGGACCGTGTCTCAGTTCCAGTGTGGCCGATCACCCTNTCAGGGTCGGCTACGCATCGTTGCCT
>n.538.6.bb_501
GGAGTTAGCCGGTGCTTCTTCTGCGGGTAACGTCAATTGCTGAGGTTATTAACCTCAACACCTTCCTCCCCGCTGAAAGTACTTTACAACCCGAAGGCCTTCTTCATACACGCGGCATGGCTGCATCAGGCTTGCGCCCATTGTGCAATATTCCCCACTGCTGCCTCCCGTAGGAGTCTGGACCGTGTCTCAGTTCCAGTGTGGCTGGGTCATCCTCTCAGACCAGCTAGGGATCGTCGCCTAGGTGAGCCGTTACCCCACCTACTAGCTAATCCCATCTGGGCACATCTGATGGCAA
>n.538.6.bb_502
GAAGTTAGCCGGTGCTTCTTCTGCAGGTACCGTCACCACAAGCTTCGCCCCTGCTGAAAGCGGTTTACAACCCGAAGGCCGTCATCCCGCACGCGGCGTTGCTGCATCAGGCTTCCGCCCATTGTGCAATATTCCCCACTGCTGCCTCCCGTAGGAGTCTGGGCCTATCTCAGTCCCAATGTGGCCGGTCGCCCTCTCAGGCCGGCTACCCGTNAAAGCCTTGGTAAGCCACTACCCACCAACAAGCTGATAAGCCGCGAGTCCATCCAAAACCGCCG
>n.538.6.bb_503
GTAGTTAGCCGGTGCTTCTTCTGCAGGTACCGTCACCACAAGCTTCGCCCCTGCTGAAAGCGGTTTACAACCCGAAGGCCGTCATCCCGCACGCGGCGTTGCTGCATCAGGCTTCCGCCCATTGTGCAATATTCCCCACTGCTGCCTCCCGTAGGAGTCTGGGCCGTATCTCAGTCCCAATGTGGCCGGTCGCCCTCTCAGGCCGGCTACCCGGTCAAAGCCTTGGTAAGCCACTACCCACCAACAAGCTGATAAGCCGCGAGTCCATCCAAAACCGCCGAAAGCTTTCCAACCCCACCATGCAGCAAGGATTCCTATCCGGTATTAGCCCCAGTTTCCTGAAGTTATCCCGAAGTCAAG
>n.538.6.bb_504
GTAGTTAGCCGTGGCTTTCTGATTAGGTACCGTCAAGACGTGCATAGTTACTTACACATTTGTTCTTCCCTAATAACAGAGTTTTACGATCCGAAGACCTTCATCACTCACGCGGCGTTGCTCCGTCAGGCTTTCGCCCATTGCGGAAGATTCCCTACTGCTGCCTCCCGTAGGAGTCTGGACCGTGTCTCAGTTCCAGTGTGGCCGATCACCCTCTCAGGGTCGGCTACGCATCGTTGCCTTGGTAAGCCGTTACCTTACCAACTAGCTAATGCGGCGCGGATCCATCTATAAGTGACAGCAAAACCGTCTTTCACTATTGAACCATGCGGTTCAATATATTATCCGGTATTAGCTCCGGTTTCCCGAAGTTATCCCAGTCTTATAGGTAGGTTATC
>n.538.6.bb_505
GTAGTTAGCCGTGGCTTTCTGATTAGGTACCGTCAAGACGTGCATAGTTACTTACACATTTGTTCTTCCCTAATAACAGAGTTTTACGATCCGAAGACCTTCATCACTCACGCGGCGTTGCTCCGTCAGGCTTTCGCCCATTGCGGAAGATTCCCTACTGCTGCCTCCCGTAGGAGTCTGGACCGTGTCTCAGTTCCTGTGTGGCCGATCACCCTCTCAGGGTCGGCTACGCATCGTTGCCTTGTAAGCCGTTACCTTACAACTAGCTAATGCGGCGCGGATCCATCTATAAGTGACAGCAAAACCGTCTTTCACTATTGAACCATGCGGTTC
>n.538.6.bb_506
GTAGTTAGCCGTGGCTTTCTGATTAGGTACCGTCAAGACGTGCATAGTTACTTACACATTTGTTCTTCCCTAATAACAGAGTTTTACGATCCGAAGACCTTCATCACTCACGCGGCGTTGCTCCGTCAGGCTTTCGCCCATTGCGGAAGATTCCCTACTGCTGCCTCCCGTAGGAGTCTGGACCGTGTCTCAGTTCCAGTGTGGCCGATCACCCTCTCAGGGTCGGCTACGCATCGTCGCCTTGGTAAGCCGTTACCTTACCAACTAGCTAATGCGGCGCGGATCCATCTATAAGTGACAGCAAAACCGTCTTTCACTATTGAACCATGCGGTTCAATATATT
>n.538.6.bb_507
GGAGTTAGCCGGTGCTTCTTCTGCGGGTAACGTCAATCGACAAGGTTATTAACCTTATCGCCTTCCTCCCCGCTGAAAGTACTTTACAACCCGAAGGCCTTCTTCATACACGCGGCATGGCTGCATCAGGCTTGCGCCCATTGTGCAATACTTCCCCACTGCTGCCCTCCGGTAGGAGTCTGGACCGTGTCTCAGTTCCAGTGTGGCTGGTCA
>n.538.6.bb_508
GTAGTTAGCCGTGGCTTTCTGATTAGGTACCGTCAAGACGTGCATAGTTACTTACACATTTGTTCTTCCCTAATAACAGAGTTTTACGATCCGAAGACCTTCATCACTCACGCGGCGTTGCTCCGTCAGGCTTTCGCCCATTGCGGAAGATTCCCTACTGCTGCCTCCCGTAGGAGTCTGGACCGTGTCTCAGTTCCAGTGTGGCCGATCACCCTCTCAGGGTCGGCTACGCATCGTTGCCTTGGTAAGCCGTTACCTTACCAACTAGCTAATGCGGCGCGGATCCATCTATAAGTGACAGCAAAACCGTCTTTCACTATTGAACCATGCGGTTCAATATATTATCCGGTATTAGCTCCCGGT
>n.538.6.bb_509
GTAGTTAGCCGGTGCTTCTTCTGCAGGTACCGTCACCACAAGCTTCGCCCCTGCTGAAAGCGGTTTACAACCCGAAGGCCGTCATCCCGCACGCGGCGTTGCTGCATCAGGCTTCCGCCCATTGTGCAATATTCCCCACTGCTGCCTCCCGTAGGAGTCTGGGCCGTATCTCAGTCCCAATGTGGCCGGTCGCCCTCTCAGGCCGGCTACCCGGTCAAAGCCTTGGTAAGCCACTACCCCACCAACAAGCTGATAAGCCGCGAGTCCATCCAAAACCGCCGAAGCTTTCCAACCCCCACCATGCAGCAAGGATTCCTATCCGGTATTAGCCCCAGTTTCC
>n.538.6.bb_510
GTAGTTAGCCGGTGCTTCTTCTGCAGGTACCGTCACCACAAGCTTCGCCCCTGCTGAAAGCGGTTTACAACCCGAAGGCCGTCATCCCGCACGCGGCGTTGCTGCATCAGGCTTCCGCCCATTGTGCAATATTCCCCACTGCTGCCTCCCGTAGGAGTCTGGGCCGTATCTCAGTCCCAATGTGGCCGGTCGCCCTCTCAGGCCGGCTACCCGGTCAAAGCCTTGGTAAGCCACTACCCCACCAACAAGCTGATAAGCCGCGAGTCCATCCAAAACCGC
>n.538.6.bb_511
GTAGTTAGCCGGTGCTTCTTCTGCAGGTACCGTCACCACAAGCTTCGCCCCTGCTGAAAGCGGTTTACAACCCGAAGGCCGTCATCCCGCACGCGGCGTTGCTGCATCAGGCTTCCGCCCATTGCGCAATATTCCCCACTGCGGCCTCCCGTAGGAGTCTGGGCCGTATCTCAGCCCAATGTGGCCGTCGCCCTCTCAGGCCGGCTACCCGGTCAAAAGGCCTTGGGTAAGCCACTACCCCACAACAAGCTGATAAGCCGCGAGTCCATCCAAAACCGCCGAAG
>n.538.6.bb_512
GTAGTTAGCCGGTGCTTCTTCTGCAGGTACCGTCACCACAAGCTTCGCCCCTGCTGAAAGCGGTTTACAACCCGAAGGCCGTCATCCCGCACGCGGCGTTGCTGCATCAGGCTTCCGCCCATTGTGCAATATTCCCCACTGCTGCCTCCCGTAGGAGTCTGGACCGTGTCTCAGTTCCAGTGTGGCTGGTCATCCTCTCAGGACCAGCTAGGGGNTCGTCGCCTAGGTGAGCCGTTACCCACTACTAGCTAATCCCATCTGGGCACATCTGATGG
>n.538.6.bb_513
GTAGTTAGCCGTGGCTTTCTGATTAGGTACCGTCAAGACGTGCATAGTTACTTACACATTTGTTCTTCCCTAATAACAGAGTTTTACGATCCGAAGACCTTCATCACTCACGCGGCGTTGCTCCGTCAGGCTTTCGCCCATTGCGGAAGATTCCCTACTGCTGCCTCCCGTAGGAGTCTGGACCGTGTCTCAGTTCCAGTGTGGCCGATCACCCTCTCAGGGTCGGCTACGCATCGTGCCTTGGTAAGCCGTTACCTTACCAACTAGCTAATGCGGCGCGGATCCATCTATAAGTGACAGCAAAACCGTCTTTCACTATTGAACCATGCGG
>n.538.6.bb_514
GTAGTTAGCCGTGGCTTTCTGATTAGGTACCGTCAAGACGTGCATAGTTACTTACACATTTGTTCTTCCCTAATAACAGAGTTTTACGATCCGAAGACCTTCATCACTCACGCGGCGTTGCTCCGTCAGGCTTTCGCCCATTGCGGAAGATTCCCTACTGCTGCCTCCCGTAGGAGTCTGGACCGTGTCTCAGTTCCAGTGTGGCCGATCACCCTCTCAGGGTCGGCTACGCATCGTTGCCTTGGTAAGCCGTTACCTTACCAACTAGCTAATGCGGCGCGGATCCATCTATAAGTGAC
>n.538.6.bb_515
GGAGTTAGCCGGTGCTTCTTCTGCGGGTAACGTCAATCGACAAGGTTATTAACCTTATCGCCTTCCTCCCCGCTGAAAGTACTTTACAACCCGAAGGCCTTCTTCATACACGCGGCATGGCTGCATCAGGCCTGCGCCCATTGTGCAATATTCCCCACTGCTGCCTCCCGTAGGAGTCTGGACCGTGTCTCAGTTCCAGTGTGGCTGGTCATCCTCTCAGACCAGCTAGGGATCGTCGCCTAGGTGAGCCGTTACCCACCTACTAGCTAATCCCATCTGGGCACATCTGATGGCAAGAGGCCCGAAGGTCCCCCTCTTTGGTCTTGCGACGTTATGCGGTATTAGCTA
>n.538.6.bb_516
GTAGTTAGCCGTGGCTTTCTGATTAGGTACCGTCAAGACGTGCATAGTTACTTACACATTTGTTCTTCCCTAATAACAGAGTTTTACGATCCGAAGACCTTCATCACTCACGCGGCGTTGCTCCGTCAGGCTCTCGCCCATTGCGGAAGATTCCCTACTGCTGCCTCCCGTAGGAGTCTGGACCGTGTCTCAGTTCCAGTGTGGCCGATCACCCTNTCAGGTCGCTACGCATCGTCGCCTTGGTAAGCCGTTACCTTACCAACTAGCTAATGCGGCGCGGATCCATCTATAAGTGACAGCAAAACCGTCTTTCACTATTGAACCATGCGGTTCAATATATTATCCGGTATT
>n.538.6.bb_517
GTAGTTAGCCGTGGCTTTCTGATTAGGTACCGTCAAGACGTGCATAGTTACTTACACATTTGTTCTTCCCTAATAACAGAGTTTTACGATCCGAAGACCTTCATCACTCACGCGGCGTTGCTCCGTCAAGCTTTCGCCCATTGCGGAAGATTCCCTACTGCTGCCTCCCGTAGGAGTCTGGACCGTGTCTCAGTTCCAGTGTGGCCGATCACCCTCTCAGGGTCGGCTACGCATCGTCGCCTTGGTAAGCCGTTACCTTACCAACTAGCTAATGCGGCGCGGATCCGTCTATAAGTGACAGCAAAACCGTCTTTCACTATTGAACCATGCGGTTCAATATATTATCCGGTATTAGCTCCGGTTTCCCCGAAGTTATCCCAGTCTTATAGGTAGGTT
>n.538.6.bb_518
GTAGTTAGCCGGTGCTTCTTCTGCAGGTACCGTCACCACAAGCTTCGCCCCTGCTGAAAGCGGTTTACAACCCGAAGGCCGTCATCCCGCACGCGGCGTTGCTGCATCAGGCTTCCGCCCATTGTGCAATATTCCCCACTGCTGCCTCCCGTGGGAGTCTGGGCCGTATCTCAGTCCCAATGTGGCCGGTCGCCCTCTCAGGCCGGCTACCCGGTCAAAGCCTTGGTAAGCCACTACCCCACCAACAAGCTGATAAGCCGCGAGTCCATCCAAAACCGCCG
>n.538.6.bb_519
GTAGTTAGCCGGTGCTTCTTCTGCAGGTACCGTCACCACAAGCTTCGCCCCTGCTGAAAGCGGTTTACAACCCGAAGGCCGTCATCCCGCACGCGGCGTTGCTGCATCAGGCTTCCGCCCATTGTGCAATATTCCCCACTGCTGCCTCCCGTAGGAGTCTGGGCCGTATCTCAGTCCCAATGTGGCCGGTCGCCCTCTCAGGCCGGCTACCCGTCAAAGCCTTGGTAAGCCACTACCCCACCAACAAGCTGATAAGCCGCGAGTCCATCCAAAACCG
>n.538.6.bb_520
GTAGTTAGCCGTGGCTTTCTGATTAGGTACCGTCAAGACGTGCATAGTTACTTACACATTTGTTCTTCCCTAATAACAGAGTTTTACGATCCGAAGACCTTCATCACTCACGCGGCGTTGCTCCGTCAGGCTTTCGCCCATTGCGGAAGATTCCCTACTGCTGCCTCCCGTAGGAGTCTGGACCGTGTCTCAGTTCCAGTGTGGCCGGTCACCCTCTCAGGGTCGGGCTACGCATCGTTGCCTTGGTAAGCCGTTACCTTACCAACTAGCTAATGCGGCGCGGATCCATCTATAAGTGACAGCAAAAGCCGTCTTTCACTATTGAACCATGCGGTTCAATATATTATCCGGTATTAGCTCCGGT
>n.538.6.bb_521
GTAGTTAGCCGTGGCTTTCTGATTAGGTACCGTCAAGACGTGCATAGTTACTTACACATTTGTTCTTCCCTAATAACAGAGTTTTACGATCCGAAGACCTTCATCACTCACGCGGCGTTGCTCCGTCAGGCTTTCGCCCATTGCGGAAGATTCCCTACTGCTGCCTCCCGTAGGAGTCTGGACCGTGTCTCAGTTCCAGTGTGGCCGATCACCCTCTCAGGGTCGGCTACGCATCGTTGCCTTGGTAAGCCGTTACCTTACCAACAAGCTAATGCGGCGCGGATCCATCTATAAGTGACAGCAAAACCGTCTTTCACTATTGAACCATGCGGTTCAATATATTATCCGGTATT
>n.538.6.bb_522
GTAGTTAGCCGTGGCTTTCTGATTAGGTACCGTCAAGACGTGCATAGTTACTTACACATTTGTTCTTCCCTAATAACAGAGTTTTACGATCCGAAGACCTTCATCACTCACGCGGCGTTGCTCCGTCAGGCTTTCGCCCATTGCGGAAGATTCCCTACTGCTGCCTCCCGTAGGAGTCTGGACCGTGTCTCAGTTCCAGTGTGGCCGATCACCCTCTCAGGGTCGGCTACGCATCGTTGCCTTGGTAAGCCGTTACCTTACAACTAGCTAATGCGGCGCGGATCCATCTATAAGTGACAGCAAAACCGTCTTTCACTATTGAACCATGCGGTTCAATATATT
>n.538.6.bb_523
GTAGTTAGCCGGTGCTTCTTCTGCAGGTACCGTCACCACAAGCTTCGCCCCTGCTGAAAGCGGTTTACAACCCGAAGGCCGTCATCCCGCACGCGGCGTTGCTGCATCAGGCTTCCGCCCATTGTGCAATATTCCCCACTGCTGCCTCCCGTAGGAGTCTGGGCCGTATCTCAGTCCCAATGTGGCCGGTCGCCCTCTCAGGCCGGCTACCCGTCAAAGCCTTGGTAAGCCACTACCCCACCAACAAGCTGATAAGCCGCGAGTCCATCCAAAACCGCCGAAGCTTTCCAACCCCACCATGCAGCAAGGATTCCTATCCGGTATTAGCCCCAGTTTCCTGAAGTTATCCCGAAGTCAAG
>n.538.6.bb_524
GTAGTTAGCCGGTGCTTCTTCTGCAGGTACCGTCACCACAAGCTTCGCCCCTGCTGAAAGCGGTTTACAACCCGAAGGCCGTCATCCCGCACGCGGCGTTGCTGCATCAGGCTTCCGCCCATTGTGCAATATTCCCCACTGCTGCCTCCCGTAGGAGTCTGGGCCGTATCTCAGTCCCAATGTGGCCGTCGCCCTCTCAGGCCGGCTACCCGTCAAAGCCTTGGTAAGCCACTACCCACCAACAAGCTGATAAGCCGCGAGTCCAT
>n.538.6.bb_525
GTAGTTAGCCGTGGCTTTCTGATTAGGTACCGTCAAGACGTGCATAGTTACTTACACATTTGTTCTTCCCTAATAACAGAGTTTTACGATCCGAAGACCTTCATCACTCACGCGGCGTTGCTCGGTCAGACTTTCGTCCATTGCCGAAGATTCCCTACTGCTGCCTCCCGTAGGAGTCTGGGCCGTGTCTCAGTCCCAGTGTGGCCGATCACCCTCTCAGGGTCGGCTATGCATCGTGGCCTTGGTGAGCCGTTACCTCACCAACTAGCTAATGCACCGGCGGGTCCATCCAT
>n.538.6.bb_526
GTAGTTAGCCGTGGCTTTCTGATTAGGTACCGTCAAGACGTGCATAGTTACTTACACATTTGTTCTTCCCTAATAACAGAGTTTTACGATCCGAAGACCTTCATCACTCACGCGGCGTTGCTCCGTCAGGCTTTCGCCCATTGCGGAAGATTCCCTACTGCTGCCTCCCGTAGGAGTCTGGACCGTGTCTCAGTTCCAGTGTGGCCGATCACCCTCTCAGGGTCGGCTACGCATCGTTGCCTTGGTAAGCCGTTACCTTACAACTAGCTAATGCGGCGCGGATCCATCT
>n.538.6.bb_527
GTAGTTAGCCGTGGCTTTCTGATTAGGTACCGTCAAGACGTGCATAGTTACTTACACATTTGTTCTTCCCTAATAACAGAGTTTTACGATCCGAAGACCTTCATCACTCACGCGGCGTTGCTCCGTCAGGCTTTCGCCCATTGCGGAAGATTCCCTACTGCTGCCTCCCGTAGGAGTCTGGACCGTGTCTCAGTTCCAGTGTGGCCGATCACCCTCTCAGGGGTCGGCTGCGCATCGTGCCTTGGTAAGCCGTTACCTTACCAACTAGCTAATGCGGCGCGGATCCATCTATAAGTGA
>n.538.6.bb_528
GTAGTTAGCCGGTGCTTCTTCTGCAGGTACCGTCACCACAAGCTTCGCCCCTGCTGAAAGCGGTTTACAACCCGAAGGCCGTCATCCCGCACGCGGCGTTGCTGCATCAGGCTTCCGCCCATTGTGCAATATTCCCCACTGCTGCCTCCCGTAGGAGTCTGGGCCGTATCTCAGTCCCAATGTGGCCGGTCGCCCTCTCAGGCCGGCTACCCGTCAAAGCCTTGGTAAGCCACTACCCCACCAACAAGCTGATAAGCCGCGAGTCCATCCAAAACCGCCGAAGCTTTCCAACCCCACCATGCAGCAAGGATTCCTATCCGGTATTAGCCCCAGTTTCCTGAAGTTA
>n.538.6.bb_529
GTAGTTAGCCGTGGCTTTCTGATTAGGTACCGTCAAGACGTGCATAGTTACTTACACATTTGTTCTTCCCTAATAACAGAGTTTTACGATCCGAAGACCTTCATCACTCACGCGGCGTTGCTCCGTCAGGCTTTCGCCCATTGCGGAAGATTCCCTACTGCTGCCTCCCGTAGGAGTCTGGACCGTGTCTCAGTTCCAGTGTGGCCGATCACCCTCTCAGGGTCGGCTACGCACCGTTGCCTTGGTAAGCCGTTACCTTACCAACTAGCTAATGCGGCGCGGATCCATCTATAAGTGACAGCAAAACCGTCTTTCACTATTGAACCATGCGGTTCAATATATTATCCGGTATT
>n.538.6.bb_530
GTAGTTAGCCGTGGCTTTCTGATTAGGTACCGTCAAGACGTGCATAGTTACTTACACATTTGTTCTTCCCTAATAACAGAGTTTTACGATCCGAAGACCTTCATCACTCACGCGGCGTTGCTCCGTCAGGCTTTCGCCCATTGCGGAAGATTCCCTACTGCTGCCTCCCGTAGGAGTCTGGACCGTGTCTCAGTTCCAGTGTGGCCGATCACCCTCTCAGGTCGGCTACGCATCGTTGCCTTGGTAAGCCGTTACCTTACCAACTAGCTAATGCGGCGCGGATCCATCTATGAGTGACAGCAAAAACCGTCTTTCACTATTGAACCATGCGGTTCAATATATTATCCGGTATTAGCTCCGGTTTCCCGAAGTTATCCCAGTCTTATAGGTAGGTTATCCCACGTGTTACTCACCCCGTCCCGCCCGCTAACGTCAGAGGAGCAAGCCTCCTCGTCTGTTCCGCTCGACTTGCATGTATTAGGGCACGCCGCCAGCGTT
>n.538.6.bb_531
GTAGTTAGCCGTGGCTTTCTGATTAGGTACCGTCAAGACGTGCATAGTTACTTACACATTTGTTCTTCCCTAATAACAGAGTTTTACGATCCGAAGACCTTCATCACTCACGCGGCGTTGCTCCGTCAGGCTTTCGCCCATTGCGGAAGATTCCCTACTGCTGCCTCCCGTAGGAGTCTGGACCGTGTCTCAGTTCCAGTGTGGCCGATCACCCTCTCAGGGTCGGCTACGCATCGTTGCCTTGGTAAGCCGTTACCTTACCAACTAGCTAATGCGGCGCGGATCCATCTATAAGTGACAGCAAAACCGTCTTTCACTATTGAACCATGCGGTTCAATATATTATCCGGTATTA
>n.538.6.bb_532
TAGTTAGCCGTGGCTTTCTGATTAGGTACCGTCAAGACGTGCATAGTTACTTACACATTTGTTCTTCCCTAATAACAGAGTTTTACGATCCGAAGACCTTCATCACTCACGCGGCGTTGCTCCGTCAGGCTTTCGCCCATTGCGGAAGATTCCCTACTGCTGCCTCCCGTAGGAGTCTGGACCGTGTCTCAGTTCCAGTGTGGCCGATCACCCTCTCAGGGTCGGCTACGCATCGTCGCCTTGGTAAGCCGTTACCTTACCAACTAGCTAATGCGGCGCGGATCCATCTATAAGTGACAGCAAAACCATCTTTCACTATTGAACCAT
>n.538.6.bb_533
GGAGTTAGCCGGTGCTTCTTCTGCGGGTAACGTCAATCGACAAGGTTATTAACCTTATCGCCTTCCTCCCCGCTGAAAGTACTTTACAACCCGAAGGCCTTCTTCATACACGCGGCATGGCTGCATCAGGCTTGCGCCCATTGTGCAATATTCCCCACTGCTGCCTCCCGTAGGAGTCTGGACCGTGTCTCAGTTCCAGTGTGGCTGGTCATCCTCTCAGACCAGCTAGGGATCGTCGCCTAGGTGAGCCGTTACCCACCTACTAGCTAATCCCATCTGGGCACATCTGATGGCAAGAGGCCCGAAGGTCCCCCTCTTTGGTCTTGCGACGTTATGCGGTATTAGCTACCGT
>n.538.6.bb_534
GTAGTTAGCCGTGGCTTTCTGATTAGGTACCGTCAAGACGTGCATAGTTACTTACACATTTGTTCTTCCCTAATAACAGAGTTTTACGATCCGAAGACCTTCATCACTCACGCGGCGTTGCTCCGTCAGGCTTTCGCCCATTGCGGAAGATTCCCTACTGCTGCCTCCCGTAGGAGTCTGGACCGTGTCTCAGTTCCAGTGTGGCCGATCACCCTCTCAGGGTCGGCTACGCATCGTTGCCTTGGTAAGCCGTTACCTTACCAACTAGCTAATGCGGCGCGGATCCATCTATAAGTGACAGCAAAACCGTCTTTCACTATTGAACCATGCGGTTCAATATATTATCCGGTATT
>n.538.6.bb_535
GTAGTTAGCCGGTGCTTCTTCTGCAGGTACCGTCACCACAAGCTTCGCCCCTGCTGAAAGCGGTTTACAACCCGAAGGCCGTCATCCCGCACGCGGCGTTGCTGCATCAGGCTTCCGCCCATTGTGCAATATTCCCCACTGCTGCCTCCCGTAGGAGTCTGGGCCGTATCTCAGTCCCAATGTGGCCGGTCGCCCTCTCAGGCCGGCTACCCGTCAAAGCCTTGGTAAGCCACTACCCACCAACAAGCTGATAAGCCGCGAGTCCATCCAAAACCGCCGAAGCTTTCCAACCCCACCATGCAGCAAGGATTCCTATCCGGTATTAGCCCCAGTTTCC
>n.538.6.bb_536
GTAGTTAGCCGTGGCTTTCTGATTAGGTACCGTCAAGACGTGCATAGTTACTTACACATTTGTTCTTCCCTAATAACAGAGTTTTACGATCCGAAGACCTTCATCACTCACGCGGCGTTGCTCCGTCAGGCTTTCGCCCATTGCGGAAGATTCCCTACTGCTGCCTCCCGTAGGAGTCTGGACCGTGTCTCAGTTCCAGTGTGGCCGATCACCCTCTCAGGGTCGGCTACGCATCGTTGCCTTGGTAAGCCGTTACCTTACAACTAGCTAATGCGGCGCGGATCCATCTATAAGTGACAGCAAAACCGTCTTTCACTATTGAACCATGCGGTTCAATATATT
>n.538.6.bb_537
GTAGTTAGCCGGTGCTTCTTCTGCAGGTACCGTCACCACAAGCTTCGCCCCTGCTGAAAGCGGTTTACAACCCGAAGGCCGTCATCCCGCACGCGGCGTTGCTGCATCAGGCTTCCGCCCATTGTGCAATATTCCCCACTGCTGCCTCCCGTAGGAGTCTGGGCCGTATCTCAGTCCCAATGTGGCCGGTCGCCCTCTCAGGCCGGCTACCCGTCAAAGCCTTGGTAAGCCACTACCCCACCAACAAGCTGATAAGCCGCGAGTCCATCCAAAACCGC
>n.538.6.bb_538
GTAGTTAGCCGGTGCTTCTTCTGCAGGTACCGTCACCACAAGCTTCGCCCCTGCTGAAAGCGGTTTACAACCCGAAGGCCGTCATCCCGCACGCGGCGTTGCTGCATCAGGCTTCCGCCCATTGTGCAATATTCCCCACTGCTGCCTCCCGTAGGAGTCTGGGCCGTATCTCAGTCCCAATGTGGCCGGTCGCCCTCTCAGGCCGGCTACCCGGTCAAAGCCTTGGTAAGCCACTACCCCACCAACAAGCTGATAAGCCGCGAGTCCATCCACAACCGCCGAAAGCTTTCCAACCCCACATGCAGCAAGGATTCCTATCCGGTATTAGCCCCAGTTTCCTGAAGTT
>n.538.6.bb_539
GTAGTTAGCCGTGGCTTTCTGATTAGGTACCGTCAAGACGTGCATAGTTACTTACACATTTGTTCTTCCCTAATAACAGAGTTTTACGATCCGAAGACCTTCATCACTCACGCGGCGTTGCTCCGTCAGGCTTTCGCCCATTGCGGAAGATTCCCTACTGCTGCCTCCCGTAGGAGTCTGGACCGTGTCTCAGTTCCAGTGTGGCCGATCACCCTCTCAGGGTCGGCTACGCATCGTTGCCTTGGTAAGCCGTTACCTTACCAACTAGCTAATGCGGCGCGGATCCATCTATAAGTGACAGCAAAACCGTCTTTCACTATTGAACCATGCGGTTCAATATATTATCCGGTATTAGCTCC
>n.538.6.bb_540
GGAGTTAGCCGGTGCTTCTTCTGCGGGTAACGTCAATCGACAAGGTTATTAACCTTATCGCCTTCCTCCCCGCTGAAAGTACTTTACAACCCGAAGGCCTTCTTCATACACGCGGCATGGCTGCATCAGGCTTGCGCCCATTGTGCAATATTCCCCACTGCTGCCTCCCGTAGGAGTCTGGACCGTGTCTCAGTTCCAGTGTGGCTGGTCATCCTCTCAGACCAGCCAGGGATCGTCGCCTAGGTGAGCCGTTACCCACCTGCTAGCTAATCCCATCTGGGCACATCTGATGGCAAGAGGCCCGAAGGT
>n.538.6.bb_541
TAGTTAGCCGTGGCTTTCTGATTAGGTACCGTCAAGACGTGCATAGTTACTTACACATTTGTTCTTCCCTAATAACAGAGTTTTACGATCCGAAGACCTTCATCACTCACGCGGCGTTGCTCCGTCAGGCTTTCGCCCATTGCCGAAGATTCCCTACTGCTGCCTCCCGTAGGAGTCTGGGCCGTGTCTCAGTCCCAGTGTGGCCGATCACCCTCTCAGGGTCGGCTATGCAT
>n.538.6.bb_542
GTAGTTAGCCGTGGCTTTCTGATTAGGTACCGTCAAGACGTGCATAGTTACTTACACATTTGTTCTTCCCTAATAACAGAGTTTTACGATCCGAAGACCTTCATCACTCACGCGGCGTTGCTCCGTCAGGCTTTCGCCCATTGCGGAAGATTCCCTACTGCTGCCTCCCGTAGGAGTCTGGACCGTGTCTCAGTTCCAGTGTGGCCGATCACCCTCTCAGGGTCGGCTACGCATCGTTGCCTTGGTAAGCCGTTACCTTACCAACTAGCTAATGCGGCGCGGATCCATCTATAAGTGACAGCAAAACCGTCTTTCACTATTGAACCATGCGGTTCAATATATTATCCGGTATTAGCTCCG
>n.538.6.bb_543
GGAGTTAGCCGGTGCTTCTTCTGCGGGTAACGTCAATCGACAAGGTTATTAACCTTATCGCCTTCCTCCCCGCTGAAAGTACTTTACAACCCGAAGGCCTTCTTCATACACGCGGCATGGCTGCATCAGGCTTGCGCCCATTGTGCAATATTCCCCACTGCTGCCTCCGTAGGAGTCTGGACCGTGTCTCAGTTCCAGTGTGGCTGGTCATCCTCTCAGACCAGCTAGGGATCGTCGCCTAGGTGAGCCGTTACCCACCTACTAGCTAATCCCATCTGGGCACATCTGATGGCAAGAGGCCCGAAGGTCCCCTCTTTGGTCTTGCGACGTTATGCGGTAT
>n.538.6.bb_544
GTAGTTAGCCGGTGCTTCTTCTGCAGGTACCGTCACCACAAGCTTCGCCCCTGCTGAAAGCGGTTTACAACCCGAAGGCCGTCATCCCGCACGCGGCGTTGCTGCATCAGGCTTCCGCCCATTGTGCAATATTCCCCACTGCTGCCTCCCGTAGGAGTCTGGGCCGTATCTCAGTCCCAATGTGGCCGGTCGCCCTCTCAGGCCGGCTACCCGTCAAAGCCTTGGTAAGCCACTACCCCACCAACAAGCTGACAAGCCGCGAGTCCATCCAAAACCGCCGAAGCTTTCCAACCCCCACCATGCAGCAAGGATTCCTATCCGGT
>n.538.6.bb_545
GGAGTTAGCCGGTGCTTCTTCTGCGGGTAACGTCAATCGACAAGGTTATTAACCTTATCGCCTTCCTCCCCGCTGAAAGTACTTTACAACCCGAAGGCCTTCTTCATACACGCGGCATGGCTGCATCAGGCTTGCGCCCATTGTGCAATATTCCCCACTGCTGCCTCCGTAGGAGTCTGGACCGTGTCTCAGTTCCAGTGTGGCTGGTCATCCTCTCAGACCAGCTAGGGATCGTCGCCTAGGTGAGCCGTTACCCACCTACTAGCTAATCCCATCTGGGCACATCTGATGGCAAGAGGCCCGAAGGTCCCCCTCTTTGGTCTTGCGACGTTATGCGGTATTAGCTACCGTTTCCAGT
>n.538.6.bb_546
GTAGTTAGCCGGTGCTTCTTCTGCAGGTACCGTCACCACAAGCTTCGCCCCTGCTGAAAGCGGTTTACAACCCGAAGGCCGTCATCCCGCACGCGGCGTTGCTGCATCAGGCTTCCGCCCATTGTGCAATATTCCCCACTGCTGCCTCCCGTAGGAGTCTGGGCCGTATCTCAGTCCCAATGTGGCCGGTCGCCCTCTCAGGCCGGCTACCCGTCAAAGCCTTGGTAAGCCACTACCCACCAACAAGCTGATAAGCCGCGAGTCCAT
>n.538.6.bb_547
GTAGTTAGCCGGTGCTTCTTCTGCAGGTACCGTCACCACAAGCTTCGCCCCTGCTGAAAGCGGTTTACAACCCGAAGGCCGTCATCCCGCACGCGGCGTTGCTGCATCAGGCTTCCGCCCATTGTGCAATATTCCCCACTGCTGCCTCCCGTAGGAGTCTGGGCCGTATCTCAGTCCCAATGTGGCCGGTCGCCCTCTCAGGCCGGCTACCCGTCAAAGCCTTGGTAAGCCACTACCCCACCAACAAGCTGATAAGCCGCGAGTCCATCCGAAACCGCCGGAGCTTTCCAACCCCACCATGCAGCAAGGATTCCTATCCGGTAT
>n.538.6.bb_548
GTAGTTAGCCGTGGCTTTCTGATTAGGTACCGTCAAGACGTGCATAGTTACTTACACATTTATTCTTCCCTAATAACAGAGTTTTACGATCCGAAGACCTTCATCACTCACGCGGCGTTGCTCCGTCAGGCTTTCGCCCATTGCGGAAGATTCCCTACTGCTGCCTCCCGTAGGAGTCTGGACCGTGTCTCAGTTCCAGTGTGGCCGATCACCCTNTCAGGGTCGGCTACGCATCGTTGCCTTGGTAAGCCGTTACCTTACCAACTAGCTAATGCGGCGCGGATCCATCTATAAGTGACAGCAAAACCGTCTTTCACTATTGAACCATGCGGTTCAATATATTATCCGGTATT
>n.538.6.bb_549
GTAGTTAGCCGTGGCTTTCTGATTAGGTACCGTCAAGACGTGCATAGTTACTTACACATTTGTTCTTCCCTAATAACAGAGTTTTACGATCCGAAGACCTTCATCACTCACGCGGCGTTGCTCCGTCAGGCTTTCGCCCATTGCGGAAGATTCCCTACTGCTGCCTCCCGTAGGAGTCTGGACCGTGTCTCAGTTCCAGTGTGGCCGATCACCCTCTCAGGGTCGGCTACGCATCGTTGCCTTGGTAAGCCGTTACCTTACCAACTAGCTAATGCGGCGCGGATCCATCTATAAGTGACAGCAAAACCGT
>n.538.6.bb_550
GTAGTTAGCCGTGGCTTTCTGATTAGGTACCGTCAAGACGTGCATAGTTACTTACACATTTGTTCTTCCCTAATAACAGAGTTTTACGATCCGAAGACCTTCATCACTCACGCGGCGTTGCTCCGTCAGGCTTTCGCCCATTGCGGAAGATTCCCTACTGCTGCCTCCCGTAGGAGTCTGGACCGTGTCTCAGTTCCAGTGTGGCCGATCACCCTCTCAGGGTCGGCTACGCATCGTTGCCTTGGTAAGCCGTTACCTTACCAACTAGCTAATGCGGCGCGGATCCATCTATAAGTGACAGCAAAACCGTCTTTCACTATTGAACCATGCGGTTCAATATATTATCCGGTATT
>n.538.6.bb_551
GTAGTTAGCCGGTGCTTCTTCTGCAGGTACCGTCACCACAAGCTTCGCCCCTGCTGAAAGCGGTTTACAACCCGAAGGCCGTCATCCCGCACGCGGCGTTGCTGCATCAGGCTTCCGCCCATTGTGCAATATTCCCCACTGCTGCCTCCCGTAGGAGTCTGGGCCGTATCTCAGTCCCAATGTGGCCGGTCGCCCTCTCAGGCCGGCTACCCGTCAAAGCCTTGGTAAGCCACTACCCACCAACAAGCTGATAAGCCGCGAGTCCATCCAAAACCGCC
>n.538.6.bb_552
GTAGTTAGCCGTGGCTTTCTGATTAGGTACCGTCAAGACGTGCATAGTTACTTACACATTTGTTCTTCCCTAATAACAGAGTTTTACGATCCGAAGACCTTCATCACTCACGCGGCGTTGCTCCGTCAGGCTTTCGCCCATTGCGGAAGATTCCCTACTGCTGCCTCCCGTAGGAGTCTGGACCGTGTCTCAGTTCCAGTGTGGCCGATCACCCTCTCAGGGTCGGCTACGCATCGTTGCCTTGGTAAGCCGTCACCTTACCAACTAGCTAATGCGGCGCGGATCCATCTATAAGTGACAGCAAAACCGTCTTTCACTATTGAGCCATGCGGTTCAATATATTATCCGGTATTAGCTCCGGTTTCCCGA
>n.538.6.bb_553
GGAGTTAGCCGGTGCTTCTTCTGCGGGTAACGTCGATCGACAAGGTTATTAACCTTATCGCCTTCCTCCCCGCTGAAAGTACTTTACAACCCGAAGGCCTTCTTCATACACGCGGCATGGCTGCATCAGGCTTGCGCCCATTGTGCAATATTCCCCACTGCTGCCTCCCGTAGGAGTCTGGACCGTGTCTCAGTTCCAGTGTGGCTGGTCATCCTCTCAGACCAGCTAGGGGATCGTCGCCTAGGTGAGCCGTTACCCACCTACTAGCTAATCCCATCTGGGCACATCTGATGGCAAGAGGCCCGAAGGTCCCCCTCTTTGGTCTTGCGACGTTATGCGGTATTAGCTA
>n.538.6.bb_554
GTAGTTAGCCGTGGCTTTCTGATTAGGTACCGTCAAGACGTGCATAGTTACTTACACATTTGTTCTTCCCTAATAACAGAGTTTTACGATCCGAAGACCTTCATCACTCACGCGGCGTTGCTCCGTCAGGCTTTCGCCCATTGCGGAAGATTCCCTACTGCTGCCTCCCGTAGGAGTCTGGACCGTGTCTCAGTTCCAGTGTGGCCGATCACCCTCTCAGGGTCGGCTACGCATCGTTGCCTTGGTAAGCCGTTACCTTACCAACTAGCTAATGCGGCGCGGATCCATCTATAAGTGACAGCAAAACCGTCTTTCACTATTGAACCATGCGGTTCAATATATTATCCGGTATTAGCTCCGGT
>n.538.6.bb_555
GGAGTTAGCCGGTGCTTCTTCTGCGGGTAACGTCAATCGACAAGGTTATTAACCTTATCGCCTTCCTCCCCGCTGAAAGTACTTTACAACCCGAAGGCCTTCTTCATACACGCGGCATGGCTGCATCAGGCTTGCGCCCATTGTGCAATATTCCCCACTGCTGCCTCCCGTAGGAGTCTGGACCGTGTCTCAGTTCCAGTGTGGCTGGTCATCCTCTCAGACCAGCTAGGGATCGTCGCCTAGGTGAGCCGTTACCCACCTACTAGCTAATCCCATCTGGGCACATCTGATGGCAAG
>n.538.6.bb_556
GTAGTTAGCCGTGGCTTTCTGATTAGGTACCGTCAAGACGTGCGATAGTTACTTACACATTTGTTCTTCCCTAATAACAGAGTTTTACGTATCCGAAGAACCTTCATCACTCACGCGGCGTTGCTCCGTCAGGCTTTCGCCCATTGCGGAAGATTCCCTACTGCTGCCTCCCGTAGGAGTCTGGACCGTGTCTCAGTTCCAGTGTGGCTGGTCATCCTCTCAGACCCAGCTAGGGATCGGTCGCCTAGGTGAGCCGTTACCCACCTACTAGC
>n.538.6.bb_557
GTAGTTAGCCGGTGCTTCTTCTGCAGGTACCGTCACCACAAGCTTCGCCCCTGCTGAAAGCGGTTTACAACCCGAAGGCCGTCATCCCGCACGCGGCGTTGCTGCATCAGGCTTCCGCCCATTGTGCAATATTCCCCACTGCTGCCTCCCGTAGGAGTCTGGGCCGTATCTCAGTCCCAATGTGGCCGGTCGCCCTCTCAGGCCGGCTACCCGTCAAAGCCTTGGTAAGCCACTACCCACCAACAAGCTGATAAGCCGCGAGTCCATCCAAAAACCGC
>n.538.6.bb_558
GTAGTTAGCCGGTGCTTCTTCTGCAGGTACCGTCACCACAAGCTTCGCCCCTGCTGAAAGCGGTTTACAACCCGAAGGCCGTCATCCCGCACGCGGCGTTGCTGCATCAGGCTTCCGCCCATTGTGCAATATTCCCCACTGCTGCCTCCCGTAGGAGTCTGGGCCGTATCTCAGTCCCAATGTGGCCGGTCGCCCTCTCAGGCCGGCTACCCGTCAAAGCCTTGGTAAGCCACTACCCCACCAACAAGCTGATAAGCCGCGAGTCCATCCAAAACCGCCGAAGCTTTCCAACCCCCACCATGCAGCAAGGATTCCTATCCGGTATTAGCCCCAGTTTCCTGAAGTTATCCCAAAGTCAAGGGCAGGTTACTCACGTGTTACTCACCCGTTCGCCA
>n.538.6.bb_559
GTAGTTAGCCGGTGCTTCTTCTGCAGGTACCGTCACCACAAGCTTCGCCCCTGCTGAAGGCGGTTTACAACCCGAAGGCCGTCATCCCGCACGCGGCGTTGCTGCATCAGGCTTCCGCCCATTGTGCAATATTCCCCACTGCTGCCTCCCGTAGGAGTCTGGGCCGTATCTCAGTCCCAATGTGGCCGGTCGCCCTCTCAGGCCGGCTACCCGTCAAAGCCTTGGTAAGCCACTACCCCACCAACAAGCTGATAAGCCGCGAGTCCATCCAAAACCGCCGAA
>n.538.6.bb_560
GTAGTTAGCCGTGGCTTTCTGATTAGGTACCGTCAAGACGTGCATAGTTACTTACACATTTGTTCTTCCCTAATAACAGAGTTTTACGATCCGAAGACCTTCATCACTCACGCGGCGTTGCTCCGTCAGGCTTTCGCCCATTGCGGAAGATTCCCTACTGCTGCCTCCCGTAGGAGTCTGGACCGTGTCTCAGTTCCAGTGTGGCCGATCACCCTCTCAGGGTCGGCTACGCATCGTTGCCTTGGTAAGCCGTTACCTTACCAACTAGCTAATGCGGCGNGGATCCATCTATAAGTGACAGCAAAACCGTCTTCACTAT
>n.538.6.bb_561
GGAGTTAGCCGGTGCTTCTTCTGCGGGTAACGTCAATCGACAAGGTTATTAACCTTATCGCCTTCCTCCCCGCTGAAAGTACTTTACAACCCGAAGGCCTTCTTCATACACGCGGCATGGCTGCATCAGGCTTGCGCCCATTGTGCAATATTCCCCACTGCTGCCTCCGTAGGAGTCTGGACCGTGTCTCAGTTCCAGTGTGGCTGGTCATCCTCTCAGACCAGCTAGGGATCGTCGCCTAGGTGAGCCGTTACCCACCTACTAGCTAATCCCATCTGGGCACATCTGATGGC
>n.538.6.bb_562
GGAGTTAGCCGGTGCTTCTTCTGCGGGTAACGTCAATTGCTGAGGTTATTAACCTCAACACCTTCCTCCCCGCTGAAAGTACTTTACAACCCGAAGGCCTTCTTCATACACGCGGCATGGCTGCATCAGGCTTGCGCCCATTGTGCAATATTCCCCACTGCTGCCTCCCGTAGGAGTCTGGACCGTGTCTCAGTTCCAGTGTGGC
>n.538.6.bb_563
GTAGTTAGCCGTGGCTTTCTGATTAGGTACCGTCAAGACGTGCATAGTTACTTACACATTTGTTCTTCCCTAATAACAGAGTTTTACGATCCGAAGACCTTCATCACTCACGCGGCGTTGCTCCGTCAGGCTTTCGCCCATTGCGGAAGATTCCCTACTGCTGCCTCCCGTAGGAGTCTGGACCGTGTCTCAGTTCCAGTGTGGCCGATCACCCTCTCAGGGTCGGCTACGCATCGTTGCCTTGGTAAGCCGTTACCTTACCAACTAGCTAATGCGGCGCGGATCCATCTATAAGTGACAGCAAACCGTCT
>n.538.6.bb_564
TAGTTAGCCGTGGCTTTCTGATTAGGTACCGTCAAGACGTGCATAGTTACTTACACATTTGTTCTTCCCTAATAACAGAGTTTTACGATCCGAAGACCTTCATCACTCACGCGGCGTTGCTCCGTCAGGCTTTCGCCCATTGCGGAAGATTCCCTACTGCTGCCTCCCGTAGGAGTCTGGACCGTGTCTCAGTTCCAGTGTGGCCGATCACCCTCTCAGGGTCGGGCTACGCATCGTTGCCTTGGTAAGCCGTTACCTTACCAACTAGCTAATGCGGCGCGGATCCATCTATAAGTGACAGCAAAACCGTCTTTCACTATTGAACCATGCGGTTCAATATATTATCCGGTATT
>n.538.6.bb_565
GTAGTTAGCCGTGGCTTTCTGATTAGGTACCGTCAAGACGTGCATAGTTACTTACACATTTATTCTTCCCTAATAACAGAGTTTTACGATCCGAAGACCTTCATCACTCACGCGGCGTTGCTCCGTCAGGCTTTCGCCCATTGCGGAAGATTCCCTACTGCTGCCTCCCGTAGGAGTCTGGACCGTGTCTCAGTTCCAGTGTGGCCGATCACCCTCTCAGGGTCGGCTACGCATCGTTGCCTTGGTAAGCCGTTACCTTACCAACTAGCTAATGCGGCGCGGATCCATCTATAAGTGACAGCAAAAACCGTCTTTCAC
>n.538.6.bb_566
AGTTAGCCGGTGCTTCTTCTGCAGGTACCGTCACCACAAGCTTCGCCCCTGCTGAAAGTACTTTACAACCCGAAGGCCTTCTTCATACACGCGGCATGGCTGCATCAGGCTTGCGCCCATTGTGCAATATTCCCCACTGCTGCCTCCCGTAGGAGTCTGGACCGTGTCTCAGTTCCAGTGTGGCTGGTCATCCTCTCAGACCAGCTAGGGATCGTCGCCTAGGTGAGCCGTTACCCCACCTACTAGCTAATCCCATCTGGGCACATCTGATGGC
>n.538.6.bb_567
GTAGTTAGCCGGTGCTTCTTCTGCAGGTACCGTCACCACAAGCTTCGCCCCTGCTGAAAGCGGTTTACAACCCGAAGGCCGTCATCCCGCACGCGGCGTTGCTGCATCAGGCTTCCGCCCATTGTGCAATATTCCCCACTGCTGCCTCCCGTAGGAGTCTGGGCCGTATCTCAGTCCCAATGTGGCCGGTCGCCCTCTCAGGCCGGCTACCCGTCAAAGCCTTGGTAAGCCACTACCCCACCAACAAGCTGATAAGCCGCGAGTCCAT
>n.538.6.bb_568
GTAGTTAGCCGTGGCTTTCTGATTAGGTACCGTCAAGACGTGCATAGTTACTTACACATTTGTTCTTCCCTAGTAACAGAGTTTTACGATCCGAAGACCTTCATCACTCACGCGGCGTTGCTCCGTCAGGCTTTCGCCCATTGCGGAAGATTCCCTACTGCTGCCTCCCGTAGGAGTCTGGACCGTGTCTCAGTTCCAGTGTGGCCGATCACCCTCTCAGGGTCGGCTACGCATCGTTGCCTTGGTAAGCCGTTACCTTACCAACTAGCTAATGCGGCGCGGATCCATCTATAAGTGACAGCAAAACCGTCTTTCACTATTGAACCATGCGGTTC
>n.538.6.bb_569
GTAGTTAGCCGTGGCTTTCTGATTAGGTACCGTCAAGAACGTGCATAGTTACTTACGACATTTGCTCTTCCCTAATAACAGAGTTTTACGATCCGAAGACCTTCATCACTCACGCGGCGTTGCTCCGTCAGGCTTTCGCCCATTGCGGAAGATTCCCTACTGCTGCCTCCCGTAGGAGTCTGGACCGTGTCTCAGTTCCAGTGTGGCCGATCACCCTNTCAGGTCGGCTACGCATCGTTGCCTTGGTAAGCCGTTACCTTACCAACTAGCTAATGCGGCGCGGATCCATCTATAAGTGACAGCAAAACCGTCTTTCACTATTGAACCATGCGGTTCAATATATTATCCGGTATTAGCT
>n.538.6.bb_570
GTAGTTAGCCGTGGCTTTCTGATTAGGTACCGTCAAGACGTGCATAGTTACTTACACATTTNTTCTTCCCTAATAACAGAGTTTTACGATCCGAAGACCTTCATCACTCACGCGGCGTTGCTCCGTCAGGCTTTCGCCCATTGCGGAAGATTCCCTACTGCTGCCTCCCGTAGGAGTCTGGACCGTGTCTCAGTTCCAGTGTGGCCGATCACCCTCTCAGGGTCGGCTACGCATCGTTGCCTTGGTAAGCCGTTACCTTACCAACTAGCTAATGCGGCGCGGATCCATCTATAAGTGACAGCAAAACCGTCTTTCACTATTGAACCATGCGG
>n.538.6.bb_571
GTAGTTAGCCGTGGCTTTCTGATTAGGTACCGTCAAGACGTGCATAGTTACTTACACATTTTGTTCTTCCCTAAATAACAGAGTTTTACGATCCGAAGACCTTCATCACTCACGCGGCGTTGCTCCGTCAGGCTTTCGCCCATTGCGGAAGATTCCCTACTGCTGCCTCCCGTAGGAGTCTGGACCGTGTCTCAGTTCCAGTGTGG
>n.538.6.bb_572
GTAGTTAGCCGGTGCTTCTTCTGCAGGTAACCGTCACCACAAGCTTCGCCCTGCTGAAGCGGTTACAACCGAGCGTCATCCGCACGCGGCGTTGCTGCATCAGGGTTCCCCCCATTGCCGAAGATTCCCTACTGCTGCCTCCCGTAGGAGTCTGGGCCGTGTCTCAGTCCCAGTGTGGCCGATCACCCTCTCAGGTCGGCTATGTATCGTTGCCTAGGTAGGCCATTACCCTACCTACTAGCTAAATACAACGCAGGTCCATCTACTAGCGATGCAATTGCATCTTTCAAGCAT
>n.538.6.bb_573
GTAGTTAGCCGTGGCTTTCTGATTAGGTACCGTCAAGACGTGCATAGTTACTTACACATTTGTTCTTCCCTAATAACAGAGTTTTACGATCCGAAGACCTTCATCACTCACGCGGCGTTGCTCCGTCAGGCTTTCGCCCATTGCGGAATATTCCCCACTGCTGCCTCCCGTAGGAGTCTGGGCCGTATCTCAGTCCCAATGTGGCCGGTCGCCCTCTCAGGGCCGGGCTACCCCGTCAAAGCCTTGGTAAGCCAC
>n.538.6.bb_574
GTAGTTAGCCGGTGCTTTCTGATTAGGTACCGTCAAGACGTGCATAGTTACTTACACATTTGTTCTTCCCTAATAACAGAGTTTTACGATCCGAAGACCTTCATCACTCACGCGGCGTTGCTCCGTCAGGCTTTCGCCCATTGCGGAAGATTCCCTACTGCTGCCTCCCGTAGGAGTCTGGACCGTGTCTCAGTTCCAGTGTGGCCGATCACCCTCTCAGGGTCGGCTACGCATCGTCGCCTTGGTAAGCCGTTACCTTACCAACTAGCTAATGCGGCGCGGATCCATCTATAAGTGACAGCAAAACCGTCTTTCCCTATTGAACCATGCGGTTCAATATATTATCCGGTATT
>n.538.6.bb_575
GGAGTTAGCCGGTGCTTCTTCTGCGGGTAACGTCAATCGACAAGGTTATTAACCTTATCGCTTTCCTCCCCGCTGAAAGTACTTTACAACCCGAAGGCCTTCTTCATACACGCGGCATGGCTGCATCAGGCTTGCGCCCATTGTGCAATATTCCCCACTGCTGCCTCCCGTAGGAGTCTGGACCGTGTCTCAGTTCCAGTGTGGCTGGTCATCCTCTCAGACCAGCTAGGGATCGTCGCCTAGGTGAGCCGTTACCCACCTACTAGCTAATCCCATCTGGGCACATCTGAT
>n.538.6.bb_576
GTAGTTAGCCGTGGCTTTCTGATTAGGTACCGTCAAGACGTGCATAGTTACTTACACATTTGTTCTTCCCTAATAACAGAGTTTTACGATCCGAAGACCTTCATCACTCACGCGGCGTTGCTCCGTCAGGCTTTCGCCCATTGCGGAAGATTCCCTACTGCTGCCTCCCGTAGGAGTCTGGACCGTGTCTCAGTTCCAGTGTGGCCGATCACCCTCTCAGGGTCGGCTACGCATCGTTGCCTTGGTAAGCCGTTACCTTACCAACTAGCTAATGCGGCGCGGATCCATCTATAAGTGACAGCAAAACCGTCTTTCACTATTGAACCATGCGGTTCAATATATTATCCGGTATTAGCTCCGGT
>n.538.6.bb_577
GCGTTAGCCGGTGCTTCTTCTGCGGGTAACGTCAATCGACAAGGTTATTAACCTTATCGCCTTCCTCCCCGCTGAAAGTACTTTACAACCCGAAGGCCTTCTTCATACACGCGGCATGGCTGCATCAGGCTTGCGCCCATTGTGCAATATTCCCCACTGCTGCCTCCGTAGGAGTCTGGACCGTGTCTCAGTTCCAGTGTGGCTGGTCATCCTCTCAGACCAGCTAGGGATCGTCGCCTAGGTGAGCCGTTACCCACCTACTAGCTAATCCCATCTGGGCACATCTGAT
>n.538.6.bb_578
GTAGTTAGCCGGTGCTTCTTCTGCAGGTACCGTCACCACAAGCTTCGCCCCTGCTGAAAGCGGTTTACAACCCGAAGGCCGTCATCCCGCACGCGGCGTTGCTGCATCAGGCTTCCGCCCATTGTGCAATATTCCCCACTGCTGCCTCCCGTAGGAGTCTGGGCCTATCTCAGTCCCAATGTGGCCGGTCGCCCTCTCGAGGCCGGCTACGCAGTCGTTGCCTTGGGTAACGCCGTTACCTACCAACTAGCTAATGCGGCGCGGATCCATCTATAAGTGA
>n.538.6.bb_579
GTAGTTAGCCGGTGCTTCTTCTGCAGGTACCGTCACCACAAGCTTCGCCCCTGCTGAAAGCGGTTTACAACCCGAAGGCCGTCATCCCGCACGCGGCGTTGCTGCATCAGGCTTCCGCCCATTGTGCAATATTCCCCACTGCTGCCTCCCGTAGGAGTCTGGGCCGTATCTCAGTCCCAATGTGGCCGGTCGCCCTCTCAGGCCGGCTACCCGGTNAAAGCCTTGGTAAGCCACTACCCACCAACAAGCTGATAAGCCGCGAGTCCATCCAAAACCGCCGAAGCTTTCCAACCCCACCATGCAGCAAGGATTCCTATCCGGTAT
>n.538.6.bb_580
GTAGTTAGCCGGTGCTTCTTCTGCAGGTACCGTCACCACAAGCTTCGCCCCTGCTGAAAGCGGTTTACAACCCGAAGGCCGTCATCCCGCACGCGGCGTTGCTGCATCAGGCTTCCGCCCATTGTGCAATATTCCCCACTGCTGCCTCCCGTAGGAGTCTGGGCCGTATCTCAGTCCCAATGTGGCCGGTCGCCCTCTCAGGCCGGCTACCCGTCAAAGGCCTTGGTAACGCCACTACCCCACCAACAAGCTGATAAGCCGCGAGTCCATCCAAAACCGC
>n.538.6.bb_581
GAAGTTAGCCGGTGCTTCTTCTGCGGGTAACGTCAATTGCTGAGGTTATTAACCTCAACACCTTCCTCCCCGCTGAAAGTACTTTACAACCCGAAGGCCTTCTTCATACACGCGGCATGGCTGCATCAGGCTTGCGCCCATTGTGCAATATTCCCCACTGCTGCCTCCCGTAGGAGTCTGGACCGTGTCTCAGTTCCAGTGTGGCTGGGTCATCCTCTCAGACCAGCTAGGGATCGTCGCCTAGGTGAGCCGTTACCCACCTACTAGCTAATCCCATCTGGGCACATCTGATGGCA
>n.538.6.bb_582
GTAGTTAGCCGTGGCTTTCTGATTAGGTACCGTCAAGACGTGCATAGTTACTTACACATTTGTTCTTCCCTAATAACAGAGTTTTACGATCCGAAGACCTTCATCACTCACGCGGCGTTGCTCCGTCAGGCTTTCGCCCATTGCGGAAGATTCCCTACTGCTGCCTCCCGTAGGAGTCTGGACCGTGTCTCAGTTCCAGTGTGGCCGATCACCCTCTCAGGGTCGGCTACGCATCGTTGCCTTGGTAAGCCGTTACCTTACCAACTAGCTAATGCGGCGCGGATCCATCTATAAGTGACAGCAAAACCGTCTTTCACTATTGAACCATGCGGTTCAATATATTATCCGGTATTA
>n.538.6.bb_583
GGAGTTAGCCGGTGCTTCTTCTGCGGGTAACGTCAATCGACAAGGTTATTAACCTTATCGCCTTCCTCCCCGCTGAAAGTACTTTACAACCCGAAGGCCTTCTTCATACACGCGGCATGGCTGCATCAGGCTTGCGCCCATTGTGCAATATTCCCCATGCTGCCTCCGTAGGAGTCTGGACCGTGTCTCAGTTCCAGTGTGGCTGGTCATCCTCTCAGACCAGCTAGGGATCGTCGCCTAGGTGAGCCGTTACCCACCTACTAGCTAATCCCATCTGGGCACATCTGATGGCAA
>n.538.6.bb_584
GGAGTTAGCCGGTGCTTCTTCTGCGGGTAACGTCAATCGATAAGGTTATTAACCTTATCGCCTTCCTCCCCGCTGAAAGTACTTTACAACCCGAAGGCCTTCTTCATACACGCGGCATGGCTGCATCAGGCTTGCGCCCATTGTGCAATACTTCCCATGCTGCCTCCGTAGGAGTCTGGACCGTGTCTCAGTTCCAGTGTGGCTGGTCATCCTCTCAGACCAGCGTAGGGATCGTCGCCTAGGTGAGCCGTTACCCACCTACTAGCTAATCCCATCTGGGCACATCTGAT
>n.538.6.bb_585
GGAGTTAGCCGGTGCTTCTTCTGCGGGTAACGTCAATCGACAAGGTTATTAACCTTATCGCCTTCCTCCCCGCTGAAAGTACTTTACAACCCGAAGGCCTTCTTCATACACGCGGCATGGCTGCATCAGGCTTGCGCCCATTGTGCAATATTCCCCATGCTGCCTCCGTAGGAGTCTGGACCGTGTCTCAGTTCCAGTGTGGCTGGTCATCCTCTCAGACCAGCTAGGGATCGTCGCCTAGGTGAGCCGTTACCCACCTACTAGCTAATCCCATCTGGGCACATCTGATGGCAAGAGGCCCGAAGGTCCCCCTCTTTGGTCTTGCGACGTTATGCGGTATTAGCTACCGTTTCCAG
>n.538.6.bb_586
GTAGTTAGCCGTGGCTTTCTGATTAGGTACCGTCAAGACGTGCATAGTTACTTACACATTTGTTCTTCCCTAATAACAGAGTTTTACGATCCGAAGACCTTCATCACTCACGCGGCGTTGCTCCGTCAGGCTTTCGCCCATTGCGGAAGATTCCCTACTGCTGCCTCCCGTAGGAGTCTGGACCGTGTCTCAGTTCCAGTGTGGCCGATCACCCTCTCAGGGTCGGCTACGCATCGTCGCCTTGGTAAGCCGTTACCTTACCAACTAGCTAATGCGGCGCGGATCCATCTATAAGTGACAGCAAAACCGTCTTTCACTATTGAACCATGCGGTTCAATATAT
>n.538.6.bb_587
GTAGTTAGCCGTGGCTTTCTGATTAGGTACCGTCAAGACGTGCATAGTTACTTACACATTTGTTCTTCCCTAATAACAGAGTTTTACGATCCGAAGACCTTCATCACTCACGCGGCGTTGCTCCGTCAGGCTTTCGCCCATTGCGGAAGATTCCCTACTGCTGCCTCCCGTAGGAGTCTGGACCGTGTCTCAGTTCCAGTGTGGCCGATCACCCTCTCAGGGTCGGCTACGCATCGTTGCCTTGGTAAGCCGTTACCTTACCAACTAGCTAATGCGGCGCGGATCCATCTATAAGTGACAGCAAAACCGTCTTTCACTATTGAACCATGCGGTTCAATATATTATCCGGTATTAGCTCCGGTTTCCCGAAGTTATCCCAGTCTTATAGGTAGGTTATCCCACGTGTTACTCACCCCGTCCCGCCCGCTAACGTCAGAGGAGCAAGCTCCCTCGTCTGTTCGCTCGACTTGCATGTATTAGGGCACGCCGCCAGCGTTCATCCT
>n.538.6.bb_588
GTAGTTAGCCGTGGCTTTCTGATTAGGTACCGTCAAGACGTGCATAGTTACTTACACATTTGTTCTTCCCTAATAACAGAGTTTTACGATCCGAAGACCTTCATCACTCACGCGGCGTTGCTCCGTCAGGCTTTCGCCCATTGCGGAAGATTCCCTACTGCTGCCTCCCGTAGGAGTCTGGACCGTGTCTCAGTTCCAGTGTGGCCGATCACCCTCTCAGGGTCGGCTACGCATCGTTCGCCTTGGTAAGCCGTTACCTTACCAACTAGCTAATGCGGCGCGGATCCATCTATAAGTGAC
>n.538.6.bb_589
GGAGTTAGCCGGTGCTTCTTCTGCGGGTAACGTCAATCGACAAGGTTATTAACCTTATCGCCTTCCTCCCCGCTGAAAGTACTTTACAACCCGAAGGCCCTCTTCATACACGCGGCATGGCTGCATCAGGCTTGCGCCCATTGTGCAATATTCCCCACTGCTGCCTCCCGTAGGAGTCTGGACCGTGTCTCAGTTCCAGTGTGGCTGGTCATCCTCTCAGACCAGCTAGGGATCGTCGCCTAGGTGAGCCGTTACCCACCTACTAGCTAATCCCATCTGGGCACATCTGAT
>n.538.6.bb_590
GTAGTTAGCCGTGGCTTTCTGATTAGGTACCGTCAAGACGTGCATAGTTACTTACACATTTNTTCTTCCCTAATAACAGAGTTTTACGATCCGAAGACCTTCATCACTCACGCGGCGTTGCTCCGTCAGGCTTTCGCCCATTCGCGGAAGATTCCCTACTGCTGCCTCCCGTAGGAGTCTGGACCGTGTCTCAGTTCCAGTGTGGCCGATCACCCTCTCAGGGTCGGCTACGCATCGTTGCCTTGGTAAGCCGTTACCTTACCAACTAGCTAATGCGGCGCGGATCCATCTATAAGTGACAGCAAAACCGTCTTTCACTATTGAACC
>n.538.6.bb_591
GTAGTTAGCCGTGGCTTTCTGATTAGGTACCGTCAAGACGTGCATAGTTACTTACACATTTATTCTTCCCTAATAACAGAGTTTTACGATCCGAAGACCTTCATCACTCACGCGGCGTTGCTCCGTCAGGCTTTCGCCCATTGCGGAAGATTCCCTACTGCTGCCTCCCGTAGGAGTCTGGACCGTGTCTCAGTTCCAGTGTGGCCGATCACCCTCTCAGGGTCGGCTACGCATCGTTGCCTTGGTAAGCCGTTACCTTACCAACTAGCTAATGCGGCGCGGATCCATCTATAAGTGACAGCAAAACCGTCTTTCACTATTGAACCATGCGGTTCAATATATTATCCGGTATTAGCTCCGGT
>n.538.6.bb_592
GTAGTTAGCCGTGGCTTTCTGATTAGGTACCGTCAAGACGTGCATAGTTACTTACACATTTGTTCTTCCCTAATAACAGAGTTTTACGATCCGAAGACCTTCATCACTCACGCGGCGTTGCTCCGTCAGGCTTCCGCCCATTGCGGAAGATTCCCTACTGCTGCCTCCCGTAGGAGTCTGGACCGTGTCTCAGTTCCAGTGTGGCCGATCACCCTCTCAGGGTCGGCTACGCATCGTTGCCTTGGTAAGCCGTTACCTTACCAACTAGCTAATGCGGCGCGGATCCATCTATAAGTGACAGCAAAACCGTCTTTCACTATTGAACCATGCGGTTCAATATATT
>n.538.6.bb_593
GTAGTTAGCCGGTGCTTCTTCTGCAGGTACCGTCACCACAAGCTTCGCCCCTGCTGAAAGCGGTTTACAACCCGAAGGCCGTCATCCCGCACGCGGCGTTGCTGCATCAGGCTTCCGCCCATTGTGCAATATTCCCCATGCTGCCTCCCGTAGGAGTCTGGGCCTATCTCAGTCCCAATGTGGCCGTCGCCCTCTCAGGCCGGCTAC
>n.538.6.bb_594
GGAGTTAGCCGGTGCTTCTTCTGCGGGTAACGTCAATCGACAAGGTTATTAACCTTATCGCCTTCCTCCCCGCTGAAAGTACTTTACAACCCGAAGGCCTTCTTCATACACGCGGCATGGCTGCATCAGGCTTGCGCCCATTGTGCAATATTCCCCACTGCTGCCTCCCGTAGGAGTCTGGACCGTGTCTCAGTTCCAGTGTGGCTGGTCATCCTCTCAGACCAGCTAGGGATCGTCGCCTAGGTGAGCCGTTACCCACCTACTAGCTAATCCCATCTGGGCACATCTGATGGCAAGAGGCCCGAAGGTCCCCCTCTTTGGTCTTGCGACGTTATGCGGTATT
>n.538.6.bb_595
GTAGTTAGCCGTGGCTTTCTGATTAGGTACCGTCAAGACATGCATAGTTACTTACACATTTGTTCTTCCCTAATAACAGAGTTTTACGATCCGAAGACCTTCATCACTCACGCGGCGTTGCTCCGTCAGGCTTTCGCCCATTGCGGAAGATTCCCTACTGCTGCCTCCCGTAGGAGTCTGGACCTGTCTCAGTTCCAGTGTGG
>n.538.6.bb_596
GAAGTTAGCCGGTGCTTCTTCTGCGGGTAACGTCAATTGCTGAGGTTATTAACCTCAACACCTTCCTCCCCGCTGAAAGTACTTTACAACCCGAAGGCCTTCTTCATACACGCGGCATGGCTGCATCAGGCTTGCGCCCATTGTGCAATATTCCCCACTGCTGCCTCCCGTAGGAGTCTGGACCGTGTCTCAGTTCCAGTGTGGCTGGGTCATCCTCTCAGACCAGCTAGGGATCGTCGCCTAGGTGAGCCGTTACCCACCTACTAGCTAATCCCATCTGGGCACATCTGATG
>n.538.6.bb_597
GTAGTTAGCCGTGGCTTTCTGATTAGGTACCGTCAAGACGTGCATAGTTACTTACACATTTATTCTTCCCTAATAACAGAGTTTTACGATCCGAAGACCTTCATCACACACGCGGCGTTGCTCCGTCAGGCTTTCGCCCATTGCGGAAGATTCCCTACTGCTGCCTCCCGTAGGAGTCTGGACCGTGTCTCAGTTCCAGTGTGGCCGATCACCCTCTCAGGGTCGGCTACGCATCGTTGCCTTGGTAAGCCGTTACCTTACCAACTAGCTAATGCGGCGCGGATCCATCTATAAGTGACAGCAAAACCGTCTTTCACTATTGAACC
>n.538.6.bb_598
GTAGTTAGCCGTGGCTTTCTGATTAGGTACCGTCAAGACGTGCATAGTTACTTACACATTTGTTCTTCCCTAATAACAGAGTTTTACGATCCGAAGACCTTCATCACTCACGCGGCGTTGCTCCGTCAGGCTTTCGCCCATTGCGGAAGATTCCCTACTGCTGCCTCCCGTAGGAGTCTGGACCGTGTCTCAGTTCCAGTGTGGCCGATCACCCTCTCAGGGTCGGCTACGCATCGTCGCCTTGG
>n.538.6.bb_599
GTAGTTAGCCGTGGCTTTCTGATTAGGTACCGTCAAGACGTGCATAGTTACTTACACATTTGTTCTTCCCTAATAACAGAGTTTTACGATCCGAAGACCTTCATCACTCACGCGGCGTTGCTCCGTCAGGCTTTCGCCCATTGCGGAAGATTCCCTACTGCTGCCTCCCGTAGGAGTCTGGACCGTGTCTCAGTTCCAGTGTGGCCGATCACCCTCTCAGGGTCGGCTACGCATCGTTGCCTTGGTAAGCCGTTACCTTACCAACTAGCTAATGCGGCGCGGATCCATCTATAAGTGACAGCAAAACCGTCTTTCACTATTGAACCATGCGGTTCAATATATTATCCGGTATT
>n.538.6.bb_600
GTAGTTAGCCGTGGCTTTCTGATTAGGTACCGTCAAGACGTGCATAGTTACTTACACATTTGTTCTTCCCTAATAACAGAGTTTTACGATCCGAAGACCTTCATCACTCACGCGGCGTTGCTCCGTCAGGCTTTCGCCCATTGCGGAAGATTCCCTACTGCTGCCTCCCGTAGGAGTCTGGACCGTGTCTCAGTTCCAGTGTGGCCGATCACCCTCTCAGGGTCGCTACGCATCGTTGCCTTGGTAAGCCGTTACCTTACAACTAGCTAATGCGGCGCGGATCCATCTATAAGTGACAGCAAAACCG
>n.538.6.bb_601
GTAGTTAGCCGGTGCTTCTTCTGCAGGTACCGTCACCACAAGCTTCGCCCCTGCTGAAAGCGGTTTACAACCCGAAGGCCGTCATCCCGCACGCGGCGTTGCTGCATCAGGCTTCCGCCCATTGTGCAATATTCCCCACTGCTGCCTCCCGTAGGAGTCTGGGCCGTATCTCAGTCCCAATGTGGCCGGTCGCCCTCTCAGGCCGGCTACCCGTCAAAGCCTTGGTAAGCCACTACCCACCAACAAGCTGATAAGCCGCGAGTCCATCCAAAACCG
>n.538.6.bb_602
GTAGTTAGCCGGTGCTTCTTCTGCAGGTACCGTCACCACAAGCTTCGCCCCTGCTGAAAGCGGTTTACAACCCGAAGGCCGTCATCCCGCACGCGGCGTTGCTGCATCAGGCTTCCGCCCATTGTGCAATATTCCCCACTGCTGCCTCCCGTAGGAGTCTGGGCCGTATCTCAGTCCCAATGTGGCCGGTCGCCCTCTCAGGCCGGCTACCCGTCAAAGCCTTGGTAAGCCACTACCCCACCAACAAGCTGATAAGCCGCGAGTCCATCCAAAACCGCCGAAGCTTTTCCAACCCCCACCATGCAGCAAGGATTCCTATCCGGTATTAGCCCCAGTTTCCTGAAGTTATCCCGAAGTCAAGGGCAGGTTACTCACGTGTTACTCACCCGTTCGCCACTCC
>n.538.6.bb_603
TAGTTAGCCGTGGCTTTCTGATTAGGTACCGTCAAGACGTGCATAGTTACTTACACATTTGTTCTTCCCTAATAACAGAGTTTTACGATCCGAAGACCTTCATCACTCACGCGGCGTTGCTCCGTCAGGCTTTCGCCCATTGCGGAAGATTCCCTACTGCTGCCTCCCGTAGGAGTCTGGACCGTGTCTCAGTTCCAGTGTGGCCGATCACCCTCTCAGGGTCGGCTACGCATCGTTGCCTTGGTAAGCCGTTACCTTACCAACTAGCTAATGCGGCGCGGATCCATCTATAAGTGACAGCAAAACCGTCTTTCACTATTGAACCATGCGGTTCAATATATTATCCGGTATT
>n.538.6.bb_604
GTAGTTAGCCGTGGCTTTCTGATTAGGTACCGTCAAGACGTGCATAGTTACTTACACATTTGTTCTTCCCTAATAACAGAGTTTTACGATCCGAAGACCTTCATCACTCACGCGGCGTTGCTCCGTCAGGCTTTCGCCCATTGCGGAAGATTCCCTACTGCTGCCTCCCGTAGGAGTCTGGACCGTGTCTCAGTTCCAGTGTGGCCGATCACCCTCTCAGGGTCGGCTACGCATCGTTGCCTTGGTAAGCCGTTACCTTACCAACTAGCTAATGCGGCGCGGATCCATCTATAAGTGACAGCAAAACCGTCTTTCACTATTGAACCATG
>n.538.6.bb_605
GTAGTTAGCCGGTGCTTCTTCTGCAGGTACCGTCACCACAAGCTTCGCCCCTGCTGAAAGCGGTTTACAACCCGAAGGCCGTCATCCCGCACGCGGCGTTGCTGCATCAGGCTTCCGCCCATTGTGCAATATTCCCCACTGCTGCCTCCCGTAGGAGTCTGGGCCGTATCTCAGTCCCAATGTGGCCGGTCGCCCTCTCAGGCCGGCTACCCGGTCAAAGCCTTGGTAAGCCACTACCCCACCAACAAGCTGATAAGCCGCGAGTCCATCCAAAACCGC
>n.538.6.bb_606
GGAGTTAGCCGGTGCTTCTTCTGCGGGTAACGTCAATCGACAGGGTTATTAACCCTGTCGCCTTCCTCCCCGCTGAAAGTACTTTACAACCCGAAGGCCTTCTTCATACACGCGGCATGGCTGCATCAGGCTTGCGCCCATTGTGCAATATTCCCCACTGCTGCCTCCGTAGGAGTCTGGACCGTGTCTCAGTTCCAGTGTGGCTGGTCATC
>n.538.6.bb_607
GTAGTTAGCCGTGGCTTTCTGATTAGGTACCGTCAAGACGTGCATAGTTACTTACACATTTGTTCTTCCCTAATAACAGAGTTTTACGATCCGAAGACCTTCATCACTCACGCGGCGTTGCTCCGTCAGGCTTTCGCCCATTGCGGAAGATTCCCTACTGCTGCCTCCCGTAGGAGTCTGGACCGTGTCTCAGTTCCAGTGTGGCCGATCACCCTCTCAGGGTCGGCTACGCATCGTTCGCCTTGGTAAGCCGTTACCTTACAACTAGCTAATGCGGCGCGGATCCATCTATAAGTGACAGC
>n.538.6.bb_608
GTAGTTAGCCGTGGCTTTCTGATTAGGTACCGTCAAGACGTGCATAGTTACTTACACATTTGTTCTTCCCTAATAACAGAGTTTTACGATCCGAAGACCTTCATCACTCACGCGGCGTTGCTCCGTCAGGCTTTCGCCCATTGCGGAAGATTCCCTACTGCTGCCTCCCGTAGGAGTCTGGACCGTGTCTCAGTTCCAGTGTGGCCGATCACCCTCTCAGGGTCGGCTACGCATCGTTGCCTTGGTAAGCCGTTACCTTACCAACTAGCTAATGCGGCGCGGATCCATCTATAAGTGACAGCAAAACCGTCTTTCACTATTGAACCATGCGGTTCAATATATTATCCGGTATTAGCTCCGGTTT
>n.538.6.bb_609
GGAGTTAGCCGGTGCTTCTTCTGCGGGTAACGTCAATCGACAAGGTTATTAACCTTATCGCCTTCCTCCCCGCTGAAAGTACTTTACAACCCGAAGGCCTTCTTCATACACGCGGCATGGCTGCATCAGGCTTGCGCCCATTGTGCAATATTCCCCATGCTGCCTCCCGTAGGAGTCTGGACCGTGTCTCAGTTCCAGTGTGGCTGGTCATCCTCTCAGACCAGCTAGGGATCGTCGCCTAGGTGAGCCGTTACCCACCTACTAGCTAATCCCATCTGGGCACATCTGATGGC
>n.538.6.bb_610
GTAGTTGGCCGTGGCTTTCTGATTAGGTACCGTCAAGACGTGCATAGTTACTTACACATTTGTTCTTCCCTAATAACAGAGTTTTACGATCCGAAGACCTTCATCACTCACGCGGCGTTGCTCCGTCAGGCTTTCGCCCATTGCGGAAGATTCCCTACTGCTGCCTCCCGTAGGAGTCTGGACCGTGTCTCAGTTCCAGTGTGGCCGATCACCCTCTCAGGGTCGGCTACGCATCGTCGCCTTGGTAAGCCGTTACCTTACCAACTAGCTAATGCGGCGCGGATCCATCTATAAGTGACAGCAAAACCGTCTTTCACTA
>n.538.6.bb_611
GTAGTTAGCCGTGGCTTTCTGATTAGGTACCGTCAAGACGTGCATAGTTACTTACACATTTGTTCTTCCCTAATAACAGAGTTTTACGATCCGAAGACCTTCATCACTCACGCGGCGTTGCTCCGTCAGGCTTTCGCCCATTGCGGAAGATTCCCTACTGCTGCCTCCCGTAGGAGTCTGGACCGTGTCTCAGTTCCAGTGTGGCCGATCACCCTCTCAGGGTCGGCTACGCATCGTTGCCTTGGTAAGCCGTTACCTTACCAACTAGCTAATGCGGCGCGGATCCATCTATAAGTGACAGCAAAACCGTCTTTCACTATTGAACCATGCGGTTCAATATATTA
>n.538.6.bb_612
GTAGTTAGCCGGTGCTTCTTCTGCAGGTACCGTCACCACAAGCTTCGCCCCTGCTGAAAGCGGTTTACAACCCGAAGGCCGTCATCCCGCACGCGGCGTTGCTGCATCAGGCTTCCGCCCATTGTGCAATATTCCCCACTGCTGCCTCCCGTAGGAGTCTGGGCCGTATCTCAGTCCCAATGTGGCCGGTCGCCCTCTCAGGCCGGCTACCCGTCAAAGCCTTGGTAAGCCACTACCCACCAACAAGCTGATAAGCCGCGAGTCCATCCAAAACCGCCGAAGCTTTCCAACCCCCACCATGCAGCAAGGATTCCTATCCGGTATTAGCCCCAGTTTCCTGAAGTTATCCCGAAGTCAAGGGCAGGTTACTCACG
>n.538.6.bb_613
GTAGTTAGCCGTGGCTTTCTGATTAGGTACCGCCAAGACGTGCATAGTTACTTACACATTTGTTCTTCCCTAATAACAGAGTTTTACGATCCGAAGACCTTCATCACTCACGCGGCGTTGCTCCGTCAGGCTTTCGCCCATTGCGGAAGATTCCCTACTGCTGCCTCCCGTAGGAGTCTGGACCGTATCTCAGTTCCAGTGTGGCCGATCACCCTCTCAGGGTCGGCTACGCATCGTTGCCTTGGTAAGCCGTTACCTTACCAACTAGCTAATGCGGCGCGGATCCATCTATAAGTGACAGCAAAACCGTCTTTCACTATTGAACCATGCGGTTCAATATATTATCCGGTATTAGCTCCGGT
>n.538.6.bb_614
GTAGTTAGCCGGTGCTTCTTCTGCAGGTACCGTCACCACAAGCTTCGCCCCTGCTGAAAGCGGTTTACAACCCGAAGGCCGTCATCCCGCACGCGGCGTTGCTGCATCAGGCTTCCGCCCATTGTGCAATATTCCCCACTGCTGCCTCCCGTAGGAGTCTGGGCCGTATCTCAGTCCCAATGTGGCCGGTCGCCCTCTCGAGGCCGGCTACCCGGTCAAAGCCTTGGTAACGCCACTACCCACCAACAAGCTGATAAGCCGCGAGTCC
>n.538.6.bb_615
GTAGTTAGCCGGTGCTTCTTCTGCAGGTACCGTCACCACAAGCTTCGCCCCTGCTGAAAGCGGTTTACAACCCGAAGGCCGTCATCCCGCACGCGGCGTTGCTGCATCAGGCTTCCGCCCATTGTGCAATATTCCCCACTGCTGCCTCCCGTAGGAGTCTGGGCCGTATCTCAGTCCCAATGTGGCCGGTCGCCCTCTCAGGCCGGCTACCCGGTCAAAGCCTTGGTAAGCCACTACCCCACCAACAAGCTGATAAGCCGCGAGTCCATCCAAAAACCGCCGAAGCTTTTCCAACCCCCACCATGCAGCAAGGATTCCTATCCGGTATTAGCCCCAGTTTCCTGAAGTTATCCCGAA
>n.538.6.bb_616
GTAGTTAGCCGGTGCTTCTTCTGCAGGTACCGTCACCACAAGCTTCGCCCCTGCTGAAAGCGGTTTACAACCCGAAGGCCGTCATCCCGCACGCGGCGTTGCTGCATCAGGCTTCCGCCCATTGTGCAATATTCCCCATGCTGCCTCCCGTAGGAGTCTGGGCCGTATCTCAGTCCCAATGTGGCCGGTCGCCCTCTCAGGCCGGCTACCCGTCAAAGCCTTGGTAAGCCACTACCCACCAACAAGCTGATAAGCCGCGAGTCC
>n.538.6.bb_617
GGAGTTAGCCGGTGCTTCTTCTGCGGGTAACGTCAATTGCTGAGGTTATTAACCTCAACACCTTCCTCCCCGCTGAAAGTACTTTACAACCCGAAGGCCTTCTTCATACACGCGGCATGGCTGCATCAGGCTTGCGCCCATTGTGCAATATTCCCCACTGCTGCCTCCCGTAGGAGTCTGGACCGTGTCTCAGTTCCAGTGTGGCTGGGTCATCCTCTCAGACCAGCTAGGGATCGTCGCCTAGGTGAGCCGTTACCCACCTACTAGCTAATCCCATCTGGGCACATCTGATGGCAA
>n.538.6.bb_618
GTAGTTAGCCGTGGCTTTCTGATTAGGTACCGTCAAGACGTGCATAGTTACTTACACATTTATTCTTCCCTAATAACAGAGTTTTACGATCCGAAGACCTTCATCACTCACGCGGCGTTGCTCCGTCAGGCTTTCGCCCATTGCGGAAGATTCCCTACTGCTGCCTCCCGTAGGAGTCTGGACCGTGTCTCAGTTCCAGTGTGGCCGATCACCCTCTCAGGGTCGGCTACGCATCGTTGCCTTGGTAAGCCGTTACCTTACCAACTAGCTAATGCGGCGCGGATCCATCTATAAGTGACAGCAAAACCGTCTTTCACTATTGAACCATGCGGTTCAATATATT
>n.538.6.bb_619
GTAGTTAGCCGGTGCTTCTTCTGCAGGTACCGTCACCACAAGCTTCGCCCCTGCTGAAAGCGGTTTACAACCCGAAGGCCGTCATCCCGCACGCGGCGTTGCTGCATCAGGCTTCCGCCCATTGTGCAATATTCCCCACTGCTGCCTCCCGTAGGAGTCTGGGCCGTATCTCAGTCCCAATGTGGCCGGTCGCCCTCTCAGGCCGGCTACCCGTCAAAGCCTTGGTAGCCACTACCCCACCAACAAGCTGATAAGCCGCGAGTCCATCCAAAACCGCCGA
>n.538.6.bb_620
GTAGTTAGCCGTGGCTTTCTGATTAGGTACCGTCAAGACGTGCATAGTTACTTACACATTTGTTCTTCCCTAATAACAGAGTTTTACGATCCGAAGACCTTCATCACTCACGCGGCGTTGCTCCGTCAGGCTTTCGCCCATTGCGGAAGATTCCCTACTGCTGCCTCCCGTAGGAGTCTGGACCGTGTCTCAGTTCCAGTGTGGCCGATCACCCTNTCAGGGTCGGCTACGCATCGTTGCCTTGGTAAGCCGTTACCTTACCAACTAGCTAATGCGGCGCGGATCCATCTATAAGTGACAGCAAAACCGTCTTTCACTATTGAACCATGCGGTTCAATATATTATCCGGTATT
>n.538.6.bb_621
GTAGTTAGCCGGTGCTTCTTCTGCAGGTACCGTCACCACAAGCTTCGCCCCTGCTGAAAGCGGTTTACAACCCGAAGGCCGTCATCCCGCACGCGGCGTTGCTGCATCAGGCTTCCGCCCATTGTGCAATATTCCCCACTGCTGCCTCCCGTAGGAGTCTGGGCCGTATCTCAGTCCCAATGTGGCCGGTCGCCCTCTCAGGCCGGCTACCCGGTCAAAGCCTTGGTAAGCCACTACCCACCAACAAGCTGATAAGCCGCGAGTCCATCCAAAACCGCCGA
>n.538.6.bb_622
GTAGTTAGCCGGTGCTTCTTCTGCAGGTACCGTCACCACAAGCTTCGCCCCTGCTGAAAGCGGTTTACAACCCGAAGGCCGTCATCCCGCACGCGGCGTTGCTGCATCAGGCTTCCGCCCATTGTGCAATATTCCCCACTGCTGCCTCCCGTAGGAGTCTGGGCCGTATCTCAGTCCCAATGTGGCCGGTCGCCCTCTCAGGCCGGCTACCCGTCAAAGCCTTGGTAAGCCACTACCCACCAACAAGCTGATAAGCCGCGAGTCCATCCAAAACCGCCGAAGCTTTTCCAACCCCACCATGCAGCAAGGATTCCTATCCGGTATTAGCCCCAGTTTCCTG
>n.538.6.bb_623
GTAGTTAGCCGTGGCTTTCTGATTAGGTACCGTCAAGACGTGCATAGTTACTTACACATTTGTTCTTCCCTAATAACAGAGTTTTACGATCCGAAGACCTTCATCACTCACGCGGCGTTGCTCCGTCAAGCTTTCGCCCATTGCGGAAGATTCCCTACTGCTGCCTCCCGTAGGAGTCTGGACCGTGTCTCAGTTCCAGTGTGGCCGATCACCCTCTCAGGGTCGGCTACGCATCGTTGCCTTGGTAAGCCGTTACCTTACCAACTAGCTAATGCGGCGCGGATCCATCTATAAGTGACAGCAAAACCGTCTTTCACTATTGAACCATGCGGTTCAATATATTATCCGGTATT
>n.538.6.bb_624
GTAGTTAGCCGGTGCTTCTTTACCCATTACCGTCACTCACGCTTCGTCACAGGCGAAAGCGGTTTACAACCCGAAGGCCGTCATCCCGCACGCGGCGTTGCTGCATCAGGCTTCCGCCCATTGTGCAATATTCCCCACTGCTGCCTCCCGTAGGAGTCTGGGCCGTATCTCAGTCCCAATGTGGCCGGTCACCCTCTCAGGCCGGCTACCCGTCAAAGCCTTGGGTAAGCCCACTACCCCACAACAAGCTGATAAGCCGCGAGTCCATCCCAACCGCCGAAACTTTCCAACCCCACCATGCAGCAGGAGCTCCTATCCGGTAT
>n.538.6.bb_625
GGAGTTAGCCGGTGCTTCTTCTGCGGGTAACGTCAATTGCTGAGGTTATTAACCTCAACACCTTCCTCCCCGCTGAAAGTACTTTACAACCCGAAGGCCTTCTTCATACACGCGGCATGGCTGCATCAGGCTTGCGCCCATTGTGCAATATTCCCCACTGCTGCCTCCCGTAGGAGTCTGGACCGTGTCTCAGTTCCAGTGTGGCTGGGTCATCCTCTCAGACCAGCTAGGGATCGTCGCCTAGGTGAGCCGTTACCCCACCTACTAGCTAATCCCATCTGGGCACATCTGATGGC
>n.538.6.bb_626
GTAGTTAGCCGTGGCTTTCTGATTAGGTACCGTCAAGACGTGCATAGTTACTTACACATTTGTTCTTCCCTAATAACAGAGTTTTACGATCCGAAGACCTTCATCACTCACGCGGCGTTGCTCCGTCAAGCTTTCGCCCATTGCGGAAGATTCCCTACTGCTGCCTCCCGTAGGAGTCTGGACCGTGTCTCAGTTCCAGTGTGGCCGATCACCCTCTCAGGGTCGGCTACGCATCGTTGCCTTGGTAAGCCGTTACCTTACCAACTAGCTAATGCGGCGCGGATCCATCTATAAGTGACAGCAAAACCGTCTTTCACTATTGAACCATGCGG
>n.538.6.bb_627
GGAGTTAGCCGGTGCTTCTTCTGCGGGTAACGTCAATTGCTGAGGTTATTAACCTCAACACCTTCCTCCCCGCTGAAAGTACTTTACAACCCGAAGGCCTTCTTCATACACGCGGCATGGCTGCATCAGGCTTGCGCCCATTGTGCAATATTCCCCACTGCTGCCTCCCGTAGGAGTCTGGACCGTGTCTCTGTTCCAGTGTGGCTGGGTCATCCTCTCAGACCAGCTAGGGATCGTCGCCTAGGTGAGCCGTTACCCCACCTACTAGCTAATCCCATCTGGGCACATCTGATGGCAA
>n.538.6.bb_628
GTAGTTAGCCGTGGCTTTCTGATTAGGTACCGTCAAGACGTGCATAGTTACTTACACATTTGTTCTTCCCTAATAACAGAGTTTTACGATCCGAAGACCTTCATCACTCACGCGGCGTTGCTCCGTCAGGCTTTCGCCCATTGCGGAAGATTCCCTACTGCTGCCTCCCGTAGGAGTCTGGACCGTGTCTCAGTTCCAGTGTGGCCGATCACCCTCTCAGGGTCGGCTACGCATCGTCGCCTTGGTAAGCCGTTACCTTACCAACTAGCTAATGCGGCGCGGATCCATCTATAAGTGACAGCAAAACCGTCTTTCACTATTGAACCATGCGGTTCAATATATTATCCCCGTATTAGTTCCGGTTTCCCCGAAGTTATCCCAGTCTTATAGGTAGGTTATCCACCGTGTTACTCACCCCGTCCCGCCCGCTAACGTCAGAGAAGCAAGCTCCCTCGTCTGTTCCGCTCGAC
>n.538.6.bb_629
GGAGTTAGCCGGTGCTTCTTCTGCGGGTAACGTCAATCGACAAGGTTATTAACCTTATCGCCTTCCTCCCCGCTGAAAGTACTTTACAACCCGAAGGCCTTCTTCATACACGCGGCATGGCTGCATCAGGCTTGCGCCCATTGTGCAATATTCCCCACTGCTGCCTCCCGTAGGAGTCTGGACCGTGTCTCAGTTCCAGTGTGGCTGGTCATCCTCTCAGACCAGCTAGGGATCGTCGCCTAGGTGAGCCGTTACCCACCTACTAGCTAATCCCATCTGGGCACATCTGATGGCAAGAGG
>n.538.6.bb_630
GTAGTTAGCCGTGGCTTTCTGATTAGGTACCGTCAAGACGTGCATAGTTACTTACACATTTGTTCTTCCCTAATAACAGAGTTTTACGATCCGAAGACCTTCATCACTCACGCGGCGTTGCTCCGTCAGGCTTTCGCCCATTGCGGAAGATTCCCTACTGCTGCCTCCCGTAGGAGTCTGGACCGTGTCTCAGTTCCAGTGTGGCCGATCACCCTCTCAGGGTCGGCTACGCATCGTTGCCTTGGTAAGCCGTTACCTTACAACTAGCTAATGCGGCGCGGATCCATCTATAAGTGACAGCAAAACCGTCTTTCACTATTGAACCATGCGGTCCAAT
>n.538.6.bb_631
GTAGTTAGCCGTGGCTTTCTGATTAGGTACCGTCAAGACGTGCATAGTTACTTACACATTTATTCTTCCCTAATAACAGAGTTTTACGATCCGAAGACCTTCATCACTCACGCGGCGTTGCTCCGTCAGGCTTTCGCCCATTGCGGAAGATTCCCTACTGCTGCCTCCCGTAGGAGTCTGGACCGTGTCTCAGTTCCAGTGTGGCCGATCCACCCTCTCAGGGTCGGCTACGCATCGTTGCCTTGGTAAGCCGTTACCTTACCAACTAGCTAATGCGGCGCGGATCCATCTATAAGTGACA
>n.538.6.bb_632
GTAGTTAGCCGTGGCTTTCTGATTGGGTACCGTCAAGACGTGCATAGTTACTTACACATTTGTTCTTCCCTAATAACAGAGTTTTACGATCCGAAGACCTTCATCACTCACGCGGCGTTGCTCCGTCAGGCTTTCGCCCATTGCGGAAGATTCCCTACTGCTGCCTCCCGTAGGAGTCTGGACCGTGTCTCAGTTCCAGTGTGGCCGATCACCCTCTCAGGGTCGGCTACGCATCGTTGCCTTGGTAAGCCGTTACCTTACCAACTAGCTAATGCGGCGCGGATCCATCTATAAGTGACAGCAAAACCGTCTTTCACTATTGAACCATGCGGTTCAATATATTATCCGGTATT
>n.538.6.bb_633
GGAGTTAGCCGGTGCTTCTTCTGCGGGTAACGTCAATCGACAAGGTTATTAACCTTATCGCCTTCCTCCCCGCTGAAAGTACTTTACAACCCGAAGGCCTTCTTCATACACGCGGCATGGCTGCATCAGGCTTGCGCCCATTGTGCAATATTCCCCACTGCTGCCTCCGTAGGAGTCTGGACCGTGTCTCAGTTCCAGTGTGGCTGGTCATCCTCTCAGACCAGCTAGGGATCGTCGCCTAGGTGAGCCGTTACCCACTACTAGCTAATCCCATCTGGGCACATCCGAT
>n.538.6.bb_634
GAAGTTAGCCGGTGCTTCTTCTGCGGGTAACGTCAATCGACAGGGTTATTAACCCTGTCGCCTTCCTCCCCGCTGAAAGTACTTTACAACCCGAAGGCCTTCTTCATACACGCGGCATGGCTGCATCAGGCTTGCGCCCATTGTGCAATATTCCCCACTGCTGCCTCCCGTAGGAGTCTGGACCGTGTCTCAGTTCCAGTGTGGCTGGTCATCCTCTCAGACCAGCTAGGGATCGTCGCCTAGGTGAGCCGTTACCCACCTACTAGCTAATCCCATCTGGGC
>n.538.6.bb_635
GTAGTTAGCCGTGGCTTTCTGATTAGGTACCGTCAAGACGTGCATAGTTACTTACACATTTATTCTTCCCTAATAACAGAGTTTTACGATCCGAAGACCTTCATCACTCACGCGGCGTTGCTCCGTCAGGCTTTCGCCCATTGCGGAAGATTCCCTACTGCTGCCTCCCGTAGGAGTCTGGACCGTGTCTCAGTTCCAGTGTGGCCGATCACCCTCTCAGGGTCGGCTACGCATCGTTGCCTTGGTAAGCCGTTACCTTACCAACTAGCTAATGCGGCGCGGATCCATCTATAAGTGACAGCAAAACCGTCTTTCACTATTGAACCATGCGGTTCAATATATTA
>n.538.6.bb_636
GTAGTTAGCCGTGGCTTTCTGATTAGGTACCGTCAAGACGTGCATAGTTACTTACACATTTGTTCTTCCCTAATAACAGAGTTTTACGATCCGAAGACCTTCATCACTCACGCGGCGTTGCTCCGTCAGGCTTTCGCCCATTGCGGAAGATTCCCTACTGCTGCCTCCCGTAGGAGTCTGGACCGTGTCTCAGTTCCAGTGTGGCCGATCACCCTCTCAGGGTCGGCTACGCATCGTCGCCTTGGTAAGCCGTTACCTTACCAACTAGCTAATGCGGCGCGGATCCATCTATAAGTGACAGCAAAACCGTCTTTCACTA
>n.538.6.bb_637
GTAGTTAGCCGGTGCTTCTTCTGCAGGTACCGTCACCACAAGCTTCGCCCCTGCTGAAAGCGGTTTACAACCCGAAGGCCGTCATCCCGCACGCGGCGTTGCTGCATCAGGCTTCCGCCCATTGTGCAATATTCCCCACTGCTGCCTCCCGTAGGAGTCTGGGCCGTATCTCAGTCCCAATGTGGCCGGTCGCCCTCTCAGGCCGGCTACCCGTCAAAGCCTTGGTAAGCCACTACCCCACCAACAAGCTGATAAGCCGCGAGTCCATCCAAAACCGCCGAAGCTTTCCAACCCCCACCATGCAGCAAGGATTCCTATCCGGTATTAGCCCCAG
>n.538.6.bb_638
GTAGTTAGCCGTGGCTTTCTGATTAGGTACCGTCAAGACGCGCATAGTTACTTACACATTTGTTCTTCCCTAATAACAGAGTTTTACGATCCGAAGACCTTCATCACTCACGCGGCGTTGCTCCGTCAGGCTTTCGCCCATTGCGGAAGATTCCCTACTGCTGCCTCCCGTAGGAGTCTGGACCGTGTCTCAGTTCCAGTGTGGCCGATCACCCTCTCAGGGTCGGCTACGCATCGTCGCCTTGGTAAGCCGTTACCTTACCAACTAGCTAATGCGGCGCGGATCCATCTATAAGTGAC
>n.538.6.bb_639
GTAGTTAGCCGTGGCTTTCTGATTAGGTACCGTCAAGACGTGCATAGTTACTTACACATTTGTTCTTCCCTAATAACAGAGTTTTACGATCCGAAGACCTTCATCACTCACGCGGCGTTGCTCCGTCAGGCTTTCGCCCATTGCGGAAGATTCCCTACTGCTGCCTCCCGTAGGAGTCTGGACCGTGTCTCAGTTCCAGTGTGGCCGATCACCCTNTCAGGGTCGGCTACGCATCGTTGCCTTGGTAAGCCGTTACCTTACAACTAGCTAATGC
>n.538.6.bb_640
GTAGTTAGCCGTGGCTTTCTGATTAGGTACCGTCAAGACGTGCATAGTTACTTACACATTTGTTCTTCCCTAATAACAGAGTTTTACGATCCGAAGACCTTCATCACTCACGCGGCGTTGCTCCGTCAGGCTTTCGCCCATTGCGGAAGATTCCCTACTGCTGCCTCCCGTAGGAGTCTGGACCGTGTCTCAGTTCCAGTGTGGCCGATCACCCTCTCAGGGTCGGCTACGCATCGTTGCCTTGGTAAGCCGTTACTTACCAACTAGCTAATGCGGCGCGGATCCATCTATAAGTGACAGCAAAACCGTCTTTCACTATTGAACCATGCGGTTC
>n.538.6.bb_641
GTAGTTAGCCGGTGCTTCTTCTGCAGGTACCGTCACCACAAGCTTCGCCCCTGCTGAAAGCGGTTTACAACCCGAAGGCCGTCATCCCGCACGCGGCGTTGCTGCATCAGGCTTCCGCCCATTGTGCAATATTCCCCACTGCTGCCTCCCGTAGGAGTCTGGGCCGTATCTCAGTCCCAATGTGGCCGGTCGCCCTCTCAGGCCGGCTACCCGGTCAAAGCCTTGGTAAGCCACTACCCCACCAACAAGCTGATAAGCCGCGAGTCCATCCAAAACCGCC
>n.538.6.bb_642
GGAGTTAGCCGGCGCTTCTTCTGCGGGTAACGTCAATCGACAAGGTTATTAACCTTATCGCCTTCCTCCCCGCTGAAAGTACTTTACAACCCGAAGGCCTTCTTCATACACGCGGCATGGCTGCATCAGGCTTGCGCCCATTGTGCAATATTCCCCACTGCTGCCTCCCGTAGGGGTCTGGACCGTGTCTCAGTTCCAGTGTGGCTGGTCATCCTCTCAGACCAGCTAGGGATCGTCGCCTAGGTGAGCCGTTACCCACCTACTAGCTAATCCCATCTGGGCACATCTGAT
>n.538.6.bb_643
GGAGTTAGCCGTGGCTTTCTGATTAGGTACCGTCAAGACGTGCATAGTTACTTACACATTTGTTCTTCCCTAATAACAGAGTTTTACGATCCGAAGACCTTCATCACTCACGCGGCGTTGCTCCGTCAGGCTTTCGCCCATTGCGGAAGATTCCCTACTGCTGCCTCCCGTAGGAGTCTGGACCGTGTCTCAGTTCCAGTGTGGCCGATCACCCTCTCAGGGTCGGCTACGCATCGTCGCCTTGGTAAGCCGTTACCTTACCAACTAGCTAATGCGGCGCGGATCCATCTATAAGTGACAGCAAAACCGTCTTTCACTATTGAACCATGCGGTTCAATATATTATCCGGTATTAGCTCCGGT
>n.538.6.bb_644
GTAGTTAGCCGTGGCTTTCTGATTAGGTACCGTCAAGACGTGCATAGTTACTTACACATTTGTTCTTCCCTAATAACAGAGTTTTACGATCCGAAGACCTTCATCACTCACGCGGCGTTGCTCCGTCAGGCTTTCGCCCATTGCGGAAGATTCCCTACTGCTGCCTCCCGTAGGAGTCTGGACCGTGTCTCAGTTCCAGTGTGGCCGATCACCCTCTCAGGGTCGGCTACGCATCGCTGCCTTGGTAAGCCGTTACCTTACAACTAGCTAATGCGGCGCGGATCCATCTATAAGTGAC
>n.538.6.bb_645
GTAGTTAGCCGTGGCTTTCTGATTAGGTACCGTCAAGACGTGCATAGTTACTTACACATTTGTTCTTCCCTAATAACAGAGTTTTACGATCCGAAGACCTTCATCACTCACGCGGCGTTGCTCCGTCAGGCTTTCGCCCATTGCGGAAGATTCCCTACTGCTGCCTCCCGTAGGAGTCTGGACCGTGTCTCAGTTCCAGTGTGGCCGATCACCCTCTCAGGGTCGGCTACGCATCGTTGCCTTGGTAAGCCGTTACCTTACCAACTAGCTAATGCGGCGCGGATCCGTCTATAAGTGACAGCAAAACCGTCTTTCACTATTGAACCATGCGGTTCAATATATTAT
>n.538.6.bb_646
GTAGTTAGCCGTGGCTTTCTGATTAGGTACCGTCAAGACGTGCATAGTTACTTACACATTTGTTCTTCCCCTAATAACAGAGTTTTACGATCCGAAGACCTTCATCACTCACGCGGCGTTGCTCCGTCAGGCTTTCGCCCATTGCGGAAGATTCCCTACTGCTGCCTCCCGTAGGAGTCTGGACCGTGTCTCAGTTCCAGTGTGGCCGATCACCCTCTCAGGGTCGGCTACGCATCGTGCCTTGGTAAGCCGTTACCTTACCAACTAGCTAATGCGGCGCGGATCCATCTATAAGTGACAGCAAAACCGTCTTTCACTATTGAACCATGCGGTTCAATATATTATCCGGTATTAGCTCCGGT
>n.538.6.bb_647
GTAGTTAGCCGTGGCTTTCTGATTAGGTACCGTCAAGACGTGCATAGTTACTTACACATTTGTTCTTCCCTAATAACAGAGTTTTACGATCCGAAGACCTTCATCACTCACGCGGCGTTGCTCCGTCAGGCTTTCGCCCATTGCGGAAGATTCCCTACTGCTGCCTCCCGTAGGAGTCTGGACCGTGTCTCAGTTCCAGTGTGGCCGATCACCCTCTCAGGGTCGGCTACGCATCGTTGCCTTGGTAAGCCGTTACCTTACCAACTAGCTAATGCGGGGCGATCCATCTATAAGTGACAGCAAAACCGTCTTTCACTATTGAACCATGCGGTTCAATATA
>n.538.6.bb_648
GTAGTTAGCCGTGGCTTTCTGATTAGGTACCGTCAAGACGTGCATAGTTACTTACACATTTGTTCTTCCCTAATAACAGAGTTTTACGATCCGAAGACCTTCATCACTCACGCGGCGTTGCTCCGTCAGGCTTTCGCCCATTGCGGAAGATTCCCTACTGCTGCCTCCCGTAGGAGTCTGGACCGTGTCTCAGTTCCAGTGTGGCCGATCACCCTCTCAGGGTCGGCTACGCATCGTTGCCTTGGTAAGCCGTTACCTTACCAACTAGCTAATGCGGCGCGGATCCATCTATAAGTGACAGCAAAACCGTCTTTCACTATTGAACCATGCGGTTCAATATATTATCCGGTATT
>n.538.6.bb_649
GTAGTTAGCCGTGGCTTTCTGATTAGGTACCGTCAAGACGTGCATAGTTACTTACACATTTGTTCTTCCCTAATAACAGAGTTTTACGATCCGAAGACCTTCATCACTCACGCGGCGTTGCTCCGTCAGGCTTTCGCCCATTGCGGAAGATTCCCTACTGCTGCCTCCCGTAGGAGTCTGGACCGTGTCTCAGTTCCAGTGTGGCCGATCACCCTCTCAGGGTCGGCTACGCATCGTTGCCTTGGTAAGCCGTTACCTTACCAACTAGCTAATGCGGCGCGGATCCATCTATAAGTGACAGCAAAACCGTCTTTCACTA
>n.538.6.bb_650
TAGTTAGCCGGTGCTTCTTCTGCAGGTACCGTCACCACAAGCTTCGCCCCTGCTGAAAGCGGTTTACAACCCGAAGGCCGTCATCCCGCACGCGGCGTTGCTGCATCAGGCTTCCGCCCATTGTGCAATATTCCCCACTGCTGCCTCCCGTAGGAGTCTGGGCCGTATCTCAGTCCCAATGTGGCCGGTCGCCCTCTCAGGCCGGCTACCCGGTCAAAGCCTTGGTAAGCCACTACCCCACCAACAAGCTGATAAGCCGCGAGTCCATCC
>n.538.6.bb_651
GTAGTTAGCCGTGGCTTTCTGATTAGGTACCGTCAAGACGTGCATAGTTACTTACACATTTGTTCTTCCCTAATAACAGAGTTTTACGATCCGAAGACCTTCATCACTCACGCGGCGTTGCTCCGTCAGGCTTTCGCCCATTGCGGAAGATTCCCTACTGCTGCCTCCCGTAGGAGTCTGGACCGTGTCTCAGTTCCAGTGTGGCCGATCACCCTCTCAGGGTCGGCTACGCATCGTTGCCTTGGTAAGCCGTTACCTTACCAACTAGCTAATGCGGCGCGGATCCATCTATAAGTGACAGCAAAACCGTCTTTCACTATTGAACCATGCGGTTCAATATATTATCCGGTATT
>n.538.6.bb_652
GGAGTTAGCCGGTGCTTCTTCTGCGGGTAACGTCAATCGATGAGGTTATTAACCTCACCGCCTTCCTCCCCGCTGAAAGTGCTTTACAACCCGAAGGCCTTCTTCACACACGCGGCATGGCCGCATCAGGCTTGCGCCCATTGTGCAATATTCCCCACTGCTGCCTCCCGTAGGAGTCTGGACCGTGTCTCAGTTCCAGTGTGGCTGGGTCATCCTCTCAGACCAGCTAGGGATCGTCGCCTAGGTGAGTCGTTACCCACCTACCAGCT
>n.538.6.bb_653
GTAGTTAACCGGTGCTTCTTCTGCAGGTACCGTCACCACAAGCTTCGCCCCTGCTGAAAGCGGTTTACAACCCGAAGGCCGTCATCCCGCACGCGGCGTTGCTGCATCAGGCTTCCGCCCATTGTGCAATATTCCCCATGCTGCCTCCCGTAGGAGTCTGGGCCGTATCTCAGTCCCAATGTGGCCGGTCGCCCTCTCAGGCCGGCTACCCG
>n.538.6.bb_654
GTAGTTAGCCGTGGCTTTCTGATTAGGTACCGTCAAGACGTGCATAGTTACTTACACATTTGTTCTTCCCTAATAACAGAGTTTTACGATCCGAAGACCTTCATCACTCACGCGGCGTTGCTCCGTCAGGCTTTCGCCCATTGCGGAAGATTCCCTACTGCTGCCTCCCGTAGGAGTCTGGACCGTGTCTCAGTTCCAGTGTGGCCGATCACCCTCTCAGGGTCGGCTACGCATCGTTGCCTTGGTAAGCCGTTACCTTACCAACTAGCTAATGCGGCGCGGATCCATCTATAAGTGACAGCAAAACCGTCTTTCACTATTGAACCATGCGGTTCAATATATTATCCGGTATTAG
>n.538.6.bb_655
GTAGTTAGCCGTGGCTTTCTGATTAGGTACCGTCAAGACGTGCATAGTTACTTACACATTTGTTCTTCCCTAATAACAGAGTTTTACGATCCGAAGACCTTCATCACTCACGCGGCGTTGCTCCGTCAGGCTTTCGCCCATTGCGGAAGATTCCCTACTGCTGCCTCCCGTAGGAGTCTAGACCGTGTCTCAGTTCCAGTGTGGCCGATCACCCTCTCAGGGTCGGCTACGCATCGTTGCCTTGGTAAGCCGTTACCTTACCAACTAGCTAATGCGGCGCGGATCCATCTATAAGTGACAGCAAAACCGTCTTTCACTATTGAACCATGCGGTTCAATATATTAT
>n.538.6.bb_656
GTAGTTAGCCGGTGCTTCTTCTGCAGGTACCGTCACCACAAGCTTCGCCCCTGCTGAAAGCGGTTTACAACCCGAAGGCCGTCATCCCGCACGCGGCGTTGCTGCATCAGGCTTCCGCCCATTGTGCAATATTCCCCACTGCTGCCTCCCGTAGGAGTCTGGGCCGTATCTCAGTCCCAATGTGGCCGGTCGCCCTCTCAGGCCGGCTACCCGTCAAAGCCTTGGTAAGCCACTACCCCACCAACAAGCTGATAAGCCGCGAGTCCATCCAAAACCGCCGAAGCTTTTCCAACCCCCACCATGCAGCAAGGATTCCTATCCGGTATTAGCCCCAGTTTCCTGAAGTTATCCCGAAGTCAAGGGCA
>n.538.6.bb_657
GTAGTTAGCCGTGGCTTTCTGATTAGGTACCGTCAAGACGTGCATAGTTACTTACACATTTGTTCTTCCCTAATAACAGAGTTTTACGATCCGAAGACCTTCATCACTCACGCGGCGTTGCTCCGTCAGGCTTTCGCCCATTGCGGAAGATTCCCTACTGCTGCCTCCCGTAGGAGTCTGGACCGTGTCTCAGTTCCAGTGTGGCCGATCACCCTCTCAGGGTCGGCTACGCATCGTTGCCTTGGTAAGCCGTTACCTTACCAACTAGCTAATGCGGCGCGGATCCATCTATAAGTGACAGCAAAACCGTCTTTCACTATTGAACCATGCGGTTCAATATATTATCCGGTATT
>n.538.6.bb_658
GTAGTTAGCCGGTGCTTCTTCTGCAGGTACCGTCACCACAAGCTTCGCCCCTGCTGAAAGCGGTTTACAACCCGAAGGCCGTCATCCCGCACGCGGCGTTGCTGCATCAGGCTTCCGCCCATTGTGCAATATTCCCCACTGCTGCCTCCCGTAGGAGTCTGGGCCGTATCTCAGTCCCAATGTGGCCGGTCGCCCTCTCAGGGCCGGCTACCCGTCAAAGGCCTTGGTAAGCCACTACCCCACCAACAAGCTGATAAGCCGCGAGTCCATCCAAAACCGCC
>n.538.6.bb_659
CGTAGTTAGCCGTGGCTTTCTGATTAGGTACCGTCAAGACGTGCATAGTTACTTACACATTTGTTCTTCCCTAATAACAGAGTTTTACGATCCGAAGACCTTCATCACTCACGCGGCGTTGCTCCGTCAGGCTTTCGCCCATTGCGGAAGATTCCCTACTGCTGCCTCCCGTAGGAGTCTGGACCGTGTCTCAGTTCCAGTGTGGCCGATCACCCTCTCAGGGTCGGCTACGCATCGTTGCCTTGGTAAGCCGTTACCTTACCAACTAGCTAATGCGGCGTGGATCCATCTATAAGTGACAGCAAAACCGTCTTTCACTATTGAACCATGCGGTTCAATATATTATCCGGTATT
>n.538.6.bb_660
GTAGTTAGCCGGTGCTTCTTCTGCAGGTACCGTCACCACAAGCTTCGCCCCTGCTGAAAGCGGTTTACAACCCGAAGGCCGTCATCCCGCACGCGGCGTTGCTGCATCAGGCTTCCGCCCATTGTGCAATATTCCCCACTGCTGCCTCCCGTAGGAGTCTGGGCCGTATCTCAGTCCCAATGTGGCCCGGTCGCCCTCTCAGGCCGGCTACCCGGTNAAAGCCTTGGTAAGCCACTACCCCACCAACAAGCTGATAAGCCGCGAGTCCATCCAAAACCGCCGAAGCTTTCCAACCCCCACCATGCAGCAAGGATTCCTATCCGGTAT
>n.538.6.bb_661
GTAGTTAGCCGGTGCTTCTTCTGCAGGTACCGTCACCACAAGCTTCGCCCCTGCTGAAAGCGGTTTACAACCCGAAGGCCGTCATCCCGCACGCGGCGTTGCTGCATCAGGCTTCCGCCCATTGTGCAATATTCCCCACTGCTGCCTCCCGTAGGAGTCTGGGCCGTATCTCAGTCCCAATGTGGCCGGTCGCCCTCTCAGGCCGGCTACCCGTCAAAGCCTTGGTAAGCCACTACCCCACCAACAAGCTGATAAGCCGCGAGTCCATCC
>n.538.6.bb_662
GTAGTTAGCCGTGGCTTTCTGATTAGGTACCGTCAAGACGTGCATAGTTACTTACACATTTGTTCTTCCCTAATAACAGAGTTTTACGATCCGAAGACCTTCATCACTCACGCGGCGTTGCTCCGTCAGGCTTTCGCCCATTGCGGAAGATTCCCTACTGCTGCCTCCCGTAGGAGTCTGGACCGTGTCTCAGTTCCAGTGTGGCCGATCACCCTCTCAGGGTCGGCTACGCATCGTCGCCTTGGTAAGCCGTTACCTTACCACTAGCTAATGCGGCGCGGATCCATCTATAAGTGAC
>n.538.6.bb_663
GTAGTTAGCCGGTGCTTCTTCTGCAGGTACCGTCACCACAAGCTTCGCCCCTGCTGAAAGCGGTTTACAACCCGAAGGCCGTCATCCCGCACGCGGCGTTGCTGCATCAGGCTTCCGCCCATTGTGCAATATTCCCCACTGCTGCCTCCCGTAGGAGTCTGGGCCGTATCTCAGTCCCAATGTGGCCGGTCGCCCTCTCAGGCCGGCTACCCGTCAAAGCCTTGGTAAGCCACTACCCCACCAACAAGCTGATAAGCCGCGAGTCCATCCAAAACCGCCGAAGCTTTTCCAACCCCACCATGCAGCAAGGATTCCTATCCGGTATTAGCCCCAGTTTCCTGAAGTTATCCCGAAGTCAAGGGCAGGTTACTCACGT
>n.538.6.bb_664
GTAGTTAGCCGTGGCTTTCTGATTAGGTACCGTCAAGACGTGCATAGTTACTTACACATTTGTTCTTCCCTAATAACAGAGTTTTACGATCCGAAGACCTTCATCACTCACGCGGCGTTGCTCCGTCAGGCTTTCGCCCATTGCGGAAGATTCCCTACTGCTGCCTCCCGTAGGAGTCTGGACCGTGTCTCAGTTCCAGTGTGGCCGATCACCCTCTCAGGGTCGGCTACGCATCGTTGCCTTGGTAAGCCGTTACCTTACCAACTAGCTAATGCGGCGCGGATCCATCTATAAGTGACAGCAAAACCGTCTTT
>n.538.6.bb_665
GTAGTTAGCCGGTGCTTCTTCTGCAGGTACCGTCACCACAAGCTTCGCCCCTGCTGAAAGCGGTTTACAACCCGAAGGCCGTCATCCCGCACGCGGCGTTGCTGCATCAGGCTTCCGCCCATTGTGCAATATTCCCCACTGCTGCCTCCCGTAGGAGTCTGGGCCGTATCTCAGTCCCAATGTGGCCGGTCGCCCTCTCAGGCCGGCTACCCGTCAAAGCCTTGGTAAGCCACTACCCCACCAACAAGCTGATAAGCCGCGAGTCCATCCAAAACCGCCGAAGCTTTCCAACCCCACCATGCAGCAAGGATTCCTAT
>n.538.6.bb_666
GTAGTTAGCCGGTGCTTCTTCTGCAGGTACCGTCACCACAAGCTTCGCCCCTGCTGAAAGCGGTTTACAACCCGAAGGCCGTCATCCCGCACGCGGCGTTGCTGCATCAGGCTTCCGCCCATTGTGCAATATTCCCCACTGCTGCCTCCCGTAGGAGTCTGGGCCGTATCTCAGTCCCAATGTGGCCGGTCGCCCTCTCAGGCCGGCTACCCGTCAAAGCCTTGGTAAGCCACTACCCACCAACAAGCTGATAAGCCGCGAGTCCATCCAAAACCGCCG
>n.538.6.bb_667
GTAGTTAGCCGTGGCTTTCTGATTAGGTACCGTCAAGACGTGCATAGTTACTTACACATTTGTTCTTCCCTAATAACAGAGTTTTACGATCCGAAGACCTTCATCACTCACGCGGCGTTGCTCCGTCAGGCTTTCGCCCATTGCGGAAGATTCCCTACTGCTGCCTCCCGTAGGAGTCTGGACCGTGTCTCAGTTCCAGTGTGGCCGATCACCCTCTCAGGGTCGGCTACGCATCGTTGCCTTGGTAAGCCGTTACCTTACCAACTAGCTAATGCGGCGCGGATCCATCTATAAGTGACAGCAAAACCGTCTTTCACTATTGAACCATGCGGTTCAATATATTATCCGGTATTAGCTCCGGTTTCC
>n.538.6.bb_668
GTAGTTAGCCGTGGCTTTCTGATTAGGTACCGTCAAGACGTGCATAGTTACTTACACATTTGTTCTTCCCTAATAACAGAGTTTTACGATCCGAAGACCTTCATCACTCACGCGGCGTTGCTCCGTCAGGCTTTCGCCCATTGCGGAAGATTCCCTACTGCTGCCTCCCGTAGGAGTCTGGACCGTGTCTCAGTTCCAGTGTGGCCGATCACCCTCTCAGGGTCGCTACGCATCGTCGCCTTGGTAAGCCGTTACCTTACCAACTAGCTAATGCGGCGCGGATCCATCTATAAGTGACAGCAAAACCGTCTTTCACTATTGAACCATGCGGTTCAATA
>n.538.6.bb_669
GTAGTTAGCCGTGGCTTTCTGATTAGGTACCGTCAAGACGTGCATAGTTACTTACACATTTGTTCTTCCCTAATAACAGAGTTTTACGATCCGAAGACCTTCATCACTCACGCGGCGTTGCTCCGTCAGGCTTTCGCCCATTGCGGAGGATTCCCTACTGCTGCCTCCCGTAGGAGTCTGGACCGTGTCTCAGTTCCAGTGTGGCCGATCACCCTCTCAGGGTCGGCTACGCATCGTTGCCTTGGTAAGCCGTTACCTTACCAACTAGCTAATGCGGCGCGGATCCATCTATAAGTGACAGCAAAACCGTCTTTCACTATTGAACCATGCGGTTCAATATATTATCCGGTA
>n.538.6.bb_670
GTAGTTAGCCGTGGCTTTCTGATTAGGTACCGTCAAGACGTGCATAGTTACTTACACATTTGTTCTTCCCTAATAACAGAGTTTTACGATCCGAAGACCTTCATCACTCACGCGGCGTTGCTCCGTCAGGCTTTCGCCCATTGCGGAAGATTCCCTACTGCTGCCTCCCGTAGGAGTCTGGACCGTGTCTCAGTTCCAGTGTGGCCGATCACCCTCTCAGGGTCGGCTACGCATCGTCGCCTTGGTAAGCCGTTACCTTACCAACTAGCTAATGCGGCGCGGATCCATCTATAAGTGACAGCAAAACCGTCTTTCACTATTGAACCATGCGGTTCAATATAT
>n.538.6.bb_671
GTAGTTAGCCGGTGCTTCTTCTGCAGGTACCGTCACCACAAGCTTCGCCCCTGCTGAAAGCGGTTTACAACCCGAAGGCCGTCATCCCGCACGCGGCGTTGCTGCATCAGGCTTCCGCCCATTGTGCAATATTCCCCACTGCTGCCTCCCGTAGGAGTCTGGGCCGTATCTCAGTCCCAATGTGGCCGGTCGCCCTCTCAGGCCGGCTACCCGTCAAAAGCCTTGGTAAGCCACTACCCCACCAACAAGCTGATAAGCCGCGAGTCCATCCAAAACCGC
>n.538.6.bb_672
GTAGTTAGCCGGTGCTTCTTCTGCAGGTACCGTCACCACAAGCTTCGCCCCTGCTGAAAGCGGTTTACAACCCGAAGGCCGTCATCCCGCACGCGGCGTTGCTGCATCAGGCTTCCGCCCATTGTGCAATATTCCCCACTGCTGCCTCCCGTAGGAGTCTGGGCCGTATCTCAGTCCCAATGTGGCCGGTCGCCCTCTCAGGCCGGCTACCCGGTCAAAGCCTTGGTAACGCCACTACCCCACCAACAAGCTGATAAGCCGCGAGTCCATCCAAAACCGCCGA
>n.538.6.bb_673
GTAGTTAGCCGTGGCTTTCTGATTAGGTACCGTCAAGACGTGCATAGTTACTTACACATTTGTTCTTCCCTAATAACAGAGTTTTACGATCCGAAGACCTTCATCACTCACGCGGCGTTGCTCCGTCAGGCTTTCGCCCATTGCGGAAGATTCCCTACTGCTGCCTCCCGTAGGAGTCTGGACCGTGTCTCAGTTCCAGTGTGGCCGATCACCCTCTCAGGGTCGGCTACGCATCGTTGCCTTGGTAAGCCGTTACCTTACCAACTAGCTAATGCGGCGCGGATCCATCTATAAGTGACAGCAAAACCGTCT
>n.538.6.bb_674
GTAGTTAGCCGTGGCTTTCTGATTAGGTACCGTCAAGACGTGCATAGTTACTTACACATTTGTTCTTCCCTAATAACAGAGTTTTACGATCCGAAGACCTTCATCACTCACGCGGCGTTGCTCCGTCAGGCTTTCGCCCATTGCGGAAGATTCCCTACTGCTGCCTCCCGTAGGAGTCTGGACCGTGTCTCAGTTCCAGTGTGGCCGATCACCCTCTCAGGGTCGGCTACGCATCGTCGCCTTGGTAAGCCGTTACCTTACCAACTAGCTAATGCGGCGCGGATCCATCTATAAGTGACAGCAAAACCGTCTTTCACTATTGAACCA
>n.538.6.bb_675
GTAGTTAGCCGGTGCTTCTTCTGCAGGTACCGTCACCACAAGCTTCGCCCCTGCTGAAAGCGGTTTACAACCCGAAGGCCGTCATCCCGCACGCGGCGTTGCTGCATCAGGCTTCCGCCCATTGTGCAATATTCCCCACTGCTGCCTCCCGTAGGAGTCTGGGCCGTATCTCAGTCCCAATGTGGCCGGTCGCCCTCTCAGGCCGGCTACCCGTCAAAGCCTTGGTAACGCCACTACCCACCAACAAGCTGATAAGCCGCGAGTCCATCC
>n.538.6.bb_676
GTAGTTAGCCGTGGCTTTCTGATTAGGTACCGTCAAGACGTGCATAGTTACTTACACATTTGTTCTTCCCTAATAACAGAGTTTTACGATCCGAAGACCTTCATCACTCACGCGGCGTTGCTCCGTCAGGCTTTCGCTCATTGCGGAAGATTCCCTACTGCTGCCTCCCGTAGGAGTCTGGACCGTGTCTCAGTTCCAGTGTGGCCGATCACCCTCTCAGGTCGGCTACGCATCGTTGCCTTGGTAAGCCGTTACCTTACCAACTAGCTAATGCGGCGCGGATCCATCTATAAGTGACAGCAAAACCGTCTTTCACTATTGAACCATGCGGTTCAATATATTATCCGGTATTAGCTCCGGTTTCCCGAAGTTATCCCAGTCTTATAGGTAGG
>n.538.6.bb_677
GTAGTTAGCCGTGGCTTTCTGATTAGGTACCGTCAAGACGTGCATAGTTACTTACACATTTGTTCTTCCCTAATAACAGAGTTTTACGATCCGAAGACCTTCATCACTCACGCGGCGTTGCTCCGTCAGGCTTTCGCCCATTGCGGAAGATTCCCTACTGCTGCCTCCCGTAGGAGTCTGGACCGTGTCTCAGTTCCAGTGTGGCCGATCACCCTCTCAGGGTCGGCTACGCATCGTTGCCTTGGTAAGCCGTTACCTTACCAACTAGCTAATGCGGCGCGGATCCATCTATAAGTGACAGCAAAACCGTCTTTCACTATTGAACCATGCGGTTCAATATATTATCCGGTATT
>n.538.6.bb_678
GTAGTTAGCCGTGGCTTTCTGATTAGGTACCGTCAAGACGTGCATAGTTACTTACACATTTATTCTTCCCTAATAACAGAGTTTTACGATCCGAAGACCTTCATCACTCGCGCGGCGTTGCTCCGTCAGGCTTTCGCCCATTGCGGAAGATTCCCTACTGCTGCCTCCCGTAGGAGTCTGGACCGTGTCTCAGTTCCAGTGTGGCCGATCACCCTCTCAGGGTCGGCTACGCATCGTTGCCTTGGTAAGCCGTTACCTTACCAACTTAGCTAATGCGGCGCGGATCCATCTATAAAGTGACAGCAAAACCGTCTTTCACTATTGAACCATGCGGTTCAATATATTATCCGGTATTAGCTCCGG
>n.538.6.bb_679
GTAGTTAGCCGGTGCTTCTTCTGCAGGTACCGTCACCACAAGCTTCGCCCCTGCTGAAAGCGGTTTACAACCCGAAGGCCGTCATCCCGCACGCGGCGTTGCTGCATCAGGCTTCCGCCCATTGTGCAATATTCCCCACTGCTGCCTCCCGTAGGAGTCTGGGCCGTATCTCAGTCCCAATGTGGCCGGTCGCCCTCTCAGGCCGGCTACCCGTCAAAGCCTTGGTAAGCCACTACCCACCAACAAGCTGATAAGCCGCGAGTCCATCCAAAACCGC
>n.538.6.bb_680
GGAGTTAGCCGGTGCTTCTTCTGCGGGTAACGTCAATCGACAAGGTTATTAACCTTATCGCCTTCCTCCCCGCTGAAAGTACTTTACAACCCGAAGGCCTTCTTCATACACGCGGCATGGCTGCATCAGGCTTGCGCCCATTGTGCAATATTCCCCACTGCTGCCTCCCGTAGGAGTCTGGACCGTGTCTCAGTTCCAGTGTGGCTGGTCATCCTCTCAGACCAGCTAGGGATCGTCGCCTAGGTGAGCCGTTACCCACCTACTAGCTAATCCCATCTGGGCACATCTGATGGC
>n.538.6.bb_681
GTAGTTAGCCGTGGCTTTCTGATTAGGTACCGTCAAGACGTGCATAGTTACTTACACATTTGTTCTTCCCTAATAACAGAGTTTTACGATCCGAAGACCTTCATCACTCACGCGGCGTTGCTCCGTCAGGCTTTCGCCCATTGCGGAAGATTCCCTACTGCTGCCTCCCGTAGGAGTCTGGACCGTGTCTCAGTTCCAGTGTGGCCGATCACCCTCTCAGGGTCGGCTACGCATCGTCGCCTTGGTAAGCCGTTACCTTACCAACTAGCTAATGCGGCGCGGATCCATCTATAAGTGACAGC
>n.538.6.bb_682
GGAGTTAGCCGGTGCTTCTTCTGCGGGTAACGTCAATTGCTGAGGTTATTAACCTCGACACCTTCCTCCCCGCTGAAAGTACTTTACAACCCGAAGGCCTTCTTCATACACGCGGCATGGCTGCATCAGGCTTGCGCCCATTGTGCAATATTCCCCACTGCTGCCTCCCGTAGGAGTCTGGACCGTGTCTCAGTTCCAGTGTGGCTGGTCATCCTCTCAGACCAGCTAGGGATCGTCGCCTAGGTGAGCCGTTACCCACCTACTAGCTAATCCCATCTGGGCAC
>n.538.6.bb_683
GTAGTTAGCCGTGGCTTTCTGATTAGGTACCGTCAAGACGTGCATAGTTACTTACACATTTGTTCTTCCCTAATAACAGAGTTTTACGATCCGAAGACCTTCATCACTCACGCGGCGTTGCTCCGTCAGGCTTTCGCCCATTGCGGAAGATTCCCTACTGCTGCCTCCCGTAGGAGTCTGGACCGTGTCTCAGTTCCAGTGTGGCCGATCACCCTCTCAGGGTCGGCTACGCATCGTTGCCTTGGTAAGCCGTTACCTTACCAACTAGCTAATGCGGCGCGGATCCATCTATAAGTGACAGCAAAACCGTCTTTCACTATTGAACCATGCGGTTCAATATATTATCCGGTATTAGCTCCGGT
>n.538.6.bb_684
GTAGTTAGCCGTGGCTTTCTGATTAGGTACCGTCAAGACGTGCATAGTTACTTACACATTTGTTCTTCCCTAATAACAGAGTTTTACGATCCGAAGACCTTCATCACTCACGCGGCGTTGCTCCGTCAGGCTTTCGCCCATTGCGGAAGATTCCCTACTGCTGCCTCCCGTAGGAGTCTGGACCGTGTCTCAGTTCCAGTGTGGCCGATCACCCTCTCAGGGTCGGCTACGCATCGTTGCCTTGGTAAGCCGTTACCTTACCAACTAGCTAATGCGGCGCGGATCCATCTATAAGTGACAGCAAAACCGTCTTTCACTATTGAACCATGCGGTTCAATATATTATCCGGTATTAGCTCCGG
>n.538.6.bb_685
GTAGTTAGCCGTGGCTTTCTGATTAGGTACCGTCAAGACGTGCATAGTTACTTACACATTTGTTCTTCCCTAATAACAGAGTTTTACGATCCGAAGACCTTCATCACTCACGCGGCGTTGCTCCGTCAGGCTTTCGCCCATTGCGGAAGATTCCCTACTGCTGCCTCCCGTAGGAGTCTGGACCGTGTCTCAGTTCCAGTGTGGCCGATCACCCTCTCAGGGTCGGCTACGCATCGTTGCCTTGGTAAGCCGTTACCTTACCAACTAGCTAATGCGGCGCGGATCCATCTATAAGTGACAGCAAAACCGTCTTTCACTATTGAACCATGCGGTTCAATATATTATCCGGTATTAGCTCCGGTTTCCCGAAGTTATCCCAGTCTTATAGGTAGGTTATCC
>n.538.6.bb_686
GTAGTTAGCCGGTGCTTCTTCTGCAGGTACCGTCACCACAAGCTTCGCCCCTGCTGAAAGCGGTTTACAACCCGAAGGCCGTCATCCCGCACGCGGCGTTGCTGCATCAGGCTTCCGCCCATTGTGCAATATTCCCCACTGCTGCCTCCCGTAGGAGTCTGGGCCGTATCTCAGTCCCAATGTGGCCGGTCGCCCTCTCAGGCCGGCTACCCGGTCAAAGGCCTTGGTAAGCCACTACCCCACCAACAAGCTGATAAGCCGCGAGTCCATCCAAAACCGCCGAAGCTTTCCAACCCCACCATGCAGCAAGGATTCCTATCCGGTATTAGCCCC
>n.538.6.bb_687
GTAGTTAGCCGTGGCTTTCTGATTAGGTACCGTCAAGACGTGCATAGTTACTTACACATTTGTTCTTCCCTAATAACAGAGTTTTACGATCCGAAGACCTTCATCACTCACGCGGCGTTGCTCCGTCAGGCTTTCGCCCATTGCGGAAGATTCCCTACTGCTGCCTCCCGTAGGAGTCTGGACCGTGTCTCAGTTCCAGTGTGGCCGATCACCCTCTCAGGGTCGGCTACGCATCGTTGCCTTGGTAAGCCGTT
>n.538.6.bb_688
GTAGTTAGCCGGTGCTTCTTCTGCAGGTACCGTCACCACAAGCTTCGCCCCTGCTGAAGCGGTTTACAACCCGAAGGCCGTCATCCCGCACGCGGCGTTGCTGCATCAGGCTTCCGCCCATTGTGCAATATTCCCCACTGCTGCCTCCCGTAGGAGTCTGGGCCGTATCTCAGTCCCAATGTGGCCGGTCGCCCTCTCAGGCCGGCTACCCGGTCAAAGCCTTGGTAAGCCACTACCCACCAACAAGCTGATAAGCCGCGAGTCCATCCAAGACCGCCG
>n.538.6.bb_689
GTAGTTAGCCGGTGCTTCTTCTGCAGGTACCGTCACCACAAGCTTCGCCCCTGCTGAAAGCGGTTTACAACCCGAAGGCCGTCATCCCGCACGCGGCGTTGCTGCATCAGGCTTCCGCCCATTGTGCAATATTCCCCACTGCTGCCTCCCGTAGGAGTCTGGACCGTATCTCAGTCCCAATGTGGCCGGTCGCCCTCTCAGGCCGGCTACCCGTCAAAGCCTTGGTAAGCCACTACCCCACCAACAAGCTGATAAGCCGCGAGTCCATCCAAAACCGCCGAAGCTTTCCAACCCCACCATGCAGCAAGGATTCCTATCCGGTATTA
>n.538.6.bb_690
GTAGTTAGCCGTGGCTTTCTGATTAGGTACCGTCAAGACGTGCATAGTTACTTACACATTTGTTCTTCCCTAATAACAGAGTTTTACGATCCGAAGACCTTCATCACTCACGCGGCGTTGCTCCGTCAGGCTTTCGCCCATTGCGGAAGATTCCCTACTGCTGCCTCCCGTAGGAGTCTGGACCGTGTCTCAGTTCCAGTGTGGCCGATCACCCTCTCAGGGTCGGCTACGCATCGTTGCCTTGGTAAGCCGTTACCTTACCAACTAGCTAATGCGGCGCGGATCCATCTATAAGTGACAGCAAAACCGTCTTTCACTATTGAACCATGCGGTTCAATATATTATCCGGTATTAGCTCCGG
>n.538.6.bb_691
GTAGTTAGCCGGTGCTTCTTCTGCAGGTACCGTCACCACAAGCTTCGCCCCTGCTGAAAGCGGTTTACAACCCGAAGGCCGTCATCCCGCACGCGGCGTTGCTGCATCAGGCTTCCGCCCATTGTGCAATATTCCCCACTGCTGCCTCCCGTAGGAGTCTGGGCCGTATCTCAGTCCCAATGTGGCCGGTCGCCCTCTCAGGCCGGCTACCCGTCAAAGCCTTGGTAAGCCGCTACCCCACCAACAAGCTGATAAGCCGCGAGTCCATCCAAAACCGCCGAAGCTTTCCAACCCCACCATGCAGCAAGGATTCCTATCCGGTATTAGCCCCAGTTTCCTGAAGTTATCCCGAAGTCAAGGGCAGGTTACTCACGTGTTACTCACCCGTT
>n.538.6.bb_692
GTAGTTAGCCGGTGCTTCTTCTGCAGGTACCGTCACCACAAGCTTCGCCCCTGCTGAAAGCGGTTTACAACCCGAAGGCCGTCATCCCGCACGCGGCGTTGCTGCATCAGGCTTCCGCCCATTGTGCAATATTCCCCACTGCTGCCTCCCGTAGGAGTCTGGGCCGTATCTCAGTCCCAATGTGGCCGGTCGCCCTCTCAGGCCGGCTACCCGGTCAAAGCCTTGGTAAGCCACTACCCACCAACAAGCTGATAAGCCGCGAGTCCATCCAAAACCGCC
>n.538.6.bb_693
GTAGTTAGCCGGTGCTTCTTCTGCAGGTACCGTCACCACAAGCTTCGCCCCTGCTGAAAGCGGTTTACAACCCGAAGGCCGTCATCCCGCACGCGGCGTTGCTGCATCAGGCTTCCGCCCATTGTGCAATATTCCCCACTGCTGCCTCCCGTAGGAGTCTGGGCCGTATCTCAGTCCCAATGTGGCCGGTCGCCCTCTCAGGCCGGCTAC
>n.538.6.bb_694
GTAGTTAGCCGTGGCTTTCTGATTAGGTACCGTCAAGACGTGCATAGTTACTTACACATTTGTTCTTCCCTAATAACAGAGTTTTACGATCCGAAGACCTTCATCACTCACGCGGCGTTGCTCCGTCAAGCTTTCGCCCATTGCGGAAGATTCCCTACTGCTGCCTCCCGTAGGGGTCTGGACCGTGTCTCAGTTCCAGTGTGGCCGATCACCCTCTCAGGGTCGGCTACGCATCGTTGCCTTGGTAAGCCGTTACCTTACCAACTAGCTAATGCGGCGCGGATCCATCTA
>n.538.6.bb_695
GTAGTTAGCCGGTGCTTCTTCTGCAGGTACCGTCACCACAAGCTTCGCCCCTGCTGAAAGCGGTTTACAACCCGAAGGCCGTCATCCCGCACGCGGCGTTGCTGCATCAGGCTTCCGCCCATTGTGCAATATTCCCCACTGCTGCCTCCCGTAGGAGTCTGGGCCGTATCTCAGTCCCAATGTGGCCGGTCGCCCTCTCAGGCCGGCTACCCGTCAAAGCCTTGGTAAGCCACTACCCCACCAACAAGCTGATAAGCCGCGAGTCCATCCAAAACCGC
>n.538.6.bb_696
GTAGTTAGCCGGTGCTTCTTCTGCAGGTACCGTCACCACAAGCTTCGCCCCTGCTGAAAGCGGTTTACAACCCGAAGGCCGTCATCCCGCACGCGGCGTTGCTGCATCAGGCTTCCGCCCATTGTGCAATATTCCCCACTGCTGCCTCCCGTAGGAGTCTGGGCCGTATCTCAGTCCCAATGTGGCCGGTCGCCCTCTCAGGCCCGGCTACCCCGGTCAAAGCCTTGGTAAGCCACTACCCCACCAACAAGCTGATAAGCCGCGAGTCCATCCAAAACCGCCG
>n.538.6.bb_697
GTAGTTAGCCGTGGCTTTCTGATTAGGTACCGTCAAGACGTGCATAGTTACTTACACATTTGTTCTTCCCTAATAACAGAGTTTTACGATCCGAAGACCTTCATCACTCACGCGGCGTTGCTCCGTCAGGCTTTCGCCCATTGCGGAAGATTCCCTACTGCTGCCTCCCGTAGGAGTCTGGACCGTGTCTCAGTTCCAGTGTGGCCGATCACCCTCTCAGGGTCGGCTACGCATCGTTGCCTTGGTAAGCCGTTACCTTACCAACTAGCTAATGCGGCGCGGATCCATCTATAAGTGACAGCAAAACCGTCTTTCACTATTGAACCATGCGGTTCAATATATTATCCGGTATT
>n.538.6.bb_698
GTAGTTAGCCGTGGCTTTCTGATTAGGTACCGTCAAGACGTGCATAGTTACTTACACATTTGTTCTTCCCTAATAACAGAGTTTTACGATCCGAAGACCTTCATCACTCACGCGGCGTTGCTCCGTCAGGCTTTCGCCCATTGCGGAAGATTCCCTACTGCTGCCTCCCGTAGGAGTCTGGACCGTGTCTCAGTTCCAGTGTGGCCGATCACCCTNTCAGGGTCGGCTACGCATCGTTGC
>n.538.6.bb_699
GTAGTTAGCCGTGGCTTTCTGATTAGGTACCGTCAAGACGTGCATAGTTACTTACACATTTGTTCTTCCCTAATAACAGAGTTTTACGATCCGAAGACCTTCATCACTCACGCGGCGTTGCTCCGTCAGGCTTTCGCCCATTGCGGAAGATTCCCTACTGCTGCCTCCCGTAGGAGTCTGGACCGTGTCTCAGTTCCAGTGTGGCCGATCACCCTCTCAGGGTCGGCTACGCATCGTCGCCTTGGTAAGCCGTTACCTTACCAACTAGCTAATGCGGCGCGGATCCATCTATAAGTGACAGCAAAACCGTCTTTCACTATTGAACCATGCGGTTCAATATAT
>n.538.6.bb_700
GTAGTTAGCCGTGGCTTTCTGATTAGGTACCGTCAAGACGTGCATAGTTACTTACACATTTGTTCTTCCCTAATAACAGAGTTTTACGATCCGAAGACCTTCATCACTCACGCGGCGTTGCTCCGTCAGGCTTTCGCCCATTGCGGAAGATTCCCTACTGCTGCCTCCCGTAGGAGTCTGGACCGTGTCTCAGTTCCAGTGTGGCCGATCACCCTCTCAGGGTCGGCTACGCATCGTTGCCTTGGTAAGCCGTTACCTTACCAACTAGCTAATGCGGCGCGGATCCATCTATAAGTGACAGCAAAACCGTCTTTCACTATTGAACCATGCGGTTCAATATATTATCCGGTATTAGCTCCGGTTTCCGAAGTTATCCCAGTCTTATAGGTAGGTTA
>n.538.6.bb_701
GTAGTTAGCCGTGGCTTTCTGATTAGGTACCGTCAAGACGTGCATAGTTACTTACACATTTGTTCTTCCCTAATAACAGAGTTTTACGATCCGAAGACCTTCATCACTCACGCGGCGTTGCTCCGTCAGGCTTTCGCCCATTGCGGAAGATTCCCTACTGCTGCCTCCCGTAGGAGTCTGGACCGTGTCTCAGTTCCAGTGTGGCCGATCACCCTCTCAGGGTCGGCTACGCATCGTCGCCTTGGTAAGCCGTTACCTTACCAACTAGCTAATGCGGCGCGGATCCATCTATAAGTGACAG
>n.538.6.bb_702
GTAGTTAGCCGGTGCTTCTTCTGCAGGTACCGTCACCACAAGCTTCGCCCCTGCTGAAAGCGGTTTACAACCCGAAGGCCGTCATCCCGCACGCGGCGTTGCTGCATCAGGCTTCCGCCCATTGTGCAATATTCCCCACTGCTGCCTCCCGTAGGAGTCTGGGCCGTATCTCAGTCCCAATGTGGCCGGTCGCCCTCTCAGGCCGGCTACCCGTCAAAGCCTTGGTAGCCACTACCCCACCAACAAGCTGATAAGCCGCGAGTCCATCC
>n.538.6.bb_703
GTAGTTAGCCGTGGCTTTCTGATTAGGTACCGTCAAGACGTGCATAGTTACTTACACATTTGTTCTTCCCTAATAACAGAGTTTTACGATCCGAAGACCTTCATCACTCACGCGGCGTTGCTCCGTCAGGCTTTCGCCCATTGCGGAAGATTCCCTACTGCTGCCTCCCGTAGGAGTCTGGACCGTGTCTCAGTTCCAGTGTGGCCGATCACCCTCTCAGGGTCGGCTACGCATCGTTGCCTTGGTAAGCCGTTACCTTACCAACTAGCTAATGCGGCGCGGATCCATCTATAAGTGACAGCAAAACCGTCTTTCACTATTGAACCATGCGGTTCAATATATTATCCGGTATT
>n.538.6.bb_704
GTAGTTAGCCGTGGCTTTCTGATTAGGTACCGTCAAGACGTGCATAGTTACTTACACATTTGTTCTTCCCTAATAACAGAGTTTTACGATCCGAAGACCTTCATCACTCACGCGGCGTTGCTCCGTCAGGCTTTCGCCCATTGCGGAAGATTCCCTACTGCTGCCTCCCGTAGGAGTCTGGACCGTGTCTCAGTTCCAGTGTGGCCGATCACCCTCTCAGGGTCGGCTACGCATCGTTGCCTTGGTAAGCCGTTACCTTACCAACTAGCTAATGCGGCGCGGAT
>n.538.6.bb_705
GGAGTTAGCCGGTGCCTCTTCTGCGGGTAACGTCAATTGCTGAGGTTATTAACCTCAACACCTTCCTCCCCGCTGAAAGTACTTTACAACCCGAAGGCCTTCTTCATACACGCGGCATGGCTGCATCAGGCTTGCGCCCATTGTGCAATATTCCCCACTGCTGCCTCCCGTAGGAGTCTGGACCGTGTCTCAGTTCCAGTGTGGCTGGGTCATCCTCTCAGACCAGCTAGGGATCGTCGCCTAGGTGAGCCGTTACCCACCTACTAGCTAATCCCATCTGGGCACATCTG
>n.538.6.bb_706
GTAGTTAGCCGTGGCTTTCTGATTAGGTACCGTCAAGACGTGCATAGTTACTTACACATTTATTCTTCCCTAATAACAGAGTTTTACGATCCGAAGACCTTCATCACTCACGCGGCGTTGCTCCGTCAGGCTTTCGCCCATTGCGGAAGATTCCCTACTGCTGCCTCCCGTAGGAGTCTGGACCGTGTCTCAGTTCCAGTGTGGCCGATCACCCTCTCGAGGGTCGGCTACGCATCGTTGCCTTGGTAAGCCGTTACCTTACCAACTAGCTAATGCGGCGCGGATCCATCT
>n.538.6.bb_707
GTAGTTAGCCGTGGCTTTCTGATTAGGTACCGTCAAGACGTGCATAGTTACTTACACATTTGTTCTTCCCTAATAACAGAGTTTTACGATCCGAAGACCTTCATCACTCACGCGGCGTTGCTCCGTCAGGCTTTCGCCCATTGCGGAAGATTCCCTACTGCTGCCTCCCGTAGGAGTCTGGACCGTGTCTCAGTTCCAGTGTGGCCGATCACCCTCTCAGGGTCGGCTACGCATCGTTGCCTTGGTAAGCCGTTACCTTACCAACTAGCTAATGCGGCGCGGATCCATCTATAAGTGACAGCAAAACCGTCTTTCACTATTGAACCATGCGGTTCAATATATTATCCGGTATTAGCTCCGGTTTCCCGAAGTTATCCCAGTCTTATAGGTAGGTTATCCCACCGTGTTACTCACCCCGTCCCGCCCGCTAACGTCAGAGGGAGCAAGCCTCCCTCGTCTGTTCGCTCGACTTGCATGTATTAGGGC
>n.538.6.bb_708
GTAGTTAGCCGTGGCTTTCTGATTAGGTACCGTCAAGACGTGCATAGTTACTTACACATTTGTTCTTCCCTAATAACAGAGTTTTACGATCCGAAGACCTTCATCACTCACGCGGCGTTGCTCCGTCAGGCTTTCGCCCATTGCGGAAGATTCCCTACTGCTGCCTCCCGTAGGAGTCTGGACCGTGTCTCAGTTCCAGTGTGGCCGATCACCCTCTCAGGGTCGGCTACGCATCGTTGCCTTGGTAAGCCGTTACCTTACCAACTAGCTAATGCGGCGCGGATCCATCTATAAGTGACAGCAAAACCGTCTTTCACTATTGAACCATGCGGTTCAATATATTATCCGGTATT
>n.538.6.bb_709
GTAGTTAGCCGTGGCTTTCTGATTAGGTACCGTCAAGACGTGCATAGTTACTTACACATTTATTCTTCCCTAATAACAGAGTTTTACGATCCGAAGACCTTCATCACTCACGCGGCGTTGCTCCGTCAGGCTTTCGCCCATTGCGGAAGATTCCCTACTGCTGCCTCCCGTAGGAGTCTGGACCGTGTCTCAGTTCCAGTGTGGCCGATCACCCTCTCAGGGTCGGCTACGCATCGTTGCCTTGGTAAGCCGTTACCTTACCAACTAGCTAATGCGGCGCGGATCCATCTATAAGTGACAGCAAAACCGTCTTTCACTATTGAACCATGCGGTTCAATATATTATCCGGTATTAGCTCCGGTTTCCCGAAGTTATCCCAGTCTTATAGGTAGGTTATCCCACCGTGTTACCTCACCCCGTCCCGCCCGCT
>n.538.6.bb_710
GTAGTTAGCCGGTGCTTCTTCTGCAGGTACCGTCACCACAAGCTTCGCCCCTGCTGAAAGCGGTTTACAACCCGAAGGCCGTCATCCCGCACGCGGCGTTGCTGCATCAGGCTTCCGCCCATTGTGCAATATTCCCCACTGCTGCCTCCCGTAGGAGTCTGGGCCGTATCTCAGTCCCAATGTGGCCGGTCGCCCTCTCAGGCCGGCTACCCGTCAAAGCCTTGGTAAGCCACTACCCCACCAACAAGCTGATAAGCCGCGAGTCCATCCAAAACCGCCGAAGCTTTCCAACCCCACCATGCAGCAAGGATTCCTATCCGGTATTAGCCCCAGTTTCCTGAAGTTATCCCGAAGTCAAAGGGCAGGTTACTCACGTGTTACTCACCCGTTCGCCA
>n.538.6.bb_711
GGAGTTAGCCGGTGCTTCTTCTGCGGGTAACGTCAATTGCTGAGGTTATTAACCTCAACACCTTCCTCCCCGCTGAAAGTACTTTACAACCCGAAGGCCTTCTTCATACACGCGGCATGGCTGCATCAGGCTTGCGCCCATTGTGCAATATTCCCCACTGCTGCCTCCCGTAGGAGTCTGGACCGTGTCTCAGTTCCAGTGTGGCTGGGTCATCCTCTCAGACCAGCTAGGGATCGTCGCCTAGGTGAGCCGTTACCCCACCTACTAGCTAATCCCATCTGGGCACATCTGATGGC
>n.538.6.bb_712
GTAGTTAGCCGTGGCTTTCTGATTAGGTACCGTCAAGACGTGCATAGTTACTTACACATTTGTTCTTCCCTAATAACAGAGTTTTACGATCCGAAGACCTTCATCACTCACGCGGCGTTGCTCCGTCAGGCTTTCGCCCATTGCGGAAGATTCCCTACTGCTGCCTCCCGTAGGAGTCTGGACCGTGTCTCAGTTCCAGTGTGGCCGATCACCCTCTCAGGGTCGGCTACGCATCGTTGCCTTGGTAAGCCGTTACCTTACCAACTAGCTAATGCGGCGCGGATCCATCTATAAGTGACAGCAAGACCGTCTTTCACTATTGAACCATGC
>n.538.6.bb_713
GTAGTTAGCCGTGGCTTTCTGATTAGGTACCGTCAAGACGTGCATAGTTACTTACACATTTGTTCTTCCCTAATAACAGAGTTTTACGATCCGAAGACCTTCATCACTCACGCGGCGTTGCTCCGTCAGGCTTTCGCCCATTGCGGAAGATTCCCTACTGCTGCCTCCCGTAGGAGTCTGGACCGTGTCTCAGTTCCAGTGTGGCCGATCACCCTCTCAGGGTCGGCTACGCATCGTCGCCTTGGTAAGCCGTTACCTTACCAACTAGCTAATGCGGCGCGGATCCATCTATAAGTGACAGCAAAACCGTCTTTCACTATTGAACCATGCGGTT
>n.538.6.bb_714
GTAGTTAGCCGTGGCTTTCTGATTAGGTACCGTCAAGACGTGCATAGTTACTTACACATTTGTTCTTCCCTAATAACAGAGTTTTACGATCCGAAGACCTTCATCACTCACGCGGCGTTGCTCCGTCAGGCTTTCGCCCATTGCGGAAGATTCCCTACTGCTGCCTCCCGTAGGAGTCTGGACCGTGTCTCAGTTCCAGTGTGGCCGATCACCCTCTCAGGGTCGGCTACGCATCGTCGCCTTGGTAAGCCGTCACCTTACCAACTAGCTAATGCGGCGCGGATCCATCTATAAGTGACAGCAAAACCGTCTTTCACTATTGAACCATGCGGTTCAATATATT
>n.538.6.bb_715
GTAGTTAGCCGTGGCTTTCTGATTAGGTACCGTCAAGACGTGCATAGTTACTTACACATTTGTTCTTCCCTAATAACAGAGTTTTACGATCCGAAGACCTTCATCACTCACGCGGCGTTGCTGCATCAGGCTTCCGCCCATTGTGCAATATTCCCCACTGCTGCCTCCCGTAGGAGTCTGGGCCGTATCTCAGTCCCAATGTGGCCGGGTCGCCCTCTCAGGCCGGCTACCCGTCAAAGCCTTGGTAAGCCACTACCCACCAACAAGCTGATAAGCCGCGAGTCCATCCAAAACCGCCGAA
>n.538.6.bb_716
GGAGTTAGCCGGTGCTTCTTCTGCGGGTAACGTCAATCGACAAGGTTATTAACCTTATCGCCTTCCTCCCCGCTGAAAGTACTTTACAACCCGAAGGCCTTCTTCATACACGCGGCATGGCTGCATCAGGCTTGCGCCCATTGTGCAATATTCCCCACTGCTGCCTCCCGTAGGAGTCTGGACCGTGTCTCAGTTCCAGTGTGGCTGGTCATCCTCTCAGACCAGCTAGGGATCGTCGCCTAGGTGAGCCGTTACCCACCTACTAGCTAATCCCATCTGGGCACATCTGATGGCAA
>n.538.6.bb_717
GGAGTTAGCCGGTGCTTCTTCTGCGGGTAACGTCAATCGACAAGGTTATTAACCTTATCGCCTTCCTCCCCGCTGAAAGTACTTTACAACCCGAAGGCCTTCTTCATACACGCGGCATGGCTGCATCAGGCTTGCGCCCATTGTGCAATATTCCCCACTGCTGCCTCCGTAGGAGTCTGGACCGTGTCTCAGTTCCAGTGTGGCTGGTCATCCTCTCAGACCAGCTAGGGATCGTC
>n.538.6.bb_718
GTAGTTAGCCGGTGCTTCTTCTGCAGGTACCGTCACCACAAGCTTCGCCCCTGCTGAAAGCGGTTTACAACCCGAAGGCCGTCATCCCGCACGCGGCGTTGCTGCATCAGGCTTCCGCCCATTGTGCAATATTCCCCACTGCTGCCTCCCGTAGGAGTCTGGGCCGTATCTCAGTCCCAATGTGGCCGGTCGCCCTCTCAGGCCGGCTACCCGGTCAAAGCCTTGGTAAGCCACTACCCCACCAACAAGCTGATAAGCCGCGAGTCCATCCAAAACCGCCGAAGCTTTTCCAACCCCCACCATGCAGCAAGGATTCCTATCCGGGTATTAGCCCCAGTTTCCTGAAGTTATCCCGAAGTCAAGGGCAGGTTACTCACGTGTACTCACCCGTTCGCCACTCGAGT
>n.538.6.bb_719
GTAGTTAGCCGTGGCTTTCTGATTAGGTACCGTCAAGACGTGCATAGTTACTTACACATTTGTTCTTCCCTAATAACAGAGTTTTACGATCCGAAGACCTTCATCACTCACGCGGCGTTGCTCCGTCAGGCTTTCGCCCATTGCGGAAGATTCCCTACTGCTGCCTCCCGTAGGAGTCTGGACCGTGTCTCAGTTCCAGTGTGGCCGATCACCCTCTCAGGGTCGGCTACGCATCGTTGCCTTGGTAAGCCGTTACCTTACCAACTAGCTAATGCGGCGCGGATCCATCTATAAGTGACAGCAAAACCGTCTTTCACTATTGAACCATGCGGTTCAATATATT
>n.538.6.bb_720
GTAGTTAGCCGTGGCTTTCTGATTAGGTACCGTCAAGACGTGCATAGTTACTTACACATTTATTCTTCCCTAATAACAGAGTTTTACGATCCGAAGACCTTCATCACTCACGCGGCGTTGCTCCGTCAGGCTTTCGCCCATTGCGGAAGATTCCCTACTGCTGCCTCCCGTAGGAGTCTGGACCGTGTCTCAGTTCCAGTGTGGCCGATCACCCTCTCAGGGTCGGCTACGCATCGTTGCCTTGGTAAGCCGTTACCTTACCAACTAGCTAATGCGGCGCGGATCTATCTATAAGTGACAGCAAAACCGTCTTTCACTATTGAACCATGCGGTTCAATATATTATCCGGTATTAGCTCCGG
>n.538.6.bb_721
GTAGTTAGCCGTGGCTTTCTGATTAGGTACCGTCAAGACGTGCATAGTTACTTACACATTTGTTCTTCCCTAATAACAGAGTTTTACGATCCGAAGACCTTCATCACTCACGCGGCGTTGCTCCGTCAGGCTTTCGCCCATTGCGGAAGATTCCCTACTGCTGCCTCCCGTAGGAGTCTGGACCGTGTCTCAGTTCCAGTGTGGCCGATCACCCTCTCAGGGTCGGCTACGCATCGTCGCCTTGGTAAGCCGTTACCTTACCGACTAGCTAATGCGGCGCGGATCCATCTATAAGTGACAGCAAAACCGTCTTTCACTATTGAACCATGCGGTTCAATATATTATCCGGTATT
>n.538.6.bb_722
GTAGTTAGCCGGTGCTTCTTCTGCAGGTACCGTCACCACAAGCTTCGCCCCTGCTGAAAGCGGTTTACAACCCGAAGGCCGTCATCCCGCACGCGGCGTTGCTGCATCAGGCTTCCGCCCATTGTGCAATATTCCCCACTGCTGCCTCCCGTAGGAGTCTGGGCCGTATCTCAGTCCCAATGTGGCCGGTCGCCCTCTCAGGCCGGCTACCCGGTCAAAGCCTTGGTAACGCCACTACCCCACCAACAAGCTGATAAGCCGCGAGTCCATCCAAAACCGCCGAAGCTTTCCAACCCCACCATGCAGCAAGGATTCCTATCCGGTATTACGCCCCAGTTTCCTGAAGTTATCCCGAAGTCAAGGGCAGGTTACTCACG
>n.538.6.bb_723
CGTGGCTTTCTGATTAGGTACCGTCAAGACGTGCATAGTTACTTACACATTTATTCTTCCCTAATAACAGGGTTTTACGATCCGAAGACCTTCATCACTCACGCGGCGTTGCTCCGTCAGGCTTTCGCCCATTGCGGAAGATTCCCTACTGCTGCCTCCCGTAGGAGTCTGGACCGTGTCTCAGTTCCAGTGTGGCCGATCACCCTCTCAGGTCGGCTACGCATCGTTGCCTTGGTAAGCCGTTACCTTACCAACTAGCTAATGCGGCGCGGATCCATCTATAAGTGACAGCAAAACCGTCTTTCACTATTGAACCATGCGGTTCAATATATTATCCGGTATT
>n.538.6.bb_724
GTAGTTAGCCGTGGCTTTCTGATTAGGTACCGTCAAGACGTGCATAGTTACTTACACATTTGTTCTTCCCTAATAACAGAGTTTTACGATCCGAAGACCTTCATCACTCACGCGGCGTTGCTCCGTCAGGCTTTCGCCCATTGCGGAAGATTCCCTACTGCTGCCTCCCGTAGGAGTCTGGACCGTGTCTCAGTTCCAGTGTGGCCGATCACCCTCTCAGGGTCGGCTACGCATCGTTGCCTTGGTAAGCCGTTACCTTACAACTAGCTAATGCGGCGCGGATCCATCTATAAGTGACAGCAAAACCGTCTTTCACTATTGAACCATGCGTAT
>n.538.6.bb_725
GTAGTTAGCCGGTGCTTCTTCTGCAGGTACCGTCACCACAAGCTTCGCCCCTGCTGAAAGCGGTTTACAACCCGAAGGCCGTCATCCCGCACGCGGCGTTGCTGCATCAGGCTTCCGCCCATTGTGCAATATTCCCCACTGCTGCCTCCCGTAGGAGTCTGGGCCGTATCTCAGTCCCAATGTGGCCGGTCGCCCTCTCAGGCCGGCTACCCGGTCAAAGCCTTGGTAAGCCACTACCCACCAACAAGCTGATAAGCCGCGAGTCCATCCAAAACCGCCGAAGCTTTCCAACCCCCACCATGCAGCAAGGATTCCTATCCGGTATTAGCCCCAGTTTCC
>n.538.6.bb_726
GTAGTTAGCCGTGGCTTTCTGATTAGGTACCGTCAAGACGTGCATAGTTACTTACACATTTGTTCTTCCCTAATAACAGAGTTTTACGATCCGAAGACCTTCATCACTCACGCGGCGTTGCTCCGTCAGCTTTCGCCCATTGCGGAAGATTCCCTACTGCTGCCTCCCGTAGGAGTCTGGACCGTGTCTCAGTTCCAGTGTGGCCGATCACCCTCTCAGGGTCGGCTACGCATCGTGCCTTGGTAAGCCGTTACCTTACCAACTAGCTAATGCGGCGCGGATCCATCTATAAGTGAC
>n.538.6.bb_727
GTAGTTAGCCGTGGCTTTCTGATTAGGTACCGTCAAGACGTGCATAGTTACTTACACATTTGTTCTTCCCTAATAACAGAGTTTTACGATCCGAAGACCTTCATCACTCACGCGGCGTTGCTCCGTCAGGCTTTCGCCCATTGCGGAAGATTCCCTACTGCTGCCTCCCGTAGGAGTCTGGACCGTGTCTCAGTTCCAGTGTGGCCGATCACCCTCTCAGGGTCGGCTACGCATCGTTGCCTTGGTAAGCCGTTACCTTACCAACTAGCTAATGCGGCGCGGATCCATCTATAAGTGACAGCAAAACCGTCT
>n.538.6.bb_728
GTAGTTAGCCGTGGCTTTCTGATTAGGTACCGTCAAGACGTGCATAGTTACTTACACATTTGTTCTTCCCTAATAACAGAGTTTTACGATCCGAAGACCTTCATCACTCACGCGGCGTTGCTCCGTCAAGCTTTCGCCCATTGCGGAAGATTCCCTACTGCTGCCTCCCGTAGGAGTCTGGACCGTGTCTCAGTTCCAGTGTGGCCGATCACCCTCTCAGGGTCGGCTACGCATCGTTGCCTTGGTAAGCCGTTACCTTACCAACTAGCTAATGCGGCGCGGATCCATCTATAAGTGACAGCAAAACCGTCTTTCACTATTGAACCATGCGGTTCAATATATATCCGGTATTAGC
>n.538.6.bb_729
GTAGTTAGCCGTGGCTTTCTGATTAGGTACCGTCAAGACGTGCATAGTTACTTACACATTTGTTCTTCCCTAATAACAGAGTTTTACGATCCGAAGACCTTCATCACTCACGCGGCGTTGCTCCGTCAGGCTTTCGCCCATTGCGGAAGATTCCCTACTGCTGCCTCCCGTAGGAGTCTGGACCGTGTCTCAGTTCCAGTGTGGCCGATCACCCTCTCAGGGTCGGCTACGCATCGTTGCCTTGGTAAGCCGTTACCTTACCAACTAGCTAATGCGGCGCGGATCCATCTATAAGTGACAGCAAAACCGTCTTTCACTATTGAACCATGCGGTTCAATATATTATCCGGTAT
>n.538.6.bb_730
GTAGTTAGCCGTGGCTTTCTGATTAGGTACCGTCAAGACGTGCATAGTTACTTACACATTTGTTCTTCCCTAATAACAGAGTTTTACGATCCGAAGACCTTCATCGCTCACGCGGCGTTGCTCCGTCAGGCTTTCGCCCATTGCGGAAGATTCCCTACTGCTGCCTCCCGTAGGAGTCTGGACCGTGTCTCAGTTCCAGTGTGGCCGATCACCCTCTCAGGGTCGGCTACGCATCGTTGCCTTGGTAAGCCGTTACCTTACCAACTAGCTAATGCGGCGCGGATCCATCTATAAGTGACAGCAAAACCGTCTTTCACTATTGAACC
>n.538.6.bb_731
GTAGTTAGCCGTGGCTTTCTGATTAGGTACCGTCAAGACGTGCATAGTTACTTACACATTTGTTCTTCCCTAATAACAGAGTTTTACGATCCGAAGACCTTCATCACTCACGCGGCGTTGCTCCGTCAGGCTTTCGCCCATTGCGGAAGATTCCCTACTGCTGCCTCCCGTAGGAGTCTGGACCGTGTCTCAGTTCCAGTGTGGCCGATCACCCTCTCAGGGTCGGCTACGCATCGTTGCCTTGGTAAGCCGTTACCTTACCAACTAGCTAATGCGGCGCGGATCCATCTATAAGTGACA
>n.538.6.bb_732
GGAGTTAGCCGGTGCTTCTTCTGCGGGTAACGTCAATCGATGAGGTTATTAACCTCACCGCCTTCCTCCCCGCTGAAAGTGCTTTACAACCCGAAGGCCTTCTTCACACACGCGGCATGGCTGCATCAGGCTTGCGCCCATTGTGCAATATTCCCCACTGCTGCCTCCCGTAGGAGTCTGGACCGTGTCTCAGTTCCAGTGTGGCTGGGTCATCCTCTCAGACCTGCTAGGGATCGTCGCCTAGGTG
>n.538.6.bb_733
GTAGTTAGCCGTGGCTTTCTGATTAGGTACCGTCAAGACGTGCATAGTTACTTACACATTTGTTCTTCCCTAATAACAGAGTTTTACGATCCGAAGACCTTCATCACTCACGCGGCGTTGCTCCGTCAGGCTTTCGCCCATTGCGGAAGATTCCCTACTGCTGCCTCCCGTAGGAGTCTGGACCGTGTCTCAGTTCCAGTGTGGCCGATCACCCTCTCAGGGTCGGCTACGCATCGTTGCCTTGGTAAGCCGTTACCTTACCAACTAGCTAATGCGGCGCGGATCCATCTATAAGT
>n.538.6.bb_734
GTAGTTAGCCGGTGCTTCTTCTGCAGGTACCGTCACCACAAGCTTCGCCCCTGCTGAAAGCGGTTTACAACCCGAAGGCCGTCATCCCGCACGCGGCGTTGCTGCATCAGGCTTCCGCCCATTGTGCAATATTCCCCACTGCTGCCTCCCGTAGGAGTCTGGGCCGTATCTCAGTCCCAATGTGGCCGGTCGCCCTCTCAGGCCCGGCTACCCGGTCAAAGCCTTGGTAACGCCACTACCCCACCAACAAGCTGATAAGCCGCGAGTCCATCCAAAACCGC
>n.538.6.bb_735
GTAGTTAGCCGTGGCTTTCTGATTAGGTACCGTCAAGACGTGCATAGTTACTTACACATTTGTTCTTCCCTAATAACAGAGTTTTACGATCCGAAGACCTTCATCACTCACGCGGCGTTGCTCCGTCAGGCTTTCGCCCATTGCGGAAGATTCCCTACTGCTGCCTCCCGTAGGAGTCTGGACCGTGTCTCAGTTCCAGTGTGGCCGATCACCCTCTCAGGGTCGGCTACGCATCGTTGCCTTGGTAAGCCGTTACCTTACCAACTAGCCAATGCGGCGCGGATCCATCTATAAGTGACAGCAAAACCGTCTTTCACTATTGAACCATGCGGTTCAATATATT
>n.538.6.bb_736
GTAGTTAGCCGTGGCTTTCTGATTAGGTACCGTCAAGACGTGCATAGTTACTTACACATTTGTTCTTCCCTAATAACAGAGTTTTACGATCCGAAGACCTTCATCACTCACGCGGCGTTGCTCCGTCAGGCTTTCGCCCATTGCGGAAGATTCCCTACTGCTGCCTCCCGTAGGAGTCTGGACCGTGTCTCAGTTCCAGTGTGGCCGATCACCCTCTCAGGGTCGGCTACGCATCGTTGCCTTGGTAAGCCGTTACCTTACCAACTAGCTAATGCGGCGCGGATCCATCTATAAGTGACAGCAAAACCGTCTTTCACTATTGAACCATGCGGTTCAATATATTATCCGGTATTAGCTCCGG
>n.538.6.bb_737
GGAGTTAGCCGGTGCTTCTTCTGCGGGTAACGTCAATTGCTGAGGTTATTAACCTCAACACCTTCCTCCCCGCTGAAAGTACTTTACAACCCGAAGGCCTTCTTCATACACGCGGCATGGCTGCATCAGGCTTGCGCCCATTGTGCAATATTCCCCACTGCTGCCTCCCGTAGGAGTCTGGACCGTGTCTCAGTTCCAGTGTGGCTGGGTCATCCTCTCAGACCAGCTAGGGATCGTCGCCTAGGTGACGCCGTTACCCACCTACTAGCTAATCCCATCTGGGCACATCT
>n.538.6.bb_738
GTAGTTAGTCGTGGCTTTCTGATTAGGTACCGTCAAGACGTGCATAGTTACTTACACATTTGTTCTTCCCTAATAACAGAGTTTTACGATCCGAAGACCTTCATCACTCACGCGGCGTTGCTCCGTCAGGCTTTCGCCCATTGCGGAAGATTCCCTACTGCTGCCTCCCGTAGGAGTCTGGACCGTGTCTCAGTTCCAGTGTGGCCGATCACCCTCTCAGGGTCGGCTACGCATCGTTGCCTTGGTAAGCCGTTACCTTACCACTAGCTAATGCGGCGCGGATCCATCTATAAGTGACAGCAAAACCGTCTTTCACTATTGAACCATGCGGTTCAATATATTATCCGGTATTAGCTCCGGT
>n.538.6.bb_739
GGAGTTAGCCGGTGCTTCTTCTGCGGGTAACGTCAATCGACAAGGTTATTAACCTTATCGCCTTCCTCCCCGCTGAAAGTACTTTACAACCCGAAGGCCTTCTTCATACACGCGGCATGGCTGCATCAGGCTTGCGCCCATTGTGCAATATTCCCCACTGCTGCCTCCCGTAGGAGTCTGGACCGTGTCTCAGTTCCAGTGTGGCTGGGTCATCCTCTCAGACCA
>n.538.6.bb_740
GGAGTTAGCCGGTGCTTCTTCTGCGGGTAACGTCAATCGACAAGGTTATTAACCTTATCGCCTTCCTCCCCGCTGAAAGTACTTTACAACCCGAAGGCCTTCTTCATACACGCGGCATGGCTGCATCAGGCTTGCGCCCATTGTGCAATATTCCCCACTGCTGCCTCCCGTAGGAGTCTGGACCGTGTCTCAGTTCCAGTGTGGCTGGTCATCCTCTCAGACCAGCTAGGGATCGTCGCCTAGGTGAGCCGTTACCCACCTACTAGCTAATCCCATCTGGGCACATCTGAT
>n.538.6.bb_741
GTAGTTAGCCGTGGCTTTCTGATTAGGTACCGTCAAGACGTGCATAGTTACTTACACATTTGTTCTTCCCTAATAACAGAGTTTTACGATCCGAAGACCTTCATCGCTCACGCGGCGTTGCTCCGTCAGGCTTTCGCCCATTGCGGAAGATTCCCTACTGCTGCCTCCCGTAGGAGTCTGGACCGTGTCTCAGTTCCAGTGTGGCCGATCACCCTCTCAGGGTCGGCTACGCATCGTTGCCTTGGTAAGCCGGCTACCTTACCAACTAGCTAATGCGGCGCGGATCCATCTATAAGTGACAGCAAAACCGTCTTTCACTATTGAACCATGCGGTTCAATATATT
>n.538.6.bb_742
GGAGTTAGCCGGTGCTTCTTCTGCGGGTAACGTCAATTGCTGAGGTTATTAACCTCAACACCTTCCTCCCCGCTGAAAGTACTTTACAACCCGAAGGCCTTCTTCATACACGCGGCATGGCTGCATCAGGCTTGCGCCCATTGTGCAATATTCCCCACTGCTGCCTCCCGTAGGAGTCTGGACCGTGTCTCAGTTCCAGTGTGGCTGGTCATCCTCTCAGACCAGCTAGGGATCGTCGCCTAGGTGAGCCGTTACCCACCTACTAGCTAATCCCATCTGGGCA
>n.538.6.bb_743
GAAGTTAGCCGGTGCTTCTTCTGCAGGTACCGTCACCACAAGCTTCGCCCCTGCTGAAAGCGGTTTACAACCCGAAGGCCGTCATCCCGCACGCGGCGTTGCTGCATCAGGCTTCCGCCCATTGTGCAATATTCCCCACTGCTGCCTCCCGTAGGAGTCTGGGCCGTATCTCAGTCCCAATGTGGCCGGTCGCCCTCTCAGGCCGGCTACCCGGTCAAAGCCTTGGTAAGCCACTACCCCACCAACAAGCTGATAAGCCGCGAGTCCATCCAAAACCGCC
>n.538.6.bb_744
GTAGTTAGCCGGTGCTTCTTCTGCAGGTACCGTCACCACAAGCTTCGCCCCTGCTGAAAGCGGTTTACAACCCGAAGGCCGTCATCCCGCACGCGGCGTTGCTGCATCAGGCTTCCGCCCATTGTGCAATATTCCCCACTGCTGCCTCCCGTAGGAGTCTGGGCCGTATCTCAGTCCCAATGTGGCCGTCGCCCTCTCAGGCCGGCTACCCGGTCAAAGCCTTGGTAACGCCACTACCCCACCAACAAGCTGATAAGCCGCGAGTCCATCC
>n.538.6.bb_745
GGAGTTAGCCGGTGCTTCTTCTGCGGGTAACGTCAATCGACAAGGTTATTAACCTTATCGCCTTCCTCCCCGCTGAAAGTACTTTACAACCCGAAGGCCTTCTTCATACACGCGGCATGGCTGCATCAGGCTTGCGCCCATTGTGCAATATTCCCCACTGCTGCCTCCGTAGGAGTCTGGACCGTGTCTCAGTCCCAGTGTGGCTGGTCATCCTCTCAGACCAGCTAGGGATCGTCGCCTAGGTGAGCCGTTACCCACCTACTAGCTAATCCCATCTGGGCACATCTGATGGC
>n.538.6.bb_746
GTAGTTAGCCGTGGCTTTCTGATTAGGTACCGTCAAGACGTGCATAGTTACTTACACATTTGTTCTTCCCTAATAACAGAGTTTTACGATCCGAAGACCTTCATCACTCACGCGGCGTTGCTCCGTCAGGCTTTCGCCCATTGCGGAAGATTCCCTACTGCTGCCTCCCGTAGGAGTCTGGACCGTGTCTCAGTTCCAGTGTGGCCGATCACCCTCTCAGGGTCGGCTACGCATCGTTGCCTTGGTAAGCCGTTACCTTACCAACTAGCTAATGCGGCGCGGATCCATCTATAAGTGACAGCAAAAACCGTCTTTCACTATTGAACCATGCGGTTCAATATATTATCCGGTATTAGCTCCGGT
>n.538.6.bb_747
GGAGTTAGCCGGTGCTTCTTCTGCGGGTAACGTCAATCGACAAGGTTATTAACCTTATCGCCTTCCTCCCCGCTGAAAGTACTTTACAACCCGAAGGCCTTCTCCATACACGCGGCATGGCTGCATCAGGCTTGCGCCCATTGTGCAATATTCCCCACTGCTGCCTCCGTAGGAGTCTGGACCGTGTCTCAGTTCCAGTGTGGCTGGTCATCCTCTCAGACCAGCTAGGGATCGTCGCTAGGTGAGCCGTTACCCACCTACTAGCTAATCCCATCTGGGCACATCTGATG
>n.538.6.bb_748
GTAGTTAGCCGTGGCTTTCTGATTAGGTACCGTCAAGACGTGCATAGTTACTTACACATTTGTTCTTCCCTAATAACAGAGTTTTACGATCCGAAGACCTTCATCACTCACGCGGCGTTGCTCCGTCAGGCTTTCGCCCATTGCGGAAGATTCCCTACTGCTGCCTCCCGTAGGAGTCTGGACCGTGTCTCAGTTCCAGTGTGGCCGATCACCCTCTCAGGGTCGGCTACGCATCGTTGCCTTGGTAAGCCGTTACCTTACCAACTAGCTAATGCGGCGCGGATCCATCTATAAGTGA
>n.538.6.bb_749
GTAGTTAGCCGTGGCTTTCTGATTAGGTACCGTCAAGACGTGCATAGTTACTTACACATTTGTTCTTCCCTAATAACAGAGTTTTACGATCCGAAGACCTTCATCACTCACGCGGCGTTGCTCCGTCAGGCTTTCGCCCATTGCGGAAGATTCCCTACTGCTGCCTCCCGTAGGAGTCTGGACCGTGTCTCAGTTCCAGTGTGGCCGATCACCCTCTCGAGGGTCGGCTACGCATCGTTGCCTTGGTAAGCCGTTACCTTACCAACTAGCTAATGCGGCGCGGATCCATCTATAAGTGACAGACAAAACCG
>n.538.6.bb_750
GTAGTTAGCCGTGGCTTTCTGATTAGGTACCGTCAAGACGTGCATAGTTACTTACACATTTGTTCTTCCCTAATAACAGAGTTTTACGATCCGAAGACCTTCATCACTCACGCGGCGTTGCTCCGTCAGGCTTTCGCCCATTGCGGAAGATTCCCTACTGCTGCCTCCCGTAGGAGTCTGGACCGTGTCTCAGTTCCAGTGTGGCCGATCACCCTCTCAGGGTCGGCTACGCATCGTTGCCTTGGTAAGCCGTTACCTTACCAACTAGCTAATGCGGCGCGGATCCATCTATAAGTGACAGCAAAACCGTCTTTCACT
>n.538.6.bb_751
GTAGTTAGCCGGTGCTTCTTCTGCAGGTACCGTCACCACAAGCTTCGCCCCTGCTGAAAGCGGTTTACAACCCGAAGGCCGTCATCCCGCACGCGGCGTTGCTGCATCAGGCTTCCGCCCATTGTGCAATATTCCCCACTGCTGCCTCCCGTAGGAGTCTGGGCCGTATCTCAGTCCCAATGTGGCCGGTCGCCCTCTCAGGCCGGCTACCCGGTCAAAGGCCTTGGGTAANCCACTACCCCACCAACAAGCTGATAAGCCGCGAGTCCATCCAAAACCGCCGAA
>n.538.6.bb_752
GTAGTTAGCCGGTGCTTCTTCTGCAGGTACCGTCACCACAAGCTTCGCCCCTGCTGAAAGCGGTTTACAACCCGAAGGCCGTCATCCCGCACGCGGCGTTGCTGCATCAGGCTTCCGCCCATTGTGCAATATTCCCCACTGCTGCCTCCCGTAGGAGTCTGGGCCGTATCTCAGTCCCAATGTGGCCGGTCGCCCTCTCAGGTCGGCTACGCATCGTTGCCTTGGTAAGCCGTTACCTTACCAACTAGCTAATGCGACGCGGATCCATCTATAAGTGACAGCAAAACCGTCTTTCACTATTGAACCATGCGGTTCAATATATTATCCGGTAT
>n.538.6.bb_753
GGAGTTAGCCGGTGCTTCTTCTGCGGGTAACGTCAATCGACAAGGTTATTAACCTTATCGCCTTCCTCCCCGCTGAAAGTACTTTACAACCCGAAGGCCTTCTTCATACACGCGGCATGGCTGCATCAGGCTTGCGCCCATTGTGCAATATTCCCCACTGCTGCCTCCCGTAGGAGTCTGGACCGTGTCTCAGTTCCAGTGTGGCTGGTCATCCTCTCAGACCAGCTAGGGATCGTCGCCTAGGTGAGCCGTTACCCACCTACTAGCTAATCCCATCTGGGCACATCTGATGGCAAGAGGCCCGAAGGTCCCCCTCTTTGGTCTTGCGACGTTATGCGGTATTAGCTACCGTTTCCAG
>n.538.6.bb_754
GTAGTTAGCCGGTGCTTCTTCTGCAGGTACCGTCACCACAAGCTTCGCCCCTGCTGAAAAGCGGTTTACAACCCGAAGGCCGTCATCCCGCACGCGGCGTTGCTGCATCAGGCTTCCGCCCATTGTGCAATATTCCCCACTGCTGCCTCCCGTAGGAGTCTGGGCCGTATCTCAGTCCCAATGTGGCCGGTCGCCCTCTCAGGCCGGCTACCCGTCAAAGCCTTGGTAAGCCACTACCCCACCAACAAGCTGATAAGCCGCGAGTCCATCCAAAACCGCCGAAGCTTTCCAACCCCACCATGCAGCAAGGATTCCTATCCGGTATTAGCCCCAGTTTCCTGAAGTTATCCCGAAGTCAAGGG
>n.538.6.bb_755
GTAGTTAGCCGTGGCTTCCTGATTAGGTACCGTCAAGACGTGCATAGTTACTTACACATTTATTCTTCCCTAATAACAGAGTTTTACGATCCGAAGACCTTCATCACTCACGCGGCGTTGCTCCGTCAGGCTTTCGCCCATTGCGGAAGATTCCCTACTGCTGCCTCCCATAGGAGTCTGGACCGTGTCTCAGTTCCAGTGTGGCCGATCACCCTCTCAGGTCGGCTACGCATCGTTGCCTTGGTAAGCCGTTACCTTACCAACTAGCTAATGCGGCGCGGATCCATCTATAAGTGACAGCAAAACCGTCTTTCACTATTGAACCATGCGGTTCAATATATTATCCGGTATTA
>n.538.6.bb_756
GTAGTTAGCCGTGGCTTTCTGATTAGGTACCGTCAAGACGTGCATAGTTACTTACACATTTGTTCTTCCCTAATAACAGAGTTTTACGATCCGAAGACCTTCATCACTCACGCGGCGTTGCTCCGTCAGGCTTTCGCCCATTGCGGAAGATTCCCTACTGCTGCCTCCCGTAGGAGTCTGGACCGTGTCTCAGTTCCAGTGTGGCCGATCACCCTCTCAGGGTCGGCTACGCATCGTTGCCTTGGTAAGCCGTTACCTTACCAACTAGCTAATGCGGCGCGGATCCATCTATAAGTGACAGCAAAACCGTCTTTCACTATTGAACCATGCGGTTCAATATATTATCCGGTATT
>n.538.6.bb_757
GTAGTTAGCCGTGGCTTTCTGATTAGGTACCGTCAAGACGTGCATAGTTACTTACACATTTGTTCTTCCCTAATAACAGAGTTTTACGATCCGAAGACCTTCATCACTCACGCGGCGTTGCTCCGTCAGGCTTTCGCCCATTGCGGAAGATTCCCTACTGCTGCCTCCCGTAGGAGTCTGGACCGTGTCTCAGTTCCAGTGTGGCCGATCACCCTNTCAGGGTCGGCTACGCATCGTTGCCTTGGTAAGCCGTTACCTTACCAACTAGCTAATGCGGCGCGGATCCATCTATAAGTGACAGCAAAACCGTCTTTCACTATTGAACCATGCGGTTCAATATATTATCCGGTATTAG
>n.538.6.bb_758
GGAGTTAGCCGGTGCTTCTTCTGCGGGTAACGTCAATTGCTGAGGTTATTAACCTCAACACCTTCCTCCCCGCTGAAAGTACTTTACAACCCGAAGGCCTTCTTCACACACGCGGCATGGCTGCATCAGGCTTGCGCCCATTGTGCAATATTCCCCACTGCTGCCTCCCGTAGGAGTCTGGACCGTGTCTCAGTTCCAGTGTGGCTGGGTCATCCTCTCAGACCAGCTAGGGATCGTCGCCTAGGTGAGCCGTTACCCCACCTACCAGCTAATCCCATCTGGGCACATCTGATGGCATGAGGCCCGAAGGTCCCCCACTTTGGTCTTGCGACGTTATGCGGTATTAGCTACCGTTTCCAGTAGTTATCCCCTCATCAGGCCAGTTTCCCCAGA
>n.538.6.bb_759
GTAGTTAGCCGTGGCTTTCTGATTAGGTACCGTCAAGACGTGCATAGTTACTTACACATTTGTTCTTCCCTAATAACAGAGTTTTACGATCCGAAGACCTTCATCACTCACGCGGCGTTGCTCCGTCAAGCTTTCGCCCATTGCGGAAGATTCCCTACTGCTGCCTCCCGTAGGAGTCTGGACCGTGTCTCAGTTCCAGTGTGGCCGATCACCCTCTCAGGGTCGGCTACGCATCGTTGCCTTGGTAAGCCGTTACCTTACCAACTAGCTAATGCGGCGCGGATCCATCTATAAGTGACAGCAAAACCGTCTTACACTATTGAACCA
>n.538.6.bb_760
GGAGTTAGCCGGTGCTTCTTCTGCGGGTAACGTCAATCGACAGGGTTATTAACCCTGTCGCCTTCCTCCCCGCTGAAGGTACTTTACAACCCGAAGGCCTTCTTCATACACGCGGCATGGCTGCATCAGGCTTGCGCCCATTGTGCAATATTCCCCACTGCTGCCTCCGTAGGAGTCTGGACCGTGTCTCAGTTCCAGTGTGGCTGGTCATCCTCTCAGACCAGCTAGGGATCGTCGCCTAGGTGAGCCGTTACCCACCTACTAGCTAATCCCATCTGGGCACATCCGATGG
>n.538.6.bb_761
GAGTTAGCCGGTGCTTCTTCTGCGGGTAACGTCAATCGACAAGGTTATTAACCTTATCGCCTTCCTCCCCGCTGAAAGTACTTTACAACCCGAAGGCCTTCTTCATACACGCGGCATGGCTGCATCAGGCTTGCGCCCATTGTGCAATATTCCCCACTGCTGCCTCCGTAGGAGTCTGGACCGTGTCTCAGTTCCAGTGTGGCTGGTCATCCTCTCAGACCAGCTAGGGATCGTCGCCTAGGTGAGCCGTTACCACCTACTAGCTAATCCCATCTGGGCACATCTGATGGC
>n.538.6.bb_762
GTAGTTAGCCGGTGCTTCTTCTGCAGGTACCGTCACCACAAGCTTCGCCCCTGCTGAAAGCGGTTTACAACCCGAAGGCCGTCATCCCGCACGCGGCGTTGCTGCATCAGGCTTCCGCCCATTGTGCAATATTCCCCACTGCTGCCTCCCGTAGGAGTCTGGGCCGTATCTCAGTCCCAATGTGGCCGGTCGCCCTCTCAGGCCGGCTACCCGGTCAAAGCCTTGGTAAGCCACTACCCCACCAACAAGCTGATAAGCCGCGAGTCCATCC
>n.538.6.bb_763
GTAGTTAGCCGGTGCTTCTTCTGCAGGTACCGTCACCACAAGCTTCGCCCCTGCTGAAAGCGGTTTACAACCCGAAGGCCGTCATCCCGCACGCGGCGTTGCTGCATCAGGCTTCCGCCCATTGTGCAATATTCCCCACTGCTGCCTCCCGTAGGAGTCTGGGCCGTATCTCAGTCCCAATGTGGCCGTCGCCCTCTCGGGCCGGCTACCCGGTCAAAGCCTTGGTAAGCCACTACCCACAACAAGCTGATAAGCCGCGAGTCCATCCAAAACCGCCG
>n.538.6.bb_764
GTAGTTAGCCGGTGCTTCTTCTGCAGGTACCGTCACCACAAGCTTCGCCCCTGCTGAAAGCGGTTTACAACCCGAAGGCCGTCATCCCGCACGCGGCGTTGCTGCATCAGGCTTCCGCCCATTGTGCAATATTCCCCACTGCTGCCTCCCGTAGGAGTCTGGGCCGTATCTCAGTCCCAATGTGGCCGGTCGCCCTCTCAGGCCGGCTACCCGTCAAAGCCTTGGTAAGCCACTACCCACCAACAAGCTGATAAGCCGCGAGTCCATCCAAAACCGCCGAAGCTTTTCCAACCCCCACCATGCAGCAAGGATTCCTATCCGGTATTAGCCCCAGTTTCCTGAAGTT
>n.538.6.bb_765
GTAGTTAGCCGTGGCTTTCTGATTAGGTACCGTCAAGACGTGCATAGTTACTTACACATTTGTTCTTCCCTAATAACAGAGTTTTACGATCCGAAGACCTTCATCACTCACGCGGCGTTGCTCCGTCAGGCTTTCGCCCATTGCGGAAGATTCCCTACTGCTGCCTCCCGTAGGAGTCTGGACCGTGTCTCAGTTCCAGTGTGGCCGATCACCCTCTCAGGGTCGGCTACGCATCGTTGCCTTGGTAAGCCGTTACCTTACCAACTAGCTAATGCGGCGCGGATCCATCTAT
>n.538.6.bb_766
GTAGTTAGCCGTGGCTTTCTGATTAGGTACCGTCAAGACGTGCATAGTTACTTACACATTTATTCTTCCCTAATAACAGAGTCTTACGATCCGAAGACCTTCATCACTCACGCGGCGTTGCTCCGTCAGGCTTTCGCCCATTGCGCAATATTCCCCACTGCTGCCTCCCGTAGGAGTTTGGACCGTGTCTCAGTTCCAATGTGGCCGATCACCCTCTCAGGTCGGCTACTGATCGTCGCCTTGGTAAGCCGTTACCTTACCAACTAGCTAATCAGACGCGGGTCCATCCTG
>n.538.6.bb_767
GTAGTTAGCCGTGGCTTTCTGATTAGGTACCGTCAAGACGTGCATAGTTACTTACACATTTGTTCTTCCCTAATAACAGAGTTTTACGATCCGAAGACCTTCATCACTCACGCGGCGTTGCTCCGTCAGGCTTTCGCCCATTGCGGAAGATTCCCTACTGCTGCCTCCCGTAGGAGTCTGGACCGTGTCTCAGTTCCAGTGTGGCCGATCACCCTCTCAGGGTCGGCTACGCATCGTTGCCTTGGTAAGCCGTTACCTTACCAACTAGCTAATGCGGCGCGGATCCATCTATAAGTGAC
>n.538.6.bb_768
GTAGTTAGCCGTGGCTTTCTGATTAGGTACCGTCAAGACGTGCATAGTTACTTACACATTTGTTCTTCCCTAATAACAGAGTTTTACGATCCGAAGACCTTCATCACTCACGCGGCGTTGCTCCGTCAGGCTTTCGCCCATTGCGGAAGATTCCCTACTGCTGCCTCCCGTAGGAGTCTGGACCGTGTCTCAGTTCCAGTGTGGCCGATCACCCTCTCAGGGTCGGCTACGCATCGTCGCCTTGGTAAGCCGTTACCTTACCAACTAGCTAATGCGGCGCGGATCCATCTATAAGTGACAACAAAACCGTCTTTCACTATTGAACCATGCGGTTCAAT
>n.538.6.bb_769
GTAGTTAGCCGTGGCTTTCTGATTAGGTACCGTCAAGACGTGCATAGTTACTTACACATTTGTTCTTCCCTAATAACAGAGTTTTACGATCCGAAGACCTTCATCACTCACGCGGCGTTGCTCCGTCAGGCTTTCGCCCATTGCGGAAGATTCCCTACTGCTGCCTCCCGTAGGAGTCTGGACCGTGTCTCAGTTCCAGTGTGGCCGATCACCCTCTCAGGGTCGGCTACGCATCGTTGCCTTGGTAAGCCGTTACCTTACCAACTAGCTAATGCGGCGCGGATCCATCTATAAGTGACAGCAAAACCGTCTTTCACTATTGAACCATGCGGTTCAATATATTATCCGGTATTAGCTCCGGTTTCCCGAAGTTATCCCAGTCTTATAGGTAGGTTATCCCACGTGTTACCTCACCCCGTCCCGCCCGCTAACGTCAGAGGGAGCAAGCTCCCTCGTCTG
>n.538.6.bb_770
GTAGTTAGCCGTGGCTTTCTGATTAGGTACCGTCAAGACGTGCATAGTTACTTACACATTTGTTCTTCCCTAATAACAGAGTTTTACGATCCGAAGACCTTCATCACTCACGCGGCGTTGCTCCGTCAGGCTTTCGCCCATTGCGGAAGATTCCCTACTGCTGCCTCCCGTAGGAGTCTGGACCGTGTCTCAGTTCCAGTGTGGCCGAT
>n.538.6.bb_771
GTAGTTAGCCGGTGCTTCTTCTGCAGGTACCGTCACCACAAGCTTCGCCCCTGCTGAAAGCGGTTTACAACCCGAAGGCCGTCATCCCGCACGCGGCGTTGCTGCATCAGGCTTCCGCCCATTGTGCAATATTCCCCACTGCTGCCTCCCGTAGGAGTCTGGGCCGTATCTCAGTCCCAATGTGGCCGGTCGCCCTCTCAGGCCGGCTACCCGTCAAAGCCTTGGTAAGCCACTACCCCACCAACAAGCTGATAAGCCGCGAGTCCATCCAAAACCGCCGAAGCTTTTCCAACCCCCACCATGCAGCAAGGATTCCTATCCGGTATTAGCCCCAGTTTCCTGAAGTTATCCCGAAGTCAAAGGGCAGGTTACTCACGTGTACTCACCCGTTCGCCA
>n.538.6.bb_772
GTAGTTAGCCGGTGCTTCTTCTGCAGGTACCGTCACCACAAGCTTCGCCCCTGCTGAAAGCGGTTTACAACCCGAAGGCCGTCATCCCGCACGCGGCGTTGCTGCATCAGGCTTCCGCCCATTGTGCAATATTCCCCACTGCTGCCTCCCGTAGGAGTCTGGGCCGTATCTCAGTCCCAATGTGGCCGGTCGCCCTCTCAGGCCGGCTACCCGTCAAAGCCTTGGTAAGCCACTACCCCACCAACAAGCTGATAAGCCGCGAGTCCATCCAAAACCGCCG
>n.538.6.bb_773
TAGTTAGCCGTGGCTTTCTGATTAGGTACCGTCAAGACGTGCATAGTTACTTACACATTTGTTCTTCCCTAATAACAGAGTTTTACGATCCGAAGACCTTCATCACTCACGCGGCGTTGCTCCGTCAGGCTTTCGCCCATTGCGGAAGATTCCCTACTGCTGCCTCCCGTAGGAGTCTGGACCGTGTCTCAGTTCCAGTGTGGCCCGATCACCCTCTCAGGGTCGGCTACGCATCGTTGCCTTGGTAAGCCGTTACCTTACCAACTAGCTAATGCGGCGCGGATCCATCTATAAAGTGACAGCAAAACCGTCTTTCACTATTGAACCATGCGGTTCAAT
>n.538.6.bb_774
GTAGTTAGCCGGTGCTTCTTCTGCAGGTACCGTCACCACAAGCTTCGCCCCTGCTGAAAGCGGTTTACAACCCGAAGGCCGTCATCCCGCACGCGGCGTTGCTGCATCAGGCTTCCGCCCATTGTGCAATATTCCCCACTGCTGCCTCCCGTAGGAGTCTGGGCCGTATCTCAGTCCCAATGTGGCCGGTCGCCCTCTCAGGCCGGCTAC
>n.538.6.bb_775
GGAGTTAGCCGGTGCTTCTTCTGCGGGTAACGTCAATCGACAAGGTTATTAACCTTATCGCCTTCCTCCCCGCTGAAAGTACTTTACAACCCGAAGGCCTTCTTCATACACGCGGCATGGCTGCATCAGGCTTGCGCCCATTGTGCAATATTCCCCACTGCTGCCTCCGTAGGAGTCTGGACCGTGTCTCAGTTCCAGTGTGGCTGGTCATCCTCTCAGACCAGCTA
>n.538.6.bb_776
GTAGTTAGCCGGTGCTTCTTCTGCAGGTACCGTCACCACAAGCTTCGCCCCTGCTGAAAGCGGTTTACAACCCGAAGGCCGTCATCCCGCACGCGGCGTTGCTGCATCAGGCTTCCGCCCATTGTGCAATATTCCCCACTGCTGCCTCCCGTAGGAGTCTGGGCCGTATCTCAGTCCCAGTGTGGCCGTCGCCCTCTCAGGCCGGCTACCCGTNAAAGCCTTGGTAAGCCACTACCCCACCAACAAGCTGATAAGCCGCGAGTCCATCCAAAACCG
>n.538.6.bb_777
GGAGTTAGCCGGTGCTTCTTCTGCGGGTAACGTCAATTGCTGAGGTTATTAACCTCAACACCTTCCTCCCCGCTGAAAGTACTTTACAACCCGAAGGCCTTCTTCATACACGCGGCATGGCTGCATCAGGCTTGCGCCCATTGTGCAATATTCCCCACTGCTGCCTCCCGTAGGAGTCTGGACCGTGTCTCAGTTCCAGTGTGGCTGGGTCATCCTCTCAGACCAGCTAGGGATCGTCGCCTAGGTGAGCCGTTACCCACCTACTAGCTAATCCCATCTGGGCACATCTGATG
>n.538.6.bb_778
GTAGTTAGCCGTGGCTTTCTGATTAGGTACCGTCAAGACGTGCATAGTTACTTACACATTTGTTCTTCCCTAATAACAGAGTTTTACGATCCGAAGACCTTCATCACTCACGCGGCGTTGCTCCGTCAGGCTTTCGCCCATTGCGGAAGATTCCCTACTGCTGCCTCCCGTAGGAGTCTGGACCGTGTCTCAGTTCCAGTGTGGCCGATCACCCTCTCAGGGTCGGCTACGCATCGTTGCCTTGGTAAGCCGTTACCTTACCAACTAGCTAATGCGGCGCGGATCCATCTATAAGTGACAGCAAAACCGTCTTTCACTATTGAACCA
>n.538.6.bb_779
TAGTTAGCCGTGGCTTTCTGATTAGGTACCGTCAAGACGTGCATAGTTACTTACACATTTGTTCTTCCCTAATAACAGAGTTTTACGATCCGAAGACCTTCATCACTCACGCGGCGTTGCTCCGTCAGGCTTTCGCCCATTGCGGAAGATTCCCTACTGCTGCCTCCCGTAGGAGTCTGGACCGTGTCTCAGTTTCAGTGTGGCCGATCACCCTCTCAGGGTCGGCTACGCATCGTCGCCTTGGTAAGCCGTTACCTTACCAACTAGCTAATGCGGCGCGGATCCATCTATAAGTGAC
>n.538.6.bb_780
GTAGTTAGCCGTGGCTTTCTGATTAGGTACCTTCAAGACGTGCATAGTTACTTACACATTTGTTCTTCCCTAATAACAGAGTTTTACGATCCGAAGACCTTCATCACTCACGCGGCGTTGCTCCGTCAGGCTTTCGCCCATTGCGGAAGATTCCCTACTGCTGCCTCCCGTAGGAGTCTGGACCGTGTCTCAGTTCCAGTGTGGCCGATCACCCTCTCAGGGTCGGCTACGCATCGTTGCCTTGGTAAGCCGTTACCTTACACTAGCTAATGCGGCGCGGATCCATCTATAAGTGACAGCAAAACCGTCTTTCACTAT
>n.538.6.bb_781
GAAGTTAGCCGGTGCTTCTTCTGCGGGTAACGTCAATTGCTGAGGTTATTAACCTCAACACCTTCCTCCCCGCTGAAAGTACTTTACAACCCGAAGGCCTTCTTCATACACGCGGCATGGCTGCATCAGGCTTGCGCCCATTGTGCAATATTCCCCACTGCTGCCTCCCGTAGGAGTCTGGACCGTGTCTCAGTTCCAGTGTGGCTGGGTCATCCTCTCAGACCAGCTAGGGATCGTCGCCTAGGTGAGCCGTTACCCACCTACTAGCTAATCCCATCTGGGCACATCTGAT
>n.538.6.bb_782
GGAGTTAGCCGGTGCTTCTTCTGCGGGTAACGTCAATCGACAAGGTTATTAACCTTATCGCCTTCCTCCCCGCTGAAAGTGCTTTACAACCCGAAGGCCTTCTTCACACACGCGGCATGGCTGCATCAGGCTTGCGCCCATTGTGCAATATTCCCCACTGCTGCCTCCCGTAGGAGTCTGGACCGTGTCTCAGTTCCAGTGTGGCTGGGTCATCCTCTCAGACCAGCTAGGGATCGTCGCCTAGGTGAGCCGTTACCCCACCTACCAGCTAATCCCATCTGGGCACATCTGATGGC
>n.538.6.bb_783
GTAGTTAGCCGGTGCTTCTTCTGCAGGTACCGTCACCACAAGCTTCGCCCCTGCTGAAAGCGGTTTACAACCCGAAGGCCGTCATCCCGCACGCGGCGTTGCTGCATCAGGCTTCCGCCCATTGTGCAATATTCCCCACTGCTGCCTCCCGTAGGAGTCTGGGCCGTATCTCAGTCCCAATGTGGCCGGTCGCCCTCTCAGGCCGGCTACCCGTCAAAGCCTTGGTAAGCCACTACCCCACCAACAAGCTGATAAGCCGCGAGTCCATCC
>n.538.6.bb_784
GTAGTTAGCCGGTGCTTCTTCTGCAGGTACCGTCACCACAAGCTTCGCCCCTGCTGAAAGCGGTTTACAACCCGAAGGCCGTCATCCCGCACGCGGCGTTGCTGCATCAGGCTTCCGCCCATTGTGCAATATTCCCCACTGCTGCCTCCCGTAGGAGTCTGGGCCGTATCTCAGTCCCAATGTGGCCGGTCGCCCTCTCAGGCCGGCTACCCGTCAAAGCCCCTGGTAAGCCACTACCCACCAACAAGCTGATAAGCCGCGAGTCCATCCAAAACCGC
>n.538.6.bb_785
GTAGTTAGCCGTGGCTTTCTGATTAGGTACCGTCAAGACGTGCATAGTTACTTACACATTTGTTCTTCCCTAATAACAGAGTTTTACGATCCGAAGACCTTCATCACTCACGCGGCGTTGCTCCGTCAGGCTTTCGCCCATTGCGGAAGATTCCCTACTGCTGCCTCCCGTAGGAGTCTGGACCGTGTCTCAGTTCCAGTGTGGCCGATCACCCTCTCAGGGTCGGCTACGCATCGTCGCCTTGGTAAGCCGTTACCTTACCAACTAGCTAATGCGGCGCGGATCCATCTATAAGTGACAGCAAAACCGTCTTTCACTATTGAACCATGCGGTTCAATATATTATCCGGTATT
>n.538.6.bb_786
GTAGTTAGCCGTGGCTTTCTGATTAGGTACCGTCAAGACGTGCATAGTTACTTACACATTTGTTCTTCCCTAATAACAGAGTTTTACGATCCGAAGACCTTCATCACTCACGCGGCGTTGCTCCGTCAGGCTTTCGCCCATTGCGGAAGATTCCCTACTGCTGCCTCCCGTAGGAGTCTGGACCGTGTCTCAGTTCCAGTGTGGCCGATCACCCTCTCAGGGTCGGCTACGCATCGTTGCCTTGGTAAGCCGTTACCTTACCAACTAGCTAATGCGGCGCGGATCCATCTATAAGTGACAGCAAAACCGTCTTTCACTATTGAACCACGCGGTTCAATATATTATCCGGTAT
>n.538.6.bb_787
GGAGTTAGCCGGTGCTTCTTCTGCGGGTAACGTCAATCGACAAGGTTATTAACCTTATCGCCTTCCTCCCCGCTGAAAGTACTTTACAACCCGAAGGCCTTCTTCATACACGCGGCATGGCTGCATCAGGCTTGCGCCCATTGTGCAATATTCCCCACTGCTGCCTCCCGTAGGAGTCTGGACCGTGTCTCAGTTCCAGTGTGGCTGGTCATCCTCTCAGACCAGCTAGGGATCGTCGCCTAGGTGAGCCGTTACCCACCTACTAGCTAATCCCATCTGGGCACATCTGATGG
>n.538.6.bb_788
GTAGTTAGCCGGTGCTTCTTCTGCAGGTACCGTCACCACAAGCTTCGCCCCTGCTGAAAGCGGTTTACAACCCGAAGGCCGTCATCCCGCACGCGGCGTTGCTGCATCAGGCTTCCGCCCATTGTGCAATATTCCCCACTGCTGCCTCCCGTAGGAGTCTGGGCCGTATCTCAGTCCCAATGTGGCCGGTCGCCCTCTCAGGCCGGCTACCCGGTCAAAGCCTTGGTAAGCCACTACCCACCAACAAGCTGATAAGCCGCGAGTCCATCCAAAACCGCCG
>n.538.6.bb_789
GTAGTTAGCCGGTGCTTCTTCTGCAGGTACCGCCACCACAAGCTTCGCCCCTGCTGAAAGCGGTTTACAACCCGAAGGCCGTCATCCCGCACGCGGCGTTGCTGCATCAGGCTTCCGCCCATTGTGCAATATTCCCCACTGCTGCCTCCCGTAGGAGTCTGGGCCGTATCTCAGTCCCAATGTGGCCGGTCGCCCTCTCAGGCCGGCTACCCGTCAAAGCCTTGGTAAGCCACTACCCCACCAACAAGCTGATAAGCCGCGAGTCCATCCAAAACCGCCGAAGCTTTTCCAACCCCACCATGCAGCAAGGATTCCTATCCGGTATTAGCCCCAGTTTCCTGAAGTTATCCCGAAGTCAAGGGCAGGTTACTCACGTGTTACC
>n.538.6.bb_790
GTAGTTAGCCGGTGCTTCTTCTGCAGGTACCGTCACCACAAGCTTCGCCCCTGCTGAAAGCGGTTTACAACCCGAAGGCCGTCATCCCGCACGCGGCGTTGCTGCATCAGGCTTCCGCCCATTGTGCAATATTCCCCACTGCTGCCTCCCGTAGGAGTCTGGGCCGTATCTCAGTCCCAATGTGGCCGGTCGCCCTCTCA
>n.538.6.bb_791
GGAGTTAGCCGGTGCTTCTTCTGCGGGTAACGTCAATTGCTGAGGTTATTAACCTCAACACCTTCCTCCCCGCTGAAAGTACTTTACAACCCGAAGGCCTTCTTCATACACGCGGCATGGCTGCATCAGGCTTGCGCCCATTGTGCAATATTCCCCACTGCTGCCTCCCGTAGGAGTCTGGACCGTGTCTCAGCTCCAGTGTGGCTGGGTCATCCTCTCAGACCAGCTAGGGATCGTCGCCTAGGTGAGCCGTTACCCACCTACTAGCTAATCCCATCTGGGCACATCTGATGGC
>n.538.6.bb_792
GTAGTTAGCCGTGGCTTTCTGATTAGGTACCGTCAAGACGTGCATAGTTACTTACACATTTATTCTTCCCTAATAACAGAGTTTTACGATCCGAAGACCTTCATCACTCACGCGGCGTTGCTCCGTCAGGCTTTCGCCCAGTGCGGAAGATTCCCTACTGCTGCCTCCCGTAGGAGTCTGGACCGTGTCTCAGTTCCAGTGTGGCCGATCACCCTCTCAGGGTCGGCTACGCATCGTTGCCTTGGTAAGCCGTTACCTTACCAACTAGCTAATGCGGCGCGGATCCATCTATAAGTGACAGCAAAACCGTCTTTCACTATTGAACCATGCGGTTCAATATATTATCCGGTATT
>n.538.6.bb_793
GTAGTTAGCCGTGGCTTTCTGATTAGGTACCGTCAAGACGTGCATAGTTACTTACACATTTGTTCTTCCCTAATAACAGAGTTTTACGATCCGAAGACCTTCATCACTCACGCGGCGTTGCTCCGTCAAGCTTTCGCCCATTGCGGAAGATTCCCTACTGCTGCCTCCCGTAGGAGTCTGGACCGTGTCTCAGTTCCAGTGTGGCCGATCACCCTCTCAGGGTCGGCTACGCATCGTTGCCTTGGTAAGCCGTTACCTTACCAACTAGCTAATGCGGCGCGGATCCATCTATAAGTGACAGCAAAACCGTCTTTCACTATTGAACCATGCGGTTCAATATATTATCCGGTATT
>n.538.6.bb_794
GTAGTTAGCCGTGGCTTTCTGATTAGGTACCGTCAAGACGTGCATAGTTACTTACACATTTGTTCTTCCCTAATAACAGAGTTTTACGATCCGAAGACCTTCATCACTCACGCGGCGTTGCTCCGTCAAGCTTTCGCCCATTGCGGAAGATTCCCTACTGCTGCCTCCGTAGGAGTCTGGACCGTGTCTCAGTTCCAGTGTGGCCGATCACCCTCTCAGGTCGCTACGCATCGTTGCCTTGGTAAGCCGTTACCTTACCAACTAGCTAATGCGGCGCGGATCCATCTATAAGTGAC
>n.538.6.bb_795
GTAGTTAGCCGGTGCTTCTTCTGCAGGTACCGTCACCACAAGCTTCGCCCCTGCTGAAAGCGGTTTACAACCCGAAGGCCGTCATCCCGCACGCGGCGTTGCTGCATCAGGCTTCCGCCCATTGTGCAATATTCCCCACTGCTGCCTCCCGTAGGAGTCTGGGCCGTATCTCAGTCCCAATGTGGCCGGTCGCCCTCTCAGGCCGGCTACCCGGTCAAAGCCTTGGTAAGCCACTACCCCACCAACAAGCTGATAAGCCGCGAGTCCATCCAAAACCGCCG
>n.538.6.bb_796
GTAGTTAGCCGTGGCTTTCTGATTAGGTACCGTCAAGACGTGCATAGTTACTTACACATTTGTTCTTCCCTAATAACAGAGTTTTACGATCCGAAGACCTTCATCACTCACGCGGCGTTGCTCCGTCAGGCTTTCGCCCATTGCGGAAGATTTCCTACTGCTGCCTCCCGTAGGAGTCTGGACCGTGTCTCAGTTCCAGTGTGGCCGATCACCCTCTCAGGGTCGGCTACGCATCGTTGCCTTGGTAAGCCGTTACCTTACCAACTAGCTAATGCGGCGCGGATCCATCTATAAGTGACAGCAAAACCGTCTTTCACTA
>n.538.6.bb_797
GTAGTTAGCCGTGGCTTTCTGATTAGGTACCGTCAAGACGTGCATAGTTACTTACACATTTGTTCTTCCCTAATAACAGAGTTTTACGATCCGAAGACCTTCATCACTCACGCGGCGTTGCTCCGTCAGGCTTTCGCCCATTGCGGAAGATTCCCTACTGCTGCCTCCCGTAGGAGTCTGGACCGTGTCTCAGTTCCAGTGTGGCCGATCACCCTCTCAGGGTCGGCTACGCATCGTTGCCTTGGTAAGCCGTTACCTTACCAACTAGCTAATGCGGCGCGGATCCATCTATAAGTGACAGCAAAACCGTCTTTCACTATTGAACCATGCGGTTCAATATATTATCCGGTAT
>n.538.6.bb_798
GTAGTTAGCCGTGGCTTTCTGATTAGGTACCGTCAAGACGTGCATAGTTACTTACACATTTGTTCTTCCCTAATAACAGAGTTTTACGATCCGAAGACCTTCATCACTCACGCGGCGTTGCTCCGTCAGGCTTTCGCCCATTGCGGAAGATTCCCTACTGCTGCCTCCCGTAGGAGTCTGGACCGTGTCTCAGTTCCAGTGTGGCCGATCACCCTCTCAGGGTCGGCTACGCATCGTTGCCTTGGTAAGCCGTTACCTTACAACTAGCTAATGCGGCGCGGATCCATCTATAAGTGAC
>n.538.6.bb_799
GGAGTTAGCCGGTGCTTCTTCTGCGGGTAACGTCAATTGCTGAGGTTATTAACCTCAACACCTTCCTCCCCGCTGAAAGTACTTTACAACCCGAAGGCCTTCTTCATACACGCGGCATGGCTGCATCAGGCTTGCGCCCATTGTGCAATATTCCCCACTGCTGCCTCCCGTAGGAGTCTGGACCGTGTCTCAGTTCCAGTGTGGCTGGGTCATCCTCTCAGACCAGCTAGGGATCGTCGCCTAGGTGAGCCGTTACCCACCTACTAGCT
>n.538.6.bb_800
GTAGTTAGCCGGTGCTTCTTCTGCAGGTACCGTCACCACAAGCTTCGCCCCTGCTGAAAGCGGTTTACAACCCGAAGGCCGTCATCCCGCACGCGGCGTTGCTGCATCAGGCTTCCGCCCATTGTGCAATATTCCCCACTGCTGCCTCCCGTAGGAGTCTGGGCCGTATCTCAGTCCCAATGTGGCCGGTCGCCCTCTCAGGCCGGCTACCCGTCAAAGCCTTGGTAAGCCACTACCCCACCAACAAGCTGATAAGCCGCGAGTCCATCCAAAACCGCCGAAGCTTTTCCAACCCCACCATGCAGCAAGGATTCCTATCCGGTATTAGCCCCAGTTTCCTGAAGTTATCCCGAAGTCAAGGGCAGGTTACTCAC
>n.538.6.bb_801
TAGTTAGCCGTGGCTTTCTGATTAGGTACCGTCAAGACGTGCATAGTTACTTACACATTTGTTCTTCCCTAATAACAGAGTTTTACGATCCGAAGACCTTCATCACTCACGCGGCGTTGCTCCGTCAGGCTTTCGCCCATTGCGGAAGATTCCCTACTGCTGCCTCCCGTAGGAGTCTGGACCGTGTCTCAGTTCCAGTGTGGCCGATCACCCTCTCAGGGTCGGGCTACGCATCGTTGCCTTGGTAAGCCGTTACCTTACCAACTAGCTAATGCGGCGCGGATCCATCTATAAGTGACAGCAAAACCGTCTTTCACTATTGAACCA
>n.538.6.bb_802
GTAGTTAGCCGTGGCTTTCTGATTAGGTACCGTCAAGACGTGCATAGTTACTTACACATTTGTTCTTCCCTAATAACAGAGTTTTACGATCCGAAGACCTTCATCACTCACGCGGCGTTGCTCCGTCAGGCTTTCGCCCATTGCGGAAGATTCCCTACTGCTGCCTCCCGTAGGAGTCTGGACCGTGTCTCAGTTCCAGTGTGGCCGATCACCCTCTCAGGGTCGGCTACGCATCGTCGCCTTGGTAAGCCGTTACCTTACCAACTAGCTAATGCGGCGCGGATCCATCTATAAGTGACAGCAAAACCGTCTTTCACTATTGAACCATGCGGTTCAATATATTATCCGGTATTAGCTCCGGTTTCCGAAGTTATCCCAGTCTTATAGGTAGGTTATCCCACGTGTTACCTCACCCCGTCCCGCCCGCTAACGTCAGAGGAGCAAGCTCCCTCGTCTGTTCGCTCGACTTG
>n.538.6.bb_803
GTAGTTAGCCGGTGCTTCTTCTGCAGGTACCGTCACCACAAGCTTCGCCCCTGCTGAAAGCGGTTTACAACCCGAAGGCCGTCATCCCGCACGCGGCGTTGCTGCATCAGGCTTCCGCCCATTGTGCAATATTCCCCACTGCTGCCTCCCGTAGGAGTCTGGGCCGTATCTCAGTCCCAATGTGGCCGTCGCCCTCTCAGGCCGGCTACCCGTCAAAGCCTTGGTAAGCCACTACCCCACCAACAAGCTGATAAGCCGCGAGTCCATCCAAAACCGCCGAAGCTTTCCAAACCCCCACCATGCAGCAAGGATTCCTATCCGGTATT
>n.538.6.bb_804
GGAGTTAGCCGGTGCTTCTTCTGCGGGTAACGTCAATTGCTGAGGTTATTAACCTCAACACCTTCCTCCCCGCTGAAAGTACTTTACAACCCGAAGGCCTTCTTCATACACGCGGCATGGCTGCATCAGGCTTGCGCCCATTGTGCAATATTCCCCACTGCTGCCTCCCGTAGGAGTCTGGACCGTGTCTCAGTTCCAGTGTGGCTGGGTCATCCTCTCAGACCAGCTAGGGATCGTCGCCTAGGTGACGCCGTT
>n.538.6.bb_805
GTAGTTAGCCGGTGCTTCTTCTGCAGGTACCGTCACCACAAGCTTCGCCCCTGCTGAAAGCGGTTTACAACCCGAAGGCCGTCATCCCGCACGCGGCGTTGCTGCATCAGGCTTCCGCCCATTGTGCAATATTCCCCACTGCTGCCTCCCGTAGGAGTCTGGGCCTATCTCAGTCCCAATGTGGCCGGTCGCCCTCTCAGGCCGGCTACCCGTNAAAGCCTTGGTAACGCCACTACCCCACCAACAAGCTGATAAGCCGCGAGTCCATCCAAAACCGCCGA
>n.538.6.bb_806
GTAGTTAGCCGTGGCTTTCTGATTAGGTACCGTCAAGACGTGCATAGTTACTTACACATTTGTTCTTCCCTAATAACAGAGTTTTACGATCCGAAGACCTTCATCACTCACGCGGCGTTGCTCCGTCAGGCTTTCGCCCATTGCGGAAGATTCCCTACTGCTGCCTCCCGTAGGAGTCTGGACCGTGTCTCAGTTCCAGTGTGGCCGATCACCCTCTCAGGGTCGGCTACGCATCGTTGCCTTGGTAAGCCGTTACCTTACCAACTAGCTAATGCGGCGCGGATCCATCTATAAGTGACAGCAAAACCGTCTTTCACTATTGAACCATGCGGTTCAATATATTATCCGGTATTAGCTCCGGTTTCCCGAAGTTATCCCAGTCTTATAGGTAGGTTATCCCACGTGTTACTCACCCCGTCCCGCCCGCTAACGTCAGAGGGAGCAAGCTCCCTCGTCTGTTCGCTCGACTTGCATGTATTAGGGCACGCCGCCAGCGTTCATCCTGAGCCA
>n.538.6.bb_807
GTAGTTAGCCGTGGCTTTCTGATTAGGTACCGTCAAGACGTGCATAGTTACTTACACATTTGTTCTTCCCTAATAACAGAGTTTTACGATCCGAAGACCTTCATCACTCACGCGGCGTTGCTCCGTCAGGCTTTCGCCCATTGCGGAAGATTCCCTACTGCTGCCTCCCGTAGGAGTCTGGACCGTGTCTCAGTTCCAGTGTGGCCGATCACCCTCTCAGGGTCGGCTACGCATCGTTGCCTTGGTAAGCCGTTACCTTACCAACTAGCTAATGCGGCGCGGATCCATCTATAAGTGA
>n.538.6.bb_808
GTAGTTAGCCGTGGCTTTCTGATTAGGTACCGTCAAGACGTGCATAGTTACTTACACATTTGTTCTTCCCTAATAACAGAGTTTTACGATCCGAAGACCTTCATCACTCACGCGGCGTTGCTCCGTCAGGCTTTCGCCCATTGCGGAAGATTCCCTACTGCTGCCTCCCGTAGGAGTCTGGACCGTGTCTCAGTTCCAGTGTGGCCGATCACCCTCTCAGGGTCGGCTACGCATCGTTGCCTTGGTAAGCCGTTACCTTACCAACTAGCTAATGCGGCGCGGATCCATCTATAAGTGACAGCAAAACCGTCTTTCACTATTGAACCATGCGGTTCAATATATTATCCGGTATTAGCTCCGGTTTCCGAAGTTATCCCAGTCTTATA
>n.538.6.bb_809
GTAGTTAGCCGGTGCTTCTTCTGCAGGTACCGTCACCACAAGCTTCGCCCCTGCCGAAAGCGGTTTACAACCCGAAGGCCGTCATCCCGCACGCGGCGTTGCTGCATCAGGCTTCCGCCCATTGTGCAATATTCCCCACTGCTGCCTCCCGTAGGAGTCTGGACCGTGTCTCAGTTCCAGTGTGGCTGGTCATCCTCTCAGACCAGCTAGGGGGATCGTCGCCTAGGTGAGCCGTTACCCCACCTACTAGCTAATCCCATCTGGGCACATCTGATGGCAAGAGGCCCGAAGGTCCCCCTCTTTGGTCTTGCGACGTTATGCGGTATTAGCTACCGTTTCCAG
>n.538.6.bb_810
GGAGTTAGCCGGTGCTTCTTCTGCGGGTAACGTCAATCGACAAGGTTATTAACCTCACNCCTTCCTCCCCGCTGAAAGTACTTTACAACCCGAAGGCCTTCTTCATACACGCGGCATGGCTGCATCAGGCTTGCGCCCATTGTGCAATATTCCCCACTGCTGCCTCCCGTAGGAGTCTGGACCGTGTCTCAGTTCCAGTGTGGCTGGGTCATCCTCTCAGACCAGCTAGGGATCGTCGCCTAGGTGAGCCGTTACCCACCTACTAGCTAATCCCATCTGGGCACATCTGATGGCAAGAGGCCCGAAGGTCCCCCTCTTTGGTCTTGCGACGTTATGCGGTATTAGCTACCGTTTCCAG
>n.538.6.bb_811
GTAGTTAGCCGTGGCTTTCTGATTAGGTACCGTCAAGACGTGCATAGTTACTTACACATTTGTTCTTCCCTAATAACAGAGTTTTACGATCCGAAGACCTTCATCACTCACGCGGCGTTGCTCCGTCAGGCTTTCGCCCATTGCGGAAGATTCCCTACTGCTGCCTCCCGTAGGAGTCTGGACCGTGTCTCAGTTCCAGTGTGGCCGATCACCCTCTCAGGGTCGGCTACGCATCGTTGCCTTGGTAAGCCGTTACCTTACCAACTAGCTAATGCGGCGCGGATCCATCTATAAGTGACAGCAAAACCGTCTTTCACTATTGAACCATGCGGTTCAATATATTATCCGGTATT
>n.538.6.bb_812
GTAGTTAGCCGTGGCTTTCTGATTAGGTACCGTCAAGACGTGCATAGTTACTTACACATTTGTTCTTCCCTAATAACAGAGTTTTACGATCCGAAGACCTTCATCACTCACGCGGCGTTGCTCCGTCAGGCTTTCGCCCATTGCGGAAGATTCCCTACTGCTGCCTCCCGTAGGAGTCTGGACCGTGTCTCAGTTCCAGTGTGGCCGATCACCCTCTCAGGGTCGGCTACGCATCGTTGCCTTGGTAAGCCGTTACCTTACCAACTAGCTAATGCGGCGCGGATCCATCT
>n.538.6.bb_813
GTAGTTAGCCGTGGCTTTCTGATTAGGTACCGTCAAGACGTGCATAGTTACTTACACATTTGTTCTTCCCTAATAACAGAGTTTTACGATCCGAAGACCTTCATCACTCACGCGGCGTTGCTCCGTCAGGCTTTCGCCCATTGCGGAAGATTCCCTACTGCTGCCTCCCGTAGGAGTCTGGACCGTGTCTCAGTTCCAGTGTGGCCGATCACCCTCTCAGGTCGGCTACGCATCGTTGCCTTGGTAAGCCGTTACCTTACCAACTAGCTAATGCGGCGCGGATCCATCTATAAGTGAC
>n.538.6.bb_814
GTAGTTAGCCGTGGCTTTCTGATTAGGTACCGTCAAGACGTGCATAGTTACTTACACATTTGTTCTTCCCTAATAACAGAGTTTTACGATCCGAAGACCTTCATCACTCACGCGGCGTTGCTCCGTCAGGCTTTCGCCCATTGCGGAAGATTCCCTACTGCTGCCTCCCGTAGGAGTCTGGACCGTGTCTCAGTTCCAGTGTGGCCGATCACCCTCTCAGGGTCGGCTACGCATCGTTGCCTTGGTAAGCCGTTACCTTACCAACTAGCTAATGCGACGCGGATCCATCTATAAGTGAC
>n.538.6.bb_815
GTAGTTAGCCGGTGCTTCTTCTGCAGGTACCGTCACCACAAGCTTCGCCCCTGCTGAAAGCGGTTTACAACCCGAAGGCCGTCATCCCGCACGCGGCGTTGCTGCATCAGGCTTCCGCCCATTGTGCAATATTCCCCACTGCTGCCTCCCGTAGGAGTCTGGGCCGTATCTCAGTCCCAATGTGGCCGGTCGCCCTCTCAGGCCGGCTACCCGGTCAAAGCCTTGGTAAGCCACTACCCCACCAACAAGCTGATAAGCCGCGAGTCCATCCAAAACCGCCGA
>n.538.6.bb_816
GTAGTTAGCCGTGGCTTTCTGATTAGGTACCGTCAAGACGTGCATAGTTACTTACACATTTGTTCTTCCCTAATAACAGAGTTTTACGATCCGAAGACCTTCATCACTCACGCGGCGTTGCTCCGTCAAGCTTTCGCCCATTGCGGAAGATTCCCTACTGCTGCCTCCCGTAGGAGTCTGGACCGTGTCTCAGTTCCAGTGTGGCCGATCACCCTCTCAGGGTCGGCTACGCATCGTTGCCTTGGTAAGCCGTTACCTTACCAACTAGCTAATGCGGCGCGGATCCATCTATAAGTGACAGCAAAACCGTCTTTCACTATTGAACCATGCGGTTCAATATATTATCCGGTATTAGCTCCGGTTTCCCGAAGTTATCCCAGTCTTATAGGTA
>n.538.6.bb_817
GTAGTTAGCCGTGGCTTTCTGATTAGGTACCGTCAAGACGTGCATAGTTACTTACACATTTGTTCTTCCCTAATAACAGAGTTTTACGATCCGAAGACCTTCATCACTCACGCGGCGTTGCTCCGTCAGGCTTTCGCCCATTGCGGAAGATTCCCTACTGCTGCCTCCGTAGGAGTCTGGACCGTGTCTCAGTTCCAGTGTGGCCGATCACCCTNTCAGGTCGGCTACGCATCGTTGCCTTGGTAAGCCGTTACCTTACCAACTAGCTAATGCGGCGCGGATCCATCTATAAGTGACAGCAAAACCGTCTTTCACTATTGAACCATGCGGTTCAATATATTATCCGGTATTAGCTCC
>n.538.6.bb_818
GTAGTTAGCCGTGGCTTTCTGATTAGGTACCGTCAAGACGTGCATAGTTACTTACACATTTGTTCTTCCCTAATAACAGAGTTTTACGATCCGAAGACCTTCATCACTCACGCGGCGTTGCTCCGTCAGGCTTTCGCCCATTGCGGAAGATTCCCTACTGCTGCCTCCCGTAGGAGTCTGGACCGTGTCTCAGTTCCAGTGTGGCCGATCACCCTCTCAGGGTCGGCTACGCATCGTCGCCTTGGTAAGCCGTTACCTTACCAAC
>n.538.6.bb_819
GTAGTTAGCCGTGGCTTTCTGATTAGGTACCGTCAAGACGTGCATAGTTACTTACACATTTGTTCTTCCCTAATAACAGAGTTTTACGATCCGAAGACCTTCATCACTCACGCGGCGTTGCTCCGTCAGGCTTTCGCCCATTGCGGAAGATTCCCTACTGCTGCCTCCCGTAGGAGTCTGGACCGTGTCTCAGTTCCAGTGTGGCCGATCACCCTCTCAGGGTCGGCTACGCATCGTTGCCTTGGTAAGCCGTTACCTTACCAACTAGCTAATGCGGCGCGGATCCATCTATAAGTGACAGCAAAAACCGTCTTTCACTATTGAACCATGCGGTTCAATATATTATCCGGTATTAGCTCCGGT
>n.538.6.bb_820
GGAGTTAGCCGTGGCTTTCTGATTAGGTACCGTCAAGACGTGCATAGTTACTTACACATTTGTTCTTCCCTAATAACAGAGTTTTACGATCCGAAGACCTTCATCACTCACGCGGCGTTGCTCCGTCAGGCTTTCGCCCATTGCGGAAGATTCCCTACTGCTGCCTCCCGTAGGAGTCTGGACCGTGTCTCAGTTCCAGTGTGGCCGATCACCCTCTCAGGGTCGGCTACGCATCGTTGCCTTGGTAAGCCGTTACCTTACCAACTAGCTAATGCGGCGCGGATCCATCTATAAGTGACAGCAAAACCGTCTTTCACTATTGAACCATGCGGTTCAATATATTATCCGGTATT
>n.538.6.bb_821
GTAGTTAGCCGGTGCTTCTTCTGCAGGTACCGTCACCACAAGCTTCGCCCCTGCTGAAAGCGGTTTACAACCCGAAGGCCGTCATCCCGCACGCGGCGTTGCTGCATCAGGCTTCCGCCCATTGTGCAATATTCCCCACTGCTGCCTCCCGTAGGAGTCTGGGCCGTATCTCAGTCCCAATGTGGCCGGTCGCCCTCTCAGGCCGGCTACCCGTCAAAGCCTTGGTAAGCCACTACCCCACCAACAAGCTGATAAGCCGCGAGTCCATCCAAAACCGCCGAAGCCTTTCCCAACCCCCACCATGCAGCAAGGATTCCTATCCGGTATTACGCCCCTAGTTT
>n.538.6.bb_822
GTAGTTAGCCGTGGCTTTCTGATTAGGTACCGTCAAGACGTGCATAGTTACTTACACATTTATTCTTCCCTAATAACAGAGTTTTACGATCCGAAGACCTTCATCACTCACGCGGCGTTGCTCCGTCAGGCTTTCGCCCATTGCGGAAGATTCCCTACTGCTGCCTCCCGTAGGAGTCTGGACCGTGTCTCAGTTCCAGTGTGGCCGATCACCCTCTCAGGGTCGGCTACGCATCGTTGCCTTGGTAAGCCGTTACCTTACCAACTAGCTAATGCGGCGCGGATCCATCTATAAGTGGCAGCAAAACCGTCTTT
>n.538.6.bb_823
AGTTAGCCGGTGCTTCCTCTGCAGGTACCGTCACCACAAGCTTCGCCCCTGCTGAAAGCGGTTTACAACCCGAAGGCCGTCATCCCGCACGCGGCGTTGCTGCATCAGGCTTCCGCCCATTGTGCAATATTCCCCACTGCTGCCTCCCGTAGGAGTCTGGGCCGTATCTCAGTCCCAATGTGGCCGGTCGCCCTCTCAGGCCGGCTACCCGTCAAAGCCTTGGTAAGCCACTACCCCACCAACAAGCTGATAAGCCGCGAGTCCATCCAAAACCGC
>n.538.6.bb_824
GTGGTTAGCCGTGGCTTTCTGATTAGGTACCGTCAAGACGTGCATAGTTACTTACACATTTGTTCTTCCCTAATAACAGAGTTTTACGATCCGAAGACCTTCATCACTCACGCGGCGTTGCTCCGTCAGGCTTTCGCCCATTGCGGAAGATTCCCTACTGCTGCCTCCCGTAGGAGTCTGGACCGTGTCTCAGTTCCAGTGTGGCCGATCACCCTCTCAGGGTCGGCTACGCATCGTTGCCTTGGTAAGCCGTTACCTTACCAACTAGCTAATGCGGCGCGGATCCATCTATAAGTGACAGCAAAACCGTCTTTCACTATTGAACCATGCGGTTCAATATATTATCCGGTATTAGCTCCGGT
>n.538.6.bb_825
GGAGTTAGCCGGTGCTTCTTCTGCGGGTAACGTCAATCGACAGGGTTATTAACCCTGTCGCCTTCCTCCCCGCTGAAAGTACTTTACAACCCGAAGGCCTTCTTCATACACGCGGCATGGCTGCATCAGGCTTGCGCCCATTGTGCAATATTCCCCACTGCTGCCTCCCGTAGGAGTCTGGACCGTGTCTCAGTTCCAGTGTGGCTGGTCATCCTCTCAGACCAGCTAGGGATCGTCGCCTAGGTGAGCCGTTACCCACCTACTAGCTAATCCCATCTGGGCA
>n.538.6.bb_826
GTAGTTAGCCGGTGCTTCTTCTGCAGGTACCGTCACCACAAGCTTCGCCCCTGCTGAAAGCGGTTTACAACCCGAAGGCCGTCATCCCGCACGCGGCGTTGCTCCGTCAGGCTTTCGCCCATTGCGGAAGGTTCCCTACTGCTGCCTCCCGTAGGAGTCTGGACCGTGTCTCAGTTCCAGTGTGGCCGATCACCCTNTCAGGTCGGCTACGCATCGTTGCCTTGGTAANCCGTTACTACAACTAGCTAATGCGGCGCGGATCCATCTATAAGTGACAGC
>n.538.6.bb_827
GTAGTTAGCCGGTGCTTCTTCTGCAGGTACCGTCACCACAAGCTTCGCCCCTGCTGAAAGCGGTTTACAACCCGAAGGCCGTCATCCCGCACGCGGCGTTGCTGCATCAGGCTTCCGCCCATTGTGCAATATTCCCCACTGCTGCCTCCCGTAGGAGTCTGGGCCGTATCTCAGTCCCAATGTGGCCGGTCGCCCTCTCAGGCCGGCTACCCGTCAAAGCCTTGGTAAGCCACTACCCCACCAACAAGCTGATAAGCCGCGAGTCCATCCAAAACCGCCG
>n.538.6.bb_828
GTAGTTAGCCGTGGCTTTCTGATTAGGTACCGTCAAGACGTGCATAGTTACTTACACATTTGTTCTTCCCTAATAACAGAGTTTTACGATCCGAAGACCTTCATCACTCACGCGGCGTTGCTCCGTCAGGCTTTCGCCCATTGCGGAAGATTCCCTACTGCTGCCTCCCGTAGGAGTCTGGACCGTGTCTCAGTTCCAGTGTGGCCGATCACCCTCTCAGGGTCGGCTACGCATCATCGCCTTGGTAAGCCGTTACCTTACCAACTAGCTAATGCGGCGCGGATCCATCTATAAGTGACAGCAAAACCGTCTTTCACTATTGAACCATGCGGTTCAATATATTATCCGGTATTAGCTCCGGTTTCCCGAAGTTACTCCCAGT
>n.538.6.bb_829
GTAGTTAGCCGGTGCTTCTTCTGCAGGTACCGTCACCACAAGCTTCGCCCCTGCTGAAAGCGGTTTACAACCCGAAGGCCGTCATCCCGCACGCGGCGTTGCTGCATCAGGCTTCCGCCCATTGTGCAATATTCCCCACTGCTGCCTCCCGTAGGAGTCTGGGCCGTATCTCAGTCCCAATGTGGCCGATCACCCTCTCAGGTCGGCTACGCATCGTTGCCTTGGTAAGCCGTTACCTTACCAACTAGCTAATGCGACGCGGATCCATCTATAAGTGACAGC
>n.538.6.bb_830
GTAGTTAGCCGTGGCTTTCTGATTAGGTACCGTCAAGACGTGCATAGTTACTTACACATTTGTTCTTCCCTAATAACAGAGTTTTACGATCCGAAGACCTTCATCACTCACGCGGCGTTGCTCCGTCAGGCTTTCGCCCATTGCGGAAGATTCCCTACTGCTGCCTCCCGTAGGAGTCTGGACCGTGTCTCAGTTCCAGTGTGGCCGATCACCCTCTCAGGGTCGGCTACGCATCGTTGCCTTGGTAAGCCGTTACCTTACCAACTAGCTAATGCGGCGCGGATCCATCTATAAGTGACAGCAAAACCGTCTTTCACTATTGAACCATGCGGTTCAATATATATCCGGTATTAGCTCCGGT
>n.538.6.bb_831
GTAGTTAGCCGTGGCTTTCTGATTAGGTACCGTCAAGACGTGCATAGTTACTTACACATTTGTTCTTCCCTAATAACAGAGTTTTACGATCCGAAGACCTTCATCACTCACGCGGCGTTGCTCCGTCAGGCTTTCGCCCATTGCGGAAGATTCCCTACTGCTGCCTCCCGTAGGAGTCTGGACCGTGTCTCAGTTCCAGTGTGGCCGATCACCCTCTCAGGGTCGGCTACGCATCGTTGCCTTGGTAAGCCGTTACCTTACCAACTAGCTAATGCGGCGCGGAT
>n.538.6.bb_832
GTAGTTAGCCGTGGCTTTCTGATTAGGTACCGTCAAGACGTGCATAGTTACTTACACATTTGTTCTTCCCTAATAACAGAGTTTTACGATCCGAAGACCTTCATCACTCACGCGGCGTTGCTCCGTCAGGCTTTCGCCCATTGCGGAAGATTCCCTACTGCTGCCTCCCGTAGGAGTCTGGACCGTGTCTCAGTTCCAGTGTGGCCGATCACCCTCTCAGGGTCGGCTACGCATCGTTGCCTTGGTAAGCCGTTACCTTACCAACTAGCTAATGCGGCGCGGATCCATCTATAAGTGACAGCAAAACCGTCTTTCACTATTGAACCATGCGGTTCAATATATTATCCGGTATTAGCTCCGGT
>n.538.6.bb_833
GTAGTTAGCCGGTGCTTCTTCTGCAGGTACCGTCACCACAAGCTTCGCCCCTGCTGAAAGCGGTTTACAACCCGAAGGCCGTCATCCCGCACGCGGCGTTGCTGCATCAGGCTTCCGCCCATTGTGCAATATTCCCCACTGCTGCCTCCCGTAGGAGTCTGGGCCGTATCTCAGTCCCAATGTGGCCGGTCGCCCTCTCAGGCCGGCTACCCGGTNAAAGCCTTGGTAAGCCACTACCCCACCAACAAGCTGATAAGCCGCGAGTCCATCCAAAACCGCCGAAGCTTTCCAACCCCCACCATGCAGCAAGGATTCCTATCCGGTATTAGCCCCAGTTTCCTGAAGTTATCCCGAAGTCAAGGGCAGGTTACTCACGTGTTACTCACCCGTTCGCCACT
>n.538.6.bb_834
TAGTTAGCCGTGGCTTTCTGATTAGGTACCGTCAAGACGTGCATAGTTACTTACACATTTGTTCTTCCCTAATAACAGAGTTTTACGATCCGAAGACCTTCATCACTCACGCGGCGTTGCTCCGTCAGGCTTTCGCCCATTGCGGAAGATTCCCTACTGCTGCCTCCCGTAGGAGTCTGGACCGTGTCTCAGTTCCAGTGTGGCCGATCACCCTCTCAGGGGTCGGCTACGCATCGTTGCCTTGGTAAGCCGTTACCTTACCAACTAGCTAATGCGACGCGGATCCATCTATAAGTGACAGCAAAACCGTCTTTCACT
>n.538.6.bb_835
GTAGTTAGCCGGTGCTTCTTCTGCAGGTACCGTCACCACAAGCTTCGCCCCTGCTGAAAGCGGTTTACAACCCGAAGGCCGTCATCCCGCACGCGGCGTTGCTGCATCAGGCTTCCGCCCATTGTGCAATATTCCCCACTGCTGCCTCCCGTAGGAGTCTGGGCCGTATCTCAGTCCCAATGTGGCCGGTCGCCCTCTCAGGCCGGCTACCCGTCAAAGCCTTGGTAAGCCACTACCCCACCAACAAGCTGATAAGCCGCGAGTCCATCCAAAACCGCCGAAGCTTTTCCAACCCCCACCATGCAGCAAGGATTCCTATCCGGTATTAGCCCCAGTTTCCTGAAGTTATCCCGAAGTCAAAGGGCAGGTTACTCACGTGT
>n.538.6.bb_836
GTAGTTAGCCGTGGCTTTCTGTATTAGGTACCGTCAAGACGTGCATAGTTACTTACACATTTAGTCCTTCCCTAATAACAGAGTTTTACGATCCGAAGACCTTCATCACTCACGCGGCGTTGCTCCGTCAGGCTTTCGCCATTGCGGAAGATTCCCTACTGCTGCCTCCCGTAGGAGTCTGGACCGTGTCTCAGTTCCAGTGTGGCCGATCAC
>n.538.6.bb_837
GTAGTTAGCCGTGGCTTTCTGATTAGGTACCGTCAAGACGTGCATAGTTACTTACACATTTGTTCTTCCCTAATAACAGAGTTTTACGATCCGAAGACCTTCATCACTCACGCGGCGTTGCTCCGTCAGGCTTTCGCCCATTGCGGAAGATTCCCTACTGCTGCCTCCCGTAGGAGTCTGGACCGTGTCTCAGTTCCAGTGTGGCCAT
>n.538.6.bb_838
GTAGTTAGCCGGTGCTTCTTCTGCAGGTACCGTCACCACAAGCTTCGCCCCTGCTGAAAGCGGTTTACAACCCGAAGGCCGTCATCCCGCACGCGGCGTTGCTGCATCAGGCTTCCGCCCATTGTGCAATATTCCCCACTGCTGCCTCCCGTAGGAGTCTGGGCCGTATCTCAGTCCCAATGTGGCCGGTCGCCCTCTCAGG
>n.538.6.bb_839
GTAGTTAGCCGTGGCTTTCTGATTAGGTACCGTCAAGACGTGCATAGTTACTTACACATTTGTTCTTCCCTAATAACAGAGTTTTACGATCCGAAGACCTTCATCACTCACGCGGCGTTGCTGCATCAGGCTTCCGCCCATTGTGCAATATTCCCCACTGCTGCCTCCCGTAGGAGTCTGGGCCGTATCTCAGTCCCAATGTGGCCGGTCGCCCTCTCAGGGCCGGCTACCCGGTCAAAGCCTTGGTAAGCCACTACCCCACCAACAAGCTGATAAGCCGCGAGTCCATCCAAAACCGCCGAAGCTTTCCAACCCCACCATGCAGCAAGGATTCCTATCCG
>n.538.6.bb_840
GTAGTTAGCCGGTTGCTTCTTCTGCAGGTACCGTCACCACAAGCTTCGCCCCTGCTGAAAGCGGTTTACAACCCGAAGGCCGTCATCCCGCACGCGGCGTTGCTGCATCAGGCTTCCGCCCATTGTGCAATATTCCCCACTGCTGCCTCCCGTGGGAGTCTGGGCCGTATCTCAGTCCCAATGTGGCCGGTCGCCCTCTCAGGCCGGCTACCCGTCAAAGCCTTGGTAACGCCACTACCCACCAACAAGCTGATAAGCCGCGAGTCCATCCAAAACCGC
>n.538.6.bb_841
GTAGTTAGCCGTGGCTTTCTGATTAGGTACCGTCAAGACGTGCATAGTTACTTACACATTTATTCTTCCCTAATAACAGAGTTTTACGATCCGAAGACCTTCATCACTCACGCGGCGTTGCTCCGTCAGGCTTTCGCCCATTGCGGAAGATTCCCTACTGCTGCCTCCCGTAGGAGTCTGGACCGTGTCTCAGTTCCAGGTGTGGCCGATCCACCCTCTCGAGGGTCGGCTACGCATCGTTGCCTTGGTAAGCCGTTACCTTACCAACTAGCTAATGCGGCGCGGATCCATCTAT
>n.538.6.bb_842
GTAGTTAGCCGTGGCTTTCTGATTAGGTACCGTCAAGACGTGCATAGTTACTTACACATTTGTTCTTCCCTAATAACAGAGTTTTACGATCCGAAGACCTTCATCACTCACGCGGCGTTGCTCCGTCAGGCTTTCGCCCATTGCGGAAGATTCCCTACTGCTGCCTCCCGTAGGAGTCTGGACCGTGTCTCAGTTCCAGTGTGGCCGATCACCCTCTCAGGGTCGGCTACGCATCGTTGCCTTGGTAAGCCGTTACCTTACCAACTAGCTAATGCGGCGCGGATCCATCTATAAGTGACAGCAAAACCGTCTTTCACTATTGAACCATGCGGTTCAATATATTATCCGGTATTAGCTCCGGTTT
>n.538.6.bb_843
TAGTTAGCCGGTGCTTCTTCTGCAGGTACCGTCACCACAAGCTTCGCCCCTGCTGAAAGCGGTTTACAACCCGAAGGCCGTCATCCCGCACGCGGCGTTGCTGCATCAGGCTTCCGCCCATTGTGCAATATTCCCCACTGCTGCCTCCCGTAGGAGTCTGGGCCGTATCTCAGTCCCAATGTGGCCGGTCGCCCTCTCAGGCCGGCTACCCGTCAAAGCCTTGGTAAGCCACTACCCACCAACAAGCTGATAAGCCGCGAGTCCATCCAAAACCGC
>n.538.6.bb_844
GTAGTTAGCCGTGGCTTTCTGATTAGGTACCGTCAGACGTGCATAGTTACTTACACATTTGTTCTTCCCTAATAACAGAGTTTTACGATCCGAAGACCTTCATCACTCACGCGGCGTTGCTCCGTCAGGCTTTCGCCCATTGCGGAAGATTCCCTACTGCTGCCTCCCGTAGGAGTCTGGACCGTGTCTCAGTTCCAGTGTGGCCGATCCACCCTCTCAGGGTCGGCTACGCATCGTTGCC
>n.538.6.bb_845
GTAGTTAGCCGTGGCTTTCTGATTAGGTACCGTCAAGACGTGCATAGTTACTTACACATTTGTTCTTCCCTAATAACAGAGTTTTACGATCCGAAGACCTTCATCACTCACGCGGCGTTGCTCCGTCAGGCTTTCGCCCATTGCGGAAGATTCCCTACTGCTGCCTCCCGTAGGAGTCTGGGCCGTATCTCAGTCCCAAT
>n.538.6.bb_846
GTAGTTAGCCGTGGCTTTCTGATTAGGTACCGTCAAGACGTGCATAGTTACTTACACATTTGTTCTTCCCTAATAACAGAGTTTTACGATCCGAAGACCTTCATCACTCACGCGGCGTTGCTCCGTCAGGCTTTCGCCCATTGCGGAAGATTCCCTACTGCTGCCTCCCGTAGGAGTCTGGACCGTGTCTCAGTTCCAGTGTGGCCGATCACCCTCTCAGGGTCGGCTACGCATCGTTGCCTTGGTAAGCCGTTACCTTACCAACTAGCTAATGCGACGCGGATCCATCTATAAGTGACAGCAAAACCGTCTTTCACTATTGAACCATGCGGTTCAATATATTATCCGGTATTAGCTCCGGT
>n.538.6.bb_847
GTAGTTAGCCGGTGCTTCTTCTGCAGGTACCGTCACCACAAGCTTCGCCCCTGCTGAAAGCGGTTTACAACCCGAAGGCCGTCATCCCGCACGCGGCGTTGCTGCATCAGGCTTCCGCCCATTGTGCAATATTCCCCACTGCTGCCTCCCGTAGGAGTCTGGGCCGTATCTCAGTCCCAATGTGGCCGGTCGCCCTCTCAG
>n.538.6.bb_848
GTAGTTAGCCGGTGCTTCTTCTGCAGGTACCGTCACCACAAGCTTCGCCCCTGCTGAAAGCGGTTTACAACCCGAAGGCCGTCATCCCGCACGCGGCGTTGCTGCATCAGGCTTCCGCCCATTGTGCAATATTCCCCACTGCTGCCTCCCGTAGGAGTCTGGGCCGTATCTCAGTCCCAATGTGGCCGGTCGCCCTCTCAGGCCGGCTACCCGGTCAAAGGCCCTTGGTAAAGCCACTACCCCACCAACAAGCTGATAAGCCGCGAGTCC
>n.538.6.bb_849
GTAGTTAGCCGGTGCTTCTTCTGCAGGTACCGTCACCACAAGCCTCGCCCCTGCTGAAAGCGGTTTACAACCCGAAGGCCGTCATCCCGCACGCGGCGTTGCTGCATCAGGCTTCCGCCCATTGTGCAATATTCCCCACTGCTGCCTCCCGTAGGAGTCTGGGCCGTATCTCAGTCCCAATGTGGCCGGTCGCCCTCTCAGGCCGGCTACCCGTCAAAGCCTTGGTAAGCCACTACCCACCAACAAGCTGATAAGCCGCGAGTCCATCCAAAACCGCCGAAGCTTTCCACCCCACCATGCAGCAA
>n.538.6.bb_850
GGAGTTAGCCGGTGCTTCTTCTGCGGGTAACGTCAATTGCTGAGGTTATTAACCTCAACACCTTCCTCCCCGCTGAAAGTACTTTACAACCCGAAGGCCTTCTTCATACACGCGGCATGGCTGCATCAGGCTTGCGCCCATTGTGCAATATTCCCCACTGCTGCCTCCCGTAGGAGTCTGGACCGTGTCTCAGTTCCAGTGTGGCTGGGTCATCCTCTCAGACCAGCTAGGGATCGTCGCCTAGGTGAGCCGTTACCCCACCTACTAGCTAATCCCATCTGGGCACATCTGATGGCAA
>n.538.6.bb_851
GTAGTTAGCCGTGGCTTTCTGATTAGGTACCGTCAAGACGTGCATAGTTACTTACACATTTATTCTTCCCTAATAACAGAGTTTTACGATCCGAAGACCTTCATCACTCACGCGGCGTTGCTCCGTCAGGCTTTCGCCCATTGCGGAAGATTCCCTACTGCTGCCTCCCGTAGGAGTCTGGACCGTGTCTCAGTTCCAGTGTGGCCGATCACCCTCTCAGGGTCGGCTACGCATCGTTGCCTTGGTAAGCCGTTACCTTACCAACTAGCTAATGCGGCGCGGATCCATCTATAAGTGACAGCAAAACCGTCTTTCACTATTGAACCATGCGGTTCAATATATTATCCGGTATTAGCTCCGGT
>n.538.6.bb_852
GTAGTTAGCCGTGGCTTTCTGATTAGGTACCGTCAAGACGTGCATAGTTACTTACACATTTATTCTTCCCTAATAACAGAGTTTTACGATCCGAAGACCTTCATCACTCACGCGGCGTTGCTGCATCAGGCTTCCGCCCATTGTGCAATATTCCCCACTGCTGCCTCCCGTAGGAGTCTGGGCCGTATCTCAGTCCCAATGGTGGCCGGGTCGCCCTCTCAGGCCGGCTACCCGTCAAAGCCTTGGTAAGCCACTACCCACCAACAAGCTGATGAGCCGCGAGTCCATCCAAAACCGC
>n.538.6.bb_853
GTAGTTAGCCGGTGCTTCTTCTGCAGGTACCGTCACCACAAGCTTCGCCCCTGCTGAAAGCGGTTTACAACCCGAAGGCCGTCATCCCGCACGCGGCGTTGCTGCATCAGGCTTCCGCCCATTGTGCAATATTCCCCACTGCTGCCTCCCGTAGGAGTCTGGGCCGTATCTCAGTCCCAATGTGGCCGGTCGCCCTCTCAGGCCGGCTACCCGTCAAAGCCTTGGTAACGCCACTACCCCACCAACAAGCTGATAAGCCGCGAGTCCATCCAAAACCGCCG
>n.538.6.bb_854
GTAGTTAGCCGTGGCTTTCTGATTAGGTACCGTCAAGACGTGCATAGTTACTTACACATTTATTCTTCCCTAATAACAGAGTTTTACGATCCGAAGACCTTCATCACTCACGCGGCGTTGCTCCGTCAGGCTTTCGCCCATTGCGGAAGATTCCCTACTGCTGCCTCCCGTAGGAGTCTGGACCGTGTCTCAGTTCCAGTGTGGCCGATCACCCTCTCAGGGTCGGCTACGCATCGTTGCCTTGGTAAGCCGTTACCTTACCAACTAGCTAATGCGGCGCGGATCCATCTATAAGTGACGGCAAAACCGTCTTTCACTATTGAACCATGCGG
>n.538.6.bb_855
GTAGTTAGCCGGTGCTTCTTCTGCAGGTACCGTCACCACAAGCTTCGCCCCTGCTGAAAGCGGTTTACAACCCGAAGGCCGTCATCCCGCACGCGGCGTTGCTGCATCAGGCTTCCGCCCATTGTGCAATATTCCCCACTGCTGCCTCCCGTAGGAGTCTGGGCCGCATCTCAGTCCCAATGTGGCCGGTCGCCCTCTCAGGCCGGCTACCCGGTCAAAGCCTTGGTAAGCCACTACCCACCAACAAGCTGATAAGCCGCGAGTCCATCCAAAACCGCCGAAGCTTTCCAACCCCCACCATGCAGCAAGGATTCCTATCCGGTATT
>n.538.6.bb_856
GGAGTTAGCCGGTGCTTCTTCTGCGGGTAACGTCAATTGCTGAGGTTATTAACCTCAACACCTTCCTCCCCGCTGAAAGTACTTACAACCCGAAGGCCTTCTTCATACACGCGGCATGGCTGCATCAGGCTTGCGCCCATTGTGCAATATTCCCCACTGCTGCCTCCCGTAGGAGTCTGGACCGTGTCTCAGTTCCAGTGTGGCTGGGTCATCCTCTCAGACCAGCTAGGGATCGTCGCCTAGGTGAGCCGTTACCCACCTACTAGCTAATCCCATCTGGGCACATCTGAT
>n.538.6.bb_857
GTAGTTAGCCGTGGCTTTCTGATTAGGTACCGTCAAGACGTGCATAGTTACTTACACATTTGTTCTTCCCTGATAACAGAGTTTTACGATCCGAAGACCTTCATCACTCACGCGGCGTTGCTCCGTCAGGCTTTCGCCCATTGCGGAAGATTCCCTACTGCTGCCTCCGTAGGAGTCTGGACCGTGTCTCAGTTCCAGTGTGGCCGATCACCCTCTCAGGTCGGCTACGCATCGTTGCCTTGGTAAGCCGTTACCTTACCAACTAGCTAATGCGGCGCGGATCCATCTATAAGTGACAGCAAAACCGTCTTTCACTATTGAACCATGCGGTTCAATATATTATCCGGTATTAGCTCCGGT
>n.538.6.bb_858
GGAGTTAGCCGGTGCTTCTTCTGCGGGTAACGTCAATTGCTGAGGTTATTAACCTCAACACCTTCCTCCCCGCTGAAAGTACTTTACAACCCGAAGGCCTTCTTCATACACGCGGCATGGCTGCATCAGGCTTGCGCCCATTGTGCAATATTCCCCACTGCTGCCTCCCGTAGGAGTCTGGACCGTGTCTCAGTTCCAGTGTGGCTGGGTCATCCTCTCAGACCAGCTAGGGATCGTCGCCTAGGTGAGCCGTTACCCCACCTACTAGCTAATCCCATCTGGGCACATCTGA
>n.538.6.bb_859
GTAGTTAGCCGTGGCTTTCTGATTAGGTACCGTCAAGACGTGCATAGTTACTTACACATTTGTTCTTCCCTAATAACAGAGTTTTACGATCCGAAGACCTTCATCACTCACGCGGCGTTGCTCCGTCAGGCTTTCGCCCATTGCGGAAGATTCCCTACTGCTGCCTCCCGTAGGAGTCTGGACCGTGTCTCAGTTCCAGTGTGGCCGATCACCCTCTCAGGGTCGGCTACGCATCGTTGCCTTGGTAAGCCGTTACCTTACCACTAGCTAATGCGGCGCGGATCCATCTATAAGTGACAGC
>n.538.6.bb_860
GTAGTTAGCCGTGGCTTTCTGATTAGGTACCGTCAAGACGTGCATAGTTACTTACACATTTGTTCTTCCCTAATAACAGAGTTTTACGATCCGAAGACCTTCATCACTCACGCGGCGTTGCTCCGTCAGGCTTTCGCCCATTGCGGAAGATTCCCTACTGCTGCCTCCCGTAGGGGTCTGGACCGTGTCTCAGTTCCAGTGTGGCCGATCACCCTCTCAGGGTCGGGCTACGCATCGTCGCCTTGGTAAGCCGTTACCTTACCAACTAGCTAATGCGGCGCGGATCCATCTATAAGTGACAGCAAAACCGTCTTTCGCTATTGAACCATGCGGTTCAATATA
>n.538.6.bb_861
GTAGTTAGCCGTGGCTTTCTGATTAGGTACCGTCAAGACGTGCATAGTTACTTACACATTTATTCTTCCCTAATAACAGAGTTTTACGATTCGAAGACCTTCATCACTCACGCGGCGTTGCTCCGTCAGGCTTTCGCCCATTGCGGAAGATTCCCTACTGCTGCCTCCCGTAGGAGTCTGGACCGTGTCTCAGTTCCAGTGTGGCCGATCACCCTCTCAGGGTCGGCTACGCATCGTTGCCTTGGTAAGCCGTTACCTTACCAACTAGCTAATGCGACGCGGATCCATCTATAAGTGACAGCAAAACCGTCTTTCACTATTGAACCATGCGGTTC
>n.538.6.bb_862
GTAGTTAGCCGGTGCTTCTTCTGCAGGTACCGTCACCACAAGCTTCGCCCCTGCTGAAAGCGGTTTACAACCCGAAGGCCGTCATCCCGCACGCGGCGTTGCTGCATCAGGCTTCCGCCCATTGTGCAATATTCCCCACTGCTGCCTCCCGTAGGAGTCTGGGCCGTATCTCAGTCCCAATGTGGCCGGTCGCCCTCTCAGGCCGGCTACCCGTCAAAGCCTTGGTAAGCCACTACCCCACCAACAAGCTGATAAGCCGCGAGTCCATCCAAAACCGCCGAAGCTTTCCCAACCCCACCATGCAGCAAGGATTCCTATCCGGTATT
>n.538.6.bb_863
GTAGTTAGCCGGTGCTTCTTCTGCAGGTACCGTCACCACAAGCTTCGCCCCTGCTGAAAGCGGTTTACAACCCGAAGGCCGTCATCCCGCACGCGGCGTTGCTGCATCAGGCTTCCGCCCATTGTGCAATATTCCCCACTGCTGCCTCCCGTAGGAGTCTGGGCCGTATCTCAGTCCCGATGTGGCCGGTCGCCCTCTCAGGCCGGCTACCCGTCAAAGCCTTGGTAAGCCACTACCCCACCAACAAGCTGATAAGCCGCGAGTCCATCCAAAACCGC
>n.538.6.bb_864
AAGTTAGCCGGTGCTTCTTCTGCGGGTAACGTCAATCGACACGGTTATTAACCGCATCGCCTTCCTCCCCGCTGAAAGTACTTTACAACCCGAAGGCCTTCTTCATACACGCGGCATGGCTGCATCAGGCTTGCGCCCATTGTGCAATATTCCCCACTGCTGCCTCCCGTAGGAGTCTGGGCCGTATCTCAGTCCCAATGTGGCCGGTCGCCCT
>n.538.6.bb_865
GTAGTTAGCCGTGGCTTTCTGATTAGGTACCGTCAAGACGTGCATAGTTACTTACACATTTGTTCTTCCCTAATAACAGAGTTTTACGATCCGAAGACCTTCATCACTCACGCGGCGTTGCTCCGTCAGGCTTTCGCCCATTGCGGAAGATTCCCTACTGCTGCCTCCCGTAGGAGTCTGGACCGTGTCTCAGTTCCAGTGTGGCCGATCCACCCTCTCAGGGTCGGCTACGCATCGTCGCCTTGGTAAGCCGTTACCTTACCAACTAGCTAATGCGGCGCGGATCCATCTATAAGTGAC
>n.538.6.bb_866
GGAGTTAGCCGGTGCTTCTTCTGCGGGTAACGTCAATCGACAAGGTTATTAACCTTATCGCCTTCCTCCCCGCTGAAAGTACTTTACAACCCGAAGGCCTTCTTCATACACGCGGCATGGCTGCATCAGGCTTGCGCCATTGTGCAATATTCCCCATGCTGCCTCCGTAGGAGTCTGGACCGTGTCTCAGTTCCAGTGTGGC
>n.538.6.bb_867
GTAGTTAGCCGTGGCTTTCTGATTAGGTACCGTCAAGACGTGCATAGTTACTTACACATTTGTTCTTCCCTAATAACAGAGTTTTACGATCCGAAGACCTTCATCACTCACGCGGCGTTGCTCCGTCAGGCTTTCGCCCATTGCGGAAGATTCCCTACTGCTGCCTCCCGTAGGAGTCTGGACCGTGTCTCAGTTCCAGTGTGGCCGATCACCCTCTCAGGGTCGGCTACGCATCGTTGCCTTGGTAAGCCGTTACCTTACCAACTAGCTAATGCGGCGCGGATCCATCTATAAGTGACAGCAAAACCGTCTTTCACTATTGAACCATGCGGTTCAATATATTATCCGGTATTAGCT
>n.538.6.bb_868
GTAGTTAGCCGTGGCTTTCTGATTAGGTACCGTCAAGACGTGCATAGTTACTTACACATTTATTCTTCCCTAATAACAGAGTTTTACGATCCGAAGACCTTCATCACTCACGCGGCGTTGCTCCGTCAGGCTTTCGCCCATTGCGGAAGATTCCCTACTGCTGCCTCCCGTAGGAGTCTGGACCGTGTCTCAGTTCCAGTGTGGCCGATCACCCTCTCAGGGTCGGCTACGCATCGTCGCCTTGGTAAGCCGTTACCTTACCAACTAGCTAATGCGGCGCGGATCCATCTATAAGTGACAGCAAAACCGTCTTTCACTATTGAACCATGCGGTTCAATATATTATCCGGTATT
>n.538.6.bb_869
GTAGTTAGCCGGTGCTTCTTCTGCAGGTACCGTCACCACAAGCTTCGCCCCTGCTGAAAGCGGTTTACAACCCGAAGGCCGTCATCCCGCACGCGGCGTTGCTGCATCAGGCTTGCGCCCATTGTGCAATATTCCCCACTGCTGCCTCCCGTAGGAGTCTGGACCGTGTCTCAGTTCCAGTGTGGCTGGTCATCCTCTCAGACCAGCTAGGGATCGTCGCCTAGGTGAGCCGT
>n.538.6.bb_870
GTAGTTAGCCGTGGCTTTCTGATTAGGTACCGTCAAGACGTGCATAGTTACTTACACATTTGTTCTTCCCTAATAACAGAGTTTTACGATCCGAAGACCTTCATCACTCACGCGGCGTTGCTCCGTCAGGCTTTCGCCCATTGCGGAAGATTCCCTACTGCTGCCTCCCGTAGGAGTCTGGACCGTGTCTCAGTTCCAGTGTGGCCGATCACCCTCTCAGGGTCGGCTACGCATCGTCGCCTTGGTAAGCCGTTACCTTACCACTAGCTAATGCGGCGCGGATCCATCTATAAGTGACAGCAAAACCGTCT
>n.538.6.bb_871
GTAGTTAGCCGGTGCTTCTTCTGCAGGTACCGTCACCACAAGCTTCGCCCCAGCTGAAAGCGGTTTACAACCCGAAGGCCGTCATCCCGCACGCGGCGTTGCTGCATCAGGCTTCCGCCCATTGTGCAATATTCCCCATGCTGCCTCCCGTAGGAGTCTGGGCCGTATCTCAGTCCCAATGTGGCCGGTCGCCCTCTCAGGCCCGGCTACCCGGTCAAAGCCTTGGTAACGCCACTACCCCACCAACAAGCTGATAAGCCGCGAGTCCATCCAAAACC
>n.538.6.bb_872
GTAGTTAGCCGTGGCTTTCTGATTAGGTACCGTCAAGACGTGCATAGTTACTTACACATTTGTTCTTCCCTAATAACAGAGTTTTACGATCCGAAGACCTTCATCACTCACGCGGCGTTGCTCCGTCAGGCTTTCGCCCATTGCGGAAGATTCCCTACTGCTGCCTCCCGTAGGAGTCTGGACCGTGTCTCAGTTCCAGTGTGGCCGATCACCCTCTCAGGGTCGGCTACGCATCGTTGCCTTGGTAAGCCGTTACCTTACAACTAGCTAATGCGGCGCGGATCCATCTATAAGTGACAGCAAAACCGTCTTTCACTATTGAACCATGCGGTTC
>n.538.6.bb_873
GTAGTTAGCCGTGGCTTTCTGATTAGGTACCGTCAAGACGTGCATAGTTACTTACACATTTGTCTTCCCTAATAACAGAGTTTTACGATCCGAAGACCTTCATCACTCACGCGGCGTTGCTCCGTCAGGCTTTCGCCCATTGCGGAAGATTCCCTACTGCTGCCTCCCGTAGGAGTCTGGACCGTGTCTCAGTTCCAGTGTGGCCGATCACCCTCTCAGGGTCGGCTACGCATCGTTGCCTTGTAAGCGTTACCTTACCAACTAGCTAATGCGGCGCGGATCCATCTATAAGTGACAGC
>n.538.6.bb_874
GTAGTTAGCCGGTGCTTCTTCTGCAGGCACCGTCACCACAAGCTTCGCCCCTGCTGAAAGCGGTTTACAACCCGAAGGCCGTCATCCCGCACGCGGCGTTGCTGCATCAGGCTTCCGCCCATTGTGCAATATTCCCCACTGCTGCCTCCCGTAGGAGTCTGGGCCGTATCTCAGTCCCAATGTGGCCGGTCGCCCTCTCAGGCCGGCTACCCGTCAAAGCCTTAGTAAGCCACTACCCACCAACAAGCTGATAAGCCGCGAGTCCATCCAAAACCGCCGAAGCTTTCCAACCCCCACATGCAGCAAGGATTCCTATCCGGTATT
>n.538.6.bb_875
GTAGTTAGCCGGTGCTTCTTCTGCAGGTACCGTCACCACAAGCTTCGCCCCTGCTGAAAGCGGTTTACAACCCGAAGGCCGTCATCCCGCACGCGGCGTTGCTGCATCAGGCTTCCGCCCATTGTGCAATATTCCCCACTGCTGCCTCCCGTAGGAGTCTGGGCCGTATCTCAGTCCCAATGTGGCCGGTCGCCCTCTCAGGCCGGCTACCCGTCAAAGCCTTGGTAAGCCACTACCCACCAACAAGCTGATAAGCCGCGAGTCCATCC
>n.538.6.bb_876
GTAGTTAGCCGGTGCTTCTTCTGCAGGTACTGTCACCACAAGCCTCGCCCCTGCTGAAAGCGGTTTACAACCCGAAGGCCGTCATCCCGCACGCGGCGTTGCTGCATCAGGCTTCCGCCCATTGTGCAATATTCCCCACTGCTGCCTCCCGTAGGAGTCTGGGCCGTATCTCAGTCCCAATGTGGCCGGTCGCCCTCTCAGGCCGGCTACCCGTCAAAGCCTTGGTAAGCCACTACCCACCAACAAGCTGATAAGCCGCGAGTCCAT
>n.538.6.bb_877
GTAGTTAGCCGGTGCTTCTTCTGCAGGTACCGTCACCACAAGCTTCGCCCCTGCTGAAAGCGGTTTACAACCCGAAGGCCGTCATCCCGCACGCGGCGTTGCTGCATCAGGCCTCCGCCCATTGTGCAATATTCCCCACTGCTGCCTCCCGTAGGAGTCTGGGCCGTATCTCAGTCCCAATGTGGCCGGTCGCCCTCTCAGGCCGGCTACCCGTNAAAGCCTTGGTAAGCCACTACCCCACCAACAAGCTGATAAGCCGCGAGTCCATCCAAAACCGCCGAAGCTTTCCCAACCCCACCATGCAGCAAGGATTCCTATCCGGTATTAGCCCCAGTTTCCTGAAGTTATCCCGAAG
>n.538.6.bb_878
GTAGTTAGCCGTGGCTTTCTGATTAGGTACCGTCAAGACGTGCATAGTTACTTACACATTTGTTCTTCCCTAATAACAGAGTTTTACGATCCGAAGACCTTCATCACTCACGCGGCGTTGCTCCGTCAGGCTTTCGCCCATTGCGGAGGATTCCCTACTGCTGCCTCCCGTAGGAGTCTGGACCGTGTCTCAGTTCCAGTGTGGCCGATCACCCTCTCAGGGTCGGCTACGCATCGTTGCCTTGGTAAGTCCGTTACCTTACCAACTAGCTAATGCGGCGCGGA
>n.538.6.bb_879
GTAGTTAGCCGTGGCTTTCTGATTAGGTACCGTCAAGACGTGCATAGTTACTTACACATCTGTTCTTCCCTAATAACAGAGTTTTACGATCCGAAGACCTTCATCACTCACGCGGCGTTGCTCCGTCAGGCTTTCGCCCATTGCGGAAGATTCCCTACTGCTGCCTCCCGTAGGAGTCTGGACCGTGTCTCAGTTCCAGTGTGGCCGATCACCCTCTCAGGTCGGCTACGCATCGTTGCCTTGGTAAGCCGTTACCTTACCAACTAGCTAATGCGGCGCGGATCCATCTATAAGTGACAGCAAAACCGTCTTTCACTATTGAACCATGCGGTTCAATATATATCCGGTATTAGCTCCGGTTTCCCGAAGTTATCCCAGTCTTATAGGTAGGTTATCCCACGT
>n.538.6.bb_880
GGAGTTAGCCGGTGCTTCTTCTGCGGGTAACGTCAATCGACAAGGTTATTAACCTTATCGCCTTCCTCCCCGCTGAAAGTACTTTACAACCCGAAGGCCTTCTTCATACACGCGGCATGGCTGCATCAGGCTTGCGCCCATTGTGCAATATTCCCCACTGCTGCCTCCCGTAGGAGTCTGGACCGTGTCTCAGTTCCAGTGTGGCTGGGTCATCCTCTCAGACCAGCTAGGGATCGTCGCCTAGGTGAGCCGTTACCCACCTACTAGCTAATCCCATCTGGGCACATCTGAT
>n.538.6.bb_881
GTAGTTAGCCGGTGCTTCTTCTGCAGGTACCGTCACCACAAGCTTCGCCCCTGCTGAAAGCGGTTTACAACCCGAAGGCCGTCATCCCGCACGCGGCGTTGCTGCATCAGGCTTCCGCCCATTGTGCAATATTCCCCACTGCTGCCTCCCGTAGGAGTCTGGGCCGTATCTCAGTCCCAATGTGGCCGGTCGCCCTCTCAGGCCGGCTACCCGTCAAAGCCTTGGTAAGCCACTACCCCACCAACAAGCTGATAAGCCGCGAGTCCATCCAAAACCGC
>n.538.6.bb_882
GTAGTTAGCCGGTGCTTCTTCTGCAGGTACCGTCACCACAAGCTTCGCCCCTGCTGAAAGCGGTTTACAACCCGAAGGCCGTCATCCCGCACGCGGCGTTGCTGCATCAGGCTTCCGCCCATTGTGCAATATTCCCCACTGCTGCCTCCCGTAGGAGTCTGGGCCGTATCTCAGTCCCAATGTGGCCGTCGCCCTCTCAGGCCGGCTACCCGGTCA
>n.538.6.bb_883
GTAGTTAGCCGGTGCTTCTTCTGCAGGTACCGTCACCACAAGCTTCGCCCCTGCTGAAAGCGGTTTACAACCCGAAGGCCGTCATCCCGCACGCGGCGTTGCTGCATCAGGCTTCCGCCCATTGTGCAATATTCCCCACTGCTGCCTCCCGTAGGAGTCTGGGCCGTATCTCAGTCCCAATGTGGCCGGTCGCCCTCTCAGGCCGGCTACCCGTCAAAGCCTTGGTAACGCCACTACCCACCAACAAGCTGATAAGCCGCGAGTCCATCCAAAACCGC
>n.538.6.bb_884
GTAGTTAGCCGTGGCTTTCTGATTAGGTACCGTCAAGACGTGCATAGTTACTTACACATTTGTTCTTCCCTAATAACAGAGTTTTACGATCCGAAGACCTTCATCACTCACGCGGCGTTGCTCCGTCAGGCTTTCGCCCATTGCGGAAGATTCCCTACTGCTGCCTCCCGTAGGAGTCTGGCCGTGTCTCAGTCCCAGTGTGGCCGATCACCCTCT
>n.538.6.bb_885
GGAGTTAGCCGGTGCTTCTTCTGCGGGCAACGTCAATCGACAAGGTTATTAACCTTATCGCCTTCCTCCCCGCTGAAAGTGCTTTACAACCCGAAGGCCTTCTTCACACACGCGGCATGGCTGCATCAGGCTTGCGCCCATTGTGCAATATTCCCCACTGCTGCCTCCCGTAGGAGTCTGGACCGTGTCTCAGTTCCAGTGTGGCTGGGTCATCCTCTCAGACCAGCTAGGGATCGTCGCCTAGGTGAGCCGTTACCCACCTACCAGCTAATCCCATCTGGGCACATCTGATGGCATGAGGCCCGAAGGTCCCCACTTTGGTCTTGCGACGTTATGCGGTATTAGCTACCGTTTCCAGTAGTTATCCCCTCATCAGGCCAGTTTCCCCAGACCATTACCTCACCCCGT
>n.538.6.bb_886
GGAGTTAGCCGGTGCTTCTTCTGCGGGTAACGTCAATCGACAAGGTTATTAACCTTATCGCCTTCCTCCCCGCTGAAAGTACTTTACAACCCGAAGGCCTTCTTCATACACGCGGCATGGCTGCATCAGGCTTGCGCCCATTGTGCAATATTCCCACTGCTGCCTCCGTAGGAGTCTGGACCGTGTCTCAGTTCCAGTGTGGCTGGTCATCCTCTCAGACCAGCTAGGGATCGTCGCCTAGGTGAGCCGTTACCCACCTACTAGCTAATCCCATCTGGGCACATCTGATGG
>n.538.6.bb_887
GGAGTTAGCCGGTGCTTCTTCTGCGGGTAACGTCAATCGACAAGGTTATTAACCTTATCGCCTTCCTCCCCGCTGAAAGTACTTTACAACCCGAAGGCCTTCTTCATACACGCGGCATGGCTGCATCAGGCTTGCGCCCATTGTGCAATATTCCCACTGCTGCCTCCGTAGGAGTCTGGACCGTGTCTCAGTTCCAGTGTGGCTGGTCATCCTCTCAGACCAGCTAGGGATCGTCGCCTAGGTGAGCCGTTACCCACCTACTAGCTAATCCCATCTGGGCACATCTGATGGC
>n.538.6.bb_888
GTAGTTAGCCGGTGCTTCTTCTGCAGGTACCGTCACCACAAGCTTCGCCCCTGCTGAAAGCGGTTTACAACCCGAAGGCCGTCATCCCGCACGCGGCGTTGCTGCATCAGGCTTCCGCCCATTGTGCAATATTCCCCACTGCTGCCTCCCGTAGGAGTCTGGGCCGTATCTCAGTCCCAATGTGGCCGGTCGCCCTCTCAGGCCGGCTACCCGTCAAAGCCTTGGTAAGCCACTACCCACCAACAAGCTGATAAGCCGCGAGTCCATCCAAAACCGCCGAAGCTTTCCAACCCCCACCATGCAGCAAGGATTCCTATCCGGTATTA
>n.538.6.bb_889
GTAGTTAGCCGGTGCTTCTTCTGCAGGTACCGTCACCACAAGCTTCGCCCCTGCTGAAAGCGGTTTACAACCCGAAGGCCGTCATCCCGCACGCGGCGTTGCTGCATCAGGCTTCCGCCCATTGTGCAATATTCCCCACTGCTGCCTCCCGTAGGAGTCTGGGCCGTATCTCAGTCCCAATGTGGCCGGTCGCCCTCTCAGGCCGGCTACCCGTCAAAGCCTTGGTAAGCCACTACCCACCAACAAGCTGATAAGCCGCGAGTCCATCCAAAACCGCCGAAGCTTTCCAACCCCACCATGCAGCAAGGATTCCTATCCGGT
>n.538.6.bb_890
GTAGTTAGCCGTGGCTTTCTGATTAGGTACCGTCAAGACGTGCATAGTTACTTACACATTTGTTCTTCCCTAATAACAGAGTTTTACGATCCGAAGACCTTCATCACTCACGCGGCGTTGCTCCGTCAGGCTTTCGCCCATTGCGGAAGATTCCCTACTGCTGCCTCCCGTAGGAGTCTGGACCGTGTCTCAGTTCCAGTGTGGCCGATCACCCTCTCAGGGTCGGCTACGCATCGTCGCCTTGGTAAGCCGTTACCTTACCAACTAGCTAATGCGGCGCGGATCCATCTATAAGTGACAGCAAAACCGTCTTTCACTATTGAACCATGCGGTTCAATATATTATCCGGTATTAGCTCCGGTTTCCCGAAGTTATCCCAGTCTTATAGGTAGGTTATCCCACGTGTTACTCCACCCCGTCCCGCCCGCTAACGTCAGAGGGAGCAAGCCTCCCTCGTCTGTTC
>n.538.6.bb_891
GTAGTTAGCCGTGGCTTTCTGATTAGGTACCGTCAAGACGTGCATAGTTACTTACACATTTCGTTCTTCCCTAATAACAGAGTTTTACGATCCGAAGACCTTCATCACTCACGCGGCGTTGCTCCGTCAGCTTTCGCCCATTGCGGAAGATTCCCTACTGCTGCCTCCCGTAGGAGTCTGGACCGTGTCTCAGTTCCAGTGTGGCCGATCACCCTCTCAGGGTCGGCTACGCATCGTTGCCTTGGTAAGCCGTTACCTTACCAACTAGCTAATGCGGCGCGGATCCATCTATAAGTGACAGCAAAACCGTCTTTCACTATTGAACCATGCGGTTCAATATATTATCCGGTATT
>n.538.6.bb_892
GTAGTTAGCCGTGGCTTTCTGATTAGGTACCGTCAAGACGTGCATAGTTACTTACACATTTGTTCTTCCCTAATAACAGAGTTTTACGATCCGAAGACCTTCATCACTCACGCGGCGTTGCTCCGTCAGGCTTTCGCCCATTGCGGAAGATTCCCTACTGCTGCCTCCCGTAGGAGTCTGGACCGTGTCTCAGTTCCAGTGTGGCCGATCACCCTCTCAGGGTCGGCTACGCATCGTCGCCTTGGTAAGCCGTTACCTTACCAACTAGCTAATGCGGCGCGGATCCATCTATAAGTGACAGCAAAACCGTCTTTCACTATTGAACCATGCGGTTCAATATATT
>n.538.6.bb_893
GTAGTTAGCCGTGGCTTTCTGATTGGGTACCGTCAAGACGTGCATAGTTACTTACACATTTGTTCTTCCCTAATAACAGAGTTTTACGATCCGAAGACCTTCATCACTCACGCGGCGTTGCTCCGTCAGGCTTTCGCCCATTGCGGAAGATTCCCTACTGCTGCCTCCCGTAGGAGTCTGGACCGTGTCTCAGTTCCAGTGTGGCCGATCACCCTCTCAGGGTCGGCTACGCATCGTCGCCTTGGTAAGCCGTTACCTTACCACTAGCTAATGCGGCGCGGATCCATCTATAAGTGACA
>n.538.6.bb_894
GTAGTTAGCCGGTGCTTCTTCTGCAGGTACCGTCACCACAAGCTTCGCCCCTGCTGAAAGCGGTTTACAACCCGAAGGCCGTCATCCCGCACGCGGCGTTGCTGCATCAGGCTTCCGCCCATTGTGCAATATTCCCCACTGCCGCCTCCCGTAGGAGTCTGGGCCGTATCTCAGTCCCAATGTGGCCGTCGCCCTCTCAGGCCGGCTACCCGGTCAAAGCCTTGGGTAANCCACTACCCCACCAACAAGCTGATAAGCCGCGAGTCCATC
>n.538.6.bb_895
GTAGTTAGCCGTGGCTTTCTGATTAGGTACCGTCAAGACGTGCATAGTTACTTACACATTTGTTCTTCCCTAATAACAGAGTTTTACGATCCGAAGACCTTCATCACTCACGCGGCGTTGCTCCGTCAGGCTTTCGCCCATTGCGGAAGATTCCCTACTGCTGCCTCCCGTAGGAGTCTGGACCGTGTCTCAGTTCCAGTGTGGCCGATCACCCTCTCAGGGTCGGCTACGCATCGTTGCCTTGGTAAGCCGTTACCTTACCAACTAGCTAATGCGGCGCGGATCCATCTATAAGTGACAGCAAAACCGTCTTTCACTATTGAACCATGCGGTTCAATAT
>n.538.6.bb_896
GTAGTTAGCCGTGGCTTTCTGATTAGGTACCGTCAAGACGTGCATAGTTACTTACACATTTGTTCTTCCCTAATAACAGAGTTTTACGATCCGAAGACCTTCATCACTCACGCGGCGTTGCTCCGTCAGGCTTTCGCCCATTGCGGAAGATTCCCTACTGCTGCCTCCCGTAGGAGTCTGGACCGTGTCTCAGTTCCAGTGTGGCCGATCACCCTCTCAGGGTCGGCTACGCATCGTTGCCTTGGTAAGCCGTTACCTTACAACTAGCTAATGCGCCGCGGGTCCATCTCATAGCGGATTGCTCCTTTGGTTGAATGATGATGCCATCTTTCAACG
>n.538.6.bb_897
GTAGTTAGCCGTGGCTTTCTGATTAGGTACCGTCAAGACGTGCATAGTTACTTACACATTTATTCTTCCCTAATAACAGAGTTTTACGATCCGAAGACCTTCATCACTCACGCGGCGTTGCTCCGTCAGGCTTTCGCCCATTGCGGAAGATTCCCTACTGCTGCCTCCCGTAGGAGTCTGGACCGTGTCTCAGTTCCAGTGTGGCCGATCACCCTCTCAGGGTCGGCTACGCATCGTTGCCTTGGTAAGCCGTTACCTTACCAACTAGCTAATGCGGCGCGGATCCATCTATAAGTGACAGCAAAACCGTCTTTCACTATTGAACCATGCGGTTCAATATATTATCCGGTATT
>n.538.6.bb_898
GTAGTTAGCCGTGGCTTTCTGATTAGGTACCGTTAAGACGTGCATAGTTACTTACACATTTGTTCTTCCCTAATAACAGAGTTTTACGATCCGAAGACCTTCATCACTCACGCGGCGTTGCTCCGTCAGGCTTTCGCCCATTGCGGAAGATTCCCTACTGCTGCCTCCCGTAGGAGTCTGGACCGTGTCTCAGTTCCAGTGTGGCCCGATCACCCTCTCAGGGTCGGCTACGCATCGTTGCCTTGGTAAAGCCGTTACCTTACCAACTAGCTAATGCGGCGCGGATCCATCTATAAGTGACAGCAAAACCGTCTTTCACTATTGAACCATGCGGTTCAATATATT
>n.538.6.bb_899
GGAGTTAGCCGTGGCTTTCTGATTAGGTACCGTCAAGACGTGCATAGTTACTTACACATTTGTTCTTCCCTAATAACAGAGTTTTACGATCCGAAGACCTTCATCACTCACGCGGCGTTGCTCCGTCAGGCTTTCGCCCATTGCGGAAGATTCCCTACTGCTGCCTCCCGTAGGAGTCTGGACCGTGTCTCAGTTCCAGTGTGGCCGATCACCCTCTCAGGGTCGGCTACGCATCGTTGCCTGGTAAGCCGTTACCTTACACTAGCTAATGCGGCGCGGATCCATCTATAAGTGACAGCAAAACGTCTTTCACTATTGAACCATGCGGTTCAATATATT
>n.538.6.bb_900
GGAGTTAGCCGTGGCTTTCTGATTAGGTACCGTCAAGACGTGCATAGTTACTTACACATTTGTTCTTCCCTAATAACAGAGTTTTACGATCCGAAGACCTTCATCACTCACGCGGCGTTGCTCCGTCAGGCTTTCGCCCATTGCGGAAGATTCCCTACTGCTGCCTCCCGTAGGAGTCTGGACCGTGTCTCAGTTCCAGTGTGGCCGATCACCCTCTCAGGGTCGGCTACGCATCGTTGCCTTGGTAAGCCGTTACCTTACCAACTAGCTAATGCGGCGCGGATCCATCTATAAGTGACAGCAAAACCGTCTTTCACTA
>n.538.6.bb_901
GTAGTTAGCCGTGGCTTTCTGATTAGGTACCGTCAAGACGTGCATAGTTACTTACACATTTGTTCTTCCCTAATAACAGAGTTTTACGATCCGAAGACCTTCATCACTCACGCGGCGTTGCTCCGTCAGGCTTTCGCCCATTGCGGAAGATTCCCTACTGCTGCCTCCCGTAGGAGTCTGGACCGTGTCTCAGTTCCAGTGTGGCCGATCACCCTCTCAGGGTCGGCTACGCATCGTTGCCTTGGTAAGCCGTTACCTTACCAACTAGCTAATGCGGCGCGGATCCATCTATAAGTGACAGCAAAACCGTCTTTCACTATTGAACCATGCGGTTCAATATATTATCCGGTATTAGCTCCCGGT
>n.538.6.bb_902
GTAGTTAGCCGTGGCTTTCTGATTAGGTACCGTCAAGACGTGCATAGTTACTTACACATTTATTCTTCCCTAATAACAGAGTTTTACGATCCGAAGACCTTCATCACTCACGCGGCGTTGCTCCGTCAGGCTTTCGCCCATTGCGGAAGATTCCCTACTGCTGCCTCCCGTAGGAGTCTGGACCGTGTCTCAGTTCCAGTGTGGCCGATCACCCTCTCAGGGTCGGCTACGCATCCGTCGCCTTGGTAAGCCGTTACCTTACCAACTAGCTAATGCGGCGCGGATCCATCTATAAGTGACAGCAAAACCGTCTTTCACTATTGAACCATGCGGTTCAATATATTAT
>n.538.6.bb_903
GTAGTTAGCCGTGGCTTTCTGATTAGGTACCGTCAAGACGTGCATAGTTACTTACACATTTGTTCTTCCCTAATAACAGAGTTTTACGATCCGAAGACCTTCATCACTCACGCGGCGTTGCTCCGTCAGGCTTTCGCCCATTGCGGAAGATTCCCTACTGCTGCCTCCCGTAGGAGTCTGGACCGTGTCTCAGTTCCAGTGTGGCCGATCACCCTCTCAGGGTCGGCTACGCATCGTCGCCTTGGTAAGCCGTTACCTTACCAACTAGCTAATGCGGCGCGGATCCATCTATAAGTGACAGCAAAACCGTCTTT
>n.538.6.bb_904
GTAGTTAGCCGTGGCTTTCTGAATAGGTACCGTCAAGACGTGCATAGTTACTTACACATTTGTTCTTCCCTAATAACAGAGTTTTACGATCCGAAGACCTTCATCACTCACGCGGCGTTGCTCCGTCAGGCTTTCGCCCATTGCGGAAGATTCCCTACTGCTGCCTCCCGTAGGAGTCTGGACCGTGTCTCAGTTCCAGTGTGGCCGATCACCCTCTCAGGGTCGGCTACGCATCGTTGCCTTGGTAAGCCGTTACCTTACCAACTAGCTAATGCGGCGCGGATCCAACTATAAGTGACAGCAAAACCGTCTTTCACTATTGAACCATGCGGTTCAATATATTATCCGGT
>n.538.6.bb_905
GTAGTTAGCCGTGGCTTTCTGATTAGGTACCGTCAAGACGTGCATAGTTACTTACACATTTGTTCTTCCCTAATAACAGAGTTTTACGATCCGAAGACCTTCATCACTCACGCGGCGTTGCTCCGTCAGGCTTTCGCCCATTGCGGAAGATTCCCTACTGCTGCCTCCCGTAGGAGTCTGGACCGTGTCTCAGTTCCAGTGTGGCCGATCACCCTCTCAGGGTCGGCTACGCATCGTTGCCTTGGTAAGCCGTTACCTTACCAACTAGCTAATGCGGCGCGGATCCATCTATAAGTGACAGCAAAAACCGTCTTTCACTATTGAACCATGCGGTTCAATATATTATCCGGTATTAGCTCCGGT
>n.538.6.bb_906
GTAGTTAGCCGGTGCTTCTTCTGCAGGTACCGTCACCACAAGCTTCGCCCCTGCTGAAAGCGGTTTACAACCCGAAGGCCGTCATCCCGCACGCGGCGTTGCTGCATCAGGCTTCCGCCCATTGTGCAATATTCCCCACTGCTGCCTCCCGTAGGAGTCTGGGCCGTATCTCAGTCCCAATGTGGCCGGTCGCCCTCTCAGGCCGGCTACCCGTCAAAGCCTTGGTAACGCCACTACCCCACCAACAAGCTGATAAGCCGCGAGTCCATCCAAAACCG
>n.538.6.bb_907
GTAGTTAGCCGTGGCTTTCTGATTAGGTACCGTCAAGACGTGCATAGTTACTTACACATTTGTTCTTCCCTAATAACAGAGTTTTACGATCCGAAGACCTTCATCACTCACGCGGCGTTGCTCCGTCAGGCTTTCGCCCATTGCGGAAGATTCCCTACTGCTGCCTCCCGTAGGAGTCTGGACCGTGTCTCAGTTCCAGTGTGGCCGATCCACCCTCTCAGGGGTCGGCTACGCATCGTTGCCTTGGTAAGCCGTTACCTTACCAACTAGCTAATGCGGCGCGGATCCATCTATAAGTGA
>n.538.6.bb_908
GTAGTTAGCCGGTGCTTCTTCTGCAGGTACCGTCACCACAAGCTTCGCCCCTGCTGAAAGCGGTTTACAACCCGAAGGCCGTCATCCCGCACGCGGCGTTGCTGCATCAGGCTTCCGCCCATTGTGCAATATTCCCCACTGCTGCCTCCCGTAGGAGTCTGGGCCGTATCTCAGTCCCAATGTGGCCGGTCGCCCTCTCAGGCCGGCTACCCGTCAAAGCCTTGGTAAGCCACTACCCCACCGACAAGCTGATAAGCCGCGAGTCCATCCAAAACCGCCGAAGCTTTTCCAACCCCACCATGCAGCAAGGATTCCTATCCGGTATTAGCCCCAGTTTCCTGAAGTTATCCCGAAG
>n.538.6.bb_909
GGAGTTAGCCGGTGCTTCCTCTGCGGGTAACGTCAATTGCTGAGGTTATTAACCTCAACACCTTCCTCCCCGCTGAAAGTACTTTACAACCCGAAGGTCTTCTTCATACACGCGGCATGGCTGCATCAGGCTTGCGCCCATTGTGCAATATTCCCCACTGCTGCCTCCCGTAGGAGTCTGGACCGTGTCTCAGTTCCAGTGTGGCTGGGTCATCCTCTCAGACCAGCTAGGGATCGTCGCCTAGGTGAGCCGTT
>n.538.6.bb_910
GTAGTTAGCCGTGGCTTTCTGATTAGGTACCGTCAAGACGTGCATAGTTACTTACACATTTGTTCTTCCCTAATAACAGAGTTTTACGATCCGAAGACCTTCATCACTCACGCGGCGTTGCTCCGTCAGGCTTTCGCCCATTGCGGAAGATTCCCTACTGCTGCCTCCCGTAGGAGTCTGGACCGTGTCTCAGTTCCAGTGTGGCCGATCACCCTCTCAGGGTCGGCTACGCATCGTTGCCTTGGTAAGCCGTTACCTTACCAACTAGCTAATGCGGCGCGGATCCATCTATAAGTGAC
>n.538.6.bb_911
GTAGTTAGCCGGTGCTTCTTCTGCAGGTACCGTCACCACAAGCTTCGCCCCTGCTGAAAGCGGTTTACAACCCGAAGGCCGTCATCCCGCACGCGGCGTTGCTGCATCAGGCTTCCGCCCATTGTGCAATATTCCCCACTGCTGCCTCCCGTAGGAGTCTGGGCCGTATCTCAGTCCCAATGTGGCCGGTCGCCCTCTCAGGCCGGCTACCCGTCAAAGCCTTGGTAAGCCACTACCCCACCAACAAGCTGATAAGCCGCGAGTCCAT
>n.538.6.bb_912
GTAGTTAGCCGTGGCTTTCTGATTAGGTACCGTCAAGACGTGCATAGTTACTTACACATTTGTTCTTCCCTAATAACAGAGTTTTACGATCCGAAGACCTTCATCACTCACGCGGCGTTGCTCCGTCAGGCTTTCGCCCATTGCGGAAGATTCCCTACTGCTGCCTCCCGTAGGAGTCTGGACCGTGTCTCAGTTCCAGTGTGGCCGATCACCCTCTCAGGGTCGGCTACGCATCGTTGCCTTGGTAAGCCGTTACCTTACCAACTAGCTAATGCGGCGCGGATCCATCTATGAGTGAC
>n.538.6.bb_913
GTAGTTAGCCGTGGCTTTCTGATTAGGTACCGTCAAGACGTGCATAGTTACTTACACATTTGTTCTTCCCCAATAACAGAGTTTTACGATCCGAAGACCTTCATCACTCACGCGGCGTTGCTCCGTCAGGCTTTCGCCCATTGCGGAAGATTCCCTACTGCTGCCTCCCGTAGGAGTCTGGACCGTGTCTCAGTTCCAGTGTGGCCGATCACCCTCTCAGGGTCGGCTACGCATCGTTGCCTTGGTAAGCCGTTACCTTACCAACTAGCTAATGCGGCGCGGATCCATCTATAAGTGACAGCAAAACCGTCTTTCACTATTGAACCATGCGGTTCAATATATTATCCGGTATTAGCTCCGGT
>n.538.6.bb_914
GTAGTTAGCCGGTGCTTCTTCTGCAGGTACCGTCACCACAAGCTTCGCCCCTGCTGAAAGCGGTTTACAACCCGAAGGCCGTCATCCCGCACGCGGCGTTGCTGCATCAGGCTTCCGCCCATTGTGCAATATTCCCCACTGCTGCCTCCCGTAGGAGTCTGGGCCGTATCTCAGTCCCAATGTGGCCGTCGCCCTCTCAGGCCGGCTACCCGTCAAAGCCTTGGTAAGCCACTACCCCACCAACAAGCTGATAAGCCGCGAGTCCATCCAAAACCGCCGA
>n.538.6.bb_915
GGAGTTAGCCGGTGCTTCTTCTGCGGGTAACGTCAATCGACAAGGTTATTAACCTTATCGCCTTCCTCCCCGCTGAAAGTACTTTACAACCCGAAGGCCTTCTTCATACACGCGGCATGGCTGCATCAGGCTTGCGCCCATTGTGCAATATTCCCCATGCTGCCCTCCCGTAGGAGTCTGGACCGTGTCTCAGTTCCAGTGTGGCTGGTCATCCTC
>n.538.6.bb_916
GGAGTTAGCCGGTGCTTCTTCTGCGGGTAACGTCAATTGCTGAGGTTATTAACCTCAACACCTTCCTCCCCGCTGAAAGTACTTTACAACCCGAAGGCCTTCTTCATACACGCGGCATGGCTGCATCAGGCTTGCGCCCATTGTGCAATATTCCCCACTGCTGCCTCCCGTAGGAGTCTGGACCGTGTCTCAGTTCCAGTGTGGCTGGGTCATCCTCTCAGACCAGCTAGGGATCGTCGCCTAGGTGAGCCGTTACCCCACCTACTAGCTAATCCCATCTGGGCACATCTGAT
>n.538.6.bb_917
GTAGTTAGCCGGTGCTTCTTCTGCAGGTACCGTCACCACAAGCTTCGCCCCTGCTGAAAGCGGTTTACAACCCGAAGGCCGTCATCCCGCACGCGGCGTTGCTGCATCAGGCTTCCGCCCATTGTGCAATATTCCCCACTGCTGCCTCCCGTAGGAGTCTGGGCCGTATCTCAGTCCCAATGTGGCCGGTCGCCCTCTCAGGCCGGCTACCCGGTCAAAGCCTTGGTAAGCCACTACCCACCAACAAGCTGATAAGCCGCGAGTCCATCCAAAACCGC
>n.538.6.bb_918
GGAGTTAGCCGGTGCTTCTTCTGCGGGTAACGTCAATCGACAAGGTTATTAACCTTATCGCCTTCCTCCCCGCTGAAAGTACTTTACAACCCGAAGGCCTTCTTCATACACGCGGCATGGCTGCATCAGGCTTGCGCCATTGTGCAATATTCCCCACTGCTGCCTCCGTAGGAGTCTGGACCGTGTCTCAGTTCCAGTGTGGCTGGTCATCCTCTCAGACCAGCTAGGGATCGTCGCCTAGGTGAGCCGTTACCCACCTACTAGCTAATCCCATCTGGGCACATCTGATGGCAAGAGGCCCGAAGGTCCCCTCTTTGGTCTTGCGACGTTATGCGGTATTAGCTACCGTTTCCA
>n.538.6.bb_919
GTAGTTAGCCGGTGCTTCTTCTGCAGGTACCGTCACCACAAGCTTCGCCCCTGCTGAAAGCGGTTTACAACCCGAAGGCCGTCATCCCGCACGCGGCGTTGCTGCATCAGGCTTCCGCCCATTGTGCAATATTCCCCACTGCTGCCTCCCGTAGGAGTCTGGGCCGTATCTCAGTCCCAATGTGGCCGGTCGCCCTCTCAGGCCGGCTACCCGTCAAAGCCTTGGTAACGCCACTACCCCACCAACAAGCTGATAAGCCGCGAGTCCATCCAAAACCGCCGA
>n.538.6.bb_920
GGAGTTAGCCGGTGCTTCTTCTGCGGGTAACGTCAATCGACAAGGTTATTAACCTTATCGCCTTCCTCCCCGCTGAAAGTACTTTACAACCCGAAGGCCTTCTTCATACACGCGGCATGGCTGCATCAGGCTTGCGCCCATTGTGCAATATTCCCCACTGCTGCCTCCGTAGGAGTCTGGACCGTGTCTCAGTTCCAGTGTGGCTGGTCATCCTCTCAGACCAGGCTAGGGATCGTCGCCTAGGTGAGCCGTTACCCACCTACTAGCTAATCCCATCTGGGC
>n.538.6.bb_921
GTAGTTAGCCGGTGCTTCTTCTGCAGGTACCGTCACCACAAGCTTCGCCCCTGCTGAAAGCGGTTTACAACCCGAAGGCCGTCATCCCGCACGCGGCGTTGCTGCATCAGGCTTCCGCCCATTGTGCAATATTCCCCACTGCTGCCTCCCGTAGGAGTCTGGGCCTATCTCAGTCCCAATGTGGCCGGTCGCCCTCTCAGGCCGGCTACCCGTCAAAGCCTTGGTAACGCCACTACCCCACCAACAAGCTGATAAGCCGCGAGTCC
>n.538.6.bb_922
GTAGTTAGCCGTGGCTTTCTGATTAGGTACCGTCAAGACGTGCATAGTTACTTACACATTTGTTCTTCCCTAATAACAGAGTTTTACGATCCGAAGACCTTCATCACTCACGCGGCGTTGCTCCGTCAGGCTTTCGCCCATTGCGGAAGATTCCCTACTGCTGCCTCCCGTAGGAGTCTGGACCGTGTCTCAGTTCCAGTGTGGCCGATCACCCTCTCAGGGTCGGCTACGCATCGTTGCCTTGGTAAGCCGTTACCTTACCAACTAGCTAATGCGGCGCGGATCCATCTATAAGTGACAGCAAAACCGTCTTTCACTATTGAACCATGCGG
>n.538.6.bb_923
GTAGTTAGCCGTGGCTTTCTGATTAGGTACCGTCAAGACGTGCATAGTTACTTACACATTTATTCTTCCCTAATAACAGAGTTTTACGATCCGAAGACCTTCATCACTCACGCGGCGTTGCTCCGTCAGGCTTTCGCCCATTGCGGAAGATTCCCTACTGCTGCCTCCCGCAGGAGTCTGGACCGTGTCTCAGTTCCAGTGTGGCCGATCACCCTCTCAGGTCGGCTACGCATCGTTGCCTTGGTAAGCCGTTACCTTACCAACTAGCTAATGCGGCGCGGATCCATCTATAAGTGAC
>n.538.6.bb_924
GTAGTTAGCCGGTGCGTCTTCTGCAGGTACCGTCACCACAAGCTTCGCCCCTGCTGAAAGCGGTTTACAACCCGAAGGCCGTCATCCCGCACGCGGCGTTGCTGCATCAGGCTTCCGCCCATTGTGCAATATTCCCCACTGCTGCCTCCCGTAGGAGTCTGGGCCGTATCTCAGTCCCAATGTGGCCGGTCGCCCTCTCAGGCCGGCTACCCGTCAAAGCCTTGGTAAGCCACTACCCCACCAACAAGCTGATAAGCCGCGAGTCCATCCAAAACCGCC
>n.538.6.bb_925
GGAGTTAGCCGGTGCTTCTTCTGCGGGTAACGTCAATTGCTGAGGTTATTAACCTCAACACCTTCCTCCCCGCTGAAAGTACTTTACAACCCGAAGGCCTTCTTCATACACGCGGCATGGCTGCATCAGGCTTGCGCCCATTGTGCAATATTCCCCACTGCTGCCTCCCGTAGGAGTCTGGACCGTGTCTCAGTTCCAGTGTGGCTGGGTCATCCTCTCAGACCAGCTAGGGATCGTCGCCTAGGTGAGCCGTTACCCACCTACTAGCTAATCCCATCTGGGCACA
>n.538.6.bb_926
GTAGTTAGCCGGTGCTTCTTCTGCAGGTACCGTCACCACAAGCTTCGCCCCTGCTGAAAGCGGTTTACAACCCGAAGGCCGTCATCCCGCACGCGGCGTTGCTGCATCAGGCTTCCGCCCATTGTGCAATATTCCCCACTGCTGCCTCCCGTAGGAGTCTGGGCCGTATCTCAGTCCCAATGTGGCCGGTCGCCCTCTCAGGCCGGCTACCCGTCAAAGCCTTGGTAAGCCACTACCCCACCAACAAGCTGATAAGCCGCGAGTCCATCC
>n.538.6.bb_927
GTAGTTAGCCGGTGCTTCTTCTGCAGGTACCGTCACCACAAGCTTCGCCCCTGCTGAAAGCGGTTTACAACCCGAAGGCCGTCATCCCGCACGCGGCGTTGCTGCATCAGGCTTCCGCCCATTGTGCAATATTCCCCACTGCTGCCTCCCGTAGGAGTCTGGGCCGTATCTCAGTCCCAATGTGGCCGGTCGCCCTCTCAGGCCGGCTACCCGTCAAAGCCTTGGTAAGCCACTACCCCACCAACAAGCTGATAAGCCGCGAGTCCATCCAAAACCGCCG
>n.538.6.bb_928
GTAGTTAGCCGTGGCTTTCTGATTAGGTACCGTCAAGACGTGCATAGTTACTTACACATTTGTTCTTCCCTAATAACAGAGTTTTACGATCCGAAGACCTTCATCACTCACGCGGCGTTGCTCCGTCAGGCTTTCGCCCATTGCGGAAGATTCCCTACTGCTGCCTCCCGTAGGAGTCTGGACCGTGTCTCAGTTCCAGTGTGGCCGATCACCCTCTCAGGGTCGGCTACGCATCGTCGCCTTGGTAAGCCGTTACCTTACCAACTAGCTAATGCGGCGCGGATCCATCTATAAGTGACAGCAAAAACCGTCTTTCACTATTGAACCATGCGGTTCAATATATTATCCGGTATTA
>n.538.6.bb_929
GTAGTTAGCCGGTGCTTCTTCTGCAGGTACCGTCACCACAAGCTTCGCCCCTGCTGAAAGCGGTTTACAACCCGAAGGCCGTCATCCCGCACGCGGCGTTGCTGCATCAGGCTTCCGCCCATTGTGCAATATTCCCCACTGCTGCCTCCCGTAGGAGTCTGGGCCGTATCTCAGTCCCAATGTGGCCGGTCGCCCTCTCAGGCCGGCTACCCGGTCAAAGCCTTGGTAAGCCACTACCCACCAACAAGCTGATAAGCCGCGAGTCCAT
>n.538.6.bb_930
GTAGTTAGCCGTGGCTTTCTGATTAGGTACCGTCAAGACGTGCATAGTTACTTACACATTTGTTCTTCCCTAATAACAGAGTTTTACGATCCGAAGACCTTCATCACTCACGCGGCGTTGCTCCGTCAGGCTTTCGCCCATTGCGGAAGATTCCCTACTGCTGCCTCCCGTAGGAGTCTGGACCGTGTCTCAGTTCCAGTGTGGCCGATCACCCTCTCAGGGTCGGCTACGCATCGTTGCCTTGGTAAGCCGTTACCTTACCAACTAGCTAATGCGGCGCGGACCCATCTATAAGTGACAGCAAAACCGTCTTTCACTATTG
>n.538.6.bb_931
GTAGTTAGCCGGTGCTTCTTCTGCAGGTACCGTCACCACAAGCTTCGCCCCTGCTGAAAGCGGTTTACAACCCGAAGGCCGTCATCCCGCACGCGGCGTTGCTGCATCAGGCTTCCGCCCATTGTGCAATATTCCCCACTGCTGCCTCCCGTAGGAGTCTGGGCCGTATCTCAGTCCCAATGTGGCCGGTCGCCCTCTCAGGCCGGCTACCCGTCAAAGCCTTGGTAAGCCACTACCCCACCAACAAGCTGATAAGCCGCGAGTCCATCCAAAACCGCCGAAGCTTTCCAACCCCACCATGCAGCAAGGATTCCTAT
>n.538.6.bb_932
GTAGTTAGCCGGTGCTTCTTCTGCAGGTACCGTCACCACAAGCTTCGCCCCTGCTGAAAGCGGTTTACAACCCGAAGGCCGTCATCCCGCACGCGGCGTTGCTGCATCAGGCTTCCGCCCATTGTGCAATATTCCCCACTGCTGCCTCCCGTAGGAGTCTGGGCCGTATCTCAGTCCCAATGTGGCCGTCGCCCTCTCAGGCCGGCTACCCGGTCAAAGCCTTGGTAACGCCACTACCCCACCAACAAGCTGATAAGCCGCGAGTCCATCCAAAACCGCCGA
>n.538.6.bb_933
GGAGTTAGCCGGTGCTTCTTCTGCGGGTAACGTCAATCGACAAGGTTATTAACCTTATCGCCTTCCTCCCCGCTGAAAGTACTTTACAACCCGAAGGCCTTCTTCATACACGCGGCATGGCTGCATCAGGCTTGCGCCCATTGTGCAATATTCCCCATGCTGCCTCCGTAGGAGTCTGGACCGTGTCTCAGTTCCAGTGTGGCTGGTCATCCTCTCAGACCAGCGTAGGGATCGTCGCCTAGGTGAGCCGTTACCCACCTACTAGCTAATCCCATCTGGGCACATCTGATG
>n.538.6.bb_934
GGAGTTAGCCGGTGCTTCTTCTGCGGGTAACGTCAATCGACAAGGTTATTAACCTTATCGCCTTCCTCCCCGCTGAAAGTACTTTACAACCCGAAGGCCTTCTTCATACACGCGGCATGGCTGCATCAGGCTTGCGCCCATTGTGCAATATTCCCCACTGCTGCCTCCCGTAGGAGTCTGGACCGTGTCTCAGTTCCAGTGTGGCTGGTCATCCTCTCAGACCAGCTAGGGATCGTCGCCTAGGTGAGCCGTTACCCACCTACTAGCTAATCCCATCTGGGCACATCTGATGGC
>n.538.6.bb_935
GTAGTTAGCCGTGGCTTTCTGATTAGGTACCGTCAAGACGTGCATAGTTACTTACACATTTATTCTTCCCTAATAACAGAGTTTTACGATCCGAAGACCTTCATCACTCACGCGGCGTTGCTCCGTCAGGCTTTCGCCCATTGCGGAAGATTCCCTACTGCTGCCTCCCGTAGGAGTCTGGACCGTGTCTCAGTTCCAGTGTGGCCGATCACCCTCTCAGGGTCGGCTACGCATCGTTGCCTTGGTAAGCCGTTACCTTACCAACTAGCTAATGCGGCGCGGATCCATCTATAAGTGACAGCAAAACCGTCTTTCACTATTGAACCATGCGG
>n.538.6.bb_936
GTAGTTAGCCATGGCTTTCTGATTAGGTACCGTCAAGACGTGCATAGTTACTTACACATTTGTTCTTCCCTAATAACAGAGTTTTACGATCCGAAGACCTTCATCACTCACGCGGCGTTGCTCCGTCAGGCTTTCGCCCATTGCGGAAGATTCCCTACTGCTGCCTCCCGTAGGAGTCTGGACCGTGTCTCAGTCCCAGTGTGGCCGATCACCCTCTCAGGGTCGCTACGCATCGTCGCCTTGGTAAGCCGTTACCTTACCAACTAGCTAATGCGGCGCGGATCCATCTATAAGTGACAGCAAAACCGTCTTT
>n.538.6.bb_937
GTAGTTAGCCGTGGCTTTCTGATTAGGTACCGTCAAGACGTGCATAGTTACTTACACATTTGTTCTTCCCTAATAACAGAGTTTTACGATCCGAAGACCTTCATCACTCACGCGGCGTTGCTCCGTCAGGCTTTCGCCCATTGCGGAAGATTCCCTACTGCTGCCTCCCGTAGGAGTCTGGACCGTGTCTCAGTTCCAGTGTGGCCGATCACCCTCTCAGGGTCGGCTACGCATCGTTGCCTTGGTAAGCCGTTACCTTACCAACTAGCTAATGCGGCGCGGATCCATCTATAAGTGACAGCAAAACCGTCTTTCACTATTGAACCATGCGG
>n.538.6.bb_938
GTAGTTAGCCGGTGCTTCTTCTGCAGGTACCGTCACCACAAGCTTCGCCCCTGCTGAAAGCGGTTTACAACCCGAAGGCCGTCATCCCGCACGCGGCGTTGCTGCATCAGGCTTCCGCCCATTGTGCAATATTCCCCACTGCTGCCTCCCGTAGGAGTCTGGGCCGTATCTCAGTCCCAATGTGGCCGGTCGCCCTCTCAGGCCGGCTACCCGTCAAAGCCTTGGTAAGCCACTACCCCACCAACAAGCTGATAAGCCGCGAGTCCATCCAAAACCGCCGAAGCTTTCCAACCCCCACCATGCAGCAAGGATTCCTATCCGGTATTAGCCCCAGTTTCCTGAAGTTATCCCGAAGTCAAGGGCAGGTTAC
>n.538.6.bb_939
GGAGTTAGCCGGTGCTTCTTCTGCGGGTAACGTCAATTGCTGAGGTTATTAACCTCAACACCTTCCTCCCCGCTGAAAGTACTTTACAACCCGAAGGCCTTCTTCATACACGCGGCATGGCTGCATCAGGCTTGCGCCCATTGTGCAATATTCCCCACTGCTGCCTCCCGTAGGAGTCTGGACCGTGTCTCAGTTCCAGTGTGGCTGGGTCATCCTCTCAGACCAGCTAGGGATCGTCGCCTAGGTGAGCCGTTACCCCACCTACTAGCTAATCCCATCTGGGCACATCTGGTGGCAA
>n.538.6.bb_940
GTAGTTAGCCGGTGCTTCTTCTGCAGGTACCGTCACCACAAGCTTCGCCCCTGCTGAAAGCGGTTTACAACCCGAAGGCCGTCATCCCGCACGCGGCGTTGCTGCATCAGGCTTCCGCCCATTGTGCAATATTCCCCACTGCTGCCTCCCGTAGGAGTCTGGGCCGTATCTCAGTCCCAATGTGGCCGGTCGCCCTCTCAGGCCGGCTACCCGTCAAAGCCTTGGTAAGCCACTACCCACCAACAAGCTGATAAGCCGCGAGTCCATCCAAAACCGCC
>n.538.6.bb_941
GTAGTTAGCCGTGGCTTTCTGATTAGGTACCGTCAAGACGTGCATAGTTACTTACACATTTATTCTTCCCTAATAACAGAGTTTTACGATCCGAAGACCTTCATCACTCACGCGGCGTTGCTCCGTCAGGCTTTCGCCCATTGCGGAAGATTCCCTATGCTGCCTCCCGTAGGAGTCTGGACCGTGTCTCAGTTCCAGTGTGGCCGATCACCCTNTCAGGGTCGGCTACGCATCGTTGCCTTGGTAAGCCGTTACCTTACCAACTAGCTAATGCGGCGCGGATCCATCTATAAGTGAC
>n.538.6.bb_942
GTAGTTAGCCGGTGCTTCTTCTGCAGGTACCGTCACCACAAGCTTCGCCCCTGCTGAAAGCGGTTTACAACCCGAAGGCCGTCATCCCGCACGCGGCGTTGCTGCATCAGGCTTCCGCCCATTGTGCAATATTCCCCACTGCTGCCTCCCGTAGGAGTCTGGGCCGTATCCCAGTCCCAATGTGGCCGGTCGCCCTCTCAGGCCGGCTACCCGTCAAAGCCTTGGGTAAGCCACTACCCTACCAACAAGCTGATAAGCCGCGAGTCCATCC
>n.538.6.bb_943
TAGTTAGCCGTGGCTTTCTGATTAGGTACCGTCAAGACGTGCATAGTTACTTACACATTTGTTCTTCCCTAATAACAGAGTTTTACGATCCGAAGACCTTCATCACTCACGCGGCGTTGCTCCGTCAGGCTTTCGCCCATTGCGGAAGATTCCCTACTGCTGCCTCCCGTAGGAGTCTGGACCGTGTCTCAGTTCCAGTGTGGCCGATCACCCTCTCAGGGTCGGGCTACGCATCGTTGCCTTGGTAAGCCGTTACCTTACCAACTAGCTAATGCGGCGCGGATCCATCTATAAGTGACAGCAAAACCGTCTTTCACTAT
>n.538.6.bb_944
GTAGTTAGCCGTGCTTCTTCCTGGCAGTAACCGTCACACAGCTTCGGCCCTGCTAGAAGCGGTTACAACCGAGGCCGTCATCCCGCACGCGGCGTTGCTGCATCAGGCTTCCGCCCATTGTGCAATATTCCCCACTGCTGCCTCCCGTAGGAGTCTGGGCCGTATCTCAGTCCCAATGTGGCCGGTCGCCCTCTCAGGCCGGCTACCCGTCAAAGCCTTGGTAAGCCACTACCCACCAACAAGCTGATAAGCCGCGAGTCCATCCAAAACCGCCGAAGCTTTCCAACCCCACCATGCAGCAAGGATTCCTATCCGGTATTAG
>n.538.6.bb_945
TAGTTAGCCGGTGCTTCTTCTGCAGGTACCGTCACCACAAGCTTCGCCCCTGCTGAAAGCGGTTTACAACCCGAAGGCCGTCATCCCGCACGCGGCGTCGCTGCATCAGGCTTCCGCCCATTGTGCAATATTCCCCACTGCTGCCTCCCGTAGGAGTCTGGGCCGTATCTCAGTCCCAATGTGGCCGGTCGCCCTCTCAGGCCGGCTACCCGGTCAAAGCCTTGGTAAGCCACTACCCCACCAACAAGCTGATAAGCCGCGAGTCCATCCAAAACCGCCGAAG
>n.538.6.bb_946
GTAGTTAGCCGTGGCTTTCTGATTAGGTACCGTCAAGACGTGCATAGTTACTTACACATTTGTTCTTCCCTAATAACAGAGTTTTACGATCCGAAGACCTTCATCACTCACGCGGCGTTGCTCCGTCAGGCTTTCGCCCATTGCGGAAGATTCCCTACTGCTGCCTCCCGTAGGAGTCTGGACCGTGTCTCAGTTCCAGTGTGGCCGATCACCCTCTCAGGGTCGGCTACGCATCGTTGCCTTGGTAAGCCGTTACCTTACCAACTAGCTAATGCGGCGCGGATCCATCTATAAGTGACAGCAAAACCGTCTTTCACTATTGAACCATGCGGTTCAATATATTATCCGGTATTAGCTCCGGTTTCCCGAAGTTATCCCAGTCTTATAGGTAGGTTATCCCACGTGTTACCTCACCCCGTCCCGCCCGCTAACGTCAGAGGGAGCAAGCCTCCCTCGTCTGTTCGCTCGGCTTG
>n.538.6.bb_947
GTAGTTAGCCGGTGCTTCTTCTGCAGGTACCGTCACCACAAGCTTCGCCCCTGCTGAAAGCGGTTTACAACCCGAAGGCCGTCATCCCGCACGCGGCGTTGCTGCATCAGGCTTCCGCCCATTGTGCAATATTCCCCACTGCTGCCTCCCGTAGGAGTCTGGGCCGTATCTCAGTCCCAATGTGCCGGTCGCCCTCTCAGGCCGGCTACCCGTCAAAGCCTTGGTAACGCCAACTACCCCACCAACAAGCTGATAAGCCGCGAGTCCATCCAAAACCGCCGA
>n.538.6.bb_948
GTAGTTAGCCGGTGCTTCTTCTGCAGGTACCGTCACCACAAGCTTCGCCCCTGCTGAAAGCGGTTTACAACCCGAAGGCCGTCATCCCGCACGCGGCGTTGCTGCATCAGGCTTCCGCCCATTGTGCAATATTCCCCACTGCTGCCTCCCGTAGGAGTCTGGGCCGTATCTCAGTCCCAATGTGGCCGGTCGCCCTCTCAGGCCGGCTACCCGTCAAAGCCTTGGTAAGCCACTACCCCACCAACAAGCTGATAAGCCGCGAGTCCATCCAAAACCGCCG
>n.538.6.bb_949
GGAGTTAGCCGGTGCTTCTTCTGCGGGTAACGTCAATCGACAAGGTTATTAACCTTATCGCCTTCCTCCCCGCTGAAAGTACTTTACAACCCGAAGGCCTTCTTCATACACGCGGCATGGCTGCATCAGGCTTGCGCCCATTGTGCAATATTCCCCACTGCTGCCTCCCGTAGGAGTCTGGACCGTGTCTCAGTTCCAGTGTGGCTGGTCATCCTCTCAGACCAGCTAGGGATCGTCGCCTAGGTGAGCCGTTACCCACCTACTAGCTAATCCCATCTGGGCACATCTGATGGCAAGAG
>n.538.6.bb_950
GTAGTTAGCCGGTGCTTCTTCTGCAGGTACCGTCACCACAAGCTTCGCCCCTGCTGAAAGCGGTTTACAACCCGAAGGCCGTCATCCCGCACGCGGCGTTGCTGCATCAGGCTTCCGCCCATTGTGCAATATTCCCCACTGCTGCCTCCCGTAGGAGTCTGGGCCGTATCTCAGTCCCAATGTGGCCGGTCGCCCTCTCAGGGCCGGCTACCCGTCAAAGCCTTGGTAAGCCACTACCCCACCAACAAGCTGATAAGCCGCGAGTCCATCC
>n.538.6.bb_951
GTAGTTAGCCGGTGCTTCTTCTGCAGGTACCGTCACCACAAGCTTCGCCCCTGCTGAAAGCGGTTTACAACCCGAAGGCCGTCATCCCGCACGCGGCGTTGCTGCATCAGGCTTCCGCCCATTGTGCAATATTCCCCATGCTGCCTCCCGTAGGAGTCTGGGCCGTATCTCAGTCCCAATGTGGCCGGTCGCCCTCTCAGGCCGGCTACCCGTCAAAGCCTTGGTAAGCCACTACCCACCAACAAGCTGATAAGCCGCGAGTCCATCCAAAACCGC
>n.538.6.bb_952
GTAGTTAGCCGGTGCTTCTTCTGCAGGTACCGTCACCACAAGCTTCGCCCCTGCTGAAAGCGGTTTACAACCCGAAGGCCGTCATCCCGCACGCGGCGTTGCTGCATCAGGCTTCCGCCCATTGTGCAATATTCCCCACTGCTGCCTCCCGTAGGAGTCTGGGCCGTATCTCAGTCCCAATGTGGCCGGTCGCCCTCTCAGGCCGGCTACCCGGTNAAAGCCTTGGTAACGCCACTACCCCACCAACAAGCTGATAAGCCGCGAGTCCATCCAAAACCGCCG
>n.538.6.bb_953
GGAGTTAGCCGGTGCTTCTTCTGCGGGTAACGTCAATTGCTGAGGTTATTAACCTCTACACCTTCCTCCCGCTAGAAAGTTACTTTACAACCCGAAGGTCCTTTTTCATACACGCGGCATGGCTGCATCAGGCTTGCGCCCATTGTGCAATATTCCCCATGCTGCCTCCGTAGGAGTCTGGACCGTGTCTCAGTTCCAGTGTGGCT
>n.538.6.bb_954
GTAGTTAGCCGTGGCTTTCTGATTAGGTACCATCAAGACGTGCATAGTTACTTACACATTTGTTCTTCCCTAATAACAGAGTTTTACGATCCGAAGACCTTCATCACTCACGCGGCGTTGCTCCGTCAGGCTTTCGCCCATTGCGGAAGATTCCCTACTGCTGCCTCCGTAGGAGTCTGGACCGTGTCTCAGTTCCAGTGTGGCCGATCACCCTCTCAGGGTCGGCTACGACATCGTTGCCTTGGTAAGCCGTTACCTTACCAACTAGCTAATGCGGCGCGGATCCATCTATAAGTGACAGCAAAACCGTCTTTCACTATTGAACCATGCGGTTCAATATATTATCCGGTATTAGCTCCGGT
>n.538.6.bb_955
GTAGTTAGCCGTGGCTTTCTGATTAGGTACCGTCAAGACGTGCATAGTTACTTACACATTTGTTCTTCCCTAATAACAGAGTTTTACGATCCGAAGACCTTCATCACCCACGCGGCGTTGCTCCGTCAGGCTTTCGCCCATTGCGGAAGATTCCCTACTGCTGCCTCCCGTAGGAGTCTGGACCGTGTCTCAGTTCCAGTGTGGCCGATCACCCTCTCAGGGTCGGCTACGCATCGTTGCCTTGGTAAGCCGTTACCTTACCAACTAGCTAATGCGGCGCGGATCCATCTATAAGTGACAGCAAAACCGTCTTTCACTATTGAACCATGCGGTTCAATATATTATCCGGTATTAGCTCCGGTTTCCCCGAAGTTATCCCAGTCTTATAGGTAGGTTA
>n.538.6.bb_956
GTAGTTAGCCGTGGCTTTCTGATTAGGTACCGTCAAGACGTGCATAGTTACTTACACATTTGTTCTTCCCTAATAACAGAGTTTTACGATCCGAAGACCTTCATCACTCACGCGGCGTTGCTCCGTCAGGCTTTCGCCCATTGCGGAAGATTCCCTACTGCTGCCTCCCGTAGGAGTCTGGACCGTGTCTCAGTTCCAGTGTGGCCGATCACCCTCTCAGGTCGGCTACGCATCGTCGCCTTGGTAAGCCGTTACCTTACCAACTAGCTAATGCGGCGCGGATCCATCTATAAGTGACAGCAAAACCGTCTTTCACTATTGAACCATGCGGTTCAATATATT
>n.538.6.bb_957
GTAGTTAGCCGGTGCTTCTTCTGCAGGTACCGTCACCACAAGCTTCGCCCCTGCTGAAAGCGGTTTACAACCCGAAGGCCGTCATCCCGCACGCGGCGTTGCTGCATCAGGCTTCCGCCCATTGTGCAATATTCCCCACTGCTGCCTCCCGTAGGAGTCTGGGCCGTATCTCAGTCCCAATGTGGCCGGTCGCCCTCTCAGGCCGGCTACCCGGTCAAAGCCTTGGTAAGCCACTACCCCACCAACAAGCTGATAAGCCGCGAGTCCATCCAAAACCGC
>n.538.6.bb_958
GTAGTTAGCCGTGGCTTTCTGATTAGGTACCGTCAAGACGTGCATAGTTACTTACACATTTGTTCTTCCCTAATAACAGAGTTTTACGATCCGAAGACCTTCATCACTCACGCGGCGTTGCTCCGTCAGGCTTTCGCCCATTGCGGAAGATTCCCTACTGCTGCCTCCCGTAGGAGTCTGGACCGTGTCTCAGTTCCAGTGTGGCCGATCACCCTCTCAGGGTCGGCTACGCATCGTCGCCTTGGTAAGCCGTTACCTTACCAACTAGCTAAATGCGGCGCGGATCCATCTATAAGTGACAGC
>n.538.6.bb_959
GTAGTTAGCCGTGGCTTTCTGATTAGGTACCGTCAAGACGTGCATAGTTACTTACACATTTGTTCTTCCCTAATAACAGAGTTTTACGATCCGAAGACCTTCATCACTCACGCGGCGTTGCTCCGTCAGGCTTTCGCCCATTGCGGAAGATTCCCTACTGCTGCCTCCCGTAGGAGTCTGGACCGTGTCTCAGTTCCAGTGTGGCCGATCACCCTCTCAGGGTCGGCTACGCATCGTCGCCTTGGTAAGCCGTTACCTTACCAACTAGCTAATGCGGCGCGGATCCATCTATAAGTGACAGCAAAACCGTCTTTCACTAT
>n.538.6.bb_960
GGAGTTAGCCGGTGTTTCTTCTGCGGGTAACGTCAATCGACAGGGTTATTAACCCTGTCGCCTTCCTCCCCGCTGAAAGTACTTTACAACCCGAAGGCCTTCTTCATACACGCGGCATGGCTGCATCAGGCTTGCGCCCATTGTGCAATATTCCCCACTGCTGCCTCCCGTAGGAGTCTGGACCGTGTCTCAGTTCCAGTGTGGCTGGTCATCCTCTCAGACCAGCTAGGGATCGTCGCCTAGGTGAGCCGTTACCCACCTACTAGCCTAATCCCATCTGGGCACATCCGA
>n.538.6.bb_961
GTAGTTAGCCGTGGCTTTCTGATTAGGTACCGTCAAGACGTGCATAGTTACTTACACATTTGTTCTTCCCTAATAACAGAGTTTTACGATCCGAAGACCTTCATCACTCACGCGGCGTTGCTCCGTCAGGCTTTCGCCCATTGCGGAAGATTCCCTACTGCTGCCTCCCGTAGGAGTCTGGACCGTGTCTCAGTTCCAGTGTGGCCGATCACCCTCTCAGGGTCGGCTACGCATCGTTGCCTTGGTAAGCCGTTACCTTACCAACTAGCTAATGCGGCGCGGATCCATCTATAAGTGACAGCAAAACCGTCTTTCACTATTGAACCATGCGGTTCAATATA
>n.538.6.bb_962
GTAGTTAGCCGTGGCTTTCTGATTAGGTACTGTCAAGACGTGCATAGTTACTTACACATTTGTTCTTCCCTAATAACAGAGTTTTACGATCCGAAGACCTTCATCACTCACGCGGCGTTGCTCCGTCAGGCTTTCGCCCATTGCGGAAGATTCCCTACTGCTGCCTCCGTAGGAGTCTGGACCGTGTCTCAGTTCCAGTGTGGCCGATCACCCTCTCGAGGGTCGCTACGCATCGTTGCCTTGGTAAGCCGTTACTTACCAACTAGCTAATGCGGCGCGGATCCATCTATAAGT
>n.538.6.bb_963
GTAGTTAGCCGTGGCTTTCTGATTAGGTACCGTCAAGACGTGCATAGTTACTTACACATTTGTTCTTCCCTAATAACAGAGTTTTACGATCCGAAGACCTTCATCACTCACGCGGCGTTGCTCCGTCAGGCTTTCGCCCATTGCGGAAGATTCCCTACTGCTGCCTCCCGTAGGAGTCTGGACCGTGTCTCAGTTCCAGTGTGGCCGATCACCCTCTCAGGGTCGGCTACGCATCGTTGCCTTGGTAAGCCGTTACCTTACCAACTAGCTAATGCGACGCGGATCCATCTATAAGTGACAGCAAAACCGTCTTTCACTATTGAACCAT
>n.538.6.bb_964
GTAGTTAGCCGGTGCTTCTTCTGCAGGTACCGTCACCACAAGCTTCGCCCCTGCTGAAAGCGGTTTACAACCCGAAGGCCGTCATCCCGCACGCGGCGTTGCTGCATCAGGCTTCCGCCCATTGTGCAATATTCCCCACTGCTGCCTCCCGTAGGAGTCCGGGCCGTATCTCAGTCCCAATGTGGCCGGTCGCCCTCTCAGGCCGGCTACCCGGTCAAAGCCTTGGGTAAGCCACTACCCCACCAACAAGCTGATAAGCCGCGAGTCCATCCAAAACCGCCGAAGC
>n.538.6.bb_965
GTAGTTAGCCGGTGCTTCTTCTGCAGGTACCGTCACCACAAGCTTCGCCCCTGCTGAAAGCGGTTTACAACCCGAAGGCCGTCATCCCGCACGCGGCGTTGCTGCATCAGGCTTCCGCCCATTGTGCAATATTCCCCACTGCTGCCTCCCGTAGGAGTCTGGGCCGTATCTCAGTCCCAATGTGGCCGGTCGCCCTCTCAGGCCGGCTACCCGTCAAAGCCTTGGTAAGCCACTACCCACCAACAAGCTGATAAGCCGCGAGTCC
>n.538.6.bb_966
GTAGTTAGCCGTGGCTTTCTGATTAGGTACCGTCAAGACGTGCATAGTTACTTACACATTTGTTCTTCCCTAATAACAGAGTTTTACGATCCGAAGACCTTCATCACTCACGCGGCGTTGCTCCGTCAGGCTTTCGCCCATTGCGGAAGATTCCCTACTGCTGCCTCCCGTAGGAGTCTGGACCGTGTCTCAGTTCCAGTGTGGCCGATCACCCTCTCAGGGTCGGCTACGCATCGTTGCCTTGGTAAGCCGTTACCTTACCAACTAGCTAATGCGGCGCGGATCCATCTATAAGTGACAGCAAAACCGTCTTTCACTATTGAACCATGCGGTTCAATATATTATCCGGTATTAG
>n.538.6.bb_967
GTAGTTAGCCGGTGCTTCTTCTGCAGGTACCGTCACCACAAGCTTCGCCCCTGCTGAAAGCGGTTTACAACCCGAAGGCCGTCATCCCGCACGCGGCGTTGCTGCATCAGGCTTCCGCCCATTGTGCAATATTCCCCACTGCTGCCTCCCGTAGGAGTCTGGGCCGTATCTCAGTCCCAATGTGGCCGGTCGCCCTCTCAGGCCGGCTACCCGGTCAAAGCCTTGGTAAGTCACTACCCACCAACAAGCTGATAAGCCGCGAGTCCATCCAAAACCGCCGAAGCTTTCCAACCCCACCGTGCAGCAAGGATTCCTATCCGGTAT
>n.538.6.bb_968
GTAGTTAGCCGTGGCTTTCTGATTAGGTACCGTCAAGACGTGCATAGTTACTTACACATTTGTTCTTCCCTAATAACAGAGTTTTACGATCCGAAGACCTTCATCACTCACGCGGCGTTGCTCCGTCAGGCTTTCGCCCATTGCGGAAGATTCCCTACTGCTGCCTCCCGTAGGAGTCTGGACCGTGTCTCAGTTCCAGTGTGGCCGATCACCCTCTCAGGGTCGGCTACGCATCGTTGCCTTGGTAAGCCGTTACCTTACCAACTAGCTAATGCGGCGCGGATCCATCTATAAGTGACAGCAAAACCGTCTTTCACTATTGAACCATGCGGTTCAATATATTATCCGGTATT
>n.538.6.bb_969
GTAGTTAGCCGTGGCTTTCTGATTAGGTACCGTCAAGACGTGCATAGTTACTTACACATTTATTCTTCCCTAATAACAGAGTTTTACGATCCGAAGACCTTCATCACTCACGCGGCGTTGCTCCGTCAGGCTTTCGCCCATTGCGGAAGATTCCCTATGCTGCCTCCCGTAGGAGTCTGGACCGTGTCTCAGTTCCAGTGTGGCCGATCACCC
>n.538.6.bb_970
GGAGTTAGCCGGTGCTTCTTCTGCGGGTAACGTCAATTGCTGAGGTTATTAACCTCAACACCTTCCTCCCCGCTGAAAGTACTTTACAACCCGAAGGCCTTCTTCATACACGCGGCATGGCTGCATCAGGCTTGCGCCCATTGTGCAATATTCCCCACTGCTGCCTCCCGTAGGAGTCTGGACCGTGTCTCAGTTCCAGTGTGGCTGGTCATCCTCTCAGACCAGCTAGGGATCGTCGCCTAGGTGAC
>n.538.6.bb_971
GTAGTTAGCCGTGGCTTTCTGATTAGGTACCGTCAAGACGTGCATAGTTACTTACACATTTGTTCTTCCCTAATAACAGAGTTTTACGATCCGAAGACCTTCATCACTCACGCGGCGTTGCTCCGTCAGGCTTTCGCCCATTGCGGAAGATTCCCTACTGCTGCCTCCCGTAGGAGTCTGGACCGTGTCTCAGTTCCAGTGTGGCCGATCACCCTCTCAGGGTCGGCTACGCATCGTCGCCTTGGTAAGCCGTTACCTTACCAACTAGCTAATGCGGCGCGGATCCATCTATAAGTGACAGCAAAAACCGTCTTTCGCTATTGAACCATGCGGTTCAATATATTATCCGGTATTAGCTCCGGTTTCCCGAAGTTATCCCAGTCTTATAGGTAGGTTA
>n.538.6.bb_972
GTAGTTAGCCGGTGCTTCTTCTGCAGGTACCGTCACCACAAGCTTCGCCCCTGCTGAAAGCGGTTTACAACCCGAAGGCCGTCATCCCGCACGCGGCGTTGCTGCATCAGGCTTCCGCCCATTGTGCAATATTCCCCACTGCTGCCTCCCGTAGGAGTCTGGGCCGTATCTCAGTCCCAATGTGGCCGGTCGCCCTCTCAGGCCGGCTACCCGGTCAAAGGCCTTGGTAACGCCACTACCCCACCAACAAGCTGATAAGCCGCGAGTCCATCCAAAACCGCC
>n.538.6.bb_973
GTAGTTAGCCGGTGCTTCTTCTGCAGGTACCGTCACCACAAGCTTCGCCCCTGCTGAAAGCGGTTTACAACCCGAAGGCCGTCATCCCGCACGCGGCGTTGCTGCATCAGGCTTCCGCCCATTGTGCAATATTCCCCACTGCTGCCTCCCGTAGGAGTCTGGGCCGTATCTCAGTCCCAATGTGGCCGGTCGCCCTCTCAGGCCGGCTACCC
>n.538.6.bb_974
GTAGTTAGCCGGTGCTTCTTCTGCAGGTACCGTCACCACAAGCTTCGCCCCTGCTGAAAGCGGTTTACAACCCGAAGGCCGTCATCCCGCACGCGGCGTTGCTGCATCAGGCTTCCGCCCATTGTGCAATATTCCCCACTGCTGCCTCCCGTAGGAGTCTGGGCCGTATCTCAGTCCCAATGTGGCCGGTCGCCCTCTCAGGCCGGGCTACCCGTCAAAGCCTTGGTAAGCCACTACCCACCAACAAGCTGATAAGCCGCGAGTCCATCC
>n.538.6.bb_975
GTAGTTAGCCGTGGCTTTCTGATTAGGTACCGTCAAGACGTGCATAGTTACTTACACATTTATTCTTCCCTAATAACAGAGTTTTACGATCCGAAGACCTTCATCACTCACGCGGCGTTGCTCCGTCAGGCTTTCGCCCATTGCGGAAGATTCCCTACTGCTGCCTCCCGTAGGAGTCTGGACCGTGTCTCAGTTCCAGTGTGGCCGATCACCCTCTCAGGGTCGGCTACGCATCGTTGCCTTGGTAAGCCGTTACCTTACCAACTAGCTAATGCGGCGCGGATCCATCTATAAGTGACAGCAAAACCGTCTTTCACTATTGAACCATGCGGTT
>n.538.6.bb_976
GTAGTTAGCCGTGGCTTTCTGATTAGGTACCGTCAAGACGTGCATAGTTACTTACACATTTGTTCTTCCCTAATAACAGAGTTTTACGATCCGAAGACCTTCATCACTCACGCGGCGTTGCTCCGTCAGGCTTTCGCCCATTGCGGAAGATTCCCTACTGCTGCCTCCCGTAGGAGTCTGGACCGTGTCTCAGTTCCAGTGTGGCCGATCACCCTCTCAGGGTCGGCTACGCATCGTTGCCTTGGTAAGCCGTTACCTTACCAACTAGCTAATGCGGCGCGGATCCATCTATAAGTGACAGCAAAACCGTCTTTCACTATTGAACCATGCGGTTCAATATATTATCCGGTATT
>n.538.6.bb_977
GTAGTTAGCCGGTGCTTCTTCTGCAGGTACCGTCACCACAAGCTTCGCCCCTGCTGAAAGCGGTTTACAACCCGAAGGCCGTCATCCCGCACGCGGCGTTGCTGCATCAGGCTTCCGCCCATTGTGCAATATTCCCCACTGCTGCCTCCCGTAGGAGTCTGGGCCGTATCTCAGTCCCAATGTGGCCGTCGCCCTCTCAGGCCGGCTACCCGTNAAAGCCTTGGTAAGCCACTACCCACCAACAAGCTGATAAGCCGCGA
>n.538.6.bb_978
GGAGTTAGCCGGTGCTTCTTCTGCGGGTAACGTCAATCGACACGGTTATTAACCGCATCGCCTTCCTCCCCGCTGAAAGTACTTTACAACCCGAAGGCCTTCTTCATACACGCGGCATGGCTGCATCAGGTCTTGCGCCCATTGTGCAATATTCCCCACTGCTGCCTCCCGTAGGAGTCTGGACCGTGTCTCAGTTCCAGTGTGGCTGGGTCATCCTCTCAGACCAGCTAGGGATCGTCGCCTAGGT
>n.538.6.bb_979
GTAGTTAGCCGTGGCTTTCTGATTAGGTACCGTCAAGACGTGCATAGTTACTTACACATTTGTTCTTCCCTAATAACAGAGTTTTACGATCCGAAGACCTTCATCACTCACGCGGCGTTGCTCCGTCAGGCTTTCGCCCATTGCGGAAGATTCCCTACTGCTGCCTCCCGTAGGAGTCTGGACCGTGTCTCAGTTCCAGTGTGGCCGATCACCCTCTCAGGGTCGGCTACGCATCGTCGCCTTGGTAAGCCGTTACCTTACCAACTAGCTAATGCGGCGCGGATCCATCTATAAGTGACAGCAAAACCGTCTTTCACTATTGAACCATGCGG
>n.538.6.bb_980
GTAGTTAGCCGTGGCTTTCTGATTAGGTACCGTCAAGACGTGCATAGTTACTTACACATTTATTCTTCCCTAATAACAGAGTTTTACGATCCGAAGACCTTCATCACTCACGCGGCGTTGCTCCGTCAGGCTTTCGCCCATTGCGGAAGATTCCCTACTGCTGCCTCCCGTAGGAGTCTGGACCGTGTCTCAGTTCCAGTGTGGCCGATCACCCTCTCAGGGTCGGCTACGCATCGTTGCCTTGGTAAGCCGTTACCTTACCAACTAGCTAATGCGGCGCGGATCCATCTATAAGTGACAGCAAAACCGTCTTTCACTA
>n.538.6.bb_981
GTAGTTAGCCGTGGCTTTCTGATTAGGTACCGTCAAGACGTGCATAGTTACTTACACATTTATTCTTCCCTAATAACAGAGTTTTACGATCCGAAGACCTTCATCACTCACGCGGCGTTGCTCCGTCAGGCTTTCGCCCATTGCGGAAGATTCCCTACTGCTGCCTCCCGTAGGAGTCTGGACCGTGTCTCAGTTCCAGTGTGGCCGATCACCCTCTCAGGGTCGGCTACGCATCGTTGCCTTGGTAAGCCGTTACCTTACCAACTAGCTAATGCGGCGCGGATCCATCTATAAGTGACAGCAAAACCGTCTTTCACTATTGAACCATGCGGTTCAATA
>n.538.6.bb_982
GTAGTTAGCCGTGGCTTTCTGATTAGGTACCGTCAAGACGTGCATAGTTACTTACACATTTGTTCTTCCCTAATAACAGAGTTTTACGATCCGAAGACCTTCATCACTCACGCGGCGTTGCTCCGTCAGGCTTTCGCCCATTGCGGAAGATTCCCTACTGCTGCCTCCCGTAGGAGTCTGGACCGTGTCTCAGTTCCAGTGTGGCCGATCACCCTCTCAGGGTCGGCTACGCATCGTTGCCTTGGTAAGCCGTTACCTTACCAACTAGCTAATGCGGCGCGGATCCATCTATAAGTGACAGCAAAACCGTCTTTCACTATTGAACCATGCGGTTCAATATATTATCCGGTATT
>n.538.6.bb_983
GTAGTTAGCCGTGGCTTTCTGATTAGGTACCGTCAAGACGTGCATAGTTACTTACACATTTGTTCTTCCCTAATAACAGAGTTTTACGATCCGAAGACCTTCATCACTCACGCGGCGTTGCTCCGTCAGGCTTTCGCCCATTGCGGAAGATTCCCTACTGCTGCCTCCGTAGGAGTCTGGACCGTGTCTCAGTTCCAGTGTGGCCGATCACCCTNTCAGGGTCGGCTACGCATCGTTGCCTTGGTAAGCCGTTACCTTACCAACTAGCTAATGCGGCGCGGATCCATCTATAAGTG
>n.538.6.bb_984
GTAGTTAGCCGGTGCTTCTTCTGCAGGTACCGTCACCACAAGCTTCGCCCCTGCTGAAAGCGGTTTACAACCCGAAGGCCGTCATCCCGCACGCGGCGTTGCTGCATCAGGCTTCCGCCCATTGTGCAATATTCCCCACTGCTGCCTCCCGTAGGAGTCTGGGCCGTATCTCAGTCCCAATGTGGCCGGTCGCCCTCTCAGGCCGGCTACCCGTCAAAGCCTTGGTAAGCCACTACCCACCAACAAGCTGATAAGCCGCGAGTCCATCCAAAACCGC
>n.538.6.bb_985
GTAGTTAGCCGTGGCTTTCTGATTAGGTACCGTCAAGACGTGCATAGTTACTTACACATTTGTTCTTCCCTAATAACAGAGTTTTACGATCCGAAGACCTTCATCACTCACGCGGCGTTGCTCCGTCAGGCTTTCGCCCATTGCGGAAGATTCCCTACTGCTGCCTCCCGTAGGAGTCTGGACCGTGTCTCAGTTCCAGTGTGGCCGATCACCCTCTCAGGGTCGGCTACGCATCGTTGCCTTGGTAAGCCGTTACCTTACCAACTAGCTAATGCGGCGCGGATCCATCTA
>n.538.6.bb_986
GTAGTTAGCCGTGGCTTTCTGATTAGGTACCGTCAAGACGTGCATAGTTACTTACATATTTGTTCTTCCCTAATAACAGAGTTTTACGATCCGAAGACCTTCATCACTCACGCGGCGTTGCTCCGTCAGGCTTTCGCCCATTGCGGAAGATTCCCTACTGCTGCCTCCCGTAGGAGTCTGGACCGTGTCTCAGTTCCAGTGTGGCCGATCACCCTCTCAGGGTCGGCTACGCATCGTTGCCTTGGTAAGCCGTTACCTTACCAACTAGCTAATGCGGCGCGGATCCATCTATAAGTGACAGCAAAACCGTCTTTCACTATTGAACCATGCGGTTCA
>n.538.6.bb_987
GTAGTTAGCCGTGGCTTTCTGATTAGGTACCGTCAAGACGTGCATAGTTACTTACACATTTGTTCTTCCCTAATAACAGAGTTTTACGATCCGAAGACCTTCATCACTCACGCGGCGTTGCTCCGTCAGGCTTTCGCCCATTGCGGAAGATTCCCTACTGCTGCCTCCCGTAGGAGTCTGGACCGTGTCTCAGTTCCAGTGTGGCCGATCACCCT
>n.538.6.bb_988
GGAGTTAGCCGGTGCTTCTTCTGCGGGTAACGTCAATCGACAAGGTTATTAACCTTATCGCCTTCCTCCCCGCTGAAAGTACTTTACAACCCGAAGGCCTTCTTCATACACGCGGCATGGCTGCATCAGGCTTGCGCCCATTGTGCAATATTCCCCATGCTGCCTCCGTAGGAGTCTGGACCGTGTCTCAGTTCCAGTGTGGCTGGTCATCCTCTCAGACCAGCTAGGGATCGTCGCCTAGGTGAGCCGTTACCCACCTACTAGCTAATCCCATCTGGGCACATCTGATG
>n.538.6.bb_989
GTAGTTAGCCGTGGCTTTCTGATTAGGTACCGTCAAGACGTGCATAGTTACTTACACATTTGTTCTTCCCTAATAACAGAGTTTTACGATCCGAAGACCTTCATCACTCACGCGGCGTTGCTCCGTCAGGCTTTCGCCCATTGCGGAAGATTCCCTACTGCTGCCTCCCGTAGGAGTCTGGACCGTGTCTCAGTTCCAGTGTGGCCGATCACCCTCTCAGGGTCGGCTACGCATTGTTGCCTTGGTAAGCCGTTACCTTACCAACTAGCTAATGCGGCGCGGATCCATCTATAAGTGACAGCAAAACCGTCTTTCACTATTGAACCATGCGGTTCAATATATTATCCGGTATTAGCTCCGGT
>n.538.6.bb_990
GTAGTTAGCCGTGGCTTTCTGATTAGGTACCGTCAAGACGTGCATAGTTACTTACACATTTGTTCTTCCCTAATAACAGAGTTTTACGATCCGAAGACCTTCATCACTCACGCGGCGTTGCTCCGTCAGGCTTTCGCCCATTGCGGAAGATTCCCTACTGCTGCCTCCCGTAGGAGTCTGGACCGTGTCTCAGTTCCAGTGTGGCCGATCACCCTCTCAGGGTCGGCTACGCATCGTCGCCTTGGTAAGCCGTTACCTTACCAACTAGCTAATGCGGCGCGGATCCATCTATAAGTGACAGCAAAACCGTCTTTCACTATTGAACCATGCGGTTCAATATATTATCCGGTATT
>n.538.6.bb_991
GTAGTTAGCCGTGGCTTTCTGATTAGGTACCGTCAAGACGTGCATAGTTACTTACACATTTGTTCTTCCCTAATAACAGAGTTTTACGATCCGAAGACCTTCATCACTCACGCGGCATTGCTCCGTCAGGCTTTCGCCCATTGCGGAAGATTCCCTACTGCTGCCTCCCGTAGGAGTCTGGACCGTGTCTCAGTTCCAGTGTGGCCGATCACCCTNTCAGGTCGGCTACGCATCGTTGCCTTGGTAAGCCGTTACCTTACCAACTAGCTAATGCGGCGCGGATCCATCTATAAGTGACAGCAAAACCGTCTTTCACTATTGAACCATGCGGTTCAATATATT
>n.538.6.bb_992
GTAGTTAGCCGGTGCTTCTTCTGCAGGTACCGTCACCACAAGCTTCGCCCCTGCTGAAAGCGGTTTACAACCCGAAGGCCGTCATCCCGCACGCGGCGTTGCTGCATCAGGCTTCCGCCCATTGTGCAATATTCCCCACTGCTGCCTCCCGTAGGAGTCTGGGCCGTATCTCAGTCCCAATGTGGCCGGTCGCCCTCTCAGGCCGGCTACCCGGTCAAAGCCTTGGTAAGCCACTACCCCACCAACAAGCTGATAAGCCGCGAGTCCATCCAAAACCGC
>n.538.6.bb_993
GTAGTTAGCCGTGGCTTTCTGATTAGGTACCGTCAAGACGTGCATAGTTACTTACACATTTGTTCTTCCCTAATAACAGAGTTTTACGATCCGAAGACCTTCATCACTCACGCGGCGTTGCTCCGTCAGGCTTTCGCCCATTGCGGAAGATTCCCTACTGCTGCCTCCCGTAGGAGTCTGGACCGTGTCTCAGTTCCAGTGTGGCCGATCACCCTCTCAGGGTCGGCTACGCATCGTTGCCTTGGTAAGCCGTTACCTTACCAACTAGCTAATGCGGCGCGGATCCATCTATAAGTGACAGCAAAACCGTCTTTCACTATTGAACCATGCGGTTCAATATATTATCCGGTAT
>n.538.6.bb_994
GTAGTTAGCCGTGGCTTTCTGATTAGGTACCGTCAAGACGTGCATAGTTACTTACACATTTGTTCTTCCCTAATAACAGAGTTTTACGATCCGAAGACCTTCATCACTCACGCGGCGTTGCTCCGTCAGGCTTTCGCCCATTGCGGAAGATTCCCTACTGCTGCCTCCCGTAGGAGTCTGGACCGTGTCTCAGTTCCAGTGTGGCCGATCACCCTCTCAGGGTCGGCTACGCATCGTTGCCTTGGTAAGCCGTTACCTTACCAACTAGCTAATGCGGCGCGGATCCATCTATAAGTGACAGCAAAACCGTCTTTCACTATTGAACCATGCGGTTCAATATATTATCCGGTATT
>n.538.6.bb_995
GTAGTTAGCCGTGGCTTTCTGATTAGGTACCGTCAAGACGTGCATAGTTACTTACACATTTGTTCTTCCCTAATAACAGAGTTTTACGATCCGAAGACCTTCATCACTCACGCGGCGTTGCTCCGTCAGGCTTTCGCCCATTGCGGAAGATTCCCTACTGCTGCCTCCCGTAGGAGTCTGGACCGTGTCTCAGTTCCAGTGTGGCCGATCACCCTCTCAGGGTCGGCTACGCATCGTTGCCTTGGTAAGCCGTTACCTTACCAACTAGCTAATGCGGCGCGGATCCATCTATAAGTGACAGCAAAACCGTCTTTCACTATTGAACCATGCGGTTCAATATATTATCCGGTATTAGCTCCGGTTTCCCGAAGTTATCCCAGTCTTATAGGTAGGTTATCCCACGTGTTACCTCCACCCCGTCCCGCCCGCTAACGTCAGAGGGAGCAAGCCTCCCTCGTCCTGTTCC
>n.538.6.bb_996
GTAGTTAGCCGGTGCTTCTTCTGCAGGTACCGTCACCACAAGCTTCGCCCTGCTGAAGCGGTTTACAACCCGAAGGCCGTCATCCCGCACGCGGCGTTGCTGCATCAGGCTTCCGCCCATTGTGCAATATTCCCCACTGCTGCCTCCCGTAGGAGTCTGGGCCGTATCTCAGTCCCAATGTGGCCGGTCGCCCTCTCAGGCCGGCTA
>n.538.6.bb_997
GTAGTTAGCCGTGGCTTTCTGATTAGGTACCGTCAAGACGTGCATAGTTACTTACACATTTGTTCTTCCCTAATAACAGAGTTTTACGATCCGAAGACCTTCATCACTCACGCGGCGTTGCTCCGTCAGGCTTTCGCCCATTGCGGAAGATTCCCTACTGCTGCCTCCCGTAGGAGTCTGGACCGTGTCTCAGTTCCAGTGTGGCCGATCACCCTNTCGGGGGTCGGCTACGCATCGTTGCCTTGGTAAGCCGTTACCTTACCAACTAGCTAATGCGGCGCGGATCCATCTATAAGTGACAGCAAAACCGTCTTTCACTATTGAACCATGCGGTTCAATATATTATCCGGTATT
>n.538.6.bb_998
TAGTTAGCCGTGGCTTTCTGATTAGGTACCGTCAAGACGTGCATAGTTACTTACACATTTGTTCTTCCCTAATAACAGAGTTTTACGATCCGAAGACCTTCATCACTCACGCGGCGTTGCTCCGTCAGGCTTTCGCCCATTGCGGAAGATTCCCTACTGCTGCCTCCCGTAGGAGTCTGGACCGTGTCTCAGTTCCAGTGTGGCCGATCACCCTCTCAGGGTCGGCTACGCATCGTTGCCTTGGTAAGCCGTTACCTTACCAACTAGCTAATGCGGCGCGGATCCATCTATAAGTGA
>n.538.6.bb_999
GTAGTTAGCCGGTGCTTCTTCTGCAGGTACCGTCACCACAAGCTTCGCCCCTGCTGAAAGCGGTTTACAACCCGAAGGCCGTCATCCCGCACGCGGCGTTGCTGCATCAGGCTTCCGCCCATTGTGCAATATTCCCCACTGCTGCCTCCCGTAGGAGTCTGGGCCGTATCTCAGTCCCAATGTGGCCGGTCGCCCTCTCAGGCCGGCTACCCGTCAAAGCCTTGGTAAGCCACTACCCCACCAACAAGCTGATAAGCCGCGAGTCCATCCAAAACCGCCGA
>n.538.6.bb_1000
GTAGTTAGCCGGTGCTTCTTCTGCAGGTACCGTCACCACAAGCTTCGCCCCTGCTGAAAGCGGTTTACAACCCGAAGGCCGTCATCCCGCACGCGGCGTTGCTGCATCAGGCTTCCGCCCATTGTGCAATATTCCCCACTGCTGCCTCCCGTAGGAGTCTGGGCCGTATCTCAGTCCCAATGTGGCCGGTCGCCCTCTCGAGGCCCGGCTACCCGTCAAAGCCTTGGTAACGCCACTACCCCACCAACAAGCTGATAAGCCGCGAGTCCATCCAAAACCGC
>n.538.6.bb_1001
GGAGTTAGCCGGTGCTTCTTCTGCGGGTAACGTCAATTGCTGAGGTTATTAACCTCAACACCTTCCTCCCCGCTGAAAGTACTTTACAACCCGAAGGCCTTCTTCATACACGCGGCATGGCTGCATCAGGCTTGCGCCCATTGTGCAATATTCCCCACTGCTGCCTCCCGTAGGAGTCTGGACCGTGTCTCAGTTCCAGTGTGGCTGGGTCATCCTCTCAGACCAGCTAGGGATCGTCGCCTAGGTGAGCCGTTACCCACCTACTAGCTAATCCCATCTGGGCACATCTGATGGC
>n.538.6.bb_1002
GTAGTTAGCCGTGGCTTTCTGATTAGGTACCGTCAAGACGTGCATAGTTACTTACACATTTGTTCTTCCCTAATAACAGAGTTTTACGATCCGAAGACCTTCATCACTCACGCGGCGTTGCTCCGTCAGGCTTTCGCCCATTGCGGAAGATTCCCTACTGCTGCCTCCCGTAGGAGTCTGGACCGTGTCTCAGTTCCAGTGTGGCCGATCACCCTCTCAGGGTCGGCTACGCATCGTTGCCTTGGTAAGCCGTTACCTTACCAACTAGCTAATGCGGCGCGGATCCATCTATAAGTGACAGCAAAACCGTCTTTCACTATTGAACCATGCGG
>n.538.6.bb_1003
GTAGTTAGCCGTGGCTTTCTGATTAGGTACCGTCAAGACGTGCATAGTTACTTACACATTTGTTCTTCCCTAATAACAGAGTTTTACGATCCGAAGACCTTCATCACTCACGCGGCGTTGCTCCGTCAGGCTTTCGCCCATTGCGGAAGATTCCCTACTGCTGCCTCCCGTAGGAGTCTGGACCGTGTCTCAGTTCCAGTGTGGCCGATCACCCTCTCAGGGTCGGCTACGCATCGTCGCCTTGGTAAGCCGTTACCTTACCAACTAGCTAATGCGGCGCGGATCCATCTATAAGTGACAG
>n.538.6.bb_1004
GTAGTTAGCCGGTGCTTCTTCTGCAGGTACCGTCACCACAAGCTTCGCCCCTGCTGAAAGCGGTTTACAACCCGAAGGCCGTCATCCCGCACGCGGCGTTGCTGCATCAGGCTTCCGCCCATTGTGCAATATTCCCCACTGCTGCCTCCCGTAGGAGTCTGGGCCGTATCTCAGTCCCAATGTGGCCGGTCGCCCTCTCAGGCCGGCTACCCGTCAAAGCCTTGGTAAGCCACTACCCCACCAACAAGCTGATAAGCCGCGAGTCCATCCAAAACCGCCGAAGCTTTCCAACCCCCACCATGCAGCAAGGATTCCTATCCGGTATTAGCCCCAGTTTCCTGAAGTTATCCCGAAGTCAAGGGCAGGTTACTCACGTGTT
>n.538.6.bb_1005
GGAGTTAGCCGGTGCTTCTTCTGCGGGTAACGTCAATTGCTGAGGTTATTAACCTCAACACCTTCCTCCCCGCTGAAAGTACTTTACAACCCGAAGGCCTTCTTCATACACGCGGCATGGCTGCATCAGGCTTGCGCCCATTGTGCAATATTCCCCACTGCTGCCTCCCGTAGGAGTCTGGACCGTGTCTCAGTTCCAGTGTGGCTGGGTCATCCTCTCAGACCAGCTAGGGATCGTCGCCTAGGTGAGCCGTTACCCACCTACCAGCTAATCCCATCTGGGCACATCTGATGGCATGA
>n.538.6.bb_1006
GTAGTTAGCCGTGGCTTTCTGATTAGGTACCGTCAAGACGTGCATAGTTACTTACACATTTGTTCTTCCCTAATAACAGAGTTTTACGATCCGAAGACCTTCATCACTCACGCGGCGTTGCTCCGTCAGGCTTTCGCCCATTGCGGAAGATTCCCTACTGCTGCCTCCCGTAGGAGTCTGGACCGTGTCTCAGTTCCAGTGTGGCCGATCACCCTCTCAGGGTCGGCTACGCATCGTTGCCTTGGTAAGCCGTTACCTTACCAACTAGCTAATGCGGCGCGGATCCATCTATAAGTGACAGCAAAACCGTCTTTCACTATTGAACCATGCGG
>n.538.6.bb_1007
GTAGTTAGCCGTGGCTTTCTGATTAGGTACCGTCAAGACGTGCATAGTTACTTACACATTTGTTCTTCCCTAATAACAGAGTTTTACGATCCGAAGACCTTCATCACTCACGCGGCGTTGCTCCGTCAGGCTTTCGCCCATTGCGGAAGATTCCCTACTGCTGCCTCCCGTAGGAGTCTGGACCGTGTCTCAGTTCCAGTGTGGCCGATCACCCTCTCAGGGTCGGCTACGCATCGTTGCCTTGGTAAGCCGTTACCTTACCAACTAGCTAATGCGGCGCGGATCCATCTATAAGTGACAGCAAAACCGTCTTTCACTATTGAACCATGCGGTTCAATATATTATCCGGTATTA
>n.538.6.bb_1008
GTAGTTAGCCGTGGCTTTCTGATTAGGTACCGTCAAGACGTGCATAGTTACTTACACATTTGTTCTTCCCTAATAACAGAGTTTTACGATCCGAAGACCTTCATCACTCACGCGGCGTTGCTCCGTCAGGCTTTCGCCCATTGCGGAAGATTCCCTACTGCTGCCTCCCGTAGGAGTCTGGACCGTGTCTCAGTTCCAGTGTGGCCGATCACCCTCTCAGGGTCGGCTACGCATCGTCGCCTTGGTAAGCCGTTACCTTACCAACTAGCTAATGCGGCGCGGATCCATCCATAAGTGACAGC
>n.538.6.bb_1009
GTAGTTAGCCGGTGCTTCTTCTGCAGGTACCGTCACCACAAGCTTCGCCCCTGCTGAAAGCGGTTTACAACCCGAAGGCCGTCATCCCGCACGCGGCGTTGCTGCATCAGGCTTCCGCCCATTGTGCAATATTCCCCACTGCTGCCTCCCGTAGGAGTCTGGGCCGTATCTCAGTCCCAATGTGGCCGGTCGCCCTCTCAGGCCGGCTACCCGTNAAAGCCTTGGTAACGCCACTACCCCACCAACAAGCTGATAAGCCGCGAGTCCATCCAAAACCGCCGAAG
>n.538.6.bb_1010
GTAGTTAGCCGTGGCTTTCTGATTAGGTACCGTCAAGACGTGCATAGTTACTTACACATTTGTTCTTCCCTAATAACAGAGTTTTACGATCCGAAGACCTTCATCACTCACGCGGCGTTGCTCCGTCAGGCTTTCGCCCATTGCGGAAGATTCCCTACTGCTGCCTCCCGTAGGAGTCTGGACCGTGTCTCAGTTCCAGTGTGGCCGATCACCCTCTCAGGGTCGGCTACGCATCGTTGCCTTGGTAAGCCGTTACCTTACCAACTAGCTAATGCGGCGCGGATCCATCTATAAGTGACAGC
>n.538.6.bb_1011
GTAGTTAGCCGTGGCTTTCTGATTAGGTACCGTCAAGACGTGCATAGTTACTTACACATTTATTCTTCCCTAATAACAGAGTTTTACGATCCGAAGACCTTCATCACTCACGCGGCGTTGCTCCGTCAGGCTTTCGCCCATTGCGGAAGATTCCCTACTGCTGCCTCCCGTAGGAGTCTGGGCCGTATCTCAGTCCCAATGTGGCTGGTCGCCCTCTCAGGGCCGGCTACCCGTCAAAGCCTTGGTAAGCCACTACCCCACCAACAAGCTGATAAGCCGCGAGTCCATCCAAAACCGCCGAAGCTTTCC
>n.538.6.bb_1012
GGAGTTAGCCGGTGCTTCTTCTGCGGGTAACGTCAATCGACAAGGTTATTAACCTTATCGCCTTCCTCCCCGCTGAAAGTACTTTACAACCCGAAGGCCTTCTTCATACACGCGGCATGGCTGCATCAGGCTTGCGCCCATTGTGCAATATTCCCCACTGCTGCCTCCGTAGGAGTCTGGACCGTGTCTCAGTTCCAGTGTGGCTGGTCATCCTCTCAGACCAGCTAGGGATCGTCGCCTAGGTGAGCCGTTACCCACCTACTAGCTAATCCCATCTGGGCACATCCGATG
>n.538.6.bb_1013
GTAGTTAGCCGTGGCTTTCTGATTAGGTACCGTCAAGACGTGCATAGTTACTTACACATTTGTTCTTCCCTAATAACAGAGTTTTACGATCCGAAGACCTTCATCACTCACGCGGCGTTGCTCCGTCAGGCTTTCGCCCATTGCGGAAGATTCCCTACTGCTGCCTCCCGTAGGAGTCTGGACCGTGTCTCAGTTCCAGTGTGGCCGATCACCCTCTCAGGGTCGGCTACGCATCGTTGCCTTGGTAAGCCGTTACCTTACCAACTAGCTAATGCGGCGCGGATCCATCTATAAGTGACAGCAAAACCGTCTTTCACTATTGAACCATGCGGTTCAATATAT
>n.538.6.bb_1014
GTAGTTAGCCGTGGCTTTCTGATTAGGTACCGTCAAGACGTGCATAGTTACTTACACATTTGTTCTTCCCTAATAACAGAGTTTTACGATCCGAAGACCTTCATCACTCACGCGGCGTTGCTCCGTCAAGCTTTCGCCCATTGCGGAAGATTCCCTACTGCTGCCTCCCGTAGGAGTCTGGACCGTGTCTCAGTTCCAGTGTGGCCGATCACCCTCTCAGGGTCGGCTACGCATCGTTGCCTTGGTAAGCCGTTACCTTACCAACTAGCTAATGCGGCGCGGATCCATCTATAAGTGACAGCAAAACCGTCTTTCACTATTGAACCATGCGG
>n.538.6.bb_1015
GTAGTTAGCCGTGGCTTTCTGATTAGGTACCGTCAAGACGTGCATAGTTACTTACACATTTGTTCTTCCCTAATAACAGAGTTTTACGATCCGAAGACCTTCATCACTCACGCGGCGTTGCTCCGTCAGGCTTTCGCCCATTGCGGAAGATTCCCTACTGCTGCCTCCCGTAGGAGTCTGGACCGTGTCTCAGTTCCAGTGTGGCCGATCACCCTCTCAGGGTCGGCTACGCATCGTTGCCTTGGTAAGCCGTTACCTTACCAACTAGCTAATGCGGCGCGGATCCATCTATAAGTGACAGCAAAACCGTCTTTCACTATTGAACCATGCGGTTC
>n.538.6.bb_1016
GTAGTTAGCCGGTGCTTCTTCTGCAGGTACCGTCACCACAAGCTTCGCCCCTGCTGAAAGCGGTTTACAACCCGAAGGCCGTCATCCCGCACGCGGCGTTGCTGCATCAGGCTTCCGCCCATTGTGCAATATTCCCCACTGCTGCCTCCCGTAGGAGTCTGGGCCGTATCTCAGTCCCAATGTGGCCGGTCGCCCTCTCAGGCCGGCTACCCGGTCAAAGCCTTGGTAAGCCACTACCCCACCAACAAGCTGATAAGCCGCGAGTCCATCCAAAACCGC
>n.538.6.bb_1017
GTAGTTAGCCGTGGCTTTCTGATTAGGTACCGTCAAGACGTGCATAGTTACTTACACATTTGTTCTTCCCTAATAACAGAGTTTTACGATCCGAAGACCTTCATCACTCACGCGGCGTTGCTCCGTCAAGCTTTCGCCCATTGCGGAAGATTCCCTACTGCTGCCTCCCGTAGGAGTCTGGACCGTGTCTCAGTTCCAGTGTGGCCGATCACCCTCTCAGGGTCGGCTACGCATCGTCGCCTTGGTAAGCCGTTACCTTACCAACTAGCTAATGCGGCGCGGATCCATCTATAAGTGACAGCAAAACCGTCTTTCACTATTGAACCATGCGGTTCAATATATTATCCGGTATTA
>n.538.6.bb_1018
GTAGTTAGCCGGTGCTTCTTCTGCAGGTACCGTCACCACAAGCTTCGCCCCTGCTGAAAGCGGTTTACAACCCGAAGGCCGTCATCCCGCACGCGGCGTTGCTGCATCAGGCTTCCGCCCATTGTGCAATATTCCCACTGCTGCCTCCCGTAGGAGTCTGGGCCGTATCTCAGTCCCAATGTGGCCGGTCGCCCTCTCAGGCCGGCTACCCGTCAAAGCCTTGGTAAGCCACTACCCCACCAACAAGCTGATAAGCCGCGAGTCCATCC
>n.538.6.bb_1019
GTAGTTAGCCGGTGCTTCTTCTGCAGGTACCGTCACCACAAGCTTCGCCCCTGCTGAAAGCGGTTACAACCCGAAGGCCGTCATCCCGCACGCGGCGTTGCTGCGTCAGGCTTCCGCCCATTGTGCAATATTCCCCACTGCTGCCTCCCGTAGGAGTCTGGGCCGTATCTCAGTCCCAATGTGGCCGGTCGCCCTCTCAGG
>n.538.6.bb_1020
GTAGTTAGCCGTGGCTTTCTGATTAGGTACCGTCAAGACGTGCATAGTTACTTACACATTTATTCTTCCCTAATAACAGAGTTTTACGATCCGAAGACCTTCATCACTCACGCGGCGTTGCTCCGTCAGGCTTACGCCCATTGCGGAAGATTCCCTACTGCTGCCTCCCGTAGGAGTCTGGACCGTGTCTCAGTTCCAGTGTGGCCGATCACCCTCTCAGGGTCGGCTACGCATCGTTGCCTTGTAAGCCGTTACCTTACCAACTAGCTAATGCGGCGCGGATCCATCTATAAGTGACAGCAAAACCGTCTTTCACTATTGAACCATGCGGTTCAATATA
>n.538.6.bb_1021
GTAGTTAGCCGTGGCTTTCTGATTAGGTACCGTCAAGACGTGCATAGTTACTTACACATTTGTTCTTCCCTAATAACAGAGTTTTACGATCCGAAGACCTTCATCACTCACGCGGCGTTGCTCCGTCAGGCTTTCGCCCATTGCGGAAGATTCCCTACTGCTGCCTCCCGTAGGAGTCTGGACCGTGTCTCAGTTCCAGTGTGGCCGATCACCCTCTCAGGGTCGGCTACGCATCGTCGCCTTGGTAAGCCGTTACCTTACCAACTAGCTAATGCGGCGCGGATCCATCTATAAGTGACAGCAAAACCGTCTTTCAC
>n.538.6.bb_1022
GTAGTTAGCCGTGGCTTTCTGATTAGGTACCGTCAAGACGTGCATAGTTACTTACACATTTGTTCTTCCCTAATAACAGAGTTTTACGATCCGAAGACCTTCATCACTCACGCGGCGTTGCTCCGTCAGGCTTTCGCCCATTGCGGAAGATTCCCTACTGCTGCCTCCCGTAGGAGTCTGGACCGTGTCTCAGTTCCAGTGTGGCCGATCACCCTCTCAGGGTCGGCTACGCATCGTTGCCTTGGTAAGCCGTTACCTTACCAACTAGCTAATGCGGCGCGGATCCATCTATAAGTGACAGCAAAACCGTCTTTCACTATTGACCATGCGGTCAATATATTATCCGG
>n.538.6.bb_1023
GGAGTTAGCCGGTGCTTCTTCTGCGGGTAACGTCAATCGAATGAGGTTATTAACCTCACCGCCTTCCTCCCCGCTGAAAGTGCTTTACAACCCGAAGGCCTTCTTCACACACGCGGCATGGCTGCATCAGGCTTGCGCCCATTGTGCAATATTCCCCACTGCTGCCTCCCGTAGGNGTCTGGACCGTGTCTCAGTTCCAGTGTGGCTGGGTCATCCT
>n.538.6.bb_1024
GTAGTTAGCCGTGGCTTTCTGATTAGGTACCGTCAAGACGTGCATAGTTACTTACACATTTGTTCTTCCCTAATAACAGAGTTTTACGATCCGAAGACCTCCATCACTCACGCGGCGTTGCTCCGTCAGGCTTTCGCCCATTGCGGAAGATTCCCTACTGCTGCCTCCCGTAGGAGTCTGGACCGTGTCTCAGTTCCAGTGTGGCCGATCACCCTCTCAGGGTCGGCTACGCATCGTTGCCTTGGTAAGCCGTTACCTTACCAACTAGCTAATGCGGCGCGGATCCATCTATAAGTGACAGCAAAACCGTCTTTCACTATTGAACCATGCGGTTCAATATATTATCCGGTATT
>n.538.6.bb_1025
GTAGTTAGCCGTGGCTTTCTGATTAGGTACCGTCAAGACGTGCATAGTTACTTACACATTTGTTCTTCCCTAATAACAGAGTTTTACGATCCGAAGACCTACATCACTCACGCGGCGTTGCTCCGTCAGGCTTTCGCCCATTGCGGAAGATTCCCTACTGCTGCCTCCCGTAGGAGTCTGGACCGTGTCTCAGTTCCAGTGTGGCCGATCACCCTCTCAGGGTCGGCTACGCATCGTCGCCTTGGTAAGCCGTTACCTTACCAACTAGCTAATGCGGCGCGGATCCATCTATAAGTGAC
>n.538.6.bb_1026
GTAGTTAGCCGTGGCTTTCTGATTAGGTACCGTCAAGACGTGCATAGTTACTTACACATTTGTTCTTCCCTAATAACAGAGTTTTACGATCCGAAGACCTTCATCACTCACGCGGCGTTGCTCCGTCAAGCTTTCGCCCATTGCGGAAGATTCCCTACTGCTGCCTCCCGTAGGAGTCTGGACCGTGTCTCAGTTCCAGTGTGGCCGATCACCCTCTCAGGGTCGGCTACGCATCGTTGCCTTGGTAAGCCGTTACCTTACCAACTAGCTAATGCGGCGCGGATCCATCTATAAGTGAC
>n.538.6.bb_1027
GTAGTTAGCCGGTGCTTCTTCTGCAGGTACCGTCACCACAAGCTTCGCCCCTGCTGAAAGCGGTTTACAACCCGAAGGCCGTCATCCCGCACGCGGCGTTGCTGCATCAGGCTTCCGCCCATTGTGCAATATTCCCCACTGCTGCCTCCCGTAGGAGTCTGGACCGTGTCTCAGTTCCAGTGTGGCTGGTCATCCTCTCAGGACCAGC
>n.538.6.bb_1028
GTAGTTAGCCGTGGCTTTCTGATTAGGTACCGTCAAGACGTGCATAGTTACTTACACATTTATTCTTCCCTAATAACAGAGTTTTACGATCCGAAGACCTTCATCACTCACGCGGCGTTGCTCCGTCAGGCTTTCGCCCATTGCGGAAGATTCCCTACTGCTGCCTCCCGTAGGAGTCTGGACCGTGTCTCAGTTCCAGTGTGGCCGGTCGCCCTCTCAGGGCCGGGCTACCCCGTCAAAGCCTTGGTAAGCCACTACCCACCAACAAGCTGATAAGCCGCGAGTCCAT
>n.538.6.bb_1029
GTAGTTAGCCGGTGCTTCTTCTGCAGGTACCGTCACCACAAGCTTCGCCCCTGCTGAAAGCGGTTTACAACCCGAAGGCCGTCATCCCGCACGCGGCGTTGCTGCATCAGGCTTCCGCCCATTGTGCAATATTCCCCACTGCTGCCTCCCGTAGGAGTCTGGGCCTATCTCAGTCCCAATGTGGCCGGTCGCCCTCTCGAGGCCGGCTACCC
>n.538.6.bb_1030
GTAGTTAGCCGTGGCTTTCTGATTAGGTACCGTCAAGACGTGCATAGTTACTTACACATTTGTTCTTCCCTAATAACAGAGTTTTACGATCCGAAGACCTTCATCACTCACGCGGCGTTGCTCCGTCAAGCTTTCGCCCATTGCGGAAGATTCCCTACTGCTGCCTCCCGTAGGAGTCTGGACCGTGTCTCAGTTCCAGTGTGGCCGAT
>n.538.6.bb_1031
GTAGTTAGCCGTGGCTTTCTGATTAGGTACCGTCAAGACGTGCATAGTTACTTACACATTTGTTCTTCCCTAATAACAGAGTTTTACGATCCGAAGACCTTCATCACTCACGCGGCGTTGCTCCGTCAGGCTTTCGCCCATTGCGGAAGATTCCCTACTGCTGCCTCCCGTAGGAGTCTGGACCGTGTCTCAGTTCCAGTGTGGCCGATCACCCTCTCAGGGTCGGCTACGCATCGTTGCCTTGGTAAGCCGTTACCTTACCACTAGCTAATGCGGCGCGGATCCATCTATAAGGTGACAGCAAAACCGTCTTTCACTATTGAAC
>n.538.6.bb_1032
GAAGTTAGCCGGTGCTTCTTCTGCGGGTAACGTCAATCGACAAGGTTATTAACCTTATCGCCTTCCTCCCCGCTGAAAGTACTTTACAACCCGAAGGCCTTCTTCATACACGCGGCATGGCTGCATCAGGCTTGCGCCCATTGTGCAATATTCCCCACTGCTGCCTCCCGTAGGAGTCTGGACCGTGTCTCGGTTCCAGTGTGGCTGGTCATCCCCTNAGACCAGCTAGGGGATCGTCGCCTAGGTGAGCCGTTACCCACCTACTAGCTAATCCATCTGGGCACATCTGAT
>n.538.6.bb_1033
GTAGTTAGCCGTGGCTTTCTGATTAGGTACCGTCAAGACGTGCATAGTTACTTACACATTTGTTCTTCCCTAATAACAGAGTTTTACGATCCGAAGACCTTCATCACTCACGCGGCGTTGCTCCGTCAGGCTTTCGCCCATTGCGGAAGATTCCCTACTGCTGCCTCCCGTAGGAGTCTGGACCGTGTCTCAGTTCCAGTGTGGCCGATCACCCTCTCAGGGTCGGCTACGCATCGTTGCCTTGGTAAGCCGTTACCTTACCAACTAGCTAATGCGGCGCGGATCCATCTATAAGTGACAGCAAAACCGTCTTTCACTATTGAACCATGC
>n.538.6.bb_1034
GGCGTTAGCCGGTGCTTCTTCTGCGGGTAACGTCAATTGCTGAGGTTATTAACCTCAACACCTTCCTCCCCGCTGAAAGTACTTTACAACCCGAAGGCCTTCTTCATACACGCGGCATGGCTGCATCAGGCTTGCGCCCATTGTGCAATATTCCCCACTGCTGCCTCCCGTAGGAGTCTGGACCGTGTCTCAGTTCCAGTGTGGCTGGGTCATCCTCTCAGACCAGCTAGGGATCGTCGCCTAGGTGAGCCGTTACCCCACCTACTAGCTAATCCCATCTGGGCACATCTGATGGCAAG
>n.538.6.bb_1035
GTAGTTAGCCGTGGCTTTCTGATTAGGTACCGTCAAGACGTGCATAGTTACTTACACATTTGTTCTTCCCTAATAACAGAGTTTTACGATCCGAAGACCTTCATCACTCACGCGGCGTTGCTCCGTCAGGCTTTCGCCCATTGCGGAAGATTCCCTACTGCTGCCTCCCGTAGGAGTCTGGACCGTGTCTCAGTTCCAGTGTGGCCGATCACCCTCTCAGGGTCGGCTACGCATCGTTGCCTTGGTAAGCCGTTACCTTACCAACTAGCTAATGCGGCGCGGATCCATCTATAAGTGACAGCAAAACCGTCTTTCACTATTGAACCATGCGGTTCAATATATTATCCGGTATTAGCT
>n.538.6.bb_1036
GTAGTTAGCCGTGGCTTTCTGATTAGGTACCGTCAAGACGTGCATAGTTACTTACACATTTGTTCTTCCCTAATAACAGAGTTTTACGATCCGAAGACCTTCATCACTCACGCGGCGTTGCTCCGTCAGGCTTTCGCCCATTGCGGAAGATTCCCTACTGCTGCCTCCCGTAGGAGTCTGGACCGTGTCTCAGTTCCAGTGTGGCCGATCACCCTNTCAGGGTCGGCTACGCATCGTCGC
>n.538.6.bb_1037
GTAGTTAGCCGTGGCTTTCTGATTAGGTACCGTCAAGACGTGCATAGTTACTTACACATTTGTTCTTCCCTAATAACAGAGTTTTACGATCCGAAGACCTTCATCACTCACGCGGCGTTGCTCCGTCAGGCTTTCGCCCATTGCGGAAGATTCCCTACTGCTGCCTCCCGTAGGAGTCTGGACCGTGTCTCAGTTCCAGTGTGGCCGATCACCCTCTCAGGGTCGGCTACGCATCGTTGCCTTGGTAAGCCGTTACCTTACCAACTAGCTAATGCGGCGCGGATCCATCTATAAGTGACAGCAAAACCGTCTTTCACTATTGAACCATGCGGTTCAATA
>n.538.6.bb_1038
GTAGTTAGCCGTGGCTTTCTGATTAGGTACCGTCAAGACGTGCATAGTTACTTACACATTTGTTCTTCCCTAATAACAGAGTTTTACGATCCGAAGACCTTCATCACTCACGCGGCGTTGCTCCGTCAGGCTTTCGCCCATTGCGGAAGATTCCCTACTGCTGCCTCCCGTAGGAGTCTGGACCGTGTCTCAGTTCCAGTGTGGCCGATCACCCTCTCAGGGTCGGCTACGCATCGTTGCCTTGGTAAGCCGTTATCTTACCAACTAGCTAATGCGGCGCGGATCCATCTATAAGTGACAGCAAAACCGTCTTTCACTATTGAACCATGCGGTTCAATATATTATCCGGTAT
>n.538.6.bb_1039
GTAGTTAGCCGTGGCTTTCTGATTAGGTACCGTCAAGACGTGCATAGTTACTTACACATTTGTTCTTCCCTAATAGCAGAGTTTTACGATCCGAAGACCTTCATCACTCACGCGGCGTTGCTCCGTCAGGCTTTCGCCCATTGCGGAAGATTCCCTACTGCTGCCTCCCGTAGGAGTCTGGACCGTGTCTCAGTTCCAGTGTGGCCGATCACCCTCTCAGGTCGGCTACGCATCGTTGCCTTGGTAAGCCGTTACCTTACCAACTAGCTAATG
>n.538.6.bb_1040
GGAGTTAGCCGGTGCTTCTTCTGCGGGTAACGTCAATCGACAAGGTTATTAACCTTATCGCCTTCCTCCCCGCTGAAAGTACTTTACAACCCGAAGGCCTTCTTCATACACGCGGCATGGCTGCATCAGGCTTGCGCCCATTGTGCAATATTCCCCACTGCTGCCTCCCGTAGGAGTCTGGACCGTGTCTCAGTTCCAGTGTGGCTGGTCATCCTCTCAGACCAGCTAGGGATCGTCGCCTAGGTGAGCCGTTACCCACCTACTAGCTAATCCCATCTGGGCACATCTGATG
>n.538.6.bb_1041
GTAGTTAGCCGGTGCTTCTTCTGCAGGTACCGTCACCACAAGCTTCGCCCCTGCTGAAAGCGGTTTACAACCCGAAGGCCGTCATCCCGCACGCGGCGTTGCTGCATCAGGCTTCCGCCCATTGTGCAATATTCCCCACTGCTGCCTCCCGTAGGAGTCTGGGCCGTATCTCAGTCCCAATGTGGCCGGTCGCCCTCTCAGGCCGGCTACCCGGTCAAAGCCTTGGTAACGCCACTACCCCACCAACAAGCTGATAAGCCGCGAGTCCATCCAAAACCGCCG
>n.538.6.bb_1042
GTAGTTAGCCGTGGCCTTCTGATTAGGTACCGTCAAGACGTGCATAGTTACTTACACATTTATTCTTCCCTAATAACAGAGTTTTACGATCCGAAGACCTTCATCACTCACGCGGCGTTGCTCCGTCAGGCTTTCGCCCATTGCGGAAGATTCCCTACTGCTGCCTCCCGTAGGAGTCTGGACCGTGTCTCAGTTCCAGTGTGGCCGATCACCCTCTCAGGGTCGGCTACGCATCGTTGCCTTGGTAAGCCGTTACCTTACCAACTAACTAATCAGACGCGGGTCCATCCTGTACTGGCTCACCTTTTGATATTCAAGAGATGCCTCTCAAATATATTATCCCGTATTAGCATACCTTTCGGTATGTATCCGTGTGTA
>n.538.6.bb_1043
GGAGTTAGCCGGTGCTTCTTCTGCGGGTAACGTCGAATCGACAGGGTTATTAACCCTGTCGCCTTCCTCCCCGCTTGAAAGTTACTTTACAACCCGAAGGCCTTCTTCATACACGCGGCATGGCTGCATCAGGCTTGCGCCCATTCGTGCAATATTCCCCACTGCTGCCTCCCGTAGGAGTCTGGACCGTGTCTCAGTTCCAGTGTGGCTGGTCACTCC
>n.538.6.bb_1044
GTAGTTAGCCGGTGCTTCTTCTGCAGGTACCGTCACCACAAGCTTCGCCCCTGCTGAAAGCGGTTTACAACCCGAAGGCCGTCATCCCGCACGCGGCGTTGCTGCATCAGGCTTCCGCCCATTGTGCAATATTCCCCACTGCTGCCTCCCGTAGGAGTCTGGGCCGTATCTCAGTCCCAATGTGGCCGGTCGCCCTCTCAGGCCGGCTACCCGTCAAAGCCTTGGTAAGCCACTACCCACCAACAAGCTGATAAGCCGCGAGTCCATCCAAAACCGCCGAAGCTTTCCAACCCCCACCGTGCAGCAAGGATTCCTATCCGGTATTAGCCCCAGTTTCC
>n.538.6.bb_1045
GAAGTTAGCCGGTGCTTCTTCTGCAGGTACCGTCACCACAAGCTTCGCCCCTGCTGAAAGCGGTTTACAACCCGAAGGCCGTCATCCCGCACGCGGCGTTGCTGCATCAGGCTTCCGCCCATTGTGCAATATTCCCCACTGCTGCCTCCCGTAGGAGTCTGGGCCGTATCTCAGTCCCAATGTGGCCGGTCGCCCTCTCAGGCCGGCTACCCGTCAAAGCCTTGGTAAGCCACTACCCCACCAACAAGCTGATAAGCCGCGAGTCC
>n.538.6.bb_1046
GTAGTTAGCCGTGGCTTTCTGATTAGGTACCGTCAAGACGTGCATAGTTACTTACACATTTATTCTTCCCTAATAACAGAGTTTTACGATCCGAAGACCTTCATCACTCACGCGGCGTTGCTCCGTCAGGCTTTCGCCCATTGCGGAAGATTCCCTACTGCTGCCTCCCGTAGGAGTCTGGACCGTGTCTCAGTTCCAGTGTGGCCGATCACCCTCTCAGGGTCGGCTACGCATCGTTGCCTTGGTAAGCCGTTACCTTACCAACTAGCTAATGCGGCGCGGATCCATCTATAAGTGA
>n.538.6.bb_1047
GTAGTTAGCCGTGGCTTTCTGATTAGGTACCGTCAAGACGTGCATAGTTACTTACACATTTGTTCTTCCCTAATAACAGAGTTTTACGATCCGAAGACCTTCATCACTCACGCGGCGTTGCTCCGTCAGGCTTTCGCCCATCGCGGAAGATTCCCTACTGCTGCCTCCCGTAGGAGTCTGGACCGTGTCTCAGTTCCAGTGTGGCCGATCACCCTNTCAGGGTCGGCTACGCATCGTCGCCTTGGTAAGCCGTTACCTTACCAACTAGCTAATGCGGCGCGGATCCATCTATAAGTGACAGCAAAACCGTCTTTCACTATTGAACCATGCGGTTC
>n.538.6.bb_1048
GTAGTTAGCCGTGGCTTTCTGATTAGGTACCGTCAAGACGTGCATAGTTACTTACACATTTGTTCTTCCCTAATAACAGAGTTTTACGATCCGAAGACCTTCATCACTCACGCGGCGTTGCTCCGTCAGGCTTTCGCCCATTGCGGAAGATTCCCTACTGCTGCCTCCCGTAGGAGTCTGGACCGTGTCTCAGTTCCAGTGTGGCCGATCACCCTCTCAGGGTCGGCTACGCATCGTTGCCTTGGTAAGCCGTTACCTTACCAACTAGCTAATGCGGCGCGGATCCATCTATAAGTGACAGCAAAACCGTCTTTCACTATTGAACCATGCGGTTCAATATATTATCCGGTATTAGCTC
>n.538.6.bb_1049
GTAGTTAGCCGGTGCTTCTTCTGCAGGTACCGTNACCACCAAGACTTCGTCCCCTCGCTGAAAGACGGTTTACGTAACCCGAACGGCCGTCCATCCCGCCACGCGGCGTTGCTGCATCAGGCTTCCGCCCATTGTGCAATATTCCCCACTGCTGCCTCCCGTAGGAGTCTGGGCCGTATCTCAGTCCCAATGTGGCCGGT
>n.538.6.bb_1050
GTAGTTAGCCGTGGCTTTCTGATTAGGTACCGTCAAGACGTGCATAGTTACTTACACATTTGTTCTTCCCTAATAACAGAGTTTTACGATCCGAAGACCTTCATCACTCACGCGGCGTTGCTCCGTCAGGCTTTCGCCCATTGCGGAAGATTCCCTACTGCTGCCTCCCGTAGGAGTCTGGACCGTGTCTCAGTTCCAGTGTGGCCGATCACCCTCTCAGGGTCGGCTACGCATCGTTGCCTTGGTAAGCCGTTACCTTACCAACTAGCTAATGCGGCGCGGATCCATCTATAAGTGACAGCAAAACCGTCTTTCACTATTGAACCATGCGGTTCAATATATTATCCGGTATTAGCTCCGGTTTCCCGAAGTTATCCCAGTCTTATAGGTAGGTTAT
>n.538.6.bb_1051
GTAGTTAGCCGGTGCTTCTTCTGCAGGTACCGTCACCACAAGCTTCGCCCCTGCTGAAAGCGGTTTACAACCCGAAGGCCGTCATCCCGCACGCGGCGTTGCTGCATCAGGCTTCCGCCCATTGTGCAATATTCCCCACTGCTGCCTCCCGTAGGAGTCTGGGCCGTATCTCAGTCCCAATGTGGCCGGTCGCCCTCTCAGGGCCGGCTACCCGTCAAAGCCTTGGTAAGCCACTACCCCACCAACAAGCTGATAAGCCGCGAGTCCAT
>n.538.6.bb_1052
GGAGTTAGCCGGTGCTTCTTCTGCGGGTAACGTCAATCGACAAGGTTATTAACCTTATCGCCTTCCTCCCCGCTGAAAGTACTTTACAACCCGAAGGCCTTCTTCATACACGCGGCATGGCTGCATCAGGCTTGCGCCCATTGTGCAATATTCCCCACTGCTGCCTCCCGTAGGAGTCTGGACCGTGTCTCAGTTCCAGTGTGGCTGGTCATCCTCTCAGACCAGCTAGGGATCGTCGCCTAGGTGAGCCGTTACCCACCTACTAGCTAATCCCAT
>n.538.6.bb_1053
GTAGTTAGCCGGTGCTTCTTCTGCAGGTACCGTCACCACAAGCTTCGCCCCTGCTGAAAGCGGTTTACAACCCGAAGGCCGTCATCCCGCACGCGGCGTTGCTGCATCAGGCTTCCGCCCATTGTGCAATATTCCCCACTGCTGCCTCCCGTAGGAGTCTGGGCCGTATCTCAGTCCCAATGTGGCCGGTCGCCCTCTCGGGCCGGCTACCCGGTCAAAGCCTTGGGTAANCCACTACCCACAACAAGCTGATAAGCCGCGAGTCCATCCAAAACCGCCGAAGCTTTCCAACCCCCACCATGCAGCAAGGATTCCTATCCGGTATTAGCCCCAGTTTCCTGAAGTTATCCCGAAGTCAAGGGCAGGTTACTCACGT
>n.538.6.bb_1054
GTAGTTAGCCGTGGCTTTCTGATTAGGTACCGTCAAGACGTGCATAGTTACTTACACATTTGTTCTTCCCTAATAACAGAGTTTTACGATCCGAAGACCTTCATCACTCACGCGGCGTTGCTCCGTCAGGCTTTCGCCCATTGCGGAAGATTCCCTACTGCTGCCTCCCGTAGGAGTCTGGACCGTGTCTCAGTTCCAGTGTGGCCGATCACCCTCTCAGGGTCGGCTACGCATCGTTGCCTTGGTAAGCCGTTACCTTACCAACTAGCTAATGCGGCGCGGATCCATCTATAAGTGACAGCAAAACCGTCTTTCACTATTGAACCATGCGGTTCAATATATTATCCGGTATTAGCTCCCGGT
>n.538.6.bb_1055
GTAGTTAGCCGGTGCTTCTTCTGCAGGTACCGTCACCACAAGCTTCGCCCCTGCTGAAAGCGGTTTACAACCCGAAGGCCGTCATCCCGCACGCGGCGTTGCTGCATCAGGCTTCCGCCCATTGTGCAATATTCCCCACTGCTGCCTCCCGTAGGAGTCTGGGCCTATCTCAGTCCCAATGTGGCCGGTCGCCCTCTCAGGCCCGGCTACCCGGTNAAAGCCTTGGTAAGCCACTACCCCACCAACAAGCTGATAAGCCGCGAGTCCATCCAAAACCGCCGAAGCTTTCCAACCCCCACCATGCAGCAAGGATTCCTATCCGGTATTAGC
>n.538.6.bb_1056
GGAGTTAGCCGGTGCTTCTTCTGCGGGTAACGTCAATCGACACGGTTATTAACCGCATCGCCCTCCTCCCCGCTGAAAGTACTTTACAACCCGAAGGCCTTCTTCATACACGCGGCATGGCTGCATCAGGCTTGCGCCCATTGTGCAATATTCCCCACTGCTGCCTCCCGTAGGAGTCTGGACCGTGTCTTAGTTCCAGTGTGGCTGGTCATCCTCTCA
>n.538.6.bb_1057
GTAGTTAGCCGTGGCTTTCTGATTAGGTACCGTCAAGACGTGCATAGTTACTTACACATTTATTCTTCCCTAATAACAGAGTTTTACGATCCGAAGACCTTCATCACTCACGCGGCGTTGCTCCGTCAGGCTTTCGCCCATTGCGGAAGATTCCCTACTGCTGCCTCCCGTAGGAGTCTGGACCGTGTCTCAGTTCCAGTGTGGCCGATCACCCTCTCAGGGTCGGCTACGCATCGTTGCCTTGGTAAGCCGTTACCTTACCAACTAGCTAATGCGGCGCGGATCCATCTATAAGTGACAGCAAAACCGTCTTTCACTATTGAACCATGCGGTTCAATATATTATCCGGTATTAGCT
>n.538.6.bb_1058
GTAGTTAGCCGTGGCTTTCTGATTAGGTACCGTCAAGACGTGCATAGTTACTTACACATTTGTTCTTCCCTAATAACAGAGTTTTACGATCCGAAGACCTTCATCACTCACGCGGCGTTGCTCCGTCAGGCTTTCGCCCATTGCGGAAGATTCCCTACTGCTGCCTCCCGTAGGAGTCTGGACCGTGTCTCAGTTCCAGTGTGGCCGATCACCCTCTCAGGGTCGGCTACGCATCGTCGCCTTGGTAAGCCGTTACCTTACCAACTAGCTAATGCGGCGCGGATCCATCTATAAGTGACAGCAAAACCGTCTTTCACTATTGAACCATGC
>n.538.6.bb_1059
GTAGTTAGCCGGTGCTTCTTCTGCAGGTACCGTCACCACAAGCTTCGCCCCTGCTGAAAGCGGTTTACAACCCGAAGGCCGTCATCCCGCACGCGGCGTTGCTGCATCAGGCTTCCGCCCATTGTGCAATATTCCCCACTGCTGCCTCCCGTAGGAGTCTGGGCCGTATCTCAGTCCCAATGTGGCCGGTCGCCCTCTCAGGCCGGCTACCCGGTCAAAGCCTTGGTAAGCCACTACCCCACCAACAAGCTGATAAGCCGCGAGTCCATCCAA
>n.538.6.bb_1060
GTAGTTAGCCGTGGCTTTCTGATTAGGTACCGTCAAGACGTGCATAGTTACTTACACATTTGTTCTTCCCTAATAACAGAGTTTTACGATCCGAAGACCTTCATCACTCACGCGGCGTTGCTCCGTCAGGCTTTCGCCCATTGCGGAAGATTCCCTACTGCTGCCTCCCGTAGGAGTCTGGACCGTGTCTCAGTTCCAGTGTGGCCGATCACCCTCTCAGGGTCGGCTACGCATCGTTGCCTTGGTAAGCCGTTACCTTACCAACTAGCTAATGCGGCGCGGATCCATCTATAAGTGACAGCAAAACCGTCTTTCACTATTGAACCATGCGGTTCAATATATTATCCGGTATTAG
>n.538.6.bb_1061
GTAGTTAGCCGTGGCTTTCTGATTAGGTACCGTCAAGACGTGCATAGTTACTTACACATTTGTTCTTCCCTAATAACAGAGTTTTACGATCCGAAGACCTTCATCACTCACGCGGCGTTGCTCCGTCAGCTTTCGCCCATTGCGGAAGATTCCCTACTGCTGCCTCCCGTAGGAGTCTGGACCGTGTCTCAGTTCCAGTGTGGCCGATCACCCTCTCAGGGTCGGCTACGCATCGTTGCCTTGGTAAGCCGTTACCTTACCAACTAGCTAATGCGGCGCGGATCCATCTATAAGTGAC
>n.538.6.bb_1062
GTAGTTAGCCGTGGCTTTCTGATTAGGTACCGTCAAGACGTGCATAGTTACTTACACATTTGTTCTTCCCTAATAACAGAGTTTTACGATCCGAAGACCTTCATCACTCACGCGGCGTTGCTCCGTCAGGCTTTCGCCCATTGCGGAAGATTCCCTACTGCTGCCTCCCGTAGGAGTCTGGACCGTGTCTCAGTTCCAGTGTGGCCGATCACCCTCTCAGGGTCGGCTACGCATCGTTGCCTTGGT
>n.538.6.bb_1063
GTAGTTAGCCGTGGCTTTCTGATTAGGTACCGTCAAGACGTGCATAGTTACTTACACATTTGTTCTTCCCTAATAACAGAGTTTTACGATCCGAAGACCTTCATCACTCACGCGGCGTTGCTCCGTCAGGCTTTCGCCCATTGCGGAAGATTCCCTACTGCTGCCTCCCGTAGGAGTCTGGACCGTGTCTCAGTTCCAGTGTGGCCGATCACCCTCTCAGGGTCGGCTACGCATCGTTGCCTTGGTAAGCCGTTACCTTACCAACTAGCTAATGCGGCGCGGATCCATCTATAAGTG
>n.538.6.bb_1064
GTAGTTAGCCGTGGCTTTCTGATTAGGTACCGTCAAGACGTGCATAGTTACTTACACATTTTGTTCTTCCCTAATAACAGAGTTTTACGATCCGAAGACCTTCATCACTCACGCGGCGTTGCTCCGTCAGGCTTTCGCCCATTGCGGAAGATTCCCTACTGCTGCCTCCCGTAGGAGTCTGGACCGTGTCTCAGTTCCAGTGTGGCCGATCACCCTCTCAGGGTCGGCTACGCATCGTTGCCTTGGTAAGCCGTTACCTTACCAACTAGCTAATGCGGCGCGGATCCATCTATAAGTGACAGACAAAACCG
>n.538.6.bb_1065
GTAGTTAGCCGTGGCTTTCTGATTAGGTACCGTCAAGACGTGCATAGTTACTTACACATTTGTTCTTCCCTAATAACAGAGTTTTACGATCCGAAGACCTTCATCACTCACGCGGCGTTGCTCCGTCAGGCTTTCGCCCATTGCGGAAGATTCCCTACTGCTGCCTCCCGTAGGAGTCTGGACCGTGTCTCAGTTCCAGTGTGGCCGATCACCCTCTCAGGGTCGGCTACGCATCGTTGCCTTGGTAAGCCGTTACCTTACCAACTAGCTAATGCGGCGCGGATCCATCTATAAGTGACAGCAAAACCGTCTTTCACTATTGAACCATGCGGTTCAATATATTATCCGGTATTAGCTCCGGT
>n.538.6.bb_1066
GTAGTTAGCCGTGGCTTTCTGATTAGGTACCGTCAAGACGTGCATAGTTACTTACACATTTGTTCTTCCCTAATAACAGAGTTTTACGATCCGAAGACCTTCATCACTCACGCGGCGTTGCTCCGTCAGGCTTTCGCCCATTGCGGAAGATTCCCTACTGCTGCCTCCCGTAGGAGTCTGGACCGTGTCTCAGTTCCAGTGTGGCCGATCACCCTCTCAGGGTCGGCTACGCATCGTCGCCTTGGTAAGCCGTTACCTTACCAACTAGCTAATGCGGCGCGGATCCATC
>n.538.6.bb_1067
GGAGTTAGCCGGTGCTTCTTCTGCGGGTAACGTCAATCGACACGGTTATTAACCGCATCGCCTTCCTCCCCGCTGAAAGTACTTTACAACCCGAAGGCCTTCTTCATACACGCGGCATGGCTGCATCAGGCTTGCGCCCATTGTGCAATATTCCCCACTGCTGCCTCCCGTAGGAGTCTGGACCGTGTCTCAGTTCCAGTGTGGCTGGGTCATCCTCTCAGACCAGCTAGGGATCGTCGCCTAGGTGAGCCGTTACCCCACCTACTAGCTAATCCCATCTGGGTTCATCTGATG
>n.538.6.bb_1068
GTAGTTAGCCGGTGCTTCTTCTGCAGGTACCGTCACCACAAGCTTCACCCCTGCTGAAAGCGGTTTACAACCCGAAGGCCGTCATCCCGCACGCGGCGTTGCTGCATCAGGCTTCCGCCCATTGTGCAATATTCCCCACTGCTGCCTCCCGTAGGAGTCTGGGCCGTATCTCAGTCCCAATGTGGCCGGTCGCCCTCTCAGGCCGGCTACCCCGGTNAAAGCCTTGGTAAAGCCACTACCCACCAACAAGCTGATAAGCCGCGAGTCCATCCAAAACCGCCGAAGCTTTTCCCAAA
>n.538.6.bb_1069
GTAGTTAGCCGTGGCTTTCTGATTAGGTACCGTCAAGACGTGCATAGTTACTTACACATTTGTTCTTCCCTAATAACAGAGTTTTACGATCCGAAGACCTTCATCACTCACGCGGCGTTGCTCCGTCAGGCTTTCGCCCATTGCGGAAGATTCCCTACTGCTGCCTCCCGTAGGAGTCTGGACCGTGTCTCAGTTCCAGTGTGGCCGATCACCCTCTCAGGGTCGGCTACGCATCGTCGCCTTGGTAAGCCGTTACCTTACCAACTAGCTAATGCGGCGCGGATCCATCTATAAGTGACAGCAAAACCGTCTTTCACTATTGAACCATGCGGTTCAATATATTATCCGGTAT
>n.538.6.bb_1070
GTAGTTAGCCGGTGCTTCTTCTGCAGGTACCGTCACCACAAGCTTCGCCCCTGCTGAAAGCGGTTTACAACCCGAAGGCCGTCATCCCGCACGCGGCGTTGCTGCATCAGGCTTCCGCCCATTGTGCAATATTCCCCACTGCTGCCTCCCGTAGGAGTCTGGGCCGTATCTCAGTCCCAATGTGGCCGGTCGCCCTCTCAGGCCGGCTACCCGTCAAAGCCTTGGTAAGCCACTACCCACCAGCAAGCTGATAAGCCGCGAGTCCAT
>n.538.6.bb_1071
GTAGTTAGCCGTGGCTTTCTGATTAGGTACCGTCAAGACGTGCATAGTTACTTACACATTTGTTCTTCCCTAATAACAGAGTTTTACGATCCGAAGACCTTCATCACTCACGCGGCGTTGCTCCGTCAGGCTTTCGCCCATTGCGGAAGATTCCCTACTGCTGCCTCCCGTAGGAGTCTGGACCGTGTCTCAGTTCCAGTGTGGCCGATCACCCTCTCAGGGTCGGCTACGCATCGTTGCCTTGGTAAGCCGTTACCTTACCACTAGCTAATGCGGCGCGGATCCATCTATAAGTGACAGCAAAACCGTCTTTCACTATTGGACCATGCGGTTCAATAT
>n.538.6.bb_1072
GTAGTTAGCCGGTGCTTCTTCTGCAGGTACCGTCACCACAAGCTTCGCCCCTGCTGAAAGCGGTTTACAACCCGAAGGCCGTCATCCCGCACGCGGCGTTGCTGCATCAGGCTTCCGCCCATTGTGCAATATTCCCCACTGCTGCCTCCCGTAGGAGTCTGGGCCGTATCTCAGTCCCAATGTGGCCGGTCGCCCTCTCAGGCCGGCTACCCGTCAAAGCCTTGGTAAGCCACTACCCACCAAC
>n.538.6.bb_1073
GTAGTTAGCCGTGGCTTTCTGATTAGGTACCGTCAAGACGTGCATAGTTACTTACACATTTATTCTTCCCTAATAACAGAGTTTTACGATCCGAAGACCTTCATCACTCACGCGGCGTTGCTCCGTCAGGCTTTCGCCCATTGCGGAAGATTCCCTACTGCTGCCTCCCGTAGGAGTCTGGACCGTGTCTCAGTTCCAGTGTGGCCGATCACCCTCTCAGGGTCGGCTACGCATCGTTGCCTTGGTAAGCCGTTACCTTACCAACTAGCTAATGCGGCGCGGATCCATCTATAAGTGACAGCAAAAACCGTCTCTCACTATTGAACCATGCGGTTCAATATATTATCCGGTATTAGCT
>n.538.6.bb_1074
GGAGTTAGCCGGTGCTTCTTCTGCGGGTAACGTCAATTGCTGAGGTTATTAACCTCAACACCTTCCTCCCCGCTGAAAGTACTTTACAACCCGAAGGCCTTCTTCATACACGCGGCATGGCTGCATCAGGCTTGCGCCCATTGTGCAATATTCCCCACTGCTGCCTCCCGTAGGAGTCTGGACCGTGTCTCAGTTCCAGTGTGGCTGGGTC
>n.538.6.bb_1075
GTAGTTAGCCGTGGCTTTCTGATTAGGTACCGTCAAGACGTGCATAGTTACTTACACATTTATTCTTCCCTAATAACAGAGTTTTACGATCCGAAGACCTTCATCACTCACGCGGCGTTGCTCCGTCAGGCTTTCGCCCATTGCGGAAGATTCCCTACTGCTGCCTCCCGTAGGAGTCTGGACCGTGTCTCAGTTCCAGTGTGGCCGATCACCCTCTCAGGGTCGGCTACGCATCGTTGCCTTGGTAAGCCGTTACCTTACCAACTAGCTAATGCGGCGCGGATCCATCTATAAGTGACAGCAAAACCGTCTTTCACTATTGAACCATGCGGTTCAATATATTATCCGGTATT
>n.538.6.bb_1076
GTAGTTAGCCGGTGCTTCTTCTGCAGGTACCGTCACCACAAGCTTCGCCCCTGCTGAAAGCGGTTTACAACCCGAAGGCCGTCATCCCGCACGCGGCGTTGCTGCATCAGGCTTCCGCCCATTGTGCAATATTCCCCACTGCTGCCTCCCGTAGGAGTCTGGGCCGTATCTCAGTCCCAATGTGGCCGGTCGCCCTCTCAGGCCGGCTACCCGTCAAAGCCTTGGTAACGCCACTACCCCACCAACAAGCTGATAAGCCGCGAGTCCATCCAAAACCG
>n.538.6.bb_1077
GTAGTTAGCCGTGGCTTTCCGATTAGGTACCGTCAAGACGTAGCATAGTTACTTACACGATTTGTTCTTCCCTAATAACAGAGTTTTACGATCCGAAGACCTTCATCACTCACGCGGCGTTGCTCCGTCAGGCTTTCGCCCATTGCGGAAGATTCCCTACTGCTGCCTCCCGTAGGAGTCTGGACCGTGTCTCAGTTCCAGTGTGGCCGATCACCCTCTCAGGGTCGGCTACGCATCGTTGCCTTGGTAAGCCGTTACCTTACCAACTAGCTAATGCGGCGCGGATCCATCTATAAAGTGACAGCAAAACCGTCTTTCACTATTGAACCATGCGGTTCAATATATT
>n.538.6.bb_1078
GTAGTTAGCCGTGGCTTTCTGATTAGGTACCGTCAAGACGTGCATAGTTACTTACACATTTGTTCTTCCCTAATAACAGAGTTTTACGATCCGAAGACCTTCATCACTCACGCGGCGTTGCTCCGTCAGGCTTTCGCCCATTGCGGAAGATTCCCTACTGCTGCCTCCCGTAGGAGTCTGGACCGTGTCTCAGTTCCAGTGTGGCCGATCACCCTCTCAGGGTCGGCTACGCATCGTTGCCTTGGTAAGCCGTTACCTTACCAACTAGCTAATGCGGCGCGGATCCATCTATAAGTGACAGCAAAACCGT
>n.538.6.bb_1079
GTAGTTAGCCGTGGCTTTCTGATTAGGTACCGTCAAGACGTGCATAGTTACTTACACATTTGTTCTTCCCTAATAACAGAGTTTTACGATCCGAAGACCTTCATCACTCACGCGGCGTTGCTCCGTCAGGCTTTCGCCCATTGCGGAAGATTCCCTACTGCTGCCTCCCGTAGGAGTCTGGACCGTGTCTCAGTTCCAGTGTGGCCGATCACCCTCTCAGGGTCGGCTACGCATCGTTGCCTTGGTAAGCCGTTACCTTACCAACTAGCTAATGCGGCGCGGATCCATCTATAAGTGACAGCAAAACCGTCTTTCACTATTGAACCATGCGGTTCAATATATTATCCGGTATTAGCTCCGGT
>n.538.6.bb_1080
TAGTTAGCCGTGGCTTTCTGATTAGGTACCGTCAAGACGTGCATAGTTACTTACACATTTGTTCTTCCCTAATAACAGAGTTTTACGATCCGAAGACCTTCATCACTCACGCGGCGTTGCTCCGTCAGGCTTTCGCCCATTGCGGAAGATTCCCTACTGCTGCCTCCCGTAGGAGTCTGGACCGTATCTCAGTCCCAATGTGGCCGGTCGCCCTCTCAGGGCCGGGCTACCCGTCAAAGCCTTGGTAAGCCACTACCCCACAACAAGCTGATAAGCCGCGAGTCCATCCAAAACCGC
>n.538.6.bb_1081
GTAGTTAGCCGGTGCTTCTTCTGCAGGTACCGTCACCACAAGCTTCGCCCCTGCTGAAAGCGGTTTACAACCCGAAGGCCGTCATCCCGCACGCGGCGTTGCTGCATCAGGCTTCCGCCCATTGTGCAATATTCCCCACTGCTGCCTCCCGTAGGAGTCTGGGCCGTATCTCAGTCCCAATGTGGCCGGTCGCCCTCTCAGGCCGGCTACCCGGTCAAAGCCTTGGTAAGCCGTTACCTTACCAACTAGCTAATGCGGCGCGGATCCATCTATAAGTGACAGCAAAACCGTCTTTCACTA
>n.538.6.bb_1082
GTAGTTAGCCGTGGCTTTCTGATTAGGTACCGTCAAGACGTGCATAGTTACTTACACATTTGTTCTTCCCTAATAACAGAGTTTTACGATCCGAAGACCTTCATCACTCACGCGGCGTTGCTCCGTCAGGCTTTCGCCCATTGCGGAAGATTCCCTACTGCTGCCTCCCGTAGGAGTCTGGACCGTGTCTCAGTTCCAGTGTGGCCGATCACCCTCTCAGGGTCGGCTACGCATCGTTGCCTTGGTAAGCCGTTACCTTACCAACTAGCTAATGCGGCGCGGATCCATCTATAAGTGAC
>n.538.6.bb_1083
GTAGTTAGCCGTGGCTTTCTGATTAGGTACCGCCAAGACGTGCATAGTTACTTACACATTTGTTCTTCCCTAATAACAGAGTTTTACGATCCGAAGACCTTCATCACTCACGCGGCGTTGCTCCGTCAGGCTTTCGCCCATTGCGGAAGATTCCCTACTGCTGCCTCCCGTAGGAGTCTGGACCGTGTCTCAGTTCCAGTGTGGCCGATCACCCTCTCAGGGTCGGCTACGCATCGTTGCCTTGGTAAGCCGTTACCTTACCAACTAGCTAATGCGGCGCGGATCCATCTATAAGT
>n.538.6.bb_1084
GTAGTTAGCCGGTGCTTCTTCTGCAGGTACCGTCACCACAAGCTTCGCCCCTGCTGAAAGCGGTTTACAACCCGAAGGCCGTCATCCCGCACGCGGCGTTGCTGCATCAGGCTTCCGCCCATTGTGCAATATTCCCCACTGCTGCCTCCCGTAGGAGTCTGGGCCGTATCTCAGTCCCAATGTGGCCGGTCGCCCTCTCAGGCCGGCTACCCGGTCTAAGCCTTGGTAAGCCACTACCCCACCAACAAGCTGATAAGCCGCGAGTCCATCCAAAACCGCCGA
>n.538.6.bb_1085
GTAGTTAGCCGGTGCTTCTTCTGCAGGTACCGTCACCACAAGCTTCGCCCCTGCTGAAAGCGGTTTACAACCCGAAGGCCGTCATCCCGCACGCGGCGTTGCTGCATCAGGCTTCCGCCCATTGTGCAATATTCCCCACTGCTGCCTCCCGTAGGAGTCTGGGCCGTATCTCAGTCCCAATGTGGCCGGTCGCCCTCTCAGGCCGGCTACCCGTCAAAGCCTTGGTAAGCCACTACCCACCAACAAGCTGATAAGCCGCGAGTCCATCCAAAACCGC
>n.538.6.bb_1086
GTAGTTAGCCGTGGCTTTCTGATTAGGTACCGTCAAGACGTGCATAGTTACTTACACATTTGTTCTTCCCTAATAACAGAGTTTTACGATCCGAAGACCTTCATCACTCACGCGGCGTTGCTCCGTCAGGCTTTCGCCCATTGCGGAAGATTCCCTACTGCTGCCTCCCGTAGGAGTCTGGACCGTGTCTCAGTTCCAGTGTGGCCGATCACCCTCTCAGGGTCGGCTACGCATCGTTGCCTTGGTAAGCCGTTACCTTACCAACTAGCTAATGCGGCGCGGATCCATCTATAAGTGACAGCAAAACCGTCTTTCACTATTGAACCATGCGGTTCAATATATTATCCGGTATTAGCTCCGGTTTCCCGAAGTTATCCCAGTCTTATAGGTAGGTTATCC
>n.538.6.bb_1087
GTAGTTAGCCGTGGCTTTCTGATTAGGTACCGTCAAGACGTGCATAGTTACTTACACATTTGTTCTTCCCTAATAACAGAGTTTTACGATCCGAAGACCTTCATCACTCACGCGGCGTTGCTCCGTCAGGCTTTCGCCCATTGCGGAAGATTCCCTACTGCTGCCTCCCGTAGGAGTCTGGACCGTGTCTCAGTTCCAGTGTGGCCGATCACCCTCTCAGGGTCGGCTACGCATCGTTGCCTTGGTAAGCCGTTACCTTACCAACTAGCTAATGCGGCGCGGATCCATCTATAAGTGACAGCAAAACCGTCTTTCACTATTGAACCATGCGGTTCAATATATTATCCGGTATT
>n.538.6.bb_1088
GTAGTTAGCCGGTGCTTCTTCTGCAGGTACCGTCACCACAAGCTTCGCCCCTGCTGAAAGCGGTTTAACAACCCGAAGGCCGTCATCCCGCACGCGGCGTTGCTGCATCAGGCTTCCGCCCATTGTGCAATATTCCCCACTGCTGCCTCCCGTAGGAGTCTGGGCCGTATCTCAGTCCCAATGTGGCCGGTCGCCCTCTCAGGCCGGCTACCCGTCAAAGCCTTGGTAAGCCACTACCCACCAACAAGCTGATAAGCCGCGAGTCCATCCAAAACCGCC
>n.538.6.bb_1089
GTAGTTAGCCGGTGCTTCTTCTGCAGGTACCGTCACCACAAGCTTCGCCCCTGCTGAAAGCGGTTTACAACCCGAAGGCCGTCATCCCGCACGCGGCGTTGCTGCATCAGGCTTCCGCCCATTGTGCAATATTCCCCACTGCTGCCTCCCGTAGGAGTCTGGGCCGTATCTCAGTCCCAATGTGGCCGGTCGCCCTCTCAGGCCGGCTACCCGGTCAAAGGCCTTGGTAACGCCACTACCCACCAACAAGCTGATAAGCCGCGAGTCCATCC
>n.538.6.bb_1090
GTAGTTAGCCGTGGCTTTCTGATTAGGTACCGTCAAGACGTGCATAGTTACTTACACATTTGTTCTTCCCTAATAACAGAGTTTTACGATCCGAAGACCTTCATCACTCACGCGGCGTTGCTCCGTCAGGCTTTCGCCCATTGCGGAAGATTCCCTACTGCTGCCTCCCGTAGGAGTCTGGACCCGTGTCTCAGTTCCAGTGTGGCCGATCACCC
>n.538.6.bb_1091
GGAGTTAGCCGGTGCTTCTTCTGCGGGTAACGTCAATCGACAAGGTTATTAACCTTATCGCCTTCTTCCCCGCTGAAAGTACTTTACAACCCGAAGGCCTTCTTCATACACGCGGCATGGCTGCATCAGGCTTGCGCCCATTGTGCAATATTCCCCACTGCTGCCTCCGTAGGAGTCTGGACCGTGTCTCAGTTCCAGTGTGGCTGGTCATCCTCTCAGACCAGCTAGGGATCGTCGCCTAGGTGAGCCGTT
>n.538.6.bb_1092
GTAGTTAGCCGTGGCTTTCTGATTAGGTACCGTCAAGACGTGCATAGTTACTTACACATTTGTTCTTCCCTAATAACAGAGTTTTACGATCCGAAGACCTTCATCACTCACGCGGCGTTGCTCCGTCAGGCTTTCGCCCATTGCGGAAGATTCCCTACTGCTGCCTCCCGTAGGAGTCTGGACCGTGTCTCAGTTCCAGTGTGGCCGATCACCCTCTCGAGGGTCGGCTACGCATCGTTGCCTTGGTAAGCCGTTACCTTACCAACTAGCTAATGCGGCGCGGATCCATCT
>n.538.6.bb_1093
GTAGTTAGCCGTGGCTTTCTGATTAGGTACCGTCAAGACGTGCATAGTTACTTACACATTTGTTCTTCCCTAATAACAGAGTTTTACGATCCGAAGACCTTCATCACTCACGCGGCGTTGCTCCGTCAGGCTTTCGCCCATTGCGGAAGATTCCCTACTGCTGCCTCCCGTAGGAGTCTGGACCGTGTCTCAGTTCCAGTGTGGCCGATCACCCTCTCAGGGTCGGCTACGCATCGTTGCCTTGGTAAGCCGTTACCTTACCAACTAGCTAATGCGGCGCGGATCCATCTATAAGTGACAGCAAAACCGTCTTTCACTATTTGAACCATGCGGTTCAATATATTATCCGGTATTAGCTCCGGTTTCCCGAAGTTATCCCAGTCTTATAGGTAGGTTATCCCACGTGTTACCTCACCCCGTCCCGCCCGCTAACGTCAGAGAAGCAAGCCTCCCTCGTCTGTTCGCTCGACTTGCATGTATTAGGGCACGCC
>n.538.6.bb_1094
GTAGTTAGCCGGTGCTTCTTCTGCAGGTACCGTCACCACAAGCTTCGCCCCTGCTGAAAGCGGTTTACAACCCGAAGGCCGTCATCCCGCACGCGGCGTTGCTGCATCAGGCTTCCGCCCATTGTGCAATATTCCCCACTGCTGCCTCCCGTAGGAGTCTGGGCCGTATCTCAGTCCCAATGTGGCCGGTCGCCCTCTCAGGCCGGCTACCCGTCAAAGCCTTGGTAAGCCACTACCCCACCAACAAGCTGATAAGCCGCGAGTCCATCCAAAACCGCCG
>n.538.6.bb_1095
GTAGTTAGCCGTGGCTTTCTGATTAGGTACCGTCAAGACGTGCATAGTTACTTACACATTTGTTCTTCCCTAATAACAGAGTTTTACGATCCGAAGACCTTCATCACTCACGCGGCGTTGCTCCGTCAGGCTTTCGCCCATTGCGGAAGATTCCCTACTGCTGCCTCCCGTAGGAGTCTGGACCGTGTCTCAGTTCCAGTGTG
>n.538.6.bb_1096
GTAGTTAGCCGGTGCTTCTTCTGCAGGTACCGTCACCACAAGCTTCGCCCCTGCTGAAGGCGGTTTACAACCCGAAGGCCGTCATCCCGCACGCGGCGTTGCTGCATCAGGCTTCCGCCCATTGTGCAATATTCCCCACTGCTGCCTCCCGTAGGAGTCTGGGCCGTATCTCAGTCCCAATGTGGCCGGTCGCCCTCTCAGGCCGGCTACCCGGTCAAAGCCTTGGTAAGCCACTACCCCACCAACAAGCTGATAAGCCGCGAGTCCATCCAAAACCGCCG
>n.538.6.bb_1097
GTAGTTAGCCGTGGCTTTCTGATTAGGTACCGTCAAGACGTGCATAGTTACTTACACATTTGTTCTTCCCTAATAACAGAGTTTTACGATCCGAAGACCTTCATCACTCACGCGGCGTTGCTCCGTCAGGCTTTCGCCCATTGCGGAAGATTCCCTACTGCTGCCTCCCGTAGGAGTCTGGACCGTGTCTCAGTTCCAGTGTGGCCGATCACCCTCTCAGGGTCGGCTACGCATCGTTGCCTTGGTAAGCCGTTACCTTACCAACTAGCTAATGCGGCGCGGATCCATCTATAAGTGACAGCAAAACCGTCTTTCACTATTGAACCATGCGGTTCAATATATTATCCGGTATTAGCTCCGGT
>n.538.6.bb_1098
GTAGTTAGCCGTGGCTTTCTGATTAGGTACCGTCAAGACGTGCATAGTTACTTACACATTTGTTCTTCCCTAATAACAGAGTTTTACGATCCGAAGACCTCCATCACTCACGCGGCGTTGCTCCGTCAGGCTTTCGCCCATTGCGGAAGATTCCCTACTGCTGCCTCCCGTAGGAGTCTGGACCGTGTCTCAGTTCCAGTGTGGCTGGTCATCCTCTCAGACCCAGCTAGGGGATCGTCGCCTAGGTGAGCCGTTACCCACCTACTAGCTAATCCCATCTGGGCACATCTGATG
>n.538.6.bb_1099
GTAGTTAGCCGTGGCTTTCTGATTAGGTACCGTCAAGACGTGCATAGTTACTTACACATTTGTTCTTCCCTAATAACAGAGTTTTACGATCCGGAGACCTTCATCACTCACGCGGCGTTGCTCCGTCAGGCTTTCGCCCATTGCGGAAGATTCCCTACTGCTGCCTCCCGTAGGAGTCTGGACCGTGTCTCAGTTCCAGTGTGGCCGATCACCCTCTCAGGGTCGGCTACGCATCGTTGCCTTGGTAAGCCGTTACCTTACCAACTAGCTAATGCGGCGCGGATCCATCTATAAGTGACAGCAAAACCGTCTTTCACTATTGAACCATGCGGTTCAATATATTATCCGGTATT
>n.538.6.bb_1100
GTAGTTAGCCGTGGCTTTCTGATTAGGTACCGTCAAGACGTGCATAGTTACTTACACATTTGTTCTTCCCTAATAACAGAGTTTTACGATCCGAAGACCTTCATCACTCACGCGGCGTTGCTCCGTCAGGCTTTCGCCCATTGCGGAAGATTCCCTACTGCTGCCTCCCGTAGGAGTCTGGACCGTGTCTCAGTTCCAGTGTGGCCGATCACCCTCTCAGGGTCGGCTACGCATCGTTGCCTTGGTAAGCCGTTACCTTACCAACTAGCTAATGCGGCGCGGATCCATCTATAAGTGACAGCAAAACCGTCTTTCACTATTGAACCATGCGGTTCAATATATTATCCGGTATTAG
>n.538.6.bb_1101
GTAGTTAGCCGTGGCTTTCTGATTAGGTACCGTCAAGACGTGCATAGTTACTTACACATTTTGTTTTTCCCTAACTAACAGAGTTTTACGTATCCGAAGACCTTCATCACTCACGCGGCGTTGCTCCGTCAGGCTTTCGCCCATTGCGGAAGATTCCCTACTGCTCGCCTCCCGTAGGAGTCTGGACCGCGTCTCAGTTCCAG
>n.538.6.bb_1102
GTAGTTAGCCGGTGCTTCTTCTGCAGGTACCGTCACCACAAGCTTCGCCCCTGCTGAAAGCGGTTTACAACCCGAAGGCCGTCATCCCGCACGCGGCGTTGCTGCATCAGGCTTCCGCCCATTGTGCAATATTCCCCACTGCTGCCTCCCGTAGGAGTCTGGGCCGTATCTCAGTCCCAATGTGGCCGGTCGCCCTCTCAGGGCCGGCTACCCGTCAAAGCCTTGGTAAGCCACTACCCCACCAACAAGCTGATAAGCCGCGAGTCCATCC
>n.538.6.bb_1103
GTAGTTAGCCGTGGCTTTCTGATTAGGTACCGTCAAGACGTGCATAGTTACTTACACATTTGTTCTTCCCTAATAACAGAGTTTTACGATCCGAAGACCTTCATCACTCACGCGGCGTTGCTCCGTCAGGCTTTCGCCCATTGCGGAAGATTCCCTACTGCTGCCTCCCGTAGGAGTCTGGACCGTGTCTCAGTTCCAGTGTGGCCGATCACCCTCTCAGGGTCGGCTACGCATCGTTGCCTTGGTAAGCCGTTACCTTACCAACTAGCTAATGCGGCGCGGATCCATCTATAAGTGAC
>n.538.6.bb_1104
GTAGTTAGCCGTGGCTTTCTGATTAGGTACCGTCAAGACGTGCATAGTTACTTACACATTTGTTCTTCCCTAATAACAGAGTTTTACGATCCGAAGACCTTCATCACTCACGCGGCGTTGCTCCGTCAGGCTTTCGCCCATTGCGGAAGATTCCCTACTGCTGCCTCCCGTAGGAGTCTGGACCGTGTCTCAGTTCCAGTGTGGCCGATCACCCTCTCAGGGTCGGCTACGCATCGTTGCCTTGGTAAGCCGTTACCTTACCAACCAGCTAATGCGGCGCGGATCCATCTATAAGTGACAGCAAAACCGTCTTTCACT
>n.538.6.bb_1105
GTAGTTAGCCGTGGCTTTCTGATTAGGTACCGTCAAGACGTGCATAGTTACTTACACATTTGTTCTTCCCTAATAACAGAGTTTTACGATCCGAAGACCTTCATCACTCACGCGGCGTTGCTCCGTCAGGCTTTCGCCCATTGCGGAAGATTCCCTACTGCTGCCTCCCGTAGGAGTCTGGACCGTGTCTCAGTTCCAGTGTGGCCGATCACCCTCTCAGGGTCGGCTACGCATCGTTGCCTTGGTAAGCCGTTACCTTACCAACTAGCTAATGCGGCGCGGATCCATCTATAAGTGACAGCAAAACCGTCTTTCACTATTGAACCA
>n.538.6.bb_1106
GTAGTTAGCCGTGGCTTTCTGATTAGGTACCGTCAAGACGTGCATAGTTACTTACACATTTGTTCTTCCCTAATAACAGAGTTTTACGATCCGAAGACCTTCATCACTCACGCGGCGTTGCTCCGTCAGGCTTTCGCCCATTGCGGAAGATTCCCTACTGCTGCCTCCCGTAGGAGTCTGGACCGTGTCTCAGTTCCAGTGTGGCCGATCACCCTCTCAGGGTCGGCTACGCATCGTCGCCTTGGTAAGCCGTTACCTTACCAACTAGCTAATGCGGCGCGGATCCATCTATAAGTGACAGCAAAACCGTCTTTCACTATTGAACCATGCGGTTCAA
>n.538.6.bb_1107
GTAGTTAGCCGGTGCTTCTTCTGCAGGTACCGTCACCACAAGCTTCGCCCCTGCTGAAGCGGTTTACAACCCGAAGGCCGTCATCCCGCACGCGGCGTTGCTGCATCAGGCTTCCGCCCATTGTGCAATATTCCCCACTGCTGCCTCCCGTAGGAGTCTGGGCCGTATCTCAGTCCCAATGTGGCCGGTCGCCCTCTCAGGCCGGCTACCCGTCAAAGCCTTGGTAAGCCACTACCCACCAACAAGCTGATAAGCCGCGAGTCCATCCAAAACCGCCG
>n.538.6.bb_1108
GTAGTTAGCCGTGGCTTTCTGATTAGGTACCGTCAAGACGTGCATAGTTACTTACACATTTGTTCTTCCCTAATAACAGAGTTTTACGATCCGAAGACCTTCATCACTCACGCGGCGTTGCTCCGTCAGGCTTTCGCCCATTGCGGAAGATTCCCTACTGTTGCCTCCCGTAGGAGTCTGGACCGTGTCTCAGTTCCAGTGTGGCCGATCACCCTCTCAGGGTCGGCTACGCATCGTTGCCTTGGTAACGCCGTTACCTTACCAACTAGCTAATGCGGCGCGGATCCATCTATAAGTGACAG
>n.538.6.bb_1109
GTAGTTAGCCGTGGCTTTCTGATTAGGTACCGTCAAGACGTGCATAGTTACTTACACATTTGTTCTTCCCTAATAACAGAGTTTTACGATCCGAAGACCTTCATCACTCACGCGGCGTTGCTCCGTCAGGCTTTCGCCCATTGCGGAAGATTCCCTACCGCTGCCTCCCGTAGGAGTCTGGACCGTGTCTCAGTTCCAGTGTGGCCGATCCACCCTCTCAGGGTCGGCTACGCATCGTCGCCTTGGTAAGCCGTTACCTTACCAACTAGCTAATGCGGCGCGGATCCATCTATAAGTGAC
>n.538.6.bb_1110
GTAGTTAGCCGTGGCTTTCTGATTAGGTACCGTCAAGACGTGCATAGTTACTTACACATTTATTCTTCCCTAATAACAGAGTTTTACGATCCGAAGACCTTCATCACTCACGCGGCGTTGCTCCGTCAGGCTTTCGCCCATTGCGGAAGATTCCCTACTGCTGCCTCCCGTAGGAGTCTGGACCGTGTCTCAGTTCCAGTGTGGCCGATCACCCTCTCAGGGTCGGCTACGCATCGTTGCCTTGGTAAGCCGTTACCTTACCAACTAGCTAATGCGGCGCGGATCCATCTATAAGTGAC
>n.538.6.bb_1111
GGAGTTAGCCGGTGCTTCTTCTGCGGGTAACGTCAATCGACAAGGTTATTAACCTTATCGCCTTCCTCCCCGCTGAAAAGTACTTTACAACCCCGAAGGCCTTCTTCATACACGCGGCATGGCTGCATCAGGCTTGCGCCCATTGTGCAATATTCCCCATGCTGCCTCCGTAGGAGTCCTGGGACCGTGTCTCGAGTTCCAGTGTGGCTGGGTC
>n.538.6.bb_1112
GTAGTTAGCCGGTGCTTCTTCTGCAGGTACCGTCACCACAAGCTTCGCCCCTGCTGAAAGCGGTTTACAACCCGAAGGCCGTCATCCCGCACGCGGCGTTGCTGCATCAGGCTTCCGCCCATTGTGCAATATTCCCCACTGCTGCCTCCCGTAGGAGTCTGGGCCGTATCTCAGTCCCAATGTGGCCGGTCGCCCTCTCAGGCCGGCTACCCGGTCAAAGCCTTGGTAAGCCACTACCCCACCAACAAGCTGATAAGCCGCGAGTCCAT
>n.538.6.bb_1113
GTAGTTAGCCGGTGCTTCTTCTGCAGGTACCGTCACCACAAGCTTCGCCCCTGCTGAAAGCGGTTTACAACCCGAAGGCCGTCATCCCGCACGCGGCGTTGCTGCATCAGGCTTCCGCCCATTGTGCAATATTCCCCACTGCTGCCTCCCGTAGGAGTCTGGGCCGTATCTCAGTCCCAATGTGGCCGGTCGCCCTCTCAGGCCGGCTACCCGGTCAAAGCCTTGGTAACGCCACTACCCCACCAACAAGCTGATAAGCCGCGAGTCCATCCAAAACCG
>n.538.6.bb_1114
GAGTTAGCCGGTGCTTCTTCTGCGGGTAACGTCAATCGACAAGGTTATTAACCTTATCGCCTTCCTCCCCGCTGAAAGTACTTTACAACCCGAAGGCCTTCTTCATACACGCGGCATGGCTGCATCAGGCTTGCGCCCATTGTGCAATATTCCCCACTGCTGCCTCCCGTAGGAGTCTGGACCGTGTCTCAGTTCCAGTGTGGCTGGGTCATCCTCTCAGACCAGCTAGGGATCGTCGCCTAGGTGAGCCGTTACCCACCTACTAGCTAATCCCATCTGGGC
>n.538.6.bb_1115
GTAGTTAGCCGTGGCTTTCTGATTAGGTACCGTCAAGACGTGCATAGTTACTTACACATTTGTTCTTCCCTAATAACAGAGTTTTACGATCCGAAGACCTTCATCACTCACGCGGCGTTGCTCCGTCCGGCTTTCGCCCATTGCGGAAGATTCCCTACTGCTGCCTCCCGTAGGAGTCTGGACCGTGTCTCAGTTCCAGTGTGGCCGATCACCCTCTCAGGGTCGGCTACGCATCGTTGCCTTGGTAAGCCGTTACCTTACCAACTAGCTAATGCGGCGCGGATCCATCTATAAGTGACAGCAAAACCGTCTTTCACTATTGAACCATGCGGTTCAATATATTATCCCGGTATTAGCTCCGGTTT
>n.538.6.bb_1116
GTAGTTAGCCGGTGCTTCTTCTGCAGGTACCGTCACCACAAGCTTCGCCCCTGCTGAAAGCGGTTTACAACCCGAAGGCCGTCATCCCGCACGCGGCGTTGCTGCATCAGGCTTCCGCCCATTGTGCAATATTCCCCACTGCTGCCTCCCGTAGGAGTCTGGGCCGTATCTCAGTCCCAATGTGGCCGGTCGCCCTCTCAGGCCGGCTACCCGTCAAAGCCTTGGTAAGCCACTACCCACCAACAAGCTGATAAGCCGCGAGTCCATCCAAAACCGCCGAAGCTTTCCAACCCCCACCATGCAGCAAGGATTCCTATCCGGTATTAGCCCCAGTTTCCTGAAGTT
>n.538.6.bb_1117
GTAGTTAGCCGTGGCTTTCTGATTAGGTACCGTCAAGACGTGCATAGTTACTTACACATTTGTTCTTCCCTAATAACAGAGTTTTACGATCCGAAGACCTTCATCACTCACGCGGCGTTGCTCCGTCAGGCTTTCGCCCATTGCGGAAGATTCCCTACTGCTGCCTCCCGTAGGAGTCTGGACCGTGTCTCAGTTCCAGTGTGGCCGATCACCCTCTCAGGGTCGGCTACGCATCGTTGCCTTGGTAAGTCCGTTACCTTACCAACTAGCTAATGCGGCGCGGATCCATCTATAAGTGA
>n.538.6.bb_1118
GTAGTTAGCCGGTGCTTCTTCTGCAGGTACCGTCACCACAAGCTTCGCCCCTGCTGAAAGCGGTTTACAACCCGAAGGCCGTCATCCCGCACGCGGCGTTGCTGCATCAGGCTTCCGCCCATTGTGCAATATTCCCCACTGCTGCCTCCCGTAGGAGTCTGGGCCGTATCTCAGTCCCAATGTGGCCGGTCGCCCTCTCAGGCCGGCTACCCG
>n.538.6.bb_1119
TAGTTAGCCGTGGCTTTCTGATTAGGTACCGTCAAGACGTGCATAGTTACTTACACATTTGTTCTTCCCTAATAACAGAGTTTTACGATCCGAAGACCTTCATCACTCACGCGGCGTTGCTCCGTCAGGCTTTCGCCCATTGCGGAAGATTCCCTACTGCTGCCTCCCGTAGGAGTCTGGACCGTGTCTCAGTTCCAGTGTGGCCGATCACCCTCTCAGGGTCGGCTACGCATCGTTGCCTTGGTAAGCCGTTACCTTACCAACTAGCTAATGCGGCGCGGATCCATCTATAAGTGACAGCAAAACCGTCTTTCACTATTGAACCATGCGGTTCAATATATTATCCCGGTATT
>n.538.6.bb_1120
TAGTTAGCCGTGGCTTTCTGATTAGGTACCGTCAAGACGTGCATAGTTACTTACACATTTATTCTTCCCTAATAACAGAGTTTTACGATCCGAAGACCTTCATCACTCACGCGGCGTTGCTCCGTCAGGCTTTCGCCCATTGCGGAAGATTCCCTACTGCTGCCTCCCGTAGGAGTCTGGACCGTGTCTCAGTTCCAGTGTGGCCGATCACCCTCTCAGGGTCGGCTACGCATCGTTGCCTTGGTAAGCCGTTACCTTACCAACTAGCTAATGCGGCGCGGATCCATCTATAAGTGACA
>n.538.6.bb_1121
GTAGTTAGCCGTGGCTTTCTGATTAGGTACCGTCAAGACGTGCATAGTTACTTACACATTTGTTCTTCCCTAATAACAGAGTTTTACGATCCGAAGACCTTCATCACTCACGCGGCGTTGCTCCGTCAGGCTTTCGCCCATTGCGGAAGATTCCCTACTGCTGCCTCCCGTAGGAGTCTGGACCGTGTCTCAGTTCCAGTGTGGCCGATCACCCTCTCAGGGTCGGCTACGCATCGTTGCCTTGGTAAGCCGTTACCTTACCAACTAGCTAATGCGGCGCGGATCCATCTATAAGTGACAGCAAAACCGTCTTTCACTATTGAACCATGCGGTTCAATATATTATCCGGTATT
>n.538.6.bb_1122
GTAGTTAGCCGTGGCTTTCTGATTAGGTACCGTCAAGACGTGCATAGTTACTTACACATTTGTTCTTCCCTAATAACAGAGTTTTACGATCCGAAGACCTTCATCACTCACGCGGCGTTGCTCCGTCAGGCTTTCGCCCATTGCGGAAGATTCCCTACTGCTGCCTCCCGTAGGAGTCTGGACCGTGTCTCAGTTCCAGTGTGGCCGATCACCCTNTCAGGGTCGGCTACGCATCGTTGCCTTGGTAAGCCGTTACCTTACCAACTAGCTAATGCGGCGCGGATCCATCTATAAGTGACAGCAAAACCGTCTTTCACTATTGAACCATGCGGTTCAATATATTATCCGGTATTAGCTCCGGTTTCCCGAAGTTATCCCAGTCTTATAGGTAGGTTATC
>n.538.6.bb_1123
GTAGTTAGCCGGTGCTTCTTCTGCAGGTACCGTCACCACAAGCTTCGCCCCTGCTGAAAGCGGTTTACAACCCGAAGGCCGTCATCCCGCACGCGGCGTTGCTGCATCAGGCTTCCGCCCATTGTGCAATATTCCCCACTGCTGCCTCCCGTAGGAGTCTGGGCCGTATCTCAGTCCCAATGTGGCCGGTCGCCCTCTCAGGCCGGCTA
>n.538.6.bb_1124
GTAGTTAGCCGGTGCTTCTTCTGCAGGTACCGTCACCACAAGCTTCGCCCCTGCTGAAAGCGGTTTACAACCCGAAGGCCGTCATCCCGCACGCGGCGTTGCTGCATCAGGCTTCCGCCCATTGTGCAATATTCCCCACTGCTGCCTCCCGTAGGAGTCTGGGCCGTATCTCAGTCCCAATGTGGCCGGTCGCCCTCTCAGGCCGGCTACCCGGTNAAAGCCTTGGTAAGCCACTACCCCACCAACAAGCTGATAAGCCGCGAGTCCATCCAAAACCGCCG
>n.538.6.bb_1125
GTAGTTAGCCGTGGCTTTCTGATTAGGTACCGTCAAGACGTGCATAGTTACTTACACATTTGTTCTTCCCTAATAACAGAGTTTTACGATCCGAAGACCTTCATCACTCACGCGGCGTTGCTCCGTCAGGCTTTCGCCCATTGCGGAAGATTCCCTACTGCTGCCTCCCGTAGGAGTCTGGACCGTGTCTCAGTTCCAGTGTGGCCGATCACCCTCTCAGGGTCGGCTACGCATCGTTGCCTTGGTAAGCCGTTACCTTACCAACTAGTTGATGCGGCGCGGATCCATCTATAAGTGACAGCAAAACCGTCTTTCACTATTGAACCATGCGGTTCAATA
>n.538.6.bb_1126
GTAGTTAGCCGGTGCTTCTTCTGCGGGTAACGTCAATTGCTGAGGTTATTAACCTCAACACCTTCCTCCCCGCTGAAAGTACTTTACAACCCGAAGGCCTTCTTCATACACGCGGCATGGCTGCATCAGGCTTGCGCCCATTGTGCAATATTCCCCACTGCTGCCTCCCGTAGGAGTCTGGACCGTGTCTCAGTTCCAGTGTGGCTGGGTCATCCTCTCAGACCAGCTAGGGATCGTCGCCTAGGT
>n.538.6.bb_1127
GTAGTTAGCCGTGGCTTTCTGATTAGGTACCGTCAAGACGTGCATAGTTACTTACACATTTTGTTCTTCCCTAATAACAGAGTTTTACGATCCGAAGACCTTCATCACTCACGCGGCGTTGCTCCGTCCGGCTTTCGCCCATTGCGGAAGATTCCCTACTGCTGCCTCCCGTAGGAGTCTGGACCGTGTCTCAGTTCCAGTGTGGCCCGATCACCCTNTCAGGGTCGGGCTACGCATCGTTGCCTTGGTAAGCCGTTACCTTACCAACTAGCTAATGCGGCGCGGATCCATCTATAAGTGACAGCAAAAACGTCTTTCACTATTGAACCATGCGGT
>n.538.6.bb_1128
GTAGTTAGCCGGTGCTTCTTCTGCAGGTACCGTCACCACAAGCTTCGCCCCTGCTGAAAGCGGTTTACAACCCGAAGGCCGTCATCCCGCACGCGGCGTTGCTGCATCAGGCTTCCGCCCATTGCGGAAGATTCCCTACTGCTGCCTCCCGTAGGAGTCTGGACCGTGTCTCAGTTCCAGTGTGGCCGATCACCCTCTCAGGTCGGCTACGCATCGTTGCCTTGGGTAAGCCCGTTACCTTACCAACTAGCTAATGCGGCGCGGATCCATCTATAAGTGAC
>n.538.6.bb_1129
GTAGTTAGCCGTGGCTTTCTGATTAGGTACCGTCAAGACGTGCATAGTTACTTACACATTTGTTCTTCCCTAATAACAGAGTTTTACGATCCGAAGACCTTCATCACTCACGCGGCGTTGCTCCGTCAGGCTTTCGCCCATTGCGGAAGATTCCCTACTGCTGCCTCCCGTAGGAGTCTGGACCGTGTCTCAGTTCCAGTGTGGCCGATCACCCTCTCAGGGTCGGCTACGCATCGTTGCCTTGGTAAGCCGTTACCTTACCAACTAGCTAATGCGGCGCGGATCCATCTAT
>n.538.6.bb_1130
GTAGTTAGCCGGTGCTTCTTCTGCAGGTACCGTCACCACAAGCTTCGCCCCTGCTGAAAGCGGTTTACAACCCGAAGGCCGTCATCCCGCACGCGGCGTTGCTGCATCAGGCTTCCGCCCATTGTGTAATATTCCCCACTGCTGCCTCCCGTAGGAGTCTGGGCCGTATCTCAGTCCCAATGTGGCCGGTCGCCCTCTCAGGCCGGCTACCCGTCAAAGCCTTGGTAAGCCACTACCCCCCAACAAGCTGATAAGCCGCGAGTCCATCCAAAACCG
>n.538.6.bb_1131
GGTAGTTAGCCGGTGCTTCTTCTGCGGGTAACGTCAATCGACAAGGTTATTAACCTTATCGCCTTCCTCCCCGCTGAAAGTACTTTACAACCCGAAGGCCTTCTTCATACACGCGGCATGGCTGCATCAGGCTTGCGCCCATTGTGCAATATTCCCCACTGCTGCCTCCGTAGGAGTCTGGACCGTGTCTCAGTTCCAGTGTGGCTGGTCATCCTCTCAGACCAGCTAGGGATCGTCGCCTAGGTGACGCCGTTACCCACCTACTAGCTAATCCCATC
>n.538.6.bb_1132
GTAGTTAGCCGTGGCTTTCTGATTAGGTACCGTCAAGACGTGCATAGTTACTTACACATTTGTTCTTCCCTAATAACAGAGTTTTACGATCCGAAGACCTTCATCACTCACGCGGCGTTGCTCCGTCAGGCTTTCGCCCATTGCGGAAGATTCCCTACTGCTGCCTCCCGTAGGAGTCTGGACCGTGTCTCAGTTCCAGTGTGGCCGATCACCCTCTCAGGGTCGGCTACGCATCGTTGCCTTGGTAAGCCGTTACCTTACCAACTAGCTAATGCGGCGCGGATCCATCTATAAGTGA
>n.538.6.bb_1133
GTAGTTAGCCGGTGCTTCTTCTGCAGGTACCGTCACCACAAGCTTCGCCCCTGCTGAAAGCGGTTTACAACCCGAAGGCCGTCATCCCGCACGCGGCGTTGCTGCATCAGGCTTCCGCCCATTGTGCAATATTCCCCACTGCTGCCTCCCGTAGGAGTCTGGGCCGTATCTCAGTCCCAATGTGGCCGGTCGCCCTCTCAGGCCGGCTACCCGGTCAAAGCCTTGGTAAGCCACTACCCCACCAACAAGCTGATAAGCCGCGAGTCCATCCAAAACCGC
>n.538.6.bb_1134
GTAGTTAGCCGTGGCTTTCTGATTAGGTACCGTCAAGACGTGCATAGTTACTTACACATTTGTTCTTCCCTAATAACAGAGTTTTACGATCCGAAGACCTTCATCACTCACGCGGCGTTGCTCCGTCAGGCTTTCGCCCATTGCGGAAGATTCCCTACTGCTGCCTCCCGTAGGAGTCTGGACCGTGTCTCAGTTCCAGTGTGGCCGATCACCCTCTCAGGGTCGGCTACGCATCGTTGCCTTGGTAAGCCGTTACCTTACCAACTAGCTAATGCGGCGCGGATCCATCTATAAGTGACAGCAAAACCGTCTTTCACTATTGAACCATGCGGTTCAATATATT
>n.538.6.bb_1135
TAGTTAGCCGTGGCTTTCTGATTAGGTACCGTCAAGACGTGCATAGTTACTTACACATTTGTTCTTCCCTAATAACAGAGTTTTACGATCCGAAGACCTTCATCACTCACGCGGCGTTGCTCCGTCAGGCTTTCGCCCATTGCGGAAGATTCCCTACTGCTGCCTCCCGTAGGAGTCTGGACCGTGTCTCAGTTCCAGTGTGGCCCGATCACCCTCTCAGGGTCGGCTACGCATCGTCGCCTTGGTAAGCCGTTACCTTACCAACTAGCTAATGCGGCGCGGATCCATCTATAAGTGACAGCAAAACCGTCTTTCACTATTGAACCATGCGGTTCAATATATTATCCGGTATTAGCTCCGGTTTCCCCGAAGTTATCCCAGTCTTATAGGT
>n.538.6.bb_1136
GGAGTTAGCCGGTGCTTCTTCTGCGGGTAACGTCAATTGCTGAGGTTATTAACCTCAACACCTTCCTCCCCGCTGAAAGTACTTTACAACCCGAAGGCCTTCTTCATACACGCGGCATGGCTGCATCAGGCTTGCGCCCATTGTGCAATATTCCCCACGCTGCCTCCCGTAGGAGTCTGGACCGTGTCTCAGTTCCAGTGTGGCTGGTCATCCTCTCAGACCAGCTAGGGGATCGTCGCCTAGGTGAGCCGTTACCCACCTACTAGCTAATCCCATCTGGGCACATCTGATGGC
>n.538.6.bb_1137
GTAGTTAGCCGTGGCTTTCTGATTAGGTACCGTCAAGACGTGCATAGTTACTTACACATTTGTTCTTCCCTAATAACAGAGTTTTACGATCCGAAGACCTTCATCACTCACGCGGCGTTGCTCCGTCAGGCTTTCGCCCATTGCGGAAGATTCCCTACTGCTGCCTCCCGTAGGAGTCTGGACCGTGTCTCAGTTCCAGTGTGGCCGATCACCCTCTCAGGGTCGGCTACGCATCGTTGCCTTGGTAAGCCGTTACCTTACAACTAGCTAATGCGGCGCGGATCCATCTATAAGTGACAGCAAAACCGTCTTTCACTATTGAACCATGCGGTTCAATATATATCCGGTATTAGCTCCGG
>n.538.6.bb_1138
GTAGTTAGCCGTGGCTTTCTGATTAGGTACCGTCAAGACGTGCATAGTTACTTACACATTTGTTCTTCCCTAATAACAGAGTTTTACGATCCGAAGACCTTCATCACTCACGCGGCGTTGCTCCGTCAGGCTTTCGCCCATTGCGGAAGATTCCCTACTGCTGCCTCCCGTAGGAGTCTGGACCGTGTCTCAGTTCCAGTGTGGCCGATCACCCTCTCAGGGTCGGCTACGCATCGTCGCCTTGGTAAGCCGTTACCTTACCAACTAGCTAATGCGGCGCGGATCCATCTATAAGTGAC
>n.538.6.bb_1139
GTAGTTAGCCGGTGCTTCTTCTGCAGGTACCGTCACCACAAGCTTCGCCCCTGCTGAAAGCGGTTTACAACCCGAAGGCCGTCATCCCGCACGCGGCGTTGCTGCATCAGGCTTCCGCCCATTGTGCAATATTCCCCACTGCTGCCTCCCGTAGGAGTCTGGGCCGTATCTCAGTCCCAATGTGGCCGGTCGCCCTCTCAGGCCGGCTACCCGTCAAAGCCTTGGTAAGCCACTACCCACCAACAAGCTGATAAGCCGCGAGTCCATCCAAAACCGCCG
>n.538.6.bb_1140
GTAGTTAGCCGGTGCTTCTTCTGCAGGTACCGTCACCACAAGCTTCGCCCCTGCTGAAAGCGGTTTACAACCCGAAGGCCGTCATCCCGCACGCGGCGTTGCTGCATCAGGCTTCCGCCCGTTGTGCAATATTCCCCACTGCTGCCTCCCGTAGGAGTCTGGGCCGTATCTCAGTCCCAATGTGGCCGTCGCCCTCTCAGGCCGGCTACCCGGTCAAAGCCTTGGTAACNCCACTACCCACAACAAGCTGATAAGCCGCGAGTCCAT
>n.538.6.bb_1141
GTAGTTAGCCGTGGCTTTCTGATTAGGTACCGTCAAGACGTGCATAGTTACTTACACATTTATTCTTCCCTAATAACAGAGTTTTACGATCCGAAGACCTTCATCACTCACGCGGCGTTGCTCCGTCAGGCTTTCGCCCATTGCGGAAGATTCCCTACTGCTGCCTCCCGTAGGAGTCTGGACCGTGTCTCAGTTCCAGTGTGGC
>n.538.6.bb_1142
GGAGTTAGCCGGTGCTTCTTCTGCGGGTAACGTCAATCGACAAGGTTATTAACCTTATCGCCTTCCTCCCCGCTGAAAGTACTTTACAACCCGAAGGCCTTCTTCATACACGCGGCATGGCTGCATCAGGCTTGCGCCCATTGTGCAATATTCCCCACTACTGCCTCCCGTAGGAGTCTGGACCGTGTCTCAGTTCCAGTGTGGCTGGTCATCCTCTCAGACCAGCTAGGGATCGTCGCCTAGGTGAGCCGTTACCCACCTACTAGCTAATCCCATCTGGGCACATC
>n.538.6.bb_1143
GGAGTTAGCCGGTGCTTCTTCTGCGGGTAACGTCAATCGACAAGGTTATTAACCTTATCGCCTTCCTCCCCGCTGAAAGTACTTTACAACCCGAAGGCCTTCTTCATACACGCGGCATGGCTGCATCAGGCTTGCGCCCATTGTGCAATATTCCCCATGCTGCCTCCGTAGGAGTCTGGACCGTGTCTCAGTTCCAGTGTGGCTGGTCATCCTCTCAGACCAGCTAGGGATCGTCGCCTAGGTGAGCCGTTACCCACCTACTAGCTAATCCCATCTGGGCACATCTG
>n.538.6.bb_1144
GTAGTTAGCCGTGGCTTTCTGATTAGGTACCGTCAAGACGTGCGTAGTTACTTACACATTTGTTCTTCCCTAATAACAGAGTTTTACGATCCGAAGACCTTCATCACTCACGCGGCGTTGCTCCGTCAGGCTTTCGCCCATTGCGGAAGATTCCCTACTGCTGCCTCCCGTAGGAGTCTGGACCGTGTCTCAGTTCCAGTGTGGCCGATCACCCTCTCAGGGTCGGCTACGCATCGTCGCCTTGGTAAGCCGTTACCTTACCAACTAGCTAATGCGGCGCGGATCCATCTATAAGTGACAGCAAAACGTCTTTCACTATTGAACCATGCGGTTCAATATATTATCCGGTATT
>n.538.6.bb_1145
GTAGTTAGCCGTGGCTTTCTGATTAGGTACCGTCAAGACGTGCATAGTTACTTACACATTTGTTCTTCCCTAATAACAGAGTTTTACGATCCGAAGACCTTCATCACTCACGCGGCGTTGCTCCGTCAGGCTTTCGCCCATTGCGGAAGATTCCCTACTGCTGCCTCCCGTAGGAGTCTGGACCGTGTCTCAGTTCCAGTGTGGCCGATCACCCTCTCAGGGTCGGCTACGCATCGTTGCCTTGGTAAGCCGTTACCTTACCAACTAGCTAATGCGGCGCGGATCCATCTATAAGTGACAGCAAAACCGTCTTTCACTATTGAACCAT
>n.538.6.bb_1146
GGAGTTAGCCGGTGCTTCTTCTGCGGTAACGTCAATTGCTGAGGTTATTAACCTCAACACCTTCCTCCCCGCTGAAACGTACTTTACAACCCGAAGGCCTTCTTCATACACGCGGCATGGCTGCATCAGGCTTGCGTCCCATTGTGCAAATATTCCCCACTGCTGCCTCCCGTAGGAGTCTGGGACCGTGTCTCAGTTCCAGTGTG
>n.538.6.bb_1147
GGAGTTAGCCGGTGCTTCTTCTGCGGGTAACGTCAATTGCTGAGGTTATTAACCTCAACACCTTCCTCCCCGCTGAAAGTACTTTACAACCCGAAGGCCTTCTTCATACACGCGGCATGGCTGCATCAGGCTTGCGCCCATTGTGCAATATTCCCCACTGCTGCCTCCCGTAGGAGTCTGGACCGTGTCTCAGTTCCAGTGTGGCTGGGTCATCCTCTCAGACCAGCTAGGGATCGTCGCCTAGGTGAGCCGTTACCCCACCTACTAGCTAATCCCATCTGGGCACAT
>n.538.6.bb_1148
GTAGTTAGCCGTGGCTTTCTGATTAGGTACCGTCAAGACGTGCATAGTTACTTACACATTTGTTCTTCCCTAATAACAGAGTTTTACGATCCGAAGACCTTCATCACTCACGCGGCGTTGCTCCGTCAGGCTTTCGCCCATTGCGGAAGATTCCCTACTGCTGCCTCCCGTAGGAGTCTGGACCGTGTCTCAGTTCCAGTGTGGCCGATCACCCTCTCAGGGTCGGCTACGCATCGTTGCCTTGGTAAGCCGTTACCTTACCAACTAGCTAATGCGGCGCGGATCCATCTATAAGTGACAGCAAAACCGTCTTTCACTATTGAACCATGCGG
>n.538.6.bb_1149
GTAGTTAGCCGTGGCTTTCTGATTAGGTACCGTCAAGACGTGCATAGTTACTTACACATATATTCTTCCCTAATAACAGAGTTTTACGATCCGAAGACCTTCATCACTCACGCGGCGTTGCTCCGTCAGGCTTTCGCCCATTGCGGAAGATTCCCTACTGCTGCCTCCCGTAGGAGTCTGGACCGTGTCTCAGTTCCAGTGTGGCCGATCACCCTCTCAGGTCGGCTACGCATCGTGCCTTGGTAAGCCGTTACCTTACCAACTAGCTAATGCGGCGCGGATCCATCTATAAGTGACAGCAAAACCGTCTTTCACTATTGAACCATGCGGTTCAATATATTATCCGGTATT
>n.538.6.bb_1150
GGAGTTAGCCGGTGCTTCTTCTGCGGGTAACGTCAATTGCTGAGGTTATTAACCTCAACACCTTCCTCCCCGCTGAAAGTACTTTACAACCCGAAGGCCTTCTTCATACACGCGGCATGGCTGCATCAGGCTTGCGCCCATTGCGCAATATTCCCCACTGCTGCCTCCCGTAGGAGTCTGGACCGTGTCTCAGTTCCAGTGTGGCTGGTCATCCTCTCAGACCAGCTAGGGATCGTCGCCTAGGTGACGCCGTTACCCACCTACTAGCTAATCCCATCTGGGCACATCTGAT
>n.538.6.bb_1151
GTAGTTAGCCGTGGCTTTCTGATTAGGTACCGTCAAGACGTGCATAGTTACTTACACATTTGTTCTTCCCTAATAACAGAGTTTTACGATCCGAAGACCTTCATCACTCACGCGGCGTTGCTCCGTCAGGCTTTCGCCCATTGCGGAAGATTCCCTACTGCTGCCTCCCGTAGGAGTCTGGACCGTGTCTCAGTTCCAGTGTGGCCGATCACCCTCTCAGGGTCGGCTACGCATCGTTGCCTTGGTAAGCCGTTACCTTACCAACTAGCTAATGCGGCGCGGATCCATCTATAAGTGACAGCAAAACCGTCTTTCACTATTGAACCATGCGGTTCAATATATT
>n.538.6.bb_1152
GTAGTTAGCCGTGGCTTTCTGATTAGGTACCGTCAAGACGTGCATAGTTACTTACACATTTGTTCTTCCCTAATAACAGAGTTTTACGATCCGAAGACCTTCATCACTCACGCGGCGTTGCTCCGTCAGGCTTTCGCCCATTGCGGAAGATTCCCTACTGCTGCCTCCCGTAGGAGTCTGGACCGTGTCTCAGTTCCAGTGTGGCCGATCACCCTCTCAGGGGTCGGCTACGCATCGTCGCCTTGGTAAGCCGTTACCTTACCAACTAGCTAATGCGGCGCGGATCCATCTATAAGTGACAGCAAAACCGTCTTTCACTATTGAACCATGCGGTTCAATATATTATCC
>n.538.6.bb_1153
GTAGTTAGCCGTGGCTTTCTGATTAGGTACCGTCAAGACGTGCATAGTTACTTACACATTTGTTCTTCCCTAATAACAGAGTTTTACGATCCGAAGACCTTCATCACTCACGCGGCGTTGCTCCGTCAAGCTTTCGCCCATTGCGGAAGATTCCCTACTGCTGCCTCCCGTAGGAGTCTGGACCGTGTCTCAGTTCCAGTGTGGCCGATCACCCTNTCAGGGTCGGCTACGCATCGTTGCCTTGGTAAGCCGTTACCTTACCAACTAGCTAATGCGGCGCGGATCCATCTATAAGTGAC
>n.538.6.bb_1154
GTAGTTAGCCGTGGCTTTCTGATTAGGTACCGTCAAGACGTGCATAGTTACTTACACATTTGTTCTTCCCTAATAACAGAGTTTTACGATCCGAAGACCTTCATCACTCACGCGGCGTTGCTCCGTCAGGCTTTCGCCCATTGCGGAAGATTCCCCACTGCTGCCTCCCGTAGGAGTCTGGGCCGTATCTCAGTCCCAATGTGGCCGGTCGCCC
>n.538.6.bb_1155
GTAGTTAGCCGGTGCTTCTTCTGCAGGTACCGTCAAGACGTGCATAGTTACTTACACATTTGTTCTTCCCTAATAACAGAGTTTTACGATCCGAAGACCTTCATCACTCACGCGGCGTTGCTCCGTCAGGCTTTCGCCCATTGCGGAAGATTCCCTACTGCTGCCTCCGTAGGAGTCTGGACCGTGTCTCAGTTCCAGTGTGGCCGATCACCCTCTCAGGGTCGGCTACGCATCGTTGCCTTGGTAAGCCGTTACCTTACCAACTAGCTAATGCGGCGCGGATCCATCTATAAGTGACAGCAAAACCGTCTTTCACTATTGAACCATGCGGTTCAATATATTATCCGGTATT
>n.538.6.bb_1156
GAAGTTAGCCGGTGCTTCTTCTGCGGGTAACGTCAATCGACAAGGTTATTAACCTTATCGCCTTCCTCCCCGCTGAAAGTACTTTACAACCCGAAGGCCTTTCTTCATACACGCGGCATGGCTGCATCAGGCTTGCGCCCATTGTGCAATATTCCCCACTGCTGCCTCCCGTAGGAGTCTGGACCGTGTCTCAGTTCCAGTGTGGCTGGTCATCCTCTCAGACCAGCTAGGGATCGTCGCCTAGGTGAGCCGTTACCCACCTACTAGCTAATCCCATCTGGGCACATCTGATGGCAAGAGGCCCGAAGGT
>n.538.6.bb_1157
GTAGTTAGCCGGTGCTTCTTCTGCAGGTACCGTCACCACAAGCTTCGCCCCTGCTGAAAGCGGTTTACAACCCGAAGGCCGTCATCCCGCACGCGGCGTTGCTGCATCAGGCTTCCGCCCATTGTGCAATATTCCCCACTGCTGCCTCCCGTAGGAGTCTGGGCCGTATCTCAGTCCCAATGTGGCCGGTCGCCCTCTCAGGCCGGCTACCCGTCAAAGGCCTTGGTAACGCCGTTACCTTACCAACTAGCTAATGCGGCGCGGATCCATCTATAAGTGAC
>n.538.6.bb_1158
GTAGTTAGCCGGTGCTTCTTCTGCAGGTACCGTCACCACAAGCTTCGCCCCTGCTGAAAGCGGTTTACAACCCGAAGGCCGTCATCCCGCACGCGGCGTTGCTGCATCAGGCTTCCGCCCATTGTGCAATATTCCCCACTGCTGCCTCCCGTAGGAGTCTGGGCCGTATCTCAGTCCCAATGTGGCCGGTCGCCCTCTCAGGCCGGCTACCCGTCAAAGCCTTGGTAAGCCACTACCCACCAACAAGCTGATAAGCCGCGAGTCCATCCAAAACCGCCGA
>n.538.6.bb_1159
GTAGTTAGCCGTGGCTTTCTGATTAGGTACCGTCAAGACGTGCATAGTTACTTACACATTTGTTCTTCCCTAATAACAGAGTTTTACGATCCGAAGACCTTCATCACTCACGCGGCGTTGCTCCGTCAGGCTTTCGCCCATTGCGGAAGATTCCCTACTGCTGCCTCCCGTAGGAGTCTGGACCGTGTCTCAGTTCCAGTGTGGCCGATCACCCTCTCAGGGTCGGCTACGCATCGTCGCCTTGGTAAGCCGTTACCTTACCAACTAGCTAATGCGGCGCGGATCCATCTATAAGTGACAGCAAAACCGTCTTTCACTATTGAACCATGCGGTTCAATATATTATCC
>n.538.6.bb_1160
GTAGTTAGCCGGTGCTTCTTCTGCAGGTACCGTCACCACAAGCTTCGCCCCTGCTGAAAGCGGTTTACAACCCGAAGGCCGTCATCCCGCACGCGGCGTTGCTGCATCAGGCTTCCGCCCATTGTGCAATATTCCCCACTGCTGCCTCCCGTAGGAGTCTGGGCCGTATCTCAGTCCCAATGTGGCCGGTCGCCCTCTCAGGCCGGCTACCCGGTCAAAGCCTTGGTAAGCCACTACCCCACCAACAAGCTGATAAGCCGCGAGTCCATCCAAAACCGC
>n.538.6.bb_1161
GGAGTTAGCCGGTGCTTCTTCTGCGGGTAACGTCAATCGACAAGGTTATTAACCTTATCGCCTTCCTCCCCGCTGAAAGTACTTTACAACCCGAAGGCCTTCTTCATACACGCGGCATGGCTGCATCAGGCTTGCGCCCATTGTGCAATATTCCCCACTGCTGCCTCCCGTAGGAGTCTGGACCGTGTCTCAGTTCCAGTGTGGCTGGGTCATCCTCTCAGACCAGCTAGGGGATCGTCGCCTAGGGTGAGCCGTTACCCACCTACTAGCTAATCCCAT
>n.538.6.bb_1162
GTAGTTAGCCGTGGCTTTCTGATTAGGTACCGTCAAGACGTGCATAGTTACTTACACATTTGTTCTTCCCTAATAACAGAGTTTTACGATCCGAAGACCTTCATCACTCACGCGGCGTTGCTCCGTCAGGCTTTCGCCCATTGCGGAAGATTCCCTACTGCTGCCTCCCGTAGGAGTCTGGACCGTGTCTCAGTTCCAGTGTGGCCGATCACCCTCTCAGGTCGGCTACGCATCGTTGCCTTGGTAAGCCGTTACCTTACCAACTAGCTAATGCGGCGCGGATCCATCTATAAGTGACAGCAAAACCGTCTTTCACTATTGAACCATGCGGTTCAATATATT
>n.538.6.bb_1163
GTAGTTAGCCGGTGCTTCTTCTGCAGGTACCGTCACCACAAGCTTCGCCCCTGCTGAAAGCGGTTTACAACCCGAAGGCCGTCATCCCGCACGCGGCGTTGCTGCATCAGGCTTCCGCCCATTGTGCAATATTCCCCACTGCTGCCTCCCGTAGGAGTCTGGGCCGTATCTCAGTCCCAATGTGGCCGGTCGCCCTCTCAGGGCCGGCTACCCGTNAAAGCCTTGGTAAGCCACTACCCACCAACAAGCTGATAAGCCGCGAGTCCATCCAAAACGCCGAAGCTTTCCAACCCCACCATGCAGCAAGGATTCCTATCCGGTATTA
>n.538.6.bb_1164
GTAGTTAGCCGTGGCTTTCTGATTAGGTACCGTCAAGACGTGCATAGTTACTTACACATTTGTTCTTCCCTAATAACAGAGTTTTACGATCCGAAGACCTTCATCACTCACGCGGCGTTGCTCCGTCAGGCTTTCGCCCATTGCGGAAGGTTCCCTACTGCTGCCCTCCCGTAGGAGTCTGGACCGTGTCTCAGTTCCAGTGTGGCCGATCACCCTCTCAGGGGTCGGCTACGCATCGTCGCCTTGGTAAGCCGTTACCTTACCAACTAGCTAATGCGGCGCGGATCCATCTATAAGTGACAGCAAAACCGTCTTTCACTA
>n.538.6.bb_1165
TAGTTAGCCGGTGCTTCTTCTGCAGGTACCGTCACCACAAGCTTCGCCCCTGCTGAAAGCGGTTTACAACCCGAAGGCCGTCATCCCGCACGCGGCGTTGCTGCATCAGGCTTCCGCCCATTGTGCAATATTCCCCACTGCTGCCTCCCGTAGGAGTCTGGGCCGTATCTCAGTCCCAATGTGGCCGGTCGCCCTCTCAGGCCGGCTACCCGGTCAAAGCCTTGGTAAGCCACTACCCCACCAACAAGCTGATAAGCCGCGAGTCCATCCAAAACCGC
>n.538.6.bb_1166
GTAGTTAGCCGGTGCTTCTTCTGCAGGTACCGTCACCACAAGCTTCGCCCCTGCTGAAAGCGGTTTACAACCCGAAGGCCGTCATCCCGCACGCGGCGTTGCTGCATCAGGCTTCCGCCCATTGTGCAATATTCCCCACTGCTGCCTCCCGTAGGAGTCTGGGCCGTATCTCAGTCCCAATGTGGCCGGTCGCCCTCTCAGGCCGGCTACCCGTCAAAGCCTTGGTAAGCCACTACCCACCAACAAGCTGATAAGCCGCGAGTCCATCCAAAACCGC
>n.538.6.bb_1167
GTAGTTAGCCGGTGCTTCTTCTGCAGGTACCGTCACCACAAGCTTCGCCCCTGCTGAAAGCGGTTTACAACCCGAAGGCCGTCATCCCGCACGCGGCGTTGCTGCATCAGGCTTCCGCCCATTGTGCAATATTCCCCACTGCTGCCTCCCGTAGGAGTCTGGGCCGTATCTCGGTCCCAATGTGGCCGGTCGCCCTCTCAGGCCGGCTACCCGGTCAAAGCCTTGGTAAGCCACTACCCACCAACAAGCTGATAAGCCGCGAGTCCATCCAAAACCGCC
>n.538.6.bb_1168
GTAGTTAGCCGGTGCTTCTTCTGCAGGTACCGTCACCACAAGCTTCGCCCCTGCTGAAAGCGGTTTACAACCCGAAGGCCGTCATCCCGCACGCGGCGTTGCTGCATCAGGCTTCCGCCCATTGTGCAATATTCCCCATGCTGCCTCCCGTAGGAGTCTGGGCCTATCTCAGTCCCAATGTGGCCGTCGCCCTCTCAGGCCGGCTACCCGTCAAAGCCTTGGTAAGCCACTACCCCACCAACAAGCTGATAAGCCGCGAGTCC
>n.538.6.bb_1169
GTAGTTAGCCGGTGCTTCTTCTGCAGGTACCGTCACCACAAGCTTCGCCCCTGCTGAAAGCGGTTTACAACCCGAAGGCCGTCATCCCGCACGCGGCGTTGCTGCATCAGGCTTCCGCCCATTGTGCAATATTCCCCACTGCTGCCTCCCGTAGGAGTCTGGGCCGTATCTCAGTCCCAATGTGGCCGGTCGCCCTCTCAGGCCGGCTACCCGGTNAAAGCCTTGGTAACGCCACTACCCCACCAACAAGCTGATAAGCCGCGAGTCCA
>n.538.6.bb_1170
GTAGTTAGCCGTGGCTTTCTGATTAGGTACCGTCAAGACGTGCATAGTTACTTACACATTTATTCTTCCCTAATAACAGAGTTTTACGATCCGAAGACCTTCATCACTCACGCGGCGTTGCTCCGTCAGGCTTTCGCCCATTGCGGAAGATTCCCTACTGCTGCCTCCCGTAGGAGTCTGGACCGTGTCTCAGTTCCAGTGTGGCCGATCACCCTCTCAGGGTCGGCTACGCATCGTTGCCTTGGTAAGCCGTTACCTTACCAACTAGCTAATGCGGCGCGGATCCATCTATAAGTGACAGCAAAACCGTCTTTCACTATTGAACCATGCGGTTCAATATATTATCCGGTATT
>n.538.6.bb_1171
GTAGTTAGCCGTGGCTTTCTGATTAGGTACCGTCAAGACGTGCATAGTTACTTACACATTTGTTCTTCCCTAATAACAGAGTTTTACGATCCGAAGACCTTCATCACTCACGCGGCGTTGCTCCGTCAGGCTTTCGCCCATTGCGGAAGATTCCCTACTGCTGCCTCCCGTAGGAGTCTGGACCGTGTCTCAGTTCCAGTGTGGCCGATCCACCCTCTCAGGGTCGGCTACGCATCGTTGCCTTGGTAAGCCGTTACCTTACCAACTAGCTAATGCGGCGCGGATCCATCTATAAGTGAC
>n.538.6.bb_1172
GTAGTTAGCCGCGGCTTTCTGATTAGGTACCGTCAAGACGTGCATAGTTACTTACACATTTATTCTTCCCTAATAACAGAGTTTTACGATTCCGAAGACCTTCATCACTCACGCGGCGTTGCTCCGTCAGGCTTTCGCCCATTGCGGAAGATTCCCTACTGCTGCCTCCCGTAGGAGTCTGGACCGTGTCTCAGTTCCAGTGTGGCCGATCACCCTCTCAGGGTCGGCTACGCATCGTTGCCTTGGTAAGCCGTTACCTTACCAACTAGCTAATGCGGCGCGGATCC
>n.538.6.bb_1173
GTAGTTAGCCGTGGCTTTCTGATTAGGTACCGTCAAGACGTGCATAGTTACTTACACATTTGTTCTTCCCTAATAACAGAGTTTTACGATCCGGAGACCTTCATCACTCACGCGGCGTTGCTCCGTCAGGCTTTCGCCCATTGCGGAAGATTCCCTACTGCTGCCTCCGTAGGAGTCTGGACCGTGTCTCAGTTCCAGTGTGGCCGAT
>n.538.6.bb_1174
[truncated: 102,710,584 more chars]
