# Supplementary figures and images for: Intestinal dysbiosis in preterm infants preceding necrotizing enterocolitis: a systematic review and meta-analysis
Source: Microbiome. 2017 Mar 9;5:31. doi: 10.1186/s40168-017-0248-8 (PMC5343300; doi:10.1186/s40168-017-0248-8)

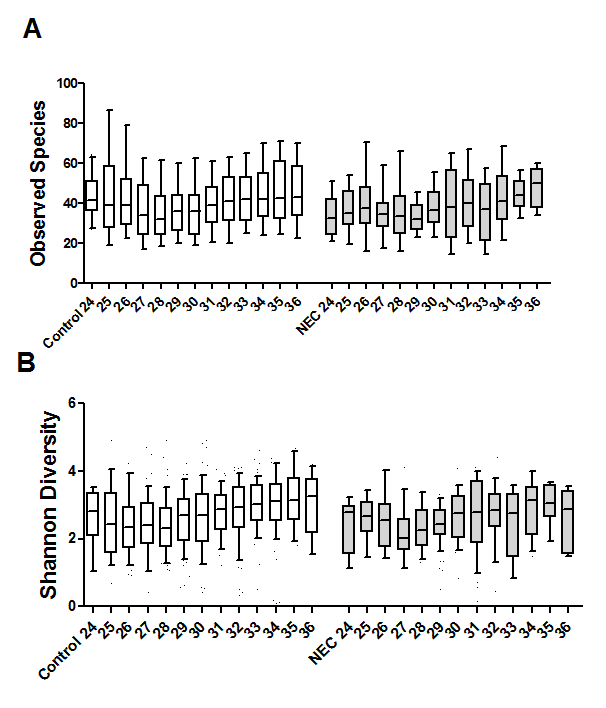

Supplement: Additional file 6: — Alpha diversity trends over corrected gestational age alpha-diversity metrics (mean with SD) by corrected gestational age (weeks). Fig. A depicts observed species (in operational taxonomic units, OTUs) and Fig. B depicts Shannon diversity index (SDI) against corrected gestational age (CGA) in controls and in infants with necrotizing enterocolitis (NEC). Data is represented in box and whisker plots with median and whiskers representing 10–90th centiles. The results of a fitted negative binomial regression model showed that the number of species increased significantly with gestational age (p < 0.001), and NEC patients tended to have fewer species than controls after controlling for gestational age (p = 0.053). (TIF 39 kb) [file 40168_2017_248_MOESM6_ESM.tif]
